# The uncharacterized SANT and BTB domain-containing protein SANBR inhibits class switch recombination

**DOI:** 10.1016/j.jbc.2021.100625

**Published:** 2021-04-06

**Authors:** Simin Zheng, Allysia J. Matthews, Numa Rahman, Kayleigh Herrick-Reynolds, Emily Sible, Jee Eun Choi, Alec Wishnie, Yan Kee Ng, Daniela Rhodes, Stephen J. Elledge, Bao Q. Vuong

**Affiliations:** 1 Department of Microbiology, Icahn School of Medicine at Mount Sinai, New York, New York, USA; 2 NTU Institute of Structural Biology, Nanyang Technological University, Singapore, Singapore; 3 Yale School of Medicine, Yale University, New Haven, Connecticut, USA; 4 Department of Biology, The Graduate Center and The City College of New York, New York, New York, USA; 5 Immunology Program, Memorial Sloan Kettering Cancer Center, New York, New York, USA; 6 Department of Genetics, Program in Virology, Howard Hughes Medical Institute, Harvard Medical School, Boston, Massachusetts, USA; 7 Division of Genetics, Department of Medicine, Brigham and Women's Hospital, Boston, Massachusetts, USA

**Keywords:** DNA recombination, B cells, immunoglobulin diversification, corepressor, AID, activation-induced cytidine deaminase, APE, apurinic/apyrimidinic endonuclease, BER, base excision repair, ChIP, chromatin immunoprecipitation, CSR, class switch recombination, DSB, double-strand break, EXO1, Exonuclease I, GST, glutathione-S-transferase, HDAC, histone deacetylase, Ig, immunoglobulin, MMR, mismatch repair, N-CoR, nuclear corepressor, SANBR, SANT and BTB domain regulator of CSR, SEC, size-exclusion chromatography, SMRT, silencing mediator of retinoic acid and thyroid hormone receptor, UNG, uracil DNA glycosylase, XRCC4, X-ray repair cross complementing 4

## Abstract

Class switch recombination (CSR) is the process by which B cells switch production from IgM/IgD to other immunoglobulin isotypes, enabling them to mount an effective immune response against pathogens. Timely resolution of CSR prevents damage due to an uncontrolled and prolonged immune response. While many positive regulators of CSR have been described, negative regulators of CSR are relatively unknown. Using an shRNA library screen targeting more than 28,000 genes in a mouse B cell line, we have identified a novel, uncharacterized protein of 82kD (KIAA1841, NM_027860), which we have named SANBR (SANT and BTB domain regulator of CSR), as a negative regulator of CSR. The purified, recombinant BTB domain of SANBR exhibited characteristic properties such as homodimerization and interaction with corepressor proteins, including HDAC and SMRT. Overexpression of SANBR inhibited CSR in primary mouse splenic B cells, and inhibition of CSR is dependent on the BTB domain while the SANT domain is largely dispensable. Thus, we have identified a new member of the BTB family that serves as a negative regulator of CSR. Future investigations to identify transcriptional targets of SANBR in B cells will reveal further insights into the specific mechanisms by which SANBR regulates CSR as well as fundamental gene regulatory activities of this protein.

Immunoglobulin (Ig) production by B cells is an essential component of the immune response against pathogens and malignancies. Upon encounter with antigen, mature B cells are activated and undergo class switch recombination (CSR) (1, 2, 3). This process changes the isotype of the Ig produced from IgM to IgG, IgE or IgA, and thus couples Ig specificity, which is determined by the variable domains, to a broad spectrum of effector functions for complete humoral immunity. Through a DNA rearrangement reaction, CSR replaces the default constant region Cμ in the Ig heavy chain (IgH) locus with one of the downstream constant regions (Cγ, Cε, or Cα) that codes for the new isotype. Following stimulation for CSR, B cells express the enzyme activation-induced cytidine deaminase (AID), which localizes to repetitive GC-rich switch (S) regions that precede each constant region (4, 5). AID deamination of the S regions initiates the formation of DNA double-strand breaks (DSBs) in Sμ and a downstream acceptor S region to delete the intervening DNA and rearrange the IgH locus to express a different Ig isotype.

Following AID deamination of S region DNA, two complementary pathways, base excision repair (BER) and mismatch repair (MMR), generate the DSBs that are necessary for CSR. In BER, uracil DNA glycosylase (UNG) removes the AID-generated uracil base to create an abasic site, which is processed into a DNA break by apurinic/apyrimidinic endonucleases (APE) (6, 7). During MMR, a complex of proteins, which includes PMS1 homolog 2 (PMS2) and Exonuclease I (EXO1), converts AID-induced U:G mismatches into DSBs (8). Proteins of the nonhomologous end-joining pathway, such as Ku70, Ku80, X-ray repair cross complementing 4 (XRCC4) and Ligase IV (Lig4), ligate DSBs in donor, and acceptor S regions together to complete CSR (1). Similarly, other factors positively regulate CSR by modulating AID expression, targeting AID to S regions, generating DSBs, and repairing DSBs (1, 3, 9, 10, 11, 12).

Chromatin architecture also influences CSR, and proteins that regulate epigenetic states have been reported to promote CSR. For example, loss of the PTIP protein decreased histone methylation and chromatin accessibility, altered germline transcription, and resulted in defects in CSR (13, 14). Phosphorylation and acetylation of histone H3 modulate AID recruitment to S regions by 14-3-3 and conditional inactivation of methyltransferases (*Suv4-20h1* and *Suv4-20h2*), which modify histone H4, in B cells significantly impairs CSR (15, 16).

In contrast, few negative regulators of CSR have been described. Previously characterized negative regulators include the poly(ADP) ribose polymerase Parp3 (17) and the aryl hydrocarbon receptor AhR (18), with many more yet to be uncovered. Parp3 prevents excessive accumulation of AID at S regions (17), while AhR limits AID expression (18). These negative regulators of CSR fine-tune humoral immune responses and AID-mediated DNA damage, which if dysregulated can lead to autoimmunity, genomic instability, and lymphomagenesis (19, 20, 21, 22, 23). To identify novel negative regulators of CSR, we performed an shRNA library screen in the CH12 mouse B cell line. Here, we report the identification and characterization of SANBR, a novel SANT- and BTB-domain containing protein as a negative regulator of CSR.

## Results

### Identification of SANBR as a negative regulator of CSR

**Figure 1:**
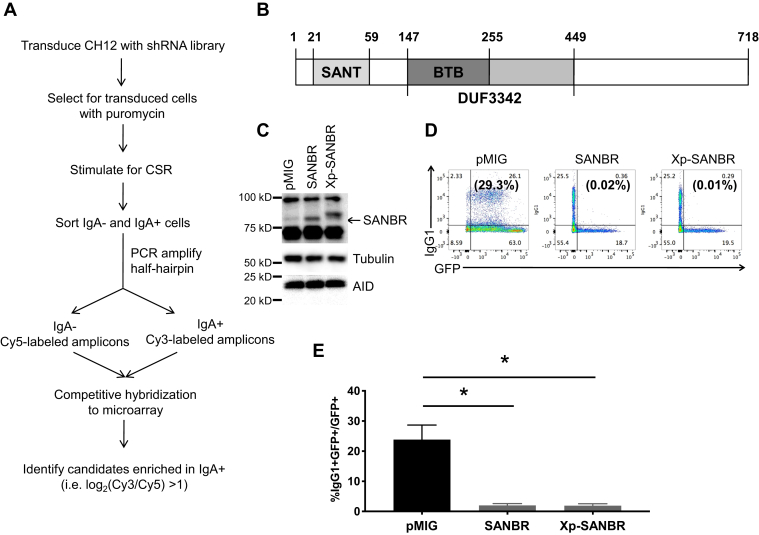
**shRNA screen identifies SANBR as a negative regulator of CSR.***A*, workflow for the shRNA screen. CH12 cells were infected with the lentiviral shRNA library, selected with puromycin, and stimulated to undergo CSR to IgA. IgA– and IgA+ cells were sorted and differentially labeled half-hairpin amplicons were generated by PCR using genomic DNA (Cy5 or Cy3 respectively). Relative abundance of individual shRNAs was determined by competitive hybridization to microarray using labeled half-hairpin amplicons. Potential negative regulators of CSR were identified as genes with at least twofold enrichment of targeting shRNA in the IgA+ population as compared with the IgA-population (log2(Cy3/Cy5) > 1). *B*, schematic representation of the domains of SANBR. *C–E*, expression of SANBR inhibits CSR. Splenic B cells were isolated from wild-type mice, stimulated for CSR with LPS+IL4, and transduced with retroviral vector control (pMIG) or vectors expressing untagged or Xpress-tagged (Xp) SANBR. *C*, overexpression of SANBR and expression of AID were determined by immunoblot. Tubulin was used as a loading control. *D*, CSR to IgG1 among the transduced cell population was determined by flow cytometry. A representative experiment is shown. The numbers in the corners of each plot indicate the percentage of cells in each quadrant while the numbers in parentheses indicate the percentage of IgG1+ cells within the GFP+ gate. *E*, the mean %IgG1+ within the GFP+ gate from three independent experiments ± SD is shown. ∗*p* < 0.05, two-tailed paired Student’s *t*-test.

To identify novel negative regulators of CSR, we performed an shRNA screen using the CH12 mouse B lymphoma cell line (Fig. 1*A*). CH12 cells are a well-established model system for the study of CSR, as they can be induced to class switch from IgM to IgA in culture when stimulated with anti-CD40, interleukin 4 (IL4), and transforming growth factor β (TGF-β), which is henceforth referred to as CIT (5, 18, 24, 25, 26, 27). CH12 cells were infected with a lentiviral shRNA library comprised of 67,676 shRNAs targeting 28,801 genes in the mouse genome (28, 29). Transduced cells were selected with puromycin and stimulated with CIT to undergo CSR to IgA. IgA- and IgA+ populations were sorted by flow cytometry and harvested for genomic DNA. The genomic DNA was used as the template in PCRs to generate fluorescence-labeled half-hairpin amplicons; IgA-amplicons were labeled with Cy5 while IgA+ amplicons were labeled with Cy3. The relative abundance of individual shRNAs was determined by competitive hybridization to custom microarrays (Fig. 1*A*). As depletion of negative regulators would likely promote CSR, we expected shRNAs that targeted them to be enriched in the IgA+ population as compared with the IgA– population. Genes with shRNAs showing at least twofold enrichment in IgA+ compared with IgA– samples, *i.e.*, log_2_(Cy3/Cy5) > 1, were identified as negative regulators (Table S1). As controls in the screen, known positive regulators of CSR resulted in negative log_2_(Cy3/Cy5) values, indicating that shRNAs for these genes are enriched in the IgA– population and their knockdown prevented CSR (Table S2). For instance, AID had a log_2_(Cy3/Cy5) of -1.42, demonstrating the validity of the shRNA screen (Fig. S1*A* and Table S2). Analysis of genes with more than a twofold difference in hybridization signals suggests that these candidates are associated with top canonical pathways, such as IL4 and cytokine signaling, which are relevant for CSR (Fig. S1*B*).

The shRNA screen identified an uncharacterized mRNA, NM_027860 (RIKEN cDNA 0610010F05 gene, 0610010F05Rik), as a candidate negative regulator of CSR based on our selection criteria of at least one targeting shRNA with log2(Cy3/Cy5) > 1 at 10% FDR (29). The log_2_(Cy3/Cy5) values for the two shRNAs in the screen targeting NM_027860 were 3.09 and –0.92. NM_027860 encodes for a well-conserved protein KIAA1841 (Fig. S2) that is expressed broadly in many cell types (30, 31). An NCBI genome database search predicts that invertebrates (*e.g.*, *Drosophila melanogaster* CG6761), vertebrates (*e.g.*, *Homo sapiens*, *Danio rerio*), and single-cell organisms (*e.g.*, Trypanosoma grayi, *Ruminococcus albus* 7) express an ortholog of KIAA1841. The mouse KIAA1841 gene is located on chromosome 11 and consists of 28 exons. While a potential splice isoform missing exons 3, 4, and 5, which encodes the first 146 amino acids, has been reported (32, 33), we were unable to detect this isoform in mouse splenic B cells (data not shown). We cloned the full-length KIAA1841 cDNA from stimulated mouse splenic B cells. Full-length KIAA1841 comprises of 718 amino acids with a predicted molecular weight of 82 kD (Fig. 1*B*). Amino acid sequence alignment by BLAST and structure prediction by Phyre2 (34) further revealed that this protein has a putative SANT domain (“switching-defective protein 3 (Swi3), adaptor 2 (Ada2), nuclear receptor corepressor (N-CoR), transcription factor (TF)IIIB”) encoded by amino acids 21–59, and a BTB domain (“broad-complex, tramtrack and bric-a-brac”) encoded by amino acids 147–255. Thus, we have named this protein SANBR (SANT and BTB domain regulator of CSR). The SANBR BTB domain overlaps a conserved domain of unknown function (DUF3342, pfam11822), which includes amino acids 147–449 (Fig. 1*B*). Although SANBR was not detected at S regions by chromatin immunoprecipitation (Fig. S3), SANBR expression was significantly increased and sustained in mouse splenic B cells upon stimulation with anti-CD40 plus IL4 or LPS plus IL4 (Fig. S4). Wild-type and AID^−/−^ B cells upregulated SANBR mRNA expression comparably, indicating that the expression of SANBR is independent of AID (Fig. S4).

To validate the identification of SANBR as a negative regulator of CSR, we retrovirally overexpressed untagged and Xpress epitope-tagged SANBR in LPS+IL4-stimulated mouse splenic B cells (Fig. 1*C*). Both the untagged and Xpress-tagged SANBR significantly reduced CSR to IgG1 (Fig. 1, *D* and *E*), reinforcing the role of SANBR as a negative regulator of CSR. Overexpression of SANBR did not affect germline transcription (Fig. S5), AID mRNA and protein expression (Figs. S5 and 1*C*), or B cell proliferation (Fig. S6). Thus, overexpression of SANBR inhibits CSR without affecting fundamental processes that are required for CSR.

### Putative BTB domain of SANBR mediates dimerization

**Figure 2:**
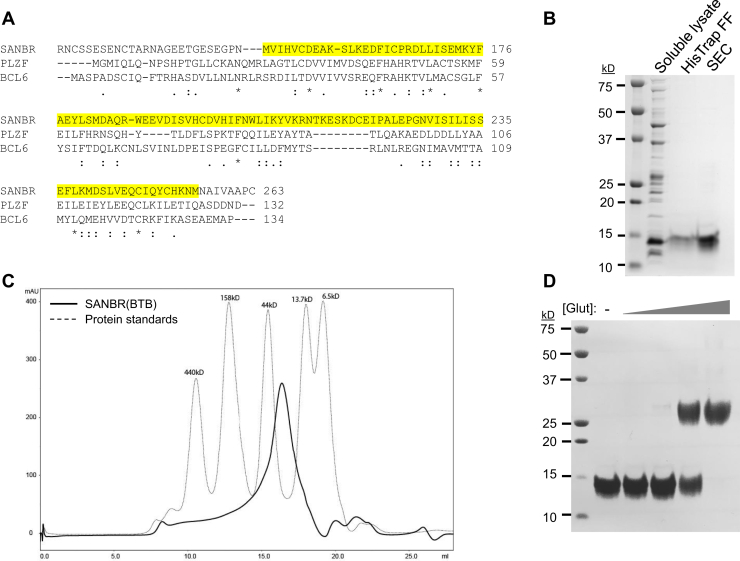
**The BTB domain of SANBR dimerizes *in vitro*.***A*, sequence alignment of the putative BTB domain of SANBR with the BTB domains of PLZF and BCL6. Alignment was performed using ClustalOmega; ∗, conserved residue, :, strongly similar residues, ., weakly similar residues. The BTB domain of SANBR (amino acids 147–255) that was predicted by Phyre2 protein fold recognition software is highlighted in *yellow*. Numbers indicate amino acid residues in each sequence. *B*, purification of recombinant BTB domain of SANBR, SANBR(BTB). The predicted BTB domain of SANBR (amino acids 144–261) was purified as a His6-tagged protein by HisTrap FF column and size-exclusion chromatography (SEC). A Coomassie-stained gel is shown. The predicted size of His6-SANBR(BTB) is 15kD. *C*, SEC analysis of purified SANBR(BTB). SEC was performed using Superdex-200 10/30 column. The chromatogram of molecular weight standards is indicated by the *dotted line*. *D*, glutaraldehyde cross-linking of purified SANBR(BTB). Purified SANBR(BTB) was treated with fivefold increasing concentrations of glutaraldehyde (Glut), before analysis by SDS-PAGE and Coomassie stain.

We next investigated the function of the putative BTB domain of SANBR. Many cellular processes require BTB domain-containing proteins, including transcription and protein degradation (35). In the immune system, several developmental pathways are controlled by BTB-containing proteins. For instance, promyelocytic leukemia zinc finger (PLZF) controls the development of NKT cells (36, 37), and B cell lymphoma 6 (BCL6) is essential for the formation and function of follicular helper T cells (T_FH_) and germinal center B cells (38, 39). The BTB domain is a well-characterized structural motif that mediates dimerization (35). The putative BTB domain of SANBR identified by Phyre2 is well conserved with the BTB domains of PLZF and BCL6 (Fig. 2*A*). As full-length SANBR is poorly expressed and aggregated in *Escherichia coli* (data not shown), a fragment containing the putative BTB domain, SANBR(BTB), was expressed as a His_6_-tagged recombinant protein and purified (Fig. 2*B*) before analysis on a size-exclusion chromatography (SEC) column (Fig. 2*C*). Comparison with protein molecular weight standards separated on the column under the same conditions indicated that SANBR(BTB) migrates as a dimer (Fig. 2*C*). To further verify that SANBR(BTB) dimerizes, we cross-linked purified SANBR(BTB) with glutaraldehyde before analysis on a denaturing SDS-PAGE (Fig. 2*D*). When cross-linked, the 15kD fragment of SANBR(BTB) formed a species approximately 30kD in size, corresponding to the expected molecular weight of a dimer (Fig. 2*D*). Thus, SANBR contains a BTB domain that dimerizes *in vitro*.

**Figure 3:**
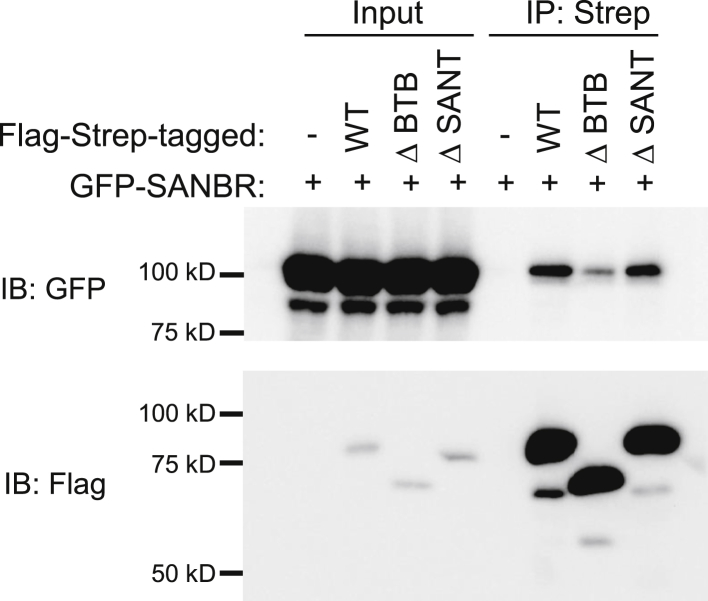
**The BTB domain of SANBR facilitates dimerization *in vivo.*** 293T cells were cotransfected with GFP-tagged SANBR and Flag-Strep-tagged wild-type (WT), ΔBTB, or ΔSANT SANBR. Flag-Strep-tagged proteins were pulled down using Strep-Tactin XT beads and bound proteins were analyzed by immunoblot using anti-GFP and anti-Flag antibodies. The results are representative of three independent pull-down experiments.

To determine if the BTB domain of SANBR promotes dimerization *in vivo*, dimerization assays were performed in 293T cells. Full-length Flag-StrepII-tagged wild-type (WT) or deletion mutants of SANBR, which lacked the BTB domain (ΔBTB, amino acids 147–255), or helix 3 of the SANT domain (40) (ΔSANT, amino acids 48–59) (Fig. S7), were coexpressed with N-terminal GFP-tagged WT SANBR (GFP-SANBR) in 293T cells (Fig. 3). Flag-StrepII-SANBR(WT) and the SANBR deletion mutant proteins were isolated with Strep-Tactin XT beads and examined for their ability to interact with GFP-SANBR (Fig. 3). Unlike WT and ΔSANT, the ΔBTB mutant protein showed reduced binding to GFP-SANBR (Fig. 3), demonstrating that the BTB domain mediates dimerization of SANBR *in vivo*. The binding of the ΔSANT mutant protein to GFP-SANBR indicates that the SANT domain is not required for dimerization of SANBR (Fig. 3), and its function in SANBR remains to be determined.

### SANBR interacts with corepressors through its putative BTB domain

**Figure 4:**
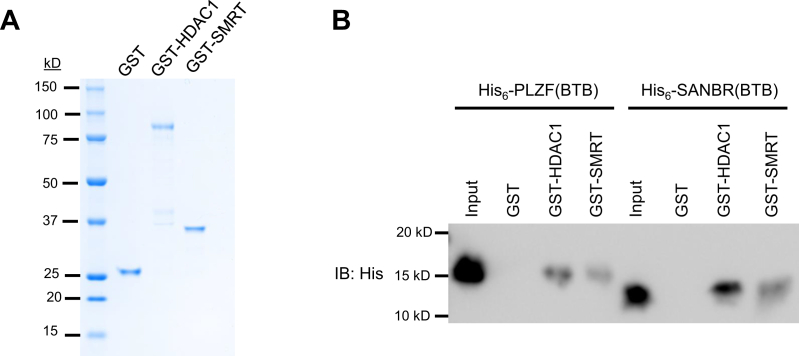
**The BTB domain of SANBR interacts with corepressors.***A*, recombinant GST, GST-HDAC1, and GST-SMRT fragments were purified and analyzed by Coomassie stain. *B*, purified GST, GST-HDAC1, and GST-SMRT fragments were incubated with equimolar purified His-tagged PLZF(BTB) or SANBR(BTB), followed by GST pull-down and analysis by immunoblot (IB) using anti-His antibody. The results are representative of three independent pull-down experiments.

BTB-containing proteins commonly function by interacting with corepressors, such as histone deacetylases (HDACs), nuclear corepressors (N-CoR), and silencing mediator of retinoic acid and thyroid hormone receptor (SMRT), *via* their BTB domain (35). To determine if the BTB domain of SANBR can bind to corepressors, we purified recombinant glutathione-S-transferase (GST) GST-tagged HDAC1 and a fragment of SMRT previously reported to interact with the BTB domain of PLZF (41) (Fig. 4*A*). These proteins were then used in binding assays with purified His_6_-tagged SANBR(BTB), where a His_6_-tagged BTB domain of PLZF was used as a positive control. Consistent with a previous report (41), His_6_-PLZF(BTB) interacted with HDAC1 and the SMRT fragment (Fig. 4*B*). Similarly, His_6_-SANBR(BTB) also bound to these corepressor proteins (Fig. 4*B*). Taken together with the earlier dimerization findings, these data suggest that SANBR contains a functional BTB domain that mediates dimer formation and binding to corepressors.

### The BTB domain of SANBR is important for the inhibition of CSR

**Figure 5:**
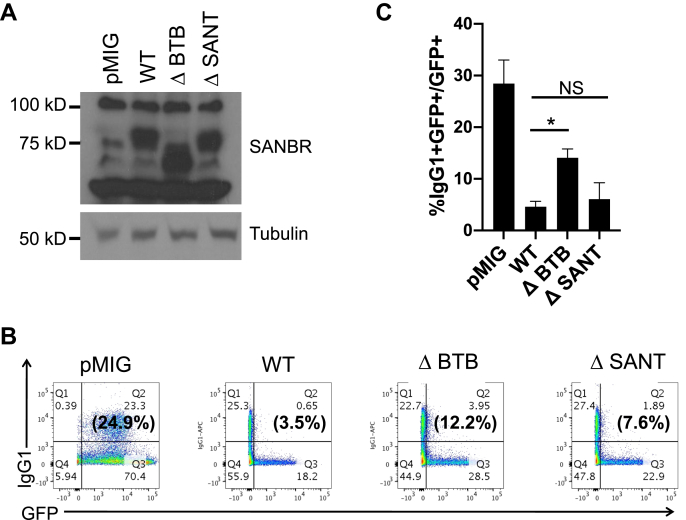
**The BTB domain of SANBR is necessary for inhibiting CSR.** Splenic B cells were isolated from mice, stimulated with LPS+IL4, and transduced with retroviral vector control (pMIG) or vectors expressing wild-type (WT), ΔBTB, or ΔSANT SANBR. *A*, expressions of SANBR and mutants were determined by immunoblot with SANBR antibodies. Tubulin was used as a loading control. *B*, CSR to IgG1 among the transduced GFP+ cells was determined by flow cytometry. A representative experiment is shown. The numbers in the corners of each plot indicate the percentage of cells in each quadrant while the numbers in parentheses indicate the percentage of IgG1+ cells within the GFP+ gate. *C*, the mean %IgG1+ within the GFP+ gate from three independent experiments ± SD is shown. ∗*p* < 0.05. NS, *p* = not significant, *p* ≥ 0.05, two-tailed paired Student’s *t*-test.

To evaluate the role of the SANBR BTB and SANT domains in the regulation of CSR, Flag-StrepII-tagged ΔBTB or ΔSANT deletion mutants were expressed in mouse splenic B cells that were stimulated for CSR to IgG1 by LPS plus IL4. As shown earlier (Fig. 1, *D* and *E*), retroviral expression of WT SANBR protein (Fig. 5*A*) reduced CSR to IgG1 in splenic B cells (Fig. 5, *B* and *C*). While the ΔSANT mutant inhibited CSR to similar levels as WT SANBR, the ΔBTB mutant only partially suppressed CSR (Fig. 5, *B* and *C*). This suggests that the BTB domain of SANBR plays an important role in the negative regulation of CSR, while the SANT domain is largely dispensable. As WT levels of CSR were not achieved with ΔBTB mutant expression, other regions of SANBR likely function in tandem with the BTB domain to mediate the suppression of CSR.

## Discussion

Our study has identified a novel SANT- and BTB-domain containing protein, SANBR, that inhibits CSR. The predicted BTB domain of SANBR displays characteristic properties of BTB domains, including homodimerization (Figs. 2, 3) as well as interaction with corepressor proteins *in vitro* (Fig. 4). To the best of our knowledge, this represents the first report demonstrating this protein as a member of the BTB protein family.

BTB protein family members often serve as important transcriptional regulators that control many developmental processes (35). For instance, PLZF interacts with N-CoR, SMRT, and HDACs to mediate transcriptional repression (41) and plays an important role in the differentiation of NKT cells (36, 37), as well as the modulation of the inflammatory response in macrophages (42). Similarly, BCL6 interacts with the corepressor BCoR to control the function of B cells and T follicular helper cells in germinal centers (43, 44). While the mechanisms by which SANBR inhibits CSR are still unclear, its ability to interact with corepressor proteins such as HDAC1 and SMRT *via* the BTB domain leads us to hypothesize that SANBR acts as a transcriptional regulator in line with other BTB protein family members (Fig. 4). SANBR may recruit these corepressors to transcriptional targets to downregulate gene expression. We have shown that germline switch transcripts and AID mRNA levels are not directly regulated by SANBR overexpression (Fig. S5). The transcriptional targets of SANBR that mediate its inhibitory effects on CSR await further investigation.

While the BTB domain alone is sufficient for homodimerization *in vitro* (Fig. 2), other regions of SANBR contribute to dimer formation. Deletion of the BTB domain alone only partially impaired dimerization *in vivo* (Fig. 3) and resulted in a corresponding partial rescue of CSR inhibition when compared with the WT protein (Fig. 5, *B* and *C*). BTB domains can also mediate heterodimerization (45). These heterodimers may have distinct functions from homodimers, which adds another layer of regulation that can influence cellular fitness (46, 47). For instance, BCL6 heterodimerizes with Miz-1 *via* its BTB domain to inhibit expression of the Miz-1 target, cyclin-dependent kinase inhibitor p21, thereby allowing for proliferation of germinal center B cells (46). Thus, SANBR may similarly associate with BCL6, or other BTB protein family members, to inhibit CSR. As SANBR could not be detected at the S regions (Fig. S3), the inhibitory effects of SANBR on CSR are unlikely due to direct activity on S region chromatin. Interestingly, SANBR is upregulated in an AID-independent manner in purified B cells that are stimulated for CSR (Fig. S4). We speculate that this increased SANBR expression promotes the inactivation of genes, which promote CSR. Given the role of BTB proteins in regulating transcription of genes that are required for immune cell development and function, additional genomic, transcriptomic, or proteomic studies will identify the specific mechanism by which SANBR regulates CSR.

Although the SANT domain is dispensable for dimerization (Fig. 3) and inhibition of CSR (Fig. 5), it may be involved in other non-CSR functions of SANBR. SANT domains are often found in chromatin remodeling proteins and function by interacting with histone tails (40, 48, 49). In concert with corepressors recruited *via* the BTB domain, the SANBR SANT domain may modify chromatin, regulate gene transcription, and/or germinal center B cell development. These and other roles of SANBR beyond inhibition of CSR await further investigation.

## Experimental procedures

### Cell culture

Primary B cells were purified from spleens of WT male and female C57BL/6 mice (2–4 months old) and stimulated with LPS and IL4 to induce CSR to IgG1 as described (50). Retroviral infection of splenic B cells was performed as described (51). CH12 cells were maintained and stimulated with anti-CD40, IL4, and TGF- β to induce CSR to IgA as described (52). C57BL/6 mice were purchased from The Jackson Laboratory. Experiments using mice were conducted according to protocols approved by The City College of New York Institutional Animal Care and Use Committee.

### Antibodies

The polyclonal SANBR antibody was produced by immunizing rabbits with the C-terminal SANBR peptide (RSKSRFGQGRPA) and isolating reactive antibodies from sera (Covance). Antibodies for flow cytometry include: anti-IgG1-APC (X56, BD Pharmingen), anti-IgA-FITC (C10-3, BD Pharmingen). Antibodies for immunoblot include: anti-AID (53), anti-tubulin (DM1A, Sigma), anti-penta-His (34660, Qiagen), anti-GFP-HRP (B-2, Santa Cruz), anti-Flag (M2, Sigma). Antibodies for chromatin immunoprecipitation (ChIP) include: anti-H3 (ab1791, Abcam) and rabbit IgG (I5006, Sigma).

### shRNA library screen

The lentiviral shRNA library targeting the mouse genome was a kind gift from S.J. Elledge (28, 29). Screening for negative regulators of CSR was adapted from previous protocols (28, 29). Briefly, CH12 cells were infected with the lentiviral shRNA library. Successfully transduced cells were selected with 3 μg/ml puromycin for 72 h and subsequently stimulated to undergo CSR to IgA for 96 h. Cells were sorted into IgA− and IgA+ populations, and genomic DNA was prepared from the sorted cells. Differentially labeled (Cy5–IgA−; Cy3–IgA+) half hairpin amplicons were PCR amplified and allowed to undergo competitive hybridization onto microarrays (Agilent). Candidate negative regulators were identified as genes that are enriched in the IgA+ population at least twofold over the IgA-population; log_2_(Cy3/Cy5) > 1.

### Statistical analysis

Statistical analysis was performed as described previously (29). Briefly, intensity-dependent loess normalization in R generated normalized log_2_(Cy3/Cy5) ratios for the probes. Probes with a signal < twofold above background and a false discovery rate (FDR) of more than 10% were removed. Genes with at least one targeting shRNA that satisfy these criteria were identified as candidates.

### Chromatin immunoprecipitation (ChIP)

Splenic B cells were retrovirally transduced (51) with vector control (pMIG) or vector expressing SANBR (pMIG-SANBR) and fixed with 1% formaldehyde (Fisher, PI28908) at 48 h after the second retroviral transduction. Cells were lysed in SDS lysis buffer (50 mM Tris pH 8, 100 mM NaCl, 5 mM EDTA, 0.02% NaN_3_, 0.5% SDS) for 10 min at room temperature. Cells were isolated by centrifugation at 135*g* for 6 min and sonicated in IP buffer (30 mM Tris pH 8, 100 mM NaCl, 5 mM EDTA, 0.02% NaN3, 0.3% SDS, 1.7% TritonX-100) using Bioruptor Pico (Diagenode) for ten cycles (30 s on, 30 s off). Cell debris was removed by high-speed centrifugation, and lysates were precleared with Protein A agarose/Salmon Sperm beads (Sigma, 16-157). Cells (2.5 − 10^6^) were immunoprecipitated with each antibody at 4 °C overnight, and 5% was taken as input prior to immunoprecipitation. Immuno-complexes were captured with ProteinA agarose/Salmon Sperm beads and washed with wash buffer (50 mM HEPES-KOH, pH 7.6, 300 mM LiCl, 1 mM EDTA, 1% NP-40, 0.7% sodium deoxycholate), followed by reversal of cross-links at 65 °C overnight. DNA fragments were purified and quantified by qPCR using iQ SYBR Green Supermix (BioRad) with primers as described (51).

### Analysis of SANBR expression

WT and AID^−/−^ mouse splenic B cells were negatively isolated using magnetic beads (Miltenyi, anti-CD43) and stimulated with either anti-CD40 and IL4 or LPS plus IL4. Cells (1 − 10^6^) were harvested at 24 h, 48 h, 72 h or 96 h poststimulation. Unstimulated B cells (10 − 10^6^) were harvested at the time of isolation as 0 h. RNA was extracted from cells using TRIzol reagent (Invitrogen) and cDNA generated by reverse transcription (RT) using SuperScript III first-strand synthesis system (Invitrogen). SANBR mRNA level was determined by qPCR using cDNA and iQ SYBR Green Supermix (BioRad); SANBR RT-qPCR Cq values were normalized to β-actin mRNA Cq values and to the respective 0 h controls. qPCR primers used were as follows: SANBR (5′-CATTTCAGTTCATTGCGATGTT-3′, 5′-AAATGACATTCCCTGGCTCTAA-3′), β-actin (5′-TGCGTGACATCAAAGAGAAG-3′, 5′-CGGATGTCAACGTCACACTT-3′).

### Protein purification

Residues 144–261 of SANBR containing the BTB domain was expressed with an N-terminal His_6_-tag in BL21DE3(RIPL) *E. coli* and induced with 0.2 mM IPTG at 16 °C for 17–20 h. Cells were lysed in lysis buffer (20 mM Tris, pH 8.5; 0.5 M NaCl, 50 mM imidazole, 1 mM TCEP, 0.5 mg/ml benzonase, 1 mM PMSF, cOmplete protease inhibitor, 100 μg/ml lysozyme) using the LM20 Microfluidizer (Microfluidics). Lysates were clarified by centrifugation, and His_6_-SANBR(BTB) was isolated on a HisTrap FF column (GE Healthcare). The column was washed with ten column volumes (CVs) of lysis buffer, and bound His_6_-SANBR(BTB) protein was eluted with five CVs of elution buffer (20 mM Tris, pH8.5, 0.15 M NaCl, 100 mM imidazole, 1 mM TCEP). Fractions containing His_6_-SANBR(BTB) were pooled, concentrated, and further purified by SEC using Superdex 200 10/300 GL (GE Healthcare) equilibrated and eluted with SEC buffer (10 mM HEPES, pH 8.0; 0.3 M NaCl, 10% glycerol, 1 mM TCEP). The BTB domain of PLZF (41, 54), His_6_-PLZF(BTB), was expressed and purified as described above.

GST-tagged mouse HDAC1 and residues 1383–1467 of mouse SMRT were expressed in BL21DE3(RIPL) *E. coli* and induced with 0.2 mM IPTG at 16 °C for 17–20 h. This region corresponds to region 1414–1498 of human SMRT, which was previously reported to interact with BTB domain of PLZF (41). Cells were lysed in lysis buffer (20 mM Tris, pH 7.5; 0.5 M NaCl, 10% glycerol, 1 mM TCEP, 0.5 mg/ml benzonase, 1 mM PMSF, cOmplete protease inhibitor, 100ug/ml lysozyme) using the LM20 Microfluidizer (Microfluidics). Lysates were clarified by centrifugation, and GST-tagged proteins were isolated on glutathione sepharose 4B resin (GE Healthcare) at 4 °C for 2 h. The resin was washed with 30 CVs of lysis buffer and bound proteins eluted with elution buffer (50 mM Tris pH 7.5, 0.15 M NaCl, 10% glycerol, 15 mM glutathione, 1 mM TCEP). Fractions containing GST-HDAC1 and GST-SMRT(1383–1467) were pooled, concentrated, and further purified on S200 10/30 GL (GE Healthcare) using (10 mM HEPES, pH7.5, 0.3 M NaCl, 10% glycerol, 1 mM TCEP).

### Glutaraldehyde cross-linking

Purified His_6_-SANBR(BTB) protein at 0.1 mg/ml in sample buffer (10 mM HEPES, pH 8.0; 0.3 M NaCl, 10% glycerol, 1 mM TCEP) was cross-linked with fivefold increasing concentration of glutaraldehyde (*i.e.*, 0.0008%, 0.0004%, 0.02%, 0.1% (v/v)) in a total volume of 10 μl at 37 °C for 5 min. The reaction was quenched by adding 1 μl of 1 M Tris, pH 8.0 and boiled in 1× SDS loading buffer, before separation on a 12% SDS-PAGE denaturing gel and stained with Coomassie blue to visualize the migration of the resultant protein species.

### *In vitro* corepressor binding assay

Purified His_6_-SANBR(BTB) or His_6_-PLZF(BTB) at 200 nM was mixed with equimolar amounts of purified GST, GST-HDAC1, or GST-SMRT(1383–1467) fragment in binding buffer (50 mM HEPES pH 8.0, 150 mM NaCl, 0.1% IGEPAL-CA630, 2 mM EDTA), in a total volume of 400 μl, at 4 °C for 2 h. Glutathione sepharose 4B resin (GE Healthcare), which was prewashed thrice with binding buffer, was added and mixed with the proteins at 4 °C for an additional 2 h, then washed thrice with binding buffer. Bound complexes were released by boiling in 2× SDS loading buffer and analyzed by immunoblot with anti-His antibody. In total, 30 ng His_6_-SANBR(BTB) or 33 ng His_6_-PLZF(BTB) (*i.e.*, 5% of that used in the assay) were loaded as inputs.

### *In vivo* dimerization assay

293T cells were cotransfected with GFP-tagged SANBR and either Flag-Strep-tagged WT KIAA; SANBRΔBTB, which lacks amino acids 147–255 or SANBRΔSANT, which lacks amino acids 48–59. After transfection (48 h), cells from two confluent wells of a six-well plate were washed with PBS and lysed with 200 μl lysis buffer (20 mM Tris, pH 7.5, 150 mM NaCl, 5% glycerol, 0.5% NP40) supplemented with 1 mM PMSF, 1 mM DTT, and cOmplete mini protease inhibitor cocktail (Roche). Lysates were clarified by centrifugation at 21,000*g* for 15 min at 4 °C and incubated with 20 μl MagStrep “type 3” XT beads (IBA) for 2 h at 4 °C to pull down Flag-Strep-tagged SANBR and mutant proteins. The beads were washed thrice with lysis buffer and bound proteins were recovered by boiling in 1× SDS loading dye and analyzed by immunoblot.

## Data availability

Raw Cy3/Cy5 fluorescence data obtained from the custom microarray are available upon request to Bao Q. Vuong at The City College of New York (bvuong@ccny.cuny.edu). All remaining data are contained within the article.

## Supporting information

This article contains supporting information.

## Conflict of interest

The authors declare that they have no conflicts of interest with the contents of this article.
