# Supplementary material for: The uncharacterized SANT and BTB domain-containing protein SANBR inhibits class switch recombination
Source: J Biol Chem. 2021 Apr 6;296:100625. doi: 10.1016/j.jbc.2021.100625 (PMC8141524; doi:10.1016/j.jbc.2021.100625)
Supplement: Figures S1 to S7 and Tables S1 and S2 [file mmc1.pdf]

Supplemental figure and table legends

Supplementary Figure 1: Analysis of the shRNA screen.

Data from the shRNA screen was analyzed using Ingenuity Pathways Analysis software to identify candidate genes with at least one shRNA resulting in more than 2-fold change in log2(Cy3/Cy5) signals at a FDR rate of 10%. **(A)** Distribution of $\log_2$(Cy3/Cy5) signals from the shRNA screen. $\text{Log}_{2(}$Cy3/Cy5) values were divided into bins size of 0.2 and plotted against the number of shRNAs in each bin. Negative regulators (red), $\log_2$(Cy3/Cy5)>1, and positive regulators (green), $\log_2$(Cy3/Cy5)<-1, are highlighted. KIAA1841 (which we have named SANBR) and AID, along with their $\log_2$(Cy/Cy5) values, are indicated. **(B)** Top canonical pathways associated with candidate genes identified in the screen.

Supplementary Figure 2: KIAA1841 (i.e. SANBR) is well conserved among vertebrates.

NCBI HomoloGene pairwise alignment scores of human KIAA1841 sequence compared to other vertebrate orthologs.

Supplementary Figure 3: SANBR is not detected at S regions.

ChIP with anti-SANBR antibody was performed on mouse splenic B cells transduced with either retroviral vector control (pMIG) or vector expressing SANBR (pMIG-SANBR). Non-specific IgG and anti-H3 antibodies were used as negative and positive controls, respectively. S regions (Sμ and Sγ1), as well as non-switch control loci DNA (Cγ1 and p53) were amplified by quantitative real-time PCR (qPCR) and normalized to input DNA. The mean of three independent experiments +/- SD is shown.

Supplementary Figure 4: SANBR expression is increased upon stimulation for CSR.

Wild-type (WT) and $\text{AID}^{-/-}$ splenic B cells were isolated and stimulated for CSR with **(A)** anti-CD40 plus IL4, or **(B)** LPS plus IL4. Total RNA was extracted at the indicated times following stimulation and the level of SANBR transcripts was determined by reverse transcription (RT)-

qPCR. Data was normalized to β-actin mRNA and the respective 0h controls; the average +/- SD of three independent experiments is shown. *$p < 0.05$, when compared to respective 0h controls.

Supplementary Figure 5: μ-germline transcripts (GLT) and AID mRNA expression are not affected by overexpression of SANBR.

Splenic B cells were isolated from wild-type mice and transduced with retroviral vector control (pMIG) or a vector expressing SANBR. Levels of μ-GLT and AID mRNA were determined at 72h post-infection by RT-qPCR, and normalized to β-actin mRNA and the pMIG control. The mean of three independent experiments +/- SD is shown. NS (not significant), $p \geq 0.05$, two-tailed paired student's t-test.

Supplementary Figure 6: Overexpression of untagged and Xpress-tagged SANBR does not affect cell proliferation.

Splenic B cells were isolated from wild-type mice, stimulated with LPS+IL4, and transduced with retroviral vector control (pMIG) or a vector expressing SANBR or Xpress (Xp)-tagged SANBR. Cells were labeled with seminaphthorhodafluor (SNARF) cell tracking dye and SNARF intensity was measured at 0h, 24h, and 48h after retroviral infection. Histograms of transduced cells (GFP+) for pMIG control, SANBR and Xp-SANBR were superimposed at each time point. Three independent retroviral infection experiments are shown.

Supplementary Figure 7: Sequence alignment of the putative SANT domain of SANBR with the SANT domain of SWI3. Alignment was performed using ClustalOmega; *, conserved residue, :, strongly similar residues, ., weakly similar residues. Numbers indicate amino acid residues in each sequence. Helix 3, which has been reported to be important for the chromatin remodeling function of SWI3 (Boyer et. al., 2002), is well conserved in SANBR.

Supplementary Table 1: Negative regulators of CSR recovered by the shRNA screen. Genes with shRNAs showing at least 2-fold enrichment in IgA+ compared to IgA- samples, i.e. $\log_2$(Cy3/Cy5)>1, and FDR less than 10 were identified as negative regulators. The novel gene of interest, NM_027860, which encodes for the protein KIAA1841 (i.e. SANBR), is highlighted in yellow.

Supplementary Table 2: Positive regulators of CSR recovered by the shRNA screen. Genes with shRNAs showing at least 2-fold enrichment in IgA- compared to IgA+ samples, i.e. $\log_2$(Cy3/Cy5)<-1, and FDR less than 10, were identified as positive regulators. Known positive regulator, activation-induced cytidine deaminase (*Aicda*), is highlighted in green.

A

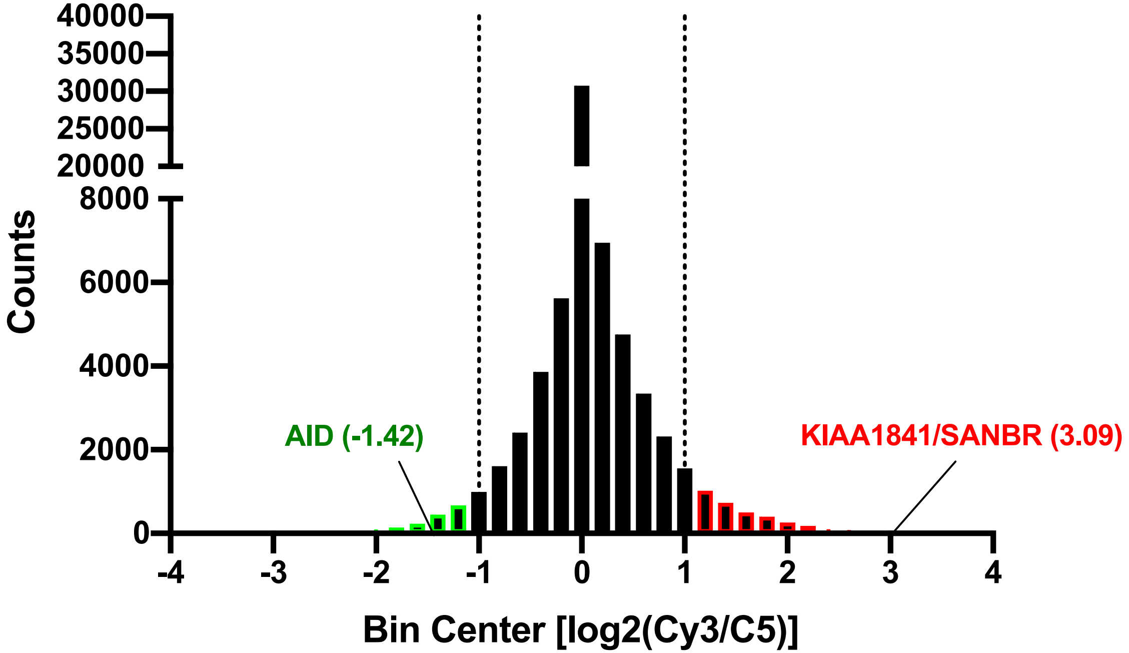

B

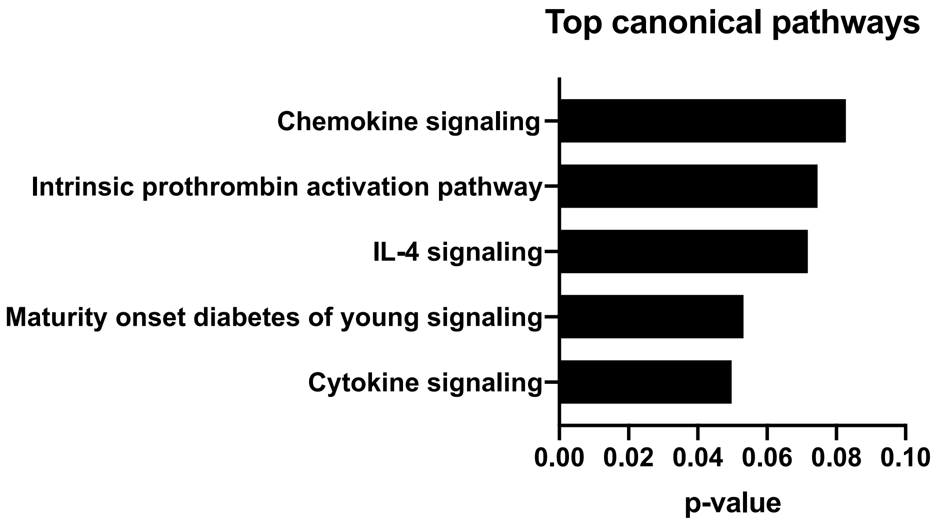

Supplementary Figure 1

Pairwise Alignment Scores

Gene		Identity (%)	
Species	Symbol	Protein	DNA
**H.sapiens**	**KIAA1841**		
vs. P.troglodytes	KIAA1841	99.2	99.5
vs. M.mulatta	LOC717910	97.8	97.7
vs. C.lupus	KIAA1841	92.3	92.2
vs. M.musculus	0610010F05Rik	88.6	88.2
vs. R.norvegicus	RGD1305110	88.4	87.2
vs. G.gallus	KIAA1841	75.9	77.1
vs. X.tropicalis	kiaa1841	70.9	70.4
vs. D.rerio	LOC793832	61.7	62.8

Supplementary Figure 2

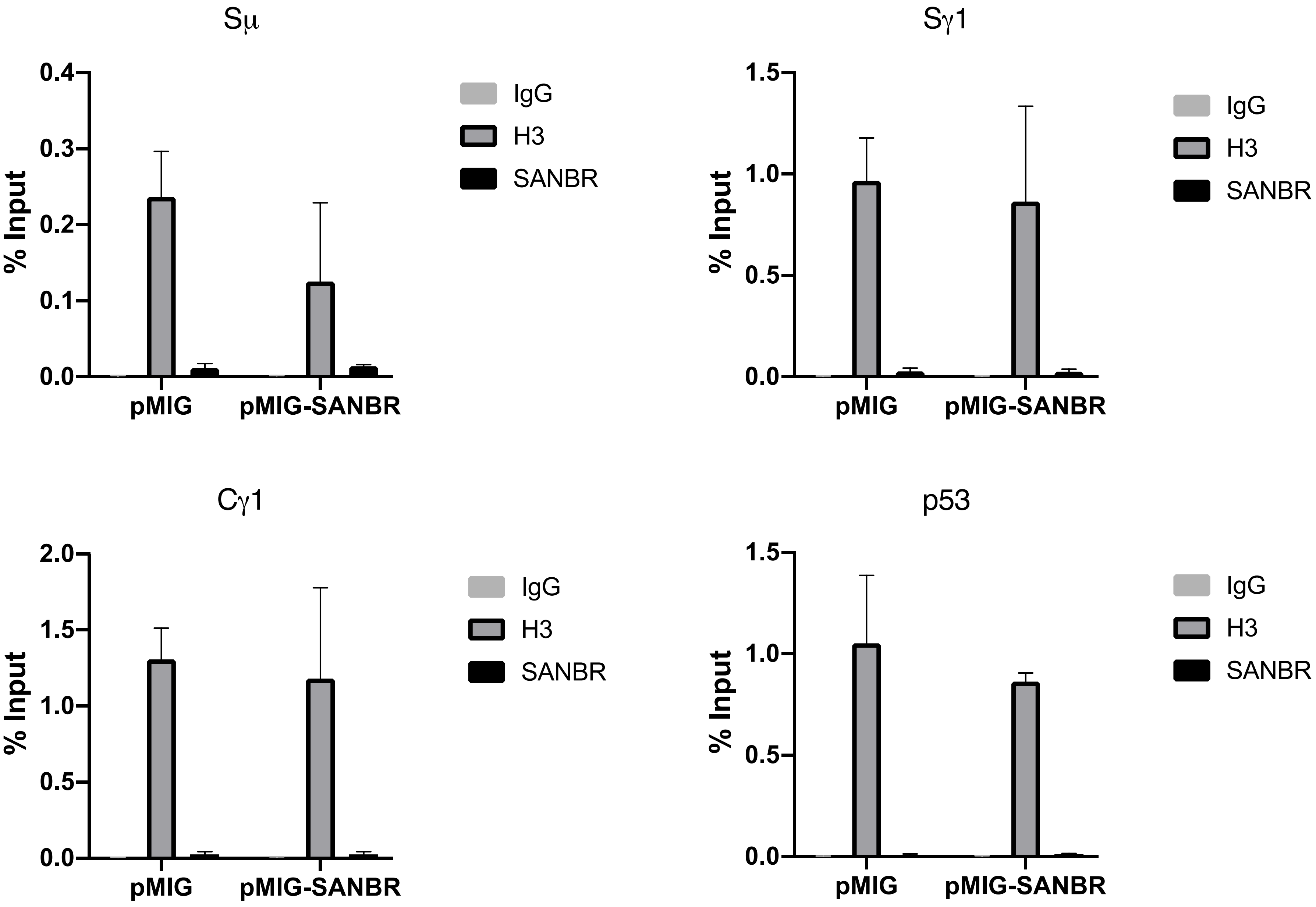

Supplementary Figure 3

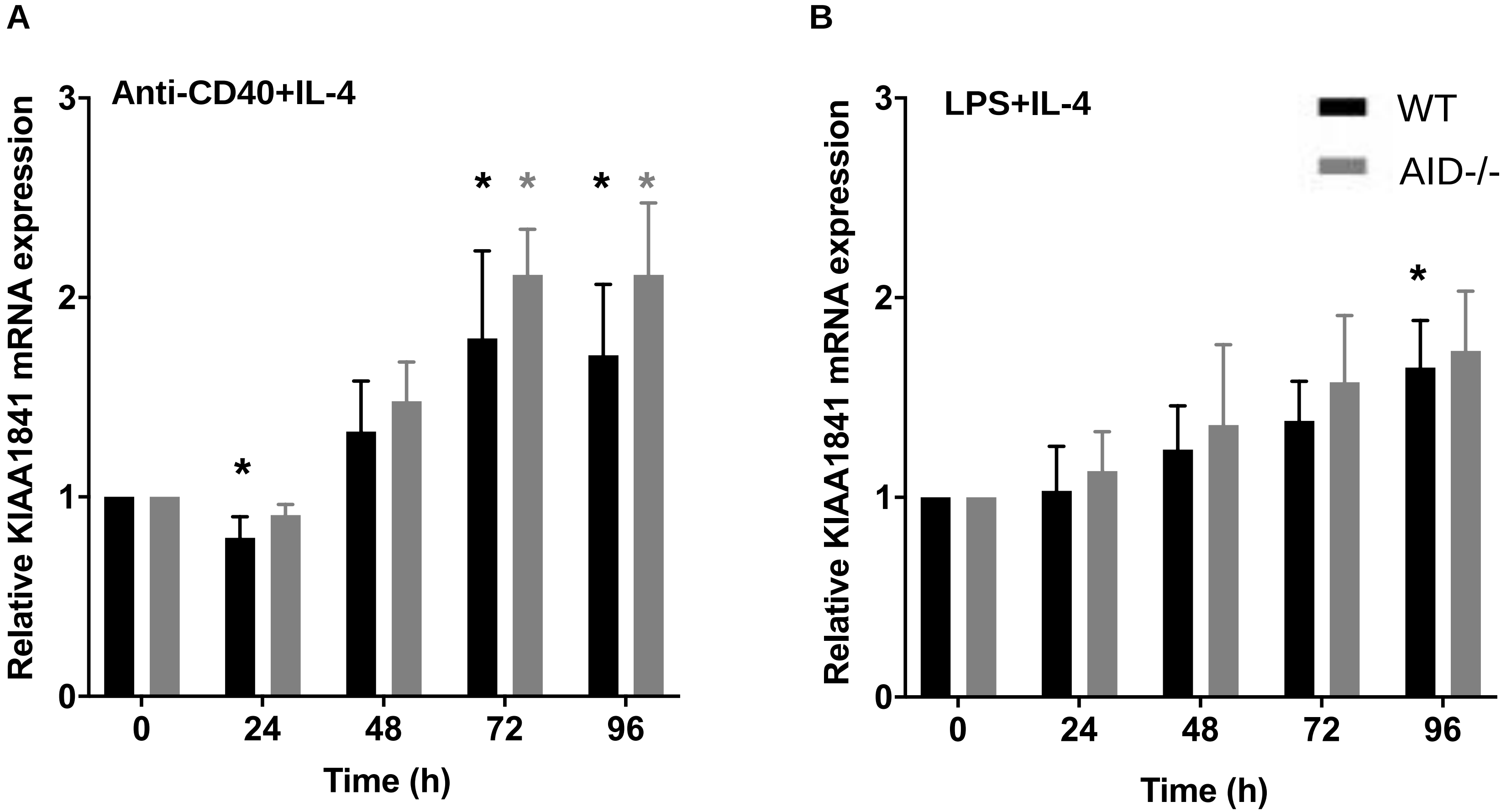

Supplementary Figure 4

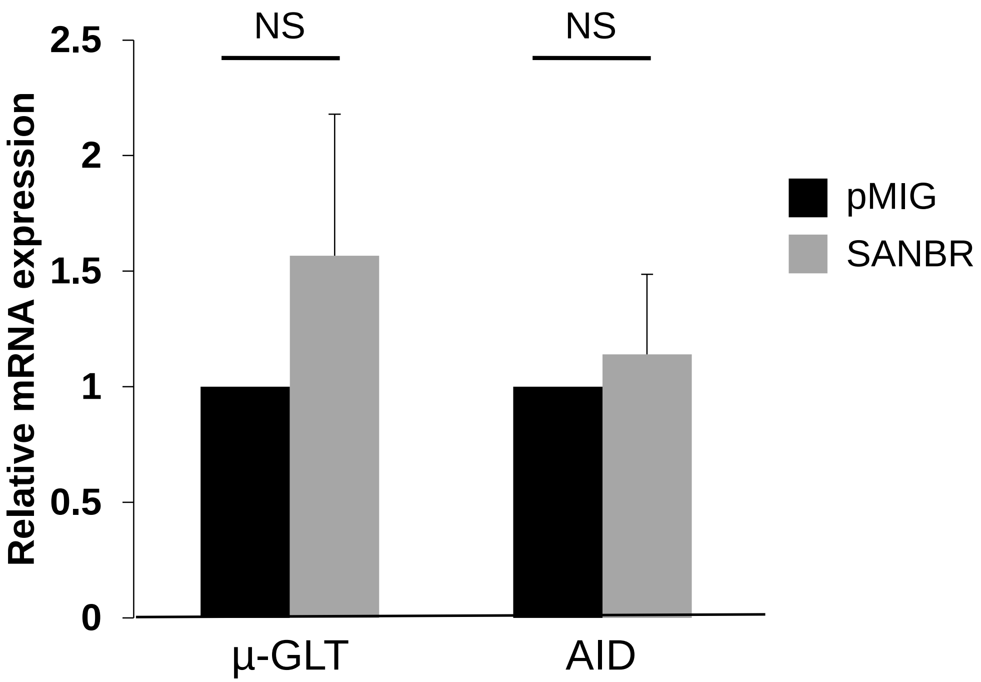

Supplementary Figure 5

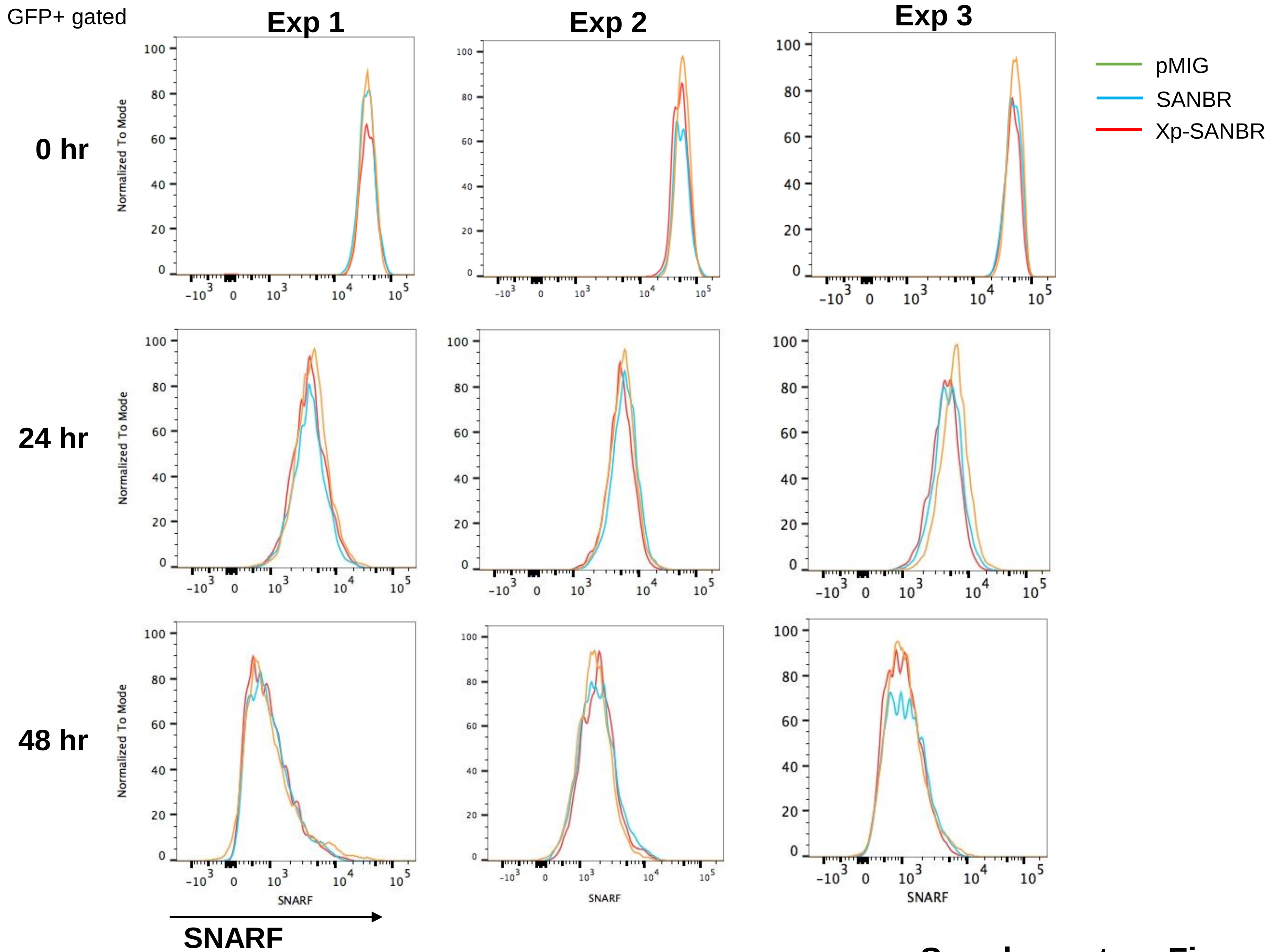

Supplementary Figure 6

```
SANBR  MVLDMILYPLIGI-PQTINWETVARLVPGLTPKECVKRFDELKS------ 60
SWI3      WSKEDLQKLLKGIQEFGADWYKVAKNVGNKSPEQCILRFLQLPIEDKFLY 576
             : :   * **     :* .**: * . :*::*: ** :*
```

Helix 3

Supplementary Figure 7

Supplementary Table 1: Negative regulators of CSR recovered by the shRNA screen

Gene.Name	log2.ratio	q.value...	Hit?	Codex_hair	Codex_97m	Codex_19m	Codex_Accession	Codex_gene.name	Codex_gene.symbol	OB_Release	OB_Plate	OB_Well_Row	OB_Well_Col	-log10(q/100)
092308m1_V2MM_141890	3.829646	3.004939	TRUE	HP_362603	TGCTGTTG	GCTTTATCC	XM_157111	Mus musculus LOC212310 (LOC212310), mR.		2.4	SM2050	h	12	1.522164293
mPool2_V2MM_133332	3.684535	3.004939	TRUE	HP_354073	TGCTGTTG	GATTCTCTT	XM_149195	RIKEN cDNA E230025N21 gene	E230025N21Rik	2.11	SM2373	e	7	1.522164293
mPool2_V2MM_2305	3.67322	2.205692	TRUE	HP_225926	TGCTGTTG	CTCCTAAA	XM_132611	similar to variable region of immunoglobulin kappa light chain		2.11	SM2381	h	4	1.656455229
mPool7_V2MM_141895	3.615101	3.004939	TRUE	HP_362608	TGCTGTTG	CGCTTTATC	XM_157111	Mus musculus LOC212310 (LOC212310), mRNA.		2.1	SM2329	a	8	1.522164293
mPool7_V2MM_88676	3.592123	2.205692	TRUE	HP_310113	TGCTGTTG	GTACAGAT	NM_175463	RIKEN cDNA A930012O16 gene	A930012O16Rik	2.9	SM2257	e	8	1.656455229
mPool7_V2MM_88676	3.592123	2.205692	TRUE	HP_310113	TGCTGTTG	GTACAGAT	NM_175463	RIKEN cD A930012O16 gene	A930012O16Rik	2.9	SM2257	e	8	1.656455229
mPool2_V2MM_145554	3.486776	3.004939	TRUE	HP_366265	TGCTGTTG	CATTCTTAT	XM_161053	Mus musculus LOC230170 (LOC230170), mR.		2.4	SM2040	e	9	1.522164293
mPool2_V2MM_145554	3.486776	3.004939	TRUE	HP_366265	TGCTGTTG	CATTCTTAT	XM_161053	Mus musculus LOC230170 (LOC230170), mRNA.		2.4	SM2040	e	9	1.522164293
mPool2_V2MM_92494	3.390594	3.004939	TRUE	HP_313842	TGCTGTTG	GTTGGAAT	NM_178720	RIKEN cDNA 9430016A21 gene	9430016A21Rik	2.6	SM2137	a	5	1.522164293
mPool2_V2MM_138848	3.381957	2.205692	TRUE	HP_359568	TGCTGTTG	GACATTAA	XM_154566	Mus musculus LOC238925 (LOC238925), mR.		2.4	SM2030	d	11	1.656455229
mPool2_V2MM_138848	3.381957	2.205692	TRUE	HP_359568	TGCTGTTG	GACATTAA	XM_154566	Mus musculus LOC238925 (LOC238925), mRNA.		2.4	SM2030	d	11	1.656455229
mPool2_V2MM_134275	3.329965	2.205692	TRUE	HP_355001	TGCTGTTG	GGCTAGTT	XM_357654	hypothetical LOC384452		2.11	SM2368	f	8	1.656455229
mPool7_V2MM_199459	3.328607	2.205692	TRUE	HP_417505	TGCTGTTG	CCATTTAA	XM_289192	Mus musculus LOC332813 (LOC332813), mRNA.		2.9	SM2276	c	3	1.656455229
mPool2_V2MM_17530	3.318772	2.205692	TRUE	HP_240804	TGCTGTTG	GATACTTT	NM_134054	RIKEN cDNA 1110002B05 gene	1110002B05Rik	2.11	SM2378	g	1	1.656455229
092308m1_V2MM_171708	3.314424	1.959154	TRUE	HP_392195	TGCTGTTG	CATGTTAG	XM_286679	Mus musculus hypothetical gene supported by AK039985 (LOC328540), mR.		2.5	SM2074	b	9	1.707931489
mPool2_V2MM_36734	3.311664	3.004939	TRUE	HP_259508	TGCTGTTG	CTTGTACAT	NM_023816	RIKEN cDNA 1700012M14 gene	1700012M14Rik	2.11	SM2392	a	9	1.522164293
mPool2_V2MM_84705	3.309396	3.004939	TRUE	HP_146966	TGCTGTTG	CTAATCATC	NM_028930	transmembrane channel-like gene family 5	Tmc5	2.6	SM2137	b	10	1.522164293
092308m1_V2MM_121144	3.273188	3.004939	TRUE	HP_342027	TGCTGTTG	CGGCATCT	XM_141454	Mus musculus similar to lactate dehydrogese [Mus musculus] (LOC228430), mR.		2.6	SM2106	g	6	1.522164293
092308m1_V2MM_124008	3.268085	2.205692	TRUE	HP_344861	TGCTGTTG	GTCTGGAA	XM_143251	Mus musculus similar to hypothetical protein [Macaca fascicularis] (LOC242043), mR.		2.6	SM2102	b	7	1.656455229
mPool7_V2MM_113184	3.252594	2.205692	TRUE	HP_334174	TGCTGTTG	CTACATTCA	XM_136853	Mus musculus LOC241181 (LOC241181), mRNA.		2.1	SM2304	d	4	1.656455229
mPool2_V2MM_120733	3.242869	2.205692	TRUE	HP_341625	TGCTGTTG	GAAATTTC	XM_141156	Mus musculus similar to olfactory receptor MOR264-19 [Mus musculus] (LOC228269), n		2.11	SM2359	f	5	1.656455229
mPool7_V2MM_79723	3.198836	3.004939	TRUE	HP_301372	TGCTGTTG	GCCAGGAT	NM_009828	cyclin A2	Ccna2	2.8	SM2227	d	7	1.522164293
092308m1_V2MM_159994	3.196066	2.205692	TRUE	HP_380587	TGCTGTTG	CATCTAACC	AK048916	cD sequence BC052055	BC052055	2.6	SM2115	f	1	1.656455229
mPool2_V2MM_14135	3.188079	2.205692	TRUE	HP_237487	TGCTGTTG	CCCTGAAA	NM_007672	cerebellar degeneration-related 2	Cdr2	2.11	SM2379	d	10	1.656455229
mPool2_V2MM_212177	3.187273	1.959154	TRUE	HP_429852	TGCTGTTG	CTTCTATCC	XM_148779	RIKEN cDNA 4930541O19 gene	4930541O19Rik	2.11	SM2365	f	7	1.707931489
mPool2_V2MM_17214	3.164486	2.205692	TRUE	HP_240494	TGCTGTTG	GAGCTAAA	NM_011623	topoisomerase (DNA) II alpha	Top2a	2.11	SM2387	h	6	1.656455229
mPool7_V2MM_67258	3.157407	2.205692	TRUE	HP_289249	TGCTGTTG	CCGTTTGCT	NM_021546	amyloid beta (A4) precursor protein-binding, family A, membe	Apba2bp	2.8	SM2237	d	7	1.656455229
mPool2_V2MM_208999	3.152098	3.004939	TRUE	HP_426776	TGCTGTTG	CTCAGGAA	XM_157672	similar to Suppressor of G2 allele of SKP1 homolog (Sgt1) (Putative 40-6-3 protein)		2.1	SM2338	a	5	1.522164293
mPool2_V2MM_94467	3.148552	2.205692	TRUE	HP_315756	TGCTGTTG	CAGCATGT	BC052532	cDNA sequence BC006779	BC006779	2.6	SM2142	a	7	1.656455229
mPool7_V2MM_202647	3.143102	2.205692	TRUE	HP_420645	TGCTGTTG	GTATGATT	AK045028	histone deacetylase 10	Hdac10	2.1	SM2317	h	10	1.656455229
mPool2_V2MM_1433	3.143072	2.205692	TRUE	HP_225074	TGCTGTTG	CCGCATAC	NM_007855	twist homolog 2 (Drosophila)	Twist2	2.11	SM2386	g	12	1.656455229
mPool2_V2MM_134121	3.135473	3.004939	TRUE	HP_354849	TGCTGTTG	CTGTCTTTA	NM_177582	hypothetical protein B930074I24		2.1	SM2349	h	4	1.522164293
092308m1_V2MM_180277	3.099315	2.205692	TRUE	HP_400751	TGCTGTTG	CTTTAGATC	XM_288480	Mus musculus LOC331828 (LOC331828), mRNA.		2.5	SM2072	h	3	1.656455229
092308m1_V2MM_180277	3.099315	2.205692	TRUE	HP_400751	TGCTGTTG	CTTTAGATC	XM_288480	Mus musculus LOC331828 (LOC331828), mR.		2.5	SM2072	h	3	1.656455229
092308m1_V2MM_156420	3.091247	2.205692	TRUE	HP_377055	TGCTGTTG	CTGCTTTAC	NM_027860	RIKEN cD 0610010F05 gene	0610010F05Rik	2.6	SM2115	e	4	1.656455229
092308m1_V2MM_70303	3.088266	3.004939	TRUE	HP_292219	TGCTGTTG	CAACCAAT	NM_028911	RIKEN cD 4933414I15 gene	4933414I15Rik	2.3	SM2006	e	3	1.522164293
mPool7_V2MM_197834	3.086251	2.205692	TRUE	HP_415896	TGCTGTTG	GATGATAA	XM_111554	similar to Aspartate aminotransferase, mitochondrial precursor (Transamise A) (Glutam		2.9	SM2266	b	12	1.656455229
mPool7_V2MM_197834	3.086251	2.205692	TRUE	HP_415896	TGCTGTTG	GATGATAA	XM_111554	similar to Aspartate aminotransferase, mitochondrial precursor (Transaminase A) (Gluta		2.9	SM2266	b	12	1.656455229
mPool7_V2MM_205300	3.075308	2.205692	TRUE	HP_423167	TGCTGTTG	CAAAGTTC	XM_138746	Mus musculus similar to thymine D glycosylase [Mus musculus] (LOC238743), mR.		2.1	SM2306	d	1	1.656455229
mPool7_V2MM_205300	3.075308	2.205692	TRUE	HP_423167	TGCTGTTG	CAAAGTTC	XM_138746	Mus musculus similar to thymine DNA glycosylase [Mus musculus] (LOC238743), mRNA.		2.1	SM2306	d	1	1.656455229
092308m1_V2MM_135215	3.072112	1.959154	TRUE	HP_355937	TGCTGTTG	CTTTATTTC	XM_489372	similar to RIKEN cD 6720401G13 gene		2.5	SM2057	a	3	1.707931489
mPool2_V2MM_212145	3.039733	3.004939	TRUE	HP_429820	TGCTGTTG	CCCTTTAAA	AK017143	RIKEN cDNA 5031425E22 gene	5031425E22Rik	2.11	SM2362	d	9	1.522164293
mPool7_V2MM_144853	3.031093	2.205692	TRUE	HP_365564	TGCTGTTG	GAGCTTAT	XM_160377	Mus musculus LOC229769 (LOC229769), mRNA.		2.1	SM2331	f	2	1.656455229
092308m1_V2MM_155240	3.030417	2.205692	TRUE	HP_375881	TGCTGTTG	GCCTGAAA	XM_197286	Mus musculus LOC271407 (LOC271407), mR.		2.6	SM2114	g	7	1.656455229
mPool2_V2MM_106531	3.008607	3.004939	TRUE	HP_327631	TGCTGTTG	GAAACATC	NM_001002927	preproenkephalin 1	Penk1	2.1	SM2316	h	4	1.522164293
mPool2_V2MM_210729	3.001055	2.205692	TRUE	HP_428468	TGCTGTTG	CGCAGTTA	XM_359191	gene model 1773, (NCBI)	Gm1773	2.11	SM2363	b	5	1.656455229
mPool2_V2MM_123973	2.985185	2.205692	TRUE	HP_344826	TGCTGTTG	GACAGGAA	XM_143235	Mus musculus similar to embryonic blastocoelar extracellular matrix protein precursor (		2.11	SM2351	d	7	1.656455229
092308m1_V2MM_171296	2.978236	2.205692	TRUE	HP_391785	TGCTGTTG	CCTAGAGT	XM_286593	Mus musculus hypothetical gene supported by AK051402 (LOC328380), mR.		2.5	SM2080	h	5	1.656455229
mPool2_V2MM_142344	2.975649	2.205692	TRUE	HP_363057	TGCTGTTG	CACGAAAC	XM_157589	Mus musculus LOC225224 (LOC225224), mRNA.		2.4	SM2039	c	5	1.656455229
mPool2_V2MM_142344	2.975649	2.205692	TRUE	HP_363057	TGCTGTTG	CACGAAAC	XM_157589	Mus musculus LOC225224 (LOC225224), mR.		2.4	SM2039	c	5	1.656455229
mPool2_V2MM_204673	2.974126	3.004939	TRUE	HP_422565	TGCTGTTG	GATGAATA	XM_129159	RIKEN cDNA 1810073H04 gene	1810073H04Rik	2.1	SM2318	f	8	1.522164293
092308m1_V2MM_172099	2.955371	3.004939	TRUE	HP_392585	TGCTGTTG	CATTCTGA	XM_286756	Mus musculus hypothetical gene supported by AK048704 (LOC328685), mRNA.		2.5	SM2090	h	5	1.522164293
092308m1_V2MM_172099	2.955371	3.004939	TRUE	HP_392585	TGCTGTTG	CATTCTGA	XM_286756	Mus musculus hypothetical gene supported by AK048704 (LOC328685), mR.		2.5	SM2090	h	5	1.522164293
mPool2_V2MM_128454	2.921823	2.205692	TRUE	HP_349268	TGCTGTTG	CAGTAGAA	XM_145689	similar to glyceraldehyde-3-phosphate dehydrogenase		2.11	SM2367	f	8	1.656455229
mPool2_V2MM_136210	2.918504	3.004939	TRUE	HP_356930	TGCTGTTG	CCTACGAA	XM_151496	Mus musculus LOC241166 (LOC241166), mRNA.		2.5	SM2053	h	11	1.522164293
mPool2_V2MM_136210	2.918504	3.004939	TRUE	HP_356930	TGCTGTTG	CCTACGAA	XM_151496	Mus musculus LOC241166 (LOC241166), mR.		2.5	SM2053	h	11	1.522164293
092308m1_V2MM_124037	2.908688	3.004939	TRUE	HP_344890	TGCTGTTG	CAATTGTC	XM_143269	Mus musculus similar to Heat shock protein HSP 90-beta (HSP 84) (LOC229352), mR.		2.6	SM2103	g	10	1.522164293
mPool2_V2MM_126884	2.906645	3.004939	TRUE	HP_347720	TGCTGTTG	CTTGATTA	XM_144862	Mus musculus similar to Gag [Ovis aries] (LOC232180), mRNA.		2.11	SM2360	d	5	1.522164293
mPool2_V2MM_10177	2.90313	3.004939	TRUE	HP_233615	TGCTGTTG	CTACTATTA	NM_007785	casein gamma	Csng	2.11	SM2374	a	10	1.522164293
092308m1_V2MM_186411	2.895897	3.004939	TRUE	HP_406882	TGCTGTTG	CCCTTCAA	XM_289534	Mus musculus LOC333278 (LOC333278), mR.		2.5	SM2084	g	3	1.522164293

092308m1_V2MM_186411	2.895897	3.004939	TRUE	HP_406882	TGCTGTTG/	CCCTTCAAC	XM_289534	Mus musculus LOC333278 (LOC333278), mRNA.		2.5	SM2084	g	3	1.522164293
mPool7_V2MM_180521	2.887155	2.205692	TRUE	HP_400995	TGCTGTTG/	CAGCCAGA	XM_288521	Mus musculus LOC331896 (LOC331896), mRNA.		2.9	SM2277	c	7	1.656455229
mPool2_V2MM_139762	2.886327	3.004939	TRUE	HP_360482	TGCTGTTG/	CCAATTACT	XM_155380	similar to Rpl7a protein		2.5	SM2055	f	2	1.522164293
mPool2_V2MM_139762	2.886327	3.004939	TRUE	HP_360482	TGCTGTTG/	CCAATTACT	XM_155380	similar to Rpl7a protein		2.5	SM2055	f	2	1.522164293
mPool2_V2MM_86036	2.885936	2.205692	TRUE	HP_307514	TGCTGTTG/	CATGTACA	NM_146605	olfactory receptor 828	Olfr828	2.6	SM2145	b	6	1.656455229
mPool2_V2MM_33233	2.885463	2.205692	TRUE	HP_256090	TGCTGTTG/	CACTAAAT(	AK044117	RIKEN cDNA C430014H23 gene	C430014H23Rik	2.11	SM2391	e	11	1.656455229
mPool7_V2MM_117268	2.884369	3.004939	TRUE	HP_338214	TGCTGTTG/	CAGATGAT	XM_139295	caspase recruitment domain family, member 6	Card6	2.1	SM2314	a	1	1.522164293
092308m1_V2MM_124502	2.881563	3.004939	TRUE	HP_345354	TGCTGTTG/	GCCTTCTTC	XM_146378	similar to HMG-1		2.6	SM2103	f	7	1.522164293
092308m1_V2MM_136210	2.874993	3.004939	TRUE	HP_356930	TGCTGTTG/	CCTACGAA	XM_151496	Mus musculus LOC241166 (LOC241166), mR.		2.5	SM2053	h	11	1.522164293
092308m1_V2MM_136210	2.874993	3.004939	TRUE	HP_356930	TGCTGTTG/	CCTACGAA	XM_151496	Mus musculus LOC241166 (LOC241166), mRNA.		2.5	SM2053	h	11	1.522164293
mPool7_V2MM_86351	2.86126	3.004939	TRUE	HP_307827	TGCTGTTG/	CCTTTATAG	NM_146691	olfactory receptor 1467	Olfr1467	2.9	SM2256	e	8	1.522164293
mPool4_V2MM_262088	2.857863	75.83547	TRUE	HP_524979	TGCTGTTG/	CTTGTACA	NM_134206	vomeronasal 1 receptor, G5	V1rg5	2.15	SM2582	f	8	0.120127608
mPool2_V2MM_159248	2.854788	3.004939	TRUE	HP_379849	TGCTGTTG/	GGGTTATC(	XM_283022	zinc finger protein, subfamily 1A, 3 (Aiolos)	Zfpn1a3	2.6	SM2132	c	5	1.522164293
092308m1_V2MM_62859	2.854269	2.205692	TRUE	HP_284955	TGCTGTTG/	CATTCTTCA	NM_010672	keratin associated protein 6-1	Krtap6-1	2.4	SM2020	f	4	1.656455229
mPool7_V2MM_179575	2.838696	2.205692	TRUE	HP_400049	TGCTGTTG/	CTTCAAAT(	XM_288356	Mus musculus LOC331675 (LOC331675), mRNA.		2.9	SM2287	g	12	1.656455229
092308m1_V2MM_142344	2.837502	2.205692	TRUE	HP_363057	TGCTGTTG/	CACGAAAC	XM_157589	Mus musculus LOC225224 (LOC225224), mRNA.		2.4	SM2039	c	5	1.656455229
092308m1_V2MM_142344	2.837502	2.205692	TRUE	HP_363057	TGCTGTTG/	CACGAAAC	XM_157589	Mus musculus LOC225224 (LOC225224), mR.		2.4	SM2039	c	5	1.656455229
mPool7_V2MM_180277	2.837056	2.205692	TRUE	HP_400751	TGCTGTTG/	CTTTAGAT(	XM_288480	Mus musculus LOC331828 (LOC331828), mRNA.		2.5	SM2072	h	3	1.656455229
mPool7_V2MM_180277	2.837056	2.205692	TRUE	HP_400751	TGCTGTTG/	CTTTAGAT(	XM_288480	Mus musculus LOC331828 (LOC331828), mR.		2.5	SM2072	h	3	1.656455229
092308m1_V2MM_149019	2.83581	1.959154	TRUE	HP_369730	TGCTGTTG/	CTGGGAAC	XM_164412	RIKEN cD 1600027J07 gene	1600027J07Rik	2.5	SM2058	h	4	1.707931489
mPool2_V2MM_89163	2.828845	2.205692	TRUE	HP_310593	TGCTGTTG/	CCTCCAACT	NM_178220	arrestin, beta 1	Arrb1	2.6	SM2143	b	9	1.656455229
092308m1_V2MM_187186	2.825406	2.205692	TRUE	HP_407655	TGCTGTTG/	CTCCTACTT	XM_289663	Mus musculus LOC333427 (LOC333427), mR.		2.5	SM2069	f	3	1.656455229
092308m1_V2MM_67710	2.824968	2.205692	TRUE	HP_289691	TGCTGTTG/	CTATTACG/	AB086123	calcium channel, voltage-dependent, L type, alpha 1D subunit	Cac1d	2.3	SM2008	e	10	1.656455229
092308m1_V2MM_83700	2.824072	3.004939	TRUE	HP_305226	TGCTGTTG/	GAGAATGC	NM_023750	zinc finger protein 84	Zfp84	2.6	SM2112	g	4	1.522164293
mPool7_V2MM_198010	2.823741	2.205692	TRUE	HP_416071	TGCTGTTG/	CTATTTCTA	XM_287838	Mus musculus hypothetical gene supported by AK048651 (LOC330802), mRNA.		2.9	SM2277	b	5	1.656455229
092308m1_V2MM_176389	2.82183	2.205692	TRUE	HP_396871	TGCTGTTG/	CCTTAAAT(	AK129335	RIKEN cDNA C230080I20 gene	C230080I20Rik	2.5	SM2077	f	6	1.656455229
092308m1_V2MM_176389	2.82183	2.205692	TRUE	HP_396871	TGCTGTTG/	CCTTAAAT(	AK129335	RIKEN cD C230080I20 gene	C230080I20Rik	2.5	SM2077	f	6	1.656455229
mPool7_V2MM_171714	2.819084	2.205692	TRUE	HP_392201	TGCTGTTG/	CAGTGTTC/	XM_286680	Mus musculus hypothetical gene supported by AK043364 (LOC328593), mRNA.		2.9	SM2294	g	11	1.656455229
mPool2_V2MM_97290	2.816496	2.205692	TRUE	HP_318532	TGCTGTTG/	CACCATTTC	XM_111418	Mus musculus similar to ribosomal protein S9, cytosolic [validated] - rat (LOC218128), m		2.6	SM2144	e	11	1.656455229
092308m1_V2MM_67000	2.815659	2.205692	TRUE	HP_289001	TGCTGTTG/	CCCAGTCA(	NM_028979	cytochrome P450, family 2, subfamily j, polypeptide 9	Cyp2j9	2.4	SM2028	b	6	1.656455229
mPool7_V2MM_177652	2.809365	3.004939	TRUE	HP_398132	TGCTGTTG/	GAATTGGA	AK084093	RIKEN cDNA D130086K05 gene	D130086K05Rik	2.5	SM2060	f	7	1.522164293
mPool7_V2MM_177652	2.809365	3.004939	TRUE	HP_398132	TGCTGTTG/	GAATTGGA	AK084093	RIKEN cD D130086K05 gene	D130086K05Rik	2.5	SM2060	f	7	1.522164293
mPool7_V2MM_102459	2.802223	3.004939	TRUE	HP_323612	TGCTGTTG/	CTGAACTT(	XM_484567	RIKEN cDNA 2310061N02 gene	2310061N02Rik	2.1	SM2302	g	2	1.522164293
092308m1_V2MM_174236	2.780406	3.004939	TRUE	HP_394722	TGCTGTTG/	CTTCCCTTG	AK087574	RIKEN cD E230011G24 gene	E230011G24Rik	2.5	SM2082	h	8	1.522164293
092308m1_V2MM_142084	2.768636	2.205692	TRUE	HP_362797	TGCTGTTG/	GACAGAAC	XM_157307	Mus musculus LOC225477 (LOC225477), mR.		2.4	SM2046	e	8	1.656455229
092308m1_V2MM_142084	2.768636	2.205692	TRUE	HP_362797	TGCTGTTG/	GACAGAAC	XM_157307	Mus musculus LOC225477 (LOC225477), mRNA.		2.4	SM2046	e	8	1.656455229
mPool2_V2MM_18361	2.753327	3.004939	TRUE	HP_241609	TGCTGTTG/	GCCTGTTT(	NM_022880	solute carrier family 29 (nucleoside transporters), member 1	Slc29a1	2.11	SM2377	a	3	1.522164293
092308m1_V2MM_175268	2.751147	3.004939	TRUE	HP_395753	TGCTGTTG/	GTCTTAATT	XM_287405	Mus musculus hypothetical gene supported by AK037004 (LOC330002), mR.		2.5	SM2086	d	10	1.522164293
mPool2_V2MM_120290	2.748502	1.959154	TRUE	HP_341188	TGCTGTTG/	CTTCATTG/	XM_140842	Mus musculus similar to glyceraldehyde-3-phosphate dehydrogenase [Mus musculus] (l		2.11	SM2370	e	4	1.707931489
092308m1_V2MM_172614	2.744887	2.205692	TRUE	HP_393100	TGCTGTTG/	CAGGAAAG	XM_286868	hypothetical protein 9330117O12		2.5	SM2072	f	12	1.656455229
mPool2_V2MM_91867	2.741015	1.959154	TRUE	HP_12628	TGCTGTTG/	CCTACAAA(	NM_178607	ring finger protein 24	Rnf24	2.6	SM2109	h	2	1.707931489
mPool2_V2MM_91867	2.741015	1.959154	TRUE	HP_12628	TGCTGTTG/	CCTACAAA(	NM_178607	ring finger protein 24	Rnf24	2.6	SM2109	h	2	1.707931489
mPool2_V2MM_148147	2.740015	3.004939	TRUE	HP_368858	TGCTGTTG/	GAAGGTTC	XM_163530	Mus musculus LOC244133 (LOC244133), mR.		2.4	SM2040	h	1	1.522164293
mPool2_V2MM_148147	2.740015	3.004939	TRUE	HP_368858	TGCTGTTG/	GAAGGTTC	XM_163530	Mus musculus LOC244133 (LOC244133), mRNA.		2.4	SM2040	h	1	1.522164293
mPool2_V2MM_16143	2.733176	2.205692	TRUE	HP_239447	TGCTGTTG/	CTTCCAGTT	NM_007520	BTB and CNC homology 1	Bach1	2.11	SM2389	e	1	1.656455229
mPool7_V2MM_196342	2.72588	2.205692	TRUE	HP_414449	TGCTGTTG/	GGACTTTA(	NM_177769	hypothetical protein 4831417L10		2.9	SM2254	h	7	1.656455229
092308m1_V2MM_85041	2.725737	3.004939	TRUE	HP_306537	TGCTGTTG/	GTGTTCTCT	NM_030139	zinc finger protein 449	Zfp449	2.6	SM2113	f	4	1.522164293
092308m1_V2MM_138848	2.720962	2.205692	TRUE	HP_359568	TGCTGTTG/	GACATTAA	XM_154566	Mus musculus LOC238925 (LOC238925), mR.		2.4	SM2030	d	11	1.656455229
092308m1_V2MM_138848	2.720962	2.205692	TRUE	HP_359568	TGCTGTTG/	GACATTAA	XM_154566	Mus musculus LOC238925 (LOC238925), mRNA.		2.4	SM2030	d	11	1.656455229
mPool7_V2MM_100846	2.716422	2.205692	TRUE	HP_322023	TGCTGTTG/	GACTGTAT(	XM_127139	echinoderm microtubule associated protein like 1	Eml1	2.1	SM2310	c	10	1.656455229
mPool7_V2MM_80549	2.715909	2.205692	TRUE	HP_302178	TGCTGTTG/	GGAAATGT	NM_011192	proteaseome (prosome, macropain) 28 subunit, 3	Psme3	2.8	SM2227	b	6	1.656455229
092308m1_V2MM_83476	2.7159	3.004939	TRUE	HP_305016	TGCTGTTG/	CACTTTATT	NM_017368	CUG triplet repeat, R binding protein 1	Cugbp1	2.6	SM2112	e	2	1.522164293
092308m1_V2MM_159791	2.714818	2.205692	TRUE	HP_380384	TGCTGTTG/	GTTTCTATT	AK081652	pam, highwire, rpm 1	Phr1	2.6	SM2116	d	3	1.656455229
092308m1_V2MM_73747	2.710122	2.205692	TRUE	HP_295571	TGCTGTTG/	GTCAGGTT	NM_146808	olfactory receptor 1240	Olfr1240	2.3	SM2015	b	2	1.656455229
092308m1_V2MM_170566	2.708142	1.959154	TRUE	HP_391059	TGCTGTTG/	GCGTTTGT	XM_286444	Mus musculus hypothetical gene supported by AK028654 (LOC328131), mR.		2.5	SM2083	b	7	1.707931489
092308m1_V2MM_177051	2.695729	3.004939	TRUE	HP_397531	TGCTGTTG/	GAAGTATT	AK047086	hypothetical protein B930018H19		2.5	SM2092	f	7	1.522164293
092308m1_V2MM_132337	2.694129	3.004939	TRUE	HP_353091	TGCTGTTG/	CTCCTTCTG	XM_148441	RIKEN cD 5730478M09 gene	5730478M09Rik	2.6	SM2103	g	11	1.522164293
mPool2_V2MM_100946	2.69225	2.205692	TRUE	HP_322122	TGCTGTTG/	CATTCTTAA	XM_127279	RIKEN cDNA 1110037N09 gene	1110037N09Rik	2.6	SM2149	b	8	1.656455229
mPool7_V2MM_74811	2.690379	2.205692	TRUE	HP_296607	TGCTGTTG/	CAGGTTTG/	BC005723	RIKEN cDNA 1700029K01 gene	1700029K01Rik	2.8	SM2246	e	8	1.656455229
092308m1_V2MM_178563	2.684133	1.959154	TRUE	HP_399037	TGCTGTTG/	CAATTTAA	XM_288183	Mus musculus LOC329171 (LOC329171), mR.		2.5	SM2082	d	4	1.707931489
mPool2_V2MM_153839	2.680466	3.004939	TRUE	HP_374482	TGCTGTTG/	GCTTGGGA	BC030424	A930003O13		2.6	SM2132	h	3	1.522164293
mPool2_V2MM_213746	2.672153	3.004939	TRUE	HP_431368	TGCTGTTG/	CTGGTTAA	XM_149205	ring finger protein 24	Rnf24	2.11	SM2371	a	9	1.522164293

092308m1_V2MM_87899	2.671989	2.205692	TRUE	HP_309346	TGCTGTTG/	GGCATTGC/	NM_175270	RIKEN cD 5730467H21 gene	5730467H21Rik	2.6	SM2106	c	10	1.656455229
mPool2_V2MM_145984	2.667191	2.205692	TRUE	HP_366695	TGCTGTTG/	CCATGTCTC	XM_161687	Mus musculus LOC242888 (LOC242888), mRNA.		2.1	SM2345	h	4	1.656455229
mPool7_V2MM_96447	2.66463	2.205692	TRUE	HP_317700	TGCTGTTG/	GATTTCTGC	XM_110690	RIKEN cDNA 1810033B17 gene	1810033B17Rik	2.9	SM2253	d	11	1.656455229
mPool7_V2MM_96447	2.66463	2.205692	TRUE	HP_317700	TGCTGTTG/	GATTTCTGC	XM_110690	RIKEN cD 1810033B17 gene	1810033B17Rik	2.9	SM2253	d	11	1.656455229
mPool2_V2MM_119501	2.661508	1.959154	TRUE	HP_340419	TGCTGTTG/	GTATTTCAC	XM_140461	RIKEN cDNA 2410072D24 gene	2410072D24Rik	2.11	SM2361	h	1	1.707931489
mPool2_V2MM_136543	2.661019	2.205692	TRUE	HP_357263	TGCTGTTG/	GTTATCTTG	XM_151819	Mus musculus LOC213261 (LOC213261), mRNA.		2.4	SM2034	c	9	1.656455229
mPool2_V2MM_136543	2.661019	2.205692	TRUE	HP_357263	TGCTGTTG/	GTTATCTTG	XM_151819	Mus musculus LOC213261 (LOC213261), mR.		2.4	SM2034	c	9	1.656455229
mPool7_V2MM_110292	2.660996	0	TRUE	NA	NA	NA	NA	NA	NA	NA	NA	NA	NA	#NUM!
mPool2_V2MM_120180	2.659371	3.004939	TRUE	HP_341082	TGCTGTTG/	CTCTCCTAA	XM_140790	similar to Rpl7a protein		2.6	SM2110	a	12	1.522164293
mPool2_V2MM_120180	2.659371	3.004939	TRUE	HP_341082	TGCTGTTG/	CTCTCCTAA	XM_140790	similar to Rpl7a protein		2.6	SM2110	a	12	1.522164293
092308m1_V2MM_142189	2.654747	2.205692	TRUE	HP_362902	TGCTGTTG/	GCTAATTTC	XM_157391	Mus musculus LOC240317 (LOC240317), mR.		2.4	SM2032	g	8	1.656455229
092308m1_V2MM_142189	2.654747	2.205692	TRUE	HP_362902	TGCTGTTG/	GCTAATTTC	XM_157391	Mus musculus LOC240317 (LOC240317), mRNA.		2.4	SM2032	g	8	1.656455229
092308m1_V2MM_141892	2.654311	2.205692	TRUE	HP_362605	TGCTGTTG/	CTTTATCCA	XM_157111	Mus musculus LOC212310 (LOC212310), mR.		2.4	SM2046	b	11	1.656455229
mPool2_V2MM_113323	2.650507	3.004939	TRUE	HP_334310	TGCTGTTG/	CCCAGAAC/	XM_136918	Mus musculus similar to glyceraldehyde-3-phosphate dehydrogenase [Mus musculus] (L		2.7	SM2154	e	9	1.522164293
mPool7_V2MM_196482	2.648947	3.004939	TRUE	HP_414586	TGCTGTTG/	CTGTCTTTA	XM_125673	CXXC finger 6	Cxxc6	2.9	SM2266	g	2	1.522164293
mPool7_V2MM_113323	2.64584	3.004939	TRUE	HP_334310	TGCTGTTG/	CCCAGAAC/	XM_136918	Mus musculus similar to glyceraldehyde-3-phosphate dehydrogenase [Mus musculus] (L		2.7	SM2154	e	9	1.522164293
092308m1_V2MM_176355	2.645547	2.205692	TRUE	HP_396837	TGCTGTTG/	CTCTGTTTG	XM_489154	RIKEN cD 9330102E08 gene	9330102E08Rik	2.5	SM2065	b	4	1.656455229
mPool2_V2MM_144423	2.642873	3.004939	TRUE	HP_365134	TGCTGTTG/	GAATTGGA.	XM_159993	Mus musculus LOC241988 (LOC241988), mRNA.		2.1	SM2344	h	1	1.522164293
mPool2_V2MM_124466	2.637419	3.004939	TRUE	HP_345318	TGCTGTTG/	CGGCAATT(	XM_143471	potassium voltage-gated channel, shaker-related subfamily, m	Kcna10	2.11	SM2357	c	5	1.522164293
mPool2_V2MM_13552	2.621147	3.004939	TRUE	HP_236917	TGCTGTTG/	CATTTGTAC	NM_026405	RAB32, member RAS oncogene family	Rab32	2.11	SM2380	b	3	1.522164293
mPool2_V2MM_32012	2.620331	3.004939	TRUE	HP_254901	TGCTGTTG/	GATGGAAG	NM_023500	McLeod syndrome gene homolog	Xkh	2.11	SM2389	a	12	1.522164293
mPool2_V2MM_124810	2.612072	3.004939	TRUE	HP_345659	TGCTGTTG/	CTTTCAAAC	XM_143641	Mus musculus similar to TRAV19 [Mus musculus] (LOC230001), mRNA.		2.11	SM2352	d	7	1.522164293
092308m1_V2MM_91867	2.607876	2.205692	TRUE	HP_12628	TGCTGTTG/	CCTACAAA(	NM_178607	ring finger protein 24	Rnf24	2.6	SM2109	h	2	1.656455229
092308m1_V2MM_91867	2.607876	2.205692	TRUE	HP_12628	TGCTGTTG/	CCTACAAA(	NM_178607	ring finger protein 24	Rnf24	2.6	SM2109	h	2	1.656455229
092308m1_V2MM_98561	2.596861	2.205692	TRUE	HP_319787	TGCTGTTG/	CGTATTTGC	XM_112650	aquaporin 9	Aqp9	2.6	SM2112	h	8	1.656455229
mPool7_V2MM_172099	2.592874	2.205692	TRUE	HP_392585	TGCTGTTG/	CATTCTGAC	XM_286756	Mus musculus hypothetical gene supported by AK048704 (LOC328685), mRNA.		2.5	SM2090	h	5	1.656455229
mPool7_V2MM_172099	2.592874	2.205692	TRUE	HP_392585	TGCTGTTG/	CATTCTGAC	XM_286756	Mus musculus hypothetical gene supported by AK048704 (LOC328685), mR.		2.5	SM2090	h	5	1.656455229
092308m1_V2MM_150902	2.591938	2.205692	TRUE	HP_371584	TGCTGTTG/	CTGAGTTA/	BC059023	expressed sequence AW060220	AW060220	2.4	SM2037	b	11	1.656455229
092308m1_V2MM_152512	2.591886	2.205692	TRUE	HP_373175	TGCTGTTG/	GCCACTTC/	XM_195409	Mus musculus similar to protease [Trichosurus vulpecula] (LOC270243), mR.		2.4	SM2030	c	4	1.656455229
092308m1_V2MM_78324	2.59181	2.205692	TRUE	HP_300016	TGCTGTTG/	GACCCAAT(	NM_021557	retinol dehydrogese 11	Rdh11	2.3	SM2007	g	7	1.656455229
mPool2_V2MM_208908	2.588866	3.004939	TRUE	HP_426688	TGCTGTTG/	CATGTTAGT	XM_163904	Mus musculus LOC244280 (LOC244280), mRNA.		2.1	SM2336	f	5	1.522164293
mPool7_V2MM_184400	2.583425	1.959154	TRUE	HP_404872	TGCTGTTG/	CCCATTTAA	XM_289192	Mus musculus LOC332813 (LOC332813), mRNA.		2.9	SM2295	c	5	1.707931489
092308m1_V2MM_154074	2.581221	3.004939	TRUE	HP_374715	TGCTGTTG/	CCTGTTTAC	NM_175535	Rho GTPase activating protein 20	Arhgap20	2.6	SM2114	f	6	1.522164293
mPool7_V2MM_107136	2.566738	2.205692	TRUE	HP_328226	TGCTGTTG/	CTGTTTACT	BC060239	RIKEN cDNA 2610109H07 gene	2610109H07Rik	2.1	SM2313	c	8	1.656455229
092308m3_V2MM_98132	2.560958	75.83547	TRUE	HP_319365	TGCTGTTG/	CCCTTTGG/	XM_194868	Mus musculus similar to hypothetical protein [Plasmodium yoelii yoelii] (LOC268610), m		2.13	SM2472	g	11	0.120127608
092308m1_V2MM_173028	2.560137	3.004939	TRUE	HP_393514	TGCTGTTG/	GATCCAAA(	XM_286944	Mus musculus hypothetical gene supported by AK089863 (LOC329049), mR.		2.5	SM2067	b	6	1.522164293
092308m1_V2MM_63008	2.556561	2.205692	TRUE	HP_285098	TGCTGTTG/	CCGATATTC	NM_013818	GTP binding protein 1	Gtpbp1	2.4	SM2025	c	6	1.656455229
mPool7_V2MM_94358	2.544241	3.004939	TRUE	HP_315650	TGCTGTTG/	GTGGTAAA	NM_183137	RIKEN cDNA 2410002I01 gene	2410002I01Rik	2.8	SM2249	g	8	1.522164293
mPool2_V2MM_21743	2.543583	3.004939	TRUE	HP_244911	TGCTGTTG/	CATTTCTTA	NM_145586	RIKEN cDNA 8430420C20 gene	8430420C20Rik	2.11	SM2389	f	9	1.522164293
mPool2_V2MM_87747	2.543217	3.004939	TRUE	HP_309195	TGCTGTTG/	CCAACTCAT	NM_175236	alcohol dehydrogese, iron containing, 1	Adhfe1	2.6	SM2108	e	12	1.522164293
mPool2_V2MM_87747	2.543217	3.004939	TRUE	HP_309195	TGCTGTTG/	CCAACTCAT	NM_175236	alcohol dehydrogenase, iron containing, 1	Adhfe1	2.6	SM2108	e	12	1.522164293
mPool2_V2MM_5090	2.537657	1.959154	TRUE	HP_228649	TGCTGTTG/	CATCTACAC	NM_010114	kallikrein 22	Klk22	2.11	SM2384	d	6	1.707931489
mPool7_V2MM_171247	2.534961	2.205692	TRUE	HP_391736	TGCTGTTG/	CCTTTATAT	XM_286584	hypothetical LOC328369		2.5	SM2080	h	2	1.656455229
mPool7_V2MM_171247	2.534961	2.205692	TRUE	HP_391736	TGCTGTTG/	CCTTTATAT	XM_286584	hypothetical LOC328369		2.5	SM2080	h	2	1.656455229
mPool2_V2MM_8473	2.534002	2.205692	TRUE	HP_231957	TGCTGTTG/	GTCTTTATA	NM_008148	glycoprotein 5 (platelet)	Gp5	2.11	SM2377	d	7	1.656455229
092308m1_V2MM_180103	2.533756	3.004939	TRUE	HP_400577	TGCTGTTG/	GTTGATTTA	XM_288450	Mus musculus LOC331788 (LOC331788), mR.		2.5	SM2082	h	7	1.522164293
092308m1_V2MM_145554	2.532402	2.205692	TRUE	HP_366265	TGCTGTTG/	CATTCTTAT	XM_161053	Mus musculus LOC230170 (LOC230170), mRNA.		2.4	SM2040	e	9	1.656455229
092308m1_V2MM_145554	2.532402	2.205692	TRUE	HP_366265	TGCTGTTG/	CATTCTTAT	XM_161053	Mus musculus LOC230170 (LOC230170), mR.		2.4	SM2040	e	9	1.656455229
mPool7_V2MM_176389	2.532005	3.004939	TRUE	HP_396871	TGCTGTTG/	CCTTAAATC	AK129335	RIKEN cD C230080I20 gene	C230080I20Rik	2.5	SM2077	f	6	1.522164293
mPool7_V2MM_176389	2.532005	3.004939	TRUE	HP_396871	TGCTGTTG/	CCTTAAATC	AK129335	RIKEN cDNA C230080I20 gene	C230080I20Rik	2.5	SM2077	f	6	1.522164293
mPool7_V2MM_139283	2.530036	3.004939	TRUE	HP_360003	TGCTGTTG/	CATTCTTTA	XM_154903	Mus musculus LOC210773 (LOC210773), mRNA.		2.1	SM2327	f	8	1.522164293
mPool2_V2MM_134455	2.529677	2.205692	TRUE	HP_355181	TGCTGTTG/	GTACATCT/	XM_149889	Mus musculus LOC232903 (LOC232903), mRNA.		2.11	SM2368	h	6	1.656455229
mPool7_V2MM_114037	2.528273	2.205692	TRUE	HP_335024	TGCTGTTG/	CCATTTGAC	XM_137252	Mus musculus similar to nuclear transport factor 2 [Rattus norvegicus] (LOC237546), mF		2.7	SM2162	b	5	1.656455229
mPool2_V2MM_12274	2.527311	3.004939	TRUE	HP_235675	TGCTGTTG/	GTGGATGA	NM_007658	cell division cycle 25 homolog A (S. cerevisiae)	Cdc25a	2.11	SM2378	c	10	1.522164293
mPool2_V2MM_8682	2.521539	3.004939	TRUE	HP_232163	TGCTGTTG/	GTTAGCTTC	NM_008258	hematological and neurological expressed sequence 1	Hn1	2.11	SM2383	a	10	1.522164293
mPool2_V2MM_91937	2.520823	2.205692	TRUE	HP_313301	TGCTGTTG/	GTACTTGTC	NM_178619	RIKEN cDNA 1810026J23 gene	1810026J23Rik	2.6	SM2138	f	1	1.656455229
mPool2_V2MM_146410	2.520178	2.205692	TRUE	HP_367121	TGCTGTTG/	CCTAAGTTC	XM_161991	Mus musculus LOC243104 (LOC243104), mRNA.		2.1	SM2347	d	7	1.656455229
mPool7_V2MM_89146	2.517894	3.004939	TRUE	HP_310576	TGCTGTTG/	GCCCTAAC/	NM_176942	gamma-aminobutyric acid (GABA-A) receptor, subunit alpha 5	Gabra5	2.9	SM2262	b	5	1.522164293
092308m1_V2MM_181334	2.512468	1.959154	TRUE	HP_401808	TGCTGTTG/	CCTATTACC	XM_288658	Mus musculus LOC328501 (LOC328501), mR.		2.5	SM2085	d	11	1.707931489
092308m1_V2MM_181334	2.512468	1.959154	TRUE	HP_401808	TGCTGTTG/	CCTATTACC	XM_288658	Mus musculus LOC328501 (LOC328501), mRNA.		2.5	SM2085	d	11	1.707931489
mPool2_V2MM_90692	2.508323	3.004939	TRUE	HP_312108	TGCTGTTG/	GGCACTAA(	AK033743	lipocalin 6	Lcn6	2.6	SM2139	h	6	1.522164293
mPool2_V2MM_114037	2.507135	2.205692	TRUE	HP_335024	TGCTGTTG/	CCATTTGAC	XM_137252	Mus musculus similar to nuclear transport factor 2 [Rattus norvegicus] (LOC237546), mF		2.7	SM2162	b	5	1.656455229

mPool2_V2MM_127305	2.5064	2.205692	TRUE	HP_348136	TGCTGTTG	GTGAAGTA	AK032959	RIKEN cDNA A430107O13 gene	A430107O13Rik		2.11	SM2351	f		3	1.656455229
092308m1_V2MM_131144	2.505932	2.205692	TRUE	HP_351910	TGCTGTTG	CAGAAATC	XM_147444	RIKEN cD 4930485B16 gene	4930485B16Rik		2.5	SM2100	a		7	1.656455229
092308m1_V2MM_93146	2.505727	1.959154	TRUE	HP_314478	TGCTGTTG	GTGGATAA	AK083705	expressed sequence AI256775	AI256775		2.6	SM2110	a		2	1.707931489
092308m1_V2MM_93146	2.505727	1.959154	TRUE	HP_314478	TGCTGTTG	GTGGATAA	AK083705	expressed sequence AI256775	AI256775		2.6	SM2110	a		2	1.707931489
092308m1_V2MM_154975	2.502832	2.205692	TRUE	HP_375616	TGCTGTTG	GTGAACTC	XM_197114	hypothetical LOC380894			2.6	SM2122	e		1	1.656455229
mPool7_V2MM_63851	2.498773	2.205692	TRUE	HP_285918	TGCTGTTG	CTCATTATC	NM_146868	olfactory receptor 894	Olfr894		2.8	SM2239	f		10	1.656455229
092308m1_V2MM_184354	2.48845	2.205692	TRUE	HP_404826	TGCTGTTG	CAGCATTT	XM_289184	Mus musculus LOC332804 (LOC332804), mRNA.			2.5	SM2069	f		4	1.656455229
092308m1_V2MM_184354	2.48845	2.205692	TRUE	HP_404826	TGCTGTTG	CAGCATTT	XM_289184	Mus musculus LOC332804 (LOC332804), mR.			2.5	SM2069	f		4	1.656455229
mPool7_V2MM_171879	2.483358	1.959154	TRUE	HP_392366	TGCTGTTG	CATTGCTCC	AK078693	RIKEN cDNA 7530414M10 gene	7530414M10Rik		2.9	SM2272	a		11	1.707931489
mPool2_V2MM_82528	2.478454	3.004939	TRUE	HP_304100	TGCTGTTG	GAGCTATT	NM_009700	aquaporin 4	Aqp4		2.7	SM2164	g		12	1.522164293
mPool2_V2MM_82528	2.478454	3.004939	TRUE	HP_304100	TGCTGTTG	GAGCTATT	NM_009700	aquaporin 4	Aqp4		2.7	SM2164	g		12	1.522164293
mPool2_V2MM_5145	2.478236	3.004939	TRUE	HP_228704	TGCTGTTG	CATTGTAA	NM_027398	Kv channel-interacting protein 1	Kcnip1		2.11	SM2389	c		1	1.522164293
mPool2_V2MM_120050	2.477423	3.004939	TRUE	HP_340958	TGCTGTTG	CTAAACAT	XM_140736	Mus musculus similar to pol protein [Homo sapiens] (LOC226212), mRNA.			2.11	SM2363	h		7	1.522164293
092308m1_V2MM_179548	2.465744	3.004939	TRUE	HP_400022	TGCTGTTG	CCTACAAT	XM_288352	Mus musculus LOC331672 (LOC331672), mRNA.			2.5	SM2091	d		5	1.522164293
092308m1_V2MM_179548	2.465744	3.004939	TRUE	HP_400022	TGCTGTTG	CCTACAAT	XM_288352	Mus musculus LOC331672 (LOC331672), mR.			2.5	SM2091	d		5	1.522164293
mPool7_V2MM_205982	2.462338	0	TRUE	HP_423829	TGCTGTTG	GTTGAAAC	BC054817	RIKEN cDNA 1810011E08 gene	1810011E08Rik		2.9	SM2299	b		1	#NUM!
mPool7_V2MM_84945	2.461374	2.205692	TRUE	HP_306444	TGCTGTTG	CTAATTCCA	NM_029891	RIKEN cDNA 9430034D17 gene	9430034D17Rik		2.9	SM2254	d		4	1.656455229
092308m1_V2MM_128357	2.455774	3.004939	TRUE	HP_349171	TGCTGTTG	CCCATTACC	XM_145657	Mus musculus similar to membrane and microfilament-associated protein p58 [Rattus n			2.6	SM2106	c		2	1.522164293
092308m1_V2MM_178888	2.451542	3.004939	TRUE	HP_399362	TGCTGTTG	CTGATTATC	XM_288237	Mus musculus LOC332503 (LOC332503), mR.			2.5	SM2086	e		9	1.522164293
092308m1_V2MM_167815	2.446867	2.205692	TRUE	HP_388327	TGCTGTTG	CCACTTCA	XM_285849	Mus musculus similar to protease [Trichosurus vulpecula] (LOC329987), mR.			2.6	SM2123	d		8	1.656455229
092308m1_V2MM_177650	2.445472	2.205692	TRUE	HP_398130	TGCTGTTG	GTCCTTGA	AK084093	RIKEN cD D130086K05 gene	D130086K05Rik		2.5	SM2095	a		3	1.656455229
092308m1_V2MM_179034	2.443166	3.004939	TRUE	HP_399508	TGCTGTTG	CAGGATAT	XM_288262	Mus musculus LOC329282 (LOC329282), mRNA.			2.5	SM2089	b		6	1.522164293
092308m1_V2MM_179034	2.443166	3.004939	TRUE	HP_399508	TGCTGTTG	CAGGATAT	XM_288262	Mus musculus LOC329282 (LOC329282), mR.			2.5	SM2089	b		6	1.522164293
092308m1_V2MM_64431	2.439037	3.004939	TRUE	HP_286486	TGCTGTTG	CTGGGAGT	XM_195585	Mus musculus similar to MAP/microtubule affinity-regulating kise like 1; MARK4 serine/			2.4	SM2020	c		12	1.522164293
mPool7_V2MM_184599	2.437695	2.205692	TRUE	HP_405071	TGCTGTTG	CACTTTCTA	XM_289225	Mus musculus LOC332850 (LOC332850), mRNA.			2.9	SM2290	e		12	1.656455229
mPool7_V2MM_184599	2.437695	2.205692	TRUE	HP_405071	TGCTGTTG	CACTTTCTA	XM_289225	Mus musculus LOC332850 (LOC332850), mR.			2.9	SM2290	e		12	1.656455229
092308m1_V2MM_80443	2.43119	3.004939	TRUE	HP_302076	TGCTGTTG	GCTTAAAG	NM_009850	CD3 antigen, gamma polypeptide	Cd3g		2.4	SM2024	f		9	1.522164293
mPool2_V2MM_30861	2.430164	2.205692	TRUE	HP_253774	TGCTGTTG	CTCAGAAT	NM_015825	SH3-binding domain glutamic acid-rich protein	Sh3bgr		2.11	SM2390	f		9	1.656455229
mPool2_V2MM_123245	2.427093	2.205692	TRUE	HP_344101	TGCTGTTG	CTTATATTG	XM_142826	Mus musculus similar to major allergen 1-like; lacrimal gland protein [Mus musculus] (L			2.11	SM2353	f		2	1.656455229
mPool7_V2MM_101648	2.426232	3.004939	TRUE	HP_322815	TGCTGTTG	CCTTTGAT	XM_127760	Mus musculus neurofilament, light polypeptide (Nefl), mRNA.			2.1	SM2301	d		3	1.522164293
092308m1_V2MM_167262	2.425224	3.004939	TRUE	HP_387776	TGCTGTTG	CACATTCCT	XM_485247	similar to arylacetamide deacetylase			2.6	SM2124	d		6	1.522164293
mPool7_V2MM_66116	2.422509	3.004939	TRUE	HP_288138	TGCTGTTG	GTGTGGAA	NM_011210	protein tyrosine phosphatase, receptor type, C	Ptprc		2.8	SM2242	c		4	1.522164293
092308m1_V2MM_153010	2.409313	3.004939	TRUE	HP_373672	TGCTGTTG	CTGCTTAA	XM_195653	Mus musculus similar to Amphoterin (LOC272391), mR.			2.6	SM2117	c		4	1.522164293
mPool2_V2MM_3920	2.4076	2.205692	TRUE	HP_227503	TGCTGTTG	GGACTTGT	NM_010836	homeo box, msh-like 3	Msx3		2.11	SM2377	h		12	1.656455229
092308m1_V2MM_136213	2.405075	2.205692	TRUE	HP_356933	TGCTGTTG	CTACGAAA	XM_151496	Mus musculus LOC241166 (LOC241166), mR.			2.5	SM2052	b		2	1.656455229
mPool4_V2MM_105816	2.403439	75.83547	TRUE	HP_326925	TGCTGTTG	CCATTGGA	XM_130816	similar to Retrovirus-related POL polyprotein			2.16	SM2625	e		4	0.120127608
mPool2_V2MM_84527	2.397941	3.004939	TRUE	HP_306038	TGCTGTTG	CATAGTAA	NM_028259	ribosomal protein S6 kinase, polypeptide 1	Rps6kb1		2.6	SM2146	g		12	1.522164293
092308m1_V2MM_171406	2.396564	2.205692	TRUE	HP_391895	TGCTGTTG	CTCGTTGA	XM_286618	hypothetical protein E030037F02			2.5	SM2073	b		12	1.656455229
092308m1_V2MM_66137	2.393384	3.004939	TRUE	HP_288159	TGCTGTTG	GTTAATAC	NM_025741	RIKEN cD 4931412G03 gene	4931412G03Rik		2.4	SM2027	e		10	1.522164293
mPool7_V2MM_174664	2.390888	3.004939	TRUE	HP_395149	TGCTGTTG	CTGTATTCT	AK028474	hypothetical protein 4631419I20			2.9	SM2276	f		9	1.522164293
mPool7_V2MM_201943	2.387435	2.205692	TRUE	HP_419969	TGCTGTTG	GGGTTTAG	AK052405	RIKEN cDNA C130089K02 gene	C130089K02Rik		2.9	SM2291	d		8	1.656455229
092308m1_V2MM_171247	2.385208	2.205692	TRUE	HP_391736	TGCTGTTG	CCTTTATAT	XM_286584	hypothetical LOC328369			2.5	SM2080	h		2	1.656455229
092308m1_V2MM_171247	2.385208	2.205692	TRUE	HP_391736	TGCTGTTG	CCTTTATAT	XM_286584	hypothetical LOC328369			2.5	SM2080	h		2	1.656455229
092308m1_V2MM_185463	2.385014	3.004939	TRUE	HP_405935	TGCTGTTG	GTCTAAGT	XM_289375	Mus musculus LOC333041 (LOC333041), mR.			2.5	SM2062	c		12	1.522164293
092308m1_V2MM_185463	2.385014	3.004939	TRUE	HP_405935	TGCTGTTG	GTCTAAGT	XM_289375	Mus musculus LOC333041 (LOC333041), mRNA.			2.5	SM2062	c		12	1.522164293
mPool7_V2MM_90186	2.381069	1.959154	TRUE	HP_311609	TGCTGTTG	CATCCAGA	NM_177750	hypothetical protein B930041G04			2.9	SM2251	e		3	1.707931489
mPool2_V2MM_129470	2.377483	3.004939	TRUE	HP_350276	TGCTGTTG	CTGAGTAT	XM_486101	similar to hypothetical protein FLJ25801			2.1	SM2350	g		6	1.522164293
mPool2_V2MM_210414	2.37623	3.004939	TRUE	HP_428167	TGCTGTTG	CTAAATAC	XM_143858	similar to Elongation factor 1-alpha 1 (EF-1-alpha-1) (Elongation factor 1 A-1) (eEF1A-1)			2.11	SM2367	h		11	1.522164293
092308m1_V2MM_165904	2.373889	2.205692	TRUE	HP_386438	TGCTGTTG	CGCTATGT	XM_285354	olfactory receptor 1065	Olfr1065		2.6	SM2123	c		6	1.656455229
092308m1_V2MM_62055	2.373677	3.004939	TRUE	HP_284171	TGCTGTTG	CACCGTCTT	NM_011059	peptidyl arginine deimise, type I	Padi1		2.3	SM2018	h		4	1.522164293
mPool2_V2MM_1570	2.373061	3.004939	TRUE	HP_225209	TGCTGTTG	CCTTAAATC	NM_025818	RIKEN cDNA 1200014J11 gene	1200014J11Rik		2.11	SM2384	f		9	1.522164293
092308m1_V2MM_131266	2.372112	3.004939	TRUE	HP_352030	TGCTGTTG	CAGCTAAG	NM_213729	expressed sequence AI842396	AI842396		2.5	SM2097	g		3	1.522164293
mPool2_V2MM_6688	2.371223	2.205692	TRUE	HP_230216	TGCTGTTG	CTTGATTTC	NM_054094	butyryl Coenzyme A synthetase 1	Bucs1		2.11	SM2389	g		2	1.656455229
mPool7_V2MM_199554	2.366627	3.004939	TRUE	HP_417598	TGCTGTTG	CTCATTATC	XM_287452	Mus musculus hypothetical gene supported by AK035988 (LOC330083), mRNA.			2.9	SM2272	b		9	1.522164293
mPool2_V2MM_157089	2.366555	3.004939	TRUE	HP_377710	TGCTGTTG	CTTGGCAT	XM_204855	Mus musculus similar to hypothetical protein D930020E02 (LOC277170), mRNA.			2.6	SM2127	h		10	1.522164293
mPool7_V2MM_86833	2.364566	1.959154	TRUE	HP_308303	TGCTGTTG	GTTATTAA	NM_173783	RIKEN cDNA B430216B18 gene	B430216B18Rik		2.9	SM2265	f		2	1.707931489
mPool2_V2MM_212162	2.361153	2.205692	TRUE	HP_429837	TGCTGTTG	CCAGATTT	XM_141639	Mus musculus similar to protease [Mus musculus] (LOC236704), mRNA.			2.11	SM2362	c		2	1.656455229
mPool7_V2MM_113519	2.360552	3.004939	TRUE	HP_334506	TGCTGTTG	CTTGCTCTC	XM_137005	Mus musculus similar to Hypothetical protein KIAA0144 (LOC212406), mRNA.			2.7	SM2154	f		2	1.522164293
092308m1_V2MM_176364	2.353708	2.205692	TRUE	HP_396846	TGCTGTTG	CATTCATTC	XM_287651	Mus musculus hypothetical gene supported by AK079645 (LOC330422), mR.			2.5	SM2079	b		1	1.656455229
092308m1_V2MM_176364	2.353708	2.205692	TRUE	HP_396846	TGCTGTTG	CATTCATTC	XM_287651	Mus musculus hypothetical gene supported by AK079645 (LOC330422), mRNA.			2.5	SM2079	b		1	1.656455229
mPool2_V2MM_162783	2.353143	1.959154	TRUE	HP_383341	TGCTGTTG	CTTTGGAA	XM_284493	Mus musculus hypothetical gene supported by AK083277 (LOC330932), mRNA.			2.6	SM2130	e		8	1.707931489
mPool5_V2MM_14453	2.352201	70.87591	TRUE	HP_237799	TGCTGTTG	CCTCCCAG	NM_025875	RNA binding motif protein 8a	Rbm8a	NA				NA		0.149501346

mPool2_V2MM_1149	2.350584	2.205692	TRUE	HP_224799	TGCTGTTG/	CATCAACT(	NM_011721	Werner syndrome homolog (human)	Wrn	2.11	SM2385	a	3	1.656455229
mPool7_V2MM_197705	2.344005	3.004939	TRUE	HP_350743	TGCTGTTG/	GCGAGTAT/	XM_356738	similar to T-cell receptor alpha variable region family 13 subfamily 1		2.9	SM2263	h	6	1.522164293
mPool2_V2MM_121994	2.343889	2.205692	TRUE	HP_342868	TGCTGTTG/	CAGTTATA(	NM_001003916	cDNA sequence AK129302	AK129302	2.1	SM2350	g	2	1.656455229
mPool2_V2MM_121891	2.34312	3.004939	TRUE	HP_342765	TGCTGTTG/	CTGAGTCT(	NM_141921	gene model 370, (NCBI)	Gm370	2.1	SM2350	f	6	1.522164293
mPool2_V2MM_209788	2.342931	2.205692	TRUE	HP_427553	TGCTGTTG/	CCAATTTCC	XM_163354	Mus musculus LOC215451 (LOC215451), mRNA.		2.1	SM2336	g	12	1.656455229
mPool2_V2MM_142189	2.340758	1.959154	TRUE	HP_362902	TGCTGTTG/	GCTAATTT(	XM_157391	Mus musculus LOC240317 (LOC240317), mRNA.		2.4	SM2032	g	8	1.707931489
mPool2_V2MM_142189	2.340758	1.959154	TRUE	HP_362902	TGCTGTTG/	GCTAATTT(	XM_157391	Mus musculus LOC240317 (LOC240317), mR.		2.4	SM2032	g	8	1.707931489
mPool2_V2MM_5233	2.336743	2.205692	TRUE	HP_228790	TGCTGTTG/	CCACTGTT(	NM_025806	RIKEN cDNA 1100001H23 gene	1100001H23Rik	2.11	SM2383	d	4	1.656455229
mPool2_V2MM_141349	2.327546	3.004939	TRUE	HP_362062	TGCTGTTG/	GAGAAATT	XM_156599	Mus musculus LOC239923 (LOC239923), mRNA.		2.1	SM2345	h	9	1.522164293
092308m1_V2MM_169742	2.326999	3.004939	TRUE	HP_390237	TGCTGTTG/	CTCTCTCTG	AK081287	hypothetical protein C030046G05		2.6	SM2119	g	1	1.522164293
mPool7_V2MM_178545	2.31982	2.205692	TRUE	HP_399019	TGCTGTTG/	GCTTAGAT(	XM_288180	Mus musculus LOC332444 (LOC332444), mR.		2.5	SM2092	f	11	1.656455229
mPool7_V2MM_178545	2.31982	2.205692	TRUE	HP_399019	TGCTGTTG/	GCTTAGAT(	XM_288180	Mus musculus LOC332444 (LOC332444), mRNA.		2.5	SM2092	f	11	1.656455229
mPool2_V2MM_127435	2.319779	3.004939	TRUE	HP_348261	TGCTGTTG/	CTAGGAAT/	XM_145191	Mus musculus similar to fatty acid binding protein (heart) like [Bos taurus] (LOC214278		2.11	SM2359	f	7	1.522164293
mPool4_V2MM_205800	2.318956	62.64426	TRUE	HP_423650	TGCTGTTG/	GCCTGTTT(	AK010497	RIKEN cDNA 2410015J15 gene	2410015J15Rik	2.16	SM2614	h	2	0.203118686
mPool2_V2MM_21542	2.318763	3.004939	TRUE	HP_244713	TGCTGTTG/	CAGTTGAC/	NM_023530	phospholipase A2, group XIIB	Pla2g12b	2.11	SM2392	c	7	1.522164293
mPool2_V2MM_134459	2.317929	2.205692	TRUE	HP_355185	TGCTGTTG/	CTAGAACT(	XM_149889	Mus musculus LOC232903 (LOC232903), mRNA.		2.11	SM2354	d	11	1.656455229
092308m1_V2MM_127416	2.3129	2.205692	TRUE	HP_348242	TGCTGTTG/	CAGAGACC	XM_145185	similar to glyceraldehyde-3-phosphate dehydrogese (phosphorylating) (EC 1.2.1.12) - m		2.6	SM2102	b	4	1.656455229
mPool2_V2MM_131495	2.312512	2.205692	TRUE	HP_352258	TGCTGTTG/	GTTATATA/	XM_147679	Mus musculus LOC209126 (LOC209126), mRNA.		2.11	SM2353	d	4	1.656455229
mPool7_V2MM_184354	2.311222	2.205692	TRUE	HP_404826	TGCTGTTG/	CAGCATTT(	XM_289184	Mus musculus LOC332804 (LOC332804), mR.		2.5	SM2069	f	4	1.656455229
mPool7_V2MM_184354	2.311222	2.205692	TRUE	HP_404826	TGCTGTTG/	CAGCATTT(	XM_289184	Mus musculus LOC332804 (LOC332804), mRNA.		2.5	SM2069	f	4	1.656455229
mPool2_V2MM_115702	2.309701	3.004939	TRUE	HP_336663	TGCTGTTG/	GCTGGAAC	XM_138606	similar to glyceraldehyde-3-phosphate dehydrogenase		2.1	SM2323	c	1	1.522164293
mPool2_V2MM_131249	2.309261	3.004939	TRUE	HP_352013	TGCTGTTG/	GTTGATTA(	AK015910	RIKEN cDNA 4930527B16 gene	4930527B16Rik	2.1	SM2347	b	8	1.522164293
mPool2_V2MM_85581	2.308452	2.205692	TRUE	HP_307062	TGCTGTTG/	GCTGAACT(	NM_146417	olfactory receptor 877	Olfr877	2.6	SM2143	h	4	1.656455229
mPool2_V2MM_132910	2.3083	3.004939	TRUE	HP_353657	TGCTGTTG/	GGCATTCT1	BC020411	RIKEN cDNA 2700010L10 gene	2700010L10Rik	2.11	SM2370	d	10	1.522164293
mPool7_V2MM_171663	2.308269	2.205692	TRUE	HP_392150	TGCTGTTG/	CATATTCTT	AK078525	activin A receptor, type 1B	Acvr1b	2.5	SM2062	a	5	1.656455229
mPool7_V2MM_171663	2.308269	2.205692	TRUE	HP_392150	TGCTGTTG/	CATATTCTT	AK078525	activin A receptor, type 1B	Acvr1b	2.5	SM2062	a	5	1.656455229
mPool2_V2MM_213136	2.305802	2.205692	TRUE	HP_430781	TGCTGTTG/	CCTTGAAT(	AK029061	RIKEN cDNA 1700020O03 gene	1700020O03Rik	2.11	SM2366	f	5	1.656455229
mPool2_V2MM_101248	2.302802	2.205692	TRUE	HP_322420	TGCTGTTG/	CTGACTTC(	AK083705	expressed sequence AI256775	AI256775	2.7	SM2157	c	1	1.656455229
mPool2_V2MM_5330	2.299528	3.004939	TRUE	HP_228882	TGCTGTTG/	CCCAAGAT(	NM_007560	bone morphogenetic protein receptor, type 1B	Bmpr1b	2.11	SM2378	e	1	1.522164293
mPool2_V2MM_164305	2.297209	3.004939	TRUE	HP_384855	TGCTGTTG/	CTGGATTG/	XM_284973	Mus musculus similar to thyroid hormone receptor-associated protein, 150 kDa subunit		2.6	SM2130	f	7	1.522164293
092308m1_V2MM_95502	2.297144	2.205692	TRUE	HP_316778	TGCTGTTG/	GGCAATTT/	NM_198017	RIKEN cD C430003P19 gene	C430003P19Rik	2.6	SM2112	h	5	1.656455229
092308m1_V2MM_174732	2.294221	2.205692	TRUE	HP_395217	TGCTGTTG/	GCAGTAAA	AK034327	hypothetical protein 9330178D15		2.9	SM2277	f	7	1.656455229
092308m1_V2MM_174732	2.294221	2.205692	TRUE	HP_395217	TGCTGTTG/	GCAGTAAA	AK034327	hypothetical protein 9330178D15		2.9	SM2277	f	7	1.656455229
092308m1_V2MM_175229	2.294108	3.004939	TRUE	HP_395714	TGCTGTTG/	CATCGAAG(	XM_287395	Mus musculus hypothetical gene supported by AK038877 (LOC329949), mR.		2.5	SM2071	b	4	1.522164293
092308m1_V2MM_91379	2.291987	2.205692	TRUE	HP_312758	TGCTGTTG/	CATACACT(	BC052827	Unknown (protein for IMAGE:30033377)		2.6	SM2113	a	6	1.656455229
mPool7_V2MM_78352	2.291468	3.004939	TRUE	HP_300041	TGCTGTTG/	GACCTATG(	NM_029624	RIKEN cDNA 2400010G15 gene	2400010G15Rik	2.8	SM2243	h	7	1.522164293
mPool2_V2MM_132669	2.290049	2.205692	TRUE	HP_353419	TGCTGTTG/	CACAGTTC(	AK014914	RIKEN cDNA 4921517O11 gene	4921517O11Rik	2.11	SM2367	e	3	1.656455229
mPool2_V2MM_133624	2.287794	2.205692	TRUE	HP_354358	TGCTGTTG/	CTGACATC1	AK014550	RIKEN cDNA 4632404M16 gene	4632404M16Rik	2.11	SM2358	d	4	1.656455229
mPool7_V2MM_117028	2.286222	1.959154	TRUE	HP_337976	TGCTGTTG/	CTGCTGCA(	XM_139196	Mus musculus similar to Hypothetical protein KIAA1002 (LOC219081), mRNA.		2.9	SM2299	e	9	1.707931489
mPool2_V2MM_109420	2.275413	2.205692	TRUE	HP_330474	TGCTGTTG/	CTTCCAAG1	XM_133877	RIKEN cDNA 4933402N03 gene	4933402N03Rik	2.7	SM2152	f	1	1.656455229
mPool2_V2MM_30402	2.274178	2.205692	TRUE	HP_253331	TGCTGTTG/	CGATGAAT	AK083102	RIKEN cDNA C630015F21 gene	C630015F21Rik	2.11	SM2392	g	6	1.656455229
mPool7_V2MM_179548	2.273032	2.205692	TRUE	HP_400022	TGCTGTTG/	CCTACAAT(	XM_288352	Mus musculus LOC331672 (LOC331672), mRNA.		2.5	SM2091	d	5	1.656455229
mPool7_V2MM_179548	2.273032	2.205692	TRUE	HP_400022	TGCTGTTG/	CCTACAAT(	XM_288352	Mus musculus LOC331672 (LOC331672), mR.		2.5	SM2091	d	5	1.656455229
mPool2_V2MM_169959	2.259909	3.004939	TRUE	HP_390454	TGCTGTTG/	CAGGGTAT(	XM_286366	Mus musculus hypothetical gene supported by AK050116 (LOC327909), mRNA.		2.6	SM2128	e	3	1.522164293
092308m1_V2MM_148639	2.256828	2.205692	TRUE	HP_369350	TGCTGTTG/	CTCCCTGC/	XM_163974	Mus musculus LOC244370 (LOC244370), mR.		2.4	SM2042	b	5	1.656455229
mPool7_V2MM_171303	2.256793	2.205692	TRUE	HP_391792	TGCTGTTG/	CTGCTCAC(	AK084007	RIKEN cDNA D130076A03 gene	D130076A03Rik	2.9	SM2272	c	3	1.656455229
mPool2_V2MM_101731	2.251934	3.004939	TRUE	HP_322896	TGCTGTTG/	CACTGTTA(	XM_127850	gene model 293, (NCBI)	Gm293	2.7	SM2157	c	3	1.522164293
mPool2_V2MM_136024	2.25192	2.205692	TRUE	HP_356744	TGCTGTTG/	GGCTCTGA(	XM_151323	Mus musculus LOC211516 (LOC211516), mRNA.		2.1	SM2335	h	9	1.656455229
mPool2_V2MM_6131	2.248339	2.205692	TRUE	HP_229671	TGCTGTTG/	CTCAGTAG(	NM_010789	myeloid ecotropic viral integration site 1	Meis1	2.11	SM2388	g	5	1.656455229
mPool2_V2MM_208005	2.245995	2.205692	TRUE	HP_425802	TGCTGTTG/	GTACTATA1	XM_489317	LOC434549		2.1	SM2336	g	4	1.656455229
092308m1_V2MM_155480	2.244592	3.004939	TRUE	HP_376119	TGCTGTTG/	CTCAGTAA(	XM_197476	Mus musculus LOC271802 (LOC271802), mR.		2.6	SM2123	d	4	1.522164293
mPool2_V2MM_3044	2.242759	2.205692	TRUE	HP_226651	TGCTGTTG/	CTTATCTCC	NM_007664	cadherin 2	Cdh2	2.7	SM2166	d	11	1.656455229
mPool2_V2MM_122581	2.240322	3.004939	TRUE	HP_343445	TGCTGTTG/	GAGGTTAT(	XM_488394	similar to 40S ribosomal protein SA (P40) (34/67 kDa laminin receptor)		2.11	SM2354	f	11	1.522164293
092308m1_V2MM_66431	2.237994	3.004939	TRUE	HP_288447	TGCTGTTG/	CAGAGACT(	NM_172991	RIKEN cD C030048B08 gene	C030048B08Rik	2.3	SM2008	e	3	1.522164293
mPool7_V2MM_100261	2.237624	3.004939	TRUE	HP_321453	TGCTGTTG/	CAATTCATT	XM_126724	fetal Alzheimer antigen	Falz	2.7	SM2152	c	9	1.522164293
092308m1_V2MM_171557	2.237398	2.205692	TRUE	HP_392046	TGCTGTTG/	GAAATGTT(	XM_286648	Mus musculus hypothetical gene supported by AK037505 (LOC328492), mR.		2.5	SM2078	e	1	1.656455229
mPool7_V2MM_195100	2.236993	2.205692	TRUE	HP_413249	TGCTGTTG/	CTGTATAT/	NM_146632	olfactory receptor 116	Olfr116	2.9	SM2254	f	8	1.656455229
mPool7_V2MM_196394	2.234656	3.004939	TRUE	HP_414499	TGCTGTTG/	CCGTAAAT(	NM_026505	BMP and activin membrane-bound inhibitor, homolog (Xenop	Bambi	2.9	SM2266	a	7	1.522164293
092308m1_V2MM_142501	2.234342	3.004939	TRUE	HP_363214	TGCTGTTG/	GACACTCA(	XM_157672	similar to Suppressor of G2 allele of SKP1 homolog (Sgt1) (Putative 40-6-3 protein)		2.4	SM2049	d	8	1.522164293
mPool2_V2MM_101888	2.234022	2.205692	TRUE	HP_323051	TGCTGTTG/	GCGCCTTT(	NM_027707	RIKEN cDNA 4933421G18 gene	4933421G18Rik	2.1	SM2309	h	11	1.656455229
mPool2_V2MM_16861	2.233186	2.205692	TRUE	HP_240149	TGCTGTTG/	GACTGGTT/	NM_008268	homeo box B5	Hoxb5	2.11	SM2389	f	4	1.656455229
mPool7_V2MM_179034	2.232598	3.004939	TRUE	HP_399508	TGCTGTTG/	CAGGATAT(	XM_288262	Mus musculus LOC329282 (LOC329282), mR.		2.5	SM2089	b	6	1.522164293

mPool7_V2MM_179034	2.232598	3.004939	TRUE	HP_399508	TGCTGTTG/	CAGGATAT(	XM_288262	Mus musculus LOC329282 (LOC329282), mRNA.			2.5	SM2089	b		6	1.522164293
mPool2_V2MM_159712	2.232264	2.205692	TRUE	HP_380305	TGCTGTTG/	CAGGAAGT	XM_283222	Mus musculus hypothetical gene supported by AK034489 (LOC328439), mRNA.			2.6	SM2126	a		7	1.656455229
092308m1_V2MM_158630	2.229339	3.004939	TRUE	HP_379240	TGCTGTTG/	CAGCCTTA/	XM_206878	Mus musculus LOC279358 (LOC279358), mR.			2.6	SM2116	g		9	1.522164293
mPool2_V2MM_93146	2.226003	2.205692	TRUE	HP_314478	TGCTGTTG/	GTGGATAA	AK083705	expressed sequence AI256775	AI256775		2.6	SM2110	a		2	1.656455229
mPool2_V2MM_93146	2.226003	2.205692	TRUE	HP_314478	TGCTGTTG/	GTGGATAA	AK083705	expressed sequence AI256775	AI256775		2.6	SM2110	a		2	1.656455229
mPool2_V2MM_8740	2.223547	3.004939	TRUE	HP_232220	TGCTGTTG/	CTTTGCATC	NM_027137	small proline rich-like 7	Sprrl7		2.11	SM2384	c		2	1.522164293
mPool2_V2MM_97667	2.223155	2.205692	TRUE	HP_318907	TGCTGTTG/	GAAGGGA/	XM_111809	Mus musculus LOC241767 (LOC241767), mRNA.			2.6	SM2144	d		3	1.656455229
mPool2_V2MM_125139	2.221033	3.004939	TRUE	HP_345983	TGCTGTTG/	GATCTTACT	XM_143811	gene model 427, (NCBI)	Gm427		2.1	SM2349	b		7	1.522164293
mPool2_V2MM_113519	2.220567	3.004939	TRUE	HP_334506	TGCTGTTG/	CTTGCTCTC	XM_137005	Mus musculus similar to Hypothetical protein KIAA0144 (LOC212406), mRNA.			2.7	SM2154	f		2	1.522164293
mPool7_V2MM_205096	2.220502	3.004939	TRUE	HP_422972	TGCTGTTG/	CTCTGTAA(	XM_136300	Mus musculus similar to MRC OX-45 surface antigen precursor (BCM1 surface antigen) (			2.1	SM2313	h		6	1.522164293
mPool2_V2MM_15479	2.220285	3.004939	TRUE	HP_238802	TGCTGTTG/	CTCTTATCT	AK047140	MAP/microtubule affinity-regulating kinase 1	Mark1		2.11	SM2383	b		1	1.522164293
092308m1_V2MM_70919	2.215476	3.004939	TRUE	HP_292820	TGCTGTTG/	CAGCTTCA(	NM_146796	olfactory receptor 1499	Olfr1499		2.4	SM2022	a		8	1.522164293
092308m1_V2MM_144230	2.213874	3.004939	TRUE	HP_364941	TGCTGTTG/	CATTGTAC(	XM_159825	Mus musculus LOC207745 (LOC207745), mRNA.			2.5	SM2058	a		5	1.522164293
092308m1_V2MM_144230	2.213874	3.004939	TRUE	HP_364941	TGCTGTTG/	CATTGTAC(	XM_159825	Mus musculus LOC207745 (LOC207745), mR.			2.5	SM2058	a		5	1.522164293
092308m1_V2MM_184599	2.213551	3.004939	TRUE	HP_405071	TGCTGTTG/	CACTTTCTA	XM_289225	Mus musculus LOC332850 (LOC332850), mRNA.			2.9	SM2290	e		12	1.522164293
092308m1_V2MM_184599	2.213551	3.004939	TRUE	HP_405071	TGCTGTTG/	CACTTTCTA	XM_289225	Mus musculus LOC332850 (LOC332850), mR.			2.9	SM2290	e		12	1.522164293
092308m1_V2MM_148845	2.212773	2.205692	TRUE	HP_369556	TGCTGTTG/	CACGTTTA(	XM_164238	Mus musculus LOC244499 (LOC244499), mR.			2.5	SM2055	b		6	1.656455229
mPool2_V2MM_214871	2.209404	2.205692	TRUE	HP_432443	TGCTGTTG/	CCTGATTTC	XM_143387	Mus musculus similar to 60S ribosomal protein L21 (LOC242101), mRNA.			2.11	SM2356	e		6	1.656455229
mPool7_V2MM_199693	2.207545	3.004939	TRUE	HP_417733	TGCTGTTG/	CAGGTTGA(	AK086149	RIKEN cDNA A730017D01 gene	A730017D01Rik		2.9	SM2291	c		11	1.522164293
092308m1_V2MM_173562	2.204047	2.205692	TRUE	HP_394048	TGCTGTTG/	CACACAGT	AK048422	hypothetical protein C130058N19			2.5	SM2086	e		11	1.656455229
092308m1_V2MM_170236	2.202563	2.205692	TRUE	HP_390731	TGCTGTTG/	CGTTTATG/	XM_286417	Mus musculus hypothetical gene supported by AK036142 (LOC328071), mR.			2.6	SM2116	g		8	1.656455229
mPool2_V2MM_19590	2.201509	3.004939	TRUE	HP_242806	TGCTGTTG/	GGTTGGAA	NM_152229	nuclear receptor subfamily 2, group E, member 1	Nr2e1		2.7	SM2164	f		3	1.522164293
mPool2_V2MM_135781	2.201352	3.004939	TRUE	HP_356503	TGCTGTTG/	CTGTTGTTC	XM_151047	Mus musculus LOC211853 (LOC211853), mRNA.			2.1	SM2343	a		10	1.522164293
092308m1_V2MM_125703	2.198339	2.205692	TRUE	HP_346546	TGCTGTTG/	GGCCTTAC/	XM_144185	Mus musculus similar to hypothetical protein C230069C04 (LOC242842), mR.			2.5	SM2099	c		11	1.656455229
mPool7_V2MM_174650	2.19784	3.004939	TRUE	HP_395135	TGCTGTTG/	CTCTTCTTA	AK082502	RIKEN cDNA C230057H02 gene	C230057H02Rik		2.5	SM2089	h		10	1.522164293
mPool7_V2MM_174650	2.19784	3.004939	TRUE	HP_395135	TGCTGTTG/	CTCTTCTTA	AK082502	RIKEN cD C230057H02 gene	C230057H02Rik		2.5	SM2089	h		10	1.522164293
mPool7_V2MM_174555	2.197047	2.205692	TRUE	HP_395040	TGCTGTTG/	GTTTATAG(	XM_287252	Mus musculus hypothetical gene supported by AK084244 (LOC329673), mRNA.			2.9	SM2291	e		4	1.656455229
mPool7_V2MM_204410	2.197023	3.004939	TRUE	HP_422310	TGCTGTTG/	GGTCTACG(	XM_139317	Mus musculus similar to Metal-response element-binding transcription factor 2 (Metal-r			2.9	SM2298	d		8	1.522164293
mPool2_V2MM_118884	2.196167	3.004939	TRUE	HP_339814	TGCTGTTG/	GTACCTCA(	XM_140107	RIKEN cDNA 1700090G07 gene	1700090G07Rik		2.11	SM2373	b		9	1.522164293
mPool7_V2MM_198063	2.196087	2.205692	TRUE	HP_416124	TGCTGTTG/	GCTGTAGC/	XM_287341	Mus musculus hypothetical gene supported by AK080995 (LOC329843), mRNA.			2.9	SM2295	d		8	1.656455229
mPool2_V2MM_169542	2.192794	3.004939	TRUE	HP_390037	TGCTGTTG/	GCTCAGAA/	XM_286284	Mus musculus hypothetical gene supported by AK049492 (LOC327825), mRNA.			2.6	SM2126	g		3	1.522164293
092308m1_V2MM_76661	2.192463	2.205692	TRUE	HP_298412	TGCTGTTG/	CTACTGTTG	NM_146732	olfactory receptor 488	Olfr488		2.4	SM2025	c		4	1.656455229
092308m1_V2MM_85838	2.188618	2.205692	TRUE	HP_307318	TGCTGTTG/	CACAATCA(	NM_146487	olfactory receptor 130	Olfr130		2.6	SM2107	f		5	1.656455229
mPool2_V2MM_133964	2.187805	3.004939	TRUE	HP_354694	TGCTGTTG/	CTATGGAT/	AK034038	RIKEN cDNA 2310058O09 gene	2310058O09Rik		2.5	SM2100	a		2	1.522164293
mPool2_V2MM_133964	2.187805	3.004939	TRUE	HP_354694	TGCTGTTG/	CTATGGAT/	AK034038	RIKEN cD 2310058O09 gene	2310058O09Rik		2.5	SM2100	a		2	1.522164293
mPool2_V2MM_159312	2.187191	2.205692	TRUE	HP_379909	TGCTGTTG/	GTATGTTAT	XM_283051	Mus musculus hypothetical gene supported by AK048793 (LOC328053), mRNA.			2.6	SM2129	c		8	1.656455229
092308m1_V2MM_77667	2.186486	1.959154	TRUE	HP_299380	TGCTGTTG/	CTGTTTGTA	NM_029478	RIKEN cD 4930579A11 gene	4930579A11Rik		2.3	SM2016	d		8	1.707931489
mPool7_V2MM_67301	2.186257	3.004939	TRUE	HP_289292	TGCTGTTG/	CCTCAAGA(	NM_025555	RIKEN cDNA 2410004B18 gene	2410004B18Rik		2.8	SM2231	b		2	1.522164293
mPool2_V2MM_157803	2.182956	3.004939	TRUE	HP_378416	TGCTGTTG/	GGCAAATG	XM_205638	Mus musculus similar to hypothetical protein FLJ38725 [Homo sapiens] (LOC277953), m			2.6	SM2127	g		11	1.522164293
mPool7_V2MM_139690	2.181734	2.205692	TRUE	HP_360410	TGCTGTTG/	CAGACAAT(	XM_155311	Mus musculus LOC211071 (LOC211071), mRNA.			2.5	SM2056	f		9	1.656455229
mPool7_V2MM_139690	2.181734	2.205692	TRUE	HP_360410	TGCTGTTG/	CAGACAAT(	XM_155311	Mus musculus LOC211071 (LOC211071), mR.			2.5	SM2056	f		9	1.656455229
092308m1_V2MM_157112	2.181489	3.004939	TRUE	HP_377733	TGCTGTTG/	CCGAAGTC	XM_204889	Mus musculus similar to nuclear domain 10 protein [Homo sapiens] (LOC278732), mR.			2.6	SM2116	e		6	1.522164293
092308m1_V2MM_83357	2.18136	3.004939	TRUE	HP_304902	TGCTGTTG/	CGCAGTAA	NM_013511	erythrocyte protein band 4.1-like 2	Epb4.1l2		2.6	SM2113	a		2	1.522164293
mPool2_V2MM_31619	2.17997	3.004939	TRUE	HP_254515	TGCTGTTG/	CTTATTGCT	NM_019911	tryptophan 2,3-dioxygenase	Tdo2		2.11	SM2389	e		6	1.522164293
mPool2_V2MM_206238	2.179866	2.205692	TRUE	HP_424075	TGCTGTTG/	CTGGAACT	XM_138606	similar to glyceraldehyde-3-phosphate dehydrogenase			2.1	SM2318	h		12	1.656455229
mPool4_V2MM_160042	2.179119	75.83547	TRUE	NA	NA	NA	NA	NA	NA	NA		NA	NA	NA		0.120127608
092308m1_V2MM_73701	2.178346	2.205692	TRUE	HP_295527	TGCTGTTG/	GTAATCTA(	NM_175034	solute carrier family 24, member 5	Slc24a5		2.4	SM2022	h		3	1.656455229
mPool2_V2MM_212273	2.176676	2.205692	TRUE	HP_429943	TGCTGTTG/	GATAGAAC	XM_141423	Mus musculus LOC241709 (LOC241709), mRNA.			2.1	SM2350	b		11	1.656455229
mPool2_V2MM_1146	2.176472	2.205692	TRUE	HP_224796	TGCTGTTG/	CATATTGAT	NM_030611	aldo-keto reductase family 1, member C6	Akr1c6		2.11	SM2377	h		7	1.656455229
092308m1_V2MM_132762	2.173996	3.004939	TRUE	HP_353512	TGCTGTTG/	CTCCAGAC(	XM_148876	Mus musculus LOC225654 (LOC225654), mR.			2.6	SM2101	h		7	1.522164293
mPool2_V2MM_205508	2.173072	2.205692	TRUE	NA	NA	NA	NA	NA	NA	NA		NA	NA	NA		1.656455229
092308m1_V2MM_124416	2.172018	3.004939	TRUE	HP_345268	TGCTGTTG/	GATCCAAT(	XM_143434	Mus musculus similar to ribosomal protein L34 [Rattus rattus] (LOC229740), mR.			2.6	SM2114	h		3	1.522164293
092308m1_V2MM_161647	2.171355	2.205692	TRUE	HP_382211	TGCTGTTG/	CATTCATAC	XM_283996	Mus musculus hypothetical gene supported by AK052563 (LOC329911), mR.			2.6	SM2118	b		8	1.656455229
mPool7_V2MM_73979	2.169804	2.205692	TRUE	HP_295796	TGCTGTTG/	GTTGTAAG/	NM_010721	lamin B1	Lmnb1		2.8	SM2244	d		10	1.656455229
092308m1_V2MM_164671	2.169254	3.004939	TRUE	HP_385214	TGCTGTTG/	GGCTTAAG/	XM_487308	similar to 40S ribosomal protein S2			2.6	SM2119	b		1	1.522164293
092308m1_V2MM_169628	2.168786	2.205692	TRUE	HP_390123	TGCTGTTG/	GCAGTACT(	AK081017	hypothetical protein B930054O08			2.6	SM2119	h		9	1.656455229
092308m1_V2MM_162965	2.167877	3.004939	TRUE	HP_383523	TGCTGTTG/	CTGCTCTGT	XM_284565	Mus musculus hypothetical gene supported by AK044672 (LOC331077), mR.			2.6	SM2116	b		2	1.522164293
mPool7_V2MM_88730	2.165839	1.959154	TRUE	HP_310166	TGCTGTTG/	CTTATGAGT	NM_175477	zinc finger protein 574	Zfp574		2.9	SM2255	a		8	1.707931489
mPool7_V2MM_103978	2.165484	2.205692	TRUE	HP_325115	TGCTGTTG/	GTTGGAGT	AK011565	RIKEN cDNA 2610027F03 gene	2610027F03Rik		2.1	SM2304	g		5	1.656455229
092308m1_V2MM_120180	2.162264	2.205692	TRUE	HP_341082	TGCTGTTG/	CTCTCCTAA	XM_140790	similar to Rpl7a protein			2.6	SM2110	a		12	1.656455229
092308m1_V2MM_120180	2.162264	2.205692	TRUE	HP_341082	TGCTGTTG/	CTCTCCTAA	XM_140790	similar to Rpl7a protein			2.6	SM2110	a		12	1.656455229
mPool7_V2MM_78895	2.16209	2.205692	TRUE	HP_300570	TGCTGTTG/	GAGGATAT	NM_026029	RIKEN cDNA 2700085E05 gene	2700085E05Rik		2.8	SM2244	b		10	1.656455229

mPool7_V2MM_111145	2.161757	2.205692	TRUE	HP_377019	TGCTGTTG/	CCCATTTAA	XM_484460	similar to 2610510D13Rik protein			2.6	SM2114	c		6	1.656455229
092308m3_V2MM_55457	2.161418	75.83547	TRUE	NA	NA	NA	NA	NA	NA	NA	NA	NA	NA	NA		0.120127608
mPool2_V2MM_212428	2.152989	3.004939	TRUE	HP_430094	TGCTGTTG/	GAGGAAAT	AK082964	cAMP responsive element binding protein 3-like 2	Creb3l2		2.11	SM2359	d		1	1.522164293
mPool7_V2MM_95623	2.152689	1.959154	TRUE	HP_316897	TGCTGTTG/	CAAGTAAG.	NM_198108	cDNA sequence BC023055	BC023055		2.8	SM2250	e		12	1.707931489
mPool7_V2MM_95623	2.152689	1.959154	TRUE	HP_316897	TGCTGTTG/	CAAGTAAG.	NM_198108	cD sequence BC023055	BC023055		2.8	SM2250	e		12	1.707931489
mPool2_V2MM_10140	2.151917	1.959154	TRUE	HP_233582	TGCTGTTG/	CTAATAAA(	NM_007853	degenerative spermatocyte homolog (Drosophila)	Degs		2.11	SM2386	h		11	1.707931489
mPool7_V2MM_171488	2.150559	3.004939	TRUE	HP_391977	TGCTGTTG/	CCAATAAT(	XM_286635	Mus musculus hypothetical gene supported by AK040469 (LOC328470), mRNA.			2.9	SM2271	c		7	1.522164293
mPool7_V2MM_176364	2.149908	1.959154	TRUE	HP_396846	TGCTGTTG/	CATTCATTC	XM_287651	Mus musculus hypothetical gene supported by AK079645 (LOC330422), mRNA.			2.5	SM2079	b		1	1.707931489
mPool7_V2MM_176364	2.149908	1.959154	TRUE	HP_396846	TGCTGTTG/	CATTCATTC	XM_287651	Mus musculus hypothetical gene supported by AK079645 (LOC330422), mR.			2.5	SM2079	b		1	1.707931489
mPool2_V2MM_132821	2.149411	2.205692	TRUE	HP_353570	TGCTGTTG/	GGAGCTAA	XM_148904	oxysterol binding protein	Osbp		2.11	SM2368	d		10	1.656455229
092308m1_V2MM_154888	2.148857	2.205692	TRUE	HP_375529	TGCTGTTG/	CACTTATTG	XM_197073	RIKEN cD 9330128J19 gene	9330128J19Rik		2.6	SM2124	a		10	1.656455229
092308m1_V2MM_73153	2.147094	2.205692	TRUE	HP_294994	TGCTGTTG/	GCATATCT(	NM_026197	RIKEN cD 2810013M15 gene	2810013M15Rik		2.3	SM2012	e		10	1.656455229
mPool7_V2MM_67969	2.146374	2.205692	TRUE	HP_289941	TGCTGTTG/	CTGAATTTA	NM_028815	leucine-rich repeats and IQ motif containing 2	Lrriq2		2.8	SM2234	d		10	1.656455229
mPool2_V2MM_112469	2.143769	3.004939	TRUE	HP_333473	TGCTGTTG/	GGCAATCT(	XM_355265	similar to usherin isoform B			2.7	SM2151	a		12	1.522164293
092308m1_V2MM_175866	2.140792	2.205692	TRUE	HP_396348	TGCTGTTG/	CAGCTAGT/	XM_287544	Mus musculus hypothetical gene supported by AK035359 (LOC330238), mR.			2.5	SM2060	b		4	1.656455229
092308m1_V2MM_174286	2.139789	1.959154	TRUE	HP_394771	TGCTGTTG/	GTTATAAC/	XM_287196	Mus musculus hypothetical gene supported by AK034924 (LOC329550), mR.			2.5	SM2076	a		10	1.707931489
092308m1_V2MM_171386	2.137622	2.205692	TRUE	HP_391875	TGCTGTTG/	GGAGTTTG.	AK053660	hypothetical protein E130119H08			2.5	SM2068	c		5	1.656455229
mPool2_V2MM_124518	2.136591	3.004939	TRUE	HP_345370	TGCTGTTG/	GGTACTAT(	XM_143495	ribosomal protein S20	Rps20		2.11	SM2357	g		2	1.522164293
092308m1_V2MM_174338	2.136563	2.205692	TRUE	HP_394823	TGCTGTTG/	CACACAGC	AK083044	RIKEN cD C530038A18 gene	C530038A18Rik		2.5	SM2087	g		8	1.656455229
mPool7_V2MM_204042	2.134788	3.004939	TRUE	HP_421960	TGCTGTTG/	GAACCTGA.	AK015711	RIKEN cDNA 4930505O20 gene	4930505O20Rik		2.1	SM2317	d		7	1.522164293
092308m1_V2MM_174678	2.132732	3.004939	TRUE	HP_395163	TGCTGTTG/	CTAAGTTTC	XM_287282	Mus musculus hypothetical gene supported by AK041457 (LOC329726), mR.			2.5	SM2062	g		2	1.522164293
mPool7_V2MM_80803	2.129937	3.004939	TRUE	HP_302427	TGCTGTTG/	GGCAAATT/	NM_028796	RIKEN cDNA 1700012B15 gene	1700012B15Rik		2.8	SM2248	h		2	1.522164293
mPool2_V2MM_106336	2.127353	2.205692	TRUE	HP_327439	TGCTGTTG/	GTGATAAA(	AK004863	alcohol dehydrogenase 6 (class V), pseudogene 1	Adh6-ps1		2.7	SM2163	d		6	1.656455229
092308m1_V2MM_126821	2.122218	3.004939	TRUE	HP_347659	TGCTGTTG/	GAAATCAG.	XM_144829	immunoglobulin kappa chain variable 28 (V28)	Igk-V28		2.6	SM2103	b		5	1.522164293
092308m1_V2MM_126821	2.122218	3.004939	TRUE	HP_347659	TGCTGTTG/	GAAATCAG.	XM_144829	immunoglobulin kappa chain variable 28 (V28)	Igk-V28		2.6	SM2103	b		5	1.522164293
mPool2_V2MM_136270	2.119626	3.004939	TRUE	HP_356990	TGCTGTTG/	GGAACAAG	XM_151594	Mus musculus LOC213763 (LOC213763), mR.			2.4	SM2039	h		8	1.522164293
mPool2_V2MM_136270	2.119626	3.004939	TRUE	HP_356990	TGCTGTTG/	GGAACAAG	XM_151594	Mus musculus LOC213763 (LOC213763), mRNA.			2.4	SM2039	h		8	1.522164293
092308m1_V2MM_156971	2.116529	1.959154	TRUE	HP_377595	TGCTGTTG/	GCCATTAT(	XM_204668	similar to growth arrest-specific 2 like 2; GAS2-related protein			2.6	SM2115	f		3	1.707931489
mPool7_V2MM_80858	2.11584	3.004939	TRUE	HP_302481	TGCTGTTG/	GGCAGAGT	NM_025765	RIKEN cDNA 4933437N03 gene	4933437N03Rik		2.8	SM2240	f		9	1.522164293
mPool2_V2MM_211508	2.115203	3.004939	TRUE	HP_429204	TGCTGTTG/	GTTGGATG(	XM_139696	Mus musculus similar to 60S RIBOSOMAL PROTEIN L9 (LOC224311), mRNA.			2.1	SM2349	a		6	1.522164293
mPool7_V2MM_116075	2.114921	2.205692	TRUE	HP_337030	TGCTGTTG/	GTGCTTATT	XM_138743	Mus musculus similar to Gag [Ovis aries] (LOC218358), mRNA.			2.7	SM2154	e		2	1.656455229
mPool2_V2MM_160396	2.112521	2.205692	TRUE	HP_380976	TGCTGTTG/	CTGGCTAA(	AK080941	ankyrin repeat and KH domain containing 1	Ankhd1		2.6	SM2128	b		3	1.656455229
mPool7_V2MM_204277	2.109901	2.205692	TRUE	HP_422181	TGCTGTTG/	CTGCTTATC	AK019136	RIKEN cDNA 2410150O07 gene	2410150O07Rik		2.9	SM2299	g		1	1.656455229
092308m1_V2MM_121702	2.107362	2.205692	TRUE	HP_342580	TGCTGTTG/	GCAAGAAT	XM_141796	Mus musculus similar to RIKEN cD 4933402E13 [Mus musculus] (LOC236842), mR.			2.5	SM2099	d		3	1.656455229
092308m1_V2MM_121702	2.107362	2.205692	TRUE	HP_342580	TGCTGTTG/	GCAAGAAT	XM_141796	Mus musculus similar to RIKEN cDNA 4933402E13 [Mus musculus] (LOC236842), mRNA.			2.5	SM2099	d		3	1.656455229
092308m1_V2MM_141720	2.106854	3.004939	TRUE	HP_362433	TGCTGTTG/	CCTGATTTG	XM_156900	Mus musculus LOC224946 (LOC224946), mR.			2.4	SM2042	f		4	1.522164293
092308m1_V2MM_133964	2.105841	3.004939	TRUE	HP_354694	TGCTGTTG/	CTATGGAT/	AK034038	RIKEN cD 2310058O09 gene	2310058O09Rik		2.5	SM2100	a		2	1.522164293
092308m1_V2MM_133964	2.105841	3.004939	TRUE	HP_354694	TGCTGTTG/	CTATGGAT/	AK034038	RIKEN cDNA 2310058O09 gene	2310058O09Rik		2.5	SM2100	a		2	1.522164293
mPool2_V2MM_91636	2.10493	2.205692	TRUE	HP_313005	TGCTGTTG/	GGACTAAA	AK046533	zinc finger, DHHC domain containing 2	Zdhhc2		2.6	SM2144	d		6	1.656455229
092308m1_V2MM_117680	2.104055	2.205692	TRUE	HP_338621	TGCTGTTG/	GGGATAAG	XM_139486	Mus musculus LOC239417 (LOC239417), mR.			2.5	SM2097	a		1	1.656455229
mPool2_V2MM_100261	2.103912	3.004939	TRUE	HP_321453	TGCTGTTG/	CAATTCATT	XM_126724	fetal Alzheimer antigen	Falz		2.7	SM2152	c		9	1.522164293
mPool4_V2MM_22779	2.102706	75.83547	TRUE	NA	NA	NA	NA	NA	NA	NA	NA	NA	NA	NA		0.120127608
mPool7_V2MM_74961	2.102405	3.004939	TRUE	HP_296750	TGCTGTTG/	CATAGATG	NM_175244	RIKEN cDNA 1700064K09 gene	1700064K09Rik		2.8	SM2245	f		3	1.522164293
mPool7_V2MM_144466	2.102218	3.004939	TRUE	HP_365177	TGCTGTTG/	CTACTTCTT	XM_160031	Mus musculus LOC241981 (LOC241981), mR.			2.4	SM2042	e		6	1.522164293
mPool7_V2MM_144466	2.102218	3.004939	TRUE	HP_365177	TGCTGTTG/	CTACTTCTT	XM_160031	Mus musculus LOC241981 (LOC241981), mRNA.			2.4	SM2042	e		6	1.522164293
mPool2_V2MM_189909	2.098125	2.205692	TRUE	HP_258410	TGCTGTTG/	CTATCAAG(	NM_177326	p21 (CDKN1A)-activated kinase 2	Pak2		2.11	SM2391	c		12	1.656455229
mPool7_V2MM_62468	2.097133	2.205692	TRUE	HP_284576	TGCTGTTG/	CAGCTAGA	NM_030218	RIKEN cDNA 9130017N09 gene	9130017N09Rik		2.8	SM2229	d		7	1.656455229
mPool2_V2MM_91761	2.096039	2.205692	TRUE	HP_313130	TGCTGTTG/	CTCATTGTC	NM_178444	EGF-like domain 7	Egfl7		2.6	SM2146	f		10	1.656455229
092308m1_V2MM_147365	2.095732	3.004939	TRUE	HP_368076	TGCTGTTG/	GGTTTAGA/	XM_162901	Mus musculus LOC208785 (LOC208785), mR.			2.4	SM2033	a		6	1.522164293
092308m1_V2MM_165237	2.095658	3.004939	TRUE	HP_385774	TGCTGTTG/	CAAGTATG/	XM_285204	Mus musculus similar to enolase 1, alpha non-neuron; alpha-enolase; 2-phospho-D-glyc			2.6	SM2114	g		9	1.522164293
mPool7_V2MM_200431	2.09333	2.205692	TRUE	HP_418466	TGCTGTTG/	CATCTATAA	XM_287522	Mus musculus hypothetical gene supported by AK046921 (LOC330195), mRNA.			2.9	SM2286	f		6	1.656455229
092308m1_V2MM_88186	2.093269	2.205692	TRUE	HP_309630	TGCTGTTG/	CACTAAAG/	NM_153538	zinc finger, CCHC domain containing 6	Zcchc6		2.6	SM2110	d		6	1.656455229
mPool2_V2MM_83274	2.093266	3.004939	TRUE	HP_304822	TGCTGTTG/	GGTGAAAT	NM_011815	FYN binding protein	Fyb		2.6	SM2132	g		10	1.522164293
092308m1_V2MM_135644	2.090897	2.205692	TRUE	HP_356366	TGCTGTTG/	CATCTATAA	XM_150884	Mus musculus LOC240965 (LOC240965), mR.			2.5	SM2052	e		5	1.656455229
mPool2_V2MM_19518	2.090357	3.004939	TRUE	HP_242734	TGCTGTTG/	GGTGCAAA	NM_134191	vomeronasal 1 receptor, E2	V1re2		2.11	SM2384	f		3	1.522164293
092308m1_V2MM_71999	2.089876	3.004939	TRUE	HP_293866	TGCTGTTG/	CTCGTAAT(	NM_146813	olfactory receptor 651	Olfr651		2.4	SM2028	e		6	1.522164293
mPool2_V2MM_22093	2.088642	1.959154	TRUE	HP_245255	TGCTGTTG/	CCTTTGTTC	NM_024191	ADP-ribosylation factor-like 2 binding protein	Arl2bp		2.11	SM2389	f		7	1.707931489
mPool2_V2MM_96594	2.088408	3.004939	TRUE	HP_317844	TGCTGTTG/	CTGAGGAC	XM_110868	similar to ORF1			2.6	SM2142	d		12	1.522164293
mPool7_V2MM_197067	2.087911	2.205692	TRUE	HP_415152	TGCTGTTG/	CAATCAGA(	NM_173780	Kruppel-like factor 8	Klf8		2.9	SM2258	h		9	1.656455229
mPool7_V2MM_198688	2.087593	2.205692	TRUE	HP_416742	TGCTGTTG/	CTTACTGAA	AK048069	hypothetical protein LOC328277			2.9	SM2296	g		3	1.656455229
mPool7_V2MM_184921	2.087238	3.004939	TRUE	HP_405393	TGCTGTTG/	GAGCTTCT(	XM_289281	Mus musculus LOC332932 (LOC332932), mRNA.			2.5	SM2069	a		6	1.522164293
mPool7_V2MM_184921	2.087238	3.004939	TRUE	HP_405393	TGCTGTTG/	GAGCTTCT(	XM_289281	Mus musculus LOC332932 (LOC332932), mR.			2.5	SM2069	a		6	1.522164293

mPool2_V2MM_205219	2.085947	2.205692	TRUE	HP_423092	TGCTGTTG/	CTCACATTC	XM_138748	Mus musculus similar to envelope protein [Ovis aries] (LOC218362), mRNA.			2.1	SM2319	a		3	1.656455229
mPool2_V2MM_143769	2.082516	2.205692	TRUE	HP_364481	TGCTGTTG/	CTTGATAG(	XM_159101	hypothetical LOC208429			2.1	SM2342	a		9	1.656455229
mPool2_V2MM_143769	2.082516	2.205692	TRUE	HP_364481	TGCTGTTG/	CTTGATAG(	XM_159101	hypothetical LOC208429			2.1	SM2342	a		9	1.656455229
092308m1_V2MM_179819	2.08022	3.004939	TRUE	HP_400293	TGCTGTTG/	CTACTTCAC	XM_288402	Mus musculus LOC327894 (LOC327894), mR.			2.5	SM2090	e		11	1.522164293
092308m1_V2MM_174650	2.078315	3.004939	TRUE	HP_395135	TGCTGTTG/	CTCTTCTTA	AK082502	RIKEN cD C230057H02 gene	C230057H02Rik		2.5	SM2089	h		10	1.522164293
092308m1_V2MM_174650	2.078315	3.004939	TRUE	HP_395135	TGCTGTTG/	CTCTTCTTA	AK082502	RIKEN cDNA C230057H02 gene	C230057H02Rik		2.5	SM2089	h		10	1.522164293
mPool7_V2MM_197174	2.077952	2.205692	TRUE	HP_415254	TGCTGTTG/	GATCATAT(	NM_146693	olfactory receptor 1462	Olfr1462		2.9	SM2260	g		3	1.656455229
092308m1_V2MM_184921	2.077901	3.004939	TRUE	HP_405393	TGCTGTTG/	GAGCTTCT(	XM_289281	Mus musculus LOC332932 (LOC332932), mR.			2.5	SM2069	a		6	1.522164293
092308m1_V2MM_184921	2.077901	3.004939	TRUE	HP_405393	TGCTGTTG/	GAGCTTCT(	XM_289281	Mus musculus LOC332932 (LOC332932), mRNA.			2.5	SM2069	a		6	1.522164293
mPool7_V2MM_201173	2.077697	2.205692	TRUE	HP_419203	TGCTGTTG/	CCTTAAGC(	XM_289648	Mus musculus LOC333392 (LOC333392), mRNA.			2.9	SM2294	b		12	1.656455229
mPool2_V2MM_101746	2.076362	2.205692	TRUE	HP_322911	TGCTGTTG/	GCTGAAGT	XM_127854	A kinase (PRKA) anchor protein 11	Akap11		2.7	SM2152	h		5	1.656455229
092308m1_V2MM_175564	2.075031	2.205692	TRUE	HP_396046	TGCTGTTG/	GCCATTGC	XM_489100	hypothetical gene supported by AK031827			2.5	SM2078	d		9	1.656455229
092308m1_V2MM_175564	2.075031	2.205692	TRUE	HP_396046	TGCTGTTG/	GCCATTGC	XM_489100	hypothetical gene supported by AK031827			2.5	SM2078	d		9	1.656455229
092308m1_V2MM_171663	2.07375	2.205692	TRUE	HP_392150	TGCTGTTG/	CATATTCTT	AK078525	activin A receptor, type 1B	Acvr1b		2.5	SM2062	a		5	1.656455229
092308m1_V2MM_171663	2.07375	2.205692	TRUE	HP_392150	TGCTGTTG/	CATATTCTT	AK078525	activin A receptor, type 1B	Acvr1b		2.5	SM2062	a		5	1.656455229
mPool2_V2MM_208454	2.073222	3.004939	TRUE	HP_426244	TGCTGTTG/	GAGAAACT	XM_160842	Mus musculus LOC242351 (LOC242351), mRNA.			2.1	SM2336	c		5	1.522164293
mPool7_V2MM_70814	2.07193	3.004939	TRUE	HP_292716	TGCTGTTG/	CAGATAAT(	NM_025711	asporin	Aspn		2.8	SM2240	e		4	1.522164293
092308m1_V2MM_173334	2.071353	3.004939	TRUE	HP_393820	TGCTGTTG/	CACAAATT(	XM_287004	Mus musculus hypothetical gene supported by AK081108 (LOC329138), mR.			2.5	SM2067	c		12	1.522164293
mPool2_V2MM_102674	2.07014	3.004939	TRUE	HP_323821	TGCTGTTG/	CCCTTGGA/	AK076291	RIKEN cDNA 4632424B03 gene			2.7	SM2153	b		6	1.522164293
mPool2_V2MM_100923	2.069492	3.004939	TRUE	HP_322099	TGCTGTTG/	CAGGATAT	AK005458	prolactin-like protein N	Prlpn		2.1	SM2324	g		1	1.522164293
mPool7_V2MM_179101	2.06843	3.004939	TRUE	HP_399575	TGCTGTTG/	CCACATTC/	XM_288274	Mus musculus LOC332550 (LOC332550), mRNA.			2.5	SM2092	a		9	1.522164293
mPool7_V2MM_179101	2.06843	3.004939	TRUE	HP_399575	TGCTGTTG/	CCACATTC/	XM_288274	Mus musculus LOC332550 (LOC332550), mR.			2.5	SM2092	a		9	1.522164293
092308m1_V2MM_179268	2.068282	2.205692	TRUE	HP_399742	TGCTGTTG/	CAGTCCTC/	XM_288302	Mus musculus LOC331620 (LOC331620), mR.			2.5	SM2094	b		10	1.656455229
092308m1_V2MM_137537	2.067598	3.004939	TRUE	HP_358257	TGCTGTTG/	CTTAAAGT(	XM_153261	similar to Rho GTPase activating protein 20			2.4	SM2046	h		8	1.522164293
092308m1_V2MM_137537	2.067598	3.004939	TRUE	HP_358257	TGCTGTTG/	CTTAAAGT(	XM_153261	similar to Rho GTPase activating protein 20			2.4	SM2046	h		8	1.522164293
mPool7_V2MM_182263	2.067132	2.205692	TRUE	HP_402737	TGCTGTTG/	CTCATTTAA	XM_288815	Mus musculus LOC332291 (LOC332291), mRNA.			2.9	SM2295	a		8	1.656455229
mPool2_V2MM_3059	2.067122	3.004939	TRUE	HP_226666	TGCTGTTG/	CTTCATTGT	NM_026437	RIKEN cDNA 1810055E12 gene	1810055E12Rik		2.11	SM2376	f		6	1.522164293
mPool7_V2MM_205775	2.063797	2.205692	TRUE	HP_286276	TGCTGTTG/	CTGAGTTT/	NM_030236	F-box only protein 34	Fbxo34		2.1	SM2302	g		8	1.656455229
092308m1_V2MM_174942	2.063045	3.004939	TRUE	HP_395427	TGCTGTTG/	CTGGAAAT	XM_287341	Mus musculus hypothetical gene supported by AK080995 (LOC329843), mR.			2.5	SM2077	a		7	1.522164293
092308m1_V2MM_184975	2.062451	2.205692	TRUE	HP_405447	TGCTGTTG/	CTGCTTGA(	XM_289290	Mus musculus LOC332940 (LOC332940), mR.			2.5	SM2095	a		4	1.656455229
mPool7_V2MM_78316	2.061128	3.004939	TRUE	HP_300008	TGCTGTTG/	GACCAGAT	NM_011192	proteaseome (prosome, macropain) 28 subunit, 3	Psme3		2.8	SM2232	a		10	1.522164293
mPool2_V2MM_104747	2.060998	2.205692	TRUE	HP_325879	TGCTGTTG/	GAGCTTAT(	AK173248	zinc finger, CCHC domain containing 2	Zcchc2		2.6	SM2149	c		4	1.656455229
mPool2_V2MM_144169	2.058879	2.205692	TRUE	HP_364880	TGCTGTTG/	CATTTGTGT	XM_159785	Mus musculus LOC207783 (LOC207783), mRNA.			2.1	SM2340	h		5	1.656455229
092308m1_V2MM_135868	2.057805	2.205692	TRUE	HP_356589	TGCTGTTG/	CATGACAC	XM_151129	Mus musculus LOC212730 (LOC212730), mR.			2.4	SM2031	d		11	1.656455229
092308m1_V2MM_141175	2.055914	2.205692	TRUE	HP_361888	TGCTGTTG/	CGGAAACA	XM_156493	Mus musculus LOC239875 (LOC239875), mR.		NA				NA		1.656455229
mPool2_V2MM_16625	2.05208	3.004939	TRUE	HP_239919	TGCTGTTG/	GACAGAGA	NM_134129	PRP19/PSO4 homolog (S. cerevisiae)	Prp19		2.11	SM2384	d		11	1.522164293
mPool2_V2MM_18303	2.051061	2.205692	TRUE	HP_241553	TGCTGTTG/	GCCTCTAC/	NM_011607	tenascin C	Tnc		2.7	SM2166	c		5	1.656455229
mPool2_V2MM_7219	2.048636	3.004939	TRUE	HP_230733	TGCTGTTG/	GAGCTACA(	NM_007798	cathepsin B	Ctsb		2.11	SM2383	a		7	1.522164293
092308m1_V2MM_160569	2.047524	3.004939	TRUE	HP_381148	TGCTGTTG/	GTTTCTGA/	XM_283567	Mus musculus similar to hypothetical protein 7 - rat (LOC329062), mR.			2.6	SM2118	h		6	1.522164293
092308m3_V2MM_41756	2.046211	60.13437	TRUE	HP_264381	TGCTGTTG/	CAGATATT	NM_009451	tubulin, beta 4	Tubb4		2.12	SM2416	e		11	0.220877236
mPool7_V2MM_86068	2.045252	1.959154	TRUE	HP_307545	TGCTGTTG/	CTATCATG(	NM_146613	olfactory receptor 973	Olfr973		2.9	SM2267	g		7	1.707931489
092308m1_V2MM_130662	2.044505	3.004939	TRUE	HP_351441	TGCTGTTG/	CATCCTTTA	XM_146968	Mus musculus similar to hypothetical protein MGC18257 [Homo sapiens] (LOC214322),			2.5	SM2100	c		9	1.522164293
092308m1_V2MM_70007	2.042037	3.004939	TRUE	HP_291928	TGCTGTTG/	GTGCAATC/	NM_007380	abl-interactor 1	Abi1		2.4	SM2022	a		6	1.522164293
092308m1_V2MM_151908	2.041136	3.004939	TRUE	HP_372575	TGCTGTTG/	CGTTTATCT	XM_194993	olfactory receptor 171	Olfr171		2.4	SM2042	e		4	1.522164293
mPool2_V2MM_166901	2.039306	3.004939	TRUE	HP_387424	TGCTGTTG/	CTGATATTT	XM_285628	Mus musculus similar to zinc finger protein 93 homolog; zinc finger protein homologous			2.6	SM2128	a		6	1.522164293
mPool7_V2MM_179484	2.039014	1.959154	TRUE	HP_399958	TGCTGTTG/	CACTAATT(	XM_288341	Mus musculus LOC331656 (LOC331656), mRNA.			2.5	SM2076	b		2	1.707931489
mPool7_V2MM_179484	2.039014	1.959154	TRUE	HP_399958	TGCTGTTG/	CACTAATT(	XM_288341	Mus musculus LOC331656 (LOC331656), mR.			2.5	SM2076	b		2	1.707931489
mPool2_V2MM_6274	2.038377	2.205692	TRUE	HP_229812	TGCTGTTG/	CTCTTATAT	NM_019586	ubiquitin-conjugating enzyme E2, J1	Ube2j1		2.11	SM2381	c		2	1.656455229
mPool7_V2MM_151720	2.03783	3.004939	TRUE	HP_372390	TGCTGTTG/	CGGATTAA(	XM_489722	similar to Rpl17 protein			2.4	SM2035	b		4	1.522164293
mPool7_V2MM_151720	2.03783	3.004939	TRUE	HP_372390	TGCTGTTG/	CGGATTAA(	XM_489722	similar to Rpl17 protein			2.4	SM2035	b		4	1.522164293
092308m1_V2MM_136460	2.0376	3.004939	TRUE	HP_357180	TGCTGTTG/	CTCTGTATT	XM_151736	Mus musculus LOC212058 (LOC212058), mR.			2.5	SM2057	a		5	1.522164293
mPool2_V2MM_210700	2.035306	3.004939	TRUE	HP_428439	TGCTGTTG/	CAGACCAG	XM_141415	Mus musculus similar to Peptidyl-prolyl cis-trans isomerase A (PPIase) (Rotamase) (Cycl			2.1	SM2350	g		8	1.522164293
092308m1_V2MM_142526	2.034763	2.205692	TRUE	HP_363239	TGCTGTTG/	CTTAGGAA	XM_157697	Mus musculus LOC240481 (LOC240481), mR.			2.5	SM2056	c		9	1.656455229
092308m1_V2MM_142526	2.034763	2.205692	TRUE	HP_363239	TGCTGTTG/	CTTAGGAA	XM_157697	Mus musculus LOC240481 (LOC240481), mRNA.			2.5	SM2056	c		9	1.656455229
mPool2_V2MM_117317	2.034715	2.205692	TRUE	HP_338263	TGCTGTTG/	CCTATGTC/	XM_139321	Mus musculus similar to protease [Mus musculus] (LOC223346), mRNA.			2.7	SM2160	f		9	1.656455229
mPool2_V2MM_85468	2.034221	3.004939	TRUE	HP_306949	TGCTGTTG/	GCCATTGC	NM_146390	olfactory receptor 1323	Olfr1323		2.6	SM2143	e		8	1.522164293
mPool2_V2MM_147923	2.03199	2.205692	TRUE	HP_368634	TGCTGTTG/	CAATTTCCA	XM_163354	Mus musculus LOC215451 (LOC215451), mRNA.			2.5	SM2052	e		6	1.656455229
mPool2_V2MM_147923	2.03199	2.205692	TRUE	HP_368634	TGCTGTTG/	CAATTTCCA	XM_163354	Mus musculus LOC215451 (LOC215451), mR.			2.5	SM2052	e		6	1.656455229
mPool2_V2MM_9420	2.030441	3.004939	TRUE	HP_232881	TGCTGTTG/	CAGCTGTA	NM_019571	transmembrane 4 superfamily member 9	Tm4sf9		2.11	SM2380	f		3	1.522164293
092308m1_V2MM_66196	2.028476	3.004939	TRUE	HP_288217	TGCTGTTG/	CAACAAAT(	NM_133196	cleavage stimulation factor, 3 pre-R subunit 2	Cstf2		2.4	SM2029	e		4	1.522164293
mPool2_V2MM_39295	2.025544	3.004939	TRUE	HP_261985	TGCTGTTG/	GCTTCAAA(	NM_130451	solute carrier family 2 (facilitated glucose transporter), memb	Slc2a10	NA				NA		1.522164293
mPool2_V2MM_84154	2.02289	1.959154	TRUE	HP_305672	TGCTGTTG/	CTATACCTA	NM_026989	RIKEN cDNA 2610019N13 gene	2610019N13Rik		2.6	SM2133	f		1	1.707931489

mPool2_V2MM_5641	2.022473	2.205692	TRUE	HP_229189	TGCTGTTG/	CCTAGAACT	NM_007622	chromobox homolog 1 (Drosophila HP1 beta)	Cbx1	2.11	SM2382	d	6	1.656455229
mPool7_V2MM_177038	2.021263	3.004939	TRUE	HP_397518	TGCTGTTG/	CCTTAAATG	AK039009	hypothetical protein A230084N10		2.5	SM2085	e	9	1.522164293
mPool7_V2MM_177038	2.021263	3.004939	TRUE	HP_397518	TGCTGTTG/	CCTTAAATG	AK039009	hypothetical protein A230084N10		2.5	SM2085	e	9	1.522164293
mPool2_V2MM_204607	2.018482	2.205692	TRUE	HP_422501	TGCTGTTG/	GTAGAATT(	AK050925	RIKEN cDNA 4930513F16 gene	4930513F16Rik	2.1	SM2319	a	6	1.656455229
mPool7_V2MM_69056	2.01462	2.205692	TRUE	HP_290998	TGCTGTTG/	GATGTGTA(	NM_008041	formyl peptide receptor, related sequence 4	Fpr-rs4	2.8	SM2242	e	6	1.656455229
mPool7_V2MM_70275	2.013936	3.004939	TRUE	HP_292191	TGCTGTTG/	CAAATATA(	NM_030150	DNA segment, Chr 11, Lothar Hennighausen 2, expressed	D11Lgp2e	2.8	SM2245	g	7	1.522164293
092308m1_V2MM_143932	2.011254	3.004939	TRUE	HP_364644	TGCTGTTG/	CAGAGATA	XM_159469	Mus musculus LOC211711 (LOC211711), mRNA.		2.4	SM2033	d	1	1.522164293
092308m1_V2MM_143932	2.011254	3.004939	TRUE	HP_364644	TGCTGTTG/	CAGAGATA	XM_159469	Mus musculus LOC211711 (LOC211711), mR.		2.4	SM2033	d	1	1.522164293
mPool7_V2MM_67685	2.009994	2.205692	TRUE	HP_289666	TGCTGTTG/	CTATACAA(	NM_011241	RAN GTPase activating protein 1	Rangap1	2.8	SM2231	e	11	1.656455229
mPool2_V2MM_118382	2.009705	3.004939	TRUE	HP_339319	TGCTGTTG/	CTTGAGGA	XM_139821	similar to RIKEN cDNA 1700029I01		2.11	SM2368	b	6	1.522164293
mPool7_V2MM_174732	2.008312	2.205692	TRUE	HP_395217	TGCTGTTG/	GCAGTAAA	AK034327	hypothetical protein 9330178D15		2.9	SM2277	f	7	1.656455229
mPool7_V2MM_174732	2.008312	2.205692	TRUE	HP_395217	TGCTGTTG/	GCAGTAAA	AK034327	hypothetical protein 9330178D15		2.9	SM2277	f	7	1.656455229
mPool7_V2MM_181893	2.007928	2.205692	TRUE	HP_402367	TGCTGTTG/	GTTTATTGG	XM_288752	Mus musculus LOC332202 (LOC332202), mRNA.		2.5	SM2089	c	5	1.656455229
mPool7_V2MM_181893	2.007928	2.205692	TRUE	HP_402367	TGCTGTTG/	GTTTATTGG	XM_288752	Mus musculus LOC332202 (LOC332202), mR.		2.5	SM2089	c	5	1.656455229
092308m1_V2MM_91659	2.007065	3.004939	TRUE	HP_313028	TGCTGTTG/	CGGAAGTC	NM_178399	RIKEN cD 3110035E14 gene	3110035E14Rik	2.6	SM2109	c	9	1.522164293
092308m1_V2MM_170685	2.00697	3.004939	TRUE	HP_391175	TGCTGTTG/	GGTTTATC/	XM_286470	Mus musculus hypothetical gene supported by AK048337 (LOC328164), mR.		2.5	SM2095	g	5	1.522164293
092308m1_V2MM_175033	2.005484	3.004939	TRUE	HP_395518	TGCTGTTG/	GATAGCCA.	AK029093	RIKEN cDNA E030045D18 gene	E030045D18Rik	2.5	SM2080	c	7	1.522164293
092308m1_V2MM_175033	2.005484	3.004939	TRUE	HP_395518	TGCTGTTG/	GATAGCCA.	AK029093	RIKEN cD E030045D18 gene	E030045D18Rik	2.5	SM2080	c	7	1.522164293
mPool2_V2MM_108684	2.003483	2.205692	TRUE	HP_329751	TGCTGTTG/	CTGAGTTCT	XM_133185	similar to hypothetical protein FLJ12895		2.7	SM2159	h	3	1.656455229
mPool7_V2MM_93943	2.003002	3.004939	TRUE	HP_315241	TGCTGTTG/	CTTGCCCA(	NM_182840	elastin microfibril interfacer 3	Emilin3	2.8	SM2249	e	9	1.522164293
092308m1_V2MM_165554	2.000053	3.004939	TRUE	HP_386089	TGCTGTTG/	CTGGAAGT.	XM_487540	LOC435573		2.6	SM2120	c	10	1.522164293
mPool2_V2MM_9341	1.998236	1.959154	TRUE	HP_232802	TGCTGTTG/	CAGATTCC(	NM_010903	nuclear factor, erythroid derived 2, like 3	Nfe2l3	2.11	SM2386	b	9	1.707931489
092308m1_V2MM_137497	1.997	3.004939	TRUE	HP_358217	TGCTGTTG/	GAAATTAT(	XM_153221	RIKEN cDNA 1110038H03 gene	1110038H03Rik	2.4	SM2042	h	10	1.522164293
092308m1_V2MM_137497	1.997	3.004939	TRUE	HP_358217	TGCTGTTG/	GAAATTAT(	XM_153221	RIKEN cD 1110038H03 gene	1110038H03Rik	2.4	SM2042	h	10	1.522164293
mPool2_V2MM_1383	1.995442	3.004939	TRUE	HP_225027	TGCTGTTG/	CCATAGACT	NM_018748	golgi autoantigen, golgin subfamily a, 4	Golga4	2.11	SM2386	f	11	1.522164293
mPool2_V2MM_163514	1.993409	3.004939	TRUE	HP_384069	TGCTGTTG/	CTGAATAT(	XM_284801	RIKEN cDNA 1700029B21 gene	1700029B21Rik	2.6	SM2127	a	8	1.522164293
mPool7_V2MM_204599	1.992271	3.004939	TRUE	HP_422493	TGCTGTTG/	CGCCAAGC.	XM_486831	similar to RIKEN cDNA 4921509A18 gene		2.1	SM2314	a	9	1.522164293
092308m1_V2MM_135381	1.991633	2.205692	TRUE	HP_356103	TGCTGTTG/	CTGTAATCT	XM_150596	Mus musculus LOC240784 (LOC240784), mR.		2.5	SM2058	e	12	1.656455229
mPool2_V2MM_144938	1.991622	3.004939	TRUE	HP_365649	TGCTGTTG/	CAATTGAC/	XM_160439	Mus musculus LOC242208 (LOC242208), mR.		2.4	SM2045	a	10	1.522164293
mPool2_V2MM_144938	1.991622	3.004939	TRUE	HP_365649	TGCTGTTG/	CAATTGAC/	XM_160439	Mus musculus LOC242208 (LOC242208), mRNA.		2.4	SM2045	a	10	1.522164293
092308m1_V2MM_144938	1.990391	3.004939	TRUE	HP_365649	TGCTGTTG/	CAATTGAC/	XM_160439	Mus musculus LOC242208 (LOC242208), mRNA.		2.4	SM2045	a	10	1.522164293
092308m1_V2MM_144938	1.990391	3.004939	TRUE	HP_365649	TGCTGTTG/	CAATTGAC/	XM_160439	Mus musculus LOC242208 (LOC242208), mR.		2.4	SM2045	a	10	1.522164293
mPool2_V2MM_116119	1.986981	2.205692	TRUE	HP_337074	TGCTGTTG/	CGTTTGAT(	XM_138761	Mus musculus similar to NADH-UBIQUINONE OXIDOREDUCTASE CHAIN 1 (LOC238703),		2.6	SM2149	f	4	1.656455229
092308m1_V2MM_75728	1.98636	2.205692	TRUE	HP_297500	TGCTGTTG/	CCCTTATAA	NM_146700	olfactory receptor 1453	Olfr1453	2.4	SM2020	c	8	1.656455229
mPool2_V2MM_104265	1.985448	3.004939	TRUE	HP_325399	TGCTGTTG/	CATGCATC(	XM_129647	glutamyl-prolyl-tRNA synthetase	Eprs	2.6	SM2149	f	9	1.522164293
092308m1_V2MM_136201	1.985416	2.205692	TRUE	HP_356921	TGCTGTTG/	CGGGAATA	NM_207031	prostate cancer associated protein 5	Pcap5	2.4	SM2035	d	10	1.656455229
mPool2_V2MM_134989	1.985106	1.959154	TRUE	HP_355713	TGCTGTTG/	GAAATCAA(	AK015733	RIKEN cDNA 4930509E16 gene	4930509E16Rik	2.11	SM2363	g	12	1.707931489
092308m1_V2MM_135825	1.983802	3.004939	TRUE	HP_356547	TGCTGTTG/	GAGTTACA(	XM_151088	Mus musculus LOC212291 (LOC212291), mR.		2.4	SM2047	h	12	1.522164293
mPool2_V2MM_208932	1.983489	2.205692	TRUE	HP_426712	TGCTGTTG/	CCCAATTTC	XM_163354	Mus musculus LOC215451 (LOC215451), mRNA.		2.1	SM2345	d	7	1.656455229
092308m1_V2MM_176881	1.979243	3.004939	TRUE	HP_397361	TGCTGTTG/	GTGATATT(	AK085344	RIKEN cD D630014A15 gene	D630014A15Rik	2.5	SM2081	e	7	1.522164293
mPool2_V2MM_92572	1.978452	3.004939	TRUE	HP_313919	TGCTGTTG/	CCCAATAC/	NM_178735	RIKEN cDNA A730069N07 gene	A730069N07Rik	2.6	SM2143	f	5	1.522164293
092308m1_V2MM_91071	1.978003	3.004939	TRUE	HP_312487	TGCTGTTG/	CTTCAAGA(	NM_177915	immunoglobulin superfamily, member 1	Igsf1	2.6	SM2113	e	12	1.522164293
092308m1_V2MM_176857	1.977693	3.004939	TRUE	HP_397337	TGCTGTTG/	GTACCAAT(	XM_287757	non-coding transcript 1	Nctc1	2.5	SM2063	h	8	1.522164293
mPool7_V2MM_108179	1.975357	3.004939	TRUE	HP_329251	TGCTGTTG/	CCAGTTAT(	BC058338	RIKEN cDNA 2210404G23 gene	2210404G23Rik	2.1	SM2315	c	8	1.522164293
mPool7_V2MM_78418	1.973083	2.205692	TRUE	HP_300104	TGCTGTTG/	GACTATCA(	NM_022332	Suppression of tumorigenicity 7	St7	2.8	SM2234	e	12	1.656455229
mPool7_V2MM_201299	1.970236	2.205692	TRUE	HP_419328	TGCTGTTG/	CAATCTATG	AK043696	RIKEN cDNA A830021F12 gene	A830021F12Rik	2.9	SM2284	d	7	1.656455229
mPool2_V2MM_139604	1.969442	3.004939	TRUE	HP_360324	TGCTGTTG/	CAGAGAGG	XM_155248	Mus musculus LOC223326 (LOC223326), mRNA.		2.1	SM2347	e	1	1.522164293
092308m1_V2MM_138094	1.967412	3.004939	TRUE	HP_358814	TGCTGTTG/	GACAGTAT(	XM_153883	Mus musculus LOC238525 (LOC238525), mR.		2.5	SM2054	a	9	1.522164293
092308m1_V2MM_138094	1.967412	3.004939	TRUE	HP_358814	TGCTGTTG/	GACAGTAT(	XM_153883	Mus musculus LOC238525 (LOC238525), mRNA.		2.5	SM2054	a	9	1.522164293
092308m3_V2MM_255457	1.966382	75.83547	TRUE	HP_318916	TGCTGTTG/	GGCTTACA(	NM_138596	D segment, Chr 13, Wayne State University 50, expressed	D13Wsu50e	2.13	SM2477	h	9	0.120127608
mPool7_V2MM_71610	1.96447	2.205692	TRUE	HP_293486	TGCTGTTG/	CGTAATCT(	XM_142620	similar to hypothetical protein FLJ90396		2.8	SM2232	b	12	1.656455229
mPool2_V2MM_170124	1.964206	2.205692	TRUE	HP_390619	TGCTGTTG/	CAGGTAAA	AK029830	trans-acting transcription factor 6	Sp6	2.6	SM2128	d	6	1.656455229
mPool2_V2MM_109155	1.963502	3.004939	TRUE	HP_330220	TGCTGTTG/	GAGATCTTT	BC057029	phosphodiesterase 2A, cGMP-stimulated	Pde2a	2.6	SM2149	c	7	1.522164293
092308m1_V2MM_170459	1.963127	3.004939	TRUE	HP_390952	TGCTGTTG/	GGCTTTAA(	XM_283177	Mus musculus similar to hypothetical protein 3 - rat (LOC328328), mRNA.		2.5	SM2077	e	6	1.522164293
092308m1_V2MM_170459	1.963127	3.004939	TRUE	HP_390952	TGCTGTTG/	GGCTTTAA(	XM_283177	Mus musculus similar to hypothetical protein 3 - rat (LOC328328), mR.		2.5	SM2077	e	6	1.522164293
mPool7_V2MM_87336	1.962984	2.205692	TRUE	HP_308794	TGCTGTTG/	GCATGTATT	NM_175141	RIKEN cDNA 1810030O07 gene	1810030O07Rik	2.9	SM2269	f	11	1.656455229
mPool2_V2MM_123668	1.962399	3.004939	TRUE	HP_344524	TGCTGTTG/	GGTATATG.	XM_143088	Mus musculus similar to small nuclear ribonucleic protein (LOC229081), mRNA.		2.11	SM2371	g	11	1.522164293
092308m1_V2MM_71710	1.961597	2.205692	TRUE	HP_293583	TGCTGTTG/	CTACCTTGA	NM_025695	SMC6 structural maintence of chromosomes 6-like 1 (yeast)	Smc6l1	2.3	SM2016	e	2	1.656455229
mPool2_V2MM_100109	1.96141	3.004939	TRUE	HP_321302	TGCTGTTG/	CATTTCTAT	NM_009546	tripartite motif protein 25	Trim25	2.7	SM2158	g	10	1.522164293
092308m1_V2MM_136270	1.960709	2.205692	TRUE	HP_356990	TGCTGTTG/	GGAACAAG	XM_151594	Mus musculus LOC213763 (LOC213763), mR.		2.4	SM2039	h	8	1.656455229
092308m1_V2MM_136270	1.960709	2.205692	TRUE	HP_356990	TGCTGTTG/	GGAACAAG	XM_151594	Mus musculus LOC213763 (LOC213763), mRNA.		2.4	SM2039	h	8	1.656455229
092308m1_V2MM_83740	1.960038	1.959154	TRUE	HP_305266	TGCTGTTG/	CTGAGTGT(	NM_024288	RIKEN cD 1110007A06 gene	1110007A06Rik	2.6	SM2113	c	8	1.707931489

092308m1_V2MM_170903	1.95963	3.004939	TRUE	HP_391393	TGCTGTTG	CTTCCTAAA	AK046068	RIKEN cD A330033J07 gene	A330033J07Rik	2.5	SM2091	b	9	1.522164293
092308m1_V2MM_132136	1.956419	2.205692	TRUE	HP_352890	TGCTGTTG	CTCTCCCG	XM_148108	RIKEN cD 4930451G09 gene	4930451G09Rik	2.6	SM2103	e	8	1.656455229
mPool7_V2MM_203240	1.956169	3.004939	TRUE	HP_421202	TGCTGTTG	GGAAACGT	XM_136539	Mus musculus similar to HMG-box containing protein 1 - rat (LOC226885), mRNA.		2.1	SM2313	a	7	1.522164293
mPool2_V2MM_139675	1.953193	2.205692	TRUE	HP_360395	TGCTGTTG	CTCTATTCA	XM_155296	Mus musculus LOC211057 (LOC211057), mR.		2.1	SM2336	c	3	1.656455229
mPool2_V2MM_139675	1.953193	2.205692	TRUE	HP_360395	TGCTGTTG	CTCTATTCA	XM_155296	Mus musculus LOC211057 (LOC211057), mRNA.		2.1	SM2336	c	3	1.656455229
mPool2_V2MM_168754	1.952362	2.205692	TRUE	HP_389256	TGCTGTTG	CTTAGCATT	XM_286058	Mus musculus similar to putative pheromone receptor [Rattus norvegicus] (LOC333227)		2.6	SM2131	c	10	1.656455229
092308m1_V2MM_147048	1.952156	2.205692	TRUE	HP_367759	TGCTGTTG	CACAAAGA	XM_162569	Mus musculus LOC243509 (LOC243509), mRNA.		2.4	SM2041	e	9	1.656455229
092308m1_V2MM_147048	1.952156	2.205692	TRUE	HP_367759	TGCTGTTG	CACAAAGA	XM_162569	Mus musculus LOC243509 (LOC243509), mR.		2.4	SM2041	e	9	1.656455229
mPool7_V2MM_73386	1.951822	3.004939	TRUE	HP_295223	TGCTGTTG	GGAAATGT	NM_025435	RIKEN cDNA 1500006O09 gene	1500006O09Rik	2.8	SM2234	c	12	1.522164293
mPool7_V2MM_182325	1.950208	3.004939	TRUE	HP_402799	TGCTGTTG	CAGTCAAT	XM_288825	Mus musculus LOC332298 (LOC332298), mR.		2.5	SM2062	c	5	1.522164293
mPool7_V2MM_182325	1.950208	3.004939	TRUE	HP_402799	TGCTGTTG	CAGTCAAT	XM_288825	Mus musculus LOC332298 (LOC332298), mRNA.		2.5	SM2062	c	5	1.522164293
mPool7_V2MM_202377	1.946956	0	TRUE	HP_92884	TGCTGTTG	CTAAGAAT	XM_128090	RIKEN cDNA D330037H05 gene	D330037H05Rik	2.9	SM2296	d	11	#NUM!
092308m3_V2MM_47630	1.945047	75.83547	TRUE	HP_270106	TGCTGTTG	CTGTGGCT	NM_138592	ubiquitin specific protease 39	Usp39	2.12	SM2416	b	5	0.120127608
092308m1_V2MM_140408	1.944185	2.205692	TRUE	HP_361128	TGCTGTTG	CAGTTTAA	XM_155942	Mus musculus similar to Voltage-dependent anion-selective channel protein 3 (VDAC-3)		2.4	SM2038	h	11	1.656455229
mPool2_V2MM_146584	1.943609	3.004939	TRUE	HP_367295	TGCTGTTG	CATAATTG	XM_162162	Mus musculus LOC243184 (LOC243184), mR.		2.5	SM2058	a	9	1.522164293
mPool2_V2MM_146584	1.943609	3.004939	TRUE	HP_367295	TGCTGTTG	CATAATTG	XM_162162	Mus musculus LOC243184 (LOC243184), mRNA.		2.5	SM2058	a	9	1.522164293
mPool7_V2MM_105217	1.942843	3.004939	TRUE	HP_326337	TGCTGTTG	CAAGTTAT	XM_130324	RIKEN cDNA 2810410A08 gene	2810410A08Rik	2.1	SM2306	h	4	1.522164293
mPool7_V2MM_107148	1.94244	2.205692	TRUE	HP_328238	TGCTGTTG	CCCAGTGG	NM_021604	agrin	Agrn	2.1	SM2301	e	5	1.656455229
mPool2_V2MM_121702	1.940567	2.205692	TRUE	HP_342580	TGCTGTTG	GCAAGAAT	XM_141796	Mus musculus similar to RIKEN cDNA 4933402E13 [Mus musculus] (LOC236842), mRNA.		2.5	SM2099	d	3	1.656455229
mPool2_V2MM_121702	1.940567	2.205692	TRUE	HP_342580	TGCTGTTG	GCAAGAAT	XM_141796	Mus musculus similar to RIKEN cD 4933402E13 [Mus musculus] (LOC236842), mR.		2.5	SM2099	d	3	1.656455229
mPool7_V2MM_197536	1.940227	3.004939	TRUE	HP_415606	TGCTGTTG	GGTGTAAT	NM_011264	REV3-like, catalytic subunit of DNA polymerase zeta RAD54 lik	Rev3l	2.9	SM2253	a	4	1.522164293
092308m1_V2MM_154123	1.939896	3.004939	TRUE	HP_374764	TGCTGTTG	GCTGTTAT	NM_201354	gene model 672, (NCBI)	Gm672	2.5	SM2091	e	12	1.522164293
mPool2_V2MM_88570	1.939516	3.004939	TRUE	HP_310007	TGCTGTTG	CACTTAGT	NM_175436	zinc finger protein 526	Zfp526	2.6	SM2147	e	3	1.522164293
mPool2_V2MM_106861	1.938486	3.004939	TRUE	HP_327954	TGCTGTTG	GTTTATATA	AK053970	RIKEN cDNA 4930538K18 gene	4930538K18Rik	2.6	SM2149	a	11	1.522164293
092308m3_V2MM_113015	1.938188	75.83547	TRUE	NA	NA	NA	NA	NA	NA	NA	NA	NA	NA	0.120127608
mPool7_V2MM_84065	1.937373	2.205692	TRUE	HP_305584	TGCTGTTG	GTCATCTA	NM_026808	RIKEN cDNA 1110028A07 gene	1110028A07Rik	2.8	SM2250	e	3	1.656455229
mPool7_V2MM_88813	1.936547	3.004939	TRUE	HP_310249	TGCTGTTG	GACCTTTC	NM_175499	SLIT and NTRK-like family, member 6	Slitrk6	2.9	SM2252	e	11	1.522164293
mPool7_V2MM_88813	1.936547	3.004939	TRUE	HP_310249	TGCTGTTG	GACCTTTC	NM_175499	SLIT and NTRK-like family, member 6	Slitrk6	2.9	SM2252	e	11	1.522164293
092308m1_V2MM_112685	1.936069	2.205692	TRUE	HP_298472	TGCTGTTG	CTATTAGG	XM_486245	similar to Rpl7a protein		2.4	SM2028	b	1	1.656455229
092308m1_V2MM_112685	1.936069	2.205692	TRUE	HP_298472	TGCTGTTG	CTATTAGG	XM_486245	similar to Rpl7a protein		2.4	SM2028	b	1	1.656455229
092308m1_V2MM_64665	1.934134	3.004939	TRUE	HP_286715	TGCTGTTG	CTTATTACA	NM_009863	cell division cycle 7 (S. cerevisiae)	Cdc7	2.4	SM2022	e	9	1.522164293
mPool2_V2MM_215307	1.931471	1.959154	TRUE	HP_432863	TGCTGTTG	GATGTGAA	XM_147068	Mus musculus similar to glyceraldehyde-3-phosphate dehydrogenase [Mus musculus] (L		2.11	SM2358	e	12	1.707931489
092308m1_V2MM_172849	1.930505	3.004939	TRUE	HP_393335	TGCTGTTG	CCATATATA	AK085159	RIKEN cDNA 4933403F05 gene	4933403F05Rik	2.5	SM2069	f	8	1.522164293
092308m1_V2MM_172849	1.930505	3.004939	TRUE	HP_393335	TGCTGTTG	CCATATATA	AK085159	RIKEN cD 4933403F05 gene	4933403F05Rik	2.5	SM2069	f	8	1.522164293
mPool2_V2MM_109617	1.929899	2.205692	TRUE	HP_330671	TGCTGTTG	CTCACTCAT	XM_486079	RIKEN cDNA 4930453L07 gene	4930453L07Rik	2.7	SM2153	b	3	1.656455229
092308m3_V2MM_33008	1.929111	75.83547	TRUE	HP_255872	TGCTGTTG	CAATGTGT	NM_008542	MAD homolog 6 (Drosophila)	Smad6	2.12	SM2429	a	10	0.120127608
mPool2_V2MM_163177	1.928686	2.205692	TRUE	HP_383733	TGCTGTTG	GGCTTTAA	XM_284694	Mus musculus hypothetical gene supported by AK039538 (LOC331396), mRNA.		2.6	SM2131	h	8	1.656455229
092308m1_V2MM_136154	1.928177	2.205692	TRUE	HP_356874	TGCTGTTG	GTTTATAA	XM_151438	Mus musculus LOC241149 (LOC241149), mR.		2.5	SM2059	g	3	1.656455229
mPool2_V2MM_212123	1.926596	3.004939	TRUE	HP_429800	TGCTGTTG	GGAGAGAA	XM_145549	similar to hypothetical protein FLJ38281		2.11	SM2364	e	10	1.522164293
mPool2_V2MM_116075	1.923422	3.004939	TRUE	HP_337030	TGCTGTTG	GTGCTTATT	XM_138743	Mus musculus similar to Gag [Ovis aries] (LOC218358), mRNA.		2.7	SM2154	e	2	1.522164293
mPool2_V2MM_92325	1.923364	3.004939	TRUE	HP_313676	TGCTGTTG	CAGCGATC	NM_178689	RIKEN cDNA 6430537H07 gene	6430537H07Rik	2.6	SM2137	d	6	1.522164293
mPool2_V2MM_108460	1.922955	1.959154	TRUE	HP_329530	TGCTGTTG	CTTATTTGG	NM_019697	potassium voltage-gated channel, Shal-related family, membe	Kcnd2	2.1	SM2309	d	9	1.707931489
mPool2_V2MM_144897	1.9224	3.004939	TRUE	HP_365608	TGCTGTTG	CAACCTTTC	XM_160409	Mus musculus LOC242190 (LOC242190), mR.		2.1	SM2334	c	11	1.522164293
mPool2_V2MM_144897	1.9224	3.004939	TRUE	HP_365608	TGCTGTTG	CAACCTTTC	XM_160409	Mus musculus LOC242190 (LOC242190), mRNA.		2.1	SM2334	c	11	1.522164293
mPool2_V2MM_215055	1.921813	2.205692	TRUE	HP_432623	TGCTGTTG	CCTTCGTTC	XM_141782	Mus musculus similar to H3 histone, family 3B [Mus musculus] (LOC245434), mRNA.		2.11	SM2355	e	1	1.656455229
092308m1_V2MM_185834	1.921408	3.004939	TRUE	HP_406306	TGCTGTTG	CCTGCATTC	XM_289437	Mus musculus LOC333103 (LOC333103), mR.		2.5	SM2094	g	2	1.522164293
092308m1_V2MM_139690	1.921377	3.004939	TRUE	HP_360410	TGCTGTTG	CAGACAAT	XM_155311	Mus musculus LOC211071 (LOC211071), mR.		2.5	SM2056	f	9	1.522164293
092308m1_V2MM_139690	1.921377	3.004939	TRUE	HP_360410	TGCTGTTG	CAGACAAT	XM_155311	Mus musculus LOC211071 (LOC211071), mRNA.		2.5	SM2056	f	9	1.522164293
mPool2_V2MM_126633	1.919759	3.004939	TRUE	HP_347475	TGCTGTTG	GCTGCATT	XM_144678	Mus musculus similar to ribosomal protein L19 [Rattus norvegicus] (LOC213268), mRNA		2.11	SM2360	g	12	1.522164293
mPool2_V2MM_212264	1.918986	1.959154	TRUE	HP_429934	TGCTGTTG	CTCTTGGTC	XM_143544	Mus musculus similar to CL2BB protein - rat (LOC215282), mRNA.		2.11	SM2352	d	1	1.707931489
mPool7_V2MM_79053	1.918247	3.004939	TRUE	HP_300728	TGCTGTTG	GAGTGGTT	NM_025453	RIKEN cDNA 1810018L02 gene	1810018L02Rik	2.8	SM2236	e	6	1.522164293
092308m1_V2MM_177264	1.91691	3.004939	TRUE	HP_397744	TGCTGTTG	GCATTTAG	AK045718	RIKEN cD C030044C12 gene	C030044C12Rik	2.5	SM2078	h	8	1.522164293
mPool2_V2MM_986	1.915567	2.205692	TRUE	HP_224642	TGCTGTTG	CAGTAAAT	XM_484397	diacylglycerol kinase, eta	Dgkh	2.11	SM2379	g	6	1.656455229
mPool7_V2MM_81233	1.911495	2.205692	TRUE	HP_302841	TGCTGTTG	GGTGTAGT	NM_172815	RIKEN cDNA 2610028F08 gene	2610028F08Rik	2.8	SM2248	c	4	1.656455229
092308m3_V2MM_96391	1.911202	75.83547	TRUE	HP_317644	TGCTGTTG	GAAATGTT	AK015302	RIKEN cD 4933432K03 gene	4933432K03Rik	2.9	SM2251	d	3	0.120127608
092308m3_V2MM_96391	1.911202	75.83547	TRUE	HP_317644	TGCTGTTG	GAAATGTT	AK015302	RIKEN cDNA 4933432K03 gene	4933432K03Rik	2.9	SM2251	d	3	0.120127608
mPool7_V2MM_180607	1.910923	3.004939	TRUE	HP_401081	TGCTGTTG	CTATGAAT	XM_358439	gene model 1573, (NCBI)	Gm1573	2.5	SM2065	h	7	1.522164293
mPool7_V2MM_180607	1.910923	3.004939	TRUE	HP_401081	TGCTGTTG	CTATGAAT	XM_358439	gene model 1573, (NCBI)	Gm1573	2.5	SM2065	h	7	1.522164293
mPool2_V2MM_105912	1.910609	3.004939	TRUE	HP_327019	TGCTGTTG	CAGAGACC	AK015427	RIKEN cDNA 4930449A18 gene	4930449A18Rik	2.7	SM2158	e	11	1.522164293
mPool6_V2MM_69203	1.910461	75.83547	TRUE	HP_291141	TGCTGTTG	GCATCTGG	NM_010319	guanine nucleotide binding protein (G protein), gamma 7 sub	Gng7	2.7	SM2175	g	5	0.120127608
mPool7_V2MM_204746	1.909558	3.004939	TRUE	HP_422636	TGCTGTTG	CTGTCAAA	XM_129787	similar to ALY		2.1	SM2303	e	11	1.522164293
092308m1_V2MM_165290	1.908716	2.205692	TRUE	HP_385827	TGCTGTTG	CTGGTGAT	XM_484618	similar to hypothetical protein FLJ38755		2.6	SM2115	h	6	1.656455229

mPool6_V2MM_35599	1.907829	75.83547	TRUE	HP_258392	TGCTGTTG/	CTAGGGAA	NM_152804	polo-like kinase 2 (Drosophila)	Plk2	2.7	SM2183	h	9	0.120127608
092308m1_V2MM_136543	1.905996	2.205692	TRUE	HP_357263	TGCTGTTG/	GTTATCTTG	XM_151819	Mus musculus LOC213261 (LOC213261), mRNA.		2.4	SM2034	c	9	1.656455229
092308m1_V2MM_136543	1.905996	2.205692	TRUE	HP_357263	TGCTGTTG/	GTTATCTTG	XM_151819	Mus musculus LOC213261 (LOC213261), mR.		2.4	SM2034	c	9	1.656455229
092308m1_V2MM_142682	1.904666	3.004939	TRUE	HP_363395	TGCTGTTG/	CTCTCTGTC	XM_157813	Mus musculus LOC240599 (LOC240599), mR.		2.4	SM2049	c	10	1.522164293
mPool7_V2MM_112685	1.901594	2.205692	TRUE	HP_298472	TGCTGTTG/	CTATTAGG/	XM_486245	similar to Rpl7a protein		2.4	SM2028	b	1	1.656455229
mPool7_V2MM_112685	1.901594	2.205692	TRUE	HP_298472	TGCTGTTG/	CTATTAGG/	XM_486245	similar to Rpl7a protein		2.4	SM2028	b	1	1.656455229
092308m1_V2MM_161230	1.901168	2.205692	TRUE	HP_381801	TGCTGTTG/	CCAGGTCT/	XM_283818	cadherin-like 26	Cdh26	2.6	SM2115	h	9	1.656455229
mPool7_V2MM_81478	1.898915	3.004939	TRUE	HP_303083	TGCTGTTG/	GTCAGTTT(	NM_172815	RIKEN cDNA 2610028F08 gene	2610028F08Rik	2.8	SM2229	g	1	1.522164293
mPool2_V2MM_210988	1.898246	3.004939	TRUE	HP_377864	TGCTGTTG/	CCTATGTAT	XM_205092	Mus musculus similar to Zinc finger protein 180 (HHZ168) (LOC277541), mRNA.		2.11	SM2364	e	9	1.522164293
mPool2_V2MM_106763	1.896387	3.004939	TRUE	HP_327857	TGCTGTTG/	CTTGCTTAC	BC037594	cDNA sequence BC037594	BC037594	2.7	SM2152	h	12	1.522164293
mPool2_V2MM_87110	1.895813	3.004939	TRUE	HP_308570	TGCTGTTG/	CAGATAATT	NM_175024	RIKEN cDNA G630049C14 gene	G630049C14Rik	2.6	SM2149	c	8	1.522164293
092308m1_V2MM_123096	1.895295	3.004939	TRUE	HP_343952	TGCTGTTG/	CACAGGTG.	XM_142669	similar to 60 kDa heat shock protein, mitochondrial precursor (Hsp60) (60 kDa chaperor		2.6	SM2102	b	6	1.522164293
092308m1_V2MM_123096	1.895295	3.004939	TRUE	HP_343952	TGCTGTTG/	CACAGGTG.	XM_142669	similar to 60 kDa heat shock protein, mitochondrial precursor (Hsp60) (60 kDa chaperor		2.6	SM2102	b	6	1.522164293
mPool2_V2MM_100653	1.895155	2.205692	TRUE	HP_321835	TGCTGTTG/	CAATTGGA(	AK032388	serologically defined colon cancer antigen 1	Sdccag1	2.7	SM2154	a	2	1.656455229
mPool7_V2MM_183702	1.894866	2.205692	TRUE	HP_404176	TGCTGTTG/	CATCAAACT	AK018908	RIKEN cDNA 1700085A12 gene	1700085A12Rik	2.9	SM2275	f	7	1.656455229
mPool2_V2MM_12204	1.893768	2.205692	TRUE	HP_235606	TGCTGTTG/	GTGACAAA	NM_134191	vomeronasal 1 receptor, E2	V1re2	2.11	SM2377	f	10	1.656455229
mPool2_V2MM_110673	1.893199	3.004939	TRUE	HP_331703	TGCTGTTG/	GAGATTGT(	BC051163	mannose phosphate isomerase 1	Mpi1	2.7	SM2162	b	11	1.522164293
mPool7_V2MM_101164	1.893125	3.004939	TRUE	HP_322338	TGCTGTTG/	GACAGACA	AK046032	RIKEN cDNA 2900024O10 gene	2900024O10Rik	2.6	SM2150	b	10	1.522164293
mPool7_V2MM_143114	1.892393	3.004939	TRUE	HP_363827	TGCTGTTG/	GGATTACT	XM_158386	Mus musculus LOC241409 (LOC241409), mRNA.		2.4	SM2043	a	12	1.522164293
mPool7_V2MM_143114	1.892393	3.004939	TRUE	HP_363827	TGCTGTTG/	GGATTACT	XM_158386	Mus musculus LOC241409 (LOC241409), mR.		2.4	SM2043	a	12	1.522164293
mPool7_V2MM_97784	1.89095	3.004939	TRUE	HP_319023	TGCTGTTG/	CTATAGAA(	XM_111892	RIKEN cDNA 1700123J19 gene	1700123J19Rik	2.9	SM2256	d	2	1.522164293
mPool7_V2MM_97784	1.89095	3.004939	TRUE	HP_319023	TGCTGTTG/	CTATAGAA(	XM_111892	RIKEN cD 1700123J19 gene	1700123J19Rik	2.9	SM2256	d	2	1.522164293
mPool2_V2MM_35528	1.890658	2.205692	TRUE	HP_258321	TGCTGTTG/	CTAAGACA/	NM_027457	RIKEN cDNA 5730437N04 gene	5730437N04Rik	2.11	SM2389	a	6	1.656455229
mPool7_V2MM_200071	1.890457	3.004939	TRUE	HP_418108	TGCTGTTG/	GGACTTTA(	XM_286571	Mus musculus hypothetical gene supported by AK051599 (LOC328315), mRNA.		2.9	SM2289	d	5	1.522164293
mPool2_V2MM_141195	1.889642	1.959154	TRUE	HP_361908	TGCTGTTG/	GACAAATT/	XM_156514	Mus musculus LOC239894 (LOC239894), mRNA.		2.4	SM2042	d	4	1.707931489
mPool2_V2MM_141195	1.889642	1.959154	TRUE	HP_361908	TGCTGTTG/	GACAAATT/	XM_156514	Mus musculus LOC239894 (LOC239894), mR.		2.4	SM2042	d	4	1.707931489
092308m1_V2MM_153410	1.889635	3.004939	TRUE	HP_374065	TGCTGTTG/	CTGTCCGTT	XM_196098	Mus musculus LOC268660 (LOC268660), mR.		2.6	SM2121	e	12	1.522164293
mPool7_V2MM_151904	1.888824	3.004939	TRUE	HP_372571	TGCTGTTG/	GCGTTTAT(	XM_194993	olfactory receptor 171	Olfr171	2.1	SM2327	c	4	1.522164293
mPool7_V2MM_176524	1.885764	1.959154	TRUE	HP_397004	TGCTGTTG/	CTCCATTG(	XM_489175	hypothetical gene supported by AK049985		2.5	SM2089	c	4	1.707931489
mPool7_V2MM_176524	1.885764	1.959154	TRUE	HP_397004	TGCTGTTG/	CTCCATTG(	XM_489175	hypothetical gene supported by AK049985		2.5	SM2089	c	4	1.707931489
mPool2_V2MM_87591	1.884702	3.004939	TRUE	HP_309043	TGCTGTTG/	GAACGTCT(	BC060730	ring finger protein 38	Rnf38	2.6	SM2144	b	3	1.522164293
mPool7_V2MM_176192	1.88464	2.205692	TRUE	HP_396674	TGCTGTTG/	CTTATTCCT	XM_287620	Mus musculus hypothetical gene supported by AK036508 (LOC330383), mRNA.		2.9	SM2292	b	9	1.656455229
mPool7_V2MM_174946	1.884374	2.205692	TRUE	HP_395431	TGCTGTTG/	CGCTGTAG(	XM_287341	Mus musculus hypothetical gene supported by AK080995 (LOC329843), mRNA.		2.9	SM2284	a	3	1.656455229
092308m1_V2MM_179537	1.884121	3.004939	TRUE	HP_400011	TGCTGTTG/	CAGTTTGG/	XM_288350	Mus musculus LOC331670 (LOC331670), mR.		2.5	SM2094	b	6	1.522164293
092308m1_V2MM_69539	1.882686	2.205692	TRUE	HP_291465	TGCTGTTG/	GCTATTTA(	NM_009923	cyclic nucleotide phosphodiesterase 1	Cnp1	2.4	SM2019	a	12	1.656455229
092308m3_V2MM_259618	1.881581	75.83547	TRUE	HP_318915	TGCTGTTG/	GCTTACAG(	NM_138596	D segment, Chr 13, Wayne State University 50, expressed	D13Wsu50e	2.13	SM2453	b	6	0.120127608
092308m1_V2MM_173441	1.881264	1.959154	TRUE	HP_393927	TGCTGTTG/	CAAACATA	AK035520	hypothetical protein 9530060I07		2.5	SM2081	c	2	1.707931489
092308m1_V2MM_173441	1.881264	1.959154	TRUE	HP_393927	TGCTGTTG/	CAAACATA	AK035520	hypothetical protein 9530060I07		2.5	SM2081	c	2	1.707931489
mPool7_V2MM_115972	1.880251	3.004939	TRUE	HP_336929	TGCTGTTG/	CTCCTTCAC	XM_138705	Mus musculus similar to RNA polymerase II transcriptional coactivator [Mus musculus] (		2.1	SM2301	h	1	1.522164293
mPool2_V2MM_3018	1.879415	3.004939	TRUE	HP_86735	TGCTGTTG/	CTGTTTGG/	NM_011448	SRY-box containing gene 9	Sox9	2.11	SM2376	g	3	1.522164293
092308m1_V2MM_153317	1.877659	2.205692	TRUE	HP_373974	TGCTGTTG/	GTCAGTAT(	XM_484109	RIKEN cD 4930417G10 gene	4930417G10Rik	2.6	SM2118	h	5	1.656455229
mPool7_V2MM_101389	1.876482	3.004939	TRUE	HP_322560	TGCTGTTG/	GTGTTTAC/	AK007938	RIKEN cDNA 1810062O18 gene	1810062O18Rik	2.1	SM2305	h	2	1.522164293
mPool2_V2MM_12514	1.874201	3.004939	TRUE	HP_235904	TGCTGTTG/	CAAGTTAA/	AF463504	metastasis associated 1	Mta1	2.11	SM2376	c	3	1.522164293
mPool7_V2MM_198072	1.87376	2.205692	TRUE	HP_416132	TGCTGTTG/	CTATCATTT	XM_287636	Mus musculus hypothetical gene supported by AK039232 (LOC330394), mRNA.		2.9	SM2279	d	2	1.656455229
092308m3_V2MM_91799	1.873063	59.99801	TRUE	HP_313168	TGCTGTTG/	CCTCTAATA	NM_178595	RIKEN cD 2210013M04 gene	2210013M04Rik	2.13	SM2479	f	5	0.221863149
092308m1_V2MM_150297	1.870964	1.959154	TRUE	HP_370990	TGCTGTTG/	GAATTATT(	BC023730	cell division cycle 27 homolog (S. cerevisiae)	Cdc27	2.4	SM2039	e	7	1.707931489
mPool2_V2MM_211266	1.870358	3.004939	TRUE	HP_340196	TGCTGTTG/	CTCTCAAT(	XM_486101	similar to hypothetical protein FLJ25801		2.1	SM2336	e	8	1.522164293
mPool7_V2MM_108856	1.868995	3.004939	TRUE	HP_329922	TGCTGTTG/	CTGAGATG	AK002462	RIKEN cDNA 0610010E21 gene	0610010E21Rik	2.1	SM2317	f	12	1.522164293
mPool2_V2MM_90839	1.868748	2.205692	TRUE	HP_312255	TGCTGTTG/	CTACAAAG	NM_177869	expressed sequence AI847670	AI847670	2.6	SM2145	g	8	1.656455229
mPool2_V2MM_141774	1.868049	2.205692	TRUE	HP_362487	TGCTGTTG/	CTTCAATA/	XM_156976	Mus musculus LOC210827 (LOC210827), mRNA.		2.4	SM2049	b	6	1.656455229
mPool2_V2MM_141774	1.868049	2.205692	TRUE	HP_362487	TGCTGTTG/	CTTCAATA/	XM_156976	Mus musculus LOC210827 (LOC210827), mR.		2.4	SM2049	b	6	1.656455229
092308m1_V2MM_92527	1.867673	3.004939	TRUE	HP_313875	TGCTGTTG/	CCAATTAA(	NM_178727	RIKEN cD D630039A03 gene	D630039A03Rik	2.6	SM2106	c	11	1.522164293
092308m1_V2MM_92527	1.867673	3.004939	TRUE	HP_313875	TGCTGTTG/	CCAATTAA(	NM_178727	RIKEN cDNA D630039A03 gene	D630039A03Rik	2.6	SM2106	c	11	1.522164293
mPool7_V2MM_79585	1.865885	3.004939	TRUE	HP_301241	TGCTGTTG/	GCATGTTT(	NM_011370	cytoplasmic FMR1 interacting protein 1	Cyfip1	2.8	SM2243	b	2	1.522164293
092308m3_V2MM_196667	1.864094	75.83547	TRUE	HP_414762	TGCTGTTG/	CCAGCCTG/	XM_113133	Mus musculus LOC195552 (LOC195552), mR.		2.13	SM2476	f	9	0.120127608
mPool2_V2MM_106855	1.86392	1.959154	TRUE	HP_327948	TGCTGTTG/	CGAACACT(	XM_131605	RIKEN cDNA 4921539E11 gene	4921539E11Rik	2.7	SM2155	e	7	1.707931489
mPool7_V2MM_83283	1.86167	3.004939	TRUE	HP_304831	TGCTGTTG/	CTGTTCAG(	NM_011914	Wolf-Hirschhorn syndrome candidate 2 (human)	Whsc2	2.9	SM2266	g	6	1.522164293
mPool7_V2MM_83283	1.86167	3.004939	TRUE	HP_304831	TGCTGTTG/	CTGTTCAG(	NM_011914	Wolf-Hirschhorn syndrome candidate 2 (human)	Whsc2	2.9	SM2266	g	6	1.522164293
mPool7_V2MM_112410	1.86156	3.004939	TRUE	HP_333414	TGCTGTTG/	CAGGAAAC	XM_136489	similar to fatty acid desaturase		2.9	SM2297	d	12	1.522164293
mPool7_V2MM_112410	1.86156	3.004939	TRUE	HP_333414	TGCTGTTG/	CAGGAAAC	XM_136489	similar to fatty acid desaturase		2.9	SM2297	d	12	1.522164293
092308m1_V2MM_179484	1.860226	2.205692	TRUE	HP_399958	TGCTGTTG/	CACTAATT(	XM_288341	Mus musculus LOC331656 (LOC331656), mR.		2.5	SM2076	b	2	1.656455229
092308m1_V2MM_179484	1.860226	2.205692	TRUE	HP_399958	TGCTGTTG/	CACTAATT(	XM_288341	Mus musculus LOC331656 (LOC331656), mRNA.		2.5	SM2076	b	2	1.656455229

mPool7_V2MM_88202	1.858436	3.004939	TRUE	HP_309644	TGCTGTTG/	CAATATTTC	NM_175342	RIKEN cD C330003B14 gene	C330003B14Rik		2.9	SM2267	h		7	1.522164293
mPool7_V2MM_88202	1.858436	3.004939	TRUE	HP_309644	TGCTGTTG/	CAATATTTC	NM_175342	RIKEN cDNA C330003B14 gene	C330003B14Rik		2.9	SM2267	h		7	1.522164293
092308m1_V2MM_152938	1.857777	3.004939	TRUE	HP_373600	TGCTGTTG/	GAGTGAAA	XM_195621	Mus musculus similar to squamous cell carcinoma antigen 2 [Mus musculus] (LOC26985			2.6	SM2117	g		7	1.522164293
mPool7_V2MM_109155	1.857661	2.205692	TRUE	HP_330220	TGCTGTTG/	GAGATCTT	BC057029	phosphodiesterase 2A, cGMP-stimulated	Pde2a		2.6	SM2149	c		7	1.656455229
092308m1_V2MM_147923	1.857462	3.004939	TRUE	HP_368634	TGCTGTTG/	CAATTTCCA	XM_163354	Mus musculus LOC215451 (LOC215451), mRNA.			2.5	SM2052	e		6	1.522164293
092308m1_V2MM_147923	1.857462	3.004939	TRUE	HP_368634	TGCTGTTG/	CAATTTCCA	XM_163354	Mus musculus LOC215451 (LOC215451), mR.			2.5	SM2052	e		6	1.522164293
092308m1_V2MM_174387	1.856481	2.205692	TRUE	HP_394872	TGCTGTTG/	GGTTAGTA	XM_287220	Mus musculus hypothetical gene supported by AK045957 (LOC329604), mR.			2.5	SM2095	d		12	1.656455229
mPool2_V2MM_145646	1.855991	3.004939	TRUE	HP_366357	TGCTGTTG/	CTGCAAGT	XM_161218	hypothetical gene supported by AK039704			2.1	SM2341	h		12	1.522164293
mPool7_V2MM_206248	1.855908	2.205692	TRUE	HP_424083	TGCTGTTG/	CTCTGCTAC	AK042897	RIKEN cDNA A930021C24 gene	A930021C24Rik		2.9	SM2300	d		7	1.656455229
092308m1_V2MM_160405	1.855381	2.205692	TRUE	HP_380985	TGCTGTTG/	CAGTCTAC/	AK088434	RIKEN cD E430016J11 gene	E430016J11Rik		2.6	SM2118	b		4	1.656455229
mPool7_V2MM_102528	1.854369	3.004939	TRUE	HP_323677	TGCTGTTG/	CTGCCATC/	AK036186	Wilms tumour 1-associating protein	Wtap		2.1	SM2303	g		5	1.522164293
mPool4_V2MM_162346	1.854303	75.83547	TRUE	NA	NA	NA	NA	NA	NA	NA		NA	NA	NA		0.120127608
mPool7_V2MM_94539	1.854125	2.205692	TRUE	HP_315826	TGCTGTTG/	CAGGAGTA	NM_183177	cDNA sequence BC052046	BC052046		2.9	SM2253	e		1	1.656455229
mPool2_V2MM_213976	1.852894	3.004939	TRUE	HP_431591	TGCTGTTG/	CTCTAATG	BC034735	RIKEN cDNA 2310047A01 gene	2310047A01Rik		2.11	SM2370	d		7	1.522164293
mPool2_V2MM_133635	1.8528	2.205692	TRUE	HP_354369	TGCTGTTG/	CCTGGAGT	BC050760	synaptotagmin 6	Syt6		2.11	SM2357	h		9	1.656455229
mPool2_V2MM_127171	1.852547	2.205692	TRUE	HP_348003	TGCTGTTG/	GATATACT	XM_145041	Mus musculus similar to RNA-binding region (RNP1, RRM) containing 2; splicing factor (			2.11	SM2369	a		1	1.656455229
092308m3_V2MM_222383	1.852379	75.83547	TRUE	NA	NA	NA	NA	NA	NA	NA		NA	NA	NA		0.120127608
mPool2_V2MM_86707	1.852379	2.205692	TRUE	HP_76909	TGCTGTTG/	GTACTGAT	NM_173760	expressed sequence AW555814	AW555814		2.6	SM2147	h		6	1.656455229
092308m1_V2MM_177190	1.852005	2.205692	TRUE	HP_397670	TGCTGTTG/	GTGCAAAT	XM_287819	Mus musculus hypothetical gene supported by AK090231 (LOC330797), mR.			2.5	SM2063	c		8	1.656455229
092308m1_V2MM_181893	1.848692	3.004939	TRUE	HP_402367	TGCTGTTG/	GTTTATTG	XM_288752	Mus musculus LOC332202 (LOC332202), mR.			2.5	SM2089	c		5	1.522164293
092308m1_V2MM_181893	1.848692	3.004939	TRUE	HP_402367	TGCTGTTG/	GTTTATTG	XM_288752	Mus musculus LOC332202 (LOC332202), mRNA.			2.5	SM2089	c		5	1.522164293
mPool7_V2MM_106336	1.847994	2.205692	TRUE	HP_327439	TGCTGTTG/	GTGATAAA	AK004863	alcohol dehydrogenase 6 (class V), pseudogene 1	Adh6-ps1		2.7	SM2163	d		6	1.656455229
092308m3_V2MM_53137	1.847345	62.64426	TRUE	HP_275503	TGCTGTTG/	GGGCAGAT	NM_016658	galactose-1-phosphate uridyl transferase	Galt		2.12	SM2416	a		7	0.203118686
mPool7_V2MM_176089	1.846342	1.959154	TRUE	HP_396571	TGCTGTTG/	CCAGGAAC	XM_287594	Mus musculus hypothetical gene supported by AK034606 (LOC330311), mRNA.			2.5	SM2063	h		12	1.707931489
mPool7_V2MM_176089	1.846342	1.959154	TRUE	HP_396571	TGCTGTTG/	CCAGGAAC	XM_287594	Mus musculus hypothetical gene supported by AK034606 (LOC330311), mR.			2.5	SM2063	h		12	1.707931489
mPool7_V2MM_98374	1.844994	3.004939	TRUE	HP_319602	TGCTGTTG/	CAGAAGAG	XM_112433	PREDICTED: Mus musculus RIKEN cDNA E130307J04 gene (E130307J04Rik), mRNA.			2.9	SM2267	e		7	1.522164293
092308m1_V2MM_62022	1.844357	3.004939	TRUE	HP_284139	TGCTGTTG/	CACCATGT	BC060160	transient receptor potential cation channel, subfamily C, mem	Trpc4		2.3	SM2014	b		12	1.522164293
mPool7_V2MM_197843	1.844133	2.205692	TRUE	HP_415905	TGCTGTTG/	CAGATTTG	NM_029498	zinc finger protein 198	Zfp198		2.8	SM2250	d		11	1.656455229
092308m3_V2MM_50774	1.843957	35.99881	TRUE	HP_273185	TGCTGTTG/	CATTTAGTT	D83204	protein tyrosine phosphatase, receptor type, J	Ptprj		2.12	SM2418	b		4	0.443711899
092308m1_V2MM_75845	1.843484	3.004939	TRUE	HP_297617	TGCTGTTG/	CCGGAAGA	NM_010720	lipase, endothelial	Lipg		2.3	SM2016	g		8	1.522164293
mPool2_V2MM_139267	1.843441	2.205692	TRUE	HP_359987	TGCTGTTG/	GTAGAAAT	XM_154892	Mus musculus LOC214538 (LOC214538), mRNA.			2.1	SM2342	c		2	1.656455229
092308m3_V2MM_141128	1.842728	59.99801	TRUE	HP_361841	TGCTGTTG/	CGGATCCC	XM_156476	Mus musculus LOC239968 (LOC239968), mR.			2.14	SM2528	c		8	0.221863149
mPool2_V2MM_105738	1.842222	3.004939	TRUE	HP_326848	TGCTGTTG/	CAGCAATT	AK005136	phosphatase and actin regulator 3	Phactr3		2.1	SM2323	c		11	1.522164293
mPool2_V2MM_105738	1.842222	3.004939	TRUE	HP_326848	TGCTGTTG/	CAGCAATT	AK005136	phosphatase and actin regulator 3	Phactr3		2.1	SM2323	c		11	1.522164293
092308m3_V2MM_144135	1.841662	66.95036	TRUE	HP_364846	TGCTGTTG/	GTAAGCGC	XM_159771	Mus musculus LOC241861 (LOC241861), mR.			2.14	SM2520	d		12	0.1742471
092308m1_V2MM_140674	1.841327	3.004939	TRUE	HP_361392	TGCTGTTG/	CACTAGAA	XM_156197	Mus musculus LOC208209 (LOC208209), mRNA.			2.5	SM2051	d		12	1.522164293
092308m1_V2MM_140674	1.841327	3.004939	TRUE	HP_361392	TGCTGTTG/	CACTAGAA	XM_156197	Mus musculus LOC208209 (LOC208209), mR.			2.5	SM2051	d		12	1.522164293
mPool2_V2MM_102079	1.841301	3.004939	TRUE	HP_323239	TGCTGTTG/	GAGAATAT	NM_198606	gene model 83, (NCBI)	Gm83		2.7	SM2162	e		2	1.522164293
mPool7_V2MM_147049	1.837501	3.004939	TRUE	HP_367760	TGCTGTTG/	CTCTTTGTA	XM_162569	Mus musculus LOC243509 (LOC243509), mR.			2.4	SM2046	d		5	1.522164293
mPool7_V2MM_147049	1.837501	3.004939	TRUE	HP_367760	TGCTGTTG/	CTCTTTGTA	XM_162569	Mus musculus LOC243509 (LOC243509), mRNA.			2.4	SM2046	d		5	1.522164293
mPool7_V2MM_92818	1.836666	3.004939	TRUE	HP_314160	TGCTGTTG/	CAGACACT	AK079097	RIKEN cDNA 9330186A19 gene	9330186A19Rik		2.9	SM2266	e		9	1.522164293
092308m1_V2MM_160106	1.836561	3.004939	TRUE	HP_380691	TGCTGTTG/	CCATAAAT	XM_283375	Mus musculus hypothetical gene supported by AK053631 (LOC328710), mR.			2.6	SM2119	g		6	1.522164293
092308m1_V2MM_157052	1.835591	3.004939	TRUE	HP_377674	TGCTGTTG/	GATGTTTA	XM_204790	Mus musculus similar to vomerosal 1 receptor, I9 [Mus musculus] (LOC277108), mR.			2.6	SM2124	g		1	1.522164293
mPool2_V2MM_92506	1.834843	3.004939	TRUE	HP_313854	TGCTGTTG/	GTAATGAT	NM_178723	zinc finger protein 533	Zfp533		2.6	SM2139	a		5	1.522164293
092308m1_V2MM_140837	1.834628	3.004939	TRUE	HP_361552	TGCTGTTG/	CTTTCTTTG	XM_156295	Mus musculus similar to pol protein [Phascolarctos cinereus] (LOC224231), mRNA.			2.1	SM2331	f		6	1.522164293
092308m1_V2MM_140837	1.834628	3.004939	TRUE	HP_361552	TGCTGTTG/	CTTTCTTTG	XM_156295	Mus musculus similar to pol protein [Phascolarctos cinereus] (LOC224231), mR.			2.1	SM2331	f		6	1.522164293
092308m1_V2MM_67924	1.834546	3.004939	TRUE	HP_289898	TGCTGTTG/	CTCTGTGA	NM_172998	RIKEN cD B830028P19 gene	B830028P19Rik		2.3	SM2011	a		4	1.522164293
mPool7_V2MM_170459	1.833132	3.004939	TRUE	HP_390952	TGCTGTTG/	GGCTTTAA	XM_283177	Mus musculus similar to hypothetical protein 3 - rat (LOC328328), mRNA.			2.5	SM2077	e		6	1.522164293
mPool7_V2MM_170459	1.833132	3.004939	TRUE	HP_390952	TGCTGTTG/	GGCTTTAA	XM_283177	Mus musculus similar to hypothetical protein 3 - rat (LOC328328), mR.			2.5	SM2077	e		6	1.522164293
mPool2_V2MM_30536	1.830474	2.205692	TRUE	HP_253460	TGCTGTTG/	CGGAATAT	NM_011941	mitogen activated protein kinase binding protein 1	Mapkbp1		2.11	SM2392	d		6	1.656455229
mPool7_V2MM_90885	1.829432	3.004939	TRUE	HP_312301	TGCTGTTG/	CTTTGAGC	NM_177880	hypothetical protein 4933411G11			2.9	SM2254	g		12	1.522164293
mPool7_V2MM_90842	1.828726	3.004939	TRUE	HP_312258	TGCTGTTG/	GGTTTACTT	NM_177869	expressed sequence AI847670	AI847670		2.8	SM2250	a		4	1.522164293
mPool7_V2MM_90842	1.828726	3.004939	TRUE	HP_312258	TGCTGTTG/	GGTTTACTT	NM_177869	expressed sequence AI847670	AI847670		2.8	SM2250	a		4	1.522164293
092308m1_V2MM_135185	1.825928	3.004939	TRUE	HP_355907	TGCTGTTG/	GTCTAAAT	XM_150392	protein phosphatase 1A, magnesium dependent, alpha isofor	Ppm1a		2.4	SM2045	e		12	1.522164293
092308m1_V2MM_135185	1.825928	3.004939	TRUE	HP_355907	TGCTGTTG/	GTCTAAAT	XM_150392	protein phosphatase 1A, magnesium dependent, alpha isofor	Ppm1a		2.4	SM2045	e		12	1.522164293
mPool2_V2MM_1304	1.825761	2.205692	TRUE	HP_224951	TGCTGTTG/	CCAATTCTA	NM_008125	gap junction membrane channel protein beta 2	Gjb2		2.11	SM2379	h		6	1.656455229
mPool7_V2MM_195719	1.82571	2.205692	TRUE	HP_413846	TGCTGTTG/	GATCATCT	NM_178376	Ras-related GTP binding A	Rraga		2.9	SM2259	e		4	1.656455229
mPool7_V2MM_63424	1.823698	2.205692	TRUE	HP_285503	TGCTGTTG/	CGGATATC	NM_177383	G protein-coupled receptor 21	Gpr21		2.8	SM2243	c		12	1.656455229
mPool7_V2MM_103070	1.821865	2.205692	TRUE	HP_324214	TGCTGTTG/	CTATTTCTA	NM_199466	echinoderm microtubule associated protein like 4	Eml4		2.1	SM2321	c		11	1.656455229
mPool2_V2MM_127282	1.821688	2.205692	TRUE	HP_348114	TGCTGTTG/	GAAAGGAG	XM_145108	Mus musculus LOC243714 (LOC243714), mRNA.			2.11	SM2369	g		5	1.656455229
092308m1_V2MM_137091	1.821151	2.205692	TRUE	HP_357811	TGCTGTTG/	GGCTCTAT	XM_152567	Mus musculus LOC237736 (LOC237736), mR.			2.5	SM2052	c		3	1.656455229
mPool7_V2MM_103172	1.820546	3.004939	TRUE	HP_324314	TGCTGTTG/	GAGACATA	XM_128858	RIKEN cDNA 4833403I15 gene	4833403I15Rik		2.1	SM2315	h		4	1.522164293

092308m1_V2MM_178263	1.820437	2.205692	TRUE	HP_398737	TGCTGTTG	CATCCTAA	XM_288120	Mus musculus hypothetical gene supported by AK054126 (LOC331473), mR.		2.5	SM2079	h	1	1.656455229
mPool7_V2MM_89907	1.820017	3.004939	TRUE	HP_311334	TGCTGTTG	CATCTTCTT	BC025913	biogenesis of lysosome-related organelles complex-1, subunit	Bloc1s3	2.9	SM2266	f	11	1.522164293
mPool7_V2MM_89907	1.820017	3.004939	TRUE	HP_311334	TGCTGTTG	CATCTTCTT	BC025913	biogenesis of lysosome-related organelles complex-1, subunit	Bloc1s3	2.9	SM2266	f	11	1.522164293
mPool7_V2MM_175564	1.818907	3.004939	TRUE	HP_396046	TGCTGTTG	GCCATTGC	XM_489100	hypothetical gene supported by AK031827		2.5	SM2078	d	9	1.522164293
mPool7_V2MM_175564	1.818907	3.004939	TRUE	HP_396046	TGCTGTTG	GCCATTGC	XM_489100	hypothetical gene supported by AK031827		2.5	SM2078	d	9	1.522164293
092308m1_V2MM_175874	1.817468	2.205692	TRUE	HP_396356	TGCTGTTG	CAAATCTT	XM_287545	similar to PABP1-dependent poly A-specific ribonuclease subunit PAN3; PABP-depender		2.5	SM2066	a	6	1.656455229
092308m3_V2MM_139822	1.817454	61.29828	TRUE	HP_360542	TGCTGTTG	GGGCCAGC	XM_155408	Mus musculus LOC239495 (LOC239495), mR.		2.14	SM2514	d	12	0.212551731
mPool6_V2MM_77615	1.81742	75.83547	TRUE	HP_299330	TGCTGTTG	CTGTGTTT	NM_025784	BCS1-like (yeast)	Bcs1l	2.7	SM2175	b	7	0.120127608
092308m1_V2MM_178019	1.814586	2.205692	TRUE	HP_398496	TGCTGTTG	CAGGGAAC	XM_288010	Mus musculus hypothetical gene supported by AK076622 (LOC331162), mR.		2.5	SM2094	h	1	1.656455229
092308m1_V2MM_178353	1.813933	3.004939	TRUE	HP_398827	TGCTGTTG	CCAAGTAC	XM_288143	Mus musculus hypothetical gene supported by AK046508 (LOC331558), mRNA.		2.5	SM2069	g	10	1.522164293
092308m1_V2MM_178353	1.813933	3.004939	TRUE	HP_398827	TGCTGTTG	CCAAGTAC	XM_288143	Mus musculus hypothetical gene supported by AK046508 (LOC331558), mR.		2.5	SM2069	g	10	1.522164293
mPool7_V2MM_150314	1.813855	3.004939	TRUE	HP_371006	TGCTGTTG	GTTTCCTAC	NM_177171	RIKEN cDNA D930036F22 gene	D930036F22Rik	2.1	SM2328	g	8	1.522164293
mPool7_V2MM_92636	1.813438	3.004939	TRUE	HP_313981	TGCTGTTG	GTAGGAGT	NM_178745	RIKEN cDNA 6330442E10 gene	6330442E10Rik	2.9	SM2262	c	6	1.522164293
mPool2_V2MM_160247	1.813302	2.205692	TRUE	HP_380830	TGCTGTTG	CCAGGAAC	XM_283435	Mus musculus similar to RIKEN cDNA 2010003O02 [Mus musculus] (LOC328796), mRNA		2.6	SM2126	d	8	1.656455229
mPool7_V2MM_74933	1.812848	2.205692	TRUE	HP_296722	TGCTGTTG	CAGTTTAT	NM_028836	chitobiase, di-N-acetyl-	Ctbs	2.8	SM2236	g	4	1.656455229
mPool2_V2MM_145856	1.812021	3.004939	TRUE	HP_366567	TGCTGTTG	GTCAGAAC	XM_161546	Mus musculus LOC242761 (LOC242761), mRNA.		2.1	SM2345	e	4	1.522164293
mPool2_V2MM_140268	1.811281	3.004939	TRUE	HP_360988	TGCTGTTG	GCTATGTG	XM_155816	Mus musculus LOC239490 (LOC239490), mR.		2.4	SM2042	g	7	1.522164293
mPool2_V2MM_140268	1.811281	3.004939	TRUE	HP_360988	TGCTGTTG	GCTATGTG	XM_155816	Mus musculus LOC239490 (LOC239490), mRNA.		2.4	SM2042	g	7	1.522164293
092308m3_V2MM_208411	1.811041	66.95036	TRUE	HP_426201	TGCTGTTG	GCACTTGC	XM_159344	Mus musculus LOC245565 (LOC245565), mR.		2.14	SM2520	b	9	0.1742471
mPool7_V2MM_84662	1.808608	2.205692	TRUE	HP_306168	TGCTGTTG	CGTTGATT	NM_028848	RIKEN cDNA 4930513F16 gene	4930513F16Rik	2.9	SM2259	b	8	1.656455229
mPool7_V2MM_77897	1.808162	3.004939	TRUE	HP_105049	TGCTGTTG	GAAACCAT	AB086123	calcium channel, voltage-dependent, L type, alpha 1D subunit	Cacna1d	2.8	SM2236	a	11	1.522164293
092308m1_V2MM_180607	1.807655	3.004939	TRUE	HP_401081	TGCTGTTG	CTATGAAT	XM_358439	gene model 1573, (NCBI)	Gm1573	2.5	SM2065	h	7	1.522164293
092308m1_V2MM_180607	1.807655	3.004939	TRUE	HP_401081	TGCTGTTG	CTATGAAT	XM_358439	gene model 1573, (NCBI)	Gm1573	2.5	SM2065	h	7	1.522164293
092308m1_V2MM_154308	1.806754	3.004939	TRUE	HP_374949	TGCTGTTG	CCCTTGGT	AK045988	RIKEN cD 6530403G13 gene	6530403G13Rik	2.6	SM2115	d	9	1.522164293
mPool2_V2MM_143833	1.804551	2.205692	TRUE	HP_364545	TGCTGTTG	CATGAACA	XM_159183	Mus musculus LOC245402 (LOC245402), mR.		2.4	SM2038	f	10	1.656455229
mPool2_V2MM_143833	1.804551	2.205692	TRUE	HP_364545	TGCTGTTG	CATGAACA	XM_159183	Mus musculus LOC245402 (LOC245402), mRNA.		2.4	SM2038	f	10	1.656455229
092308m1_V2MM_171110	1.801508	2.205692	TRUE	HP_391599	TGCTGTTG	CGTATTTAC	XM_286558	Mus musculus hypothetical gene supported by AK044419 (LOC328319), mR.		2.5	SM2072	b	7	1.656455229
092308m1_V2MM_69402	1.801135	3.004939	TRUE	HP_36745	TGCTGTTG	GCCTGACT	BC082537	RIKEN cD 2900042E01 gene	2900042E01Rik	2.3	SM2003	f	3	1.522164293
mPool6_V2MM_158860	1.800228	75.83547	TRUE	HP_379465	TGCTGTTG	GCTGCTGT	XM_282894	Mus musculus hypothetical gene supported by AK042566 (LOC327726), mRNA.		2.8	SM2208	e	6	0.120127608
mPool4_V2MM_15470	1.800172	75.83547	TRUE	HP_238793	TGCTGTTG	CTCTGTGT	NM_011407	schlafen 1	Slfn1	2.15	SM2573	b	2	0.120127608
mPool7_V2MM_84868	1.799685	2.205692	TRUE	HP_306368	TGCTGTTG	CTTCATCCC	NM_029705	Machado-Joseph disease (spinocerebellar ataxia 3, olivopontc	Mjd	2.9	SM2261	c	5	1.656455229
mPool7_V2MM_196416	1.799305	3.004939	TRUE	HP_414521	TGCTGTTG	CTGTAAGT	NM_175479	RIKEN cDNA A330008L17 gene	A330008L17Rik	2.9	SM2254	a	4	1.522164293
mPool2_V2MM_212683	1.798955	2.205692	TRUE	HP_430345	TGCTGTTG	CACATCAT	XM_142433	similar to hypothetical protein		2.11	SM2370	a	6	1.656455229
mPool2_V2MM_110315	1.796038	3.004939	TRUE	HP_331352	TGCTGTTG	GACCCTAA	XM_134580	gene model 177, (NCBI)	Gm177	2.6	SM2149	h	5	1.522164293
092308m1_V2MM_151863	1.795947	2.205692	TRUE	HP_372530	TGCTGTTG	CCCATGTT	XM_194974	similar to ribosomal protein		2.5	SM2052	h	3	1.656455229
mPool2_V2MM_85938	1.7941	3.004939	TRUE	HP_307418	TGCTGTTG	CAGCTATT	NM_146524	olfactory receptor 855	Olfr855	2.6	SM2146	c	10	1.522164293
092308m1_V2MM_146584	1.793485	3.004939	TRUE	HP_367295	TGCTGTTG	CATAATTG	XM_162162	Mus musculus LOC243184 (LOC243184), mR.		2.5	SM2058	a	9	1.522164293
092308m1_V2MM_146584	1.793485	3.004939	TRUE	HP_367295	TGCTGTTG	CATAATTG	XM_162162	Mus musculus LOC243184 (LOC243184), mRNA.		2.5	SM2058	a	9	1.522164293
mPool7_V2MM_177261	1.792334	3.004939	TRUE	NA	NA	NA	NA	NA	NA	NA	NA	NA	NA	1.522164293
092308m1_V2MM_139509	1.790566	3.004939	TRUE	HP_360229	TGCTGTTG	CTACTATCA	XM_155129	Mus musculus LOC239255 (LOC239255), mRNA.		2.4	SM2040	h	5	1.522164293
092308m1_V2MM_139509	1.790566	3.004939	TRUE	HP_360229	TGCTGTTG	CTACTATCA	XM_155129	Mus musculus LOC239255 (LOC239255), mR.		2.4	SM2040	h	5	1.522164293
092308m1_V2MM_187096	1.788755	2.205692	TRUE	HP_407565	TGCTGTTG	CCCTTAAG	XM_289648	Mus musculus LOC333392 (LOC333392), mR.		2.5	SM2072	e	8	1.656455229
mPool7_V2MM_68109	1.787386	1.959154	TRUE	HP_107766	TGCTGTTG	CTGGAAAC	NM_026360	DEAD (Asp-Glu-Ala-Asp) box polypeptide 47	Ddx47	2.8	SM2243	d	1	1.707931489
mPool7_V2MM_173441	1.787223	3.004939	TRUE	HP_393927	TGCTGTTG	CAAACATA	AK035520	hypothetical protein 9530060I07		2.5	SM2081	c	2	1.522164293
mPool7_V2MM_173441	1.787223	3.004939	TRUE	HP_393927	TGCTGTTG	CAAACATA	AK035520	hypothetical protein 9530060I07		2.5	SM2081	c	2	1.522164293
092308m1_V2MM_158543	1.785906	3.004939	TRUE	HP_379153	TGCTGTTG	CTCAAGCA	XM_206745	Mus musculus LOC279156 (LOC279156), mR.		2.6	SM2115	e	10	1.522164293
mPool2_V2MM_133812	1.78557	3.004939	TRUE	HP_354545	TGCTGTTG	CTGCTTATC	NM_177669	RIKEN cDNA A630098G03 gene	A630098G03Rik	2.11	SM2364	a	10	1.522164293
mPool2_V2MM_214867	1.785552	3.004939	TRUE	HP_432439	TGCTGTTG	CATGATTA	XM_485031	olfactory receptor 48	Olfr48	2.11	SM2351	f	12	1.522164293
092308m1_V2MM_158554	1.784753	3.004939	TRUE	HP_379164	TGCTGTTG	GCCCAAAT	XM_206756	similar to F1N19.3		2.6	SM2121	f	9	1.522164293
092308m1_V2MM_74731	1.783928	3.004939	TRUE	HP_296529	TGCTGTTG	CAGGAACT	NM_173431	RIKEN cD 1700047E16 gene	1700047E16Rik	2.3	SM2009	e	9	1.522164293
mPool2_V2MM_128028	1.783348	3.004939	TRUE	HP_348846	TGCTGTTG	GCCTTTAG	XM_145500	RIKEN cDNA 1110003H10 gene	1110003H10Rik	2.11	SM2351	h	6	1.522164293
mPool2_V2MM_101379	1.782748	3.004939	TRUE	NA	NA	NA	NA	NA	NA	NA	NA	NA	NA	1.522164293
092308m1_V2MM_68358	1.782434	3.004939	TRUE	HP_290320	TGCTGTTG	CTTTCTTGC	NM_146851	olfactory receptor 295	Olfr295	2.3	SM2009	f	4	1.522164293
mPool7_V2MM_202589	1.782339	3.004939	TRUE	HP_420589	TGCTGTTG	CCTGGAGA	XM_127675	Mus musculus similar to KIAA1607 protein [Homo sapiens] (LOC218897), mRNA.		2.1	SM2306	b	1	1.522164293
mPool2_V2MM_20404	1.781467	2.205692	TRUE	HP_222494	TGCTGTTG	GTTATATA	NM_010756	v-maf musculoaponeurotic fibrosarcoma oncogene family, pr	Mafg	2.11	SM2386	f	10	1.656455229
mPool2_V2MM_134442	1.781304	3.004939	TRUE	HP_355168	TGCTGTTG	CTGTTCTTA	AK046418	RIKEN cD 4930415J05 gene	4930415J05Rik	2.6	SM2104	c	10	1.522164293
mPool2_V2MM_134442	1.781304	3.004939	TRUE	HP_355168	TGCTGTTG	CTGTTCTTA	AK046418	RIKEN cDNA 4930415J05 gene	4930415J05Rik	2.6	SM2104	c	10	1.522164293
092308m1_V2MM_183610	1.781	2.205692	TRUE	HP_404084	TGCTGTTG	CCGCTTAA	XM_289049	Mus musculus LOC333489 (LOC333489), mR.		2.5	SM2081	b	8	1.656455229
092308m3_V2MM_206507	1.780459	75.83547	TRUE	HP_424329	TGCTGTTG	CACACTTT	XM_128374	zinc finger protein 294	Zfp294	2.1	SM2318	g	12	0.120127608
092308m3_V2MM_206507	1.780459	75.83547	TRUE	HP_424329	TGCTGTTG	CACACTTT	XM_128374	zinc finger protein 294	Zfp294	2.1	SM2318	g	12	0.120127608
092308m1_V2MM_142730	1.779285	2.205692	TRUE	HP_363443	TGCTGTTG	GTGAATAT	XM_157867	Mus musculus LOC240547 (LOC240547), mR.		2.5	SM2059	g	4	1.656455229
092308m1_V2MM_142730	1.779285	2.205692	TRUE	HP_363443	TGCTGTTG	GTGAATAT	XM_157867	Mus musculus LOC240547 (LOC240547), mRNA.		2.5	SM2059	g	4	1.656455229

mPool7_V2MM_171353	1.779272	1.959154	TRUE	HP_391842	TGCTGTTG/	GATTTATTA	XM_488707	hypothetical gene supported by AK048353		2.9	SM2274	b	3	1.707931489
092308m1_V2MM_125180	1.777149	3.004939	TRUE	HP_346024	TGCTGTTG/	CAGCAAGA	BC059253	CDK5 regulatory subunit associated protein 2	Cdk5rap2	2.6	SM2101	a	7	1.522164293
092308m1_V2MM_170810	1.775773	3.004939	TRUE	HP_391300	TGCTGTTG/	CCCAGAAC(	AK045681	hypothetical protein B230303A05		2.5	SM2080	a	8	1.522164293
mPool2_V2MM_125198	1.775367	3.004939	TRUE	HP_346042	TGCTGTTG/	CATGTTGA1	XM_143850	Mus musculus similar to pre-mRNA splicing factor Prp16; pre-mRNA splicing factor ATP-		2.11	SM2366	c	8	1.522164293
092308m3_V2MM_59498	1.774868	62.64426	TRUE	HP_281676	TGCTGTTG/	GCCTTTGCT	NM_029295	chemokine-like factor	Cklf	2.12	SM2407	c	9	0.203118686
mPool7_V2MM_85962	1.774285	3.004939	TRUE	HP_307442	TGCTGTTG/	CATGGATA(	NM_146530	olfactory receptor 1186	Olfr1186	2.9	SM2268	e	12	1.522164293
mPool7_V2MM_179499	1.773159	3.004939	TRUE	HP_399973	TGCTGTTG/	CAGAGTTT(	XM_288344	Mus musculus LOC327806 (LOC327806), mRNA.		2.5	SM2062	e	2	1.522164293
mPool7_V2MM_179499	1.773159	3.004939	TRUE	HP_399973	TGCTGTTG/	CAGAGTTT(	XM_288344	Mus musculus LOC327806 (LOC327806), mR.		2.5	SM2062	e	2	1.522164293
mPool2_V2MM_212338	1.772796	2.205692	TRUE	HP_430008	TGCTGTTG/	GACATAAT	AK048574	RIKEN cDNA C130078N17 gene	C130078N17Rik	2.1	SM2350	a	3	1.656455229
mPool7_V2MM_67315	1.772663	3.004939	TRUE	HP_289306	TGCTGTTG/	CCTCCGCT(	NM_021532	dapper homolog 1, antagonist of beta-catenin (xenopus)	Dact1	2.8	SM2230	h	1	1.522164293
092308m1_V2MM_62745	1.771299	3.004939	TRUE	HP_284843	TGCTGTTG/	CATCCAAT(	Z11981	plasmacytoma variant translocation 1	Pvt1	2.3	SM2013	h	7	1.522164293
mPool2_V2MM_133636	1.771069	3.004939	TRUE	HP_354370	TGCTGTTG/	CTGGAGTT	BC050760	synaptotagmin 6	Syt6	2.11	SM2374	f	3	1.522164293
mPool7_V2MM_75235	1.770862	3.004939	TRUE	HP_297018	TGCTGTTG/	CCACCAAT/	NM_026391	protein phosphatase 2, regulatory subunit B, delta isoform	Ppp2r2d	2.8	SM2240	b	4	1.522164293
mPool7_V2MM_140837	1.77001	2.205692	TRUE	HP_361552	TGCTGTTG/	CTTTCTTTG	XM_156295	Mus musculus similar to pol protein [Phascolarctos cinereus] (LOC224231), mRNA.		2.1	SM2331	f	6	1.656455229
mPool7_V2MM_140837	1.77001	2.205692	TRUE	HP_361552	TGCTGTTG/	CTTTCTTTG	XM_156295	Mus musculus similar to pol protein [Phascolarctos cinereus] (LOC224231), mR.		2.1	SM2331	f	6	1.656455229
092308m1_V2MM_128385	1.767592	3.004939	TRUE	HP_349199	TGCTGTTG/	CTCATGTT(	XM_145667	transient receptor potential cation channel, subfamily M, mer	Trpm1	2.5	SM2100	g	12	1.522164293
mPool7_V2MM_195901	1.765877	3.004939	TRUE	HP_414020	TGCTGTTG/	CATGGTTA(	NM_178665	LIM domain containing preferred translocation partner in lipo	Lpp	2.8	SM2250	b	1	1.522164293
mPool2_V2MM_132692	1.764918	3.004939	TRUE	HP_353442	TGCTGTTG/	GAGACATT	AK044430	mutated in colorectal cancers	Mcc	2.1	SM2350	a	8	1.522164293
mPool7_V2MM_151228	1.762266	2.205692	TRUE	HP_371899	TGCTGTTG/	GTGAAATA	NM_001002272	trophinin	Tro	2.4	SM2050	c	10	1.656455229
mPool7_V2MM_151228	1.762266	2.205692	TRUE	HP_371899	TGCTGTTG/	GTGAAATA	NM_001002272	trophinin	Tro	2.4	SM2050	c	10	1.656455229
mPool7_V2MM_179069	1.759908	3.004939	TRUE	HP_399543	TGCTGTTG/	CTATTAAA(	XM_288269	Mus musculus LOC332542 (LOC332542), mR.		2.5	SM2091	c	7	1.522164293
mPool7_V2MM_179069	1.759908	3.004939	TRUE	HP_399543	TGCTGTTG/	CTATTAAA(	XM_288269	Mus musculus LOC332542 (LOC332542), mRNA.		2.5	SM2091	c	7	1.522164293
mPool7_V2MM_196009	1.758391	3.004939	TRUE	HP_414125	TGCTGTTG/	CTTGTATA(	NM_173757	mitochondrial ribosomal protein S27	Mrps27	2.9	SM2266	e	5	1.522164293
mPool7_V2MM_86066	1.757119	3.004939	TRUE	HP_307543	TGCTGTTG/	GAGTTTAC/	NM_146613	olfactory receptor 973	Olfr973	2.9	SM2270	b	6	1.522164293
mPool7_V2MM_170871	1.757114	3.004939	TRUE	HP_391361	TGCTGTTG/	CGTTAAAT(	XM_286509	Mus musculus hypothetical gene supported by AK037084 (LOC328225), mR.		2.5	SM2072	g	5	1.522164293
mPool7_V2MM_170871	1.757114	3.004939	TRUE	HP_391361	TGCTGTTG/	CGTTAAAT(	XM_286509	Mus musculus hypothetical gene supported by AK037084 (LOC328225), mRNA.		2.5	SM2072	g	5	1.522164293
mPool2_V2MM_192335	1.756741	1.959154	TRUE	HP_337891	TGCTGTTG/	GTAACATA(	XM_354831	olfactomedin 4	Olfm4	2.1	SM2308	h	6	1.707931489
mPool2_V2MM_126250	1.756123	2.205692	TRUE	HP_347093	TGCTGTTG/	CGTACAAC/	XM_144444	Mus musculus similar to 60S ACIDIC RIBOSOMAL PROTEIN P1 (LOC243162), mRNA.		2.11	SM2355	e	7	1.656455229
mPool2_V2MM_210602	1.755649	3.004939	TRUE	HP_428346	TGCTGTTG/	CAACCACA(	XM_143694	Mus musculus similar to glyceraldehyde-3-phosphate dehydrogenase [Mus musculus] (l		2.11	SM2362	e	12	1.522164293
mPool2_V2MM_211682	1.755483	3.004939	TRUE	HP_429374	TGCTGTTG/	GGCAATAA	XM_143642	Mus musculus similar to eukaryotic translation initiation factor 4A1; initiation factor eIF		2.11	SM2352	f	11	1.522164293
mPool7_V2MM_172300	1.754816	2.205692	TRUE	HP_392786	TGCTGTTG/	GTTTGTAA/	XM_286796	Mus musculus hypothetical gene supported by AK042793 (LOC328772), mR.		2.5	SM2079	c	5	1.656455229
mPool7_V2MM_172300	1.754816	2.205692	TRUE	HP_392786	TGCTGTTG/	GTTTGTAA/	XM_286796	Mus musculus hypothetical gene supported by AK042793 (LOC328772), mRNA.		2.5	SM2079	c	5	1.656455229
mPool2_V2MM_4516	1.754703	3.004939	TRUE	HP_228089	TGCTGTTG/	GTTATAAA(	NM_027374	peptidylprolyl isomerase (cyclophilin)-like 3	Ppil3	2.11	SM2375	b	1	1.522164293
mPool7_V2MM_195279	1.754677	3.004939	TRUE	HP_413422	TGCTGTTG/	CCATATGT(	NM_146436	olfactory receptor 998	Olfr998	2.9	SM2257	d	12	1.522164293
mPool7_V2MM_195279	1.754677	3.004939	TRUE	HP_413422	TGCTGTTG/	CCATATGT(	NM_146436	olfactory receptor 998	Olfr998	2.9	SM2257	d	12	1.522164293
mPool2_V2MM_151870	1.752762	3.004939	TRUE	HP_372537	TGCTGTTG/	CTCTGTGTT	XM_194977	similar to Cytochrome P450, family 2, subfamily d, polypeptide 13		2.1	SM2345	g	5	1.522164293
092308m1_V2MM_176983	1.750992	2.205692	TRUE	HP_397463	TGCTGTTG/	CTACTTACA	AK081575	expressed sequence W91709	W91709	2.5	SM2069	b	3	1.656455229
mPool2_V2MM_6424	1.750325	2.205692	TRUE	HP_229960	TGCTGTTG/	CTGCTAAA(	NM_177992	guanosine monophosphate reductase 2	Gmpr2	2.11	SM2377	f	4	1.656455229
092308m1_V2MM_170621	1.749821	2.205692	TRUE	HP_391111	TGCTGTTG/	CTGTCAGT(	XM_286455	Mus musculus hypothetical gene supported by AK039743 (LOC328163), mR.		2.5	SM2060	f	3	1.656455229
mPool2_V2MM_210737	1.7487	2.205692	TRUE	HP_428476	TGCTGTTG/	CTAAGTAT(	XM_144834	gene model 1409, (NCBI)	Gm1409	2.11	SM2371	e	3	1.656455229
mPool2_V2MM_128586	1.748574	3.004939	TRUE	HP_349399	TGCTGTTG/	CTACTGAG/	XM_145773	Mus musculus similar to olfactory receptor MOR10-1 [Mus musculus] (LOC233592), mR		2.11	SM2364	g	2	1.522164293
mPool2_V2MM_170258	1.748132	2.205692	TRUE	HP_390753	TGCTGTTG/	CACACACT(	AK083360	hypothetical protein C920021A13		2.6	SM2132	b	2	1.656455229
mPool7_V2MM_116096	1.747098	2.205692	TRUE	HP_337051	TGCTGTTG/	CTGATATT(	XM_138748	Mus musculus similar to envelope protein [Ovis aries] (LOC218362), mRNA.		2.6	SM2150	c	2	1.656455229
mPool2_V2MM_10590	1.746522	3.004939	TRUE	HP_234016	TGCTGTTG/	CTGAAACT	NM_007687	cofilin 1, non-muscle	Cfl1	2.11	SM2386	b	3	1.522164293
092308m1_V2MM_167494	1.746275	3.004939	TRUE	HP_388007	TGCTGTTG/	CACAGAGG	XM_285777	Mus musculus similar to Involved in chitin synthase III activity, also required for homozy		2.6	SM2117	g	10	1.522164293
mPool7_V2MM_103760	1.746163	1.959154	TRUE	HP_324898	TGCTGTTG/	GCTTATTTG	AK008974	RIKEN cD 2210418G03 gene	2210418G03Rik	2.9	SM2297	a	4	1.707931489
mPool7_V2MM_103760	1.746163	1.959154	TRUE	HP_324898	TGCTGTTG/	GCTTATTTG	AK008974	RIKEN cDNA 2210418G03 gene	2210418G03Rik	2.9	SM2297	a	4	1.707931489
mPool7_V2MM_175995	1.745676	3.004939	TRUE	HP_396477	TGCTGTTG/	CACACCCT(	AK033923	hypothetical protein 9330117B14		2.9	SM2274	f	4	1.522164293
mPool2_V2MM_121810	1.745608	2.205692	TRUE	HP_342685	TGCTGTTG/	CTGTCTTAT	XM_141857	Mus musculus similar to RIKEN cDNA 1700042B14 [Mus musculus] (LOC236872), mRNA		2.11	SM2364	h	7	1.656455229
092308m1_V2MM_161736	1.742692	3.004939	TRUE	HP_382300	TGCTGTTG/	CTCTCGGA(	XM_284026	Mus musculus hypothetical gene supported by AK030056 (LOC329969), mR.		2.6	SM2115	d	10	1.522164293
mPool2_V2MM_32277	1.741137	3.004939	TRUE	HP_255163	TGCTGTTG/	GGAAATTA	NM_023587	protein tyrosine phosphatase-like (proline instead of catalytic	Ptplb	2.11	SM2392	b	11	1.522164293
mPool7_V2MM_89806	1.741072	3.004939	TRUE	HP_311234	TGCTGTTG/	GTGAGCGT	NM_177674	hypothetical protein A530045M11		2.9	SM2269	b	6	1.522164293
092308m1_V2MM_175197	1.740383	2.205692	TRUE	HP_395682	TGCTGTTG/	CTGTTTCTC	XM_287390	Mus musculus hypothetical gene supported by AK089399 (LOC329956), mR.		2.5	SM2095	c	11	1.656455229
mPool7_V2MM_199289	1.738678	3.004939	TRUE	HP_417337	TGCTGTTG/	CATTGTTTC	XM_286732	expressed sequence AU022320	AU022320	2.9	SM2288	h	11	1.522164293
mPool2_V2MM_130962	1.738175	3.004939	TRUE	HP_351737	TGCTGTTG/	GTCTGTAC/	XM_147124	RIKEN cDNA 5830454E08 gene	5830454E08Rik	2.11	SM2360	d	8	1.522164293
mPool7_V2MM_85524	1.738001	0	TRUE	HP_307005	TGCTGTTG/	GTCCTACT(	NM_146402	olfactory receptor 1303	Olfr1303	2.9	SM2254	h	9	#NUM!
092308m3_V2MM_196361	1.737549	75.83547	TRUE	HP_414466	TGCTGTTG/	CCTGGAAC	NM_010165	eyes absent 2 homolog (Drosophila)	Eya2	2.12	SM2450	h	9	0.120127608
mPool7_V2MM_90042	1.737532	2.205692	TRUE	HP_311468	TGCTGTTG/	GTGAGTGG	NM_177721	RAN binding protein 6	Ranbp6	2.9	SM2254	a	3	1.656455229
mPool7_V2MM_90042	1.737532	2.205692	TRUE	HP_311468	TGCTGTTG/	GTGAGTGG	NM_177721	RAN binding protein 6	Ranbp6	2.9	SM2254	a	3	1.656455229
092308m1_V2MM_144171	1.736964	3.004939	TRUE	HP_364882	TGCTGTTG/	CCATTTGT(	XM_159785	Mus musculus LOC207783 (LOC207783), mR.		2.5	SM2056	a	3	1.522164293
mPool2_V2MM_4227	1.735435	2.205692	TRUE	HP_227803	TGCTGTTG/	GGGTTTAT/	NM_007568	betacellulin, epidermal growth factor family member	Btc	2.11	SM2385	b	2	1.656455229
092308m1_V2MM_161793	1.734441	3.004939	TRUE	HP_382357	TGCTGTTG/	CAGAGTAC(	XM_284053	six transmembrane epithelial antigen of prostate 2	Steap2	2.6	SM2118	b	7	1.522164293

mPool2_V2MM_2066	1.733813	3.004939	TRUE	HP_225694	TGCTGTTG/	CTACTACTA	NM_008288	hydroxysteroid 11-beta dehydrogenase 1	Hsd11b1		2.11	SM2385	g		3	1.522164293
092308m1_V2MM_127129	1.733755	3.004939	TRUE	HP_347961	TGCTGTTG/	CCACCTTAC	XM_145005	IQ motif and Sec7 domain 3	Iqsec3		2.6	SM2104	a		9	1.522164293
mPool2_V2MM_107909	1.732894	2.205692	TRUE	HP_328988	TGCTGTTG/	CTGATCAT/	AK014627	RIKEN cDNA 4731417B20 gene	4731417B20Rik		2.6	SM2148	c		11	1.656455229
mPool7_V2MM_83844	1.732078	2.205692	TRUE	HP_305368	TGCTGTTG/	GATTAAATI	NM_025700	phosphoglucomutase 1	Pgm1		2.9	SM2270	c		9	1.656455229
mPool7_V2MM_83844	1.732078	2.205692	TRUE	HP_305368	TGCTGTTG/	GATTAAATI	NM_025700	phosphoglucomutase 1	Pgm1		2.9	SM2270	c		9	1.656455229
mPool2_V2MM_210885	1.731604	2.205692	TRUE	HP_428620	TGCTGTTG/	GCCAGATT	XM_143332	RIKEN cDNA 2010204N08 gene	2010204N08Rik		2.11	SM2368	g		5	1.656455229
mPool2_V2MM_132088	1.731498	2.205692	TRUE	HP_352842	TGCTGTTG/	GTTATAGA/	XM_489508	RIKEN cDNA 1810013L24 gene	1810013L24Rik		2.11	SM2359	c		8	1.656455229
092308m3_V2MM_32715	1.731085	66.95036	TRUE	HP_255590	TGCTGTTG/	GTGCTTCC(	NM_145602	N-myc downstream regulated gene 4	Ndrg4		2.11	SM2400	h		9	0.1742471
mPool2_V2MM_142730	1.730535	2.205692	TRUE	HP_363443	TGCTGTTG/	GTGAATAT	XM_157867	Mus musculus LOC240547 (LOC240547), mR.			2.5	SM2059	g		4	1.656455229
mPool2_V2MM_142730	1.730535	2.205692	TRUE	HP_363443	TGCTGTTG/	GTGAATAT	XM_157867	Mus musculus LOC240547 (LOC240547), mRNA.			2.5	SM2059	g		4	1.656455229
mPool2_V2MM_122425	1.729666	3.004939	TRUE	HP_343291	TGCTGTTG/	GGATAAGT	XM_142191	gene model 384, (NCBI)	Gm384		2.1	SM2349	a		10	1.522164293
092308m1_V2MM_136395	1.729153	2.205692	TRUE	HP_357115	TGCTGTTG/	CTGCAGAG	XM_151691	Mus musculus LOC237325 (LOC237325), mR.			2.4	SM2046	d		9	1.656455229
mPool2_V2MM_129626	1.728163	2.205692	TRUE	HP_350427	TGCTGTTG/	CTAATACT/	XM_146376	Mus musculus similar to Ddx10 protein (LOC234281), mRNA.			2.11	SM2373	f		1	1.656455229
mPool7_V2MM_207918	1.727276	1.959154	TRUE	HP_425715	TGCTGTTG/	CAGATAGC	AY462058	kininogen 1	Kng1		2.1	SM2332	g		6	1.707931489
mPool5_V2MM_59506	1.727234	66.95036	TRUE	HP_281684	TGCTGTTG/	GCGAAGAT	NM_010111	ephrin B2	Efnb2	NA				NA		0.1742471
mPool7_V2MM_116116	1.727206	1.959154	TRUE	HP_337071	TGCTGTTG/	CTACAATA/	XM_138757	Mus musculus similar to ribosomal protein L7a, cytosolic [validated] - rat (LOC238757), r			2.9	SM2300	c		1	1.707931489
mPool2_V2MM_142526	1.726834	0	TRUE	HP_363239	TGCTGTTG/	CTTAGGAA	XM_157697	Mus musculus LOC240481 (LOC240481), mRNA.			2.5	SM2056	c		9	#NUM!
mPool2_V2MM_142526	1.726834	0	TRUE	HP_363239	TGCTGTTG/	CTTAGGAA	XM_157697	Mus musculus LOC240481 (LOC240481), mR.			2.5	SM2056	c		9	#NUM!
mPool4_V2MM_206874	1.726041	59.99801	TRUE	HP_424691	TGCTGTTG/	CGCACCAG	XM_155817	Mus musculus LOC239491 (LOC239491), mRNA.			2.16	SM2628	h		10	0.221863149
092308m1_V2MM_70012	1.725899	3.004939	TRUE	HP_291933	TGCTGTTG/	GTGCCATT(	XM_358362	protein tyrosine phosphatase, receptor type Z, polypeptide 1	Ptprz1		2.3	SM2018	g		1	1.522164293
mPool2_V2MM_121852	1.725074	3.004939	TRUE	HP_342727	TGCTGTTG/	GACACCTT(	XM_141896	Mus musculus similar to actin, beta [Bos taurus] (LOC212460), mRNA.			2.11	SM2372	h		6	1.522164293
mPool7_V2MM_179672	1.724805	2.205692	TRUE	HP_400146	TGCTGTTG/	AAATCTCA(	XM_288374	Mus musculus LOC331689 (LOC331689), mRNA.			2.9	SM2274	a		6	1.656455229
mPool2_V2MM_137497	1.722985	3.004939	TRUE	HP_358217	TGCTGTTG/	GAAATTAT(	XM_153221	RIKEN cD 1110038H03 gene	1110038H03Rik		2.4	SM2042	h		10	1.522164293
mPool2_V2MM_137497	1.722985	3.004939	TRUE	HP_358217	TGCTGTTG/	GAAATTAT(	XM_153221	RIKEN cDNA 1110038H03 gene	1110038H03Rik		2.4	SM2042	h		10	1.522164293
092308m3_V2MM_107546	1.722655	62.64426	TRUE	HP_328629	TGCTGTTG/	GTAGCAAA	XM_284166	RIKEN cD 5430427O21 gene	5430427O21Rik		2.1	SM2320	c		8	0.203118686
092308m3_V2MM_107546	1.722655	62.64426	TRUE	HP_328629	TGCTGTTG/	GTAGCAAA	XM_284166	RIKEN cDNA 5430427O21 gene	5430427O21Rik		2.1	SM2320	c		8	0.203118686
mPool2_V2MM_130006	1.722553	2.205692	TRUE	HP_350805	TGCTGTTG/	GAGCTAAT(	XM_146551	Mus musculus LOC209349 (LOC209349), mR.			2.6	SM2102	a		1	1.656455229
mPool2_V2MM_130006	1.722553	2.205692	TRUE	HP_350805	TGCTGTTG/	GAGCTAAT(	XM_146551	Mus musculus LOC209349 (LOC209349), mRNA.			2.6	SM2102	a		1	1.656455229
092308m1_V2MM_177760	1.721438	3.004939	TRUE	HP_398240	TGCTGTTG/	GAGAATGA	XM_489292	hypothetical protein 4933400C23			2.5	SM2087	h		7	1.522164293
092308m1_V2MM_71136	1.720414	2.205692	TRUE	HP_293030	TGCTGTTG/	CATATATA/	NM_010652	killer cell lectin-like receptor subfamily C, member 1	Klrc1		2.4	SM2021	c		5	1.656455229
mPool2_V2MM_2250	1.720029	3.004939	TRUE	HP_225873	TGCTGTTG/	CTCATTGCT	NM_025972	N-acylsphingosine amidohydrolase (acid ceramidase)-like	Asahl		2.11	SM2383	b		7	1.522164293
092308m1_V2MM_90381	1.719739	3.004939	TRUE	HP_311801	TGCTGTTG/	CACTACAT(	BC072604	DEP domain containing 5	Depdc5		2.6	SM2113	a		3	1.522164293
mPool6_V2MM_159578	1.719659	75.83547	TRUE	NA	NA	NA	NA	NA	NA	NA		NA	NA	NA		0.120127608
mPool7_V2MM_78398	1.719277	3.004939	TRUE	HP_300084	TGCTGTTG/	GACGGATA	NM_011240	RAN binding protein 2	Ranbp2		2.8	SM2240	h		6	1.522164293
mPool4_V2MM_21305	1.716725	60.13437	TRUE	HP_244480	TGCTGTTG/	CAGCGAGG	NM_173070	small proline-rich protein 4	Sprr4		2.16	SM2619	a		2	0.220877236
092308m1_V2MM_179320	1.716666	3.004939	TRUE	HP_399794	TGCTGTTG/	GAATTTATI	XM_288311	gene model 1550, (NCBI)	Gm1550		2.5	SM2080	e		5	1.522164293
092308m1_V2MM_179320	1.716666	3.004939	TRUE	HP_399794	TGCTGTTG/	GAATTTATI	XM_288311	gene model 1550, (NCBI)	Gm1550		2.5	SM2080	e		5	1.522164293
mPool2_V2MM_214253	1.716632	3.004939	TRUE	HP_431854	TGCTGTTG/	CAATTATA/	XM_140452	Mus musculus LOC240195 (LOC240195), mRNA.			2.11	SM2369	a		7	1.522164293
mPool7_V2MM_104337	1.714827	3.004939	TRUE	HP_325471	TGCTGTTG/	GAGAGAAT	XM_129704	potassium voltage-gated channel, subfamily Q, member 5	Kcnq5		2.7	SM2153	h		9	1.522164293
092308m1_V2MM_77656	1.714723	3.004939	TRUE	HP_299371	TGCTGTTG/	CTGTTTAA(	AK004695	vacuolar protein sorting 11 (yeast)	Vps11		2.3	SM2001	f		9	1.522164293
mPool7_V2MM_199967	1.714685	2.205692	TRUE	HP_418004	TGCTGTTG/	CTGAGGAT(	XM_289070	Mus musculus LOC333520 (LOC333520), mRNA.			2.9	SM2289	h		4	1.656455229
092308m1_V2MM_177436	1.714304	2.205692	TRUE	HP_397916	TGCTGTTG/	CATGTATT	XM_287874	Mus musculus hypothetical gene supported by AK084195 (LOC330879), mR.			2.5	SM2071	h		8	1.656455229
mPool7_V2MM_136545	1.71387	3.004939	TRUE	HP_357265	TGCTGTTG/	GAGTTATC	XM_151819	Mus musculus LOC213261 (LOC213261), mRNA.			2.1	SM2332	a		10	1.522164293
mPool2_V2MM_111896	1.712256	2.205692	TRUE	HP_332903	TGCTGTTG/	CGAGGTTC/	XM_136234	Mus musculus similar to Rbm6 protein (LOC240782), mRNA.			2.7	SM2154	h		10	1.656455229
mPool7_V2MM_72556	1.711047	2.205692	TRUE	HP_294412	TGCTGTTG/	CTGTTATAT	XM_283218	kinesin family member 13B	Kif13b		2.8	SM2229	h		7	1.656455229
mPool2_V2MM_1143	1.710174	2.205692	TRUE	HP_224793	TGCTGTTG/	CATATTCA(	NM_027216	solute carrier family 39 (metal ion transporter), member 11	Slc39a11		2.11	SM2380	d		6	1.656455229
092308m1_V2MM_176265	1.710005	1.959154	TRUE	HP_396747	TGCTGTTG/	GAGCTATT(	AK028992	RIKEN cD 6230400G14 gene	6230400G14Rik		2.5	SM2076	c		11	1.707931489
092308m1_V2MM_179579	1.709722	2.205692	TRUE	HP_400053	TGCTGTTG/	GAGTATCA/	XM_288357	Mus musculus LOC331677 (LOC331677), mRNA.			2.5	SM2087	g		7	1.656455229
092308m1_V2MM_179579	1.709722	2.205692	TRUE	HP_400053	TGCTGTTG/	GAGTATCA/	XM_288357	Mus musculus LOC331677 (LOC331677), mR.			2.5	SM2087	g		7	1.656455229
mPool2_V2MM_110362	1.709357	2.205692	TRUE	HP_331398	TGCTGTTG/	CTGAGTTT(	XM_134619	matrix metalloproteinase 27	Mmp27		2.1	SM2323	d		9	1.656455229
mPool2_V2MM_39693	1.70718	3.004939	TRUE	HP_262376	TGCTGTTG/	GGCAGAAA	NM_028139	ataxin 7-like 4	Atxn7l4		2.11	SM2390	c		12	1.522164293
mPool2_V2MM_21683	1.705785	2.205692	TRUE	HP_244854	TGCTGTTG/	CATGTTAA1	NM_030712	chemokine (C-X-C motif) receptor 6	Cxcr6		2.11	SM2391	a		8	1.656455229
092308m1_V2MM_160811	1.704706	1.959154	TRUE	HP_381389	TGCTGTTG/	CATATTAT(	AK040883	leucine-rich repeat-containing G protein-coupled receptor 6	Lgr6		2.6	SM2125	e		10	1.707931489
mPool2_V2MM_126032	1.703954	3.004939	TRUE	HP_346875	TGCTGTTG/	CAGCCACA/	XM_144331	Mus musculus similar to Adaptor protein complex AP-2, alpha 2 subunit (LOC242987), n			2.1	SM2349	c		9	1.522164293
092308m1_V2MM_172832	1.703612	3.004939	TRUE	HP_393318	TGCTGTTG/	GATTAAATI	XM_286907	Mus musculus hypothetical gene supported by AK048737 (LOC328947), mRNA.			2.5	SM2089	f		8	1.522164293
092308m1_V2MM_172832	1.703612	3.004939	TRUE	HP_393318	TGCTGTTG/	GATTAAATI	XM_286907	Mus musculus hypothetical gene supported by AK048737 (LOC328947), mR.			2.5	SM2089	f		8	1.522164293
mPool2_V2MM_131554	1.703386	3.004939	TRUE	HP_352316	TGCTGTTG/	CCCATGAA(	BC013539	immunoglobulin heavy chain (J558 family)	Igh-VJ558		2.11	SM2360	f		7	1.522164293
mPool2_V2MM_136416	1.702517	2.205692	TRUE	HP_357136	TGCTGTTG/	CTGATACC/	XM_151707	Mus musculus LOC237268 (LOC237268), mRNA.			2.5	SM2054	d		3	1.656455229
mPool2_V2MM_136416	1.702517	2.205692	TRUE	HP_357136	TGCTGTTG/	CTGATACC/	XM_151707	Mus musculus LOC237268 (LOC237268), mR.			2.5	SM2054	d		3	1.656455229
092308m1_V2MM_141774	1.701996	3.004939	TRUE	HP_362487	TGCTGTTG/	CTTCAATA/	XM_156976	Mus musculus LOC210827 (LOC210827), mR.			2.4	SM2049	b		6	1.522164293
092308m1_V2MM_141774	1.701996	3.004939	TRUE	HP_362487	TGCTGTTG/	CTTCAATA/	XM_156976	Mus musculus LOC210827 (LOC210827), mRNA.			2.4	SM2049	b		6	1.522164293
mPool2_V2MM_128057	1.700216	3.004939	TRUE	HP_348873	TGCTGTTG/	CACTAGTC(	XM_145511	heat shock protein, alpha-crystallin-related, B6	Hspb6		2.11	SM2373	a		3	1.522164293

mPool7_V2MM_78595	1.699674	3.004939	TRUE	HP_300276	TGCTGTTG/	GAGACAGA	NM_013709	Sh3 domain YSC-like 1	Sh3yl1		2.8	SM2233	c		4	1.522164293
092308m3_V2MM_59009	1.69903	75.83547	TRUE	HP_281220	TGCTGTTG/	GCAGTCAG	NM_028561	spermatogenesis associated glutamate (E)-rich protein 4b	Speer4b		2.12	SM2436	c		10	0.120127608
mPool2_V2MM_214957	1.698929	3.004939	TRUE	HP_432527	TGCTGTTG/	CCAAGAAA	XM_141921	gene model 370, (NCBI)	Gm370		2.11	SM2359	c		4	1.522164293
mPool2_V2MM_193324	1.697744	3.004939	TRUE	HP_329142	TGCTGTTG/	GGACCAAA	XM_132579	PEST-containing nuclear protein			2.1	SM2318	c		10	1.522164293
mPool2_V2MM_193324	1.697744	3.004939	TRUE	HP_329142	TGCTGTTG/	GGACCAAA	XM_132579	PEST-containing nuclear protein			2.1	SM2318	c		10	1.522164293
092308m3_V2MM_48710	1.697484	59.99801	TRUE	HP_271167	TGCTGTTG/	GCCAGTTG	NM_177337	ADP-ribosylation factor-like 11	Arl11		2.12	SM2446	g		2	0.221863149
mPool7_V2MM_71767	1.69744	2.205692	TRUE	HP_293639	TGCTGTTG/	CTATTTATA	XM_139060	similar to TRAV3-1			2.8	SM2230	d		5	1.656455229
mPool2_V2MM_125590	1.697176	2.205692	TRUE	HP_346433	TGCTGTTG/	GGGACCAT	XM_144109	gene model 435, (NCBI)	Gm435		2.11	SM2369	c		3	1.656455229
mPool7_V2MM_108133	1.697088	3.004939	TRUE	HP_329206	TGCTGTTG/	GCTGGAAT	AK036732	hypothetical protein 9830168K20			2.1	SM2306	a		11	1.522164293
mPool6_V2MM_159050	1.694999	69.72441	TRUE	HP_379652	TGCTGTTG/	CCAGCAGA	XM_282962	Mus musculus hypothetical gene supported by AK029003 (LOC327858), mRNA.			2.7	SM2195	h		9	0.156615137
mPool2_V2MM_113276	1.694849	2.205692	TRUE	HP_334263	TGCTGTTG/	CACAGTAT	XM_136902	Mus musculus similar to hypothetical protein A830058L05 [Mus musculus] (LOC241217			2.7	SM2152	f		4	1.656455229
mPool2_V2MM_13348	1.694825	2.205692	TRUE	HP_236718	TGCTGTTG/	CAGTTATT	NM_010738	lymphocyte antigen 6 complex, locus A	Ly6a		2.11	SM2379	b		5	1.656455229
mPool4_V2MM_220434	1.69329	75.83547	TRUE	HP_453398	TGCTGTTG/	CTGTGAGT	NM_028737	RIKEN cDNA 4931406B18 gene	4931406B18Rik		2.15	SM2590	f		10	0.120127608
mPool2_V2MM_230	1.692944	2.205692	TRUE	HP_223905	TGCTGTTG/	CAATTAAT	NM_010823	myeloproliferative leukemia virus oncogene	Mpl		2.11	SM2383	h		3	1.656455229
092308m1_V2MM_138667	1.692732	3.004939	TRUE	HP_359387	TGCTGTTG/	GAGCACTC	XM_154403	Mus musculus LOC238812 (LOC238812), mR.			2.5	SM2056	b		6	1.522164293
092308m1_V2MM_138667	1.692732	3.004939	TRUE	HP_359387	TGCTGTTG/	GAGCACTC	XM_154403	Mus musculus LOC238812 (LOC238812), mRNA.			2.5	SM2056	b		6	1.522164293
mPool7_V2MM_181334	1.692111	3.004939	TRUE	HP_401808	TGCTGTTG/	CCTATTACC	XM_288658	Mus musculus LOC328501 (LOC328501), mR.			2.5	SM2085	d		11	1.522164293
mPool7_V2MM_181334	1.692111	3.004939	TRUE	HP_401808	TGCTGTTG/	CCTATTACC	XM_288658	Mus musculus LOC328501 (LOC328501), mRNA.			2.5	SM2085	d		11	1.522164293
mPool6_V2MM_169583	1.691789	75.83547	TRUE	HP_390078	TGCTGTTG/	CAGGGAAG	AK081028	hypothetical protein B930071A02			2.8	SM2213	a		8	0.120127608
mPool7_V2MM_179579	1.691606	2.205692	TRUE	HP_400053	TGCTGTTG/	GAGTATCA	XM_288357	Mus musculus LOC331677 (LOC331677), mRNA.			2.5	SM2087	g		7	1.656455229
mPool7_V2MM_179579	1.691606	2.205692	TRUE	HP_400053	TGCTGTTG/	GAGTATCA	XM_288357	Mus musculus LOC331677 (LOC331677), mR.			2.5	SM2087	g		7	1.656455229
mPool2_V2MM_107243	1.6913	1.959154	TRUE	HP_328332	TGCTGTTG/	CCTGTAAT	AK122544	DNA segment, Chr 5, Wayne State University 178, expressed	D5Wsu178e		2.1	SM2319	f		1	1.707931489
mPool2_V2MM_111848	1.690387	2.205692	TRUE	HP_332855	TGCTGTTG/	CAGCTATG	XM_136205	G protein-coupled receptor 39	Gpr39		2.1	SM2320	d		7	1.656455229
mPool2_V2MM_123198	1.690271	3.004939	TRUE	HP_344054	TGCTGTTG/	CAGAATCC	XM_142755	similar to hypothetical protein FLJ90396			2.11	SM2355	a		8	1.522164293
mPool7_V2MM_172684	1.687238	2.205692	TRUE	HP_393170	TGCTGTTG/	CTAGAGAC	XM_286882	Mus musculus hypothetical gene supported by AK085983 (LOC328888), mRNA.			2.9	SM2282	b		5	1.656455229
mPool7_V2MM_173731	1.686504	3.004939	TRUE	HP_394217	TGCTGTTG/	CTTAATTGT	XM_488936	LOC433405			2.9	SM2273	c		8	1.522164293
mPool2_V2MM_212465	1.686325	3.004939	TRUE	HP_430131	TGCTGTTG/	CAGCTTTG	NM_207632	olfactory receptor 1118	Olfr1118		2.11	SM2355	a		5	1.522164293
mPool7_V2MM_89292	1.683966	3.004939	TRUE	HP_310722	TGCTGTTG/	GTGGATAT	NM_177564	cDNA sequence BC022224	BC022224		2.9	SM2259	c		2	1.522164293
092308m1_V2MM_136416	1.683669	3.004939	TRUE	HP_357136	TGCTGTTG/	CTGATACC	XM_151707	Mus musculus LOC237268 (LOC237268), mR.			2.5	SM2054	d		3	1.522164293
092308m1_V2MM_136416	1.683669	3.004939	TRUE	HP_357136	TGCTGTTG/	CTGATACC	XM_151707	Mus musculus LOC237268 (LOC237268), mRNA.			2.5	SM2054	d		3	1.522164293
mPool2_V2MM_207912	1.683554	3.004939	TRUE	HP_425709	TGCTGTTG/	CATCTTCCT	XM_195479	Mus musculus similar to argonaute 5 protein [Mus musculus] (LOC269496), mRNA.			2.1	SM2335	b		8	1.522164293
mPool5_V2MM_160937	1.680462	75.83547	TRUE	HP_381514	TGCTGTTG/	GCCGCAAG	XM_486014	similar to zinc finger and BTB domain containing 2		NA				NA		0.120127608
mPool4_V2MM_114976	1.680189	75.83547	TRUE	HP_335949	TGCTGTTG/	CTTTGGATT	XM_138051	Mus musculus similar to Heterogeneous nuclear ribonucleoprotein A1 (Helix-destabilizi			2.16	SM2626	d		6	0.120127608
092308m1_V2MM_164058	1.67844	3.004939	TRUE	HP_384608	TGCTGTTG/	GCATGTAA	XM_486406	similar to godotropin inducible ovarian transcription factor 1			2.6	SM2120	e		9	1.522164293
mPool2_V2MM_144690	1.676626	3.004939	TRUE	HP_365401	TGCTGTTG/	GCTATGGA	XM_160200	Mus musculus LOC213229 (LOC213229), mRNA.			2.1	SM2334	g		2	1.522164293
mPool2_V2MM_125824	1.676015	3.004939	TRUE	HP_346667	TGCTGTTG/	CAAATAGA	XM_144242	heterogeneous nuclear ribonucleoprotein A3	Hnrpa3		2.11	SM2354	e		8	1.522164293
mPool7_V2MM_152662	1.675901	3.004939	TRUE	HP_373324	TGCTGTTG/	CCAAGAAG	XM_195495	Mus musculus similar to ribosomal protein S17 [Mus musculus] (LOC269534), mRNA.			2.1	SM2329	c		3	1.522164293
mPool2_V2MM_213282	1.675459	3.004939	TRUE	HP_430922	TGCTGTTG/	GGATTATT	XM_141458	Mus musculus similar to olfactory receptor MOR245-10 [Mus musculus] (LOC228435), n			2.11	SM2362	a		4	1.522164293
mPool2_V2MM_211088	1.674746	3.004939	TRUE	HP_428814	TGCTGTTG/	GACTGAAG	XM_147017	similar to Chromosome 6 open reading frame 117			2.1	SM2348	f		1	1.522164293
092308m1_V2MM_149887	1.67447	3.004939	TRUE	HP_370597	TGCTGTTG/	GTTTAGTTA	XM_165303	Mus musculus LOC208460 (LOC208460), mR.			2.4	SM2049	a		6	1.522164293
mPool2_V2MM_103007	1.673055	1.959154	TRUE	HP_324152	TGCTGTTG/	GAACTGAA	XM_128751	cysteine-rich motor neuron 1	Crim1		2.1	SM2311	c		3	1.707931489
mPool7_V2MM_141500	1.672061	3.004939	TRUE	HP_362213	TGCTGTTG/	ATCCCAAG	XM_156723	Mus musculus LOC210877 (LOC210877), mR.			2.5	SM2057	d		7	1.522164293
mPool7_V2MM_141500	1.672061	3.004939	TRUE	HP_362213	TGCTGTTG/	ATCCCAAG	XM_156723	Mus musculus LOC210877 (LOC210877), mRNA.			2.5	SM2057	d		7	1.522164293
092308m1_V2MM_132242	1.671695	2.205692	TRUE	HP_352996	TGCTGTTG/	CCTCCTAAT	AK080331	RIKEN cD A630055G03 gene	A630055G03Rik		2.6	SM2111	b		9	1.656455229
092308m1_V2MM_63607	1.670955	3.004939	TRUE	HP_285683	TGCTGTTG/	CTAAGCAA	NM_029682	RIKEN cD 1700095N21 gene	1700095N21Rik		2.3	SM2013	c		5	1.522164293
092308m1_V2MM_123913	1.669969	3.004939	TRUE	HP_344768	TGCTGTTG/	GACTACAG	XM_143206	Mus musculus similar to zo pellucida protein 1 [Gallus gallus] (LOC215202), mR.			2.5	SM2099	c		1	1.522164293
mPool7_V2MM_81409	1.669353	3.004939	TRUE	HP_303015	TGCTGTTG/	GTATTTATG	NM_172859	RIKEN cDNA 2810039F03 gene	2810039F03Rik		2.8	SM2231	a		4	1.522164293
092308m1_V2MM_171812	1.66744	2.205692	TRUE	HP_392299	TGCTGTTG/	CTGCTTATT	XM_286699	Mus musculus hypothetical gene supported by AK029886 (LOC328542), mR.			2.5	SM2073	h		7	1.656455229
mPool2_V2MM_95470	1.667251	3.004939	TRUE	HP_316746	TGCTGTTG/	CCGTCTGT	NM_198010	ankyrin repeat domain 17	Ankrd17		2.6	SM2134	g		5	1.522164293
mPool7_V2MM_84826	1.665726	3.004939	TRUE	HP_306326	TGCTGTTG/	CTGGGACT	NM_029545	RIKEN cDNA 6530401N04 gene	6530401N04Rik		2.9	SM2258	d		12	1.522164293
mPool7_V2MM_195814	1.665548	1.959154	TRUE	HP_413937	TGCTGTTG/	GGGCATTG	NM_175270	RIKEN cDNA 5730467H21 gene	5730467H21Rik		2.9	SM2251	f		3	1.707931489
092308m1_V2MM_181041	1.665303	2.205692	TRUE	HP_401515	TGCTGTTG/	GACATTATT	XM_288609	Mus musculus LOC332013 (LOC332013), mR.			2.5	SM2062	c		10	1.656455229
092308m1_V2MM_181041	1.665303	2.205692	TRUE	HP_401515	TGCTGTTG/	GACATTATT	XM_288609	Mus musculus LOC332013 (LOC332013), mRNA.			2.5	SM2062	c		10	1.656455229
092308m1_V2MM_150691	1.664663	2.205692	TRUE	HP_371379	TGCTGTTG/	GTAGGACA	NM_177857	RIKEN cD A930010I20 gene	A930010I20Rik		2.4	SM2041	g		4	1.656455229
mPool2_V2MM_112258	1.664356	2.205692	TRUE	HP_333263	TGCTGTTG/	GGCCATTT	XM_488159	similar to protease			2.7	SM2157	b		10	1.656455229
092308m3_V2MM_83872	1.662958	35.99881	TRUE	HP_305394	TGCTGTTG/	CTGAGTAA	NM_025890	RIKEN cD 2410004A20 gene	2410004A20Rik		2.13	SM2458	g		4	0.443711899
092308m3_V2MM_25446	1.662316	59.99801	TRUE	HP_248513	TGCTGTTG/	CAGCCAGA	NM_172411	RIKEN cD 2310007B03 gene	2310007B03Rik		2.12	SM2427	g		12	0.221863149
mPool7_V2MM_95902	1.660192	1.959154	TRUE	NA	NA	NA	NA	NA	NA	NA		NA	NA	NA		1.707931489
092308m1_V2MM_154894	1.65988	3.004939	TRUE	HP_375535	TGCTGTTG/	CCATGACA	XM_197074	Mus musculus LOC271145 (LOC271145), mR.			2.6	SM2122	h		2	1.522164293
mPool2_V2MM_214331	1.659023	2.205692	TRUE	HP_431929	TGCTGTTG/	GAAAGTTC	XM_147679	Mus musculus LOC209126 (LOC209126), mRNA.			2.11	SM2365	c		8	1.656455229
092308m3_V2MM_59041	1.657501	61.29828	TRUE	HP_281250	TGCTGTTG/	GCATCCAG	NM_177030	RIKEN cD C330023D02 gene	C330023D02Rik		2.12	SM2416	g		6	0.212551731
mPool2_V2MM_118588	1.657123	3.004939	TRUE	HP_339522	TGCTGTTG/	GATGAGGT	XM_139952	Mus musculus similar to olfactory receptor MOR121-1 [Mus musculus] (LOC209762), m			2.11	SM2355	a		3	1.522164293

mPool2_V2MM_97315	1.65693	3.004939	TRUE	HP_318557	TGCTGTTG	GATCAAGT	XM_111430	Mus musculus similar to serine protease inhibitor 10 [Mus musculus] (LOC193433), mRN		2.6	SM2107	d	4	1.522164293
mPool2_V2MM_97315	1.65693	3.004939	TRUE	HP_318557	TGCTGTTG	GATCAAGT	XM_111430	Mus musculus similar to serine protease inhibitor 10 [Mus musculus] (LOC193433), mR.		2.6	SM2107	d	4	1.522164293
mPool2_V2MM_101471	1.65571	3.004939	TRUE	HP_322642	TGCTGTTG	GCTCTGTTC	AK044563	DNA segment, Chr 14, Abbott 1 expressed	D14Abb1e	2.7	SM2153	b	4	1.522164293
mPool2_V2MM_115134	1.654911	3.004939	TRUE	HP_336104	TGCTGTTG	CATGAGTT	XM_138128	Mus musculus similar to Embryonic growth-associated (LOC217630), mRNA.		2.6	SM2149	c	2	1.522164293
092308m3_V2MM_49151	1.65458	62.64426	TRUE	HP_271597	TGCTGTTG	GGCAAAGG	NM_172524	RIKEN cD 9530066K23 gene	9530066K23Rik	2.12	SM2416	a	4	0.203118686
mPool2_V2MM_104352	1.654459	3.004939	TRUE	HP_325486	TGCTGTTG	CCTATTGCC	AK077997	RIKEN cDNA 6130401J04 gene	6130401J04Rik	2.7	SM2157	e	2	1.522164293
mPool7_V2MM_64588	1.654208	2.205692	TRUE	HP_286638	TGCTGTTG	CTGTGTTT	NM_026367	G patch domain containing 2	Gpatc2	2.8	SM2232	e	10	1.656455229
mPool6_V2MM_161630	1.653826	60.13437	TRUE	HP_382194	TGCTGTTG	CCCTGATT	XM_283985	Mus musculus hypothetical gene supported by AK089333 (LOC329899), mRNA.		2.8	SM2209	d	9	0.220877236
092308m1_V2MM_62958	1.653809	3.004939	TRUE	HP_285050	TGCTGTTG	CCAGTAAA	NM_011316	serum amyloid A 4	Saa4	2.4	SM2020	g	8	1.522164293
092308m1_V2MM_87165	1.65377	2.205692	TRUE	HP_308624	TGCTGTTG	CTGGTTAG	NM_175099	replication initiator 1	Repin1	2.6	SM2108	f	11	1.656455229
mPool7_V2MM_187884	1.653066	1.959154	TRUE	HP_408348	TGCTGTTG	CATGGACA	XM_489130	hypothetical gene supported by AK018238; AK046289		2.5	SM2076	h	2	1.707931489
mPool7_V2MM_187884	1.653066	1.959154	TRUE	HP_408348	TGCTGTTG	CATGGACA	XM_489130	hypothetical gene supported by AK018238; AK046289		2.5	SM2076	h	2	1.707931489
mPool2_V2MM_149064	1.652872	2.205692	TRUE	HP_369775	TGCTGTTG	CCATGATA	XM_164446	Mus musculus LOC244637 (LOC244637), mRNA.		2.1	SM2344	c	9	1.656455229
092308m3_V2MM_237848	1.652659	60.13437	TRUE	HP_489691	TGCTGTTG	CTATGAGC	XM_154800	Mus musculus LOC239116 (LOC239116), mR.		2.14	SM2528	d	5	0.220877236
mPool7_V2MM_84788	1.652556	2.205692	TRUE	HP_306291	TGCTGTTG	CCTCCATTC	NM_029282	RIKEN cDNA 2610036D13 gene	2610036D13Rik	2.9	SM2256	g	1	1.656455229
092308m1_V2MM_141969	1.651983	3.004939	TRUE	HP_362682	TGCTGTTG	CCTGGAAT	XM_157241	Mus musculus LOC240232 (LOC240232), mR.		2.5	SM2052	h	11	1.522164293
mPool2_V2MM_19547	1.651104	3.004939	TRUE	HP_242763	TGCTGTTG	GGTGTAAA	XM_133269	Mus musculus similar to Glycogen synthase kinase-3 alpha (GSK-3 alpha) (Factor A) (FA)		2.11	SM2376	g	4	1.522164293
mPool7_V2MM_92122	1.650871	3.004939	TRUE	HP_313485	TGCTGTTG	CATTGAAT	NM_178653	RIKEN cD C330023F11 gene	C330023F11Rik	2.9	SM2263	g	9	1.522164293
mPool7_V2MM_92122	1.650871	3.004939	TRUE	HP_313485	TGCTGTTG	CATTGAAT	NM_178653	RIKEN cDNA C330023F11 gene	C330023F11Rik	2.9	SM2263	g	9	1.522164293
mPool7_V2MM_62581	1.650682	3.004939	TRUE	HP_284686	TGCTGTTG	CAGGTAGT	NM_146936	olfactory receptor 1417	Olfr1417	2.8	SM2242	c	7	1.522164293
092308m1_V2MM_123721	1.650348	3.004939	TRUE	HP_344577	TGCTGTTG	CTTTATCGA	XM_143117	Mus musculus similar to peptidylprolyl isomerase D (cyclophilin D); hCyP40 [Homo sapie		2.6	SM2114	g	3	1.522164293
mPool7_V2MM_81341	1.649455	2.205692	TRUE	HP_302948	TGCTGTTG	GTACGATA	NM_021704	chemokine (C-X-C motif) ligand 12	Cxcl12	2.8	SM2243	h	11	1.656455229
mPool7_V2MM_184802	1.648989	3.004939	TRUE	HP_405274	TGCTGTTG	CGGTTAAT	XM_289261	Mus musculus LOC332907 (LOC332907), mRNA.		2.5	SM2086	c	5	1.522164293
mPool7_V2MM_184802	1.648989	3.004939	TRUE	HP_405274	TGCTGTTG	CGGTTAAT	XM_289261	Mus musculus LOC332907 (LOC332907), mR.		2.5	SM2086	c	5	1.522164293
mPool7_V2MM_116331	1.648744	2.205692	TRUE	HP_337286	TGCTGTTG	GAGTCTAG	XM_138876	gene model 280, (NCBI)	Gm280	2.1	SM2302	e	3	1.656455229
092308m1_V2MM_165864	1.647286	3.004939	TRUE	HP_386398	TGCTGTTG	GTCAATTC	XM_285342	cardiomyopathy associated 3	Cmya3	2.6	SM2125	a	8	1.522164293
mPool7_V2MM_172357	1.644231	2.205692	TRUE	HP_392843	TGCTGTTG	CCATGGTG	AK051520	hypothetical protein D130054H01		2.5	SM2095	c	6	1.656455229
mPool7_V2MM_172357	1.644231	2.205692	TRUE	HP_392843	TGCTGTTG	CCATGGTG	AK051520	hypothetical protein D130054H01		2.5	SM2095	c	6	1.656455229
092308m3_V2MM_238015	1.643452	75.83547	TRUE	HP_489856	TGCTGTTG	GCCTCTGC	XM_158288	Mus musculus LOC241355 (LOC241355), mR.		2.14	SM2517	g	5	0.120127608
092308m3_V2MM_112866	1.643403	66.95036	TRUE	HP_333858	TGCTGTTG	GCTGTCGA	AK122502	cD sequence BC056494	BC056494	2.13	SM2486	h	2	0.1742471
mPool6_V2MM_68227	1.642929	75.83547	TRUE	HP_290192	TGCTGTTG	CTGTGTGT	NM_026100	RIKEN cDNA 1700055O19 gene	1700055O19Rik	2.7	SM2177	b	11	0.120127608
092308m1_V2MM_187364	1.642089	3.004939	TRUE	HP_407833	TGCTGTTG	GGGAAATC	XM_289743	Mus musculus LOC333651 (LOC333651), mR.		2.5	SM2080	b	9	1.522164293
mPool2_V2MM_213434	1.640589	2.205692	TRUE	HP_431065	TGCTGTTG	CGGGACCA	XM_144109	gene model 435, (NCBI)	Gm435	2.11	SM2362	e	1	1.656455229
mPool7_V2MM_67030	1.640248	3.004939	TRUE	HP_289030	TGCTGTTG	CCCATTACA	NM_010484	solute carrier family 6 (neurotransmitter transporter, seroton	Slc6a4	2.8	SM2233	g	1	1.522164293
mPool6_V2MM_160680	1.638854	69.72441	TRUE	HP_381259	TGCTGTTG	GAGCTCAT	NM_010678	lymphoid nuclear protein related to AF4	Laf4	2.8	SM2207	c	9	0.156615137
092308m1_V2MM_186219	1.638758	3.004939	TRUE	HP_406691	TGCTGTTG	CAGAGAAT	XM_289502	Mus musculus LOC333219 (LOC333219), mRNA.		2.5	SM2066	d	12	1.522164293
092308m1_V2MM_186219	1.638758	3.004939	TRUE	HP_406691	TGCTGTTG	CAGAGAAT	XM_289502	Mus musculus LOC333219 (LOC333219), mR.		2.5	SM2066	d	12	1.522164293
mPool2_V2MM_214049	1.635909	3.004939	TRUE	HP_431663	TGCTGTTG	GAATTTAT	XM_143381	Mus musculus similar to 60S acidic ribosomal protein P1 (LOC229548), mRNA.		2.11	SM2354	g	3	1.522164293
092308m1_V2MM_146848	1.635419	3.004939	TRUE	HP_367559	TGCTGTTG	CGGGTAAC	XM_162361	Mus musculus LOC210681 (LOC210681), mR.		2.4	SM2045	b	3	1.522164293
092308m1_V2MM_146848	1.635419	3.004939	TRUE	HP_367559	TGCTGTTG	CGGGTAAC	XM_162361	Mus musculus LOC210681 (LOC210681), mR.		2.4	SM2045	b	3	1.522164293
mPool2_V2MM_128478	1.635243	2.205692	TRUE	HP_349292	TGCTGTTG	GTCATAGA	XM_145695	Mus musculus similar to glyceraldehyde-3-phosphate dehydrogenase [Mus musculus] (l		2.11	SM2364	a	5	1.656455229
mPool2_V2MM_30073	1.634736	3.004939	TRUE	HP_253013	TGCTGTTG	CAGTACAT	NM_148948	Dicer1, Dcr-1 homolog (Drosophila)	Dicer1	2.11	SM2391	b	5	1.522164293
092308m3_V2MM_48109	1.633919	75.83547	TRUE	HP_270573	TGCTGTTG	GACTGGAG	NM_016784	pleiotropic regulator 1, PRL1 homolog (Arabidopsis)	Plrg1	2.12	SM2437	d	2	0.120127608
mPool2_V2MM_12429	1.631928	2.205692	TRUE	HP_235822	TGCTGTTG	CAAATATT	NM_007550	Bloom syndrome homolog (human)	Blm	2.11	SM2383	c	5	1.656455229
092308m3_V2MM_104592	1.631834	73.80895	TRUE	HP_325724	TGCTGTTG	CTGACAGA	NM_198899	UDP-glucose ceramide glucosyltransferase-like 1	Ugcgl1	2.9	SM2296	d	12	0.131891001
092308m3_V2MM_104592	1.631834	73.80895	TRUE	HP_325724	TGCTGTTG	CTGACAGA	NM_198899	UDP-glucose ceramide glucosyltransferase-like 1	Ugcgl1	2.9	SM2296	d	12	0.131891001
mPool2_V2MM_123892	1.631352	3.004939	TRUE	HP_344747	TGCTGTTG	CGAGTTCA	XM_143201	similar to Polyadenylate-binding protein 4 (Poly(A)-binding protein 4) (PABP 4) (Inducibl		2.11	SM2361	g	7	1.522164293
092308m1_V2MM_64758	1.629823	3.004939	TRUE	HP_286808	TGCTGTTG	CTTGTAAC	NM_026202	RIKEN cD 2610529H08 gene	2610529H08Rik	2.3	SM2007	h	1	1.522164293
092308m1_V2MM_185449	1.62962	2.205692	TRUE	HP_405921	TGCTGTTG	GACCTTTC	XM_289372	SEC31-like 1 (S. cerevisiae)	Sec31l1	2.5	SM2094	b	3	1.656455229
092308m1_V2MM_170776	1.629174	3.004939	TRUE	HP_391266	TGCTGTTG	CAATCTTAC	AK049051	engulfment and cell motility 1, ced-12 homolog (C. elegans)	Elmo1	2.5	SM2069	c	4	1.522164293
092308m1_V2MM_170776	1.629174	3.004939	TRUE	HP_391266	TGCTGTTG	CAATCTTAC	AK049051	engulfment and cell motility 1, ced-12 homolog (C. elegans)	Elmo1	2.5	SM2069	c	4	1.522164293
092308m1_V2MM_182147	1.62846	3.004939	TRUE	HP_402621	TGCTGTTG	GGTGTACA	XM_288795	Mus musculus LOC332264 (LOC332264), mRNA.		2.5	SM2095	a	12	1.522164293
092308m1_V2MM_182147	1.62846	3.004939	TRUE	HP_402621	TGCTGTTG	GGTGTACA	XM_288795	Mus musculus LOC332264 (LOC332264), mR.		2.5	SM2095	a	12	1.522164293
mPool7_V2MM_64772	1.627981	2.205692	TRUE	HP_286822	TGCTGTTG	CTTGTTATT	NM_007484	ras homolog gene family, member C	Rhoc	2.8	SM2234	a	12	1.656455229
mPool7_V2MM_88549	1.627651	2.205692	TRUE	HP_309986	TGCTGTTG	GTCTGTAA	NM_175431	RIKEN cDNA A830093I24 gene	A830093I24Rik	2.9	SM2262	c	4	1.656455229
mPool7_V2MM_90928	1.626334	3.004939	TRUE	HP_312344	TGCTGTTG	CTGCTAAG	NM_177888	zinc finger protein 78	Zfp78	2.9	SM2255	g	8	1.522164293
mPool7_V2MM_90928	1.626334	3.004939	TRUE	HP_312344	TGCTGTTG	CTGCTAAG	NM_177888	zinc finger protein 78	Zfp78	2.9	SM2255	g	8	1.522164293
mPool2_V2MM_117781	1.626197	3.004939	TRUE	HP_338721	TGCTGTTG	CAGAGGTT	XM_139533	RIKEN cDNA A630029G22 gene	A630029G22Rik	2.11	SM2369	f	8	1.522164293
mPool2_V2MM_202297	1.625141	3.004939	TRUE	HP_420308	TGCTGTTG	CACCTTCTA	XM_136883	Mus musculus similar to KIAA1007 protein; adrenal gland protein AD-005 [Homo sapien		2.1	SM2333	c	4	1.522164293
092308m3_V2MM_233281	1.623726	59.99801	TRUE	HP_485199	TGCTGTTG	CACAGCAA	NM_199147	cD sequence BC062185	BC062185	2.14	SM2503	f	12	0.221863149
092308m3_V2MM_208742	1.621922	75.83547	TRUE	HP_426524	TGCTGTTG	CCAACTTA	XM_154583	Mus musculus LOC238932 (LOC238932), mR.		2.14	SM2512	g	8	0.120127608
092308m1_V2MM_95911	1.62175	2.205692	TRUE	HP_317178	TGCTGTTG	CTGGTGAC	XM_109763	Mus musculus LOC192909 (LOC192909), mR.		2.6	SM2110	a	11	1.656455229

092308m1_V2MM_147408	1.618192	2.205692	TRUE	HP_368119	TGCTGTTG/	GAAACAGT	XM_162954	Mus musculus LOC243724 (LOC243724), mR.			2.5	SM2058	d		5	1.656455229
092308m1_V2MM_147408	1.618192	2.205692	TRUE	HP_368119	TGCTGTTG/	GAAACAGT	XM_162954	Mus musculus LOC243724 (LOC243724), mRNA.			2.5	SM2058	d		5	1.656455229
092308m1_V2MM_153474	1.617355	3.004939	TRUE	HP_374129	TGCTGTTG/	CCTTGGTA	AK013526	RIKEN cD 2900011G08 gene	2900011G08Rik		2.6	SM2115	b		12	1.522164293
mPool7_V2MM_62079	1.616822	2.205692	TRUE	HP_284194	TGCTGTTG/	CACCTTAA(	NM_146864	olfactory receptor 768	Olfr768		2.8	SM2236	b		8	1.656455229
mPool2_V2MM_142836	1.615709	2.205692	TRUE	HP_363549	TGCTGTTG/	GGTCCTAT	XM_157999	Mus musculus LOC213992 (LOC213992), mRNA.			2.1	SM2333	f		1	1.656455229
mPool2_V2MM_142836	1.615709	2.205692	TRUE	HP_363549	TGCTGTTG/	GGTCCTAT	XM_157999	Mus musculus LOC213992 (LOC213992), mR.			2.1	SM2333	f		1	1.656455229
092308m1_V2MM_184054	1.615425	3.004939	TRUE	HP_404528	TGCTGTTG/	GCCTAGAT(	XM_289132	Mus musculus LOC331157 (LOC331157), mR.			2.5	SM2081	b		7	1.522164293
mPool2_V2MM_121026	1.615336	3.004939	TRUE	HP_341912	TGCTGTTG/	CATTGGCC	XM_141393	Mus musculus LOC241683 (LOC241683), mRNA.			2.11	SM2361	d		2	1.522164293
mPool2_V2MM_137537	1.613534	2.205692	TRUE	HP_358257	TGCTGTTG/	CTTAAAGT(	XM_153261	similar to Rho GTPase activating protein 20			2.4	SM2046	h		8	1.656455229
mPool2_V2MM_137537	1.613534	2.205692	TRUE	HP_358257	TGCTGTTG/	CTTAAAGT(	XM_153261	similar to Rho GTPase activating protein 20			2.4	SM2046	h		8	1.656455229
mPool2_V2MM_116048	1.613069	3.004939	TRUE	HP_337003	TGCTGTTG/	GTCTGGAT(	XM_488036	similar to MrgA4 RF-amide G protein-coupled receptor			2.1	SM2319	g		10	1.522164293
mPool7_V2MM_170515	1.612718	2.205692	TRUE	HP_391008	TGCTGTTG/	GCATATTA(	AK087847	RIKEN cDNA E330029E12 gene	E330029E12Rik		2.5	SM2068	f		4	1.656455229
mPool7_V2MM_170515	1.612718	2.205692	TRUE	HP_391008	TGCTGTTG/	GCATATTA(	AK087847	RIKEN cD E330029E12 gene	E330029E12Rik		2.5	SM2068	f		4	1.656455229
mPool2_V2MM_91548	1.612562	3.004939	TRUE	NA	NA	NA	NA	NA	NA	NA		NA	NA	NA		1.522164293
mPool7_V2MM_186370	1.612237	3.004939	TRUE	HP_406842	TGCTGTTG/	GTGAAGGA	XM_289527	RIKEN cDNA A030007E19 gene	A030007E19Rik		2.9	SM2282	c		12	1.522164293
mPool2_V2MM_113507	1.611841	3.004939	TRUE	HP_334494	TGCTGTTG/	GTCTTTATC	XM_136994	gene model 229, (NCBI)	Gm229		2.7	SM2157	h		3	1.522164293
mPool2_V2MM_115503	1.611368	3.004939	TRUE	HP_336464	TGCTGTTG/	CCCTTCAA(	XM_138425	Mus musculus similar to general transcription factor IIa, 2 (12kD subunit) [Rattus norveg			2.7	SM2159	b		11	1.522164293
092308m1_V2MM_136568	1.611111	3.004939	TRUE	HP_357288	TGCTGTTG/	CCTCATCCA	XM_151865	Mus musculus LOC215140 (LOC215140), mRNA.			2.4	SM2050	b		7	1.522164293
092308m1_V2MM_136568	1.611111	3.004939	TRUE	HP_357288	TGCTGTTG/	CCTCATCCA	XM_151865	Mus musculus LOC215140 (LOC215140), mR.			2.4	SM2050	b		7	1.522164293
mPool2_V2MM_25233	1.610976	1.959154	TRUE	HP_248305	TGCTGTTG/	CACATCAA	NM_133680	RIKEN cDNA 5730414C17 gene	5730414C17Rik		2.11	SM2391	f		4	1.707931489
mPool2_V2MM_122861	1.609653	3.004939	TRUE	HP_343719	TGCTGTTG/	CATAAAGC	XM_142466	Mus musculus similar to GLE1-like, RNA export mediator; GLE1 (yeast homolog)-like, RN			2.11	SM2358	a		9	1.522164293
092308m1_V2MM_75421	1.609311	3.004939	TRUE	HP_297200	TGCTGTTG/	CCATTGGA(	NM_010424	hemochromatosis	Hfe		2.3	SM2002	h		8	1.522164293
mPool7_V2MM_181041	1.609267	1.959154	TRUE	HP_401515	TGCTGTTG/	GACATTAT	XM_288609	Mus musculus LOC332013 (LOC332013), mR.			2.5	SM2062	c		10	1.707931489
mPool7_V2MM_181041	1.609267	1.959154	TRUE	HP_401515	TGCTGTTG/	GACATTAT	XM_288609	Mus musculus LOC332013 (LOC332013), mRNA.			2.5	SM2062	c		10	1.707931489
092308m1_V2MM_179098	1.609079	3.004939	TRUE	HP_399572	TGCTGTTG/	CACATTCAT	XM_288274	Mus musculus LOC332550 (LOC332550), mR.			2.5	SM2086	d		11	1.522164293
mPool7_V2MM_206355	1.609063	3.004939	TRUE	HP_424182	TGCTGTTG/	CTGTTAAA(	AK032192	potassium inwardly-rectifying channel, subfamily J, member 1	Kcnj16		2.9	SM2298	b		7	1.522164293
mPool2_V2MM_123965	1.608449	2.205692	TRUE	HP_344818	TGCTGTTG/	CAGGAATG	XM_143234	Mus musculus similar to embryonic blastocoelar extracellular matrix protein precursor [			2.11	SM2367	f		4	1.656455229
092308m1_V2MM_173192	1.608417	2.205692	TRUE	HP_393678	TGCTGTTG/	CAGAGTAA	XM_286976	Mus musculus hypothetical gene supported by AK013638; AK030685; AK049099; NM_1			2.5	SM2084	d		9	1.656455229
092308m3_V2MM_202996	1.607836	75.83547	TRUE	HP_420973	TGCTGTTG/	CATCAGAA	NM_133738	anthrax toxin receptor 2	Antxr2		2.13	SM2497	e		7	0.120127608
mPool2_V2MM_88659	1.60698	2.205692	TRUE	HP_310096	TGCTGTTG/	CCATTTGG(	NM_175460	nicotinamide nucleotide adenylyltransferase 2	Nmnat2		2.6	SM2134	c		3	1.656455229
mPool4_V2MM_163217	1.602686	61.29828	TRUE	HP_383772	TGCTGTTG/	GTGTTCAA(	XM_284721	gene model 774, (NCBI)	Gm774		2.16	SM2649	a		8	0.212551731
mPool7_V2MM_147048	1.602061	2.205692	TRUE	HP_367759	TGCTGTTG/	CACAAAGA	XM_162569	Mus musculus LOC243509 (LOC243509), mRNA.			2.4	SM2041	e		9	1.656455229
mPool7_V2MM_147048	1.602061	2.205692	TRUE	HP_367759	TGCTGTTG/	CACAAAGA	XM_162569	Mus musculus LOC243509 (LOC243509), mR.			2.4	SM2041	e		9	1.656455229
092308m1_V2MM_151228	1.601286	2.205692	TRUE	HP_371899	TGCTGTTG/	GTGAAATA	NM_001002272	trophinin	Tro		2.4	SM2050	c		10	1.656455229
092308m1_V2MM_151228	1.601286	2.205692	TRUE	HP_371899	TGCTGTTG/	GTGAAATA	NM_001002272	trophinin	Tro		2.4	SM2050	c		10	1.656455229
092308m1_V2MM_130006	1.59975	3.004939	TRUE	HP_350805	TGCTGTTG/	GAGCTAAT(	XM_146551	Mus musculus LOC209349 (LOC209349), mR.			2.6	SM2102	a		1	1.522164293
092308m1_V2MM_130006	1.59975	3.004939	TRUE	HP_350805	TGCTGTTG/	GAGCTAAT(	XM_146551	Mus musculus LOC209349 (LOC209349), mRNA.			2.6	SM2102	a		1	1.522164293
092308m1_V2MM_84816	1.599032	3.004939	TRUE	HP_306316	TGCTGTTG/	CAGTAACT	NM_029498	zinc finger protein 198	Zfp198		2.6	SM2109	d		8	1.522164293
mPool2_V2MM_125294	1.598047	2.205692	TRUE	HP_346138	TGCTGTTG/	CACCTCCT	XM_143897	Mus musculus similar to zinc finger protein 352 (LOC242525), mRNA.			2.1	SM2349	b		5	1.656455229
mPool7_V2MM_179143	1.597158	2.205692	TRUE	HP_399617	TGCTGTTG/	GGGAGACT	XM_288281	Mus musculus LOC332557 (LOC332557), mRNA.			2.9	SM2296	e		5	1.656455229
mPool7_V2MM_186219	1.597042	3.004939	TRUE	HP_406691	TGCTGTTG/	CAGAGAAT	XM_289502	Mus musculus LOC333219 (LOC333219), mR.			2.5	SM2066	d		12	1.522164293
mPool7_V2MM_186219	1.597042	3.004939	TRUE	HP_406691	TGCTGTTG/	CAGAGAAT	XM_289502	Mus musculus LOC333219 (LOC333219), mRNA.			2.5	SM2066	d		12	1.522164293
mPool6_V2MM_66304	1.596576	61.29828	TRUE	HP_288323	TGCTGTTG/	CACCAAGA	NM_011156	prolyl endopeptidase	Prep		2.7	SM2173	g		2	0.212551731
092308m1_V2MM_132069	1.596359	3.004939	TRUE	HP_352823	TGCTGTTG/	CTGTTTGG/	BC028850	RIKEN cD 1500002B03 gene	1500002B03Rik		2.6	SM2104	h		9	1.522164293
mPool6_V2MM_159548	1.596288	59.99801	TRUE	HP_380141	TGCTGTTG/	GCCTAGGT/	AK079330	RIKEN cDNA 9630025I21 gene	9630025I21Rik		2.7	SM2200	e		1	0.221863149
mPool2_V2MM_33465	1.595741	3.004939	TRUE	HP_256317	TGCTGTTG/	CAGATGAG	NM_144895	spastic paraplegia 20, spartin (Troyer syndrome) homolog (hu	Spg20		2.11	SM2389	h		12	1.522164293
092308m1_V2MM_148147	1.595412	3.004939	TRUE	HP_368858	TGCTGTTG/	GAAGGTTC	XM_163530	Mus musculus LOC244133 (LOC244133), mRNA.			2.4	SM2040	h		1	1.522164293
092308m1_V2MM_148147	1.595412	3.004939	TRUE	HP_368858	TGCTGTTG/	GAAGGTTC	XM_163530	Mus musculus LOC244133 (LOC244133), mR.			2.4	SM2040	h		1	1.522164293
mPool7_V2MM_68331	1.595013	2.205692	TRUE	HP_290294	TGCTGTTG/	CTTTAAGTA	NM_030081	zinc finger, FYVE domain containing 20	Zfyve20		2.8	SM2247	a		2	1.656455229
mPool2_V2MM_159305	1.594981	2.205692	TRUE	HP_379902	TGCTGTTG/	GAGGTAGA	XM_283042	Mus musculus hypothetical gene supported by AK051127 (LOC328052), mRNA.			2.6	SM2128	h		1	1.656455229
mPool5_V2MM_45643	1.594664	69.72441	TRUE	HP_268170	TGCTGTTG/	CAAGGACT	NM_010171	coagulation factor III	F3	NA				NA		0.156615137
mPool7_V2MM_64143	1.594046	3.004939	TRUE	HP_286205	TGCTGTTG/	CTGACACC	NM_025781	RIKEN cDNA 9030409E16 gene	9030409E16Rik		2.8	SM2242	f		5	1.522164293
092308m3_V2MM_227395	1.594037	75.83547	TRUE	HP_479394	TGCTGTTG/	GCCTTTGA(	NM_183217	cD sequence BC042698	BC042698		2.13	SM2469	c		11	0.120127608
092308m3_V2MM_30001	1.592893	35.99881	TRUE	HP_252946	TGCTGTTG/	CAGGCTGC	NM_015740	biogenesis of lysosome-related organelles complex-1, subunit	Bloc1s1		2.12	SM2431	f		4	0.443711899
mPool2_V2MM_17541	1.591709	3.004939	TRUE	HP_240815	TGCTGTTG/	GATAGGTT	NM_133206	zinc and ring finger 1	Znrf1		2.11	SM2381	c		12	1.522164293
mPool7_V2MM_202608	1.590471	2.205692	TRUE	HP_420607	TGCTGTTG/	CCAAGTTT(	AK015845	RIKEN cDNA 4930519L02 gene	4930519L02Rik		2.9	SM2299	h		12	1.656455229
mPool7_V2MM_113916	1.590406	1.959154	TRUE	HP_334903	TGCTGTTG/	GATCTATT(	XM_137192	similar to EC1-V2R pheromone receptor protein			2.1	SM2306	f		11	1.707931489
092308m3_V2MM_194750	1.589511	61.29828	TRUE	HP_412902	TGCTGTTG/	GATGCTCG	NM_178645	bleomycin hydrolase	Blmh		2.12	SM2450	h		4	0.212551731
092308m3_V2MM_112923	1.588719	69.72441	TRUE	HP_333915	TGCTGTTG/	GGTAAGCT	XM_136719	Mus musculus LOC213721 (LOC213721), mR.			2.13	SM2494	c		1	0.156615137
mPool2_V2MM_21587	1.587724	3.004939	TRUE	HP_244758	TGCTGTTG/	CATATCAT(	NM_011991	COP9 (constitutive photomorphogenic) homolog, subunit 3 (A	Cops3		2.7	SM2165	b		11	1.522164293
092308m1_V2MM_173404	1.587555	3.004939	TRUE	HP_393890	TGCTGTTG/	GTAATAAG	NM_172463	sushi, nidogen and EGF-like domains 1	Sned1		2.5	SM2079	a		8	1.522164293
092308m3_V2MM_206242	1.586854	75.83547	TRUE	HP_424077	TGCTGTTG/	GCAACGCT/	XM_137051	Mus musculus similar to Gag [Ovis aries] (LOC212923), mRNA.			2.9	SM2300	d		5	0.120127608

092308m3_V2MM_206242	1.586854	75.83547	TRUE	HP_424077	TGCTGTTG	GCAACGCT	XM_137051	Mus musculus similar to Gag [Ovis aries] (LOC212923), mR.			2.9	SM2300	d		5	0.120127608
mPool7_V2MM_173267	1.58674	3.004939	TRUE	HP_393753	TGCTGTTG	GTAATTAT	XM_286992	Mus musculus hypothetical gene supported by AK031258 (LOC329151), mRNA.			2.9	SM2284	e		8	1.522164293
mPool2_V2MM_135185	1.586555	3.004939	TRUE	HP_355907	TGCTGTTG	GTCTAAAT	XM_150392	protein phosphatase 1A, magnesium dependent, alpha isofor	Ppm1a		2.4	SM2045	e		12	1.522164293
mPool2_V2MM_135185	1.586555	3.004939	TRUE	HP_355907	TGCTGTTG	GTCTAAAT	XM_150392	protein phosphatase 1A, magnesium dependent, alpha isofor	Ppm1a		2.4	SM2045	e		12	1.522164293
092308m1_V2MM_63506	1.586532	3.004939	TRUE	HP_285583	TGCTGTTG	CGGTGCTC	NM_029597	RIKEN cD 1700018B08 gene	1700018B08Rik		2.4	SM2025	g		4	1.522164293
mPool7_V2MM_96391	1.585834	3.004939	TRUE	HP_317644	TGCTGTTG	GAAATGTT	AK015302	RIKEN cDNA 4933432K03 gene	4933432K03Rik		2.9	SM2251	d		3	1.522164293
mPool7_V2MM_96391	1.585834	3.004939	TRUE	HP_317644	TGCTGTTG	GAAATGTT	AK015302	RIKEN cD 4933432K03 gene	4933432K03Rik		2.9	SM2251	d		3	1.522164293
mPool2_V2MM_112209	1.585509	2.205692	TRUE	HP_333215	TGCTGTTG	CTGCATAG	XM_136373	RIKEN cDNA 4930413P14 gene	4930413P14Rik		2.6	SM2148	b		11	1.656455229
mPool6_V2MM_5275	1.584245	59.99801	TRUE	HP_228829	TGCTGTTG	CCAGTGTG	NM_011629	nuclear receptor subfamily 2, group C, member 1	Nr2c1		2.7	SM2172	a		6	0.221863149
mPool7_V2MM_87647	1.583858	2.205692	TRUE	HP_309097	TGCTGTTG	CCACTATA	NM_175212	RIKEN cDNA 4930438D12 gene	4930438D12Rik		2.9	SM2263	f		5	1.656455229
mPool7_V2MM_87647	1.583858	2.205692	TRUE	HP_309097	TGCTGTTG	CCACTATA	NM_175212	RIKEN cD 4930438D12 gene	4930438D12Rik		2.9	SM2263	f		5	1.656455229
mPool2_V2MM_146549	1.583004	2.205692	TRUE	HP_367260	TGCTGTTG	CCTGCTCTT	XM_162150	Mus musculus LOC243169 (LOC243169), mRNA.			2.1	SM2337	c		10	1.656455229
mPool7_V2MM_205921	1.582907	3.004939	TRUE	HP_423769	TGCTGTTG	CTGGGAAC	AK122392	expressed sequence AI447928	AI447928		2.1	SM2307	b		5	1.522164293
092308m3_V2MM_85571	1.582464	61.29828	TRUE	HP_307052	TGCTGTTG	CCATGAAG	NM_146414	olfactory receptor 1431	Olfr1431		2.13	SM2476	g		11	0.212551731
092308m3_V2MM_114909	1.582009	75.83547	TRUE	HP_335882	TGCTGTTG	GCACTGAA	XM_138011	RIKEN cDNA E030025L21 gene	E030025L21Rik		2.1	SM2306	b		6	0.120127608
092308m3_V2MM_114909	1.582009	75.83547	TRUE	HP_335882	TGCTGTTG	GCACTGAA	XM_138011	RIKEN cD E030025L21 gene	E030025L21Rik		2.1	SM2306	b		6	0.120127608
mPool2_V2MM_4273	1.581661	3.004939	TRUE	HP_227849	TGCTGTTG	GTACTTCA	NM_019580	membrane interacting protein of RGS16			2.11	SM2379	b		7	1.522164293
mPool5_V2MM_171369	1.580833	75.83547	TRUE	HP_391858	TGCTGTTG	GACCCACA	XM_286611	Mus musculus hypothetical gene supported by AK040135 (LOC328411), mRNA.		NA				NA		0.120127608
mPool2_V2MM_104822	1.580602	2.205692	TRUE	HP_325951	TGCTGTTG	GCTGTACA	AK015207	RIKEN cDNA 4930426L09 gene	4930426L09Rik		2.7	SM2153	d		8	1.656455229
mPool2_V2MM_211363	1.578616	2.205692	TRUE	HP_429066	TGCTGTTG	CAACTGAC	XM_489556	similar to hypothetical protein			2.11	SM2361	h		10	1.656455229
mPool2_V2MM_105368	1.578457	3.004939	TRUE	HP_326487	TGCTGTTG	CCCATGAA	AK016695	RIKEN cDNA 4933406J08 gene	4933406J08Rik		2.1	SM2319	b		11	1.522164293
092308m1_V2MM_178182	1.577267	2.205692	TRUE	HP_398656	TGCTGTTG	CATATATC	AK035100	RIKEN cD 9430088F21 gene	9430088F21Rik		2.5	SM2072	a		3	1.656455229
092308m3_V2MM_33913	1.576187	75.83547	TRUE	HP_256748	TGCTGTTG	CATGATGT	NM_024435	neurotensin	Nts		2.11	SM2393	h		2	0.120127608
092308m1_V2MM_174234	1.575705	3.004939	TRUE	HP_394720	TGCTGTTG	CTTGCTGT	AK087574	RIKEN cD E230011G24 gene	E230011G24Rik		2.5	SM2060	h		8	1.522164293
mPool2_V2MM_210407	1.575421	3.004939	TRUE	HP_428160	TGCTGTTG	GGTGCAGT	XM_142191	gene model 384, (NCBI)	Gm384		2.11	SM2373	f		12	1.522164293
mPool2_V2MM_101245	1.574392	3.004939	TRUE	HP_322417	TGCTGTTG	CTAACCAA	AK083705	expressed sequence AI256775	AI256775		2.1	SM2324	e		1	1.522164293
mPool2_V2MM_101245	1.574392	3.004939	TRUE	HP_322417	TGCTGTTG	CTAACCAA	AK083705	expressed sequence AI256775	AI256775		2.1	SM2324	e		1	1.522164293
mPool2_V2MM_97289	1.573556	2.205692	TRUE	HP_318531	TGCTGTTG	CTCTGAAA	XM_111418	Mus musculus similar to ribosomal protein S9, cytosolic [validated] - rat (LOC218128), m			2.1	SM2318	c		12	1.656455229
092308m1_V2MM_178624	1.572382	3.004939	TRUE	HP_399098	TGCTGTTG	GTGATAAG	XM_288193	Mus musculus LOC332456 (LOC332456), mR.			2.5	SM2088	b		1	1.522164293
092308m1_V2MM_178624	1.572382	3.004939	TRUE	HP_399098	TGCTGTTG	GTGATAAG	XM_288193	Mus musculus LOC332456 (LOC332456), mRNA.			2.5	SM2088	b		1	1.522164293
092308m1_V2MM_119523	1.572155	3.004939	TRUE	HP_340441	TGCTGTTG	CTTCCACA	XM_140470	Mus musculus similar to ribosomal protein L10 [Rattus norvegicus] (LOC225234), mR.			2.5	SM2097	d		1	1.522164293
092308m1_V2MM_119523	1.572155	3.004939	TRUE	HP_340441	TGCTGTTG	CTTCCACA	XM_140470	Mus musculus similar to ribosomal protein L10 [Rattus norvegicus] (LOC225234), mRNA			2.5	SM2097	d		1	1.522164293
mPool2_V2MM_88856	1.572011	2.205692	TRUE	HP_310290	TGCTGTTG	CTCCTTTGG	NM_175510	RIKEN cDNA C030018G13 gene	C030018G13Rik		2.6	SM2134	e		1	1.656455229
092308m1_V2MM_141195	1.57155	2.205692	TRUE	HP_361908	TGCTGTTG	GACAAATT	XM_156514	Mus musculus LOC239894 (LOC239894), mRNA.			2.4	SM2042	d		4	1.656455229
092308m1_V2MM_141195	1.57155	2.205692	TRUE	HP_361908	TGCTGTTG	GACAAATT	XM_156514	Mus musculus LOC239894 (LOC239894), mR.			2.4	SM2042	d		4	1.656455229
092308m1_V2MM_144387	1.571385	3.004939	TRUE	HP_365098	TGCTGTTG	CTGGTCAC	XM_159943	Mus musculus LOC241958 (LOC241958), mR.			2.4	SM2038	d		2	1.522164293
092308m1_V2MM_78999	1.571372	2.205692	TRUE	HP_300674	TGCTGTTG	GAGTATAC	NM_146775	olfactory receptor 473	Olfr473		2.4	SM2026	a		9	1.656455229
092308m1_V2MM_70732	1.57053	3.004939	TRUE	HP_292637	TGCTGTTG	CAGAATGA	NM_053090	down-regulated by Ctnnb1, a			2.4	SM2021	e		5	1.522164293
mPool2_V2MM_212032	1.570461	2.205692	TRUE	HP_429713	TGCTGTTG	CACTTATTC	XM_141313	Mus musculus LOC214223 (LOC214223), mRNA.			2.11	SM2367	e		1	1.656455229
092308m3_V2MM_197834	1.5698	75.83547	TRUE	HP_415896	TGCTGTTG	GATGATAA	XM_111554	similar to Aspartate aminotransferase, mitochondrial precursor (Transaminase A) (Gluta			2.9	SM2266	b		12	0.120127608
092308m3_V2MM_197834	1.5698	75.83547	TRUE	HP_415896	TGCTGTTG	GATGATAA	XM_111554	similar to Aspartate aminotransferase, mitochondrial precursor (Transamise A) (Glutam			2.9	SM2266	b		12	0.120127608
mPool2_V2MM_112686	1.56727	3.004939	TRUE	HP_180329	TGCTGTTG	GAAGCTAT	XM_486245	similar to Rpl7a protein			2.6	SM2150	f		9	1.522164293
092308m1_V2MM_123687	1.566683	3.004939	TRUE	HP_344543	TGCTGTTG	CAATGAAT	XM_143099	Mus musculus similar to glyceraldehyde-3-phosphate dehydrogese [Mus musculus] (LO			2.5	SM2097	d		9	1.522164293
mPool2_V2MM_107197	1.56653	3.004939	TRUE	HP_328286	TGCTGTTG	CAGCCAAG	AK017309	peroxisome biogenesis factor 1	Pex1		2.7	SM2154	g		3	1.522164293
mPool2_V2MM_118608	1.565198	3.004939	TRUE	HP_339541	TGCTGTTG	CTCTAAAT	XM_139997	Mus musculus similar to glyceraldehyde-3-phosphate dehydrogenase [Mus musculus] (L			2.11	SM2372	d		1	1.522164293
mPool2_V2MM_143932	1.564727	3.004939	TRUE	HP_364644	TGCTGTTG	CAGAGATA	XM_159469	Mus musculus LOC211711 (LOC211711), mR.			2.4	SM2033	d		1	1.522164293
mPool2_V2MM_143932	1.564727	3.004939	TRUE	HP_364644	TGCTGTTG	CAGAGATA	XM_159469	Mus musculus LOC211711 (LOC211711), mRNA.			2.4	SM2033	d		1	1.522164293
092308m1_V2MM_179050	1.563952	3.004939	TRUE	HP_399524	TGCTGTTG	CCAGTTTG	XM_288266	Mus musculus LOC332539 (LOC332539), mRNA.			2.5	SM2070	b		6	1.522164293
092308m1_V2MM_179050	1.563952	3.004939	TRUE	HP_399524	TGCTGTTG	CCAGTTTG	XM_288266	Mus musculus LOC332539 (LOC332539), mR.			2.5	SM2070	b		6	1.522164293
mPool5_V2MM_19921	1.56273	66.95036	TRUE	HP_243129	TGCTGTTG	GTCTCGGA	NM_025807	solute carrier family 16 (monocarboxylic acid transporters), m	Slc16a9	NA				NA		0.1742471
mPool4_V2MM_214759	1.561206	75.83547	TRUE	HP_432333	TGCTGTTG	CAACCATG	XM_149204	RIKEN cDNA 2610507N02 gene	2610507N02Rik		2.16	SM2639	c		11	0.120127608
092308m1_V2MM_147040	1.55998	3.004939	TRUE	HP_367751	TGCTGTTG	CAGTTATG	XM_162552	Mus musculus LOC243500 (LOC243500), mR.			2.4	SM2045	a		5	1.522164293
mPool2_V2MM_123084	1.559656	2.205692	TRUE	HP_343940	TGCTGTTG	GGTGAAAT	XM_142640	serine (or cysteine) proteinase inhibitor, clade B (ovalbumin),	Serpinb3b		2.11	SM2368	e		1	1.656455229
092308m1_V2MM_141042	1.559204	3.004939	TRUE	HP_361755	TGCTGTTG	GCCTATCG	XM_156433	Mus musculus LOC239944 (LOC239944), mR.			2.4	SM2045	c		6	1.522164293
092308m3_V2MM_91895	1.557893	35.99881	TRUE	HP_313260	TGCTGTTG	GCCATTGG	NM_178612	RIKEN cD 2610019P18 gene	2610019P18Rik		2.13	SM2485	h		9	0.443711899
mPool2_V2MM_211803	1.557815	3.004939	TRUE	HP_429489	TGCTGTTG	GCACCTAT	XM_143256	similar to hypothetical protein C130079G13			2.11	SM2353	e		3	1.522164293
mPool7_V2MM_175565	1.557728	3.004939	TRUE	HP_396047	TGCTGTTG	CTGATAGC	XM_489100	hypothetical gene supported by AK031827			2.9	SM2287	b		2	1.522164293
mPool2_V2MM_125842	1.557424	3.004939	TRUE	HP_346685	TGCTGTTG	GAGCCATT	XM_144249	Mus musculus similar to vacuole membrane protein 1 [Rattus norvegicus] (LOC231084),			2.11	SM2365	a		4	1.522164293
mPool7_V2MM_202467	1.557327	2.205692	TRUE	HP_420474	TGCTGTTG	CTGGTGAC	AK075654	RIKEN cDNA 3830408G10 gene	3830408G10Rik		2.1	SM2313	c		12	1.656455229
mPool2_V2MM_208600	1.55657	2.205692	TRUE	HP_329664	TGCTGTTG	GAGAAGAG	XM_289903	RIKEN cDNA 2500002G23 gene	2500002G23Rik		2.1	SM2334	g		6	1.656455229
mPool4_V2MM_235772	1.555647	75.83547	TRUE	HP_487635	TGCTGTTG	GTGTACAT	XM_155527	Mus musculus LOC207548 (LOC207548), mRNA.			2.16	SM2644	e		11	0.120127608
092308m3_V2MM_226600	1.554549	60.13437	TRUE	HP_478616	TGCTGTTG	CTGTCTCTG	NM_178690	RIKEN cD 4732493F09 gene	4732493F09Rik		2.13	SM2482	d		6	0.220877236

mPool7_V2MM_196687	1.554047	3.004939	TRUE	HP_414782	TGCTGTTG	CTGTGAAA	NM_175519	potassium channel tetramerisation domain containing 8	Kctd8		2.9	SM2265	g		5	1.522164293
092308m1_V2MM_169472	1.553705	3.004939	TRUE	HP_389967	TGCTGTTG	CCTCCGTA	XM_286267	Mus musculus hypothetical gene supported by AK038448 (LOC327785), mR.			2.6	SM2122	b		10	1.522164293
092308m3_V2MM_103006	1.553484	75.83547	TRUE	NA	NA	NA	NA	NA	NA	NA		NA	NA	NA		0.120127608
mPool7_V2MM_91650	1.552805	3.004939	TRUE	HP_313019	TGCTGTTG	GTTATTACT	NM_178397	RIKEN cDNA 2210404D11 gene	2210404D11Rik		2.9	SM2255	g		12	1.522164293
mPool2_V2MM_128961	1.551638	2.205692	TRUE	HP_349774	TGCTGTTG	GATTATCA	XM_146032	Mus musculus similar to ORC1-related protein (LOC233711), mRNA.			2.11	SM2365	d		9	1.656455229
092308m3_V2MM_226910	1.551575	75.83547	TRUE	HP_478919	TGCTGTTG	GTGTTTATA	NM_178613	RIKEN cD 4933433P14 gene	4933433P14Rik		2.13	SM2474	d		11	0.120127608
mPool6_V2MM_81066	1.550831	75.83547	TRUE	HP_302685	TGCTGTTG	GGCTGCTT	NM_008071	gamma-aminobutyric acid (GABA-A) receptor, subunit beta 3	Gabrb3		2.7	SM2181	h		11	0.120127608
mPool7_V2MM_84322	1.549913	3.004939	TRUE	HP_305835	TGCTGTTG	CTGTATAA	XM_484255	nucleolar protein 8	Nol8		2.9	SM2268	h		7	1.522164293
mPool7_V2MM_82060	1.549911	3.004939	TRUE	HP_303646	TGCTGTTG	GTTCAAGT	NM_146966	olfactory receptor 1247	Olfr1247		2.8	SM2229	f		4	1.522164293
092308m1_V2MM_145690	1.549745	2.205692	TRUE	HP_366401	TGCTGTTG	GAGGAAAT	XM_161242	Mus musculus LOC242544 (LOC242544), mR.		NA				NA		1.656455229
mPool2_V2MM_144083	1.549507	1.959154	TRUE	HP_364794	TGCTGTTG	CCAGATAG	AY462058	kininogen 1	Kng1		2.1	SM2335	e		2	1.707931489
mPool2_V2MM_209186	1.548832	3.004939	TRUE	HP_426962	TGCTGTTG	CTGGAAAT	XM_151696	Mus musculus LOC237328 (LOC237328), mRNA.			2.1	SM2336	h		2	1.522164293
mPool7_V2MM_183932	1.548526	3.004939	TRUE	HP_404406	TGCTGTTG	CACCTCCT	XM_289106	Mus musculus LOC331565 (LOC331565), mRNA.			2.9	SM2277	g		2	1.522164293
mPool7_V2MM_174757	1.548195	3.004939	TRUE	HP_395242	TGCTGTTG	CAATCCGC	XM_287297	Mus musculus hypothetical gene supported by AK080820 (LOC329769), mRNA.			2.9	SM2288	e		12	1.522164293
mPool2_V2MM_98015	1.547167	1.959154	TRUE	HP_319251	TGCTGTTG	GGATATTG	XM_112038	Mus musculus similar to putative pheromone receptor (LOC195691), mRNA.			2.6	SM2145	g		3	1.707931489
092308m3_V2MM_194721	1.546031	75.83547	TRUE	HP_412875	TGCTGTTG	CACCCGGT	NM_027008	potassium channel tetramerisation domain containing 5	Kctd5		2.8	SM2249	a		6	0.120127608
092308m3_V2MM_194721	1.546031	75.83547	TRUE	HP_412875	TGCTGTTG	CACCCGGT	NM_027008	potassium channel tetramerisation domain containing 5	Kctd5		2.8	SM2249	a		6	0.120127608
mPool2_V2MM_105383	1.545677	3.004939	TRUE	HP_326499	TGCTGTTG	GACTATAC	BC024760	cDNA sequence BC024760	BC024760		2.7	SM2154	e		7	1.522164293
mPool2_V2MM_18640	1.544649	3.004939	TRUE	HP_241886	TGCTGTTG	GCTCCTATT	NM_018864	inositol (myo)-1(or 4)-monophosphatase 1	Impa1		2.11	SM2381	g		3	1.522164293
092308m3_V2MM_103386	1.543647	60.13437	TRUE	HP_324528	TGCTGTTG	CAGCTGTT	AK020725	RIKEN cD 2700046A07 gene	2700046A07Rik		2.14	SM2506	c		6	0.220877236
mPool7_V2MM_173876	1.54347	2.205692	TRUE	HP_394362	TGCTGTTG	CAGTGTCA	AK038492	UDP-N-acetyl-alpha-D-galactosamine:polypeptide N-acetylgal	Galnt13		2.9	SM2271	b		1	1.656455229
mPool2_V2MM_13272	1.543097	2.205692	TRUE	HP_236643	TGCTGTTG	CAGTCATT	NM_009062	regulator of G-protein signaling 4	Rgs4		2.11	SM2375	h		8	1.656455229
mPool2_V2MM_213248	1.542836	3.004939	TRUE	HP_430888	TGCTGTTG	CGTATTATA	XM_141639	Mus musculus similar to protease [Mus musculus] (LOC236704), mRNA.			2.11	SM2372	d		11	1.522164293
mPool6_V2MM_160575	1.541416	69.72441	TRUE	HP_381154	TGCTGTTG	CATCGCGT	XM_283569	Mus musculus hypothetical gene supported by AK081501 (LOC329063), mRNA.			2.8	SM2206	a		4	0.156615137
mPool2_V2MM_124711	1.538964	3.004939	TRUE	HP_345560	TGCTGTTG	GTCAGTGT	XM_143599	Mus musculus similar to RNA polymerase II transcriptional coactivator [Mus musculus] (			2.11	SM2367	c		2	1.522164293
092308m1_V2MM_174386	1.536601	3.004939	TRUE	HP_394871	TGCTGTTG	GGGTTAGT	XM_287220	Mus musculus hypothetical gene supported by AK045957 (LOC329604), mR.			2.5	SM2079	g		10	1.522164293
092308m1_V2MM_144635	1.536364	3.004939	TRUE	HP_365346	TGCTGTTG	CATCCCAC	XM_160151	Mus musculus LOC212834 (LOC212834), mR.			2.5	SM2057	f		9	1.522164293
mPool7_V2MM_197062	1.536062	2.205692	TRUE	HP_415147	TGCTGTTG	GTCTATTCA	NM_146660	olfactory receptor 1135	Olfr1135		2.9	SM2255	g		6	1.656455229
mPool2_V2MM_4364	1.535818	2.205692	TRUE	HP_227939	TGCTGTTG	GTCTTTGAT	NM_027356	RIKEN cDNA 2700038N03 gene	2700038N03Rik		2.11	SM2383	e		11	1.656455229
mPool2_V2MM_111805	1.535686	3.004939	TRUE	HP_332812	TGCTGTTG	GACAAATG	XM_136178	expressed sequence AI132431	AI132431		2.7	SM2158	b		10	1.522164293
092308m1_V2MM_187047	1.534193	3.004939	TRUE	HP_407516	TGCTGTTG	CAAAGAAC	XM_289640	Mus musculus LOC333402 (LOC333402), mR.			2.5	SM2072	f		3	1.522164293
092308m1_V2MM_187047	1.534193	3.004939	TRUE	HP_407516	TGCTGTTG	CAAAGAAC	XM_289640	Mus musculus LOC333402 (LOC333402), mRNA.			2.5	SM2072	f		3	1.522164293
mPool6_V2MM_155861	1.534173	69.72441	TRUE	HP_376500	TGCTGTTG	CATATTTG	XM_197863	Mus musculus similar to hypothetical protein MGC19022 [Mus musculus] (LOC272156),			2.7	SM2199	c		7	0.156615137
mPool4_V2MM_215022	1.534074	62.64426	TRUE	HP_432591	TGCTGTTG	CAGCCACT	XM_141013	Mus musculus similar to mitochondrial ribosomal protein S36; similar to RIKEN cDNA 11			2.1	SM2349	d		8	0.203118686
mPool2_V2MM_213314	1.533775	3.004939	TRUE	HP_430953	TGCTGTTG	CCCTGTGA	XM_149823	Mus musculus LOC232625 (LOC232625), mRNA.			2.11	SM2361	b		3	1.522164293
mPool5_V2MM_160442	1.533276	70.87591	TRUE	HP_381022	TGCTGTTG	GCGTCTAT	AK033711	hypothetical protein 9130229N11		NA				NA		0.149501346
092308m1_V2MM_172048	1.531848	2.205692	TRUE	HP_392534	TGCTGTTG	CAACAGTC	XM_286748	Mus musculus hypothetical gene supported by AK079475 (LOC328668), mR.			2.5	SM2081	a		11	1.656455229
092308m3_V2MM_88257	1.531505	66.95036	TRUE	HP_309699	TGCTGTTG	GTTAACAT	NM_175355	RIKEN cD E330013P04 gene	E330013P04Rik		2.9	SM2254	e		10	0.1742471
092308m3_V2MM_88257	1.531505	66.95036	TRUE	HP_309699	TGCTGTTG	GTTAACAT	NM_175355	RIKEN cDNA E330013P04 gene	E330013P04Rik		2.9	SM2254	e		10	0.1742471
092308m1_V2MM_167173	1.531002	3.004939	TRUE	HP_387687	TGCTGTTG	CAGCCTAT	XM_285713	Mus musculus similar to variant-specific surface protein S7 [Giardia intestilis] (LOC3296			2.6	SM2124	d		10	1.522164293
092308m1_V2MM_70292	1.530813	3.004939	TRUE	HP_292208	TGCTGTTG	CAAATTTG	NM_010514	insulin-like growth factor 2	Igf2		2.3	SM2009	g		10	1.522164293
mPool2_V2MM_118341	1.530787	2.205692	TRUE	HP_339278	TGCTGTTG	CCTGTATA	XM_139796	Mus musculus similar to putative pheromone receptor [Mus musculus] (LOC224580), m			2.11	SM2354	a		12	1.656455229
mPool2_V2MM_96187	1.530785	3.004939	TRUE	HP_317447	TGCTGTTG	CTTCCTAAA	XM_489704	similar to G protein coupled receptor			2.6	SM2138	a		1	1.522164293
mPool7_V2MM_176671	1.529466	3.004939	TRUE	HP_397151	TGCTGTTG	CTCAGGAG	XM_287716	Mus musculus hypothetical gene supported by AK085843 (LOC330610), mRNA.			2.9	SM2273	f		1	1.522164293
mPool7_V2MM_173142	1.529013	3.004939	TRUE	HP_393628	TGCTGTTG	GGAGTAAT	XM_286966	Mus musculus hypothetical gene supported by AK051123 (LOC329083), mR.			2.5	SM2089	f		4	1.522164293
mPool7_V2MM_173142	1.529013	3.004939	TRUE	HP_393628	TGCTGTTG	GGAGTAAT	XM_286966	Mus musculus hypothetical gene supported by AK051123 (LOC329083), mRNA.			2.5	SM2089	f		4	1.522164293
092308m1_V2MM_179101	1.528739	3.004939	TRUE	HP_399575	TGCTGTTG	CCACATTCA	XM_288274	Mus musculus LOC332550 (LOC332550), mR.			2.5	SM2092	a		9	1.522164293
092308m1_V2MM_179101	1.528739	3.004939	TRUE	HP_399575	TGCTGTTG	CCACATTCA	XM_288274	Mus musculus LOC332550 (LOC332550), mRNA.			2.5	SM2092	a		9	1.522164293
mPool4_V2MM_221042	1.528693	75.83547	TRUE	HP_453983	TGCTGTTG	GTTCAAGT	NM_146828	olfactory receptor 975	Olfr975		2.15	SM2598	c		10	0.120127608
mPool2_V2MM_120787	1.527815	3.004939	TRUE	HP_341678	TGCTGTTG	CCAGTAAT	XM_485031	olfactory receptor 48	Olfr48		2.11	SM2353	a		3	1.522164293
092308m1_V2MM_184437	1.527239	3.004939	TRUE	HP_404909	TGCTGTTG	CTGTCTAT	XM_289198	Mus musculus LOC332831 (LOC332831), mR.			2.5	SM2067	b		3	1.522164293
mPool2_V2MM_169682	1.526893	3.004939	TRUE	HP_390177	TGCTGTTG	CAACCAAT	XM_286315	Mus musculus hypothetical gene supported by AK049252 (LOC327897), mRNA.			2.6	SM2128	c		4	1.522164293
092308m1_V2MM_72432	1.526423	2.205692	TRUE	HP_294290	TGCTGTTG	CTGGTCAG	NM_173433	hypothetical protein 4932416A15			2.3	SM2012	a		5	1.656455229
mPool7_V2MM_186962	1.525513	3.004939	TRUE	HP_407431	TGCTGTTG	CTATTTGG	XM_289626	Mus musculus LOC333388 (LOC333388), mRNA.			2.9	SM2292	h		6	1.522164293
mPool2_V2MM_105540	1.525092	3.004939	TRUE	HP_326652	TGCTGTTG	GGCTAACT	NM_177652	ryanodine receptor 3	Ryr3		2.7	SM2159	c		7	1.522164293
mPool2_V2MM_98480	1.524275	3.004939	TRUE	HP_319706	TGCTGTTG	CCGTGTTG	XM_112566	Mus musculus LOC193371 (LOC193371), mRNA.			2.6	SM2138	d		4	1.522164293
mPool4_V2MM_236964	1.523556	75.83547	TRUE	NA	NA	NA	NA	NA	NA	NA		NA	NA	NA		0.120127608
mPool2_V2MM_87388	1.521684	2.205692	TRUE	HP_308844	TGCTGTTG	GACTCTTCA	NM_175153	RIKEN cDNA 2010321M09 gene	2010321M09Rik		2.6	SM2141	b		2	1.656455229
mPool2_V2MM_150955	1.520861	1.959154	TRUE	HP_371630	TGCTGTTG	CTCTTGAA	XM_194334	RIKEN cDNA 0710005I19 gene	0710005I19Rik		2.1	SM2345	f		5	1.707931489
092308m1_V2MM_82790	1.519708	2.205692	TRUE	HP_304353	TGCTGTTG	CCAGGAGT	NM_010470	heterochromatin protein 1, binding protein 3	Hp1bp3		2.6	SM2109	b		5	1.656455229
mPool7_V2MM_194397	1.519333	3.004939	TRUE	HP_412562	TGCTGTTG	GAAATAAT	NM_175009	RIKEN cDNA 1810057B09 gene	1810057B09Rik		2.9	SM2265	b		2	1.522164293
mPool7_V2MM_194397	1.519333	3.004939	TRUE	HP_412562	TGCTGTTG	GAAATAAT	NM_175009	RIKEN cD 1810057B09 gene	1810057B09Rik		2.9	SM2265	b		2	1.522164293

mPool4_V2MM_203900	1.518837	35.99881	TRUE	HP_421826	TGCTGTTG	CACCAGTG	XM_138918	Mus musculus similar to erythrocyte membrane protein 3 [Plasmodium falciparum 3D7		2.16	SM2621	d	5	0.443711899
092308m3_V2MM_86162	1.517734	75.83547	TRUE	HP_307638	TGCTGTTG	CTGCTCATT	NM_146635	olfactory receptor 1489	Olfr1489	2.13	SM2458	d	10	0.120127608
092308m3_V2MM_93062	1.517347	59.99801	TRUE	HP_314396	TGCTGTTG	CAGTGAGA	NM_178897	expressed sequence AW125391	AW125391	2.9	SM2262	h	7	0.221863149
092308m3_V2MM_93062	1.517347	59.99801	TRUE	HP_314396	TGCTGTTG	CAGTGAGA	NM_178897	expressed sequence AW125391	AW125391	2.9	SM2262	h	7	0.221863149
mPool2_V2MM_14561	1.516491	2.205692	TRUE	HP_237906	TGCTGTTG	CCTGTAAA	NM_008328	interferon activated gene 203	Ifi203	2.11	SM2388	h	11	1.656455229
mPool2_V2MM_83438	1.515601	3.004939	TRUE	HP_304980	TGCTGTTG	GATCCTAT	NM_015767	tocopherol (alpha) transfer protein	Ttpa	2.6	SM2135	f	4	1.522164293
092308m1_V2MM_144680	1.515213	2.205692	TRUE	HP_365391	TGCTGTTG	CATATTATG	XM_160198	Mus musculus LOC213218 (LOC213218), mR.		2.4	SM2047	c	6	1.656455229
092308m1_V2MM_172940	1.514759	3.004939	TRUE	HP_393426	TGCTGTTG	CAGCCCAT	XM_286926	gene model 815, (NCBI)	Gm815	2.5	SM2060	d	2	1.522164293
092308m1_V2MM_143833	1.514155	3.004939	TRUE	HP_364545	TGCTGTTG	CATGAACA	XM_159183	Mus musculus LOC245402 (LOC245402), mR.		2.4	SM2038	f	10	1.522164293
092308m1_V2MM_143833	1.514155	3.004939	TRUE	HP_364545	TGCTGTTG	CATGAACA	XM_159183	Mus musculus LOC245402 (LOC245402), mRNA.		2.4	SM2038	f	10	1.522164293
092308m1_V2MM_182065	1.513401	3.004939	TRUE	HP_402539	TGCTGTTG	CTCATCCGT	XM_288781	Mus musculus LOC332243 (LOC332243), mR.		2.5	SM2068	h	6	1.522164293
mPool2_V2MM_31752	1.512977	3.004939	TRUE	HP_254645	TGCTGTTG	GAAAGTAG	NM_019708	short coiled-coil protein	Scoc	2.11	SM2392	d	2	1.522164293
mPool2_V2MM_131650	1.512862	2.205692	TRUE	HP_352411	TGCTGTTG	CATGTGTT	BC023150	heterogeneous nuclear ribonucleoprotein A0	Hnrpa0	2.11	SM2373	c	7	1.656455229
mPool2_V2MM_25174	1.512408	2.205692	TRUE	HP_248247	TGCTGTTG	CAATGTTA	NM_009194	solute carrier family 12, member 2	Slc12a2	2.7	SM2168	c	1	1.656455229
092308m1_V2MM_155615	1.511965	2.205692	TRUE	HP_376254	TGCTGTTG	CAAGATCC	XM_197644	Mus musculus LOC271898 (LOC271898), mR.		2.6	SM2115	b	3	1.656455229
mPool7_V2MM_206173	1.511791	3.004939	TRUE	HP_424012	TGCTGTTG	GAATGTAA	XM_128618	similar to zinc finger protein 97		2.1	SM2317	h	4	1.522164293
092308m1_V2MM_172295	1.510556	2.205692	TRUE	HP_392781	TGCTGTTG	CATACTGG	XM_286795	Mus musculus hypothetical gene supported by AK035334 (LOC328762), mR.		2.5	SM2076	b	11	1.656455229
mPool2_V2MM_5054	1.510332	2.205692	TRUE	HP_228613	TGCTGTTG	CAGTTAGA	NM_008260	forkhead box A3	Foxa3	2.11	SM2387	a	6	1.656455229
mPool7_V2MM_176264	1.50943	3.004939	TRUE	HP_396746	TGCTGTTG	CATGCTAT	AK028992	RIKEN cDNA 6230400G14 gene	6230400G14Rik	2.9	SM2273	g	3	1.522164293
092308m3_V2MM_96516	1.508449	35.99881	TRUE	HP_317767	TGCTGTTG	CAGAGCAT	XM_110787	UPF3 regulator of nonsense transcripts homolog B (yeast)	Upf3b	2.12	SM2450	g	3	0.443711899
092308m3_V2MM_202732	1.50821	75.83547	TRUE	HP_420722	TGCTGTTG	CCATTATTC	XM_138204	Mus musculus similar to RIKEN cDNA 2810037C14 [Mus musculus] (LOC217805), mRNA		2.1	SM2319	b	7	0.120127608
092308m3_V2MM_202732	1.50821	75.83547	TRUE	HP_420722	TGCTGTTG	CCATTATTC	XM_138204	Mus musculus similar to RIKEN cD 2810037C14 [Mus musculus] (LOC217805), mR.		2.1	SM2319	b	7	0.120127608
mPool7_V2MM_203995	1.50764	3.004939	TRUE	HP_421914	TGCTGTTG	GCTCTAAC	BC050039	RIKEN cDNA D330013L20 gene	D330013L20Rik	2.1	SM2303	g	8	1.522164293
mPool2_V2MM_83891	1.50646	2.205692	TRUE	HP_305413	TGCTGTTG	GAGCTGTA	NM_026112	zinc finger protein 606	Zfp606	2.6	SM2134	e	5	1.656455229
mPool6_V2MM_27137	1.505746	75.83547	TRUE	HP_250165	TGCTGTTG	CTGTGTAG	AK041928	RIKEN cDNA A630047E20 gene	A630047E20Rik	2.7	SM2182	a	3	0.120127608
mPool7_V2MM_214362	1.505284	2.205692	TRUE	HP_304161	TGCTGTTG	GACTTTAT	X60958	CD80 antigen	Cd80	2.9	SM2270	a	8	1.656455229
092308m3_V2MM_126924	1.505177	75.83547	TRUE	HP_281955	TGCTGTTG	GCTGCCCT	NM_011653	tubulin, alpha 1	Tuba1	2.6	SM2111	c	1	0.120127608
mPool7_V2MM_183717	1.50354	3.004939	TRUE	HP_404191	TGCTGTTG	CAGAGACT	XM_289068	Mus musculus LOC333518 (LOC333518), mRNA.		2.5	SM2070	d	9	1.522164293
mPool7_V2MM_183717	1.50354	3.004939	TRUE	HP_404191	TGCTGTTG	CAGAGACT	XM_289068	Mus musculus LOC333518 (LOC333518), mR.		2.5	SM2070	d	9	1.522164293
092308m3_V2MM_101003	1.503375	75.83547	TRUE	HP_322179	TGCTGTTG	GGTGTATA	XM_488906	LOC433341		2.13	SM2490	c	8	0.120127608
mPool7_V2MM_85669	1.503228	2.205692	TRUE	HP_307150	TGCTGTTG	CTGTATATT	NM_146438	olfactory receptor 938	Olfr938	2.9	SM2267	h	4	1.656455229
mPool7_V2MM_85669	1.503228	2.205692	TRUE	HP_307150	TGCTGTTG	CTGTATATT	NM_146438	olfactory receptor 938	Olfr938	2.9	SM2267	h	4	1.656455229
mPool2_V2MM_132452	1.501643	3.004939	TRUE	HP_353203	TGCTGTTG	CCAACTGA	XM_489556	similar to hypothetical protein		2.11	SM2362	b	4	1.522164293
mPool2_V2MM_107065	1.501547	2.205692	TRUE	HP_328156	TGCTGTTG	CGCCCATG	NM_198610	cDNA sequence BC055811	BC055811	2.1	SM2323	a	9	1.656455229
mPool2_V2MM_34221	1.501173	2.205692	TRUE	HP_257048	TGCTGTTG	CCATACAG	NM_019720	cytochrome b-561 domain containing 2	Cyb561d2	2.11	SM2390	h	5	1.656455229
092308m1_V2MM_127942	1.501066	3.004939	TRUE	HP_348760	TGCTGTTG	CAGTGATT	XM_145466	similar to cytochrome P450 CYP2B21		2.6	SM2104	g	2	1.522164293
mPool6_V2MM_79262	1.500817	62.64426	TRUE	HP_300928	TGCTGTTG	GATGTGAG	AF285588	sal-like 4 (Drosophila)	Sall4	2.8	SM2218	d	9	0.203118686
mPool7_V2MM_104355	1.499271	3.004939	TRUE	HP_325489	TGCTGTTG	CCCTATTG	AK077997	RIKEN cDNA 6130401J04 gene	6130401J04Rik	2.6	SM2149	e	1	1.522164293
mPool2_V2MM_131836	1.498981	3.004939	TRUE	HP_352596	TGCTGTTG	GAAGAAGT	AK018473	RIKEN cDNA 8430437N05 gene	8430437N05Rik	2.11	SM2357	c	2	1.522164293
mPool2_V2MM_89062	1.497919	3.004939	TRUE	HP_310493	TGCTGTTG	GGTATTGG	AK031726	RIKEN cDNA C130073D16 gene	C130073D16Rik	2.6	SM2139	b	4	1.522164293
mPool7_V2MM_106117	1.497455	2.205692	TRUE	HP_327223	TGCTGTTG	CTTTATGA	BC064474	cingulin	Cgn	2.1	SM2315	g	5	1.656455229
mPool7_V2MM_109915	1.497161	2.205692	TRUE	HP_330960	TGCTGTTG	CTGGATCA	AK016466	RIKEN cDNA 4931420C21 gene	4931420C21Rik	2.9	SM2299	g	7	1.656455229
mPool2_V2MM_92734	1.496458	3.004939	TRUE	HP_314078	TGCTGTTG	GCTCCTTAT	NM_178764	RIKEN cDNA B930006L02 gene	B930006L02Rik	2.6	SM2147	g	3	1.522164293
mPool7_V2MM_70831	1.495737	3.004939	TRUE	HP_292732	TGCTGTTG	CAGATGGA	NM_026055	ribosomal protein L39	Rpl39	2.8	SM2237	c	6	1.522164293
mPool2_V2MM_127131	1.495649	2.205692	TRUE	HP_347963	TGCTGTTG	GCCTCAAC	XM_145005	IQ motif and Sec7 domain 3	Iqsec3	2.11	SM2361	c	5	1.656455229
mPool4_V2MM_16927	1.494767	61.29828	TRUE	HP_240213	TGCTGTTG	GACTTTGT	NM_030597	LSM2 homolog, U6 small nuclear RNA associated (S. cerevisia	Lsm2	2.15	SM2577	a	2	0.212551731
mPool2_V2MM_19545	1.494639	2.205692	TRUE	HP_242761	TGCTGTTG	GGTGTAAA	NM_134190	vomeronasal 1 receptor, E1	V1re1	2.11	SM2374	f	11	1.656455229
mPool7_V2MM_182988	1.494619	3.004939	TRUE	HP_403462	TGCTGTTG	CAGTCTGT	XM_288940	Mus musculus LOC332580 (LOC332580), mRNA.		2.9	SM2285	f	5	1.522164293
092308m1_V2MM_169424	1.494514	3.004939	TRUE	HP_389919	TGCTGTTG	CCCTGAAC	AK039487	RIKEN cD A330049N07 gene	A330049N07Rik	2.6	SM2122	e	3	1.522164293
mPool2_V2MM_100395	1.494109	2.205692	TRUE	HP_321586	TGCTGTTG	CTATTTCTG	XM_126847	kelch repeat and BTB (POZ) domain containing 9	Kbtbd9	2.7	SM2163	e	1	1.656455229
mPool2_V2MM_115091	1.493748	3.004939	TRUE	NA	NA	NA	NA	NA	NA	NA	NA	NA	NA	1.522164293
092308m1_V2MM_68318	1.493576	2.205692	TRUE	HP_290281	TGCTGTTG	CTTGGCAT	NM_013737	phospholipase A2, group VII (platelet-activating factor acetylh	Pla2g7	2.4	SM2020	h	2	1.656455229
mPool2_V2MM_8298	1.493277	2.205692	TRUE	HP_231786	TGCTGTTG	GTATAAAC	XM_122407	gene model 1818, (NCBI)	Gm1818	2.11	SM2383	e	12	1.656455229
mPool6_V2MM_154711	1.493077	35.99881	TRUE	HP_375352	TGCTGTTG	CAACGAGA	XM_196979	Mus musculus LOC270985 (LOC270985), mRNA.		2.7	SM2197	d	10	0.443711899
mPool5_V2MM_182203	1.492862	62.64426	TRUE	HP_402677	TGCTGTTG	CTGCTGGC	XM_288804	Mus musculus LOC332255 (LOC332255), mRNA.		NA			NA	0.203118686
mPool2_V2MM_143911	1.492824	3.004939	TRUE	HP_364623	TGCTGTTG	CAGCTATA	XM_159415	Mus musculus LOC245652 (LOC245652), mRNA.		2.5	SM2054	f	5	1.522164293
mPool2_V2MM_143911	1.492824	3.004939	TRUE	HP_364623	TGCTGTTG	CAGCTATA	XM_159415	Mus musculus LOC245652 (LOC245652), mR.		2.5	SM2054	f	5	1.522164293
mPool2_V2MM_123086	1.492113	2.205692	TRUE	HP_343942	TGCTGTTG	GGGTGAAA	XM_142640	serine (or cysteine) proteinase inhibitor, clade B (ovalbumin),	Serpinb3b	2.6	SM2104	a	5	1.656455229
mPool2_V2MM_123086	1.492113	2.205692	TRUE	HP_343942	TGCTGTTG	GGGTGAAA	XM_142640	serine (or cysteine) proteise inhibitor, clade B (ovalbumin), m	Serpinb3b	2.6	SM2104	a	5	1.656455229
mPool7_V2MM_203153	1.491948	3.004939	TRUE	HP_421117	TGCTGTTG	GCTCTTAA	AK021149	potassium voltage-gated channel, subfamily H (eag-related), r	Kcnh3	2.9	SM2297	d	9	1.522164293
mPool2_V2MM_134899	1.491897	3.004939	TRUE	HP_355623	TGCTGTTG	GATACCTC	XM_486209	similar to sc328		2.11	SM2374	g	1	1.522164293
mPool7_V2MM_68429	1.490868	3.004939	TRUE	HP_290387	TGCTGTTG	GAAATTCT	AK046834	RIKEN cDNA A230065J02 gene	A230065J02Rik	2.8	SM2244	b	6	1.522164293

mPool2_V2MM_166417	1.490852	3.004939	TRUE	HP_386945	TGCTGTTG	CCCAACATT	XM_285495	Mus musculus similar to TRAF6-inhibitory zinc finger protein; TRAF6-binding zinc finger			2.6	SM2132	e		2	1.522164293
mPool2_V2MM_104372	1.490102	3.004939	TRUE	HP_325506	TGCTGTTG	CTCCGATCT	XM_129726	RIKEN cDNA 2410118I19 gene	2410118I19Rik		2.7	SM2163	g		2	1.522164293
mPool4_V2MM_110485	1.488575	60.13437	TRUE	HP_331519	TGCTGTTG	CCCATTATG	AK014852	RIKEN cDNA 4921509F24 gene	4921509F24Rik		2.16	SM2612	h		2	0.220877236
092308m1_V2MM_78092	1.488531	3.004939	TRUE	HP_299794	TGCTGTTG	GAAGTTAA	NM_172817	zinc finger protein 647	Zfp647		2.4	SM2022	c		5	1.522164293
092308m3_V2MM_46522	1.487615	75.83547	TRUE	HP_105243	TGCTGTTG	CCCTGTGTT	XM_484805	similar to Cytochrome P450, family 2, subfamily c, polypeptide 40			2.12	SM2416	e		7	0.120127608
092308m1_V2MM_67026	1.487005	3.004939	TRUE	HP_289026	TGCTGTTG	CCCATGCG	NM_053085	transcription factor 23	Tcf23		2.3	SM2016	h		7	1.522164293
092308m3_V2MM_33765	1.485245	75.83547	TRUE	HP_256605	TGCTGTTG	CAGTGGAA	NM_133658	excision repair cross-complementing rodent repair deficiency,	Ercc3		2.12	SM2432	d		6	0.120127608
mPool2_V2MM_118795	1.484785	3.004939	TRUE	HP_339725	TGCTGTTG	CTAATGAA	XM_140071	Mus musculus similar to protease [Mus musculus] (LOC215435), mRNA.			2.11	SM2368	b		8	1.522164293
mPool4_V2MM_124214	1.484565	75.83547	TRUE	HP_345066	TGCTGTTG	CTCGTGGT	XM_143348	Mus musculus similar to protease [Mus musculus] (LOC213288), mRNA.			2.14	SM2532	c		9	0.120127608
092308m3_V2MM_34522	1.484526	59.99801	TRUE	HP_257342	TGCTGTTG	CCCGGAGG	NM_153407	cD sequence BC035295	BC035295		2.12	SM2427	e		10	0.221863149
mPool2_V2MM_109764	1.484005	2.205692	TRUE	HP_330814	TGCTGTTG	CTGATTGA	AK020544	RIKEN cDNA 9530004P13 gene	9530004P13Rik		2.6	SM2148	g		12	1.656455229
092308m3_V2MM_23256	1.483568	75.83547	TRUE	HP_246390	TGCTGTTG	CTGCTGCT	NM_009295	syntaxin binding protein 1	Stxbp1		2.12	SM2426	b		5	0.120127608
mPool2_V2MM_165536	1.483557	3.004939	TRUE	HP_386071	TGCTGTTG	CTGAGGAA	XM_285270	Mus musculus similar to RIKEN cDNA 4932409F11 [Mus musculus] (LOC328896), mRNA.			2.6	SM2130	b		9	1.522164293
mPool2_V2MM_105600	1.482355	2.205692	TRUE	HP_326711	TGCTGTTG	GGGATGAT	XM_130642	RIKEN cDNA 5430405G05 gene	5430405G05Rik		2.6	SM2147	a		9	1.656455229
mPool7_V2MM_93732	1.482234	3.004939	TRUE	HP_315036	TGCTGTTG	CGAAATGA	NM_181748	G protein-coupled receptor 120	Gpr120		2.9	SM2263	e		11	1.522164293
mPool7_V2MM_93732	1.482234	3.004939	TRUE	HP_315036	TGCTGTTG	CGAAATGA	NM_181748	G protein-coupled receptor 120	Gpr120		2.9	SM2263	e		11	1.522164293
mPool7_V2MM_177109	1.482223	3.004939	TRUE	HP_397589	TGCTGTTG	GTGTAAAT	XM_287805	Mus musculus hypothetical gene supported by AK032925 (LOC330762), mRNA.			2.5	SM2063	h		6	1.522164293
mPool7_V2MM_177109	1.482223	3.004939	TRUE	HP_397589	TGCTGTTG	GTGTAAAT	XM_287805	Mus musculus hypothetical gene supported by AK032925 (LOC330762), mR.			2.5	SM2063	h		6	1.522164293
092308m3_V2MM_29314	1.482012	75.83547	TRUE	HP_252277	TGCTGTTG	CAAAGTTG	NM_146253	zinc finger protein 482	Zfp482		2.12	SM2425	c		1	0.120127608
mPool2_V2MM_118493	1.481935	3.004939	TRUE	HP_339428	TGCTGTTG	GATATGAA	XM_139876	cDNA sequence D10628	D10628		2.11	SM2359	d		4	1.522164293
092308m1_V2MM_123086	1.481921	2.205692	TRUE	HP_343942	TGCTGTTG	GGGTGAAA	XM_142640	serine (or cysteine) proteinase inhibitor, clade B (ovalbumin),	Serpinb3b		2.6	SM2104	a		5	1.656455229
092308m1_V2MM_123086	1.481921	2.205692	TRUE	HP_343942	TGCTGTTG	GGGTGAAA	XM_142640	serine (or cysteine) proteise inhibitor, clade B (ovalbumin), m	Serpinb3b		2.6	SM2104	a		5	1.656455229
mPool2_V2MM_109641	1.48147	3.004939	TRUE	HP_330695	TGCTGTTG	GACCTTGT	XM_134026	RIKEN cDNA 2310051N18 gene	2310051N18Rik		2.6	SM2150	a		12	1.522164293
mPool7_V2MM_202438	1.481425	3.004939	TRUE	HP_420445	TGCTGTTG	CTGCTTAA	XM_485379	similar to 2610030H06Rik protein			2.1	SM2306	h		2	1.522164293
092308m1_V2MM_177029	1.481365	2.205692	TRUE	HP_397509	TGCTGTTG	CCATTGGA	XM_287788	Mus musculus hypothetical gene supported by AK036103 (LOC330739), mRNA.			2.5	SM2081	a		1	1.656455229
092308m1_V2MM_177029	1.481365	2.205692	TRUE	HP_397509	TGCTGTTG	CCATTGGA	XM_287788	Mus musculus hypothetical gene supported by AK036103 (LOC330739), mR.			2.5	SM2081	a		1	1.656455229
mPool2_V2MM_111245	1.481108	3.004939	TRUE	HP_332262	TGCTGTTG	CTGATGAT	AK015052	RIKEN cDNA 4930402K13 gene	4930402K13Rik		2.7	SM2160	h		10	1.522164293
mPool7_V2MM_207952	1.481019	3.004939	TRUE	HP_425749	TGCTGTTG	GTCCAAGT	XM_162924	Mus musculus LOC243703 (LOC243703), mRNA.			2.1	SM2331	c		3	1.522164293
mPool7_V2MM_116587	1.480241	3.004939	TRUE	HP_337538	TGCTGTTG	GATGCCTT	XM_138996	Mus musculus LOC239041 (LOC239041), mRNA.			2.1	SM2303	h		8	1.522164293
092308m1_V2MM_153117	1.479187	3.004939	TRUE	HP_373778	TGCTGTTG	GGTGGTAT	XM_195745	Mus musculus similar to Rse H, putative [Plasmodium yoelii yoelii] (LOC270119), mR.			2.6	SM2125	a		2	1.522164293
mPool2_V2MM_107262	1.479027	3.004939	TRUE	HP_328351	TGCTGTTG	CCAATGAG	NM_027855	RIKEN cDNA 0610007C21 gene	0610007C21Rik		2.1	SM2323	d		10	1.522164293
092308m1_V2MM_136532	1.478567	3.004939	TRUE	HP_357252	TGCTGTTG	CAAATGAA	XM_151813	Mus musculus LOC213257 (LOC213257), mR.			2.4	SM2039	d		6	1.522164293
092308m1_V2MM_176912	1.477147	3.004939	TRUE	HP_397392	TGCTGTTG	CTGCTTATT	XM_287767	Mus musculus hypothetical gene supported by AK038473 (LOC330714), mR.			2.5	SM2066	f		1	1.522164293
092308m1_V2MM_80619	1.476967	3.004939	TRUE	HP_302246	TGCTGTTG	GGAATTGT	NM_025692	ubiquitin-activating enzyme E1-domain containing 1	Ube1dc1		2.4	SM2027	h		3	1.522164293
mPool7_V2MM_68711	1.476635	2.205692	TRUE	HP_290661	TGCTGTTG	GACTTTACT	NM_026173	RIKEN cDNA 1200014M14 gene	1200014M14Rik		2.8	SM2227	b		11	1.656455229
092308m3_V2MM_27443	1.476623	35.99881	TRUE	HP_250462	TGCTGTTG	GACACAAG	NM_030718	ABO blood group (transferase A, alpha 1-3-N-acetylgalactosan	Abo		2.12	SM2421	a		4	0.443711899
mPool2_V2MM_27473	1.476308	1.959154	TRUE	HP_250491	TGCTGTTG	GACAGATC	NM_133767	mitochondrial translational initiation factor 2	Mtif2		2.11	SM2389	c		11	1.707931489
mPool2_V2MM_215158	1.476302	3.004939	TRUE	HP_432719	TGCTGTTG	CTGGAATA	XM_146473	Mus musculus similar to nuclear autoantigenic sperm protein [Mus musculus] (LOC2346			2.11	SM2366	f		11	1.522164293
092308m1_V2MM_168196	1.476272	3.004939	TRUE	HP_388703	TGCTGTTG	CCTCCTGTA	XM_485762	similar to hypothetical protein C230069C04			2.6	SM2125	a		11	1.522164293
mPool4_V2MM_139312	1.47614	62.64426	TRUE	HP_360032	TGCTGTTG	GACTTTGA	XM_154921	Mus musculus LOC219168 (LOC219168), mRNA.			2.16	SM2635	a		3	0.203118686
mPool7_V2MM_98281	1.475935	3.004939	TRUE	HP_319512	TGCTGTTG	CAGGGAAA	XM_112336	Mus musculus LOC195594 (LOC195594), mRNA.			2.9	SM2270	g		1	1.522164293
mPool2_V2MM_117768	1.475755	2.205692	TRUE	NA	NA	NA	NA	NA	NA	NA	NA	NA	NA	NA		1.656455229
092308m1_V2MM_166043	1.47574	2.205692	TRUE	HP_386574	TGCTGTTG	CCTCGGACT	XM_285397	Mus musculus similar to KIAA1856 protein [Homo sapiens] (LOC329487), mR.			2.6	SM2115	b		8	1.656455229
mPool7_V2MM_65246	1.47525	2.205692	TRUE	HP_287287	TGCTGTTG	GCATATAT	AK085850	RIKEN cDNA D830016O14 gene	D830016O14Rik		2.8	SM2242	h		7	1.656455229
mPool2_V2MM_212572	1.475089	3.004939	TRUE	HP_430238	TGCTGTTG	CTAACTAA	XM_140908	ribosomal protein S27a	Rps27a		2.11	SM2369	d		5	1.522164293
mPool2_V2MM_127574	1.474393	2.205692	TRUE	HP_348398	TGCTGTTG	CTATTATG	XM_145254	RIKEN cDNA A630082K20 gene	A630082K20Rik		2.11	SM2354	b		3	1.656455229
mPool7_V2MM_68759	1.474098	3.004939	TRUE	HP_290705	TGCTGTTG	GAGAGAAT	NM_053117	par-6 partitioning defective 6 homolog gamma (C. elegans)	Pard6g		2.8	SM2231	c		6	1.522164293
mPool2_V2MM_189240	1.472754	2.205692	TRUE	HP_256004	TGCTGTTG	CACATTTCT	NM_146062	periphilin 1	Pphln1		2.11	SM2389	h		8	1.656455229
mPool2_V2MM_158160	1.47241	2.205692	TRUE	HP_378770	TGCTGTTG	GTGGTTAA	XM_206274	Mus musculus LOC278520 (LOC278520), mRNA.			2.6	SM2132	d		8	1.656455229
092308m3_V2MM_225744	1.471041	75.83547	TRUE	HP_477784	TGCTGTTG	GTGACTGT	NM_181753	opsin 5	Opn5		2.13	SM2468	b		11	0.120127608
092308m3_V2MM_233230	1.470003	75.83547	TRUE	HP_485149	TGCTGTTG	GCTTGTAA	XM_484981	D segment, Chr 2, Brigham & Womens Genetics 1335 express	D2Bwg1335e		2.13	SM2486	e		8	0.120127608
092308m1_V2MM_154518	1.469995	3.004939	TRUE	HP_375159	TGCTGTTG	CTCGTATTT	XM_196806	Mus musculus LOC270769 (LOC270769), mR.			2.6	SM2116	b		11	1.522164293
mPool7_V2MM_147548	1.469821	3.004939	TRUE	HP_368259	TGCTGTTG	CCACGTCA	XM_163078	Mus musculus LOC243797 (LOC243797), mRNA.			2.5	SM2052	c		6	1.522164293
mPool7_V2MM_147548	1.469821	3.004939	TRUE	HP_368259	TGCTGTTG	CCACGTCA	XM_163078	Mus musculus LOC243797 (LOC243797), mR.			2.5	SM2052	c		6	1.522164293
092308m3_V2MM_205360	1.469072	75.83547	TRUE	HP_423227	TGCTGTTG	CCGAGAAG	XM_132788	similar to KIAA0218			2.13	SM2489	c		11	0.120127608
092308m3_V2MM_100381	1.467128	62.64426	TRUE	HP_321572	TGCTGTTG	GCCTTCCC	AK006597	RIKEN cD 1700034J04 gene	1700034J04Rik		2.14	SM2501	a		1	0.203118686
092308m3_V2MM_98617	1.466784	75.83547	TRUE	HP_319843	TGCTGTTG	CAGATGCG	XM_112797	Mus musculus LOC215587 (LOC215587), mR.			2.9	SM2270	d		3	0.120127608
092308m3_V2MM_98617	1.466784	75.83547	TRUE	HP_319843	TGCTGTTG	CAGATGCG	XM_112797	Mus musculus LOC215587 (LOC215587), mRNA.			2.9	SM2270	d		3	0.120127608
mPool7_V2MM_103607	1.466715	2.205692	TRUE	HP_324745	TGCTGTTG	CCTGTATTT	XM_129159	RIKEN cDNA 1810073H04 gene	1810073H04Rik		2.1	SM2303	e		4	1.656455229
mPool2_V2MM_22454	1.466102	3.004939	TRUE	HP_245607	TGCTGTTG	CGTATTATT	NM_009285	stanniocalcin 1	Stc1		2.7	SM2165	g		9	1.522164293
mPool2_V2MM_115703	1.466051	3.004939	TRUE	HP_336664	TGCTGTTG	GACAGACA	XM_138607	similar to hypothetical protein FLJ30829; RNA-binding region (RNP1, RRM) containing 6			2.7	SM2155	h		10	1.522164293
mPool2_V2MM_109890	1.465927	3.004939	TRUE	HP_330936	TGCTGTTG	GAAACATG	XM_486116	cDNA sequence D10627	D10627		2.7	SM2163	h		11	1.522164293

mPool7_V2MM_100109	1.464743	3.004939	TRUE	HP_321302	TGCTGTTG	CATTTCTAT	NM_009546	tripartite motif protein 25	Trim25		2.7	SM2158	g		10	1.522164293
mPool7_V2MM_85492	1.46439	3.004939	TRUE	HP_306973	TGCTGTTG	CAATACTCT	NM_146395	olfactory receptor 1276	Olfr1276		2.8	SM2249	a		10	1.522164293
mPool7_V2MM_177132	1.464243	2.205692	TRUE	HP_397612	TGCTGTTG	CGAATTTG	XM_287810	Mus musculus hypothetical gene supported by AK049065 (LOC330779), mR.			2.5	SM2087	d		7	1.656455229
mPool7_V2MM_177132	1.464243	2.205692	TRUE	HP_397612	TGCTGTTG	CGAATTTG	XM_287810	Mus musculus hypothetical gene supported by AK049065 (LOC330779), mRNA.			2.5	SM2087	d		7	1.656455229
092308m1_V2MM_126100	1.463406	3.004939	TRUE	HP_346943	TGCTGTTG	GTGGCAAA	XM_144377	Mus musculus similar to crumbs homolog 1 [Mus musculus] (LOC231351), mR.			2.6	SM2103	a		4	1.522164293
mPool2_V2MM_116096	1.463322	2.205692	TRUE	HP_337051	TGCTGTTG	CTGATATT	XM_138748	Mus musculus similar to envelope protein [Ovis aries] (LOC218362), mRNA.			2.6	SM2150	c		2	1.656455229
mPool4_V2MM_21536	1.462926	61.29828	TRUE	HP_244707	TGCTGTTG	CAGTTCTG	NM_148931	solute carrier family 6 (neurotransmitter transporter, glycine)	Slc6a5		2.15	SM2599	e		2	0.212551731
092308m1_V2MM_181542	1.462457	3.004939	TRUE	HP_402016	TGCTGTTG	CGAAGGAG	XM_288693	Mus musculus LOC332121 (LOC332121), mR.			2.5	SM2060	d		4	1.522164293
092308m1_V2MM_183622	1.460482	3.004939	TRUE	HP_404096	TGCTGTTG	CTTTAAGA	XM_289051	similar to bM64F17.3 (putative novel protein)			2.5	SM2069	e		2	1.522164293
mPool4_V2MM_213671	1.459972	60.13437	TRUE	HP_431293	TGCTGTTG	CAATAGTG	XM_140140	Mus musculus similar to uracil DNA glycosylase [Homo sapiens] (LOC225099), mRNA.			2.14	SM2544	f		2	0.220877236
092308m1_V2MM_149043	1.459215	3.004939	TRUE	HP_369754	TGCTGTTG	CTGCCTGT	XM_164432	Mus musculus LOC244522 (LOC244522), mR.			2.5	SM2057	f		12	1.522164293
mPool2_V2MM_123300	1.459045	2.205692	TRUE	HP_344156	TGCTGTTG	GATAAGAC	XM_142871	Mus musculus similar to hypothetical protein FLJ90396 [Homo sapiens] (LOC236334), m			2.11	SM2352	f		6	1.656455229
092308m1_V2MM_177871	1.456781	2.205692	TRUE	NA	NA	NA	NA	NA	NA	NA		NA	NA	NA		1.656455229
092308m1_V2MM_170974	1.456765	2.205692	TRUE	HP_391464	TGCTGTTG	CCTTTACTC	XM_286529	gene model 1576, (NCBI)	Gm1576		2.5	SM2093	g		11	1.656455229
092308m1_V2MM_170974	1.456765	2.205692	TRUE	HP_391464	TGCTGTTG	CCTTTACTC	XM_286529	gene model 1576, (NCBI)	Gm1576		2.5	SM2093	g		11	1.656455229
mPool7_V2MM_76262	1.456687	3.004939	TRUE	HP_298026	TGCTGTTG	CGATCATT	NM_025422	RIKEN cDNA 1110055L24 gene	1110055L24Rik		2.8	SM2227	g		1	1.522164293
mPool2_V2MM_107397	1.45601	3.004939	TRUE	HP_328481	TGCTGTTG	GCTCTAGT	AK075654	RIKEN cDNA 3830408G10 gene	3830408G10Rik		2.7	SM2156	e		7	1.522164293
mPool2_V2MM_145046	1.455564	3.004939	TRUE	HP_365757	TGCTGTTG	CTGGTATT	XM_160513	Mus musculus LOC215240 (LOC215240), mRNA.			2.1	SM2340	e		8	1.522164293
092308m1_V2MM_92238	1.455531	3.004939	TRUE	HP_313597	TGCTGTTG	CTAACTAG	NM_178674	F-box and leucine-rich repeat protein 21	Fbxl21		2.6	SM2112	b		12	1.522164293
092308m1_V2MM_131488	1.455522	2.205692	TRUE	HP_352251	TGCTGTTG	CTAGGAGA	AK020496	RIKEN cD 9430078K24 gene	9430078K24Rik		2.5	SM2097	d		5	1.656455229
092308m1_V2MM_176622	1.454717	2.205692	TRUE	HP_397102	TGCTGTTG	CCTGTATTC	XM_287707	Mus musculus hypothetical gene supported by AK045013 (LOC330567), mR.			2.5	SM2065	a		6	1.656455229
092308m3_V2MM_202739	1.452713	62.64426	TRUE	HP_420729	TGCTGTTG	CCACAGAA	XM_132795	gene model 154, (NCBI)	Gm154		2.13	SM2494	e		1	0.203118686
mPool2_V2MM_83636	1.452372	3.004939	TRUE	HP_305166	TGCTGTTG	CTCATTGA	NM_023127	polymerase (RNA) II (DNA directed) polypeptide K	Polr2k		2.6	SM2140	e		7	1.522164293
mPool7_V2MM_173969	1.451997	3.004939	TRUE	HP_394455	TGCTGTTG	CCTAGTGT	XM_287126	Mus musculus hypothetical gene supported by AK082531 (LOC329411), mRNA.			2.9	SM2278	f		1	1.522164293
mPool2_V2MM_42767	1.451741	3.004939	TRUE	HP_265370	TGCTGTTG	CGCATTGG	NM_009692	apolipoprotein A-I	Apoa1		2.7	SM2165	g		12	1.522164293
mPool2_V2MM_14313	1.451211	3.004939	TRUE	HP_237663	TGCTGTTG	CCGCTTCTT	NM_019394	melanoma inhibitory activity 1	Mia1		2.11	SM2381	h		12	1.522164293
092308m1_V2MM_173934	1.450953	2.205692	TRUE	HP_394420	TGCTGTTG	CTATATCA	XM_287120	Mus musculus hypothetical gene supported by AK034841 (LOC329386), mR.			2.5	SM2069	e		7	1.656455229
092308m1_V2MM_173934	1.450953	2.205692	TRUE	HP_394420	TGCTGTTG	CTATATCA	XM_287120	Mus musculus hypothetical gene supported by AK034841 (LOC329386), mRNA.			2.5	SM2069	e		7	1.656455229
mPool6_V2MM_73675	1.450365	75.83547	TRUE	HP_125750	TGCTGTTG	GGTGTTCA	NM_025739	RIKEN cDNA 4931406I20 gene	4931406I20Rik		2.7	SM2173	b		4	0.120127608
mPool4_V2MM_37040	1.449677	75.83547	TRUE	HP_259811	TGCTGTTG	GAATCTGT	NM_031385	testis expressed gene 18	Tex18		2.15	SM2579	g		8	0.120127608
092308m1_V2MM_63029	1.448431	3.004939	TRUE	HP_285119	TGCTGTTG	CCGGATGT	NM_030237	spermatogenic Zip 1			2.3	SM2015	h		6	1.522164293
092308m1_V2MM_151907	1.448138	3.004939	TRUE	HP_372574	TGCTGTTG	GGCGTTTA	XM_194993	olfactory receptor 171	Olfr171		2.4	SM2040	b		5	1.522164293
mPool7_V2MM_95356	1.447855	3.004939	TRUE	HP_316632	TGCTGTTG	CTGTTTGTT	NM_197985	adiponectin receptor 2	Adipor2		2.9	SM2255	a		10	1.522164293
mPool5_V2MM_7657	1.447542	75.83547	TRUE	HP_231160	TGCTGTTG	GCCACAGT	NM_008170	glutamate receptor, ionotropic, NMDA2A (epsilon 1)	Grin2a	NA				NA		0.120127608
mPool2_V2MM_21718	1.447502	2.205692	TRUE	HP_244886	TGCTGTTG	CATTGAGC	NM_027564	RIKEN cDNA 4921507P07 gene	4921507P07Rik		2.11	SM2387	g		9	1.656455229
092308m1_V2MM_125765	1.447302	3.004939	TRUE	HP_346608	TGCTGTTG	CCAGTCTA	XM_144221	similar to hypothetical protein MGC19022			2.6	SM2111	g		1	1.522164293
092308m1_V2MM_125765	1.447302	3.004939	TRUE	HP_346608	TGCTGTTG	CCAGTCTA	XM_144221	similar to hypothetical protein MGC19022			2.6	SM2111	g		1	1.522164293
mPool6_V2MM_77610	1.44722	60.13437	TRUE	HP_299325	TGCTGTTG	CTGTGTGA	NM_025482	tumor protein D52-like 2	Tpd52l2		2.8	SM2222	a		4	0.220877236
092308m3_V2MM_112366	1.447196	69.72441	TRUE	HP_333371	TGCTGTTG	CCTTTGAT	XM_136450	Mus musculus similar to glyceraldehyde-3-phosphate dehydrogese [Rattus norvegicus] (			2.13	SM2497	h		10	0.156615137
mPool7_V2MM_104352	1.446502	3.004939	TRUE	HP_325486	TGCTGTTG	CCTATTGC	AK077997	RIKEN cDNA 6130401J04 gene	6130401J04Rik		2.7	SM2157	e		2	1.522164293
092308m1_V2MM_138711	1.446331	3.004939	TRUE	HP_359431	TGCTGTTG	CGAAATAT	XM_154438	Mus musculus LOC238835 (LOC238835), mRNA.			2.4	SM2045	d		6	1.522164293
092308m1_V2MM_138711	1.446331	3.004939	TRUE	HP_359431	TGCTGTTG	CGAAATAT	XM_154438	Mus musculus LOC238835 (LOC238835), mR.			2.4	SM2045	d		6	1.522164293
mPool7_V2MM_179050	1.446286	3.004939	TRUE	HP_399524	TGCTGTTG	CCAGTTTG	XM_288266	Mus musculus LOC332539 (LOC332539), mRNA.			2.5	SM2070	b		6	1.522164293
mPool7_V2MM_179050	1.446286	3.004939	TRUE	HP_399524	TGCTGTTG	CCAGTTTG	XM_288266	Mus musculus LOC332539 (LOC332539), mR.			2.5	SM2070	b		6	1.522164293
092308m3_V2MM_149943	1.445488	75.83547	TRUE	HP_370653	TGCTGTTG	CCACGAAT	XM_165354	Mus musculus LOC214929 (LOC214929), mR.			2.14	SM2528	h		11	0.120127608
mPool2_V2MM_9944	1.445152	3.004939	TRUE	HP_233389	TGCTGTTG	CGCTTTATC	NM_011677	uracil-DNA glycosylase	Ung		2.11	SM2379	h		4	1.522164293
mPool4_V2MM_262310	1.444862	75.83547	TRUE	HP_521401	TGCTGTTG	GTGTCTGT	NM_203507	cDNA sequence BC016198	BC016198		2.16	SM2617	h		3	0.120127608
mPool2_V2MM_9907	1.443454	3.004939	TRUE	HP_233354	TGCTGTTG	CGCCGTAA	NM_011643	transient receptor potential cation channel, subfamily C, mem	Trpc1		2.11	SM2385	a		4	1.522164293
mPool4_V2MM_16028	1.443318	75.83547	TRUE	HP_239335	TGCTGTTG	CTGTGTGT	NM_198412	DnaJ (Hsp40) homolog, subfamily C, member 6	Dnajc6	NA				NA		0.120127608
mPool2_V2MM_106877	1.442866	3.004939	TRUE	HP_327970	TGCTGTTG	CATACATC	XM_131619	cDNA sequence BC022150	BC022150		2.7	SM2159	c		12	1.522164293
092308m1_V2MM_77283	1.442456	3.004939	TRUE	HP_299004	TGCTGTTG	CTGCCATCT	NM_026015	RIKEN cD 2610510L01 gene	2610510L01Rik		2.3	SM2016	b		8	1.522164293
mPool2_V2MM_211496	1.441848	3.004939	TRUE	HP_429192	TGCTGTTG	GATGAGCT	XM_156070	similar to stefin A2			2.11	SM2372	b		7	1.522164293
092308m3_V2MM_226323	1.440703	75.83547	TRUE	HP_478351	TGCTGTTG	GGAGAAAT	NM_175029	APG4 (ATG4) autophagy-related homolog C (S. cerevisiae)	Apg4c		2.13	SM2473	g		2	0.120127608
mPool5_V2MM_173749	1.440344	70.87591	TRUE	HP_394235	TGCTGTTG	CAGGCTGG	XM_287087	Mus musculus hypothetical gene supported by AK083495 (LOC329349), mRNA.		NA				NA		0.149501346
092308m3_V2MM_188570	1.440253	75.83547	TRUE	HP_321924	TGCTGTTG	CAAATTAG	NM_011344	Sel1 (suppressor of lin-12) 1 homolog (C. elegans)	Sel1h		2.7	SM2168	h		10	0.120127608
092308m3_V2MM_188570	1.440253	75.83547	TRUE	HP_321924	TGCTGTTG	CAAATTAG	NM_011344	Sel1 (suppressor of lin-12) 1 homolog (C. elegans)	Sel1h		2.7	SM2168	h		10	0.120127608
mPool4_V2MM_239018	1.440179	60.13437	TRUE	HP_490837	TGCTGTTG	GTATATTT	XM_143027	Mus musculus similar to envelope protein [Trichosurus vulpecula] (LOC236517), mRNA.			2.14	SM2548	h		2	0.220877236
092308m1_V2MM_156660	1.439884	2.205692	TRUE	HP_377289	TGCTGTTG	CCACTTTCT	AK013636	RIKEN cD 2900042B11 gene	2900042B11Rik		2.6	SM2117	c		7	1.656455229
mPool6_V2MM_76955	1.438621	75.83547	TRUE	HP_298688	TGCTGTTG	CTCGTGGC	NM_021791	double C2, gamma	Doc2g		2.7	SM2172	f		11	0.120127608
mPool7_V2MM_177678	1.437435	2.205692	TRUE	HP_398158	TGCTGTTG	GGATTATT	XM_287927	Mus musculus hypothetical gene supported by AK035601 (LOC330954), mRNA.			2.5	SM2067	h		11	1.656455229
mPool7_V2MM_177678	1.437435	2.205692	TRUE	HP_398158	TGCTGTTG	GGATTATT	XM_287927	Mus musculus hypothetical gene supported by AK035601 (LOC330954), mR.			2.5	SM2067	h		11	1.656455229
mPool7_V2MM_171932	1.437425	3.004939	TRUE	HP_392418	TGCTGTTG	CCCTTAACA	XM_286725	Mus musculus hypothetical gene supported by AK036009 (LOC328675), mR.			2.5	SM2088	f		9	1.522164293

mPool7_V2MM_171932	1.437425	3.004939	TRUE	HP_392418	TGCTGTTG/	CCCTTAACA	XM_286725	Mus musculus hypothetical gene supported by AK036009 (LOC328675), mRNA.		2.5	SM2088	f	9	1.522164293
mPool2_V2MM_8277	1.436967	3.004939	TRUE	HP_231765	TGCTGTTG/	GTAAGTGT	NM_028060	solute carrier family 35, member F2	Slc35f2	2.11	SM2386	b	7	1.522164293
092308m3_V2MM_144130	1.436502	75.83547	TRUE	HP_364841	TGCTGTTG/	CTTGGCCA/	XM_159764	Mus musculus LOC241881 (LOC241881), mR.		2.14	SM2526	e	3	0.120127608
mPool4_V2MM_78137	1.436109	60.13437	TRUE	HP_299836	TGCTGTTG/	GAATGGAT	NM_025504	RIKEN cDNA 2310004L02 gene	2310004L02Rik	2.15	SM2588	f	11	0.220877236
092308m1_V2MM_117818	1.436038	3.004939	TRUE	HP_338758	TGCTGTTG/	CAGAAAGT	XM_139540	PDZ domain containing RING finger 4		2.6	SM2104	d	1	1.522164293
mPool2_V2MM_22530	1.435996	3.004939	TRUE	HP_245680	TGCTGTTG/	CTAACCATT	NM_030705	mesoderm development candidate 1	Mesdc1	2.11	SM2390	c	8	1.522164293
092308m1_V2MM_187836	1.43561	2.205692	TRUE	HP_408300	TGCTGTTG/	CTGAAATT(	XM_290161	Mus musculus LOC333779 (LOC333779), mRNA.		2.5	SM2094	h	2	1.656455229
092308m1_V2MM_187836	1.43561	2.205692	TRUE	HP_408300	TGCTGTTG/	CTGAAATT(	XM_290161	Mus musculus LOC333779 (LOC333779), mR.		2.5	SM2094	h	2	1.656455229
mPool6_V2MM_193327	1.435352	75.83547	TRUE	HP_411790	TGCTGTTG/	CCGTTCTGT	NM_001006680	potassium voltage-gated channel, subfamily Q, member 2	Kcnq2	2.7	SM2180	f	3	0.120127608
092308m3_V2MM_55020	1.435206	66.95036	TRUE	HP_277327	TGCTGTTG/	CCCAGGAA	AK129138	zinc finger protein 96	Zfp96	2.12	SM2446	h	4	0.1742471
092308m1_V2MM_176179	1.435037	2.205692	TRUE	HP_396661	TGCTGTTG/	GGAAAGTA	AK046665	RIKEN cD 9530026P05 gene	9530026P05Rik	2.5	SM2072	f	9	1.656455229
mPool2_V2MM_3528	1.434641	3.004939	TRUE	HP_227121	TGCTGTTG/	GATAGGAA	NM_026742	RIKEN cDNA 1110007M04 gene	1110007M04Rik	2.11	SM2375	f	9	1.522164293
mPool4_V2MM_213659	1.434521	66.95036	TRUE	HP_431281	TGCTGTTG/	GGAGCCTA	XM_147019	Mus musculus similar to WD40-repeat type I transmembrane protein A72.5 [Mus muscu		2.16	SM2631	d	12	0.1742471
092308m3_V2MM_40374	1.43424	60.13437	TRUE	HP_263040	TGCTGTTG/	GTCCCTGG(	NM_009289	STE20-like kise (yeast)	Slk	2.12	SM2424	c	10	0.220877236
092308m1_V2MM_137283	1.433549	3.004939	TRUE	HP_358003	TGCTGTTG/	CAACCAAC/	XM_152972	Mus musculus LOC237973 (LOC237973), mRNA.		2.5	SM2053	f	4	1.522164293
092308m1_V2MM_137283	1.433549	3.004939	TRUE	HP_358003	TGCTGTTG/	CAACCAAC/	XM_152972	Mus musculus LOC237973 (LOC237973), mR.		2.5	SM2053	f	4	1.522164293
092308m3_V2MM_104323	1.433405	75.83547	TRUE	HP_325457	TGCTGTTG/	CTTACAAA(	AK036315	RIKEN cDNA 9630055N22 gene	9630055N22Rik	2.1	SM2304	a	12	0.120127608
092308m3_V2MM_104323	1.433405	75.83547	TRUE	HP_325457	TGCTGTTG/	CTTACAAA(	AK036315	RIKEN cD 9630055N22 gene	9630055N22Rik	2.1	SM2304	a	12	0.120127608
092308m1_V2MM_147733	1.430171	3.004939	TRUE	HP_368444	TGCTGTTG/	CAATGGAT/	XM_163222	similar to PIN4 protein		2.4	SM2044	f	3	1.522164293
mPool4_V2MM_105972	1.42983	75.83547	TRUE	HP_327079	TGCTGTTG/	CTGCTGCT(	BC025640	pygopus 2	Pygo2	2.16	SM2610	h	2	0.120127608
092308m1_V2MM_131449	1.429086	3.004939	TRUE	HP_352212	TGCTGTTG/	CCCAGTCT(	XM_488636	hypothetical gene supported by AK048895		2.5	SM2099	e	9	1.522164293
mPool4_V2MM_45737	1.427752	70.87591	TRUE	HP_268260	TGCTGTTG/	CACCCTGG/	NM_173398	G protein-coupled receptor 171	Gpr171	2.16	SM2607	c	7	0.149501346
mPool2_V2MM_96077	1.427742	3.004939	TRUE	HP_317341	TGCTGTTG/	GCCCTGAA/	BC011440	histone 1, H2bc	Hist1h2bc	2.6	SM2137	h	6	1.522164293
mPool7_V2MM_90036	1.427581	3.004939	TRUE	HP_311462	TGCTGTTG/	CTAGATGA(	NM_177718	hypothetical protein A430031N04		2.9	SM2253	c	3	1.522164293
mPool2_V2MM_150932	1.427536	3.004939	TRUE	HP_371609	TGCTGTTG/	CAGGATGA	NM_011090	paired-Ig-like receptor A3	Pira3	2.4	SM2039	h	1	1.522164293
mPool2_V2MM_150932	1.427536	3.004939	TRUE	HP_371609	TGCTGTTG/	CAGGATGA	NM_011090	paired-Ig-like receptor A3	Pira3	2.4	SM2039	h	1	1.522164293
092308m1_V2MM_159051	1.42726	3.004939	TRUE	HP_379653	TGCTGTTG/	CAGCAGAA	XM_282962	Mus musculus hypothetical gene supported by AK029003 (LOC327858), mR.		2.6	SM2119	h	7	1.522164293
092308m1_V2MM_172934	1.426825	3.004939	TRUE	HP_393420	TGCTGTTG/	CAGGGTCA	XM_286925	Mus musculus hypothetical gene supported by AK083639 (LOC329014), mRNA.		2.5	SM2069	d	8	1.522164293
092308m1_V2MM_172934	1.426825	3.004939	TRUE	HP_393420	TGCTGTTG/	CAGGGTCA	XM_286925	Mus musculus hypothetical gene supported by AK083639 (LOC329014), mR.		2.5	SM2069	d	8	1.522164293
mPool2_V2MM_210580	1.426637	3.004939	TRUE	HP_428326	TGCTGTTG/	CCATTTCCT	AK080244	RIKEN cDNA A530088I07 gene	A530088I07Rik	2.1	SM2348	h	8	1.522164293
mPool4_V2MM_4871	1.426268	75.83547	TRUE	HP_228438	TGCTGTTG/	CAGATACT(	NM_053271	regulating synaptic membrane exocytosis 2	Rims2	2.15	SM2598	b	5	0.120127608
092308m1_V2MM_171932	1.425525	1.959154	TRUE	HP_392418	TGCTGTTG/	CCCTTAACA	XM_286725	Mus musculus hypothetical gene supported by AK036009 (LOC328675), mR.		2.5	SM2088	f	9	1.707931489
092308m1_V2MM_171932	1.425525	1.959154	TRUE	HP_392418	TGCTGTTG/	CCCTTAACA	XM_286725	Mus musculus hypothetical gene supported by AK036009 (LOC328675), mRNA.		2.5	SM2088	f	9	1.707931489
092308m1_V2MM_159279	1.42511	3.004939	TRUE	HP_379876	TGCTGTTG/	GGTTAAGG	AK036657	RIKEN cD 5830411O07 gene	5830411O07Rik	2.6	SM2114	h	12	1.522164293
mPool7_V2MM_103050	1.424667	3.004939	TRUE	HP_324195	TGCTGTTG/	CTATTGAA/	XM_128767	RIKEN cDNA 4921513D11 gene	4921513D11Rik	2.9	SM2300	f	9	1.522164293
mPool2_V2MM_85970	1.424203	2.205692	TRUE	HP_307450	TGCTGTTG/	CTCAAATGT	NM_146533	olfactory receptor 1367	Olfr1367	2.6	SM2144	d	4	1.656455229
mPool7_V2MM_101698	1.423955	2.205692	TRUE	HP_322864	TGCTGTTG/	GTGGAGAT	XM_127811	RIKEN cDNA 1700020M16 gene	1700020M16Rik	2.9	SM2299	f	12	1.656455229
092308m3_V2MM_224880	1.422472	75.83547	TRUE	HP_476941	TGCTGTTG/	GCCGAAAT(	NM_053157	beta-amyloid binding protein precursor	Bbp	2.12	SM2450	b	12	0.120127608
092308m3_V2MM_101053	1.420914	75.83547	TRUE	HP_322228	TGCTGTTG/	GACCTCAT(	NM_175150	RIKEN cD 2310047H23 gene	2310047H23Rik	2.1	SM2308	c	1	0.120127608
092308m3_V2MM_101053	1.420914	75.83547	TRUE	HP_322228	TGCTGTTG/	GACCTCAT(	NM_175150	RIKEN cDNA 2310047H23 gene	2310047H23Rik	2.1	SM2308	c	1	0.120127608
092308m1_V2MM_69950	1.420911	2.205692	TRUE	HP_291873	TGCTGTTG/	GTGAAATT(	NM_025626	RIKEN cD 3110001A13 gene	3110001A13Rik	2.4	SM2020	d	7	1.656455229
092308m3_V2MM_209057	1.420835	61.29828	TRUE	HP_426833	TGCTGTTG/	CTGCTCAG(	XM_156248	Mus musculus LOC224196 (LOC224196), mRNA.		2.1	SM2332	d	9	0.212551731
092308m3_V2MM_209057	1.420835	61.29828	TRUE	HP_426833	TGCTGTTG/	CTGCTCAG(	XM_156248	Mus musculus LOC224196 (LOC224196), mR.		2.1	SM2332	d	9	0.212551731
mPool4_V2MM_90391	1.420092	69.72441	TRUE	HP_311811	TGCTGTTG/	GCTTCATG(	NM_177787	hypothetical protein 9830102E05		2.16	SM2626	d	8	0.156615137
mPool2_V2MM_7999	1.419749	2.205692	TRUE	HP_59095	TGCTGTTG/	GCTATAGT(	NM_030675	cerebral cavernous malformations 1	Ccm1	2.11	SM2381	a	2	1.656455229
092308m1_V2MM_156212	1.419701	3.004939	TRUE	HP_376851	TGCTGTTG/	CCTTGAAG/	XM_198207	Mus musculus LOC272573 (LOC272573), mR.		2.6	SM2116	g	1	1.522164293
092308m1_V2MM_169663	1.419463	3.004939	TRUE	HP_390158	TGCTGTTG/	CTACTGAT(	AK039689	hypothetical protein A330087I24		2.6	SM2116	a	7	1.522164293
mPool5_V2MM_8062	1.419216	75.83547	TRUE	HP_231553	TGCTGTTG/	GCTGGATG	L42339	sodium channel, voltage-gated, type I, alpha polypeptide	Scn1a	NA			NA	0.120127608
mPool7_V2MM_81537	1.419145	2.205692	TRUE	HP_303141	TGCTGTTG/	GTCCTAAAT	NM_176967	RIKEN cDNA A430072C10 gene	A430072C10Rik	2.8	SM2243	f	8	1.656455229
mPool2_V2MM_208283	1.419128	2.205692	TRUE	HP_426076	TGCTGTTG/	CTACTAGG(	XM_154578	Mus musculus LOC218719 (LOC218719), mRNA.		2.1	SM2342	h	2	1.656455229
092308m1_V2MM_141358	1.418852	2.205692	TRUE	HP_362071	TGCTGTTG/	GTGGGTAC	XM_156600	Mus musculus LOC239924 (LOC239924), mR.		2.4	SM2046	a	7	1.656455229
mPool2_V2MM_9686	1.418838	3.004939	TRUE	HP_233141	TGCTGTTG/	CATCTATTC	NM_007856	7-dehydrocholesterol reductase	Dhcr7	2.11	SM2382	h	2	1.522164293
mPool4_V2MM_50742	1.418562	75.83547	TRUE	HP_273155	TGCTGTTG/	CATTCAAT(	NM_177593	hypothetical protein MGC48079		2.16	SM2630	a	10	0.120127608
mPool7_V2MM_174322	1.41812	3.004939	TRUE	HP_394807	TGCTGTTG/	GCATCCAA/	XM_287205	Mus musculus hypothetical gene supported by AK037602 (LOC329578), mR.		2.5	SM2063	h	9	1.522164293
mPool7_V2MM_174322	1.41812	3.004939	TRUE	HP_394807	TGCTGTTG/	GCATCCAA/	XM_287205	Mus musculus hypothetical gene supported by AK037602 (LOC329578), mRNA.		2.5	SM2063	h	9	1.522164293
092308m1_V2MM_159103	1.418021	2.205692	TRUE	HP_379704	TGCTGTTG/	CTGCTAAC/	XM_282974	gene model 739, (NCBI)	Gm739	2.6	SM2115	c	5	1.656455229
mPool4_V2MM_103	1.417965	73.80895	TRUE	HP_223785	TGCTGTTG/	CAACCAGA	NM_030558	carbonic anhydrase 15	Car15	2.15	SM2580	e	7	0.131891001
092308m1_V2MM_144203	1.416695	3.004939	TRUE	HP_364914	TGCTGTTG/	GTCCCTTCA	XM_159803	Mus musculus LOC207831 (LOC207831), mR.		2.5	SM2057	g	5	1.522164293
mPool4_V2MM_217576	1.416598	75.83547	TRUE	HP_450649	TGCTGTTG/	GTGTTTCTT	NM_011966	proteasome (prosome, macropain) subunit, alpha type 4	Psma4	2.15	SM2574	e	6	0.120127608
mPool4_V2MM_188490	1.416037	59.99801	TRUE	HP_298500	TGCTGTTG/	CTCAAGTT(	NM_010550	interleukin 11 receptor, alpha chain 2	Il11ra2	2.15	SM2571	a	12	0.221863149
mPool2_V2MM_202759	1.415503	2.205692	TRUE	HP_420747	TGCTGTTG/	CCAGAACA	XM_137990	Mus musculus similar to glyceraldehyde-3-phosphate dehydrogenase (phosphorylating)		2.1	SM2324	b	5	1.656455229
092308m3_V2MM_93509	1.415109	66.95036	TRUE	HP_314823	TGCTGTTG/	GCTGCTAG(	NM_181413	ankyrin repeat and SAM domain containing 1	Anks1	2.13	SM2458	f	10	0.1742471

mPool7_V2MM_172037	1.413912	2.205692	TRUE	HP_392523	TGCTGTTG	GGAAGAG	XM_286742	gene model 813, (NCBI)	Gm813		2.9	SM2273	d		12	1.656455229
092308m3_V2MM_58014	1.41357	75.83547	TRUE	HP_280255	TGCTGTTG	GACTGGTT	NM_008866	lysophospholipase 1	Lypla1		2.12	SM2445	a		8	0.120127608
mPool7_V2MM_174595	1.413392	3.004939	TRUE	HP_395080	TGCTGTTG	CTCCTTAAC	XM_287259	Mus musculus hypothetical gene supported by AK053945 (LOC329683), mR.			2.5	SM2068	f		8	1.522164293
mPool7_V2MM_174595	1.413392	3.004939	TRUE	HP_395080	TGCTGTTG	CTCCTTAAC	XM_287259	Mus musculus hypothetical gene supported by AK053945 (LOC329683), mRNA.			2.5	SM2068	f		8	1.522164293
092308m3_V2MM_86500	1.412265	60.13437	TRUE	HP_307974	TGCTGTTG	CAGTCCAT	NM_172675	syntaxin 16	Stx16		2.13	SM2460	d		4	0.220877236
mPool2_V2MM_149801	1.411882	3.004939	TRUE	HP_370511	TGCTGTTG	CATTGATG	XM_165199	Mus musculus LOC209819 (LOC209819), mRNA.			2.1	SM2344	f		1	1.522164293
mPool2_V2MM_128720	1.411333	3.004939	TRUE	HP_349533	TGCTGTTG	GTGTTCTTT	XM_145886	gene model 491, (NCBI)	Gm491		2.11	SM2370	h		7	1.522164293
092308m3_V2MM_102242	1.411324	75.83547	TRUE	HP_323398	TGCTGTTG	GCCGTGGT	XM_128190	DEAD (Asp-Glu-Ala-Asp) box polypeptide 23	Ddx23		2.13	SM2500	d		2	0.120127608
mPool2_V2MM_13293	1.411054	3.004939	TRUE	HP_236663	TGCTGTTG	CAGTGAAC	NM_053229	vomeronasal 1 receptor, B8	V1rb8		2.11	SM2375	e		11	1.522164293
mPool2_V2MM_83233	1.410856	3.004939	TRUE	HP_304781	TGCTGTTG	CTGTAAGT	AK041435	utrophin	Utrn		2.6	SM2144	f		2	1.522164293
mPool7_V2MM_68027	1.41072	3.004939	TRUE	HP_289998	TGCTGTTG	CTGATTCTA	AK031552	RIKEN cDNA 4732447D17 gene	4732447D17Rik		2.8	SM2235	e		6	1.522164293
092308m3_V2MM_54987	1.410596	75.83547	TRUE	HP_277296	TGCTGTTG	CCCACTTTG	NM_153540	expressed sequence C85492	C85492		2.12	SM2414	c		12	0.120127608
092308m1_V2MM_146127	1.410463	2.205692	TRUE	HP_366838	TGCTGTTG	CAGCTTAT	XM_161773	hypothetical gene supported by AK036869			2.4	SM2050	a		1	1.656455229
092308m1_V2MM_146127	1.410463	2.205692	TRUE	HP_366838	TGCTGTTG	CAGCTTAT	XM_161773	hypothetical gene supported by AK036869			2.4	SM2050	a		1	1.656455229
092308m3_V2MM_56950	1.410207	75.83547	TRUE	HP_279219	TGCTGTTG	CTGGCTCT	NM_017392	cadherin EGF LAG seven-pass G-type receptor 2	Celsr2		2.12	SM2439	c		8	0.120127608
092308m1_V2MM_166469	1.408364	3.004939	TRUE	HP_386997	TGCTGTTG	CAGGATAT	XM_285507	Mus musculus similar to nucleosome assembly protein 1-like 2; brain specific gene BPX			2.6	SM2116	f		10	1.522164293
mPool7_V2MM_66479	1.408283	2.205692	TRUE	HP_288492	TGCTGTTG	CAGCAAAG	NM_009836	chaperonin subunit 3 (gamma)	Cct3		2.8	SM2228	a		9	1.656455229
mPool2_V2MM_109720	1.407546	3.004939	TRUE	HP_330773	TGCTGTTG	GACATGGG	XM_134085	RIKEN cDNA 1700083M11 gene	1700083M11Rik		2.1	SM2312	g		5	1.522164293
mPool2_V2MM_109720	1.407546	3.004939	TRUE	HP_330773	TGCTGTTG	GACATGGG	XM_134085	RIKEN cD 1700083M11 gene	1700083M11Rik		2.1	SM2312	g		5	1.522164293
mPool7_V2MM_79534	1.406818	3.004939	TRUE	HP_301190	TGCTGTTG	GCATATTC	NM_029460	RIKEN cDNA 1700108E19 gene	1700108E19Rik		2.8	SM2248	b		7	1.522164293
092308m3_V2MM_194961	1.406358	75.83547	TRUE	HP_413111	TGCTGTTG	GGCCGTAA	XM_111160	Mus musculus similar to eukaryotic translation initiation factor 4A1; initiation factor eIF			2.9	SM2252	b		3	0.120127608
092308m3_V2MM_194961	1.406358	75.83547	TRUE	HP_413111	TGCTGTTG	GGCCGTAA	XM_111160	Mus musculus similar to eukaryotic translation initiation factor 4A1; initiation factor eIF			2.9	SM2252	b		3	0.120127608
mPool7_V2MM_170974	1.405913	2.205692	TRUE	HP_391464	TGCTGTTG	CCTTTACTC	XM_286529	gene model 1576, (NCBI)	Gm1576		2.5	SM2093	g		11	1.656455229
mPool7_V2MM_170974	1.405913	2.205692	TRUE	HP_391464	TGCTGTTG	CCTTTACTC	XM_286529	gene model 1576, (NCBI)	Gm1576		2.5	SM2093	g		11	1.656455229
mPool2_V2MM_83061	1.404691	3.004939	TRUE	HP_304615	TGCTGTTG	CACAGAAC	NM_011278	ring finger protein 4	Rnf4		2.6	SM2133	a		8	1.522164293
mPool7_V2MM_200241	1.40469	3.004939	TRUE	HP_418278	TGCTGTTG	GGCATATT	XM_288127	Mus musculus hypothetical gene supported by AK053873 (LOC331495), mRNA.			2.9	SM2276	h		9	1.522164293
mPool2_V2MM_144230	1.404636	3.004939	TRUE	HP_364941	TGCTGTTG	CATTGTAC	XM_159825	Mus musculus LOC207745 (LOC207745), mRNA.			2.5	SM2058	a		5	1.522164293
mPool2_V2MM_144230	1.404636	3.004939	TRUE	HP_364941	TGCTGTTG	CATTGTAC	XM_159825	Mus musculus LOC207745 (LOC207745), mR.			2.5	SM2058	a		5	1.522164293
mPool2_V2MM_12938	1.404025	3.004939	TRUE	HP_236321	TGCTGTTG	CAGAGATT	NM_018818	choroidermia	Chm		2.11	SM2389	a		2	1.522164293
092308m3_V2MM_44128	1.403683	62.64426	TRUE	HP_266694	TGCTGTTG	CTGTTTGT	NM_172588	RIKEN cD A130038L21 gene	A130038L21Rik		2.12	SM2411	h		3	0.203118686
092308m1_V2MM_61794	1.403167	3.004939	TRUE	HP_283917	TGCTGTTG	CAAGGTGA	NM_011084	phosphatidylinositol 3-kise, C2 domain containing, gamma po	Pik3c2g		2.4	SM2019	h		12	1.522164293
092308m1_V2MM_146706	1.402544	3.004939	TRUE	HP_367417	TGCTGTTG	CAATGGTG	XM_162225	Mus musculus LOC243248 (LOC243248), mR.			2.5	SM2056	c		11	1.522164293
mPool2_V2MM_157721	1.402144	3.004939	TRUE	HP_378335	TGCTGTTG	CCACTGTA	NM_024477	expressed sequence AI428795	AI428795		2.6	SM2132	e		1	1.522164293
mPool2_V2MM_985	1.402002	3.004939	TRUE	HP_224641	TGCTGTTG	CAGTAAAG	NM_012013	factor in the germline alpha	Figla		2.11	SM2386	c		12	1.522164293
092308m3_V2MM_203319	1.401706	75.83547	TRUE	HP_309838	TGCTGTTG	CTCTTCTCC	NM_175397	RIKEN cD 5830484A20 gene	5830484A20Rik		2.9	SM2269	b		7	0.120127608
092308m3_V2MM_203319	1.401706	75.83547	TRUE	HP_309838	TGCTGTTG	CTCTTCTCC	NM_175397	RIKEN cDNA 5830484A20 gene	5830484A20Rik		2.9	SM2269	b		7	0.120127608
092308m3_V2MM_25958	1.401588	75.83547	TRUE	HP_249014	TGCTGTTG	CCCATCGG	NM_030739	vomerosal 1 receptor, D4	V1rd4		2.12	SM2433	a		11	0.120127608
092308m1_V2MM_139518	1.401294	2.205692	TRUE	HP_360238	TGCTGTTG	CAGCATAT	XM_155135	Mus musculus LOC239258 (LOC239258), mR.			2.5	SM2052	b		10	1.656455229
mPool4_V2MM_50539	1.400632	75.83547	TRUE	HP_272955	TGCTGTTG	CAGTGCAA	AK029735	RIKEN cDNA 4930518F03 gene	4930518F03Rik	NA				NA		0.120127608
mPool2_V2MM_101669	1.399984	3.004939	TRUE	HP_13937	TGCTGTTG	CAGTAAAT	AK088299	regulator of chromosome condensation (RCC1) and BTB (POZ)	Rcbtb1		2.1	SM2318	f		10	1.522164293
092308m1_V2MM_173349	1.399787	2.205692	TRUE	HP_393835	TGCTGTTG	CACCCTTCT	XM_287009	Mus musculus hypothetical gene supported by AK036841 (LOC329196), mR.			2.5	SM2077	a		3	1.656455229
092308m3_V2MM_91083	1.399619	60.13437	TRUE	HP_312499	TGCTGTTG	CACTTAGC	AK034060	hypothetical protein 9330152L17			2.13	SM2463	g		2	0.220877236
mPool2_V2MM_188431	1.398882	3.004939	TRUE	HP_332062	TGCTGTTG	GAATTTAT	NM_009917	chemokine (C-C motif) receptor 5	Ccr5		2.7	SM2165	f		8	1.522164293
mPool2_V2MM_127737	1.396732	2.205692	TRUE	HP_348559	TGCTGTTG	GTGACTAA	XM_145359	Mus musculus similar to vomerosal 1 receptor, G1 [Mus musculus] (LOC232868), mR.			2.6	SM2114	c		4	1.656455229
mPool2_V2MM_127737	1.396732	2.205692	TRUE	HP_348559	TGCTGTTG	GTGACTAA	XM_145359	Mus musculus similar to vomeronasal 1 receptor, G1 [Mus musculus] (LOC232868), mRI			2.6	SM2114	c		4	1.656455229
092308m3_V2MM_37115	1.396609	62.64426	TRUE	HP_259883	TGCTGTTG	GACACAGG	NM_146252	TBC1 domain family, member 13	Tbc1d13		2.12	SM2431	g		6	0.203118686
mPool2_V2MM_213927	1.396378	2.205692	TRUE	HP_431544	TGCTGTTG	CGGCAACA	XM_140048	Mus musculus similar to erythrocyte protein band 4.1-like 4b; expressed in high-metast			2.11	SM2351	a		6	1.656455229
mPool2_V2MM_159763	1.395906	3.004939	TRUE	HP_380356	TGCTGTTG	CAGATAAG	XM_283239	Mus musculus hypothetical gene supported by AK048863 (LOC328455), mRNA.			2.6	SM2128	d		1	1.522164293
092308m3_V2MM_141606	1.395755	69.72441	TRUE	HP_362319	TGCTGTTG	CTGTTTCA	XM_156811	Mus musculus LOC211465 (LOC211465), mR.			2.14	SM2518	c		11	0.156615137
mPool2_V2MM_20622	1.395571	3.004939	TRUE	HP_243811	TGCTGTTG	CAAATTAG	NM_008707	N-myristoyltransferase 1	Nmt1		2.11	SM2392	c		10	1.522164293
mPool2_V2MM_213769	1.395301	2.205692	TRUE	HP_431390	TGCTGTTG	GTTGTTCCT	XM_143184	similar to ribosomal protein L21			2.11	SM2366	d		5	1.656455229
mPool2_V2MM_169067	1.395029	3.004939	TRUE	HP_389567	TGCTGTTG	GGTATAAA	XM_286130	Mus musculus similar to ribosomal protein S6 (LOC330873), mRNA.			2.6	SM2128	a		1	1.522164293
mPool4_V2MM_128857	1.394013	61.29828	TRUE	HP_349670	TGCTGTTG	GAGAAGAA	XM_145946	Mus musculus similar to REN [Mus musculus] (LOC244145), mRNA.			2.14	SM2531	e		5	0.212551731
mPool4_V2MM_34724	1.393375	75.83547	TRUE	HP_257533	TGCTGTTG	CCGATACG	NM_019763	Msx2 interacting nuclear target protein			2.15	SM2574	c		12	0.120127608
mPool7_V2MM_68362	1.39232	3.004939	TRUE	HP_290324	TGCTGTTG	CTTTGCAA	NM_176972	ubiquitin specific protease 37	Usp37		2.8	SM2245	e		6	1.522164293
092308m1_V2MM_156354	1.391838	2.205692	TRUE	HP_376990	TGCTGTTG	CATTTAAAT	NM_212484	RIKEN cD A230103N10 gene	A230103N10Rik		2.6	SM2122	d		7	1.656455229
092308m1_V2MM_182797	1.391389	3.004939	TRUE	HP_403271	TGCTGTTG	GAGAGTCC	XM_288908	Mus musculus LOC332375 (LOC332375), mR.			2.5	SM2095	h		7	1.522164293
mPool2_V2MM_117469	1.391119	3.004939	TRUE	HP_338413	TGCTGTTG	GGGATTCT	XM_139384	Mus musculus similar to DKFZP434I092 protein (LOC239370), mRNA.			2.1	SM2348	a		1	1.522164293
092308m1_V2MM_184558	1.390746	2.205692	TRUE	HP_405030	TGCTGTTG	CCAATTATA	XM_289218	Mus musculus LOC332821 (LOC332821), mR.			2.5	SM2081	f		12	1.656455229
mPool2_V2MM_13630	1.390481	2.205692	TRUE	HP_236995	TGCTGTTG	CCAATAAA	NM_026435	RIKEN cDNA 1810045K17 gene	1810045K17Rik		2.11	SM2379	f		4	1.656455229
092308m1_V2MM_86116	1.39026	3.004939	TRUE	HP_307593	TGCTGTTG	CAGTGCTT	NM_146622	olfactory receptor 360	Olfr360		2.6	SM2113	a		1	1.522164293
mPool2_V2MM_169930	1.389017	3.004939	TRUE	HP_390425	TGCTGTTG	CTGAATTTA	XM_488597	hypothetical protein A730013G04			2.6	SM2131	g		1	1.522164293

092308m1_V2MM_141250	1.388988	3.004939	TRUE	HP_361963	TGCTGTTG	CTATGGGA	XM_156547	Mus musculus LOC239907 (LOC239907), mR.		2.4	SM2035	e	4	1.522164293
092308m1_V2MM_170863	1.388959	3.004939	TRUE	HP_391353	TGCTGTTG	CCGTTGAA	AK078039	hypothetical protein 6330400D04		2.5	SM2092	a	11	1.522164293
mPool2_V2MM_103237	1.388613	3.004939	TRUE	HP_324379	TGCTGTTG	CTGAGAGA	XM_128893	Nedd4 family interacting protein 1	Ndfip1	2.6	SM2148	h	9	1.522164293
092308m1_V2MM_181803	1.388076	3.004939	TRUE	HP_402277	TGCTGTTG	GAGACGAA	XM_288737	Mus musculus LOC332180 (LOC332180), mRNA.		2.5	SM2065	h	3	1.522164293
092308m1_V2MM_181803	1.388076	3.004939	TRUE	HP_402277	TGCTGTTG	GAGACGAA	XM_288737	Mus musculus LOC332180 (LOC332180), mR.		2.5	SM2065	h	3	1.522164293
mPool2_V2MM_134829	1.387898	3.004939	TRUE	HP_355553	TGCTGTTG	GTCTGGAA	XM_489254	RIKEN cDNA 6030466F02 gene	6030466F02Rik	2.11	SM2371	f	5	1.522164293
092308m1_V2MM_66786	1.386769	3.004939	TRUE	HP_288794	TGCTGTTG	CCAAGGTA	NM_007465	baculoviral IAP repeat-containing 2	Birc2	2.3	SM2009	f	9	1.522164293
mPool6_V2MM_82018	1.386704	75.83547	TRUE	HP_303604	TGCTGTTG	GTGTTTATT	NM_173434	RIKEN cDNA 9930111J21 gene	9930111J21Rik	2.8	SM2222	d	11	0.120127608
mPool6_V2MM_75286	1.386459	60.13437	TRUE	HP_297068	TGCTGTTG	CCAGCATC	NM_026319	coiled-coil domain containing 2	Ccdc2	2.8	SM2220	f	2	0.220877236
092308m1_V2MM_175917	1.385919	2.205692	TRUE	HP_396399	TGCTGTTG	CCATTCAG	AK029922	RIKEN cDNA 4930528J11 gene	4930528J11Rik	2.5	SM2083	d	4	1.656455229
092308m1_V2MM_175917	1.385919	2.205692	TRUE	HP_396399	TGCTGTTG	CCATTCAG	AK029922	RIKEN cD 4930528J11 gene	4930528J11Rik	2.5	SM2083	d	4	1.656455229
mPool2_V2MM_107081	1.385118	3.004939	TRUE	HP_328172	TGCTGTTG	GAAGAGTA	NM_133788	isoprenylcysteine carboxyl methyltransferase	Icmt	2.1	SM2318	d	5	1.522164293
092308m3_V2MM_119927	1.38479	75.83547	TRUE	HP_277316	TGCTGTTG	CCCAGATG	NM_146696	olfactory receptor 1477	Olfr1477	2.12	SM2410	c	1	0.120127608
mPool7_V2MM_72120	1.384529	3.004939	TRUE	HP_293985	TGCTGTTG	CTCTTTAAC	NM_146786	olfactory receptor 914	Olfr914	2.8	SM2241	g	12	1.522164293
mPool7_V2MM_78317	1.383992	3.004939	TRUE	HP_300009	TGCTGTTG	GACCAGGA	NM_009945	cytochrome c oxidase, subunit VIIa 2	Cox7a2	2.8	SM2248	b	2	1.522164293
mPool2_V2MM_128145	1.383839	3.004939	TRUE	HP_348959	TGCTGTTG	CAGAAACT	XM_145551	Mus musculus LOC233181 (LOC233181), mRNA.		2.1	SM2350	a	5	1.522164293
mPool2_V2MM_148872	1.383768	3.004939	TRUE	HP_369583	TGCTGTTG	CATCAACC	XM_164276	Mus musculus LOC244567 (LOC244567), mR.		2.1	SM2333	b	6	1.522164293
mPool2_V2MM_148872	1.383768	3.004939	TRUE	HP_369583	TGCTGTTG	CATCAACC	XM_164276	Mus musculus LOC244567 (LOC244567), mRNA.		2.1	SM2333	b	6	1.522164293
092308m1_V2MM_160643	1.383334	3.004939	TRUE	HP_381222	TGCTGTTG	GGTATCAA	XM_283590	Mus musculus similar to hypothetical protein 7 - rat (LOC329168), mR.		2.6	SM2119	h	6	1.522164293
092308m3_V2MM_96506	1.383121	75.83547	TRUE	HP_317758	TGCTGTTG	CTGTGTTT	XM_110772	Mus musculus similar to proteasome (prosome, macropain) subunit, beta type, 7 [Rattu		2.13	SM2470	a	10	0.120127608
092308m1_V2MM_124311	1.383058	1.959154	TRUE	HP_345163	TGCTGTTG	CCTTTACCT	XM_143381	Mus musculus similar to 60S acidic ribosomal protein P1 (LOC229548), mRNA.		2.5	SM2097	h	1	1.707931489
092308m1_V2MM_124311	1.383058	1.959154	TRUE	HP_345163	TGCTGTTG	CCTTTACCT	XM_143381	Mus musculus similar to 60S acidic ribosomal protein P1 (LOC229548), mR.		2.5	SM2097	h	1	1.707931489
092308m1_V2MM_172357	1.383023	3.004939	TRUE	HP_392843	TGCTGTTG	CCATGGTG	AK051520	hypothetical protein D130054H01		2.5	SM2095	c	6	1.522164293
092308m1_V2MM_172357	1.383023	3.004939	TRUE	HP_392843	TGCTGTTG	CCATGGTG	AK051520	hypothetical protein D130054H01		2.5	SM2095	c	6	1.522164293
mPool6_V2MM_168245	1.382849	59.99801	TRUE	HP_388752	TGCTGTTG	GACTTAGT	XM_285938	Mus musculus similar to amiloride binding protein 1 precursor; Amiloride-binding prote		2.8	SM2205	f	2	0.221863149
092308m1_V2MM_128297	1.382158	3.004939	TRUE	HP_349111	TGCTGTTG	CTTAGAAT	XM_145626	Mus musculus similar to envelope protein [Ovis aries] (LOC233260), mR.		2.5	SM2097	h	9	1.522164293
mPool7_V2MM_63244	1.3817	3.004939	TRUE	HP_285330	TGCTGTTG	CGAGTTAC	AK080900	cholinergic receptor, nicotinic, alpha polypeptide 5	Chrna5	2.8	SM2235	c	4	1.522164293
mPool2_V2MM_15141	1.381519	2.205692	TRUE	HP_238471	TGCTGTTG	CTATTCAA	NM_018755	plasma glutamate carboxypeptidase		2.11	SM2377	c	12	1.656455229
092308m1_V2MM_92679	1.380676	1.959154	TRUE	HP_314023	TGCTGTTG	GACTTGAA	AK030182	RIKEN cD A430081C19 gene	A430081C19Rik	2.6	SM2110	c	7	1.707931489
mPool7_V2MM_135449	1.379237	3.004939	TRUE	HP_356171	TGCTGTTG	CTTGTCTA	XM_150641	Mus musculus LOC240827 (LOC240827), mR.		2.4	SM2035	a	11	1.522164293
mPool7_V2MM_135449	1.379237	3.004939	TRUE	HP_356171	TGCTGTTG	CTTGTCTA	XM_150641	Mus musculus LOC240827 (LOC240827), mRNA.		2.4	SM2035	a	11	1.522164293
092308m3_V2MM_29803	1.379024	61.29828	TRUE	HP_252756	TGCTGTTG	CAGAGTGA	NM_011923	angiopoietin-like 2	Angptl2	2.12	SM2426	a	5	0.212551731
mPool2_V2MM_114333	1.378143	2.205692	TRUE	HP_335318	TGCTGTTG	GCCCTTAC	XM_137447	Mus musculus similar to DNA-directed RNA polymerase beta subunit 2 [Leptocoryphium		2.7	SM2157	a	2	1.656455229
092308m1_V2MM_143911	1.37805	3.004939	TRUE	HP_364623	TGCTGTTG	CAGCTATA	XM_159415	Mus musculus LOC245652 (LOC245652), mR.		2.5	SM2054	f	5	1.522164293
092308m1_V2MM_143911	1.37805	3.004939	TRUE	HP_364623	TGCTGTTG	CAGCTATA	XM_159415	Mus musculus LOC245652 (LOC245652), mRNA.		2.5	SM2054	f	5	1.522164293
mPool7_V2MM_62878	1.377679	2.205692	TRUE	HP_284974	TGCTGTTG	CATTTATAC	NM_146885	olfactory receptor 1294	Olfr1294	2.8	SM2233	e	8	1.656455229
mPool2_V2MM_141179	1.377065	3.004939	TRUE	HP_361892	TGCTGTTG	GAAATTTA	XM_156499	Mus musculus LOC239882 (LOC239882), mRNA.		2.4	SM2036	d	2	1.522164293
mPool2_V2MM_141179	1.377065	3.004939	TRUE	HP_361892	TGCTGTTG	GAAATTTA	XM_156499	Mus musculus LOC239882 (LOC239882), mR.		2.4	SM2036	d	2	1.522164293
mPool7_V2MM_201386	1.377022	1.959154	TRUE	HP_419415	TGCTGTTG	CCTCCTCTT	XM_287151	Mus musculus hypothetical gene supported by AK039753 (LOC329508), mRNA.		2.9	SM2285	b	9	1.707931489
092308m1_V2MM_179499	1.376769	3.004939	TRUE	HP_399973	TGCTGTTG	CAGAGTTT	XM_288344	Mus musculus LOC327806 (LOC327806), mRNA.		2.5	SM2062	e	2	1.522164293
092308m1_V2MM_179499	1.376769	3.004939	TRUE	HP_399973	TGCTGTTG	CAGAGTTT	XM_288344	Mus musculus LOC327806 (LOC327806), mR.		2.5	SM2062	e	2	1.522164293
mPool4_V2MM_241261	1.37659	75.83547	TRUE	HP_493024	TGCTGTTG	GGCATTGA	XM_147063	gene model 518, (NCBI)	Gm518	2.14	SM2540	d	6	0.120127608
mPool2_V2MM_214087	1.376574	3.004939	TRUE	HP_431695	TGCTGTTG	CCATTGAA	XM_141846	Mus musculus similar to olfactory receptor MOR264-5 (LOC236866), mRNA.		2.11	SM2357	g	8	1.522164293
mPool2_V2MM_1363	1.375258	3.004939	TRUE	HP_225008	TGCTGTTG	CCAGGATT	NM_026793	myc target 1	Myct1	2.11	SM2386	h	8	1.522164293
mPool2_V2MM_10867	1.375201	3.004939	TRUE	HP_234289	TGCTGTTG	CTGGGATA	NM_010867	myomesin 1	Myom1	2.11	SM2377	f	6	1.522164293
092308m3_V2MM_143329	1.373046	75.83547	TRUE	HP_364041	TGCTGTTG	CAGAGCAT	XM_158642	Mus musculus LOC214960 (LOC214960), mR.		2.14	SM2520	f	4	0.120127608
092308m1_V2MM_145363	1.372971	0	TRUE	HP_366074	TGCTGTTG	GAGCTAGT	XM_487784	similar to hypothetical protein FLJ35802		2.4	SM2049	g	8	#NUM!
mPool2_V2MM_202732	1.372744	3.004939	TRUE	HP_420722	TGCTGTTG	CCATTATTC	XM_138204	Mus musculus similar to RIKEN cDNA 2810037C14 [Mus musculus] (LOC217805), mRNA		2.1	SM2319	b	7	1.522164293
mPool2_V2MM_202732	1.372744	3.004939	TRUE	HP_420722	TGCTGTTG	CCATTATTC	XM_138204	Mus musculus similar to RIKEN cD 2810037C14 [Mus musculus] (LOC217805), mR.		2.1	SM2319	b	7	1.522164293
092308m3_V2MM_20653	1.372689	69.72441	TRUE	HP_243841	TGCTGTTG	CAACGTGT	NM_030724	uridine monophosphate kise	Umpk	2.12	SM2422	d	9	0.156615137
mPool7_V2MM_180746	1.372306	3.004939	TRUE	HP_401220	TGCTGTTG	CCCATATTC	XM_288558	Mus musculus LOC331956 (LOC331956), mRNA.		2.5	SM2088	h	1	1.522164293
mPool7_V2MM_180746	1.372306	3.004939	TRUE	HP_401220	TGCTGTTG	CCCATATTC	XM_288558	Mus musculus LOC331956 (LOC331956), mR.		2.5	SM2088	h	1	1.522164293
092308m1_V2MM_181014	1.372215	3.004939	TRUE	HP_401488	TGCTGTTG	GAGATGTT	XM_288604	LOC433061		2.5	SM2081	f	5	1.522164293
092308m3_V2MM_87414	1.371194	75.83547	TRUE	HP_308870	TGCTGTTG	CTGTCGAT	NM_175161	RIKEN cD 4931422A03 gene	4931422A03Rik	2.13	SM2469	b	12	0.120127608
mPool2_V2MM_19053	1.371023	3.004939	TRUE	HP_242288	TGCTGTTG	GGAGTTAT	NM_007641	membrane-spanning 4-domains, subfamily A, member 1	Ms4a1	2.11	SM2381	b	11	1.522164293
mPool2_V2MM_34625	1.370983	3.004939	TRUE	HP_257439	TGCTGTTG	CCCTGGAG	NM_172310	RIKEN cDNA A530046H20 gene	A530046H20Rik	2.11	SM2391	h	11	1.522164293
mPool2_V2MM_129787	1.370312	2.205692	TRUE	HP_350588	TGCTGTTG	GAGGATAT	XM_146456	Mus musculus similar to cytochrome c (LOC234608), mRNA.		2.11	SM2357	h	8	1.656455229
mPool2_V2MM_116258	1.369758	3.004939	TRUE	HP_337213	TGCTGTTG	CTAAGTGT	XM_138836	Mus musculus similar to RIKEN cDNA 2610018L09; expressed sequence AV083614 [Mus		2.1	SM2312	f	7	1.522164293
mPool2_V2MM_4470	1.36973	3.004939	TRUE	HP_228044	TGCTGTTG	GTGTAACT	L42338	sodium channel, voltage-gated, type IX, alpha polypeptide	Scn9a	2.11	SM2384	a	1	1.522164293
092308m3_V2MM_203923	1.369369	75.83547	TRUE	HP_421847	TGCTGTTG	GGCAACGC	XM_137051	Mus musculus similar to Gag [Ovis aries] (LOC212923), mR.		2.13	SM2487	b	9	0.120127608
mPool2_V2MM_213066	1.369171	3.004939	TRUE	HP_377867	TGCTGTTG	CTATGTATT	XM_205092	Mus musculus similar to Zinc finger protein 180 (HHZ168) (LOC277541), mRNA.		2.11	SM2355	h	8	1.522164293
092308m1_V2MM_173142	1.368296	3.004939	TRUE	HP_393628	TGCTGTTG	GGAGTAAT	XM_286966	Mus musculus hypothetical gene supported by AK051123 (LOC329083), mRNA.		2.5	SM2089	f	4	1.522164293

092308m1_V2MM_173142	1.368296	3.004939	TRUE	HP_393628	TGCTGTTG/	GGAGTAAT	XM_286966	Mus musculus hypothetical gene supported by AK051123 (LOC329083), mR.			2.5	SM2089	f		4	1.522164293
mPool7_V2MM_90210	1.367455	3.004939	TRUE	HP_311633	TGCTGTTG/	CTCATATTC	NM_177756	glycosyltransferase 25 domain containing 2	Glt25d2		2.9	SM2255	f		1	1.522164293
mPool2_V2MM_7117	1.367284	2.205692	TRUE	HP_230633	TGCTGTTG/	GAGACATT(	NM_018827	cytokine receptor-like factor 1	Crlf1		2.11	SM2384	e		2	1.656455229
mPool4_V2MM_9537	1.367204	35.99881	TRUE	HP_232997	TGCTGTTG/	CAGGTTGC	NM_028034	RIKEN cDNA 2410004F06 gene	2410004F06Rik		2.16	SM2603	h		4	0.443711899
mPool2_V2MM_138233	1.36587	2.205692	TRUE	HP_358953	TGCTGTTG/	GTGTTAAG	XM_154067	Mus musculus LOC238611 (LOC238611), mRNA.			2.1	SM2343	d		3	1.656455229
mPool6_V2MM_156063	1.365658	75.83547	TRUE	HP_376702	TGCTGTTG/	CAAGTGTG.	XM_198081	Mus musculus LOC272439 (LOC272439), mRNA.			2.8	SM2207	b		6	0.120127608
092308m3_V2MM_44054	1.365549	75.83547	TRUE	HP_266620	TGCTGTTG/	CTGTGCGT(	NM_021421	D segment, Chr 1, ERATO Doi 396, expressed	D1Ertd396e		2.12	SM2418	c		4	0.120127608
mPool4_V2MM_154881	1.365505	75.83547	TRUE	HP_375522	TGCTGTTG/	GAAATCAC	XM_197072	Mus musculus LOC271143 (LOC271143), mRNA.		NA				NA		0.120127608
mPool7_V2MM_197384	1.36546	3.004939	TRUE	HP_415456	TGCTGTTG/	CTATTCTCC	NM_178921	expressed sequence AI987712	AI987712		2.9	SM2254	f		7	1.522164293
092308m1_V2MM_73666	1.365188	2.205692	TRUE	HP_295495	TGCTGTTG/	GGTCTTTG(	NM_029569	ankyrin repeat and SOCs box-containing protein 5	Asb5		2.3	SM2004	b		11	1.656455229
092308m3_V2MM_233179	1.365051	75.83547	TRUE	HP_400724	TGCTGTTG/	CAATATCA(	XM_288475	Mus musculus LOC331813 (LOC331813), mR.			2.13	SM2486	a		5	0.120127608
mPool2_V2MM_169309	1.364618	3.004939	TRUE	HP_389804	TGCTGTTG/	CTGCTTCTC	XM_286231	Mus musculus hypothetical gene supported by AK028286 (LOC327723), mRNA.			2.6	SM2128	h		7	1.522164293
mPool5_V2MM_377	1.364452	66.95036	TRUE	HP_224047	TGCTGTTG/	CACCCTAAT	NM_007752	ceruloplasmin	Cp	NA				NA		0.1742471
mPool2_V2MM_9971	1.364444	2.205692	TRUE	HP_233415	TGCTGTTG/	CGGAGAGA	NM_133208	zinc finger protein 287	Zfp287		2.11	SM2378	b		12	1.656455229
mPool4_V2MM_220522	1.362919	75.83547	TRUE	HP_453484	TGCTGTTG/	GGTATAGT(	NM_053096	camello-like 2	Cml2		2.15	SM2592	b		2	0.120127608
mPool2_V2MM_8819	1.362743	2.205692	TRUE	HP_232299	TGCTGTTG/	CAAATATC(	NM_177719	RIKEN cDNA 4932411A10 gene	4932411A10Rik		2.11	SM2378	d		7	1.656455229
mPool7_V2MM_205643	1.36191	3.004939	TRUE	HP_423498	TGCTGTTG/	CTTTGATG(	XM_127760	Mus musculus neurofilament, light polypeptide (Nefl), mRNA.			2.1	SM2317	g		11	1.522164293
092308m1_V2MM_140937	1.361806	3.004939	TRUE	HP_361650	TGCTGTTG/	GGTGCTAT(	XM_156372	similar to RIKEN cD 1810036I24			2.4	SM2042	a		2	1.522164293
092308m1_V2MM_117571	1.361793	2.205692	TRUE	HP_338512	TGCTGTTG/	CATAATCA/	XM_139413	Mus musculus similar to env protein [Sus scrofa] (LOC223510), mR.			2.5	SM2098	e		12	1.656455229
092308m1_V2MM_129870	1.361555	3.004939	TRUE	HP_350671	TGCTGTTG/	CTCAGGGA	XM_146492	RIKEN cDNA 1700031F13 gene	1700031F13Rik		2.6	SM2105	d		1	1.522164293
092308m1_V2MM_129870	1.361555	3.004939	TRUE	HP_350671	TGCTGTTG/	CTCAGGGA	XM_146492	RIKEN cD 1700031F13 gene	1700031F13Rik		2.6	SM2105	d		1	1.522164293
mPool2_V2MM_25870	1.361468	3.004939	TRUE	HP_248928	TGCTGTTG/	CCATTCAAA	NM_145542	S-adenosylhomocysteine hydrolase-like 1	Ahcyl1		2.11	SM2389	d		8	1.522164293
092308m1_V2MM_172020	1.360958	3.004939	TRUE	HP_392506	TGCTGTTG/	CAGTTTAA/	XM_286739	Mus musculus hypothetical gene supported by AK086345 (LOC328673), mRNA.			2.5	SM2071	f		10	1.522164293
092308m1_V2MM_172020	1.360958	3.004939	TRUE	HP_392506	TGCTGTTG/	CAGTTTAA/	XM_286739	Mus musculus hypothetical gene supported by AK086345 (LOC328673), mR.			2.5	SM2071	f		10	1.522164293
mPool2_V2MM_88194	1.360565	3.004939	TRUE	HP_309638	TGCTGTTG/	CATTCAGT/	NM_175340	NHL repeat containing 1	Nhlrc1		2.6	SM2144	h		6	1.522164293
mPool7_V2MM_84338	1.35974	3.004939	TRUE	HP_305851	TGCTGTTG/	CAAGGGAT	NM_027642	PHD finger protein 6	Phf6		2.9	SM2269	g		8	1.522164293
092308m3_V2MM_43881	1.359124	75.83547	TRUE	HP_266450	TGCTGTTG/	CTGCTTTCT	NM_177069	RIKEN cD E330009P21 gene	E330009P21Rik		2.12	SM2413	h		8	0.120127608
mPool7_V2MM_189131	1.358544	3.004939	TRUE	HP_90702	TGCTGTTG/	GAGATATA	NM_025513	exosome component 3	Exosc3		2.1	SM2302	h		7	1.522164293
mPool7_V2MM_189131	1.358544	3.004939	TRUE	HP_90702	TGCTGTTG/	GAGATATA	NM_025513	exosome component 3	Exosc3		2.1	SM2302	h		7	1.522164293
mPool7_V2MM_108516	1.358177	2.205692	TRUE	HP_329583	TGCTGTTG/	GATATTTG1	BC060132	RIKEN cDNA 2410127E18 gene	2410127E18Rik		2.1	SM2314	h		4	1.656455229
mPool6_V2MM_58613	1.357369	75.83547	TRUE	HP_280830	TGCTGTTG/	GAGTTCAT(	NM_010206	fibroblast growth factor receptor 1	Fgfr1		2.7	SM2185	c		2	0.120127608
092308m3_V2MM_210133	1.357159	75.83547	TRUE	HP_427893	TGCTGTTG/	GAGAACAA	XM_195264	similar to 60S ribosomal protein L23a			2.14	SM2523	f		10	0.120127608
092308m1_V2MM_164762	1.357066	3.004939	TRUE	HP_385302	TGCTGTTG/	GATCCCTTT	XM_285090	RIKEN cD 4930563I02 gene	4930563I02Rik		2.6	SM2119	f		12	1.522164293
mPool6_V2MM_190132	1.356935	75.83547	TRUE	HP_294121	TGCTGTTG/	CTGATTGT(	XM_487040	similar to proliferation associated nuclear element 1 isoform 1; proliferation associated			2.7	SM2174	h		9	0.120127608
mPool2_V2MM_214912	1.356424	3.004939	TRUE	HP_432483	TGCTGTTG/	CTATGATT/	XM_149213	RIKEN cDNA 2610318C08 gene	2610318C08Rik		2.11	SM2363	b		7	1.522164293
mPool7_V2MM_68410	1.356248	3.004939	TRUE	HP_290370	TGCTGTTG/	GAAATAAC	XM_484640	similar to Ab2-450			2.8	SM2245	h		3	1.522164293
mPool4_V2MM_36459	1.35572	61.29828	TRUE	HP_259239	TGCTGTTG/	CTGTAAGA.	NM_008618	malate dehydrogenase 1, NAD (soluble)	Mdh1		2.15	SM2575	e		8	0.212551731
mPool2_V2MM_131867	1.355553	3.004939	TRUE	HP_352627	TGCTGTTG/	CATAGTTT(	XM_147865	Mus musculus LOC219109 (LOC219109), mRNA.			2.11	SM2353	b		8	1.522164293
092308m1_V2MM_124793	1.35512	2.205692	TRUE	HP_345642	TGCTGTTG/	CTGACCTA(	XM_143634	Mus musculus similar to hypothetical protein [Dictyostelium discoideum] (LOC242308),			2.5	SM2097	b		2	1.656455229
mPool7_V2MM_105148	1.355073	3.004939	TRUE	HP_326272	TGCTGTTG/	GGCTTAGA	AK079931	peptidyl-prolyl isomerase G (cyclophilin G)	Ppig		2.9	SM2300	a		9	1.522164293
092308m1_V2MM_141179	1.35469	3.004939	TRUE	HP_361892	TGCTGTTG/	GAAATTTA(	XM_156499	Mus musculus LOC239882 (LOC239882), mRNA.			2.4	SM2036	d		2	1.522164293
092308m1_V2MM_141179	1.35469	3.004939	TRUE	HP_361892	TGCTGTTG/	GAAATTTA(	XM_156499	Mus musculus LOC239882 (LOC239882), mR.			2.4	SM2036	d		2	1.522164293
mPool7_V2MM_203507	1.354421	3.004939	TRUE	HP_421455	TGCTGTTG/	GTGCTCTA(	XM_128618	similar to zinc finger protein 97			2.9	SM2299	a		12	1.522164293
mPool6_V2MM_154578	1.354106	35.99881	TRUE	HP_375219	TGCTGTTG/	CATGGAAG	XM_196854	Mus musculus LOC270856 (LOC270856), mRNA.			2.7	SM2191	c		8	0.443711899
mPool2_V2MM_145920	1.353848	3.004939	TRUE	HP_366631	TGCTGTTG/	CTGATTCCT	XM_161614	Mus musculus LOC242812 (LOC242812), mRNA.			2.1	SM2337	b		7	1.522164293
092308m3_V2MM_94622	1.353759	61.29828	TRUE	HP_315909	TGCTGTTG/	GATTTAAT/	NM_183192	cD sequence BC027582	BC027582		2.13	SM2483	c		7	0.212551731
mPool4_V2MM_146407	1.353673	75.83547	TRUE	HP_367118	TGCTGTTG/	CTAAGTTC/	XM_161991	Mus musculus LOC243104 (LOC243104), mRNA.			2.16	SM2644	h		12	0.120127608
092308m1_V2MM_118369	1.353132	3.004939	TRUE	HP_339306	TGCTGTTG/	CTTACAAA1	XM_139818	Mus musculus similar to hypothetical protein FLJ32191 [Homo sapiens] (LOC224603), m			2.6	SM2101	a		6	1.522164293
092308m1_V2MM_118369	1.353132	3.004939	TRUE	HP_339306	TGCTGTTG/	CTTACAAA1	XM_139818	Mus musculus similar to hypothetical protein FLJ32191 [Homo sapiens] (LOC224603), m			2.6	SM2101	a		6	1.522164293
092308m3_V2MM_51219	1.352802	75.83547	TRUE	HP_273622	TGCTGTTG/	CTACTATGT	NM_010174	fatty acid binding protein 3, muscle and heart	Fabp3		2.12	SM2445	f		11	0.120127608
mPool2_V2MM_23246	1.352559	2.205692	TRUE	HP_246380	TGCTGTTG/	CTGCTGAA(	NM_009373	transglutaminase 2, C polypeptide	Tgm2		2.7	SM2166	b		9	1.656455229
092308m1_V2MM_179849	1.351574	3.004939	TRUE	HP_400323	TGCTGTTG/	CAGCAAAG	XM_288408	Mus musculus LOC331731 (LOC331731), mR.			2.5	SM2096	b		2	1.522164293
092308m3_V2MM_103016	1.351204	62.64426	TRUE	HP_324161	TGCTGTTG/	CTGGGTTA	XM_128755	Mus musculus spastic paraplegia 4 homolog (human) (Spg4), mR.			2.13	SM2494	g		10	0.203118686
mPool7_V2MM_203752	1.350019	3.004939	TRUE	HP_421684	TGCTGTTG/	GAAGCTAT	XM_285947	Mus musculus similar to ribosomal protein L7a; surfeit 3 [Mus musculus] (LOC333105), r			2.1	SM2322	e		1	1.522164293
mPool7_V2MM_196913	1.349989	3.004939	TRUE	HP_415002	TGCTGTTG/	CCAATACA1	NM_178735	RIKEN cDNA A730069N07 gene	A730069N07Rik		2.9	SM2259	c		12	1.522164293
mPool7_V2MM_67681	1.349842	2.205692	TRUE	HP_289662	TGCTGTTG/	CTAGTATT/	NM_172931	RIKEN cDNA B230358A15 gene	B230358A15Rik		2.8	SM2228	h		6	1.656455229
mPool4_V2MM_138982	1.349787	75.83547	TRUE	HP_359702	TGCTGTTG/	GTCACTGA	XM_154625	Mus musculus LOC218785 (LOC218785), mRNA.			2.16	SM2646	e		4	0.120127608
092308m1_V2MM_155277	1.34867	3.004939	TRUE	HP_375917	TGCTGTTG/	GCTATTGT(	XM_197334	Mus musculus LOC271472 (LOC271472), mR.			2.6	SM2122	a		11	1.522164293
092308m1_V2MM_159110	1.347827	3.004939	TRUE	HP_379711	TGCTGTTG/	CTATGGAA.	XM_282976	similar to hypothetical protein FLJ20345			2.6	SM2121	e		3	1.522164293
092308m3_V2MM_37804	1.347705	35.99881	TRUE	HP_260547	TGCTGTTG/	GAGCTGCA.	AK032985	RIKEN cD 2210018M11 gene	2210018M11Rik		2.12	SM2421	a		5	0.443711899
mPool7_V2MM_62510	1.347177	3.004939	TRUE	HP_284616	TGCTGTTG/	CAGGACTT(	NM_009897	creatine kinase, mitochondrial 1, ubiquitous	Ckmt1		2.8	SM2244	a		2	1.522164293
092308m1_V2MM_182121	1.345644	3.004939	TRUE	HP_402595	TGCTGTTG/	GTTAGAGC.	XM_288790	Mus musculus LOC332251 (LOC332251), mR.			2.5	SM2085	c		7	1.522164293

092308m3_V2MM_49039	1.344435	75.83547	TRUE	HP_271487	TGCTGTTG	GCTGCTTT	NM_177175	RIKEN cD A930001M12 gene	A930001M12Rik		2.12	SM2403	e		10	0.120127608
092308m3_V2MM_26629	1.34437	60.13437	TRUE	HP_249667	TGCTGTTG	CTCACATTA	NM_133865	D cross-link repair 1B, PSO2 homolog (S. cerevisiae)	Dclre1b		2.12	SM2429	e		4	0.220877236
mPool7_V2MM_187401	1.343034	3.004939	TRUE	HP_284980	TGCTGTTG	CATTTCATT	AK087793	importin 11	Ipo11		2.9	SM2287	f		8	1.522164293
mPool6_V2MM_4129	1.342903	61.29828	TRUE	HP_227706	TGCTGTTG	GGGACTGA	NM_008193	guanylate kinase 1	Guk1		2.7	SM2182	h		4	0.212551731
mPool7_V2MM_197099	1.342702	1.959154	TRUE	HP_415183	TGCTGTTG	CTATTTCTT	NM_173783	RIKEN cDNA B430216B18 gene	B430216B18Rik		2.9	SM2254	a		11	1.707931489
mPool7_V2MM_71118	1.342637	3.004939	TRUE	HP_293012	TGCTGTTG	CATAAGAG	XM_128178	splicing factor, arginine/serine-rich 2, interacting protein	Sfrs2ip		2.8	SM2228	d		6	1.522164293
mPool2_V2MM_131509	1.341959	3.004939	TRUE	HP_352272	TGCTGTTG	CACCCAAA	XM_147687	cDNA sequence BC042761	BC042761		2.7	SM2160	h		7	1.522164293
mPool4_V2MM_208232	1.341815	75.83547	TRUE	HP_426026	TGCTGTTG	CTGTGTTG	XM_162903	Mus musculus LOC208790 (LOC208790), mRNA.			2.16	SM2634	f		9	0.120127608
mPool4_V2MM_107294	1.340547	75.83547	TRUE	HP_328382	TGCTGTTG	CTGGGCAT	AK006670	RIKEN cDNA 1700041E20 gene	1700041E20Rik		2.16	SM2615	b		11	0.120127608
mPool7_V2MM_181741	1.340467	3.004939	TRUE	HP_402215	TGCTGTTG	CAAGTTAG	XM_288727	Mus musculus LOC332169 (LOC332169), mRNA.			2.5	SM2082	d		7	1.522164293
mPool7_V2MM_181741	1.340467	3.004939	TRUE	HP_402215	TGCTGTTG	CAAGTTAG	XM_288727	Mus musculus LOC332169 (LOC332169), mR.			2.5	SM2082	d		7	1.522164293
092308m3_V2MM_102585	1.340278	60.13437	TRUE	HP_323734	TGCTGTTG	CCAAGGAA	BC019219	RIKEN cD 2610036F08 gene	2610036F08Rik		2.13	SM2489	f		2	0.220877236
mPool7_V2MM_66554	1.340191	3.004939	TRUE	HP_288567	TGCTGTTG	CAGGGATA	NM_172914	hypothetical protein 4933409I22			2.8	SM2240	b		6	1.522164293
092308m3_V2MM_28083	1.339447	61.29828	TRUE	HP_251077	TGCTGTTG	GCACCAGG	M24780	phosphoglycerate kise 1, pseudogene 1	Pgk1-ps1		2.12	SM2428	c		2	0.212551731
092308m3_V2MM_235610	1.339331	75.83547	TRUE	HP_487475	TGCTGTTG	GGAACTTA	XM_157106	Mus musculus LOC211989 (LOC211989), mR.		NA				NA		0.120127608
092308m3_V2MM_86934	1.338899	75.83547	TRUE	HP_33284	TGCTGTTG	GGTGGACA	NM_174852	PHD finger protein 12	Phf12		2.13	SM2469	g		11	0.120127608
092308m1_V2MM_168117	1.33885	1.959154	TRUE	HP_388624	TGCTGTTG	GAAATTGT	XM_285912	Mus musculus similar to RE12057p (LOC330264), mR.			2.6	SM2117	f		8	1.707931489
mPool2_V2MM_162105	1.338726	3.004939	TRUE	HP_382666	TGCTGTTG	GAAAGTAT	XM_284203	Mus musculus hypothetical gene supported by AK030693 (LOC330316), mRNA.			2.6	SM2127	f		10	1.522164293
mPool6_V2MM_155837	1.338314	69.72441	TRUE	HP_376476	TGCTGTTG	GTTGCTCT	XM_197851	Mus musculus LOC269619 (LOC269619), mRNA.			2.8	SM2212	f		9	0.156615137
092308m3_V2MM_48464	1.338009	75.83547	TRUE	HP_270924	TGCTGTTG	GATTCAAC	AK044730	RIKEN cD A930037J23 gene	A930037J23Rik		2.12	SM2445	h		6	0.120127608
092308m1_V2MM_94159	1.337499	3.004939	TRUE	HP_315453	TGCTGTTG	CCTTAACT	AK013327	RIKEN cD 9130017K11 gene	9130017K11Rik		2.6	SM2108	e		10	1.522164293
092308m1_V2MM_123221	1.33728	3.004939	TRUE	HP_344077	TGCTGTTG	CATGTATG	XM_142785	RIKEN cD C030035D04 gene	C030035D04Rik		2.6	SM2103	d		7	1.522164293
092308m3_V2MM_93385	1.336599	75.83547	TRUE	HP_314702	TGCTGTTG	CACCCTAG	NM_181345	nucleophosmin/nucleoplasmin 2	Npm2		2.9	SM2265	e		1	0.120127608
092308m3_V2MM_93385	1.336599	75.83547	TRUE	HP_314702	TGCTGTTG	CACCCTAG	NM_181345	nucleophosmin/nucleoplasmin 2	Npm2		2.9	SM2265	e		1	0.120127608
092308m1_V2MM_181932	1.336473	3.004939	TRUE	HP_402406	TGCTGTTG	CACCAAGC	XM_288759	Mus musculus LOC332197 (LOC332197), mR.			2.5	SM2094	d		3	1.522164293
mPool2_V2MM_100868	1.336464	3.004939	TRUE	HP_322044	TGCTGTTG	GACCATAT	XM_127169	Mus musculus similar to periaxin (LOC217886), mRNA.			2.1	SM2312	c		3	1.522164293
mPool2_V2MM_114036	1.336367	2.205692	TRUE	HP_335023	TGCTGTTG	GTGAGACT	NM_001003717	oxysterol binding protein-like 8	Osbpl8		2.7	SM2157	d		3	1.656455229
092308m1_V2MM_152690	1.335699	3.004939	TRUE	HP_373352	TGCTGTTG	CTGAATTT	XM_195505	Mus musculus similar to hypothetical protein MGC34837 [Homo sapiens] (LOC269555),			2.6	SM2114	c		10	1.522164293
mPool2_V2MM_210411	1.335659	2.205692	TRUE	HP_428164	TGCTGTTG	CAACATAA	XM_145002	Mus musculus similar to hypothetical protein MGC34648 [Homo sapiens] (LOC214520),			2.11	SM2354	b		10	1.656455229
mPool7_V2MM_90177	1.335654	3.004939	TRUE	HP_311601	TGCTGTTG	GTTATCCTT	NM_177748	killer immunoglobulin-like receptor-like 2			2.9	SM2259	d		7	1.522164293
mPool7_V2MM_90177	1.335654	3.004939	TRUE	HP_311601	TGCTGTTG	GTTATCCTT	NM_177748	killer immunoglobulin-like receptor-like 2			2.9	SM2259	d		7	1.522164293
092308m3_V2MM_85206	1.335163	61.29828	TRUE	HP_306691	TGCTGTTG	CAGTATAT	NM_133225	acyl-Coenzyme A binding domain containing 3	Acbd3		2.8	SM2250	c		11	0.212551731
092308m3_V2MM_85206	1.335163	61.29828	TRUE	HP_306691	TGCTGTTG	CAGTATAT	NM_133225	acyl-Coenzyme A binding domain containing 3	Acbd3		2.8	SM2250	c		11	0.212551731
mPool2_V2MM_16553	1.334731	2.205692	TRUE	HP_239849	TGCTGTTG	GACAAATC	NM_134199	vomeronasal 1 receptor, F2	V1rf2		2.11	SM2381	f		10	1.656455229
mPool4_V2MM_262223	1.334692	75.83547	TRUE	HP_522972	TGCTGTTG	CTATTCCTG	XM_195633	Mus musculus similar to vomeronasal receptor V1RD12 [Mus musculus] (LOC272357), n			2.16	SM2622	a		8	0.120127608
092308m1_V2MM_147832	1.334451	3.004939	TRUE	HP_368543	TGCTGTTG	GAAATCTA	XM_163306	Mus musculus LOC215016 (LOC215016), mR.			2.4	SM2049	a		9	1.522164293
092308m3_V2MM_45464	1.334246	66.95036	TRUE	HP_267997	TGCTGTTG	GTGCATAT	AK038854	neurexin I	Nrxn1		2.12	SM2407	c		4	0.1742471
mPool6_V2MM_74693	1.334019	75.83547	TRUE	HP_296494	TGCTGTTG	CAGCTAAT	NM_007381	acetyl-Coenzyme A dehydrogenase, long-chain	Acadl		2.8	SM2218	c		11	0.120127608
092308m1_V2MM_178348	1.3338	3.004939	TRUE	HP_398822	TGCTGTTG	GTCCTAAG	AK081752	hypothetical protein C130073P14			2.5	SM2092	a		3	1.522164293
mPool4_V2MM_210787	1.333753	66.95036	TRUE	HP_428523	TGCTGTTG	GTGAGCAA	XM_142291	Mus musculus similar to differentiation inhibitor protein Id2A - human (LOC237200), mF			2.14	SM2541	f		1	0.1742471
092308m3_V2MM_142011	1.332867	60.13437	TRUE	HP_362724	TGCTGTTG	CACATCAT	XM_157271	Mus musculus LOC240249 (LOC240249), mR.			2.14	SM2512	g		6	0.220877236
mPool7_V2MM_185131	1.332667	3.004939	TRUE	HP_405603	TGCTGTTG	CTACTTGA	XM_289316	Mus musculus LOC332973 (LOC332973), mR.			2.5	SM2066	f		10	1.522164293
mPool7_V2MM_185131	1.332667	3.004939	TRUE	HP_405603	TGCTGTTG	CTACTTGA	XM_289316	Mus musculus LOC332973 (LOC332973), mRNA.			2.5	SM2066	f		10	1.522164293
092308m1_V2MM_141085	1.331848	2.205692	TRUE	HP_361798	TGCTGTTG	CTATTGAG	XM_156459	Mus musculus LOC239959 (LOC239959), mR.			2.4	SM2036	g		12	1.656455229
092308m1_V2MM_141085	1.331848	2.205692	TRUE	HP_361798	TGCTGTTG	CTATTGAG	XM_156459	Mus musculus LOC239959 (LOC239959), mRNA.			2.4	SM2036	g		12	1.656455229
092308m3_V2MM_234348	1.331842	75.83547	TRUE	HP_486227	TGCTGTTG	GTTATCCT	XM_137038	Mus musculus similar to protease [Mus musculus] (LOC215893), mR.			2.13	SM2491	a		3	0.120127608
092308m3_V2MM_231569	1.331187	61.29828	TRUE	HP_483532	TGCTGTTG	CACATGGA	XM_131217	cD sequence BC007180	BC007180		2.13	SM2495	c		9	0.212551731
mPool6_V2MM_166047	1.331078	75.83547	TRUE	HP_386578	TGCTGTTG	GTCACGGA	XM_285398	similar to High mobility group protein 1-like 10 (HMG-1L10)			2.8	SM2206	h		4	0.120127608
mPool2_V2MM_1144	1.33081	3.004939	TRUE	HP_224794	TGCTGTTG	CATATTCA	XM_136490	Mus musculus similar to degenerative spermatocyte-like protein RDES [Rattus norvegicu			2.11	SM2377	b		8	1.522164293
092308m3_V2MM_189641	1.329225	69.72441	TRUE	HP_327131	TGCTGTTG	GCTTATGTT	XM_355444	RIKEN cD 1700021C14 gene	1700021C14Rik		2.14	SM2507	h		10	0.156615137
mPool7_V2MM_209654	1.328044	3.004939	TRUE	HP_427421	TGCTGTTG	CCCAAGGA	XM_151310	Mus musculus LOC211412 (LOC211412), mRNA.			2.1	SM2329	h		11	1.522164293
mPool7_V2MM_209654	1.328044	3.004939	TRUE	HP_427421	TGCTGTTG	CCCAAGGA	XM_151310	Mus musculus LOC211412 (LOC211412), mR.			2.1	SM2329	h		11	1.522164293
092308m1_V2MM_64478	1.32689	3.004939	TRUE	HP_286531	TGCTGTTG	CTGGTGAT	NM_146836	olfactory receptor 1132	Olfr1132		2.3	SM2010	e		3	1.522164293
mPool2_V2MM_123173	1.326661	2.205692	TRUE	HP_344029	TGCTGTTG	CAATTTGA	XM_142727	nuclear antigen Sp100	Sp100		2.11	SM2369	d		9	1.656455229
mPool4_V2MM_140191	1.326409	70.87591	TRUE	HP_360911	TGCTGTTG	GTGATCAC	XM_155791	Mus musculus LOC239475 (LOC239475), mRNA.			2.16	SM2637	g		5	0.149501346
mPool4_V2MM_110591	1.325895	75.83547	TRUE	HP_331625	TGCTGTTG	CAAGATTG	XM_489276	RIKEN cDNA 1700036A12 gene	1700036A12Rik		2.16	SM2608	c		8	0.120127608
092308m1_V2MM_158721	1.325738	3.004939	TRUE	HP_379331	TGCTGTTG	GACCTTTA	XM_207141	similar to SmcY protein (Histocompatibility Y antigen) (H-Y)			2.6	SM2121	d		6	1.522164293
mPool7_V2MM_80618	1.324783	3.004939	TRUE	HP_302245	TGCTGTTG	GGAATTAG	NM_025702	RIKEN cDNA 3526402H21 gene	3526402H21Rik		2.8	SM2243	c		11	1.522164293
092308m1_V2MM_173571	1.324646	3.004939	TRUE	HP_394057	TGCTGTTG	CAGACATG	XM_287051	Mus musculus hypothetical gene supported by AK042187 (LOC329287), mR.			2.5	SM2072	e		2	1.522164293
092308m3_V2MM_88748	1.324347	62.64426	TRUE	HP_310184	TGCTGTTG	GTTCTTATT	NM_175481	glutamate receptor, ionotropic, kaite 4	Grik4		2.13	SM2480	h		12	0.203118686
mPool7_V2MM_92571	1.323838	2.205692	TRUE	HP_313918	TGCTGTTG	GACCTTAA	NM_178735	RIKEN cDNA A730069N07 gene	A730069N07Rik		2.9	SM2253	d		7	1.656455229
mPool2_V2MM_143843	1.323587	3.004939	TRUE	HP_364555	TGCTGTTG	GCTCACAG	XM_159211	Mus musculus LOC245417 (LOC245417), mRNA.			2.1	SM2324	g		11	1.522164293

mPool7_V2MM_200046	1.323157	2.205692	TRUE	HP_418083	TGCTGTTG/	CATCTACTT	AK080130	hypothetical protein A530065N20		2.9	SM2284	c	3	1.656455229
mPool7_V2MM_195927	1.322947	3.004939	TRUE	HP_414045	TGCTGTTG/	CAATGTTC/	NM_176837	Rho GTPase activating protein 18	Arhgap18	2.9	SM2264	h	1	1.522164293
mPool2_V2MM_2099	1.322328	0	TRUE	HP_225727	TGCTGTTG/	CTATACCTC	NM_011406	solute carrier family 8 (sodium/calcium exchanger), member	Slc8a1	2.11	SM2387	d	4	#NUM!
092308m3_V2MM_51919	1.322024	75.83547	TRUE	HP_274306	TGCTGTTG/	CTGGAATA	NM_028534	stromal membrane-associated protein 1	Smap1	2.12	SM2405	a	3	0.120127608
mPool7_V2MM_195087	1.321884	3.004939	TRUE	HP_413236	TGCTGTTG/	GGCTCAGT	XM_110936	similar to peptidyl-dipeptidase A (EC 3.4.15.1), testis - mouse		2.9	SM2253	a	3	1.522164293
mPool2_V2MM_108097	1.32131	3.004939	TRUE	HP_329171	TGCTGTTG/	GTCACTTC/	AK002701	RIKEN cDNA 0610030E20 gene	0610030E20Rik	2.7	SM2151	g	12	1.522164293
092308m1_V2MM_165846	1.321286	3.004939	TRUE	HP_386380	TGCTGTTG/	CCCGAGAA	NM_026361	plakophilin 4	Pkp4	2.6	SM2118	a	10	1.522164293
mPool2_V2MM_24795	1.320845	2.205692	TRUE	HP_247883	TGCTGTTG/	GGTGTTGT	NM_019702	Hbs1-like (S. cerevisiae)	Hbs1l	2.11	SM2390	a	3	1.656455229
092308m1_V2MM_181407	1.319822	3.004939	TRUE	HP_401881	TGCTGTTG/	CAGACCAA	XM_288670	Mus musculus LOC332096 (LOC332096), mR.		2.5	SM2060	e	4	1.522164293
092308m1_V2MM_177974	1.31922	2.205692	TRUE	HP_398451	TGCTGTTG/	GACCTGAT	XM_287992	Mus musculus hypothetical gene supported by AK078598 (LOC331116), mR.		2.5	SM2091	h	9	1.656455229
092308m3_V2MM_197217	1.318688	75.83547	TRUE	HP_415294	TGCTGTTG/	CAGCTCAC	XM_487805	similar to limitin		2.9	SM2255	c	7	0.120127608
092308m3_V2MM_197217	1.318688	75.83547	TRUE	HP_415294	TGCTGTTG/	CAGCTCAC	XM_487805	similar to limitin		2.9	SM2255	c	7	0.120127608
mPool2_V2MM_86235	1.318485	3.004939	TRUE	HP_307711	TGCTGTTG/	CTGGCAC/	NM_146658	olfactory receptor 1131	Olfr1131	2.6	SM2141	d	8	1.522164293
092308m3_V2MM_43419	1.3182	75.83547	TRUE	HP_266002	TGCTGTTG/	CTCCTCTAT	NM_016667	syntrophin, basic 1	Sntb1	2.12	SM2403	b	8	0.120127608
mPool2_V2MM_115572	1.317861	3.004939	TRUE	HP_336533	TGCTGTTG/	CCTGTTGC	XM_138503	Mus musculus similar to spermine synthase; spermidine aminopropyltransferase [Homc		2.7	SM2157	h	1	1.522164293
092308m1_V2MM_182565	1.317846	3.004939	TRUE	HP_403039	TGCTGTTG/	GACTAAAT	XM_288868	RIKEN cDNA 4930546C10 gene	4930546C10Rik	2.5	SM2071	d	10	1.522164293
092308m1_V2MM_182565	1.317846	3.004939	TRUE	HP_403039	TGCTGTTG/	GACTAAAT	XM_288868	RIKEN cD 4930546C10 gene	4930546C10Rik	2.5	SM2071	d	10	1.522164293
mPool7_V2MM_85297	1.317825	3.004939	TRUE	HP_306781	TGCTGTTG/	GCTTTATC/	NM_133907	ubiquitin protein ligase E3C	Ube3c	2.9	SM2267	e	12	1.522164293
mPool7_V2MM_198272	1.317695	3.004939	TRUE	HP_416329	TGCTGTTG/	CAGGGATT	XM_287379	Mus musculus hypothetical gene supported by AK039622 (LOC329946), mRNA.		2.9	SM2276	h	1	1.522164293
mPool7_V2MM_65152	1.317428	3.004939	TRUE	HP_287196	TGCTGTTG/	GATATGAG	NM_025687	testis expressed gene 12	Tex12	2.8	SM2231	f	2	1.522164293
092308m1_V2MM_84959	1.317312	2.205692	TRUE	HP_306458	TGCTGTTG/	CATAATTA/	NM_029947	PR domain containing 8	Prdm8	2.6	SM2110	g	2	1.656455229
mPool2_V2MM_101887	1.31709	3.004939	TRUE	HP_323050	TGCTGTTG/	GGCTAAAT	NM_027707	RIKEN cDNA 4933421G18 gene	4933421G18Rik	2.7	SM2157	c	8	1.522164293
mPool2_V2MM_210897	1.316985	3.004939	TRUE	HP_428632	TGCTGTTG/	GAGATTAG	AK021091	tryptophan rich basic protein	Wrb	2.11	SM2353	e	5	1.522164293
mPool4_V2MM_197645	1.316257	75.83547	TRUE	HP_415711	TGCTGTTG/	CGTGATGC	XM_125547	solute carrier family 22 (organic cation transporter), member	Slc22a16	2.16	SM2624	g	4	0.120127608
mPool2_V2MM_147313	1.31617	2.205692	TRUE	HP_368024	TGCTGTTG/	GGAAACAA	XM_162860	Mus musculus LOC232484 (LOC232484), mR.		2.5	SM2052	f	9	1.656455229
mPool2_V2MM_147313	1.31617	2.205692	TRUE	HP_368024	TGCTGTTG/	GGAAACAA	XM_162860	Mus musculus LOC232484 (LOC232484), mRNA.		2.5	SM2052	f	9	1.656455229
092308m1_V2MM_125029	1.31501	3.004939	TRUE	HP_345874	TGCTGTTG/	CATCCTGG	XM_143748	forkhead box E1 (thyroid transcription factor 2)	Foxe1	2.6	SM2111	e	8	1.522164293
mPool2_V2MM_3511	1.314849	2.205692	TRUE	HP_227105	TGCTGTTG/	GAGTTACA	NM_011562	teratocarcinoma-derived growth factor	Tdgf1	2.11	SM2380	e	10	1.656455229
mPool6_V2MM_160019	1.314336	75.83547	TRUE	HP_380612	TGCTGTTG/	CCAACTGT/	AK084162	Casitas B-lineage lymphoma b	Cblb	2.7	SM2196	d	11	0.120127608
mPool2_V2MM_153912	1.314013	3.004939	TRUE	HP_374555	TGCTGTTG/	CAGAGATT	BC068148	RIKEN cDNA 4732460K03 gene	4732460K03Rik	2.6	SM2128	b	4	1.522164293
mPool4_V2MM_212376	1.31331	61.29828	TRUE	HP_430042	TGCTGTTG/	GCACATGA	XM_358748	zinc finger protein 597	Zfp597	2.14	SM2535	a	6	0.212551731
mPool6_V2MM_223790	1.313082	61.29828	TRUE	HP_475915	TGCTGTTG/	CCTAGGAA	XM_284817	similar to bM64F17.3 (putative novel protein)		2.7	SM2197	h	4	0.212551731
092308m1_V2MM_181239	1.313008	3.004939	TRUE	HP_401713	TGCTGTTG/	CGGGTAGA	XM_288642	Mus musculus LOC332046 (LOC332046), mR.		2.5	SM2070	d	11	1.522164293
mPool6_V2MM_159820	1.312976	66.95036	TRUE	HP_380413	TGCTGTTG/	GCATTCGT	AK048688	F-box and leucine-rich repeat protein 7	Fbxl7	2.8	SM2206	d	1	0.1742471
mPool7_V2MM_113276	1.312816	3.004939	TRUE	HP_334263	TGCTGTTG/	CACAGTAT	XM_136902	Mus musculus similar to hypothetical protein A830058L05 [Mus musculus] (LOC241217		2.7	SM2152	f	4	1.522164293
mPool4_V2MM_130642	1.3127	75.83547	TRUE	HP_351421	TGCTGTTG/	CAGCCCAA	XM_146963	RIKEN cDNA C530008M07 gene	C530008M07Rik	2.11	SM2372	c	6	0.120127608
mPool2_V2MM_6731	1.311953	3.004939	TRUE	HP_230258	TGCTGTTG/	CTTTGATTC	NM_144500	oxysterol binding protein-like 2	Osbpl2	2.11	SM2387	h	11	1.522164293
092308m1_V2MM_82112	1.310989	2.205692	TRUE	HP_303695	TGCTGTTG/	GTTGCTTT/	NM_176998	RIKEN cD A830027B17 gene	A830027B17Rik	2.3	SM2016	b	9	1.656455229
092308m3_V2MM_58280	1.310958	75.83547	TRUE	HP_280510	TGCTGTTG/	GAGATTAT	AK083747	RIKEN cD D030074E01 gene	D030074E01Rik	2.12	SM2411	d	6	0.120127608
092308m3_V2MM_147598	1.310865	59.99801	TRUE	HP_368309	TGCTGTTG/	GCAACGCA	XM_163110	Mus musculus LOC243757 (LOC243757), mR.		2.4	SM2044	e	9	0.221863149
092308m3_V2MM_36096	1.310844	75.83547	TRUE	HP_258881	TGCTGTTG/	CTGAGAAA	NM_023595	deoxyuridine triphosphatase	Dutp	2.11	SM2396	c	12	0.120127608
mPool6_V2MM_162517	1.310776	75.83547	TRUE	HP_383076	TGCTGTTG/	CACCCTATT	XM_284382	Mus musculus hypothetical gene supported by AK032428 (LOC330700), mRNA.		2.8	SM2208	g	10	0.120127608
mPool4_V2MM_46831	1.310754	75.83547	TRUE	HP_269323	TGCTGTTG/	CGACGTTG	NM_139228	rhomboid, veinlet-like 4 (Drosophila)	Rhbdl4	2.15	SM2600	c	1	0.120127608
mPool2_V2MM_214537	1.310336	2.205692	TRUE	HP_432122	TGCTGTTG/	GAAGTTAT/	XM_141933	similar to Smage-1 protein		2.11	SM2368	d	1	1.656455229
mPool6_V2MM_37864	1.30933	75.83547	TRUE	HP_260606	TGCTGTTG/	GAGGCCAA	NM_009247	serine (or cysteine) proteinase inhibitor, clade A, member 1e	Serpina1e	2.7	SM2170	g	6	0.120127608
092308m1_V2MM_119905	1.308989	3.004939	TRUE	HP_340819	TGCTGTTG/	CAACTAGT	XM_488868	hypothetical gene supported by AK041445		2.5	SM2099	b	3	1.522164293
092308m1_V2MM_157072	1.308938	2.205692	TRUE	HP_377693	TGCTGTTG/	GCTCCTGT	XM_204841	gene model 703, (NCBI)	Gm703	2.6	SM2116	g	7	1.656455229
092308m1_V2MM_131242	1.308465	3.004939	TRUE	HP_352007	TGCTGTTG/	CTCCCAAG/	AB093288	cytoplasmic FMR1 interacting protein 2	Cyfip2	2.5	SM2100	d	9	1.522164293
mPool2_V2MM_137283	1.308407	2.205692	TRUE	HP_358003	TGCTGTTG/	CAACCAAC/	XM_152972	Mus musculus LOC237973 (LOC237973), mRNA.		2.5	SM2053	f	4	1.656455229
mPool2_V2MM_137283	1.308407	2.205692	TRUE	HP_358003	TGCTGTTG/	CAACCAAC/	XM_152972	Mus musculus LOC237973 (LOC237973), mR.		2.5	SM2053	f	4	1.656455229
092308m3_V2MM_23028	1.307768	61.29828	TRUE	HP_246167	TGCTGTTG/	CTGAAATA	NM_145578	ubiquitin-conjugating enzyme E2M (UBC12 homolog, yeast)	Ube2m	2.12	SM2430	c	7	0.212551731
mPool6_V2MM_79122	1.307434	75.83547	TRUE	HP_300792	TGCTGTTG/	GATACCTTC	NM_010695	lipocalin 4	Lcn4	2.8	SM2223	g	4	0.120127608
mPool2_V2MM_125948	1.305386	3.004939	TRUE	HP_346791	TGCTGTTG/	CCCAAGAT	XM_144299	similar to CG31559-PA		2.11	SM2362	f	2	1.522164293
mPool7_V2MM_95102	1.304662	3.004939	TRUE	HP_316384	TGCTGTTG/	GATTCAGA	NM_183390	kelch-like 6 (Drosophila)	Klhl6	2.9	SM2253	e	12	1.522164293
mPool7_V2MM_95102	1.304662	3.004939	TRUE	HP_316384	TGCTGTTG/	GATTCAGA	NM_183390	kelch-like 6 (Drosophila)	Klhl6	2.9	SM2253	e	12	1.522164293
mPool4_V2MM_157080	1.30386	60.13437	TRUE	HP_377701	TGCTGTTG/	GAATGGAC	XM_204847	Mus musculus similar to protease [Trichosurus vulpecula] (LOC277161), mRNA.		NA			NA	0.220877236
mPool7_V2MM_186224	1.303803	3.004939	TRUE	HP_406696	TGCTGTTG/	CAAGTAAA	XM_289503	Mus musculus LOC333220 (LOC333220), mRNA.		2.5	SM2060	c	4	1.522164293
mPool7_V2MM_186224	1.303803	3.004939	TRUE	HP_406696	TGCTGTTG/	CAAGTAAA	XM_289503	Mus musculus LOC333220 (LOC333220), mR.		2.5	SM2060	c	4	1.522164293
092308m3_V2MM_92129	1.302436	59.99801	TRUE	HP_313492	TGCTGTTG/	GCAAGACC	NM_178655	ankyrin 2, brain	Ank2	2.13	SM2464	g	11	0.221863149
092308m1_V2MM_176254	1.301912	2.205692	TRUE	HP_396736	TGCTGTTG/	CCAAATAT	XM_287631	Mus musculus RIKEN cD 3110052D19 gene (3110052D19Rik), mR.		2.5	SM2085	f	3	1.656455229
092308m1_V2MM_63085	1.301278	3.004939	TRUE	HP_285175	TGCTGTTG/	CCTCAGTTC	U47328	histocompatibility 2, K1, K region	H2-K1	2.4	SM2019	d	8	1.522164293
092308m3_V2MM_93253	1.30127	75.83547	TRUE	HP_314580	TGCTGTTG/	GATGGAAA	NM_178936	RIKEN cD 4930577M16 gene	4930577M16Rik	2.9	SM2265	h	5	0.120127608

092308m3_V2MM_93253	1.30127	75.83547	TRUE	HP_314580	TGCTGTTG	GATGGAAA	NM_178936	RIKEN cDNA 4930577M16 gene	4930577M16Rik		2.9	SM2265	h		5	0.120127608
mPool7_V2MM_171499	1.301051	3.004939	TRUE	HP_391988	TGCTGTTG	CTTGGATA	AK043702	RIKEN cDNA A830021K08 gene	A830021K08Rik		2.9	SM2281	b		9	1.522164293
mPool2_V2MM_104605	1.301025	3.004939	TRUE	HP_325737	TGCTGTTG	CACTGAGG	XM_129858	similar to General transcription factor 3C polypeptide 3 (Transcription factor IIIC-gamma			2.7	SM2162	d		7	1.522164293
mPool4_V2MM_92064	1.300716	75.83547	TRUE	HP_313427	TGCTGTTG	CCAAGGGA	NM_178642	expressed sequence AU040576	AU040576		2.16	SM2608	f		5	0.120127608
mPool7_V2MM_170548	1.300373	3.004939	TRUE	HP_391041	TGCTGTTG	CTTGGTTA	XM_286438	Mus musculus hypothetical gene supported by AK090201 (LOC328098), mRNA.			2.9	SM2291	f		11	1.522164293
092308m1_V2MM_166416	1.299703	2.205692	TRUE	HP_386944	TGCTGTTG	CTGCAAAT	XM_285495	Mus musculus similar to TRAF6-inhibitory zinc finger protein; TRAF6-binding zinc finger			2.6	SM2122	f		1	1.656455229
mPool4_V2MM_217595	1.299404	69.72441	TRUE	HP_450667	TGCTGTTG	CTGGTGGT	NM_011227	RAB20, member RAS oncogene family	Rab20		2.15	SM2570	h		4	0.156615137
mPool4_V2MM_195467	1.299216	75.83547	TRUE	HP_413607	TGCTGTTG	CACGGAAT	NM_146994	olfactory receptor 201	Olfr201		2.15	SM2593	c		3	0.120127608
mPool7_V2MM_171062	1.296879	3.004939	TRUE	HP_391551	TGCTGTTG	GGTTTACTT	AK052551	hypothetical protein D430050G20			2.5	SM2075	d		1	1.522164293
mPool4_V2MM_205752	1.296597	75.83547	TRUE	HP_423603	TGCTGTTG	CCAAGCAG	AK081462	neuropilin (NRP) and tolloid (TLL)-like 2	Neto2		2.16	SM2618	h		2	0.120127608
mPool2_V2MM_165073	1.296259	3.004939	TRUE	HP_385611	TGCTGTTG	CTTGACAA	XM_285162	Mus musculus similar to Gag [Ovis aries] (LOC332147), mRNA.			2.6	SM2128	e		6	1.522164293
mPool2_V2MM_108926	1.296093	2.205692	TRUE	HP_329992	TGCTGTTG	CAAGCATT	XM_133390	similar to low density lipoprotein receptor-related protein 3			2.7	SM2158	d		4	1.656455229
mPool2_V2MM_95105	1.29546	2.205692	TRUE	HP_316387	TGCTGTTG	CTAAGCTA	NM_183391	tumor necrosis factor (ligand) superfamily, member 18	Tnfsf18		2.6	SM2106	f		11	1.656455229
mPool2_V2MM_95105	1.29546	2.205692	TRUE	HP_316387	TGCTGTTG	CTAAGCTA	NM_183391	tumor necrosis factor (ligand) superfamily, member 18	Tnfsf18		2.6	SM2106	f		11	1.656455229
mPool2_V2MM_132410	1.295242	2.205692	TRUE	HP_353161	TGCTGTTG	GAGTTTAT	XM_358797	similar to Stefin homolog			2.11	SM2362	e		10	1.656455229
mPool6_V2MM_160009	1.295129	75.83547	TRUE	HP_380602	TGCTGTTG	CGCTTGCT	XM_489526	hypothetical gene supported by AK080987			2.8	SM2202	e		11	0.120127608
mPool7_V2MM_172897	1.294525	3.004939	TRUE	HP_393383	TGCTGTTG	CTCATTATC	XM_286918	Mus musculus hypothetical gene supported by AK084135 (LOC328996), mRNA.			2.9	SM2276	b		4	1.522164293
092308m3_V2MM_204747	1.29448	60.13437	TRUE	HP_422637	TGCTGTTG	GCCACAGA	XM_132795	gene model 154, (NCBI)	Gm154		2.1	SM2322	e		4	0.220877236
092308m3_V2MM_204747	1.29448	60.13437	TRUE	HP_422637	TGCTGTTG	GCCACAGA	XM_132795	gene model 154, (NCBI)	Gm154		2.1	SM2322	e		4	0.220877236
092308m1_V2MM_148152	1.294315	3.004939	TRUE	HP_368863	TGCTGTTG	GGTAAATA	XM_163535	Mus musculus LOC244134 (LOC244134), mRNA.			2.5	SM2052	f		11	1.522164293
092308m1_V2MM_148152	1.294315	3.004939	TRUE	HP_368863	TGCTGTTG	GGTAAATA	XM_163535	Mus musculus LOC244134 (LOC244134), mR.			2.5	SM2052	f		11	1.522164293
092308m1_V2MM_120042	1.294306	3.004939	TRUE	HP_340950	TGCTGTTG	CATGTCTT	XM_140732	Mus musculus similar to siah1A protein [Rattus norvegicus] (LOC226200), mR.			2.6	SM2101	b		11	1.522164293
mPool7_V2MM_78169	1.293934	3.004939	TRUE	HP_299867	TGCTGTTG	GACAAAGA	NM_023051	calsyntenin 1	Clstn1		2.8	SM2233	e		11	1.522164293
mPool4_V2MM_4912	1.293914	70.87591	TRUE	HP_228476	TGCTGTTG	CAGCCAAT	BC034893	RIKEN cDNA 3110037K17 gene	3110037K17Rik		2.15	SM2597	e		3	0.149501346
mPool4_V2MM_227	1.293673	75.83547	TRUE	HP_223902	TGCTGTTG	CAATGTGT	NM_027188	SET and MYND domain containing 3	Smyd3		2.15	SM2582	f		1	0.120127608
mPool2_V2MM_7217	1.293628	3.004939	TRUE	HP_230731	TGCTGTTG	GAGCTAAT	NM_026627	RIKEN cDNA 1700113O17 gene	1700113O17Rik		2.11	SM2376	e		6	1.522164293
mPool7_V2MM_136065	1.29294	1.959154	TRUE	HP_356785	TGCTGTTG	GCTGAATC	XM_151339	Mus musculus LOC211802 (LOC211802), mRNA.			2.4	SM2040	e		8	1.707931489
mPool7_V2MM_136065	1.29294	1.959154	TRUE	HP_356785	TGCTGTTG	GCTGAATC	XM_151339	Mus musculus LOC211802 (LOC211802), mR.			2.4	SM2040	e		8	1.707931489
092308m3_V2MM_141975	1.290409	59.99801	TRUE	HP_362688	TGCTGTTG	GATTATGT	XM_157245	Mus musculus LOC240234 (LOC240234), mR.			2.14	SM2525	e		3	0.221863149
092308m3_V2MM_82338	1.289881	75.83547	TRUE	HP_303917	TGCTGTTG	CCTACATG	NM_008400	integrin alpha L	Itgal		2.13	SM2468	e		3	0.120127608
mPool2_V2MM_118274	1.289156	3.004939	TRUE	HP_339211	TGCTGTTG	CAGTCATA	XM_139758	Mus musculus similar to formyl peptide receptor, related sequence 3 [Mus musculus] (L			2.11	SM2358	a		12	1.522164293
092308m1_V2MM_68971	1.28892	3.004939	TRUE	HP_290915	TGCTGTTG	GAGTTATA	NM_025510	RIKEN cD 2310004I24 gene	2310004I24Rik		2.4	SM2021	b		4	1.522164293
mPool4_V2MM_20367	1.288546	61.29828	TRUE	HP_243566	TGCTGTTG	GTGTTGTG	NM_153080	target of myb1-like 2 (chicken)	Tom1l2		2.16	SM2602	b		12	0.212551731
092308m3_V2MM_25306	1.288441	35.99881	TRUE	HP_248375	TGCTGTTG	CACTGACT	NM_007908	eukaryotic elongation factor-2 kise	Eef2k		2.12	SM2401	g		3	0.443711899
mPool2_V2MM_18541	1.288124	2.205692	TRUE	HP_241789	TGCTGTTG	GCTAAATG	NM_025820	Crn, crooked neck-like 1 (Drosophila)	Crnkl1		2.11	SM2377	g		7	1.656455229
mPool2_V2MM_160947	1.287844	2.205692	TRUE	HP_381524	TGCTGTTG	GTCCTGCTT	XM_484975	hypothetical gene supported by AK048278			2.6	SM2131	a		9	1.656455229
mPool2_V2MM_136212	1.287828	3.004939	TRUE	HP_356932	TGCTGTTG	GTCGAAAT	XM_151496	Mus musculus LOC241166 (LOC241166), mR.			2.4	SM2042	c		3	1.522164293
mPool2_V2MM_136212	1.287828	3.004939	TRUE	HP_356932	TGCTGTTG	GTCGAAAT	XM_151496	Mus musculus LOC241166 (LOC241166), mRNA.			2.4	SM2042	c		3	1.522164293
mPool7_V2MM_104283	1.287633	3.004939	TRUE	HP_325417	TGCTGTTG	GAACCAAT	XM_129655	cDNA sequence BC013667	BC013667		2.1	SM2304	h		9	1.522164293
mPool7_V2MM_104283	1.287633	3.004939	TRUE	HP_325417	TGCTGTTG	GAACCAAT	XM_129655	cD sequence BC013667	BC013667		2.1	SM2304	h		9	1.522164293
mPool7_V2MM_194879	1.28745	2.205692	TRUE	HP_413030	TGCTGTTG	CCCTTTCTT	NM_183126	RIKEN cDNA 6030498E09 gene	6030498E09Rik		2.9	SM2267	h		8	1.656455229
mPool7_V2MM_194879	1.28745	2.205692	TRUE	HP_413030	TGCTGTTG	CCCTTTCTT	NM_183126	RIKEN cD 6030498E09 gene	6030498E09Rik		2.9	SM2267	h		8	1.656455229
mPool6_V2MM_224007	1.285019	75.83547	TRUE	HP_476102	TGCTGTTG	GCTGTATT	XM_484861	similar to axonemal dynein heavy chain 7			2.7	SM2198	a		9	0.120127608
092308m1_V2MM_155374	1.284987	3.004939	TRUE	HP_376013	TGCTGTTG	GAGATAAA	XM_197402	Mus musculus LOC271565 (LOC271565), mR.			2.6	SM2122	g		12	1.522164293
092308m1_V2MM_174345	1.284919	3.004939	TRUE	HP_394830	TGCTGTTG	GATTTAAT	XM_287210	Mus musculus LOC329595 (LOC329595), mR.			2.5	SM2062	g		3	1.522164293
mPool5_V2MM_66560	1.284912	61.29828	TRUE	HP_288573	TGCTGTTG	CAGGGTTT	NM_010323	gonadotropin releasing hormone receptor	Gnrhr	NA				NA		0.212551731
092308m3_V2MM_237569	1.28491	66.95036	TRUE	HP_489415	TGCTGTTG	GAGCTTCT	XM_155441	Mus musculus LOC239511 (LOC239511), mR.			2.14	SM2524	a		3	0.1742471
092308m1_V2MM_187569	1.284424	2.205692	TRUE	HP_408034	TGCTGTTG	CATTTAATT	XM_290006	Mus musculus similar to pol polyprotein [Trichosurus vulpecula] (LOC333873), mR.			2.5	SM2065	f		3	1.656455229
mPool2_V2MM_28070	1.282798	2.205692	TRUE	HP_251065	TGCTGTTG	GCAATAAC	NM_030730	steroid receptor-interacting SNF2 domain protein			2.11	SM2391	f		10	1.656455229
mPool6_V2MM_82021	1.282521	59.99801	TRUE	HP_303607	TGCTGTTG	GTGTTTCTC	NM_146752	olfactory receptor 1106	Olfr1106		2.7	SM2179	a		6	0.221863149
092308m1_V2MM_161814	1.282315	3.004939	TRUE	HP_382378	TGCTGTTG	CACCTGGT	AK122544	D segment, Chr 5, Wayne State University 178, expressed	D5Wsu178e		2.6	SM2115	h		11	1.522164293
092308m1_V2MM_176186	1.282285	3.004939	TRUE	HP_396668	TGCTGTTG	GAAGCTAT	XM_287619	Mus musculus hypothetical gene supported by AK035337 (LOC330373), mR.			2.5	SM2094	a		8	1.522164293
092308m1_V2MM_66050	1.281985	3.004939	TRUE	HP_80906	TGCTGTTG	GTGCTTTAT	NM_010446	forkhead box A2	Foxa2		2.4	SM2027	f		10	1.522164293
092308m1_V2MM_163750	1.281981	2.205692	TRUE	HP_384305	TGCTGTTG	CCATATAA	XM_284847	Mus musculus similar to KIAA1466 protein [Homo sapiens] (LOC329320), mR.			2.6	SM2116	f		2	1.656455229
mPool5_V2MM_912	1.281942	66.95036	TRUE	HP_224568	TGCTGTTG	CAGGCTGC	NM_007808	cytochrome c, somatic	Cycs	NA				NA		0.1742471
mPool2_V2MM_101104	1.281923	2.205692	TRUE	HP_322279	TGCTGTTG	CTTACTTG	AK006112	RIKEN cDNA 0610007P08 gene	0610007P08Rik		2.6	SM2150	c		4	1.656455229
mPool7_V2MM_187236	1.281511	2.205692	TRUE	HP_407705	TGCTGTTG	CATAGTTA	XM_289671	Mus musculus LOC333434 (LOC333434), mRNA.			2.9	SM2286	g		7	1.656455229
092308m3_V2MM_116020	1.280604	75.83547	TRUE	HP_336977	TGCTGTTG	CATCCAAG	XM_138719	Mus musculus similar to endogenous retroviral family W, env(C7), member 1 (syncytin);			2.14	SM2508	f		12	0.120127608
mPool4_V2MM_131730	1.280494	75.83547	TRUE	HP_352490	TGCTGTTG	GTCCGTGT	AK014636	RIKEN cDNA 4733401I05 gene	4733401I05Rik		2.16	SM2631	c		4	0.120127608
mPool6_V2MM_238650	1.279919	75.83547	TRUE	HP_474866	TGCTGTTG	CCCACTGC	XM_141448	Mus musculus similar to protease [Trichosurus vulpecula] (LOC212819), mRNA.			2.8	SM2212	e		8	0.120127608
mPool2_V2MM_30527	1.279875	3.004939	TRUE	HP_253451	TGCTGTTG	CGGAAACT	NM_145492	zinc finger protein 521	Zfp521		2.11	SM2389	g		10	1.522164293
092308m3_V2MM_111611	1.279309	59.99801	TRUE	HP_332623	TGCTGTTG	CGCTATCT	XM_135982	RIKEN cD 6530406P05 gene	6530406P05Rik		2.13	SM2491	h		12	0.221863149

092308m1_V2MM_148248	1.279012	3.004939	TRUE	HP_368959	TGCTGTTG	CACAGGAC	XM_163622	Mus musculus LOC207376 (LOC207376), mRNA.			2.5	SM2056	f		2	1.522164293
092308m1_V2MM_148248	1.279012	3.004939	TRUE	HP_368959	TGCTGTTG	CACAGGAC	XM_163622	Mus musculus LOC207376 (LOC207376), mR.			2.5	SM2056	f		2	1.522164293
mPool2_V2MM_212991	1.278948	3.004939	TRUE	HP_430643	TGCTGTTG	CAGCTTTG	XM_488592	RIKEN cDNA 5133400J02 gene	5133400J02Rik		2.11	SM2352	e		9	1.522164293
092308m3_V2MM_47599	1.278706	75.83547	TRUE	HP_270076	TGCTGTTG	CTGTCTGC	NM_080846	RIKEN cD 2310056K19 gene	2310056K19Rik		2.12	SM2405	d		12	0.120127608
092308m1_V2MM_137567	1.278446	3.004939	TRUE	HP_358287	TGCTGTTG	CCAGGACA	XM_153307	Mus musculus LOC208340 (LOC208340), mR.			2.5	SM2053	g		9	1.522164293
092308m1_V2MM_178239	1.277698	3.004939	TRUE	HP_398713	TGCTGTTG	CACCAAGC	AK032250	hypothetical protein 6430511F03			2.5	SM2086	f		5	1.522164293
092308m3_V2MM_95294	1.276198	61.29828	TRUE	HP_316570	TGCTGTTG	CAGCCTAT	NM_197943	RUN and TBC1 domain containing 1	Rutbc1		2.9	SM2254	d		3	0.212551731
092308m3_V2MM_95294	1.276198	61.29828	TRUE	HP_316570	TGCTGTTG	CAGCCTAT	NM_197943	RUN and TBC1 domain containing 1	Rutbc1		2.9	SM2254	d		3	0.212551731
092308m3_V2MM_93163	1.275389	75.83547	TRUE	HP_314493	TGCTGTTG	GTCTGGAT	NM_178919	expressed sequence AI451006	AI451006		2.13	SM2473	e		2	0.120127608
mPool4_V2MM_227676	1.275148	75.83547	TRUE	HP_479668	TGCTGTTG	GTATTAAT	XM_286653	Mus musculus hypothetical gene supported by AK038948 (LOC328512), mRNA.		NA				NA		0.120127608
092308m3_V2MM_95830	1.274739	75.83547	TRUE	HP_317098	TGCTGTTG	CTTGTGTG	AK047814	RIKEN cD A730024A03 gene	A730024A03Rik		2.12	SM2449	d		5	0.120127608
mPool2_V2MM_121209	1.274586	2.205692	TRUE	HP_342091	TGCTGTTG	CACAAATA	XM_141541	similar to 40S ribosomal protein S16			2.11	SM2369	h		5	1.656455229
092308m3_V2MM_224463	1.274567	59.99801	TRUE	HP_476532	TGCTGTTG	GCATCGAC	AK032385	D segment, Chr 14, ERATO Doi 171, expressed	D14Ertd171e		2.13	SM2482	h		2	0.221863149
mPool4_V2MM_214845	1.274325	75.83547	TRUE	HP_180329	TGCTGTTG	GAAGCTAT	XM_486245	similar to Rpl7a protein			2.1	SM2308	h		9	0.120127608
092308m1_V2MM_169374	1.274293	2.205692	TRUE	HP_389869	TGCTGTTG	CAAATATG	AK045844	hypothetical protein B230314J19			2.6	SM2123	d		10	1.656455229
mPool7_V2MM_185237	1.273848	3.004939	TRUE	HP_405709	TGCTGTTG	GAGCTAAA	XM_289336	Mus musculus LOC333017 (LOC333017), mRNA.			2.9	SM2272	h		8	1.522164293
mPool5_V2MM_7199	1.273055	75.83547	TRUE	HP_230713	TGCTGTTG	GAGCCAGT	NM_054085	expressed sequence AW319487	AW319487	NA				NA		0.120127608
092308m3_V2MM_110527	1.273049	75.83547	TRUE	HP_331561	TGCTGTTG	CAGTTGAA	XM_134736	zinc finger protein 26	Zfp26		2.13	SM2500	c		10	0.120127608
mPool7_V2MM_152047	1.271704	3.004939	TRUE	HP_372714	TGCTGTTG	GCAAATGA	XM_195063	Mus musculus similar to potassium channel regulatory factor (LOC271501), mRNA.			2.1	SM2325	e		6	1.522164293
092308m3_V2MM_225789	1.271662	75.83547	TRUE	HP_477828	TGCTGTTG	CACGAGGC	XM_125996	similar to SEC14-like protein 3 (45 kDa secretory protein) (rsec45)			2.13	SM2479	c		6	0.120127608
mPool2_V2MM_125119	1.271155	3.004939	TRUE	HP_345963	TGCTGTTG	GAAGAAGT	XM_143807	Mus musculus similar to alpha-2u-globulin IV precursor - mouse (LOC209155), mRNA.			2.11	SM2368	h		9	1.522164293
mPool7_V2MM_110117	1.271136	3.004939	TRUE	HP_331158	TGCTGTTG	GTGATATT	XM_134391	Mus musculus nuclear factor of activated T-cells, cytoplasmic 3 (Nfatc3), mRNA.			2.9	SM2299	d		4	1.522164293
092308m3_V2MM_86482	1.270881	66.95036	TRUE	HP_307957	TGCTGTTG	CCCTCAGG	NM_172601	RAB2B, member RAS oncogene family	Rab2b		2.13	SM2471	a		10	0.1742471
mPool4_V2MM_209512	1.270453	75.83547	TRUE	HP_427282	TGCTGTTG	CTCCACAT	XM_195276	Mus musculus similar to hypothetical protein [Plasmodium falciparum 3D7] (LOC27053		NA				NA		0.120127608
092308m1_V2MM_163858	1.270306	3.004939	TRUE	HP_384411	TGCTGTTG	CAGTTGGT	XM_284870	Mus musculus similar to hypothetical protein D930020E02 [Mus musculus] (LOC327754			2.6	SM2123	c		9	1.522164293
mPool2_V2MM_107492	1.270014	2.205692	TRUE	HP_328576	TGCTGTTG	GAATCGAA	XM_132134	RIKEN cDNA 1110019K23 gene	1110019K23Rik		2.6	SM2149	f		7	1.656455229
mPool2_V2MM_44691	1.270002	3.004939	TRUE	HP_267247	TGCTGTTG	GCATTGGC	NM_009692	apolipoprotein A-I	Apoa1		2.7	SM2165	b		6	1.522164293
mPool2_V2MM_21454	1.269953	2.205692	TRUE	HP_244627	TGCTGTTG	CAGTAGTA	NM_027722	nudix (nucleoside diphosphate linked moiety X)-type motif 4	Nudt4		2.11	SM2390	d		10	1.656455229
092308m3_V2MM_51736	1.269804	75.83547	TRUE	HP_274129	TGCTGTTG	CTGACTTG	NM_138683	thrombospondin type 1 domain containing gene			2.12	SM2409	c		3	0.120127608
092308m3_V2MM_111320	1.26953	75.83547	TRUE	HP_332337	TGCTGTTG	CATAGGAG	XM_135683	similar to tripartite motif-containing 43			2.1	SM2302	e		11	0.120127608
092308m3_V2MM_111320	1.26953	75.83547	TRUE	HP_332337	TGCTGTTG	CATAGGAG	XM_135683	similar to tripartite motif-containing 43			2.1	SM2302	e		11	0.120127608
092308m3_V2MM_20561	1.269264	75.83547	TRUE	HP_243754	TGCTGTTG	GAATGGCA	NM_030734	defensin beta 5	Defb5		2.12	SM2434	e		11	0.120127608
092308m1_V2MM_136398	1.268689	2.205692	TRUE	HP_357118	TGCTGTTG	GTTCTTCAC	XM_151696	Mus musculus LOC237328 (LOC237328), mR.			2.4	SM2043	e		12	1.656455229
092308m1_V2MM_161461	1.268399	3.004939	TRUE	HP_382025	TGCTGTTG	GCCAGAAT	XM_283928	Mus musculus hypothetical gene supported by AK042285 (LOC329760), mR.			2.6	SM2120	b		12	1.522164293
mPool7_V2MM_66155	1.268317	3.004939	TRUE	HP_288177	TGCTGTTG	GTTGAAGA	NM_025630	RIKEN cDNA 2010009L17 gene	2010009L17Rik		2.8	SM2231	h		1	1.522164293
mPool5_V2MM_230540	1.268193	75.83547	TRUE	HP_482525	TGCTGTTG	GATGTGTG	AK079812	RIKEN cDNA 9130227L01 gene	9130227L01Rik	NA				NA		0.120127608
092308m1_V2MM_127737	1.266666	3.004939	TRUE	HP_348559	TGCTGTTG	GTGACTAA	XM_145359	Mus musculus similar to vomeronasal 1 receptor, G1 [Mus musculus] (LOC232868), mR			2.6	SM2114	c		4	1.522164293
092308m1_V2MM_127737	1.266666	3.004939	TRUE	HP_348559	TGCTGTTG	GTGACTAA	XM_145359	Mus musculus similar to vomerosal 1 receptor, G1 [Mus musculus] (LOC232868), mR.			2.6	SM2114	c		4	1.522164293
mPool4_V2MM_125960	1.266542	75.83547	TRUE	HP_346803	TGCTGTTG	CTACTGAT	XM_144307	Mus musculus similar to 40S ribosomal protein S2 (LOC231294), mRNA.			2.14	SM2538	b		11	0.120127608
mPool2_V2MM_124154	1.266422	3.004939	TRUE	HP_345006	TGCTGTTG	GAAAGTAA	XM_143326	Mus musculus LOC229387 (LOC229387), mRNA.			2.11	SM2371	b		5	1.522164293
mPool7_V2MM_179995	1.265819	3.004939	TRUE	HP_400469	TGCTGTTG	CATATTTA	XM_288432	Mus musculus LOC331767 (LOC331767), mR.			2.5	SM2069	a		2	1.522164293
mPool7_V2MM_179995	1.265819	3.004939	TRUE	HP_400469	TGCTGTTG	CATATTTA	XM_288432	Mus musculus LOC331767 (LOC331767), mRNA.			2.5	SM2069	a		2	1.522164293
mPool5_V2MM_52592	1.265314	62.64426	TRUE	HP_274969	TGCTGTTG	GAGTATGT	NM_008970	parathyroid hormone-like peptide	Pthlh	NA				NA		0.203118686
mPool2_V2MM_99002	1.264925	2.205692	TRUE	HP_320218	TGCTGTTG	GTCCAATT	AK013759	RIKEN cDNA 2900069M18 gene	2900069M18Rik		2.6	SM2147	a		7	1.656455229
092308m1_V2MM_181021	1.264763	3.004939	TRUE	HP_401495	TGCTGTTG	CAGATTAG	XM_288605	Mus musculus LOC332003 (LOC332003), mRNA.			2.5	SM2095	d		3	1.522164293
092308m1_V2MM_181021	1.264763	3.004939	TRUE	HP_401495	TGCTGTTG	CAGATTAG	XM_288605	Mus musculus LOC332003 (LOC332003), mR.			2.5	SM2095	d		3	1.522164293
mPool5_V2MM_211	1.26442	75.83547	TRUE	HP_223886	TGCTGTTG	CAATGAAG	NM_008211	H3 histone, family 3B	H3f3b	NA				NA		0.120127608
mPool6_V2MM_65669	1.264243	75.83547	TRUE	HP_287703	TGCTGTTG	GGCTGCAG	NM_172908	RIKEN cDNA 9230106D23 gene	9230106D23Rik		2.7	SM2181	g		3	0.120127608
mPool5_V2MM_159885	1.264116	75.83547	TRUE	HP_380478	TGCTGTTG	CTGGCCTC	XM_283291	Mus musculus hypothetical gene supported by AK027976 (LOC328554), mRNA.		NA				NA		0.120127608
092308m3_V2MM_103042	1.26304	75.83547	TRUE	HP_324187	TGCTGTTG	GATACCGT	XM_484662	WD repeat domain 43	Wrd43		2.13	SM2490	f		6	0.120127608
mPool7_V2MM_76348	1.263026	2.205692	TRUE	HP_298108	TGCTGTTG	CGCCTTCTA	NM_011351	sema domain, transmembrane domain (TM), and cytoplasmic	Sema6c		2.8	SM2234	h		3	1.656455229
mPool2_V2MM_163010	1.262763	3.004939	TRUE	HP_383568	TGCTGTTG	GTAGAAAC	XM_284594	Mus musculus similar to RIKEN cDNA D130040H23; hypothetical protein D130040H23 [			2.6	SM2130	g		3	1.522164293
mPool2_V2MM_118336	1.262731	3.004939	TRUE	HP_339273	TGCTGTTG	GATATACC	XM_139794	Mus musculus similar to calcium-sensing receptor related protein 3 (LOC224578), mRN			2.1	SM2350	b		5	1.522164293
mPool7_V2MM_203135	1.262663	3.004939	TRUE	HP_421102	TGCTGTTG	CCTGATGG	XM_131689	RIKEN cDNA 4930429A08 gene	4930429A08Rik		2.1	SM2315	f		10	1.522164293
092308m1_V2MM_179069	1.261419	3.004939	TRUE	HP_399543	TGCTGTTG	CTATTAAA	XM_288269	Mus musculus LOC332542 (LOC332542), mRNA.			2.5	SM2091	c		7	1.522164293
092308m1_V2MM_179069	1.261419	3.004939	TRUE	HP_399543	TGCTGTTG	CTATTAAA	XM_288269	Mus musculus LOC332542 (LOC332542), mR.			2.5	SM2091	c		7	1.522164293
092308m3_V2MM_91853	1.260797	75.83547	TRUE	HP_313220	TGCTGTTG	CTGCAATT	NM_178605	D segment, Chr 13, Wayne State University 177, expressed	D13Wsu177e		2.13	SM2458	f		8	0.120127608
092308m3_V2MM_93732	1.260601	75.83547	TRUE	HP_315036	TGCTGTTG	CGAAATGA	NM_181748	G protein-coupled receptor 120	Gpr120		2.9	SM2263	e		11	0.120127608
092308m3_V2MM_93732	1.260601	75.83547	TRUE	HP_315036	TGCTGTTG	CGAAATGA	NM_181748	G protein-coupled receptor 120	Gpr120		2.9	SM2263	e		11	0.120127608
092308m3_V2MM_38908	1.26049	62.64426	TRUE	HP_261613	TGCTGTTG	GCCTTGGA	NM_146242	leucine rich repeat containing 10	Lrrc10		2.12	SM2421	a		6	0.203118686
092308m3_V2MM_194705	1.260414	75.83547	TRUE	HP_412859	TGCTGTTG	GAAATCAG	NM_178119	centaurin, gamma 2	Centg2		2.13	SM2478	b		9	0.120127608
mPool2_V2MM_215236	1.259445	2.205692	TRUE	HP_432795	TGCTGTTG	CCTGTTAC	XM_145781	Mus musculus similar to olfactory receptor MOR23-1 [Mus musculus] (LOC233600), mR			2.11	SM2366	b		1	1.656455229

092308m3_V2MM_148738	1.259292	75.83547	TRUE	HP_369449	TGCTGTTG/	CCAAGTGT	XM_164093	Mus musculus LOC244445 (LOC244445), mR.		2.14	SM2517	d	4	0.120127608
mPool7_V2MM_77900	1.259168	2.205692	TRUE	HP_299607	TGCTGTTG/	GAAACGTG	NM_029362	RIKEN cDNA 2010012F05 gene	2010012F05Rik	2.8	SM2248	c	5	1.656455229
mPool7_V2MM_93273	1.258875	3.004939	TRUE	HP_314600	TGCTGTTG/	GTCCTTTGT	NM_180958	RIKEN cDNA 4930532D21 gene	4930532D21Rik	2.9	SM2269	a	12	1.522164293
092308m1_V2MM_95054	1.258243	3.004939	TRUE	HP_316337	TGCTGTTG/	GCAGTTAA(	NM_183313	hypothetical protein D930018N13		2.6	SM2113	h	7	1.522164293
092308m1_V2MM_171454	1.25819	3.004939	TRUE	HP_391943	TGCTGTTG/	CCAAGTAC/	AK048966	RIKEN cD C230086J09 gene	C230086J09Rik	2.5	SM2086	f	2	1.522164293
092308m1_V2MM_122466	1.257098	3.004939	TRUE	HP_343332	TGCTGTTG/	GAGAGCTT	BC053004	glycosyltransferase 28 domain containing 1	Glt28d1	2.6	SM2101	f	11	1.522164293
mPool2_V2MM_83049	1.257038	2.205692	TRUE	HP_304604	TGCTGTTG/	CTGGGAAT	NM_011267	regulator of G-protein signaling 16	Rgs16	2.6	SM2138	a	4	1.656455229
092308m1_V2MM_167663	1.256768	3.004939	TRUE	HP_388176	TGCTGTTG/	GGCTAAGT	XM_285812	Mus musculus similar to protease [Mus musculus] (LOC329865), mR.		2.6	SM2124	g	11	1.522164293
mPool7_V2MM_170526	1.256519	2.205692	TRUE	HP_391019	TGCTGTTG/	GTGTCTAC/	AK034287	leucine rich repeat and fibronectin type III domain containing	Lrfn5	2.9	SM2276	a	8	1.656455229
092308m3_V2MM_60972	1.255802	75.83547	TRUE	HP_283119	TGCTGTTG/	GTCCTGAA/	NM_009503	valosin containing protein	Vcp	2.12	SM2412	b	2	0.120127608
mPool2_V2MM_107743	1.255407	2.205692	TRUE	HP_328825	TGCTGTTG/	CACGGAAA	AK016965	RIKEN cDNA 4933428F06 gene	4933428F06Rik	2.7	SM2158	f	1	1.656455229
092308m3_V2MM_105467	1.255256	75.83547	TRUE	HP_326582	TGCTGTTG/	GTGGTCCA(	XM_130523	D segment, Chr 2, Brigham & Womens Genetics 1356 express	D2Bwg1356e	2.14	SM2508	e	11	0.120127608
mPool2_V2MM_157758	1.254475	3.004939	TRUE	HP_378371	TGCTGTTG/	CTATAAAT(	XM_205565	RIKEN cDNA 4930526H21 gene	4930526H21Rik	2.6	SM2126	b	2	1.522164293
mPool2_V2MM_129889	1.254189	3.004939	TRUE	HP_350690	TGCTGTTG/	GAAGAAAT	BC041776	RIKEN cDNA D630040G17 gene	D630040G17Rik	2.11	SM2371	e	8	1.522164293
092308m3_V2MM_234254	1.25414	75.83547	TRUE	HP_486136	TGCTGTTG/	CCACTTCTC	XM_136417	hypothetical LOC268730		2.13	SM2499	h	1	0.120127608
mPool6_V2MM_155870	1.254106	75.83547	TRUE	HP_376509	TGCTGTTG/	CAGGAGAA	NM_181857	DNA polymerase N	Poln	2.7	SM2190	h	9	0.120127608
mPool2_V2MM_62998	1.25371	2.205692	TRUE	HP_285088	TGCTGTTG/	CCGAGACA	NM_008087	growth arrest specific 2	Gas2	2.7	SM2166	d	5	1.656455229
092308m3_V2MM_38939	1.253641	35.99881	TRUE	HP_261642	TGCTGTTG/	GCGACTCT(	NM_007996	ferredoxin 1	Fdx1	2.11	SM2394	a	10	0.443711899
092308m1_V2MM_187100	1.253581	3.004939	TRUE	HP_407569	TGCTGTTG/	CTGAGACA.	XM_289649	Mus musculus LOC333411 (LOC333411), mR.		2.5	SM2070	d	2	1.522164293
mPool7_V2MM_79004	1.253445	3.004939	TRUE	HP_300679	TGCTGTTG/	GAGTATTC(	NM_025479	RIKEN cDNA 2810021B07 gene	2810021B07Rik	2.8	SM2246	h	7	1.522164293
mPool2_V2MM_120546	1.253206	3.004939	TRUE	HP_341440	TGCTGTTG/	GTGAAATT	XM_140991	Mus musculus similar to ribosomal protein S6 [Gallus gallus] (LOC241487), mRNA.		2.11	SM2354	c	10	1.522164293
mPool7_V2MM_109298	1.252512	3.004939	TRUE	HP_330360	TGCTGTTG/	GAAGAGAT	XM_355946	RIKEN cDNA C030048B12 gene	C030048B12Rik	2.1	SM2310	b	8	1.522164293
mPool2_V2MM_212633	1.252406	3.004939	TRUE	HP_430297	TGCTGTTG/	GGAACGTA	XM_140116	similar to ribosomal protein S24		2.11	SM2352	h	9	1.522164293
mPool7_V2MM_75847	1.252022	3.004939	TRUE	HP_297619	TGCTGTTG/	CCGGATAT	NM_033560	DNA segment, Chr 8, ERATO Doi 531, expressed	D8Ertd531e	2.8	SM2236	e	9	1.522164293
mPool7_V2MM_98250	1.251865	3.004939	TRUE	HP_319481	TGCTGTTG/	GGACAAGA	XM_112298	similar to RIKEN cDNA 1700029I01		2.9	SM2258	f	5	1.522164293
092308m3_V2MM_45265	1.2518	59.99801	TRUE	HP_267804	TGCTGTTG/	GGTCAAGC	NM_010155	Ets2 repressor factor	Erf	2.12	SM2437	a	7	0.221863149
092308m1_V2MM_74261	1.251215	2.205692	TRUE	HP_296067	TGCTGTTG/	CACAGTAG	NM_183120	RIKEN cD 1110048D14 gene	1110048D14Rik	2.4	SM2024	h	2	1.656455229
092308m1_V2MM_62780	1.251181	3.004939	TRUE	HP_284878	TGCTGTTG/	CATCTTATG	NM_146748	olfactory receptor 661	Olfr661	2.4	SM2026	f	8	1.522164293
mPool4_V2MM_225529	1.250858	75.83547	TRUE	HP_477575	TGCTGTTG/	CTTTGGTCT	NM_178386	RIKEN cDNA 1700034J06 gene	1700034J06Rik	2.16	SM2605	b	12	0.120127608
092308m3_V2MM_236006	1.250853	62.64426	TRUE	HP_487867	TGCTGTTG/	CTTCCCAG/	XM_150399	Mus musculus LOC208932 (LOC208932), mR.		2.14	SM2519	a	6	0.203118686
092308m1_V2MM_61824	1.250278	3.004939	TRUE	HP_283946	TGCTGTTG/	CAATCAGA(	NM_176900	RIKEN cD C730034F03 gene	C730034F03Rik	2.3	SM2009	f	11	1.522164293
mPool2_V2MM_9768	1.250272	3.004939	TRUE	HP_233219	TGCTGTTG/	CATTGGAA/	NM_008444	kinesin family member 3B	Kif3b	2.11	SM2385	g	1	1.522164293
mPool2_V2MM_9754	1.25027	3.004939	TRUE	HP_233205	TGCTGTTG/	CATTCCCTC	NM_011660	thioredoxin 1	Txn1	2.11	SM2387	f	1	1.522164293
mPool2_V2MM_111510	1.249649	3.004939	TRUE	HP_332523	TGCTGTTG/	CGCTGAAC.	AK018320	RIKEN cDNA 6530403M18 gene	6530403M18Rik	2.7	SM2153	d	10	1.522164293
mPool2_V2MM_18604	1.249593	3.004939	TRUE	HP_241851	TGCTGTTG/	GCTATTGCT	NM_144559	Fc receptor-like 3	Fcrl3	2.11	SM2385	f	5	1.522164293
092308m3_V2MM_143023	1.249406	75.83547	TRUE	HP_363736	TGCTGTTG/	GTGAAGAG	XM_158268	Mus musculus LOC241344 (LOC241344), mR.		2.14	SM2526	f	9	0.120127608
mPool7_V2MM_96180	1.249143	3.004939	TRUE	HP_317440	TGCTGTTG/	CCTATTATT	XM_110248	F-box protein 11	Fbxo11	2.9	SM2268	a	8	1.522164293
092308m3_V2MM_196430	1.247642	75.83547	TRUE	HP_414534	TGCTGTTG/	CACTGTTT/	NM_183311	hypothetical protein B930076A02		2.13	SM2477	f	4	0.120127608
092308m1_V2MM_125452	1.247425	2.205692	TRUE	HP_346296	TGCTGTTG/	CTGTCTAAT	XM_144012	Mus musculus similar to beta-adaptin - rat (LOC230638), mRNA.		2.6	SM2102	h	5	1.656455229
092308m1_V2MM_125452	1.247425	2.205692	TRUE	HP_346296	TGCTGTTG/	CTGTCTAAT	XM_144012	Mus musculus similar to beta-adaptin - rat (LOC230638), mR.		2.6	SM2102	h	5	1.656455229
mPool2_V2MM_213180	1.247231	3.004939	TRUE	HP_430822	TGCTGTTG/	CTACTTAAA	XM_142166	Mus musculus similar to ribosomal protein S7 (LOC245623), mRNA.		2.11	SM2373	a	9	1.522164293
092308m1_V2MM_170513	1.247013	1.959154	TRUE	HP_391006	TGCTGTTG/	GCATAGTT(	XM_488637	hypothetical gene supported by AK087847		2.5	SM2062	d	7	1.707931489
mPool4_V2MM_30524	1.24668	75.83547	TRUE	HP_253448	TGCTGTTG/	CGCTTTGC(	NM_024177	mitochondrial ribosomal protein L38	Mrpl38	2.15	SM2572	f	6	0.120127608
092308m3_V2MM_232423	1.246578	75.83547	TRUE	HP_484369	TGCTGTTG/	CATATACT(	XM_111313	similar to small nuclear R		2.13	SM2500	h	12	0.120127608
mPool2_V2MM_169983	1.246037	3.004939	TRUE	HP_390478	TGCTGTTG/	CTCATAAT/	XM_286370	Mus musculus hypothetical gene supported by AK086479 (LOC327911), mRNA.		2.6	SM2128	g	5	1.522164293
092308m3_V2MM_89430	1.245931	62.64426	TRUE	HP_165858	TGCTGTTG/	CTTGTTGA/	NM_177595	RIKEN cD 9430023B20 gene	9430023B20Rik	2.13	SM2456	h	12	0.203118686
092308m3_V2MM_233661	1.245839	70.87591	TRUE	HP_485566	TGCTGTTG/	CCGAAGCA	XM_133622	hepatitis B virus x associated protein	Hbxap	2.13	SM2498	h	4	0.149501346
mPool2_V2MM_12638	1.245531	3.004939	TRUE	HP_143760	TGCTGTTG/	CACAGTGT	NM_011593	tissue inhibitor of metalloproteinase 1	Timp1	2.11	SM2380	g	11	1.522164293
092308m3_V2MM_30237	1.244755	75.83547	TRUE	HP_253176	TGCTGTTG/	CATCTGGA/	BC065171	CCR4-NOT transcription complex, subunit 2	Cnot2	2.12	SM2424	c	5	0.120127608
092308m1_V2MM_63866	1.24412	3.004939	TRUE	HP_285933	TGCTGTTG/	CTCCACCTC	NM_025786	RIKEN cD 9130020G10 gene	9130020G10Rik	2.3	SM2016	e	7	1.522164293
mPool2_V2MM_15761	1.243947	2.205692	TRUE	HP_239078	TGCTGTTG/	CTGCTAAT(	NM_008372	interleukin 7 receptor	Il7r	2.11	SM2379	f	6	1.656455229
mPool2_V2MM_132868	1.243817	3.004939	TRUE	HP_353616	TGCTGTTG/	GGAAGGAA	AK015981	RIKEN cDNA 4930535F04 gene	4930535F04Rik	2.11	SM2371	c	6	1.522164293
mPool4_V2MM_226810	1.243374	61.29828	TRUE	HP_478819	TGCTGTTG/	CCTAAGCA(	NM_177827	hypothetical protein 9530053H22		2.16	SM2624	h	4	0.212551731
mPool7_V2MM_93408	1.243225	3.004939	TRUE	HP_314724	TGCTGTTG/	CTGCAATT/	NM_181392	DNA segment, Chr 17, Wayne State University 155, expressed	D17Wsu155e	2.9	SM2257	e	2	1.522164293
092308m3_V2MM_234885	1.24206	75.83547	TRUE	HP_486755	TGCTGTTG/	CTATTGTGT	XM_153848	Mus musculus LOC238501 (LOC238501), mR.		2.14	SM2514	c	1	0.120127608
092308m1_V2MM_177109	1.241297	3.004939	TRUE	HP_397589	TGCTGTTG/	GTGTAAAT	XM_287805	Mus musculus hypothetical gene supported by AK032925 (LOC330762), mR.		2.5	SM2063	h	6	1.522164293
092308m1_V2MM_177109	1.241297	3.004939	TRUE	HP_397589	TGCTGTTG/	GTGTAAAT	XM_287805	Mus musculus hypothetical gene supported by AK032925 (LOC330762), mRNA.		2.5	SM2063	h	6	1.522164293
mPool4_V2MM_47449	1.241049	75.83547	TRUE	HP_269930	TGCTGTTG/	CTGCCATT(	NM_175441	RIKEN cDNA D830007F02 gene	D830007F02Rik	2.16	SM2622	d	6	0.120127608
mPool7_V2MM_204911	1.240922	3.004939	TRUE	HP_422795	TGCTGTTG/	CCAAGAAG	XM_138950	Mus musculus LOC239003 (LOC239003), mRNA.		2.1	SM2306	b	9	1.522164293
092308m3_V2MM_48991	1.240044	75.83547	TRUE	HP_271441	TGCTGTTG/	GCTCCAGT/	NM_145927	farnesyltransferase, CAAX box, beta	Fntb	2.12	SM2446	f	2	0.120127608
mPool6_V2MM_46500	1.240012	59.99801	TRUE	HP_269000	TGCTGTTG/	CCCTGCTC(	NM_153805	protein kinase N3	Pkn3	2.7	SM2186	a	1	0.221863149
mPool6_V2MM_166521	1.239925	73.80895	TRUE	HP_387049	TGCTGTTG/	CATAAGCA(	XM_285524	gene model 1539, (NCBI)	Gm1539	2.7	SM2192	d	12	0.131891001

mPool2_V2MM_100265	1.239674	3.004939	TRUE	HP_321457	TGCTGTTG/	CCGCCATC(	BC025575	cDNA sequence BC025575	BC025575		2.7	SM2160	h		8	1.522164293
092308m1_V2MM_142060	1.239671	3.004939	TRUE	HP_362773	TGCTGTTG/	GATGTTCA1	XM_157294	Mus musculus LOC240262 (LOC240262), mR.			2.4	SM2032	d		8	1.522164293
mPool2_V2MM_139300	1.239618	3.004939	TRUE	HP_360020	TGCTGTTG/	GACAGCAT	XM_154905	Mus musculus LOC210787 (LOC210787), mRNA.			2.1	SM2334	h		12	1.522164293
mPool7_V2MM_205425	1.238526	3.004939	TRUE	HP_423289	TGCTGTTG/	CCGAGAGT	XM_128377	RIKEN cDNA 4921511H13 gene	4921511H13Rik		2.1	SM2303	b		3	1.522164293
mPool5_V2MM_180385	1.238283	66.95036	TRUE	HP_400859	TGCTGTTG/	CACAGTCC(	XM_288498	Mus musculus LOC328105 (LOC328105), mRNA.		NA				NA		0.1742471
092308m1_V2MM_69811	1.238273	3.004939	TRUE	HP_291735	TGCTGTTG/	GTAGGGAA	NM_010459	homeo box B4	Hoxb4		2.3	SM2005	g		7	1.522164293
mPool2_V2MM_151163	1.236129	3.004939	TRUE	HP_371834	TGCTGTTG/	GGGATGAT	NM_001004364	gene model 592, (NCBI)	Gm592		2.1	SM2343	a		12	1.522164293
092308m3_V2MM_203136	1.236094	75.83547	TRUE	HP_421103	TGCTGTTG/	GCCTCATT(	XM_129935	Mus musculus similar to nuclear pore-targeting complex component of 58 kDa (LOC227			2.9	SM2299	c		4	0.120127608
092308m3_V2MM_203136	1.236094	75.83547	TRUE	HP_421103	TGCTGTTG/	GCCTCATT(	XM_129935	Mus musculus similar to nuclear pore-targeting complex component of 58 kDa (LOC227			2.9	SM2299	c		4	0.120127608
mPool6_V2MM_167391	1.235898	75.83547	TRUE	HP_387904	TGCTGTTG/	GACTACAT(	XM_285759	Mus musculus similar to tumor protein, translationally-controlled 1 [Rattus norvegicus]			2.7	SM2194	b		5	0.120127608
mPool2_V2MM_94842	1.235321	3.004939	TRUE	HP_316125	TGCTGTTG/	CCCAGTTG/	NM_183269	RIKEN cDNA 4930511N19 gene	4930511N19Rik		2.6	SM2136	b		9	1.522164293
092308m1_V2MM_151402	1.234206	2.205692	TRUE	HP_372073	TGCTGTTG/	CCATAGCT	XM_194738	Mus musculus similar to ATP synthase, H+ transporting, mitochondrial F0 complex, subu			2.5	SM2059	e		5	1.656455229
092308m3_V2MM_233077	1.234182	75.83547	TRUE	HP_485000	TGCTGTTG/	GTCTGTCT(	AK021026	RIKEN cD B430319H21 gene	B430319H21Rik		2.13	SM2500	d		1	0.120127608
092308m1_V2MM_183706	1.233168	2.205692	TRUE	HP_404180	TGCTGTTG/	CATGAAAT	XM_289066	RIKEN cD 1700085A12 gene	1700085A12Rik		2.5	SM2072	e		4	1.656455229
mPool6_V2MM_33930	1.233143	59.99801	TRUE	HP_256764	TGCTGTTG/	CATGTCAT(	NM_011849	NIMA (never in mitosis gene a)-related expressed kinase 4	Nek4		2.7	SM2182	g		2	0.221863149
092308m1_V2MM_176986	1.233134	2.205692	TRUE	HP_397466	TGCTGTTG/	CATATATA(	AK081575	expressed sequence W91709	W91709		2.5	SM2083	b		1	1.656455229
092308m1_V2MM_176986	1.233134	2.205692	TRUE	HP_397466	TGCTGTTG/	CATATATA(	AK081575	expressed sequence W91709	W91709		2.5	SM2083	b		1	1.656455229
mPool7_V2MM_205147	1.233094	3.004939	TRUE	HP_423021	TGCTGTTG/	GAGGATGC	XM_127013	RIKEN cDNA 2600001A11 gene	2600001A11Rik		2.9	SM2300	d		4	1.522164293
mPool7_V2MM_79645	1.23278	3.004939	TRUE	HP_301300	TGCTGTTG/	GCCACAAC/	NM_021887	interleukin 21 receptor	Il21r		2.8	SM2238	a		6	1.522164293
mPool7_V2MM_201416	1.232656	3.004939	TRUE	HP_419445	TGCTGTTG/	CTGCCAAA(	XM_288575	Mus musculus LOC331967 (LOC331967), mRNA.			2.9	SM2288	a		5	1.522164293
mPool4_V2MM_159534	1.231956	66.95036	TRUE	HP_380127	TGCTGTTG/	CTGGAGCT	XM_283144	similar to regulator of sex-limitation candidate 24			2.16	SM2650	c		11	0.1742471
mPool4_V2MM_41824	1.231886	62.64426	TRUE	HP_264447	TGCTGTTG/	CAGCCAGC	NM_017400	SH3-domain GRB2-like 3	Sh3gl3		2.15	SM2564	b		12	0.203118686
mPool2_V2MM_3169	1.231545	3.004939	TRUE	HP_226775	TGCTGTTG/	CTTGTTATT	XM_486385	similar to RIKEN cDNA 2610042L04			2.11	SM2387	a		3	1.522164293
mPool7_V2MM_145089	1.231136	3.004939	TRUE	HP_365800	TGCTGTTG/	CTATTCTAA	XM_160546	Mus musculus LOC215301 (LOC215301), mR.			2.4	SM2049	a		4	1.522164293
mPool7_V2MM_145089	1.231136	3.004939	TRUE	HP_365800	TGCTGTTG/	CTATTCTAA	XM_160546	Mus musculus LOC215301 (LOC215301), mRNA.			2.4	SM2049	a		4	1.522164293
092308m1_V2MM_152312	1.231104	3.004939	TRUE	HP_372975	TGCTGTTG/	GTAAGTCA(	XM_195257	Mus musculus similar to Major uriry protein 5 precursor (MUP 5) (LOC272721), mR.			2.4	SM2031	e		5	1.522164293
mPool4_V2MM_47689	1.231064	75.83547	TRUE	HP_270162	TGCTGTTG/	CTTATTGG(	NM_023868	ryanodine receptor 2, cardiac	Ryr2		2.15	SM2568	h		7	0.120127608
092308m1_V2MM_176141	1.231009	3.004939	TRUE	HP_396623	TGCTGTTG/	CAGTGAAT/	XM_485788	hypothetical protein 9130221F21			2.5	SM2070	a		8	1.522164293
mPool7_V2MM_144281	1.230849	2.205692	TRUE	HP_364992	TGCTGTTG/	GCAAAGAA	XM_159840	Mus musculus LOC208254 (LOC208254), mRNA.			2.1	SM2332	c		10	1.656455229
mPool2_V2MM_189948	1.230282	3.004939	TRUE	HP_347774	TGCTGTTG/	GTCGCCTC	XM_355798	similar to carbohydrate (chondroitin 4) sulfotransferase 13; chondroitin 4-O-sulfotransf			2.11	SM2355	g		6	1.522164293
092308m1_V2MM_160694	1.23006	2.205692	TRUE	HP_381273	TGCTGTTG/	CTCCTTTAA	XM_283612	Mus musculus similar to pol polyprotein [Trichosurus vulpecula] (LOC329162), mR.			2.4	SM2045	f		2	1.656455229
mPool2_V2MM_123920	1.229957	3.004939	TRUE	HP_344775	TGCTGTTG/	CTCCAGGA	XM_143210	Mus musculus similar to bendless protein [Rattus norvegicus] (LOC241994), mRNA.			2.11	SM2359	a		3	1.522164293
092308m3_V2MM_225633	1.229808	59.99801	TRUE	HP_477676	TGCTGTTG/	CAGAGTTC(	NM_011030	procollagen-proline, 2-oxoglutarate 4-dioxygese (proline 4-hy	P4ha1		2.13	SM2454	h		10	0.221863149
092308m3_V2MM_100826	1.229612	75.83547	TRUE	HP_322003	TGCTGTTG/	CATAGTCA(	BC055027	expressed sequence AI132487	AI132487		2.1	SM2310	h		1	0.120127608
092308m3_V2MM_100826	1.229612	75.83547	TRUE	HP_322003	TGCTGTTG/	CATAGTCA(	BC055027	expressed sequence AI132487	AI132487		2.1	SM2310	h		1	0.120127608
mPool2_V2MM_136163	1.229573	3.004939	TRUE	HP_356883	TGCTGTTG/	CAGCTTAC/	XM_151461	Mus musculus LOC208337 (LOC208337), mRNA.			2.1	SM2336	d		2	1.522164293
mPool2_V2MM_127709	1.228634	2.205692	TRUE	HP_348531	TGCTGTTG/	CGAAAGGT	XM_145347	Mus musculus similar to Zinc finger protein 134 (LOC209850), mRNA.			2.11	SM2352	g		7	1.656455229
092308m3_V2MM_54981	1.228278	75.83547	TRUE	HP_277290	TGCTGTTG/	CCCACTGC(	NM_177027	RIKEN cD 4930572I07 gene	4930572I07Rik		2.12	SM2442	b		7	0.120127608
mPool2_V2MM_37833	1.227882	2.205692	TRUE	HP_260575	TGCTGTTG/	GAGGAAAT	NM_031374	testis expressed gene 15	Tex15		2.11	SM2392	g		1	1.656455229
mPool4_V2MM_101480	1.227184	75.83547	TRUE	HP_322651	TGCTGTTG/	CTGTGTGC(	XM_127657	RIKEN cDNA 2310032M22 gene			2.16	SM2606	f		12	0.120127608
092308m3_V2MM_90023	1.227109	75.83547	TRUE	HP_311449	TGCTGTTG/	GACCATTG/	AK030707	expressed sequence AI836003	AI836003		2.13	SM2470	c		10	0.120127608
mPool5_V2MM_11148	1.22701	61.29828	TRUE	HP_234565	TGCTGTTG/	CTTGATGA(	NM_007655	CD79A antigen (immunoglobulin-associated alpha)	Cd79a	NA				NA		0.212551731
092308m3_V2MM_145141	1.226871	75.83547	TRUE	HP_365852	TGCTGTTG/	CTACTCTG(	XM_160598	Mus musculus LOC242260 (LOC242260), mR.			2.14	SM2510	b		5	0.120127608
mPool2_V2MM_8808	1.226809	3.004939	TRUE	HP_232288	TGCTGTTG/	CAAAGTAT	NM_007646	CD38 antigen	Cd38		2.11	SM2375	h		2	1.522164293
mPool2_V2MM_120784	1.225891	3.004939	TRUE	HP_341676	TGCTGTTG/	CCCAGTAA	XM_485031	olfactory receptor 48	Olfr48		2.11	SM2359	b		1	1.522164293
092308m3_V2MM_84260	1.225693	75.83547	TRUE	HP_305776	TGCTGTTG/	CTTTCTATG	NM_027405	RIKEN cD 1700020O03 gene	1700020O03Rik		2.13	SM2473	h		2	0.120127608
092308m3_V2MM_232758	1.225394	35.99881	TRUE	HP_484691	TGCTGTTG/	CACCTTTA(	AK017151	RIKEN cD 5033404E19 gene	5033404E19Rik		2.13	SM2490	g		2	0.443711899
mPool7_V2MM_186924	1.22426	3.004939	TRUE	HP_407393	TGCTGTTG/	CTGCAAAT/	XM_289619	Mus musculus LOC333363 (LOC333363), mR.			2.5	SM2068	c		10	1.522164293
mPool7_V2MM_186924	1.22426	3.004939	TRUE	HP_407393	TGCTGTTG/	CTGCAAAT/	XM_289619	Mus musculus LOC333363 (LOC333363), mRNA.			2.5	SM2068	c		10	1.522164293
mPool7_V2MM_199863	1.224071	3.004939	TRUE	HP_417901	TGCTGTTG/	CTGTCATTT	AK048402	RIKEN cDNA C130057M05 gene	C130057M05Rik		2.9	SM2291	b		11	1.522164293
092308m1_V2MM_174282	1.223776	2.205692	TRUE	HP_394767	TGCTGTTG/	CACCAGGG	XM_287196	Mus musculus hypothetical gene supported by AK034924 (LOC329550), mR.			2.5	SM2084	c		1	1.656455229
092308m1_V2MM_174282	1.223776	2.205692	TRUE	HP_394767	TGCTGTTG/	CACCAGGG	XM_287196	Mus musculus hypothetical gene supported by AK034924 (LOC329550), mRNA.			2.5	SM2084	c		1	1.656455229
092308m1_V2MM_62732	1.22372	2.205692	TRUE	HP_284830	TGCTGTTG/	CATATTTCA	XM_143364	Mus musculus similar to pyridoxine kise [Plasmodium falciparum 3D7] (LOC213571), mF			2.3	SM2014	c		5	1.656455229
092308m1_V2MM_63001	1.223661	1.959154	TRUE	HP_285091	TGCTGTTG/	CCGAGGAC	NM_172920	RIKEN cD 1100001I19 gene	1100001I19Rik		2.3	SM2005	b		8	1.707931489
092308m1_V2MM_171804	1.222807	3.004939	TRUE	HP_392291	TGCTGTTG/	GCACAATC	XM_286696	Mus musculus hypothetical LOC328615 (LOC328615), mR.			2.5	SM2090	a		10	1.522164293
mPool4_V2MM_194251	1.222462	70.87591	TRUE	HP_412425	TGCTGTTG/	CTCACTTG/	XM_487805	similar to limitin			2.16	SM2607	b		4	0.149501346
mPool2_V2MM_10286	1.22242	3.004939	TRUE	HP_233724	TGCTGTTG/	CTCACCTA(	NM_053224	vomeronasal 1 receptor, A9	V1ra9		2.11	SM2381	d		7	1.522164293
mPool2_V2MM_165780	1.221891	3.004939	TRUE	HP_386314	TGCTGTTG/	GACTTAGC/	XM_285325	Mus musculus similar to protease [Mus musculus] (LOC329363), mRNA.			2.6	SM2127	c		2	1.522164293
092308m1_V2MM_175083	1.221261	3.004939	TRUE	HP_395568	TGCTGTTG/	GACTGAAC/	AK003729	RIKEN cD 1110015M06 gene	1110015M06Rik		2.5	SM2060	d		10	1.522164293
092308m3_V2MM_233770	1.220894	75.83547	TRUE	HP_485672	TGCTGTTG/	GTCCAGGG	XM_132152	RIKEN cD 3110007P09 gene	3110007P09Rik		2.14	SM2505	d		2	0.120127608
mPool7_V2MM_63636	1.220771	1.959154	TRUE	HP_285712	TGCTGTTG/	CTACTACA(	NM_146756	olfactory receptor 608	Olfr608		2.8	SM2240	e		1	1.707931489
092308m3_V2MM_101123	1.22061	66.95036	TRUE	HP_322298	TGCTGTTG/	CGCGGCAA	NM_001001321	solute carrier family 35, member D2	Slc35d2		2.13	SM2490	f		5	0.1742471

mPool2_V2MM_212105	1.220542	3.004939	TRUE	HP_429782	TGCTGTTG/	GGCATTGT/	XM_146652	Mus musculus similar to protease [Mus musculus] (LOC234930), mRNA.			2.11	SM2356	g		2	1.522164293
092308m3_V2MM_52597	1.219206	66.95036	TRUE	HP_274974	TGCTGTTG/	GAGTGAAG	NM_008966	prostaglandin F receptor	Ptgfr		2.12	SM2446	f		10	0.1742471
mPool7_V2MM_107197	1.217956	3.004939	TRUE	HP_328286	TGCTGTTG/	CAGCCAAG	AK017309	peroxisome biogenesis factor 1	Pex1		2.7	SM2154	g		3	1.522164293
mPool7_V2MM_178288	1.217727	3.004939	TRUE	HP_398762	TGCTGTTG/	CAGAATTG	XM_288127	Mus musculus hypothetical gene supported by AK053873 (LOC331495), mRNA.			2.9	SM2272	e		12	1.522164293
mPool4_V2MM_121736	1.217604	75.83547	TRUE	HP_342614	TGCTGTTG/	CATGTCTCT	XM_141818	Mus musculus similar to 60S RIBOSOMAL PROTEIN L18 (LOC236846), mRNA.			2.15	SM2551	g		5	0.120127608
mPool7_V2MM_78128	1.217157	3.004939	TRUE	HP_299827	TGCTGTTG/	GAATGAAG	NM_025418	RIKEN cDNA 1110059P08 gene	1110059P08Rik		2.8	SM2245	a		5	1.522164293
092308m3_V2MM_236535	1.217052	75.83547	TRUE	HP_488393	TGCTGTTG/	CTGACATT(	XM_157329	Mus musculus LOC225501 (LOC225501), mR.			2.14	SM2512	d		11	0.120127608
mPool5_V2MM_34126	1.216719	59.99801	TRUE	HP_256957	TGCTGTTG/	CCACTGCT(	NM_009321	tubulin cofactor a	Tbca	NA				NA		0.221863149
092308m3_V2MM_42114	1.216047	75.83547	TRUE	HP_264730	TGCTGTTG/	CAGTGTGT(	NM_009541	zinc finger and BTB domain containing 17	Zbtb17		2.12	SM2412	b		1	0.120127608
092308m1_V2MM_141173	1.215995	3.004939	TRUE	HP_361886	TGCTGTTG/	GGAAACAA	XM_156493	Mus musculus LOC239875 (LOC239875), mR.			2.4	SM2033	h		3	1.522164293
mPool7_V2MM_178556	1.21597	3.004939	TRUE	HP_399030	TGCTGTTG/	GTGTCATC/	XM_288181	Mus musculus LOC332445 (LOC332445), mRNA.			2.5	SM2065	e		4	1.522164293
mPool7_V2MM_178556	1.21597	3.004939	TRUE	HP_399030	TGCTGTTG/	GTGTCATC/	XM_288181	Mus musculus LOC332445 (LOC332445), mR.			2.5	SM2065	e		4	1.522164293
mPool4_V2MM_129839	1.215958	69.72441	TRUE	HP_350640	TGCTGTTG/	GAGCTCCT	XM_146477	gene model 1705, (NCBI)	Gm1705		2.14	SM2549	a		7	0.156615137
092308m3_V2MM_57096	1.215591	75.83547	TRUE	HP_279360	TGCTGTTG/	CTGTCTGG(	NM_153585	CCR4-NOT transcription complex, subunit 10	Cnot10		2.12	SM2416	f		11	0.120127608
092308m1_V2MM_86452	1.215223	3.004939	TRUE	HP_307928	TGCTGTTG/	CTCTTAAAT	NM_172468	RIKEN cDNA 4732481H14 gene	4732481H14Rik		2.6	SM2113	b		1	1.522164293
092308m1_V2MM_86452	1.215223	3.004939	TRUE	HP_307928	TGCTGTTG/	CTCTTAAAT	NM_172468	RIKEN cD 4732481H14 gene	4732481H14Rik		2.6	SM2113	b		1	1.522164293
092308m3_V2MM_115897	1.215145	70.87591	TRUE	HP_336855	TGCTGTTG/	GACCAAGA	XM_138679	Mus musculus similar to zinc finger protein 91 (HPF7, HTF10) [Homo sapiens] (LOC2183			2.1	SM2312	e		2	0.149501346
092308m3_V2MM_115897	1.215145	70.87591	TRUE	HP_336855	TGCTGTTG/	GACCAAGA	XM_138679	Mus musculus similar to zinc finger protein 91 (HPF7, HTF10) [Homo sapiens] (LOC2183			2.1	SM2312	e		2	0.149501346
mPool4_V2MM_214734	1.215122	75.83547	TRUE	HP_432309	TGCTGTTG/	CTGGCACT/	XM_145127	Mus musculus similar to set gene (LOC232637), mRNA.			2.16	SM2646	b		9	0.120127608
092308m3_V2MM_103162	1.215093	75.83547	TRUE	HP_324304	TGCTGTTG/	GTGCAAAG	XM_128854	RIKEN cD 2900055J20 gene	2900055J20Rik		2.14	SM2506	h		12	0.120127608
092308m1_V2MM_173900	1.214873	3.004939	TRUE	HP_394386	TGCTGTTG/	GTAGGAAG	XM_287114	Mus musculus hypothetical gene supported by AK052519 (LOC329402), mR.			2.5	SM2079	h		2	1.522164293
092308m1_V2MM_173900	1.214873	3.004939	TRUE	HP_394386	TGCTGTTG/	GTAGGAAG	XM_287114	Mus musculus hypothetical gene supported by AK052519 (LOC329402), mRNA.			2.5	SM2079	h		2	1.522164293
mPool2_V2MM_109640	1.214814	1.959154	TRUE	HP_330694	TGCTGTTG/	GGATTTAG(	XM_134026	RIKEN cDNA 2310051N18 gene	2310051N18Rik		2.7	SM2163	f		7	1.707931489
mPool2_V2MM_131800	1.213732	3.004939	TRUE	HP_352560	TGCTGTTG/	GGTTAAAG	XM_147845	Mus musculus LOC219184 (LOC219184), mRNA.			2.11	SM2367	h		2	1.522164293
mPool4_V2MM_151462	1.213468	75.83547	TRUE	HP_372133	TGCTGTTG/	GTCTCAAT/	XM_194774	Mus musculus similar to RIKEN cDNA 1110001A07 [Mus musculus] (LOC270833), mRNA			2.16	SM2650	b		3	0.120127608
mPool4_V2MM_186539	1.213016	61.29828	TRUE	HP_407010	TGCTGTTG/	GTCATTAT/	XM_289555	Mus musculus LOC333293 (LOC333293), mRNA.		NA				NA		0.212551731
092308m1_V2MM_92813	1.212434	2.205692	TRUE	HP_314155	TGCTGTTG/	CTGGCCTG/	NM_178780	RIKEN cD E130307J04 gene	E130307J04Rik		2.6	SM2108	d		6	1.656455229
mPool7_V2MM_63423	1.212083	2.205692	TRUE	HP_285502	TGCTGTTG/	CGGATAGA	BC043139	RIKEN cDNA 2700082D03 gene	2700082D03Rik		2.8	SM2244	c		8	1.656455229
mPool7_V2MM_140674	1.211769	3.004939	TRUE	HP_361392	TGCTGTTG/	CACTAGAA(	XM_156197	Mus musculus LOC208209 (LOC208209), mRNA.			2.5	SM2051	d		12	1.522164293
mPool7_V2MM_140674	1.211769	3.004939	TRUE	HP_361392	TGCTGTTG/	CACTAGAA(	XM_156197	Mus musculus LOC208209 (LOC208209), mR.			2.5	SM2051	d		12	1.522164293
mPool2_V2MM_210622	1.21095	3.004939	TRUE	HP_428365	TGCTGTTG/	GAAGTTAT(	AK015148	RIKEN cDNA 4930415H17 gene	4930415H17Rik		2.11	SM2356	b		12	1.522164293
mPool2_V2MM_21024	1.210728	3.004939	TRUE	HP_244205	TGCTGTTG/	CACTGATT(	NM_146145	Janus kinase 1	Jak1		2.11	SM2392	e		12	1.522164293
mPool2_V2MM_109775	1.209404	3.004939	TRUE	HP_330825	TGCTGTTG/	GATGTTATT	NM_027973	myeloid leukemia factor 1 interacting protein	Mlf1ip		2.7	SM2155	g		9	1.522164293
mPool4_V2MM_120134	1.209255	75.83547	TRUE	HP_106881	TGCTGTTG/	CAGTGTGA	XM_140772	gene model 340, (NCBI)	Gm340		2.14	SM2550	b		3	0.120127608
092308m1_V2MM_149037	1.208607	2.205692	TRUE	HP_369748	TGCTGTTG/	GAGACTTA(	XM_164431	Mus musculus LOC244521 (LOC244521), mR.			2.5	SM2058	c		8	1.656455229
mPool7_V2MM_83050	1.208544	3.004939	TRUE	HP_304605	TGCTGTTG/	GATTTGCT(	NM_011267	regulator of G-protein signaling 16	Rgs16		2.8	SM2250	h		7	1.522164293
mPool2_V2MM_132535	1.207848	2.205692	TRUE	HP_353286	TGCTGTTG/	GTCTTAAA(	AF032130	diabetic embryopathy 1	Dep1		2.11	SM2362	h		9	1.656455229
mPool5_V2MM_6244	1.205951	60.13437	TRUE	HP_229782	TGCTGTTG/	CTCTGAGT(	NM_007557	bone morphogenetic protein 7	Bmp7	NA				NA		0.220877236
mPool2_V2MM_30550	1.205938	3.004939	TRUE	HP_253474	TGCTGTTG/	CGGAGTAG	AK087545	heterogeneous nuclear ribonucleoprotein U-like 1	Hnrpul1		2.11	SM2390	g		11	1.522164293
mPool5_V2MM_158870	1.204813	61.29828	TRUE	HP_379475	TGCTGTTG/	GATTGTGT(	XM_282897	Mus musculus hypothetical gene supported by AK032987 (LOC327724), mRNA.		NA				NA		0.212551731
092308m1_V2MM_97001	1.204625	2.205692	TRUE	HP_318244	TGCTGTTG/	GCCGTAAT(	XM_111160	Mus musculus similar to eukaryotic translation initiation factor 4A1; initiation factor eIF			2.6	SM2109	a		11	1.656455229
mPool2_V2MM_212790	1.204301	3.004939	TRUE	HP_430448	TGCTGTTG/	GAAAGAGA	XM_149459	hypothetical LOC230628			2.11	SM2371	f		12	1.522164293
092308m1_V2MM_170774	1.203418	3.004939	TRUE	HP_391264	TGCTGTTG/	CACTGATC(	AK049051	engulfment and cell motility 1, ced-12 homolog (C. elegans)	Elmo1		2.5	SM2089	a		4	1.522164293
mPool2_V2MM_696	1.203314	2.205692	TRUE	HP_224359	TGCTGTTG/	CAGAGTTT/	NM_144552	syntaxin binding protein 6 (amisyn)	Stxbp6		2.11	SM2380	g		6	1.656455229
mPool4_V2MM_192385	1.203062	75.83547	TRUE	HP_411180	TGCTGTTG/	CCCGGAGC	NM_198959	hypocretin (orexin) receptor 1	Hcrtr1		2.16	SM2626	h		8	0.120127608
mPool2_V2MM_2758	1.2025	3.004939	TRUE	HP_226370	TGCTGTTG/	CTGGAGAT(	NM_172120	vacuolar protein sorting 41 (yeast)	Vps41		2.11	SM2385	b		3	1.522164293
mPool2_V2MM_104021	1.20204	3.004939	TRUE	HP_325158	TGCTGTTG/	CACTTTAAC	XM_129443	protein tyrosine phosphatase, receptor type, f polypeptide (P	Ppfia4		2.7	SM2159	f		4	1.522164293
mPool2_V2MM_94190	1.202007	3.004939	TRUE	HP_315484	TGCTGTTG/	GTATTTGT(	NM_183100	RIKEN cDNA 5430414B19 gene	5430414B19Rik		2.6	SM2146	h		6	1.522164293
092308m3_V2MM_54088	1.201759	60.13437	TRUE	HP_276425	TGCTGTTG/	CAGATGAA	XM_486633	similar to Xlr-related, meiosis regulated			2.12	SM2441	c		1	0.220877236
092308m1_V2MM_142075	1.200922	3.004939	TRUE	HP_362788	TGCTGTTG/	GTACAACT	XM_157299	Mus musculus LOC240266 (LOC240266), mRNA.			2.4	SM2046	a		9	1.522164293
092308m1_V2MM_142075	1.200922	3.004939	TRUE	HP_362788	TGCTGTTG/	GTACAACT	XM_157299	Mus musculus LOC240266 (LOC240266), mR.			2.4	SM2046	a		9	1.522164293
092308m1_V2MM_168480	1.200464	3.004939	TRUE	HP_388983	TGCTGTTG/	CCTATACCT	XM_286000	Mus musculus similar to zinc finger protein 93 homolog; zinc finger protein homologous			2.6	SM2114	h		7	1.522164293
092308m1_V2MM_88753	1.200049	3.004939	TRUE	HP_310189	TGCTGTTG/	CGGATAGA	AK028335	ubiquitin specific protease 28	Usp28		2.6	SM2112	g		9	1.522164293
092308m1_V2MM_88753	1.200049	3.004939	TRUE	HP_310189	TGCTGTTG/	CGGATAGA	AK028335	ubiquitin specific protease 28	Usp28		2.6	SM2112	g		9	1.522164293
092308m1_V2MM_86955	1.200042	3.004939	TRUE	HP_308420	TGCTGTTG/	CATTGCAA/	AK083909	RIKEN cD C030011O14 gene	C030011O14Rik		2.6	SM2112	f		11	1.522164293
mPool5_V2MM_199116	1.199548	75.83547	TRUE	HP_417168	TGCTGTTG/	CTGTGACT(	XM_289259	Mus musculus LOC332905 (LOC332905), mRNA.		NA				NA		0.120127608
mPool6_V2MM_154058	1.199513	35.99881	TRUE	HP_374700	TGCTGTTG/	CCTGTAAG(	BC056393	RIKEN cDNA 2600009P04 gene	2600009P04Rik		2.7	SM2191	h		6	0.443711899
mPool2_V2MM_89236	1.19948	2.205692	TRUE	HP_310666	TGCTGTTG/	GGATGAAG	BC056493	G protein-coupled receptor 123	Gpr123		2.6	SM2146	h		7	1.656455229
mPool6_V2MM_163145	1.19934	61.28368	TRUE	HP_383701	TGCTGTTG/	CAGACAAA	AK054302	expressed sequence C87414	C87414		2.8	SM2202	e		6	0.212655147
092308m3_V2MM_27305	1.198866	75.83547	TRUE	HP_250328	TGCTGTTG/	GAAACTAG	NM_028193	BRF1 homolog, subunit of R polymerase III transcription initia	Brf1		2.11	SM2399	h		1	0.120127608
mPool2_V2MM_107820	1.198858	2.205692	TRUE	HP_328901	TGCTGTTG/	CACTTACT(	AK006923	RIKEN cDNA 1700069L16 gene	1700069L16Rik		2.1	SM2323	a		6	1.656455229
mPool2_V2MM_155880	1.198419	3.004939	TRUE	HP_376519	TGCTGTTG/	GGCTCTCT(	XM_197869	Mus musculus LOC272173 (LOC272173), mRNA.			2.6	SM2129	b		5	1.522164293

mPool2_V2MM_106552	1.19833	2.205692	TRUE	HP_327652	TGCTGTTG/	GTCACAAA(	NM_199467	RIKEN cDNA F730047E07 gene	F730047E07Rik		2.7	SM2155	h		6	1.656455229
mPool4_V2MM_131911	1.198155	75.83547	TRUE	HP_352671	TGCTGTTG/	CTGTGTGG	XM_488731	hypothetical protein A530026G17			2.16	SM2637	d		9	0.120127608
mPool4_V2MM_37052	1.198027	69.72441	TRUE	HP_259823	TGCTGTTG/	GAATGCCT(	NM_144859	praja 2, RING-H2 motif containing	Pja2		2.15	SM2583	b		5	0.156615137
092308m1_V2MM_68315	1.19782	3.004939	TRUE	HP_290278	TGCTGTTG/	CTTGGAAT/	NM_030261	sestrin 3	Sesn3		2.4	SM2024	g		4	1.522164293
mPool4_V2MM_132131	1.197516	75.83547	TRUE	HP_352885	TGCTGTTG/	CCCTTTGG1	AK014907	RIKEN cDNA 4921517D16 gene	4921517D16Rik		2.14	SM2535	h		11	0.120127608
mPool4_V2MM_44096	1.197252	75.83547	TRUE	HP_266662	TGCTGTTG/	CTGTTCAC/	NM_147218	ATP-binding cassette, sub-family A (ABC1), member 6	Abca6		2.15	SM2599	a		12	0.120127608
092308m1_V2MM_144898	1.196292	2.205692	TRUE	HP_365609	TGCTGTTG/	CCAACCTTT	XM_160409	Mus musculus LOC242190 (LOC242190), mR.			2.5	SM2051	e		11	1.656455229
092308m3_V2MM_232072	1.196219	60.13437	TRUE	HP_484024	TGCTGTTG/	GCGCTTCT(	NM_022999	proline-rich Gla (G-carboxyglutamic acid) polypeptide 2	Prrg2		2.14	SM2504	d		1	0.220877236
mPool5_V2MM_12733	1.196184	75.83547	TRUE	HP_236118	TGCTGTTG/	CACCTTTG/	NM_010893	neuraminidase 1	Neu1	NA				NA		0.120127608
092308m1_V2MM_184101	1.195897	3.004939	TRUE	HP_404575	TGCTGTTG/	CAGGCTAT(	XM_289141	Mus musculus LOC332728 (LOC332728), mR.			2.5	SM2084	c		3	1.522164293
092308m1_V2MM_184101	1.195897	3.004939	TRUE	HP_404575	TGCTGTTG/	CAGGCTAT(	XM_289141	Mus musculus LOC332728 (LOC332728), mRNA.			2.5	SM2084	c		3	1.522164293
mPool6_V2MM_169845	1.195666	75.83547	TRUE	HP_390340	TGCTGTTG/	GAAGGGAA	XM_286346	Mus musculus hypothetical gene supported by AK076588 (LOC327906), mRNA.			2.7	SM2192	a		4	0.120127608
mPool7_V2MM_65931	1.195444	3.004939	TRUE	HP_287960	TGCTGTTG/	GTCCCACC(	NM_025629	thrombospondin, type I domain containing 6	Thsd6		2.8	SM2245	c		5	1.522164293
mPool6_V2MM_81411	1.195153	75.83547	TRUE	HP_303017	TGCTGTTG/	GTATTTGT(	NM_013663	splicing factor, arginine/serine-rich 3 (SRp20)	Sfrs3		2.8	SM2226	g		12	0.120127608
mPool6_V2MM_61849	1.1947	75.83547	TRUE	HP_283971	TGCTGTTG/	CAATTACA(	NM_025705	discoidin, CUB and LCCL domain containing 1	Dcbld1		2.7	SM2176	a		5	0.120127608
mPool7_V2MM_96426	1.194547	3.004939	TRUE	HP_317679	TGCTGTTG/	CACCAGGT(	L17069	myeloid/lymphoid or mixed-lineage leukemia	Mll		2.9	SM2260	d		1	1.522164293
092308m3_V2MM_87630	1.194491	75.83547	TRUE	HP_309080	TGCTGTTG/	CTGCTTGC(	NM_175208	RIKEN cD 4931431C16 gene	4931431C16Rik		2.13	SM2466	e		12	0.120127608
092308m3_V2MM_94116	1.194032	75.83547	TRUE	HP_315410	TGCTGTTG/	CCCATGGT	NM_183036	defensin beta 38	Defb38		2.9	SM2256	a		6	0.120127608
092308m3_V2MM_94116	1.194032	75.83547	TRUE	HP_315410	TGCTGTTG/	CCCATGGT	NM_183036	defensin beta 38	Defb38		2.9	SM2256	a		6	0.120127608
mPool2_V2MM_165085	1.193766	3.004939	TRUE	HP_385623	TGCTGTTG/	CCCAAAGA	XM_285164	Mus musculus similar to protease [Mus musculus] (LOC332160), mRNA.			2.6	SM2130	f		3	1.522164293
mPool4_V2MM_122191	1.19353	59.99801	TRUE	HP_343063	TGCTGTTG/	CTTCAGCCT	XM_484474	similar to High mobility group protein 1 (HMG-1) (Amphoterin) (Heparin-binding protei			2.14	SM2534	h		12	0.221863149
mPool2_V2MM_202256	1.193343	2.205692	TRUE	HP_420269	TGCTGTTG/	GAGCCTAT	AK033513	RIKEN cDNA 6030408C04 gene	6030408C04Rik		2.1	SM2323	b		12	1.656455229
mPool6_V2MM_2520	1.193163	66.95036	TRUE	HP_226135	TGCTGTTG/	CTGAAGAG	NM_007841	DEAD (Asp-Glu-Ala-Asp) box polypeptide 6	Ddx6		2.7	SM2170	f		2	0.1742471
mPool7_V2MM_102530	1.192928	3.004939	TRUE	HP_323679	TGCTGTTG/	GCTCCAAT(	AK036186	Wilms tumour 1-associating protein	Wtap		2.1	SM2310	e		2	1.522164293
mPool4_V2MM_45263	1.192751	75.83547	TRUE	HP_267802	TGCTGTTG/	GGTATTCT(	NM_177102	RIKEN cDNA A830041P22 gene	A830041P22Rik		2.16	SM2620	d		3	0.120127608
092308m3_V2MM_209703	1.192534	75.83547	TRUE	HP_427468	TGCTGTTG/	GACCAGGG	XM_156218	similar to plakoglobin			2.1	SM2327	f		9	0.120127608
092308m3_V2MM_209703	1.192534	75.83547	TRUE	HP_427468	TGCTGTTG/	GACCAGGG	XM_156218	similar to plakoglobin			2.1	SM2327	f		9	0.120127608
092308m3_V2MM_206814	1.19196	66.95036	TRUE	HP_424632	TGCTGTTG/	GAAGTCTA(	XM_158055	Mus musculus LOC227534 (LOC227534), mR.			2.14	SM2520	a		12	0.1742471
092308m3_V2MM_109571	1.191822	75.83547	TRUE	HP_330625	TGCTGTTG/	GCAAGCTA(	AY660739	RIKEN cDNA 4833424K13 gene	4833424K13Rik		2.1	SM2318	f		11	0.120127608
092308m3_V2MM_109571	1.191822	75.83547	TRUE	HP_330625	TGCTGTTG/	GCAAGCTA(	AY660739	RIKEN cD 4833424K13 gene	4833424K13Rik		2.1	SM2318	f		11	0.120127608
mPool5_V2MM_7534	1.191779	75.83547	TRUE	HP_231039	TGCTGTTG/	GCAATGTG(	NM_011697	vascular endothelial growth factor B	Vegfb	NA				NA		0.120127608
092308m3_V2MM_52863	1.191195	69.72441	TRUE	HP_275235	TGCTGTTG/	GCTGCAGA	NM_177078	adrenergic receptor kise, beta 2	Adrbk2		2.12	SM2439	f		7	0.156615137
092308m1_V2MM_73288	1.190996	2.205692	TRUE	HP_295125	TGCTGTTG/	GCCTTTGT(	AK080731	RIKEN cD A930024F17 gene	A930024F17Rik		2.3	SM2012	f		11	1.656455229
092308m1_V2MM_66531	1.19087	3.004939	TRUE	HP_288544	TGCTGTTG/	CAGCTTGT	NM_053070	carbonic anhydrase 7	Car7		2.4	SM2026	a		2	1.522164293
092308m1_V2MM_179498	1.190267	3.004939	TRUE	HP_399972	TGCTGTTG/	GCCAGAGT	XM_288344	Mus musculus LOC327806 (LOC327806), mR.			2.5	SM2087	c		7	1.522164293
092308m1_V2MM_155307	1.189461	3.004939	TRUE	HP_375946	TGCTGTTG/	GAACTAAC	XM_197341	Mus musculus LOC271503 (LOC271503), mR.			2.6	SM2123	b		12	1.522164293
092308m3_V2MM_61595	1.18944	75.83547	TRUE	HP_283726	TGCTGTTG/	GTTTATTG(	NM_010203	fibroblast growth factor 5	Fgf5		2.12	SM2411	d		10	0.120127608
mPool4_V2MM_242072	1.18903	75.83547	TRUE	HP_493822	TGCTGTTG/	CTGTGAAT(	XM_488025	similar to hypothetical protein FLJ38281			2.14	SM2545	f		11	0.120127608
mPool5_V2MM_5818	1.188487	75.83547	TRUE	HP_229366	TGCTGTTG/	CGAGGAAC	NM_175493	G protein-coupled receptor 68	Gpr68	NA				NA		0.120127608
mPool2_V2MM_9386	1.188204	3.004939	TRUE	HP_232847	TGCTGTTG/	CAGCTACA	NM_145141	expressed sequence BB219290	BB219290		2.11	SM2389	b		3	1.522164293
mPool6_V2MM_70998	1.18798	73.80895	TRUE	HP_292898	TGCTGTTG/	CAGGTCAG	NM_013848	erythroblast membrane-associated protein	Ermap		2.8	SM2226	h		9	0.131891001
092308m1_V2MM_177421	1.187738	2.205692	TRUE	HP_397901	TGCTGTTG/	CATTTACA/	XM_287871	Mus musculus hypothetical gene supported by AK086986 (LOC330881), mR.			2.5	SM2076	e		9	1.656455229
092308m1_V2MM_160259	1.187652	3.004939	TRUE	HP_380842	TGCTGTTG/	GTTTCCAA(	XM_283439	expressed sequence AW049306	AW049306		2.6	SM2121	c		8	1.522164293
092308m3_V2MM_44914	1.187644	59.99801	TRUE	HP_267461	TGCTGTTG/	GCTGTGAG	NM_010181	fibrillin 2	Fbn2		2.12	SM2446	a		1	0.221863149
mPool2_V2MM_53930	1.187433	3.004939	TRUE	HP_160904	TGCTGTTG/	CACTTAAG	NM_010167	eyes absent 4 homolog (Drosophila)	Eya4		2.7	SM2166	d		8	1.522164293
mPool2_V2MM_5320	1.18668	3.004939	TRUE	HP_228872	TGCTGTTG/	CCATTATT(	NM_025957	CEA-related cell adhesion molecule 14	Ceacam14		2.11	SM2386	a		4	1.522164293
092308m3_V2MM_44284	1.186638	75.83547	TRUE	HP_266849	TGCTGTTG/	CTTGTTGAT	NM_172618	BTB (POZ) domain containing 9	Btbd9		2.12	SM2418	e		2	0.120127608
092308m1_V2MM_129465	1.186589	3.004939	TRUE	HP_350271	TGCTGTTG/	GGTTGTAT(	XM_146310	Mus musculus LOC244446 (LOC244446), mR.			2.5	SM2099	e		4	1.522164293
mPool2_V2MM_162396	1.186203	2.205692	TRUE	HP_382957	TGCTGTTG/	CTTGCTTA/	XM_284334	Mus musculus similar to hypothetical protein FLJ13373 [Homo sapiens] (LOC330618), m			2.6	SM2131	b		11	1.656455229
092308m3_V2MM_85465	1.186023	75.83547	TRUE	HP_306946	TGCTGTTG/	GCCGCCAT/	NM_146389	olfactory receptor 1350	Olfr1350		2.13	SM2453	d		9	0.120127608
mPool2_V2MM_90015	1.185477	3.004939	TRUE	HP_311441	TGCTGTTG/	CTGTGAAC	NM_177715	potassium channel tetramerisation domain containing 12	Kctd12		2.6	SM2133	d		4	1.522164293
mPool4_V2MM_115429	1.18546	75.83547	TRUE	HP_336390	TGCTGTTG/	CTGCAAGT(	XM_487246	similar to Ig heavy chain precursor V region (VAR100) - mouse			2.16	SM2620	g		6	0.120127608
mPool4_V2MM_111822	1.185376	75.83547	TRUE	HP_332829	TGCTGTTG/	CTGGCAAG	XM_355234	kinesin family member 14	Kif14		2.16	SM2613	d		6	0.120127608
092308m1_V2MM_66945	1.185253	3.004939	TRUE	HP_288948	TGCTGTTG/	CCCACAGT(	NM_011059	peptidyl arginine deimise, type I	Padi1		2.3	SM2010	g		11	1.522164293
mPool2_V2MM_10496	1.184955	3.004939	TRUE	HP_233927	TGCTGTTG/	CTCTACGT/	NM_027275	RIKEN cDNA 2810422B04 gene	2810422B04Rik		2.11	SM2382	a		6	1.522164293
mPool7_V2MM_178353	1.184255	3.004939	TRUE	HP_398827	TGCTGTTG/	CCAAGTAC	XM_288143	Mus musculus hypothetical gene supported by AK046508 (LOC331558), mR.			2.5	SM2069	g		10	1.522164293
mPool7_V2MM_178353	1.184255	3.004939	TRUE	HP_398827	TGCTGTTG/	CCAAGTAC	XM_288143	Mus musculus hypothetical gene supported by AK046508 (LOC331558), mRNA.			2.5	SM2069	g		10	1.522164293
mPool7_V2MM_102051	1.184227	3.004939	TRUE	HP_323211	TGCTGTTG/	CCTTAAAT(	NM_026813	RIKEN cDNA 8430411H09 gene	8430411H09Rik		2.9	SM2299	d		7	1.522164293
092308m3_V2MM_38616	1.184218	75.83547	TRUE	HP_261336	TGCTGTTG/	GCCATAAA(	NM_144828	protein phosphatase 1, regulatory (inhibitor) subunit 1B	Ppp1r1b		2.11	SM2399	f		1	0.120127608
092308m3_V2MM_227514	1.184077	75.83547	TRUE	HP_479512	TGCTGTTG/	CTACAGCT/	XM_124482	ATP synthase, H+ transporting, mitochondrial F0 complex, sub	Atp5l		2.13	SM2456	d		12	0.120127608
mPool4_V2MM_158175	1.183038	60.13437	TRUE	HP_378785	TGCTGTTG/	CTTGGCAG(	XM_206288	Mus musculus LOC237944 (LOC237944), mRNA.		NA				NA		0.220877236
mPool2_V2MM_134397	1.182529	3.004939	TRUE	HP_355123	TGCTGTTG/	CTATAACA/	XM_149838	Mus musculus LOC232644 (LOC232644), mRNA.			2.11	SM2363	d		2	1.522164293

mPool2_V2MM_2496	1.182396	3.004939	TRUE	HP_226112	TGCTGTTG/	CTCTTGTAT	NM_018866	chemokine (C-X-C motif) ligand 13	Cxcl13		2.11	SM2386	h		12	1.522164293
mPool4_V2MM_87936	1.182141	59.99801	TRUE	HP_309383	TGCTGTTG/	GACTTGCT(	NM_175276	formin-family protein FHOS2			2.16	SM2624	e		10	0.221863149
092308m3_V2MM_56691	1.181326	75.83547	TRUE	HP_278966	TGCTGTTG/	CTGACTGT/	BC055876	RIKEN cD 1110007C02 gene	1110007C02Rik		2.12	SM2443	c		7	0.120127608
mPool2_V2MM_118660	1.181325	2.205692	TRUE	HP_339591	TGCTGTTG/	GTTCAAAG(	AK053237	myosin head domain containing 1	Myohd1		2.11	SM2368	e		5	1.656455229
092308m3_V2MM_139311	1.181177	60.13437	TRUE	HP_360031	TGCTGTTG/	CGACTTTG/	XM_154921	Mus musculus LOC219168 (LOC219168), mR.			2.14	SM2516	c		8	0.220877236
092308m3_V2MM_105808	1.180869	75.83547	TRUE	HP_326917	TGCTGTTG/	CTGATTGG	NM_008875	phospholipase D1	Pld1		2.13	SM2490	b		8	0.120127608
mPool7_V2MM_185502	1.180513	3.004939	TRUE	HP_405974	TGCTGTTG/	CCTTCTCAA	XM_289381	Mus musculus LOC333054 (LOC333054), mRNA.			2.5	SM2071	d		5	1.522164293
mPool7_V2MM_185502	1.180513	3.004939	TRUE	HP_405974	TGCTGTTG/	CCTTCTCAA	XM_289381	Mus musculus LOC333054 (LOC333054), mR.			2.5	SM2071	d		5	1.522164293
092308m1_V2MM_158758	1.18036	3.004939	TRUE	HP_379368	TGCTGTTG/	CACAAGAC	XM_207576	Mus musculus similar to envelope protein [Sus scrofa] (LOC280316), mR.			2.6	SM2117	d		5	1.522164293
mPool2_V2MM_164934	1.180347	2.205692	TRUE	HP_385472	TGCTGTTG/	CAAGTTGG	XM_285134	Mus musculus similar to pol protein [Sus scrofa] (LOC328552), mRNA.			2.6	SM2127	b		2	1.656455229
mPool6_V2MM_68559	1.179988	75.83547	TRUE	HP_290517	TGCTGTTG/	GACAGAAA	NM_025670	RIKEN cDNA 5730403B10 gene	5730403B10Rik		2.8	SM2215	d		12	0.120127608
mPool4_V2MM_189499	1.179796	75.83547	TRUE	HP_327305	TGCTGTTG/	GGCTCAGG	NM_194343	tripartite motif-containing 45	Trim45		2.16	SM2623	d		2	0.120127608
092308m1_V2MM_131052	1.179749	2.205692	TRUE	HP_351821	TGCTGTTG/	CTTCCTTAA	AK006428	RIKEN cD 1700027J07 gene	1700027J07Rik		2.5	SM2097	h		5	1.656455229
092308m1_V2MM_67801	1.179586	3.004939	TRUE	HP_289778	TGCTGTTG/	CTCCAAAG	NM_009803	nuclear receptor subfamily 1, group I, member 3	Nr1i3		2.3	SM2001	f		4	1.522164293
mPool4_V2MM_34025	1.179514	75.83547	TRUE	HP_256857	TGCTGTTG/	CCAAGAGA	NM_028228	PIN2/TRF1-interacting protein			2.15	SM2597	g		5	0.120127608
mPool6_V2MM_156589	1.177245	75.83547	TRUE	HP_377220	TGCTGTTG/	CTGTGCCT/	XM_203991	similar to SHPS-1			2.7	SM2194	b		4	0.120127608
092308m1_V2MM_133518	1.177072	3.004939	TRUE	HP_354257	TGCTGTTG/	CCTTCAATC	XM_149291	hypothetical gene supported by AK012899			2.5	SM2097	g		10	1.522164293
mPool2_V2MM_87228	1.176901	3.004939	TRUE	HP_308687	TGCTGTTG/	CGAAGAAT	NM_175111	Hspb associated protein 1	Hspbap1		2.6	SM2141	c		3	1.522164293
mPool2_V2MM_114347	1.175589	3.004939	TRUE	HP_335328	TGCTGTTG/	GCAGTATT/	XM_137453	Mus musculus similar to protease [Ovis aries] (LOC216550), mRNA.			2.1	SM2323	e		8	1.522164293
mPool7_V2MM_200840	1.17557	2.205692	TRUE	HP_418872	TGCTGTTG/	CTTTAGTCA	AK042686	hypothetical protein A730015I17			2.9	SM2283	h		9	1.656455229
mPool7_V2MM_178028	1.175099	2.205692	TRUE	HP_398505	TGCTGTTG/	GGTACAGC	XM_288012	similar to p47 protein isoform a			2.9	SM2284	c		5	1.656455229
mPool7_V2MM_65519	1.174923	3.004939	TRUE	HP_287555	TGCTGTTG/	GGACTAGA	NM_025769	RIKEN cDNA 5430404L10 gene	5430404L10Rik		2.8	SM2239	b		6	1.522164293
mPool4_V2MM_20096	1.174707	75.83547	TRUE	HP_243302	TGCTGTTG/	GTGCCAGA	NM_026449	UDP-N-acetyl-alpha-D-galactosamine:polypeptide N-acetylgal	Galnt15		2.15	SM2597	b		3	0.120127608
mPool2_V2MM_142227	1.174661	2.205692	TRUE	HP_362940	TGCTGTTG/	GTCATTACT	XM_157427	Mus musculus LOC240337 (LOC240337), mRNA.			2.1	SM2334	h		1	1.656455229
092308m1_V2MM_165913	1.173436	3.004939	TRUE	HP_386447	TGCTGTTG/	CAGATTTA1	NM_146592	Mus musculus similar to protease [Mus musculus] (LOC210103), mR.			2.6	SM2118	c		9	1.522164293
mPool7_V2MM_147294	1.173302	3.004939	TRUE	HP_368005	TGCTGTTG/	CAGTTAAG	XM_162853	Mus musculus LOC243690 (LOC243690), mRNA.			2.1	SM2329	e		3	1.522164293
mPool4_V2MM_44518	1.171515	61.29828	TRUE	HP_267074	TGCTGTTG/	GAGCTGAA	NM_010088	decidual/trophoblast prolactin-related protein	Dtprp		2.15	SM2575	c		9	0.212551731
092308m3_V2MM_37343	1.17137	60.13437	TRUE	HP_260103	TGCTGTTG/	GACGATGA	NM_007928	MAP/microtubule affinity-regulating kise 2	Mark2		2.11	SM2400	h		12	0.220877236
mPool6_V2MM_33614	1.17129	75.83547	TRUE	HP_256460	TGCTGTTG/	CAGCTTGT(	NM_007962	epithelial V-like antigen 1	Eva1		2.7	SM2170	a		7	0.120127608
mPool7_V2MM_62388	1.17114	3.004939	TRUE	HP_284497	TGCTGTTG/	CAGATTAT(	NM_007392	actin, alpha 2, smooth muscle, aorta	Acta2		2.8	SM2246	b		6	1.522164293
mPool2_V2MM_57065	1.170621	3.004939	TRUE	HP_279329	TGCTGTTG/	CTGTATCTC	NM_010107	ephrin A1	Efna1		2.7	SM2165	c		8	1.522164293
092308m3_V2MM_41743	1.170269	61.28368	TRUE	HP_264368	TGCTGTTG/	CAGAGGTT	NM_020565	sulfotransferase family 3A, member 1	Sult3a1		2.12	SM2417	h		6	0.212655147
mPool2_V2MM_120226	1.170055	3.004939	TRUE	HP_341127	TGCTGTTG/	CACAGAAT(	XM_140812	gene model 343, (NCBI)	Gm343		2.11	SM2368	h		2	1.522164293
mPool2_V2MM_120226	1.170055	3.004939	TRUE	HP_341127	TGCTGTTG/	CACAGAAT(	XM_140812	gene model 343, (NCBI)	Gm343		2.11	SM2368	h		2	1.522164293
mPool2_V2MM_114615	1.169766	3.004939	TRUE	HP_335592	TGCTGTTG/	GTGAGCTC	XM_137762	similar to PROHIBITIN (B-CELL RECEPTOR ASSOCIATED PROTEIN 32) (BAP 32)			2.7	SM2161	b		2	1.522164293
mPool2_V2MM_84461	1.169437	3.004939	TRUE	HP_305972	TGCTGTTG/	CCCTTAAG/	NM_028173	translocating chain-associating membrane protein 1	Tram1		2.6	SM2141	h		6	1.522164293
mPool2_V2MM_166076	1.169275	3.004939	TRUE	HP_386607	TGCTGTTG/	CAGCTAAA(	XM_285404	Mus musculus similar to ATP synthase, H+ transporting, mitochondrial F0 complex, subu			2.6	SM2130	h		5	1.522164293
mPool4_V2MM_241187	1.168214	60.13437	TRUE	HP_492950	TGCTGTTG/	CTCTAAAT(	XM_357569	gene model 1396, (NCBI)	Gm1396		2.14	SM2543	g		2	0.220877236
mPool7_V2MM_114177	1.167586	2.205692	TRUE	HP_335163	TGCTGTTG/	CTATCGAG(	XM_137307	Mus musculus similar to 60S RIBOSOMAL PROTEIN L29 (P23) (LOC216263), mRNA.			2.7	SM2158	h		1	1.656455229
mPool6_V2MM_72850	1.167445	75.83547	TRUE	HP_294699	TGCTGTTG/	GAAGCCTG	NM_010405	hemoglobin X, alpha-like embryonic chain in Hba complex	Hba-x		2.7	SM2180	e		5	0.120127608
092308m3_V2MM_117273	1.167386	75.83547	TRUE	HP_338219	TGCTGTTG/	GATGTGTG	XM_139295	caspase recruitment domain family, member 6	Card6		2.14	SM2507	e		7	0.120127608
092308m1_V2MM_187780	1.167259	2.205692	TRUE	HP_408244	TGCTGTTG/	CGAAATAT	XM_290140	Mus musculus LOC333674 (LOC333674), mR.			2.5	SM2076	h		11	1.656455229
mPool7_V2MM_70593	1.167152	3.004939	TRUE	HP_292501	TGCTGTTG/	CACCTTAA(	NM_025734	potassium voltage-gated channel, subfamily G, member 4	Kcng4		2.8	SM2229	g		8	1.522164293
mPool5_V2MM_178000	1.166189	75.83547	TRUE	HP_398477	TGCTGTTG/	GCCGGTGC	XM_287998	RIKEN cDNA 1700008B15 gene	1700008B15Rik	NA				NA		0.120127608
mPool6_V2MM_224067	1.166102	70.87591	TRUE	HP_476153	TGCTGTTG/	CAAAGGAG	XM_484949	hypothetical gene supported by AK040286			2.8	SM2212	c		8	0.149501346
mPool5_V2MM_7716	1.164838	66.95036	TRUE	HP_231214	TGCTGTTG/	GCCATTGT(	XM_136256	Mus musculus similar to protease (LOC240779), mRNA.		NA				NA		0.1742471
mPool4_V2MM_118347	1.16421	61.29828	TRUE	HP_339284	TGCTGTTG/	CACATGGA	XM_139800	similar to actin related protein 2/3 complex subunit 2; ARP2/3 protein complex subunit			2.16	SM2629	a		11	0.212551731
mPool2_V2MM_146021	1.164176	3.004939	TRUE	HP_366732	TGCTGTTG/	CTCTGAGA(	XM_161699	Mus musculus LOC242896 (LOC242896), mRNA.			2.1	SM2336	c		8	1.522164293
092308m3_V2MM_96297	1.163738	75.83547	TRUE	HP_254749	TGCTGTTG/	GACTGATC	XM_284619	similar to Cdv3 protein			2.12	SM2436	c		2	0.120127608
mPool5_V2MM_164644	1.163568	75.83547	TRUE	HP_385187	TGCTGTTG/	GACATATT/	XM_285057	Mus musculus similar to ubiquitin specific protease 10 [Homo sapiens] (LOC328338), mI		NA				NA		0.120127608
mPool2_V2MM_102905	1.163516	3.004939	TRUE	HP_324051	TGCTGTTG/	GACATAAT(	XM_128692	dishevelled associated activator of morphogenesis 2	Daam2		2.7	SM2157	f		6	1.522164293
mPool7_V2MM_173934	1.163251	3.004939	TRUE	HP_394420	TGCTGTTG/	CTATATCAA	XM_287120	Mus musculus hypothetical gene supported by AK034841 (LOC329386), mRNA.			2.5	SM2069	e		7	1.522164293
mPool7_V2MM_173934	1.163251	3.004939	TRUE	HP_394420	TGCTGTTG/	CTATATCAA	XM_287120	Mus musculus hypothetical gene supported by AK034841 (LOC329386), mR.			2.5	SM2069	e		7	1.522164293
mPool2_V2MM_148209	1.163156	2.205692	TRUE	HP_368920	TGCTGTTG/	GAAGTAGA	XM_163597	Mus musculus LOC210179 (LOC210179), mR.			2.4	SM2030	e		5	1.656455229
mPool2_V2MM_148209	1.163156	2.205692	TRUE	HP_368920	TGCTGTTG/	GAAGTAGA	XM_163597	Mus musculus LOC210179 (LOC210179), mRNA.			2.4	SM2030	e		5	1.656455229
mPool2_V2MM_20	1.162433	3.004939	TRUE	HP_223703	TGCTGTTG/	GTCTCCTA/	D29668	immunoglobulin kappa chain variable 8 (V8)	Igk-V8		2.11	SM2384	h		4	1.522164293
092308m3_V2MM_58849	1.161888	59.99801	TRUE	HP_281064	TGCTGTTG/	GCAACATG	NM_147014	olfactory receptor 1048	Olfr1048		2.12	SM2411	b		5	0.221863149
mPool4_V2MM_11016	1.161439	75.83547	TRUE	HP_234436	TGCTGTTG/	CTGTGTGA	NM_175284	frizzled homolog 10 (Drosophila)	Fzd10		2.16	SM2618	f		12	0.120127608
mPool6_V2MM_158511	1.161054	75.83547	TRUE	HP_379121	TGCTGTTG/	GCTTTCAA(	XM_206707	Mus musculus LOC279102 (LOC279102), mRNA.			2.7	SM2189	a		4	0.120127608
mPool2_V2MM_132331	1.160861	3.004939	TRUE	HP_353085	TGCTGTTG/	CCAGTTCT(	XM_358823	leucine rich repeat containing 15	Lrrc15		2.5	SM2099	f		9	1.522164293
mPool2_V2MM_132331	1.160861	3.004939	TRUE	HP_353085	TGCTGTTG/	CCAGTTCT(	XM_358823	leucine rich repeat containing 15	Lrrc15		2.5	SM2099	f		9	1.522164293
092308m3_V2MM_36537	1.160679	61.29828	TRUE	HP_259315	TGCTGTTG/	CTGTGATT	NM_011985	matrix metalloproteise 23	Mmp23		2.11	SM2396	c		5	0.212551731

mPool7_V2MM_74105	1.160661	3.004939	TRUE	HP_295919	TGCTGTTG/	CAAGAATT/	NM_009888	complement component factor h	Cfh		2.8	SM2232	a		11	1.522164293
mPool2_V2MM_141085	1.160474	2.205692	TRUE	HP_361798	TGCTGTTG/	CTATTGAG/	XM_156459	Mus musculus LOC239959 (LOC239959), mR.			2.4	SM2036	g		12	1.656455229
mPool2_V2MM_141085	1.160474	2.205692	TRUE	HP_361798	TGCTGTTG/	CTATTGAG/	XM_156459	Mus musculus LOC239959 (LOC239959), mRNA.			2.4	SM2036	g		12	1.656455229
092308m1_V2MM_145508	1.160388	3.004939	TRUE	HP_366219	TGCTGTTG/	CTTGGTCTT	XM_161006	Mus musculus LOC242463 (LOC242463), mR.			2.4	SM2043	c		12	1.522164293
092308m1_V2MM_145508	1.160388	3.004939	TRUE	HP_366219	TGCTGTTG/	CTTGGTCTT	XM_161006	Mus musculus LOC242463 (LOC242463), mRNA.			2.4	SM2043	c		12	1.522164293
092308m3_V2MM_151658	1.160368	35.99881	TRUE	HP_372328	TGCTGTTG/	CTCCTGTA(	XM_194896	Mus musculus similar to MRDS1 protein [Homo sapiens] (LOC268648), mR.			2.14	SM2513	g		7	0.443711899
mPool2_V2MM_11352	1.160003	3.004939	TRUE	HP_234765	TGCTGTTG/	GACTGGAT	NM_008217	hyaluronan synthase 3	Has3		2.7	SM2165	f		7	1.522164293
mPool2_V2MM_97978	1.159849	3.004939	TRUE	HP_319214	TGCTGTTG/	CCACTGTG/	XM_112001	Mus musculus LOC194863 (LOC194863), mRNA.			2.6	SM2133	b		10	1.522164293
092308m1_V2MM_95567	1.159462	3.004939	TRUE	HP_190094	TGCTGTTG/	GGGATAAT	NM_198033	expressed sequence AW060766	AW060766		2.6	SM2107	h		3	1.522164293
092308m3_V2MM_109745	1.159295	75.83547	TRUE	HP_330795	TGCTGTTG/	GAAACAAT(	AK019126	LSM6 homolog, U6 small nuclear R associated (S. cerevisiae)	Lsm6		2.1	SM2311	e		9	0.120127608
092308m3_V2MM_109745	1.159295	75.83547	TRUE	HP_330795	TGCTGTTG/	GAAACAAT(	AK019126	LSM6 homolog, U6 small nuclear RNA associated (S. cerevisiae	Lsm6		2.1	SM2311	e		9	0.120127608
mPool2_V2MM_82342	1.158811	3.004939	TRUE	HP_303920	TGCTGTTG/	GTCACCAT(	NM_008441	kinesin family member 1B	Kif1b		2.6	SM2144	h		4	1.522164293
092308m3_V2MM_106033	1.15869	66.95036	TRUE	HP_327139	TGCTGTTG/	GTGATACC(	XM_130987	retinoic acid receptor responder (tazarotene induced) 1	Rarres1		2.1	SM2315	a		5	0.1742471
092308m3_V2MM_106033	1.15869	66.95036	TRUE	HP_327139	TGCTGTTG/	GTGATACC(	XM_130987	retinoic acid receptor responder (tazarotene induced) 1	Rarres1		2.1	SM2315	a		5	0.1742471
mPool7_V2MM_146127	1.158583	3.004939	TRUE	HP_366838	TGCTGTTG/	CAGCTTATT	XM_161773	hypothetical gene supported by AK036869			2.4	SM2050	a		1	1.522164293
mPool7_V2MM_146127	1.158583	3.004939	TRUE	HP_366838	TGCTGTTG/	CAGCTTATT	XM_161773	hypothetical gene supported by AK036869			2.4	SM2050	a		1	1.522164293
092308m3_V2MM_24874	1.158542	75.83547	TRUE	HP_247960	TGCTGTTG/	GTCCCAGT	NM_178775	ribosomal protein S6 kise, 52kDa, polypeptide 1	Rps6kc1		2.12	SM2426	g		9	0.120127608
092308m1_V2MM_94697	1.158208	3.004939	TRUE	HP_315982	TGCTGTTG/	CTACTAGT/	NM_183214	RIKEN cD A830019P07 gene	A830019P07Rik		2.6	SM2109	f		7	1.522164293
mPool7_V2MM_108418	1.158064	3.004939	TRUE	HP_329488	TGCTGTTG/	GGAATCAT/	BC035209	PTPRF interacting protein, binding protein 1 (liprin beta 1)	Ppfibp1		2.7	SM2153	c		8	1.522164293
mPool5_V2MM_83212	1.157719	75.83547	TRUE	HP_304762	TGCTGTTG/	GCTTGAGA	NM_011591	translocator of inner mitochondrial membrane 17b	Timm17b	NA				NA		0.120127608
mPool7_V2MM_110094	1.157703	2.205692	TRUE	HP_331137	TGCTGTTG/	CTCAAGTA(	BC059844	RIKEN cDNA 4930566A11 gene	4930566A11Rik		2.1	SM2303	d		6	1.656455229
mPool6_V2MM_168312	1.156912	75.83547	TRUE	HP_388818	TGCTGTTG/	GGCATCCT(	XM_285956	Mus musculus similar to glyceraldehyde-3-phosphate dehydrogenase [Rattus norvegicu			2.8	SM2211	f		5	0.120127608
mPool2_V2MM_136965	1.155604	3.004939	TRUE	HP_357685	TGCTGTTG/	GTTAGTAC/	XM_488581	LOC432511			2.1	SM2334	d		12	1.522164293
092308m3_V2MM_206971	1.155346	75.83547	TRUE	HP_424787	TGCTGTTG/	GTAGCAGG	XM_162125	Mus musculus LOC243151 (LOC243151), mR.			2.1	SM2325	e		1	0.120127608
092308m3_V2MM_206971	1.155346	75.83547	TRUE	HP_424787	TGCTGTTG/	GTAGCAGG	XM_162125	Mus musculus LOC243151 (LOC243151), mRNA.			2.1	SM2325	e		1	0.120127608
092308m1_V2MM_144190	1.153888	2.205692	TRUE	HP_364901	TGCTGTTG/	CCATGGAA	XM_159798	Mus musculus LOC207822 (LOC207822), mR.			2.4	SM2045	h		7	1.656455229
092308m3_V2MM_33201	1.152973	75.83547	TRUE	HP_256058	TGCTGTTG/	CACCTTGTT	NM_133774	StAR-related lipid transfer (START) domain containing 4	Stard4		2.12	SM2435	f		1	0.120127608
mPool2_V2MM_124311	1.152429	2.205692	TRUE	HP_345163	TGCTGTTG/	CCTTTACCT	XM_143381	Mus musculus similar to 60S acidic ribosomal protein P1 (LOC229548), mRNA.			2.5	SM2097	h		1	1.656455229
mPool2_V2MM_124311	1.152429	2.205692	TRUE	HP_345163	TGCTGTTG/	CCTTTACCT	XM_143381	Mus musculus similar to 60S acidic ribosomal protein P1 (LOC229548), mR.			2.5	SM2097	h		1	1.656455229
mPool6_V2MM_79209	1.152404	75.83547	TRUE	HP_300877	TGCTGTTG/	GATGATAT(	NM_026197	RIKEN cDNA 2810013M15 gene	2810013M15Rik		2.8	SM2223	g		2	0.120127608
092308m3_V2MM_22487	1.152294	62.64426	TRUE	HP_245638	TGCTGTTG/	CGTGGTAT/	NM_178446	cD sequence BC013481	BC013481		2.11	SM2395	a		2	0.203118686
092308m3_V2MM_55747	1.152215	75.83547	TRUE	HP_278040	TGCTGTTG/	CGAACTAA(	NM_029292	RIKEN cD 1700008F21 gene	1700008F21Rik		2.12	SM2441	d		10	0.120127608
092308m1_V2MM_175542	1.151973	3.004939	TRUE	HP_396024	TGCTGTTG/	GCTTCTGA/	XM_287472	gene model 834, (NCBI)	Gm834		2.5	SM2093	f		12	1.522164293
092308m3_V2MM_126795	1.151875	60.13437	TRUE	HP_276695	TGCTGTTG/	CAGTGCCA(	XM_144814	gene model 459, (NCBI)	Gm459		2.12	SM2437	a		3	0.220877236
mPool4_V2MM_126431	1.151527	70.87591	TRUE	HP_347273	TGCTGTTG/	GTTGTTCA/	XM_144553	Mus musculus similar to 25 oligoadenylate synthetase-2 [Rattus norvegicus] (LOC23170			2.16	SM2638	a		7	0.149501346
mPool7_V2MM_105520	1.151276	3.004939	TRUE	HP_326632	TGCTGTTG/	GGCAGTTC	XM_130565	RIKEN cDNA 4732486I23 gene	4732486I23Rik		2.1	SM2313	e		4	1.522164293
mPool2_V2MM_96051	1.150916	3.004939	TRUE	HP_317315	TGCTGTTG/	GAATTGGA	XM_110002	similar to oogenesin			2.6	SM2139	a		2	1.522164293
mPool2_V2MM_109275	1.150877	3.004939	TRUE	HP_330337	TGCTGTTG/	CTCTCAGA/	XM_133717	RIKEN cDNA 4933427G17 gene	4933427G17Rik		2.1	SM2324	a		10	1.522164293
mPool2_V2MM_141475	1.150611	2.205692	TRUE	HP_362188	TGCTGTTG/	CCCTCTCTG	XM_156701	Mus musculus LOC213062 (LOC213062), mRNA.			2.1	SM2342	h		7	1.656455229
mPool7_V2MM_148663	1.149645	3.004939	TRUE	HP_369374	TGCTGTTG/	CTTTGATA/	XM_164008	Mus musculus LOC210862 (LOC210862), mR.			2.1	SM2328	e		1	1.522164293
mPool7_V2MM_148663	1.149645	3.004939	TRUE	HP_369374	TGCTGTTG/	CTTTGATA/	XM_164008	Mus musculus LOC210862 (LOC210862), mRNA.			2.1	SM2328	e		1	1.522164293
mPool5_V2MM_4059	1.149432	75.83547	TRUE	HP_227639	TGCTGTTG/	GGCTACCT(	NM_026582	RIKEN cDNA 5031439A09 gene	5031439A09Rik	NA				NA		0.120127608
mPool6_V2MM_70121	1.149132	75.83547	TRUE	HP_292039	TGCTGTTG/	GTGTGTGT/	NM_028726	RIKEN cDNA 4632404H12 gene	4632404H12Rik		2.7	SM2173	d		7	0.120127608
mPool7_V2MM_198418	1.14851	2.205692	TRUE	HP_416474	TGCTGTTG/	GAGTTCTT(	XM_287701	Mus musculus hypothetical gene supported by AK081791 (LOC330572), mRNA.			2.9	SM2271	c		12	1.656455229
mPool7_V2MM_143579	1.148032	3.004939	TRUE	HP_364291	TGCTGTTG/	CAGAGGAA	XM_158861	Mus musculus LOC241609 (LOC241609), mRNA.			2.1	SM2329	c		2	1.522164293
mPool2_V2MM_103648	1.147853	3.004939	TRUE	HP_324786	TGCTGTTG/	CTCAGAAG(	XM_129179	Mus musculus similar to Elongation factor 1-alpha 1 (EF-1-alpha-1) (Elongation factor 1 /			2.7	SM2151	e		10	1.522164293
mPool7_V2MM_115572	1.147766	3.004939	TRUE	HP_336533	TGCTGTTG/	CCTGTTGCT	XM_138503	Mus musculus similar to spermine synthase; spermidine aminopropyltransferase [Homo			2.7	SM2157	h		1	1.522164293
092308m1_V2MM_158501	1.147538	2.205692	TRUE	HP_379111	TGCTGTTG/	CTCACCACT	XM_206687	Mus musculus LOC279081 (LOC279081), mR.			2.6	SM2115	d		5	1.656455229
mPool7_V2MM_203726	1.147261	1.959154	TRUE	HP_409239	TGCTGTTG/	CAAGAAAT/	NM_199055	carbohydrate (N-acetylgalactosamine 4-0) sulfotransferase 9	Chst9		2.1	SM2301	d		2	1.707931489
mPool2_V2MM_96152	1.147171	3.004939	TRUE	HP_317412	TGCTGTTG/	CTCGAATC(	NM_010479	heat shock protein 1A	Hspa1a		2.6	SM2140	g		4	1.522164293
mPool4_V2MM_108910	1.146959	75.83547	TRUE	HP_329976	TGCTGTTG/	GCCTAGAC(	AK028022	RIKEN cDNA 1190020J12 gene	1190020J12Rik		2.16	SM2605	c		12	0.120127608
mPool4_V2MM_229820	1.146797	75.83547	TRUE	HP_481807	TGCTGTTG/	GGCTCTTG	XM_488696	hypothetical protein B930071L05		NA				NA		0.120127608
092308m3_V2MM_233858	1.146316	75.83547	TRUE	HP_485757	TGCTGTTG/	CATTTGACT	XM_137252	Mus musculus similar to nuclear transport factor 2 [Rattus norvegicus] (LOC237546), mF			2.14	SM2503	e		9	0.120127608
mPool4_V2MM_33162	1.146267	75.83547	TRUE	HP_256022	TGCTGTTG/	CACCCACA(	NM_080451	synaptopodin 2	Synpo2		2.16	SM2603	h		1	0.120127608
mPool7_V2MM_94142	1.146061	3.004939	TRUE	HP_315436	TGCTGTTG/	GTGCGTTG(	NM_183089	RIKEN cDNA 2600005O03 gene	2600005O03Rik		2.9	SM2256	a		11	1.522164293
mPool2_V2MM_97302	1.145999	3.004939	TRUE	HP_318544	TGCTGTTG/	CAATCAGA(	XM_111423	Mus musculus similar to protease [Mus musculus] (LOC195221), mRNA.			2.6	SM2143	c		9	1.522164293
092308m1_V2MM_118470	1.145543	3.004939	TRUE	HP_339405	TGCTGTTG/	CGAATGAC(	XM_139866	similar to cytochrome P450 CYP4F16			2.5	SM2097	b		11	1.522164293
092308m3_V2MM_231561	1.145432	75.83547	TRUE	HP_483524	TGCTGTTG/	CAGAGGCA	AK087368	myeloid/lymphoid or mixed-lineage leukemia 3	Mll3		2.14	SM2502	e		1	0.120127608
092308m1_V2MM_152581	1.145242	3.004939	TRUE	HP_373243	TGCTGTTG/	GTGGACAT(	XM_195448	Mus musculus similar to DJ (Hsp40) homolog, subfamily B, member 6 isoform a; Heat sh			2.4	SM2035	g		3	1.522164293
092308m1_V2MM_152581	1.145242	3.004939	TRUE	HP_373243	TGCTGTTG/	GTGGACAT(	XM_195448	Mus musculus similar to DnaJ (Hsp40) homolog, subfamily B, member 6 isoform a; Heat			2.4	SM2035	g		3	1.522164293
mPool7_V2MM_171834	1.144883	3.004939	TRUE	HP_392321	TGCTGTTG/	GGAAGAAC	XM_286706	hypothetical gene supported by AK040104			2.5	SM2090	e		2	1.522164293
mPool7_V2MM_171834	1.144883	3.004939	TRUE	HP_392321	TGCTGTTG/	GGAAGAAC	XM_286706	hypothetical gene supported by AK040104			2.5	SM2090	e		2	1.522164293

092308m1_V2MM_173927	1.144809	2.205692	TRUE	HP_394413	TGCTGTTG/	CAGAAATG	AK033904	hypothetical protein 9330112M16			2.5	SM2088	a		1	1.656455229
092308m1_V2MM_173927	1.144809	2.205692	TRUE	HP_394413	TGCTGTTG/	CAGAAATG	AK033904	hypothetical protein 9330112M16			2.5	SM2088	a		1	1.656455229
mPool4_V2MM_124074	1.14462	75.83547	TRUE	HP_344927	TGCTGTTG/	CAGATTTCT	XM_143290	Mus musculus similar to endothelial-monocyte activating polypeptide II (LOC229494), m			2.14	SM2543	b		9	0.120127608
092308m1_V2MM_67649	1.144481	3.004939	TRUE	HP_289630	TGCTGTTG/	CTAAGTTAC	AK051378	mitogen-activated protein kise kise kise 13 LocusID: 9175	Map3k13		2.4	SM2024	g		3	1.522164293
092308m3_V2MM_233523	1.14435	75.83547	TRUE	HP_485434	TGCTGTTG/	CCGTCAAT/	AK020441	RIKEN cD 9430027B09 gene	9430027B09Rik		2.13	SM2487	c		10	0.120127608
092308m3_V2MM_151297	1.143842	75.83547	TRUE	HP_371968	TGCTGTTG/	GTCTCTATC	XM_194682	similar to L-lactate dehydrogese A chain (LDH-A) (LDH muscle subunit) (LDH-M)			2.14	SM2523	g		1	0.120127608
mPool4_V2MM_181343	1.14296	62.64426	TRUE	HP_401817	TGCTGTTG/	GTCTCCGA/	XM_288660	Mus musculus LOC332070 (LOC332070), mRNA.		NA				NA		0.203118686
mPool7_V2MM_173900	1.142758	3.004939	TRUE	HP_394386	TGCTGTTG/	GTAGGAAG	XM_287114	Mus musculus hypothetical gene supported by AK052519 (LOC329402), mR.			2.5	SM2079	h		2	1.522164293
mPool7_V2MM_173900	1.142758	3.004939	TRUE	HP_394386	TGCTGTTG/	GTAGGAAG	XM_287114	Mus musculus hypothetical gene supported by AK052519 (LOC329402), mRNA.			2.5	SM2079	h		2	1.522164293
092308m3_V2MM_115106	1.142545	59.99801	TRUE	HP_336076	TGCTGTTG/	CTATTGCTC	XM_138114	Mus musculus similar to glyceraldehyde-3-phosphate dehydrogese (LOC217616), mR.			2.1	SM2307	b		9	0.221863149
092308m3_V2MM_115106	1.142545	59.99801	TRUE	HP_336076	TGCTGTTG/	CTATTGCTC	XM_138114	Mus musculus similar to glyceraldehyde-3-phosphate dehydrogenase (LOC217616), mR			2.1	SM2307	b		9	0.221863149
092308m1_V2MM_120199	1.142293	2.205692	TRUE	HP_341101	TGCTGTTG/	CGGTATAA(	XM_140800	dehydrogenase E1 and transketolase domain containing 1	Dhtkd1		2.11	SM2368	b		3	1.656455229
092308m1_V2MM_120199	1.142293	2.205692	TRUE	HP_341101	TGCTGTTG/	CGGTATAA(	XM_140800	dehydrogese E1 and transketolase domain containing 1	Dhtkd1		2.11	SM2368	b		3	1.656455229
092308m1_V2MM_174038	1.142277	3.004939	TRUE	HP_394524	TGCTGTTG/	CATAGTCTT	XM_287146	Mus musculus hypothetical gene supported by AK080575 (LOC329468), mR.			2.5	SM2069	e		8	1.522164293
mPool5_V2MM_12865	1.141786	35.99881	TRUE	HP_236248	TGCTGTTG/	CAGAAGCC	NM_007557	bone morphogenetic protein 7	Bmp7	NA				NA		0.443711899
mPool7_V2MM_177805	1.141473	2.205692	TRUE	HP_398285	TGCTGTTG/	CAGATAAT(	AK076537	RIKEN cDNA 4833445I07 gene	4833445I07Rik		2.5	SM2067	h		6	1.656455229
mPool7_V2MM_177805	1.141473	2.205692	TRUE	HP_398285	TGCTGTTG/	CAGATAAT(	AK076537	RIKEN cD 4833445I07 gene	4833445I07Rik		2.5	SM2067	h		6	1.656455229
092308m3_V2MM_52677	1.141256	75.83547	TRUE	HP_275053	TGCTGTTG/	GCAGCAGG	XM_126673	SMAD specific E3 ubiquitin protein ligase 2	Smurf2		2.12	SM2440	e		4	0.120127608
mPool2_V2MM_141569	1.141107	3.004939	TRUE	HP_362282	TGCTGTTG/	CCTAGGAA.	XM_156781	Mus musculus LOC210475 (LOC210475), mRNA.			2.1	SM2335	d		6	1.522164293
mPool7_V2MM_171571	1.140959	3.004939	TRUE	HP_392060	TGCTGTTG/	GACTCAAG(	AK030258	RIKEN cDNA 4930556M19 gene	4930556M19Rik		2.9	SM2295	d		2	1.522164293
092308m3_V2MM_106878	1.138996	75.83547	TRUE	HP_327971	TGCTGTTG/	GAGACCTA.	XM_131619	cD sequence BC022150	BC022150		2.14	SM2506	h		5	0.120127608
mPool7_V2MM_196450	1.138675	3.004939	TRUE	HP_414554	TGCTGTTG/	CGTTGTTT(	NM_172015	isoleucine-tRNA synthetase	Iars		2.9	SM2254	d		11	1.522164293
mPool7_V2MM_196450	1.138675	3.004939	TRUE	HP_414554	TGCTGTTG/	CGTTGTTT(	NM_172015	isoleucine-tR synthetase	Iars		2.9	SM2254	d		11	1.522164293
mPool4_V2MM_136264	1.13815	61.29828	TRUE	HP_356984	TGCTGTTG/	CAAATATG(	XM_151591	Mus musculus LOC213852 (LOC213852), mRNA.			2.14	SM2529	e		5	0.212551731
mPool7_V2MM_187047	1.138097	3.004939	TRUE	HP_407516	TGCTGTTG/	CAAAGAAC.	XM_289640	Mus musculus LOC333402 (LOC333402), mRNA.			2.5	SM2072	f		3	1.522164293
mPool7_V2MM_187047	1.138097	3.004939	TRUE	HP_407516	TGCTGTTG/	CAAAGAAC.	XM_289640	Mus musculus LOC333402 (LOC333402), mR.			2.5	SM2072	f		3	1.522164293
mPool4_V2MM_198581	1.137742	75.83547	TRUE	HP_416635	TGCTGTTG/	GGACTGCA	XM_287402	gene model 833, (NCBI)	Gm833	NA				NA		0.120127608
092308m3_V2MM_204989	1.137483	75.83547	TRUE	HP_422870	TGCTGTTG/	CCAATGGA.	XM_486261	glutaminyl-tR synthetase	Qars		2.13	SM2499	a		9	0.120127608
mPool2_V2MM_126885	1.137264	3.004939	TRUE	HP_347721	TGCTGTTG/	CTGAGGCT(	XM_144862	Mus musculus similar to Gag [Ovis aries] (LOC232180), mRNA.			2.11	SM2358	a		8	1.522164293
mPool2_V2MM_120199	1.137135	2.205692	TRUE	HP_341101	TGCTGTTG/	CGGTATAA(	XM_140800	dehydrogenase E1 and transketolase domain containing 1	Dhtkd1		2.11	SM2368	b		3	1.656455229
mPool2_V2MM_120199	1.137135	2.205692	TRUE	HP_341101	TGCTGTTG/	CGGTATAA(	XM_140800	dehydrogese E1 and transketolase domain containing 1	Dhtkd1		2.11	SM2368	b		3	1.656455229
092308m3_V2MM_112859	1.136834	66.95036	TRUE	HP_333851	TGCTGTTG/	CAATGGAT	XM_136697	cDNA sequence BC056494	BC056494		2.1	SM2321	g		2	0.1742471
092308m3_V2MM_112859	1.136834	66.95036	TRUE	HP_333851	TGCTGTTG/	CAATGGAT	XM_136697	cD sequence BC056494	BC056494		2.1	SM2321	g		2	0.1742471
mPool7_V2MM_112723	1.136815	3.004939	TRUE	HP_333715	TGCTGTTG/	CCGAGTAC.	XM_136631	Mus musculus similar to ribosomal protein L31 [Rattus norvegicus] (LOC227150), mRNA			2.1	SM2305	d		10	1.522164293
mPool7_V2MM_62723	1.13665	3.004939	TRUE	HP_284821	TGCTGTTG/	CATATATAC	NM_009988	coxsackievirus and adenovirus receptor	Cxadr		2.8	SM2240	h		7	1.522164293
mPool4_V2MM_122872	1.136649	75.83547	TRUE	HP_343730	TGCTGTTG/	GAGATAGT	XM_142472	similar to putative pheromone receptor (Go-VN4)			2.14	SM2531	c		12	0.120127608
mPool2_V2MM_121432	1.136327	3.004939	TRUE	HP_342313	TGCTGTTG/	GAGGTCAT(	XM_488332	similar to LRRG00119			2.11	SM2368	b		12	1.522164293
092308m1_V2MM_179440	1.135939	3.004939	TRUE	HP_399914	TGCTGTTG/	CATAATCA(	XM_288334	Mus musculus LOC331652 (LOC331652), mR.			2.5	SM2085	g		2	1.522164293
mPool2_V2MM_141176	1.135592	2.205692	TRUE	HP_361889	TGCTGTTG/	GAAACAAG	XM_156493	Mus musculus LOC239875 (LOC239875), mR.			2.4	SM2050	e		3	1.656455229
mPool2_V2MM_141176	1.135592	2.205692	TRUE	HP_361889	TGCTGTTG/	GAAACAAG	XM_156493	Mus musculus LOC239875 (LOC239875), mRNA.			2.4	SM2050	e		3	1.656455229
mPool2_V2MM_8880	1.135526	3.004939	TRUE	HP_232360	TGCTGTTG/	CAAGCGGA	NM_026480	RIKEN cDNA 2410146L05 gene	2410146L05Rik		2.11	SM2377	e		1	1.522164293
092308m1_V2MM_178954	1.135254	3.004939	TRUE	HP_399428	TGCTGTTG/	GGAAGCAT	XM_288248	Mus musculus LOC332522 (LOC332522), mRNA.			2.5	SM2081	b		1	1.522164293
092308m1_V2MM_178954	1.135254	3.004939	TRUE	HP_399428	TGCTGTTG/	GGAAGCAT	XM_288248	Mus musculus LOC332522 (LOC332522), mR.			2.5	SM2081	b		1	1.522164293
092308m3_V2MM_113039	1.135088	75.83547	TRUE	HP_334030	TGCTGTTG/	CTGGGAGC	XM_136780	Mus musculus similar to translation elongation factor eEF-1 alpha chain - rat (LOC22730			2.13	SM2490	f		2	0.120127608
mPool2_V2MM_158991	1.134922	2.205692	TRUE	HP_379595	TGCTGTTG/	GTAGTTTA/	XM_483938	RIKEN cDNA 1500026H17 gene	1500026H17Rik		2.6	SM2131	a		8	1.656455229
mPool5_V2MM_169518	1.13484	75.83547	TRUE	HP_390013	TGCTGTTG/	CTGTTTCTT	AK037372	RIKEN cDNA A130012E19 gene	A130012E19Rik	NA				NA		0.120127608
092308m3_V2MM_194363	1.134718	75.83547	TRUE	HP_412532	TGCTGTTG/	CGGTGTTT/	NM_178613	RIKEN cD 4933433P14 gene	4933433P14Rik		2.9	SM2268	g		2	0.120127608
092308m3_V2MM_194363	1.134718	75.83547	TRUE	HP_412532	TGCTGTTG/	CGGTGTTT/	NM_178613	RIKEN cDNA 4933433P14 gene	4933433P14Rik		2.9	SM2268	g		2	0.120127608
mPool6_V2MM_68187	1.134322	75.83547	TRUE	HP_290155	TGCTGTTG/	CTGTCCCTC	NM_009774	budding uninhibited by benzimidazoles 3 homolog (S. cerevisi	Bub3		2.8	SM2225	a		11	0.120127608
092308m1_V2MM_146447	1.134263	3.004939	TRUE	HP_367158	TGCTGTTG/	CGGATTAA.	XM_162010	Mus musculus LOC243064 (LOC243064), mR.			2.4	SM2031	a		4	1.522164293
mPool2_V2MM_144266	1.132677	3.004939	TRUE	HP_364977	TGCTGTTG/	GACAATGT(	XM_159833	Mus musculus LOC208237 (LOC208237), mRNA.			2.1	SM2335	h		8	1.522164293
092308m1_V2MM_178872	1.132057	3.004939	TRUE	HP_399346	TGCTGTTG/	CCCATCATC	XM_288234	Mus musculus LOC332510 (LOC332510), mR.			2.5	SM2092	e		4	1.522164293
092308m3_V2MM_113763	1.131893	75.83547	TRUE	HP_334750	TGCTGTTG/	CATGACAG.	XM_137098	Mus musculus similar to Nucleolar protein Nop56 (Nucleolar protein 5A) (LOC215992), r			2.13	SM2489	g		7	0.120127608
mPool4_V2MM_201687	1.131885	75.83547	TRUE	HP_419715	TGCTGTTG/	CAAAGCAT(	XM_288529	Mus musculus LOC331914 (LOC331914), mRNA.		NA				NA		0.120127608
092308m1_V2MM_160758	1.131563	3.004939	TRUE	HP_381337	TGCTGTTG/	CATCGTTAC	XM_283630	Mus musculus hypothetical gene supported by AK077805 (LOC329212), mR.			2.6	SM2123	b		7	1.522164293
mPool2_V2MM_165660	1.130546	3.004939	TRUE	HP_386194	TGCTGTTG/	CATGAACA'	XM_484793	similar to Peptidyl-prolyl cis-trans isomerase A (PPIase) (Rotamase) (Cyclophilin A) (Cycl			2.6	SM2130	a		6	1.522164293
mPool5_V2MM_3632	1.130373	75.83547	TRUE	HP_227225	TGCTGTTG/	GCATTGAC'	XM_356218	mannosidase 2, alpha B1	Man2b1	NA				NA		0.120127608
mPool2_V2MM_111512	1.130306	2.205692	TRUE	HP_332525	TGCTGTTG/	GGTAAACT(	XM_135893	coagulation factor IX	F9		2.7	SM2164	b		1	1.656455229
mPool2_V2MM_3115	1.130195	3.004939	TRUE	HP_226722	TGCTGTTG/	CTTGACAA/	XM_136256	Mus musculus similar to protease (LOC240779), mRNA.			2.11	SM2387	d		12	1.522164293
mPool2_V2MM_120783	1.129895	2.205692	TRUE	HP_341675	TGCTGTTG/	CAGTAATT/	XM_485031	olfactory receptor 48	Olfr48		2.11	SM2362	a		2	1.656455229
mPool4_V2MM_49706	1.12972	75.83547	TRUE	HP_272144	TGCTGTTG/	GTCTGCAT(	XM_357629	similar to immunoglobulin light chain variable region			2.16	SM2633	c		4	0.120127608
mPool2_V2MM_135742	1.129156	3.004939	TRUE	HP_356464	TGCTGTTG/	CAAACTAT/	XM_151017	Mus musculus LOC241032 (LOC241032), mR.			2.4	SM2034	h		3	1.522164293

mPool2_V2MM_135742	1.129156	3.004939	TRUE	HP_356464	TGCTGTTG	CAAACTAT	XM_151017	Mus musculus LOC241032 (LOC241032), mRNA.			2.4	SM2034	h		3	1.522164293
092308m3_V2MM_100181	1.128946	75.83547	TRUE	HP_321373	TGCTGTTG	GGCTAAGA	BC048675	RIKEN cDNA 1700012B07 gene	1700012B07Rik		2.1	SM2309	e		1	0.120127608
092308m3_V2MM_100181	1.128946	75.83547	TRUE	HP_321373	TGCTGTTG	GGCTAAGA	BC048675	RIKEN cD 1700012B07 gene	1700012B07Rik		2.1	SM2309	e		1	0.120127608
mPool5_V2MM_20464	1.12862	75.83547	TRUE	HP_243659	TGCTGTTG	GTTGATGG	NM_007831	deleted in colorectal carcinoma	Dcc	NA				NA		0.120127608
mPool7_V2MM_107239	1.127445	3.004939	TRUE	HP_328328	TGCTGTTG	GCACACTT	XM_131953	similar to CG9004-PA			2.1	SM2315	c		4	1.522164293
mPool5_V2MM_139958	1.127332	35.99881	TRUE	HP_360678	TGCTGTTG	CGCCTAGA	XM_155564	similar to CUB and Sushi multiple domains 3 isoform 1		NA				NA		0.443711899
mPool4_V2MM_41437	1.127305	75.83547	TRUE	HP_264073	TGCTGTTG	CACATGCT	NM_153601	glutamate-ammonia ligase (glutamine synthase) domain conta	Gluld1		2.16	SM2601	e		11	0.120127608
mPool4_V2MM_212650	1.127075	61.29828	TRUE	HP_430312	TGCTGTTG	GAGATATT	XM_140011	Mus musculus similar to probable cell division control protein p55CDC - rat (LOC224854			2.16	SM2648	b		10	0.212551731
mPool7_V2MM_70926	1.126741	3.004939	TRUE	HP_292827	TGCTGTTG	CAGCTTGT	NM_009875	cyclin-dependent kinase inhibitor 1B (P27)	Cdkn1b		2.8	SM2229	b		3	1.522164293
092308m3_V2MM_94440	1.126522	75.83547	TRUE	HP_315729	TGCTGTTG	CTCTTCAA	XM_487324	polymerase (R) III (D directed) polypeptide A	Polr3a		2.13	SM2453	e		12	0.120127608
mPool7_V2MM_201249	1.126381	3.004939	TRUE	HP_419279	TGCTGTTG	CATAGTTG	XM_289314	Mus musculus LOC332963 (LOC332963), mRNA.			2.9	SM2289	f		12	1.522164293
mPool2_V2MM_758	1.126109	3.004939	TRUE	HP_224419	TGCTGTTG	CAGCATTA	NM_026577	RIKEN cDNA C530009C10 gene	C530009C10Rik		2.11	SM2385	h		4	1.522164293
mPool5_V2MM_18015	1.126075	75.83547	TRUE	HP_241274	TGCTGTTG	GCCACAGT	NM_008289	hydroxysteroid 11-beta dehydrogenase 2	Hsd11b2	NA				NA		0.120127608
mPool2_V2MM_121742	1.125719	2.205692	TRUE	HP_342620	TGCTGTTG	CATGGGCT	XM_141826	Mus musculus similar to RIKEN cDNA 2610301B20; EST AI428449 [Mus musculus] (LOC2			2.11	SM2351	b		4	1.656455229
mPool7_V2MM_196145	1.125673	3.004939	TRUE	HP_414254	TGCTGTTG	GGCAAATT	NM_183219	hypothetical protein B230218O03			2.9	SM2268	h		6	1.522164293
mPool7_V2MM_196145	1.125673	3.004939	TRUE	HP_414254	TGCTGTTG	GGCAAATT	NM_183219	hypothetical protein B230218O03			2.9	SM2268	h		6	1.522164293
mPool2_V2MM_214369	1.125612	3.004939	TRUE	HP_431964	TGCTGTTG	GGAGGATC	XM_139471	Mus musculus similar to envelope protein [Ovis aries] (LOC239411), mRNA.			2.11	SM2358	e		7	1.522164293
092308m3_V2MM_54599	1.125167	75.83547	TRUE	HP_276922	TGCTGTTG	CATTTGGG	NM_025329	RIKEN cD 0610012D17 gene	0610012D17Rik		2.12	SM2407	e		3	0.120127608
mPool4_V2MM_132731	1.124736	66.95036	TRUE	HP_353481	TGCTGTTG	CGTGCACA	XM_148860	armadillo repeat containing 4	Armc4		2.14	SM2541	e		7	0.1742471
092308m3_V2MM_146128	1.124712	75.83547	TRUE	HP_366839	TGCTGTTG	CAGTCAAG	XM_161773	hypothetical gene supported by AK036869			2.14	SM2519	a		11	0.120127608
092308m3_V2MM_105815	1.124543	75.83547	TRUE	NA	NA	NA	NA	NA	NA	NA	NA	NA		NA		0.120127608
092308m3_V2MM_203704	1.124481	75.83547	TRUE	HP_421639	TGCTGTTG	CCTACAAA	XM_139220	similar to ribosomal protein S15; rat insulinoma gene			2.13	SM2498	b		5	0.120127608
092308m3_V2MM_143822	1.124345	59.99801	TRUE	HP_364534	TGCTGTTG	GAAATGGA	XM_159171	Mus musculus LOC245389 (LOC245389), mR.			2.14	SM2526	e		4	0.221863149
092308m3_V2MM_87599	1.124285	75.83547	TRUE	HP_309049	TGCTGTTG	CCTGTGTG	NM_175203	RIKEN cD 4833408A19 gene	4833408A19Rik		2.13	SM2453	g		2	0.120127608
092308m3_V2MM_35636	1.124259	75.83547	TRUE	HP_258428	TGCTGTTG	CTATGTGA	XM_111356	immunoglobulin heavy chain (V7183 family)	Igh-V7183		2.11	SM2397	h		10	0.120127608
092308m3_V2MM_226127	1.123799	75.83547	TRUE	HP_478159	TGCTGTTG	CGAGCATT	XM_483874	RIKEN cD 1700021A07 gene	1700021A07Rik		2.13	SM2453	g		5	0.120127608
092308m3_V2MM_25093	1.122007	60.13437	TRUE	HP_248172	TGCTGTTG	AGAGTCCA	AF190666	limb region 1	Lmbr1		2.12	SM2433	g		7	0.220877236
mPool2_V2MM_102558	1.121838	3.004939	TRUE	HP_323707	TGCTGTTG	GTGCATAT	NM_009558	zinc finger protein 51	Zfp51		2.7	SM2160	b		5	1.522164293
092308m1_V2MM_131538	1.121298	3.004939	TRUE	HP_352301	TGCTGTTG	CCACCTCTT	AK005401	RIKEN cD 1600002O04 gene	1600002O04Rik		2.6	SM2111	a		5	1.522164293
mPool4_V2MM_213633	1.121283	75.83547	TRUE	HP_431256	TGCTGTTG	GTCATCCT	NM_198673	cDNA sequence BC049697	BC049697		2.14	SM2543	a		8	0.120127608
092308m1_V2MM_159773	1.121147	3.004939	TRUE	HP_380366	TGCTGTTG	CAACCAAT	XM_283241	Mus musculus hypothetical gene supported by AK034333 (LOC328474), mR.			2.6	SM2117	h		9	1.522164293
mPool4_V2MM_25400	1.120282	75.83547	TRUE	HP_248468	TGCTGTTG	CAGAGGAA	NM_145601	cDNA sequence BC016201	BC016201		2.15	SM2589	b		6	0.120127608
092308m3_V2MM_38398	1.120219	59.99801	TRUE	HP_261125	TGCTGTTG	GCAGCCAT	AK044117	RIKEN cD C430014H23 gene	C430014H23Rik		2.12	SM2418	h		7	0.221863149
mPool2_V2MM_213506	1.12014	2.205692	TRUE	HP_431133	TGCTGTTG	GTCAAATT	XM_139486	Mus musculus LOC239417 (LOC239417), mRNA.			2.11	SM2357	b		2	1.656455229
mPool2_V2MM_84812	1.120085	3.004939	TRUE	HP_306312	TGCTGTTG	CAGTAAAG	NM_029498	zinc finger protein 198	Zfp198		2.6	SM2141	f		10	1.522164293
092308m3_V2MM_22500	1.119573	75.83547	TRUE	HP_245651	TGCTGTTG	CGTGTGTG	NM_009120	SAR1a gene homolog 1 (S. cerevisiae)	Sara1		2.11	SM2400	a		7	0.120127608
092308m3_V2MM_28986	1.119553	75.83547	TRUE	HP_142382	TGCTGTTG	GTGAAGCC	NM_146150	rdilysin, N-arginine dibasic convertase, NRD convertase 1	Nrd1		2.11	SM2398	d		1	0.120127608
mPool2_V2MM_86459	1.119209	3.004939	TRUE	HP_307935	TGCTGTTG	CTGTGTCT	NM_172474	RIKEN cDNA 5230400J09 gene	5230400J09Rik		2.6	SM2134	g		1	1.522164293
mPool4_V2MM_57320	1.118912	69.72441	TRUE	HP_279577	TGCTGTTG	CTTGAAGA	NM_172762	DNA segment, Chr 8, ERATO Doi 233, expressed	D8Ertd233e		2.15	SM2585	d		9	0.156615137
mPool2_V2MM_132235	1.118679	3.004939	TRUE	HP_352989	TGCTGTTG	CAGGAAAG	AK045005	Casitas B-lineage lymphoma b	Cblb		2.11	SM2351	a		3	1.522164293
mPool5_V2MM_5387	1.11808	75.83547	TRUE	HP_228938	TGCTGTTG	CCCAGGGA	NM_019466	Down syndrome critical region homolog 1 (human)	Dscr1	NA				NA		0.120127608
mPool6_V2MM_63892	1.118066	75.83547	TRUE	HP_285959	TGCTGTTG	CTCCCAGT	NM_030150	DNA segment, Chr 11, Lothar Hennighausen 2, expressed	D11Lgp2e		2.8	SM2219	h		6	0.120127608
mPool5_V2MM_7596	1.117929	73.80895	TRUE	HP_231100	TGCTGTTG	GCAGTTGA	NM_007674	caudal type homeo box 4	Cdx4	NA				NA		0.131891001
mPool7_V2MM_203147	1.117295	3.004939	TRUE	HP_421112	TGCTGTTG	CAGCTGAA	XM_130845	RIKEN cDNA E430012K20 gene	E430012K20Rik		2.9	SM2297	h		12	1.522164293
092308m3_V2MM_83794	1.117049	75.83547	TRUE	HP_305318	TGCTGTTG	CCAATTCT	NM_025499	RIKEN cD 1700027M21 gene	1700027M21Rik		2.13	SM2468	g		11	0.120127608
092308m1_V2MM_173519	1.116759	1.959154	TRUE	HP_394005	TGCTGTTG	CAAGAAAT	XM_287042	gene model 819, (NCBI)	Gm819		2.5	SM2082	e		10	1.707931489
mPool5_V2MM_166083	1.116696	75.83547	TRUE	HP_386614	TGCTGTTG	GCTGATGT	XM_487675	similar to neonatal submandibular gland protein B		NA				NA		0.120127608
mPool5_V2MM_13730	1.116324	59.99801	TRUE	NA	NA	NA	NA	NA	NA	NA	NA	NA		NA		0.221863149
mPool2_V2MM_28792	1.116099	2.205692	TRUE	HP_251771	TGCTGTTG	GTAAGAAA	NM_011762	zinc finger protein 59	Zfp59		2.11	SM2389	e		11	1.656455229
mPool5_V2MM_6492	1.116082	75.83547	TRUE	HP_230025	TGCTGTTG	CTGGCTTT	NM_011756	zinc finger protein 36	Zfp36	NA				NA		0.120127608
092308m3_V2MM_116394	1.115923	73.80895	TRUE	HP_337349	TGCTGTTG	CAAAGTTG	XM_138909	Mus musculus similar to endogenous retroviral family W, env(C7), member 1 (syncytin);			2.13	SM2487	c		3	0.131891001
mPool7_V2MM_149886	1.115651	3.004939	TRUE	HP_370596	TGCTGTTG	CAGGGTTT	XM_165303	Mus musculus LOC208460 (LOC208460), mR.			2.1	SM2331	a		8	1.522164293
mPool7_V2MM_149886	1.115651	3.004939	TRUE	HP_370596	TGCTGTTG	CAGGGTTT	XM_165303	Mus musculus LOC208460 (LOC208460), mRNA.			2.1	SM2331	a		8	1.522164293
mPool2_V2MM_86642	1.115368	2.205692	TRUE	HP_308113	TGCTGTTG	CATAGGAA	NM_173746	hypothetical protein C130086A10			2.6	SM2141	c		5	1.656455229
mPool7_V2MM_67874	1.11536	1.959154	TRUE	HP_289849	TGCTGTTG	CTCTAAGT	NM_030014	hook homolog 1 (Drosophila)	Hook1		2.8	SM2241	c		5	1.707931489
092308m1_V2MM_70780	1.115202	3.004939	TRUE	HP_292683	TGCTGTTG	CAGAGCAG	XM_195466	gene model 659, (NCBI)	Gm659		2.3	SM2004	g		8	1.522164293
mPool6_V2MM_64852	1.115182	75.83547	TRUE	HP_286900	TGCTGTTG	GAACTTTC	NM_007435	ATP-binding cassette, sub-family D (ALD), member 1	Abcd1		2.7	SM2181	c		1	0.120127608
092308m1_V2MM_134577	1.115112	3.004939	TRUE	HP_355302	TGCTGTTG	CCTCAAAT	AK017247	RIKEN cD 5430400N05 gene	5430400N05Rik		2.6	SM2113	e		11	1.522164293
mPool2_V2MM_120124	1.114877	3.004939	TRUE	HP_341031	TGCTGTTG	GAGAGAAA	XM_484819	cDNA sequence BC029127	BC029127		2.11	SM2367	f		10	1.522164293
mPool4_V2MM_179286	1.114359	75.83547	TRUE	HP_399760	TGCTGTTG	CTGTCTGT	XM_288305	Mus musculus LOC331613 (LOC331613), mRNA.		NA				NA		0.120127608
mPool7_V2MM_93767	1.113544	3.004939	TRUE	HP_315069	TGCTGTTG	CAGTTCTT	NM_181754	G protein-coupled receptor 141	Gpr141		2.9	SM2270	f		7	1.522164293
092308m3_V2MM_43600	1.113469	75.83547	TRUE	HP_266174	TGCTGTTG	CTCTTGTG	NM_016804	metaxin 2	Mtx2		2.12	SM2417	b		8	0.120127608

092308m3_V2MM_29298	1.113445	75.83547	TRUE	HP_252261	TGCTGTTG/	CAAAGCAAT	NM_146145	Janus kise 1	Jak1		2.12	SM2423	c		8	0.120127608
092308m1_V2MM_96559	1.113225	2.205692	TRUE	HP_317809	TGCTGTTG/	CACCTATAC	XM_110852	amyotrophic lateral sclerosis 2 (juvenile) chromosome region,	Als2cr19		2.6	SM2110	c		11	1.656455229
mPool2_V2MM_130410	1.113144	3.004939	TRUE	HP_351199	TGCTGTTG/	CATAACAG/	XM_146855	similar to BCL2/adenovirus E1B 19kDa-interacting protein 1, NIP2; BCL2/adenovirus E1B			2.11	SM2365	h		6	1.522164293
mPool2_V2MM_2146	1.113022	2.205692	TRUE	HP_225774	TGCTGTTG/	CTCACACAT	NM_010743	interleukin 1 receptor-like 1	Il1rl1		2.11	SM2387	f		10	1.656455229
mPool7_V2MM_197100	1.112647	2.205692	TRUE	HP_415184	TGCTGTTG/	CTCTTACCT	AK083918	spastic paraplegia 3A homolog (human)	Spg3a		2.9	SM2254	g		9	1.656455229
mPool7_V2MM_197100	1.112647	2.205692	TRUE	HP_415184	TGCTGTTG/	CTCTTACCT	AK083918	spastic paraplegia 3A homolog (human)	Spg3a		2.9	SM2254	g		9	1.656455229
092308m3_V2MM_35016	1.112546	59.99801	TRUE	HP_257822	TGCTGTTG/	CCTGTGAA/	NM_009322	T-box brain gene 1	Tbr1		2.11	SM2394	c		12	0.221863149
092308m3_V2MM_210322	1.112281	59.99801	TRUE	HP_428076	TGCTGTTG/	GAGAACTG	XM_162001	Mus musculus LOC243061 (LOC243061), mR.			2.14	SM2519	b		8	0.221863149
mPool2_V2MM_125363	1.11227	2.205692	TRUE	HP_346207	TGCTGTTG/	GACATTAG(	NM_001004178	hypothetical gene supported by BC064115			2.11	SM2361	a		11	1.656455229
092308m3_V2MM_47433	1.111944	61.29828	TRUE	HP_269915	TGCTGTTG/	CTGCATTG(	NM_145842	vomerosal 1 receptor, E9	V1re9		2.12	SM2445	h		12	0.212551731
mPool5_V2MM_8676	1.111489	75.83547	TRUE	HP_232157	TGCTGTTG/	GTTAAGCA(	NM_153091	suppression of tumorigenicity 7-like	St7l	NA				NA		0.120127608
mPool6_V2MM_164021	1.111095	75.83547	TRUE	HP_384571	TGCTGTTG/	CATCTTGG1	XM_284905	Mus musculus similar to pORF1 [Plasmodium yoelii yoelii] (LOC331722), mRNA.			2.7	SM2192	e		2	0.120127608
mPool7_V2MM_203319	1.109669	3.004939	TRUE	HP_309838	TGCTGTTG/	CTCTTCTCC	NM_175397	RIKEN cD 5830484A20 gene	5830484A20Rik		2.9	SM2269	b		7	1.522164293
mPool7_V2MM_203319	1.109669	3.004939	TRUE	HP_309838	TGCTGTTG/	CTCTTCTCC	NM_175397	RIKEN cDNA 5830484A20 gene	5830484A20Rik		2.9	SM2269	b		7	1.522164293
mPool6_V2MM_193872	1.109488	75.83547	TRUE	HP_379324	TGCTGTTG/	CTATGAAG(	XM_289920	filamin, alpha	Flna		2.8	SM2205	g		10	0.120127608
mPool6_V2MM_64404	1.109358	75.83547	TRUE	HP_286459	TGCTGTTG/	CTGGCCCT(	NM_009954	breast cancer anti-estrogen resistance 1	Bcar1		2.7	SM2178	b		5	0.120127608
mPool2_V2MM_113470	1.109327	1.959154	TRUE	HP_334457	TGCTGTTG/	CTGACATG/	XM_136985	Mus musculus similar to triosephosphate isomerase 1 (LOC215969), mRNA.			2.7	SM2157	b		11	1.707931489
092308m1_V2MM_156055	1.108968	3.004939	TRUE	HP_376694	TGCTGTTG/	GAAGATCC	XM_198073	Mus musculus LOC272426 (LOC272426), mR.			2.6	SM2122	f		3	1.522164293
mPool5_V2MM_154160	1.108963	75.83547	TRUE	HP_374801	TGCTGTTG/	CACAGTGT	XM_196624	Mus musculus LOC271637 (LOC271637), mRNA.		NA				NA		0.120127608
mPool4_V2MM_262148	1.108854	75.83547	TRUE	HP_522917	TGCTGTTG/	GCCTGCTT(	NM_138658	cDNA sequence AF085738	AF085738		2.15	SM2576	e		8	0.120127608
mPool2_V2MM_11459	1.108714	3.004939	TRUE	HP_234869	TGCTGTTG/	GAGTCAAT	NM_028018	RIKEN cDNA 2400003C14 gene	2400003C14Rik		2.11	SM2374	f		10	1.522164293
mPool2_V2MM_86999	1.108382	3.004939	TRUE	HP_106759	TGCTGTTG/	CAGATAAA	NM_174996	DNA segment, Chr 4, ERATO Doi 22, expressed	D4Ertd22e		2.6	SM2107	c		11	1.522164293
mPool2_V2MM_86999	1.108382	3.004939	TRUE	HP_106759	TGCTGTTG/	CAGATAAA	NM_174996	D segment, Chr 4, ERATO Doi 22, expressed	D4Ertd22e		2.6	SM2107	c		11	1.522164293
092308m1_V2MM_183421	1.108179	2.205692	TRUE	HP_403895	TGCTGTTG/	GAACCTAC	XM_289016	Mus musculus LOC332676 (LOC332676), mR.			2.5	SM2094	d		7	1.656455229
092308m1_V2MM_183443	1.107854	3.004939	TRUE	HP_403917	TGCTGTTG/	CACTGAGG	XM_289020	Mus musculus LOC332650 (LOC332650), mR.			2.5	SM2078	b		3	1.522164293
mPool2_V2MM_6003	1.107806	2.205692	TRUE	HP_229546	TGCTGTTG/	CGTCTTTG/	NM_008410	integral membrane protein 2B	Itm2b		2.11	SM2382	g		3	1.656455229
092308m1_V2MM_139106	1.107258	3.004939	TRUE	HP_359826	TGCTGTTG/	CATCTTGG(	XM_154777	Mus musculus LOC239075 (LOC239075), mRNA.			2.4	SM2043	c		6	1.522164293
092308m1_V2MM_139106	1.107258	3.004939	TRUE	HP_359826	TGCTGTTG/	CATCTTGG(	XM_154777	Mus musculus LOC239075 (LOC239075), mR.			2.4	SM2043	c		6	1.522164293
092308m3_V2MM_147852	1.107204	75.83547	TRUE	HP_368563	TGCTGTTG/	CCAAGAAA	XM_163317	Mus musculus LOC215331 (LOC215331), mR.			2.14	SM2517	c		10	0.120127608
092308m3_V2MM_41221	1.106744	75.83547	TRUE	HP_263865	TGCTGTTG/	CAAGATGT(	NM_016774	ATP synthase, H+ transporting mitochondrial F1 complex, bet/	Atp5b		2.12	SM2447	d		1	0.120127608
mPool2_V2MM_203365	1.106155	3.004939	TRUE	HP_421319	TGCTGTTG/	GAAATTTA(	XM_137252	Mus musculus similar to nuclear transport factor 2 [Rattus norvegicus] (LOC237546), mF			2.1	SM2318	b		12	1.522164293
mPool6_V2MM_160401	1.106057	75.83547	TRUE	HP_380981	TGCTGTTG/	CATCTAATC	NM_175506	a disintegrin-like and metalloprotease (reprolysin type) with t	Adamts19		2.8	SM2210	g		6	0.120127608
092308m3_V2MM_58035	1.105898	75.83547	TRUE	HP_30978	TGCTGTTG/	GACTTATT(	NM_016716	cullin 3	Cul3		2.12	SM2413	a		8	0.120127608
mPool2_V2MM_101136	1.105848	1.959154	TRUE	HP_322311	TGCTGTTG/	GCATTTATC	AK019536	RIKEN cDNA 4921504N20 gene	4921504N20Rik		2.7	SM2159	a		1	1.707931489
092308m3_V2MM_85318	1.105816	59.99801	TRUE	HP_306800	TGCTGTTG/	CTGGGAAC	NM_133979	expressed sequence AI604832	AI604832		2.13	SM2465	e		8	0.221863149
092308m1_V2MM_132170	1.105423	3.004939	TRUE	HP_352924	TGCTGTTG/	GGTAGCGT	XM_148178	RIKEN cD D930038M13 gene	D930038M13Rik		2.6	SM2106	a		9	1.522164293
mPool2_V2MM_126677	1.105078	3.004939	TRUE	HP_347518	TGCTGTTG/	CAAGTCAAT	XM_144734	Mus musculus similar to RNA helicase TNZ2 - mouse (LOC245881), mRNA.			2.11	SM2352	a		8	1.522164293
mPool7_V2MM_204465	1.104199	2.205692	TRUE	HP_422364	TGCTGTTG/	GCTGTTAA/	AK032192	potassium inwardly-rectifying channel, subfamily J, member 1	Kcnj16		2.9	SM2297	g		9	1.656455229
092308m1_V2MM_76998	1.103889	3.004939	TRUE	HP_298731	TGCTGTTG/	CTCTCTTAC	NM_146641	olfactory receptor 1164	Olfr1164		2.3	SM2011	c		4	1.522164293
mPool2_V2MM_206908	1.103539	2.205692	TRUE	HP_424725	TGCTGTTG/	GAAATGAT	XM_157374	Mus musculus LOC240302 (LOC240302), mRNA.			2.1	SM2341	a		2	1.656455229
mPool4_V2MM_43506	1.103405	75.83547	TRUE	HP_266084	TGCTGTTG/	CTCTCTTGG	NM_010178	FUS interacting protein (serine-arginine rich) 1	Fusip1		2.15	SM2570	g		6	0.120127608
092308m1_V2MM_185131	1.102773	3.004939	TRUE	HP_405603	TGCTGTTG/	CTACTTGAC	XM_289316	Mus musculus LOC332973 (LOC332973), mR.			2.5	SM2066	f		10	1.522164293
092308m1_V2MM_185131	1.102773	3.004939	TRUE	HP_405603	TGCTGTTG/	CTACTTGAC	XM_289316	Mus musculus LOC332973 (LOC332973), mRNA.			2.5	SM2066	f		10	1.522164293
mPool2_V2MM_213256	1.102731	3.004939	TRUE	HP_430896	TGCTGTTG/	GAAATACA(	BC066073	EST AA407452	AA407452		2.1	SM2350	c		6	1.522164293
mPool2_V2MM_114532	1.101883	3.004939	TRUE	HP_335509	TGCTGTTG/	CTGGAAAC	XM_137579	Mus musculus similar to olfactory receptor 30; olfactory receptor MOR281-1 [Mus musc			2.1	SM2318	g		10	1.522164293
mPool4_V2MM_28237	1.101222	75.83547	TRUE	HP_251230	TGCTGTTG/	GCCAGCAT(	NM_172262	amine oxidase, flavin containing 1	Aof1		2.15	SM2594	e		7	0.120127608
092308m3_V2MM_93505	1.100611	75.83547	TRUE	HP_314819	TGCTGTTG/	CACTTGAC(	NM_181412	zinc finger, BED domain containing 4	Zbed4		2.13	SM2455	e		10	0.120127608
mPool7_V2MM_89836	1.100438	2.205692	TRUE	HP_311264	TGCTGTTG/	GTGAATTA(	BC048817	RIKEN cDNA A730098D12 gene	A730098D12Rik		2.9	SM2270	d		5	1.656455229
092308m3_V2MM_35142	1.100327	62.64426	TRUE	HP_257943	TGCTGTTG/	CGACCTCC	NM_144902	solute carrier family 35 (UDP-N-acetylglucosamine (UDP-Glcc)	Slc35a3		2.11	SM2393	g		9	0.203118686
092308m3_V2MM_44493	1.099446	75.83547	TRUE	HP_28639	TGCTGTTG/	GAGATATT(	NM_008978	protein tyrosine phosphatase, non-receptor type 20	Ptpn20		2.12	SM2441	e		3	0.120127608
mPool4_V2MM_17740	1.099198	61.29828	TRUE	HP_241009	TGCTGTTG/	GCAACAGA	NM_145363	oculospanin			2.15	SM2587	g		7	0.212551731
092308m3_V2MM_23726	1.099118	75.83547	TRUE	HP_246850	TGCTGTTG/	CTTGTTGTT	NM_172272	cD sequence BC027073	BC027073		2.12	SM2428	f		9	0.120127608
092308m3_V2MM_58066	1.0986	75.83547	TRUE	HP_280306	TGCTGTTG/	GACTTTCT/	NM_025391	RIKEN cD 1110017C15 gene	1110017C15Rik		2.12	SM2414	a		9	0.120127608
092308m3_V2MM_103631	1.098363	75.83547	TRUE	NA	NA	NA	NA	NA	NA	NA		NA	NA	NA		0.120127608
092308m3_V2MM_107543	1.09827	75.83547	TRUE	HP_328626	TGCTGTTG/	CCGCAGAT(	XM_284166	RIKEN cD 5430427O21 gene	5430427O21Rik		2.14	SM2503	a		6	0.120127608
092308m1_V2MM_120808	1.098122	3.004939	TRUE	HP_341698	TGCTGTTG/	CTCAGAAA(	XM_141251	Mus musculus similar to cyclophilin A [Felis catus] (LOC241552), mR.			2.5	SM2098	b		9	1.522164293
092308m1_V2MM_120808	1.098122	3.004939	TRUE	HP_341698	TGCTGTTG/	CTCAGAAA(	XM_141251	Mus musculus similar to cyclophilin A [Felis catus] (LOC241552), mRNA.			2.5	SM2098	b		9	1.522164293
092308m1_V2MM_165344	1.0978	1.959154	TRUE	HP_385881	TGCTGTTG/	CGTCATTAC	XM_356940	similar to 60S ribosomal protein L9			2.6	SM2123	f		6	1.707931489
mPool4_V2MM_262197	1.097653	75.83547	TRUE	HP_521669	TGCTGTTG/	CTGATGCT(	NM_199033	expressed sequence AU067695	AU067695		2.16	SM2607	e		2	0.120127608
092308m1_V2MM_83706	1.097111	3.004939	TRUE	HP_305232	TGCTGTTG/	CAGGAAAT	NM_023900	pleckstrin homology domain containing, family J member 1	Plekhj1		2.6	SM2113	b		2	1.522164293
mPool2_V2MM_159987	1.097057	3.004939	TRUE	HP_380580	TGCTGTTG/	GTCCCAAA	NM_198629	hypothetical gene supported by AK045595			2.5	SM2131	f		3	1.522164293
mPool2_V2MM_137311	1.096773	3.004939	TRUE	HP_358031	TGCTGTTG/	CCAAGGAA	XM_153003	Mus musculus LOC237998 (LOC237998), mRNA.			2.1	SM2333	g		12	1.522164293

092308m1_V2MM_73791	1.096688	3.004939	TRUE	HP_295615	TGCTGTTG	GTCTCTGG	NM_009849	ectonucleoside triphosphate diphosphohydrolase 2	Entpd2		2.3	SM2003	g		9	1.522164293
092308m1_V2MM_151927	1.095985	2.205692	TRUE	HP_372594	TGCTGTTG	CTGTTTATC	XM_195000	expressed sequence AI413631	AI413631		2.4	SM2041	e		8	1.656455229
092308m3_V2MM_111750	1.095825	66.95036	TRUE	NA	NA	NA	NA	NA	NA	NA		NA	NA	NA		0.1742471
mPool6_V2MM_68198	1.095784	75.83547	TRUE	HP_290164	TGCTGTTG	CTGTCTTTG	NM_146930	olfactory receptor 791	Olfr791		2.8	SM2216	f		7	0.120127608
mPool2_V2MM_546	1.095609	3.004939	TRUE	HP_224215	TGCTGTTG	CACTGTGT	NM_007664	cadherin 2	Cdh2		2.7	SM2165	c		10	1.522164293
mPool2_V2MM_133000	1.095114	3.004939	TRUE	HP_353747	TGCTGTTG	GTTACATA	AK047658	RIKEN cDNA C130080N23 gene	C130080N23Rik		2.11	SM2361	g		3	1.522164293
mPool4_V2MM_43999	1.094979	75.83547	TRUE	HP_266566	TGCTGTTG	CTGTATTTC	XM_139056	gene model 290, (NCBI)	Gm290		2.16	SM2617	h		6	0.120127608
mPool2_V2MM_5165	1.094798	3.004939	TRUE	HP_228723	TGCTGTTG	CCAACCAA	NM_018768	syntaxin 8	Stx8		2.11	SM2385	f		6	1.522164293
mPool5_V2MM_8350	1.094178	59.99801	TRUE	HP_231837	TGCTGTTG	GTCAGTTA	NM_008223	serine (or cysteine) proteinase inhibitor, clade D, member 1	Serpind1	NA				NA		0.221863149
mPool2_V2MM_121889	1.093951	2.205692	TRUE	HP_342763	TGCTGTTG	CTCTGAAC	XM_141920	similar to hypothetical protein MGC37588			2.11	SM2363	c		5	1.656455229
mPool2_V2MM_155145	1.093726	2.205692	TRUE	HP_375786	TGCTGTTG	CCTGTTAG	XM_197224	Mus musculus LOC268804 (LOC268804), mRNA.			2.6	SM2131	h		12	1.656455229
092308m1_V2MM_175932	1.093374	3.004939	TRUE	HP_396414	TGCTGTTG	CAGGAATA	XM_287564	Mus musculus hypothetical gene supported by AK089795 (LOC330271), mRNA.			2.5	SM2072	b		10	1.522164293
092308m1_V2MM_175932	1.093374	3.004939	TRUE	HP_396414	TGCTGTTG	CAGGAATA	XM_287564	Mus musculus hypothetical gene supported by AK089795 (LOC330271), mR.			2.5	SM2072	b		10	1.522164293
092308m3_V2MM_47249	1.093243	60.13437	TRUE	HP_269734	TGCTGTTG	CTCGGAAT	NM_177177	RIKEN cD 5930403N24 gene	5930403N24Rik		2.12	SM2441	g		8	0.220877236
mPool4_V2MM_45829	1.092768	75.83547	TRUE	HP_268349	TGCTGTTG	CAGAAGTA	XM_489874	similar to TRAV9-1		NA				NA		0.120127608
092308m1_V2MM_148520	1.09275	2.205692	TRUE	HP_369231	TGCTGTTG	CTGTTTATG	XM_163870	Mus musculus LOC234017 (LOC234017), mR.			2.4	SM2035	f		9	1.656455229
092308m3_V2MM_113083	1.092457	75.83547	TRUE	HP_334073	TGCTGTTG	GCCATAAA	XM_136816	similar to 60S ribosomal protein L35			2.14	SM2503	f		5	0.120127608
mPool2_V2MM_154208	1.092442	3.004939	TRUE	HP_374849	TGCTGTTG	CTATGAAG	XM_196676	Mus musculus LOC271710 (LOC271710), mRNA.			2.6	SM2132	b		3	1.522164293
mPool7_V2MM_178954	1.092104	3.004939	TRUE	HP_399428	TGCTGTTG	GGAAGCAT	XM_288248	Mus musculus LOC332522 (LOC332522), mR.			2.5	SM2081	b		1	1.522164293
mPool7_V2MM_178954	1.092104	3.004939	TRUE	HP_399428	TGCTGTTG	GGAAGCAT	XM_288248	Mus musculus LOC332522 (LOC332522), mRNA.			2.5	SM2081	b		1	1.522164293
092308m3_V2MM_194272	1.091981	75.83547	TRUE	HP_412446	TGCTGTTG	CCAGGATG	NM_175350	RIKEN cD 4933402B14 gene	4933402B14Rik		2.13	SM2460	h		5	0.120127608
mPool2_V2MM_143000	1.09158	3.004939	TRUE	HP_363713	TGCTGTTG	CTGTTAAG	XM_158204	Mus musculus LOC241380 (LOC241380), mR.			2.4	SM2050	g		5	1.522164293
mPool2_V2MM_143000	1.09158	3.004939	TRUE	HP_363713	TGCTGTTG	CTGTTAAG	XM_158204	Mus musculus LOC241380 (LOC241380), mRNA.			2.4	SM2050	g		5	1.522164293
092308m1_V2MM_122300	1.091047	2.205692	TRUE	HP_343169	TGCTGTTG	GTAATAAA	XM_142110	gene model 381, (NCBI)	Gm381		2.6	SM2103	d		10	1.656455229
092308m1_V2MM_122300	1.091047	2.205692	TRUE	HP_343169	TGCTGTTG	GTAATAAA	XM_142110	gene model 381, (NCBI)	Gm381		2.6	SM2103	d		10	1.656455229
mPool7_V2MM_142721	1.090622	3.004939	TRUE	HP_363434	TGCTGTTG	CAGATTCA	XM_157862	Mus musculus LOC240631 (LOC240631), mR.			2.5	SM2051	h		12	1.522164293
mPool7_V2MM_142721	1.090622	3.004939	TRUE	HP_363434	TGCTGTTG	CAGATTCA	XM_157862	Mus musculus LOC240631 (LOC240631), mRNA.			2.5	SM2051	h		12	1.522164293
092308m3_V2MM_146518	1.090149	75.83547	TRUE	HP_367229	TGCTGTTG	GGCTGTGG	XM_162119	Mus musculus LOC231511 (LOC231511), mR.			2.14	SM2510	b		7	0.120127608
mPool4_V2MM_133211	1.090085	75.83547	TRUE	NA	NA	NA	NA	NA	NA	NA		NA	NA	NA		0.120127608
092308m1_V2MM_173837	1.08963	3.004939	TRUE	HP_394323	TGCTGTTG	CGTGTTCC	XM_287101	Mus musculus hypothetical gene supported by AK030988 (LOC329361), mR.			2.5	SM2084	e		3	1.522164293
mPool4_V2MM_128749	1.089256	59.99801	TRUE	HP_349562	TGCTGTTG	CTATGAGT	XM_145899	Mus musculus similar to putative pheromone receptor (Go-VN3) (LOC244115), mRNA.			2.14	SM2550	d		6	0.221863149
mPool2_V2MM_137685	1.08918	3.004939	TRUE	HP_358405	TGCTGTTG	CTCATATTG	XM_153460	Mus musculus LOC238186 (LOC238186), mRNA.			2.1	SM2334	d		10	1.522164293
092308m1_V2MM_93507	1.089168	3.004939	TRUE	HP_314821	TGCTGTTG	GTAAAGCT	NM_181413	ankyrin repeat and SAM domain containing 1	Anks1		2.6	SM2106	e		11	1.522164293
mPool4_V2MM_42825	1.089081	61.29828	TRUE	HP_265426	TGCTGTTG	CGCGGTTT	NM_028688	RIKEN cDNA 1700029M20 gene	1700029M20Rik		2.15	SM2583	c		8	0.212551731
092308m3_V2MM_98396	1.088204	75.83547	TRUE	HP_319624	TGCTGTTG	GATACTGG	XM_112451	Mus musculus LOC195654 (LOC195654), mR.			2.13	SM2477	g		11	0.120127608
mPool2_V2MM_147640	1.087987	3.004939	TRUE	HP_368351	TGCTGTTG	CTGTCTAA	XM_163135	Mus musculus LOC243740 (LOC243740), mRNA.			2.4	SM2046	b		10	1.522164293
mPool2_V2MM_147640	1.087987	3.004939	TRUE	HP_368351	TGCTGTTG	CTGTCTAA	XM_163135	Mus musculus LOC243740 (LOC243740), mR.			2.4	SM2046	b		10	1.522164293
mPool4_V2MM_177587	1.087748	75.83547	TRUE	HP_398067	TGCTGTTG	GCTGTGAT	XM_287909	Mus musculus LOC330964 (LOC330964), mRNA.		NA				NA		0.120127608
mPool2_V2MM_97303	1.087746	2.205692	TRUE	HP_386447	TGCTGTTG	CAGATTTA	XM_146592	Mus musculus similar to protease [Mus musculus] (LOC210103), mRNA.			2.6	SM2118	c		9	1.656455229
092308m3_V2MM_233631	1.087213	75.83547	TRUE	HP_485536	TGCTGTTG	CGAAAGGT	XM_136764	similar to RNP particle component			2.14	SM2509	e		3	0.120127608
mPool2_V2MM_142284	1.087074	3.004939	TRUE	HP_362997	TGCTGTTG	GCAAATGT	XM_157529	Mus musculus LOC240393 (LOC240393), mRNA.			2.1	SM2338	a		11	1.522164293
mPool6_V2MM_224135	1.086731	75.83547	TRUE	HP_476210	TGCTGTTG	CCCATACA	XM_195628	vomeronasal 1 receptor, D11	V1rd11		2.8	SM2213	f		3	0.120127608
092308m1_V2MM_125714	1.086598	3.004939	TRUE	HP_346557	TGCTGTTG	CATAGAGC	XM_144191	Mus musculus similar to Nedd4 binding protein 1 (LOC214795), mR.			2.5	SM2099	e		6	1.522164293
092308m1_V2MM_151143	1.086586	3.004939	TRUE	HP_371815	TGCTGTTG	GTGTGATT	XM_194518	Mus musculus similar to pol polyprotein [Trichosurus vulpecula] (LOC270309), mR.			2.4	SM2031	f		3	1.522164293
mPool2_V2MM_88780	1.086251	2.205692	TRUE	HP_310216	TGCTGTTG	GACCATAC	NM_175494	zinc finger protein 367	Zfp367		2.6	SM2143	f		1	1.656455229
mPool6_V2MM_155147	1.086239	75.83547	TRUE	HP_375788	TGCTGTTG	CTGTTAGC	XM_197224	Mus musculus LOC268804 (LOC268804), mRNA.			2.8	SM2212	b		5	0.120127608
092308m1_V2MM_91516	1.086147	3.004939	TRUE	HP_312888	TGCTGTTG	CAGGACAT	NM_178373	cell death-inducing DFFA-like effector c	Cidec		2.6	SM2109	d		1	1.522164293
092308m1_V2MM_68795	1.085744	2.205692	TRUE	HP_290741	TGCTGTTG	GAGATACA	NM_053142	protocadherin beta 17	Pcdhb17		2.3	SM2014	c		3	1.656455229
mPool2_V2MM_110894	1.085267	2.205692	TRUE	HP_331917	TGCTGTTG	CAGGGCAG	BC048918	RIKEN cDNA 4931406O17 gene	4931406O17Rik		2.7	SM2155	e		3	1.656455229
mPool2_V2MM_208740	1.085141	3.004939	TRUE	HP_426522	TGCTGTTG	GAGTCAAA	XM_157959	Mus musculus LOC213485 (LOC213485), mRNA.			2.1	SM2335	c		5	1.522164293
092308m3_V2MM_145760	1.085064	59.99801	TRUE	HP_366471	TGCTGTTG	CACTGTGG	XM_161356	similar to 60S ribosomal protein L29 (P23)			2.14	SM2523	c		7	0.221863149
mPool7_V2MM_68821	1.084678	3.004939	TRUE	HP_290767	TGCTGTTG	GAGATTTA	NM_026036	chemokine-like factor super family 6	Cklfsf6		2.8	SM2238	e		12	1.522164293
mPool2_V2MM_106722	1.084084	2.205692	TRUE	HP_327820	TGCTGTTG	CTGGTGAC	NM_024433	methylthioadenosine phosphorylase	Mtap		2.6	SM2149	c		5	1.656455229
mPool2_V2MM_9744	1.083877	3.004939	TRUE	HP_233195	TGCTGTTG	CATTAAAC	NM_008219	hemoglobin Z, beta-like embryonic chain	Hbb-bh1		2.11	SM2378	b		5	1.522164293
mPool5_V2MM_177410	1.083826	75.83547	TRUE	HP_397890	TGCTGTTG	GTTTCTTTG	XM_489247	hypothetical gene supported by AK080922		NA				NA		0.120127608
092308m3_V2MM_87004	1.08359	75.83547	TRUE	HP_308465	TGCTGTTG	CACGTTTA	NM_174997	cD sequence BC043098	BC043098		2.9	SM2266	g		11	0.120127608
092308m3_V2MM_87004	1.08359	75.83547	TRUE	HP_308465	TGCTGTTG	CACGTTTA	NM_174997	cDNA sequence BC043098	BC043098		2.9	SM2266	g		11	0.120127608
mPool4_V2MM_146577	1.083291	75.83547	TRUE	HP_367288	TGCTGTTG	CTGTGACT	XM_162158	Mus musculus LOC243180 (LOC243180), mRNA.			2.16	SM2644	f		6	0.120127608
mPool4_V2MM_73597	1.083253	75.83547	TRUE	HP_295428	TGCTGTTG	GGGATTAT	NM_176935	RIKEN cDNA F730015K02 gene	F730015K02Rik		2.16	SM2615	c		12	0.120127608
mPool4_V2MM_108049	1.082411	75.83547	TRUE	HP_329123	TGCTGTTG	CTGTGTAA	AK078040	cDNA sequence BC052360	BC052360		2.16	SM2624	b		5	0.120127608
mPool7_V2MM_90810	1.08233	2.205692	TRUE	HP_312226	TGCTGTTG	CATAATCTT	NM_177864	RIKEN cDNA A030013N09 gene	A030013N09Rik		2.9	SM2261	a		1	1.656455229
mPool7_V2MM_185500	1.082286	3.004939	TRUE	HP_405972	TGCTGTTG	CTTCTCAAT	XM_289381	Mus musculus LOC333054 (LOC333054), mRNA.			2.5	SM2095	c		3	1.522164293

mPool7_V2MM_185500	1.082286	3.004939	TRUE	HP_405972	TGCTGTTG/	CTTCTCAAT	XM_289381	Mus musculus LOC333054 (LOC333054), mR.			2.5	SM2095	c		3	1.522164293
092308m1_V2MM_146752	1.082242	2.205692	TRUE	HP_367463	TGCTGTTG/	GCACAATT(	XM_162261	Mus musculus LOC208184 (LOC208184), mR.			2.4	SM2046	f		6	1.656455229
092308m1_V2MM_146752	1.082242	2.205692	TRUE	HP_367463	TGCTGTTG/	GCACAATT(	XM_162261	Mus musculus LOC208184 (LOC208184), mRNA.			2.4	SM2046	f		6	1.656455229
mPool7_V2MM_87298	1.0819	3.004939	TRUE	HP_308756	TGCTGTTG/	CTCATACAT	NM_175126	zinc finger, CCHC domain containing 3	Zcchc3		2.9	SM2257	h		5	1.522164293
mPool7_V2MM_87298	1.0819	3.004939	TRUE	HP_308756	TGCTGTTG/	CTCATACAT	NM_175126	zinc finger, CCHC domain containing 3	Zcchc3		2.9	SM2257	h		5	1.522164293
mPool4_V2MM_106653	1.081656	75.83547	TRUE	HP_327752	TGCTGTTG/	GCACTTAT(	XM_131400	mitochondrial carrier triple repeat 1	Mcart1		2.16	SM2627	b		10	0.120127608
mPool2_V2MM_125934	1.081409	3.004939	TRUE	HP_346777	TGCTGTTG/	CTAAGCAT(	XM_144292	gene model 446, (NCBI)	Gm446		2.11	SM2360	h		8	1.522164293
mPool2_V2MM_128875	1.080771	3.004939	TRUE	HP_349688	TGCTGTTG/	GCAATAAT(	NM_206897	olfactory receptor 6	Olfr6		2.11	SM2353	f		9	1.522164293
mPool4_V2MM_176399	1.080719	75.83547	TRUE	HP_396881	TGCTGTTG/	GGAAGGAT	XM_287660	Mus musculus LOC330446 (LOC330446), mRNA.		NA				NA		0.120127608
mPool4_V2MM_38510	1.080539	75.83547	TRUE	HP_261235	TGCTGTTG/	GCCAACGT(	NM_031384	testis expressed gene 11	Tex11		2.16	SM2604	f		9	0.120127608
092308m1_V2MM_170515	1.080342	2.205692	TRUE	HP_391008	TGCTGTTG/	GCATATTA(	AK087847	RIKEN cDNA E330029E12 gene	E330029E12Rik		2.5	SM2068	f		4	1.656455229
092308m1_V2MM_170515	1.080342	2.205692	TRUE	HP_391008	TGCTGTTG/	GCATATTA(	AK087847	RIKEN cD E330029E12 gene	E330029E12Rik		2.5	SM2068	f		4	1.656455229
mPool7_V2MM_181518	1.079946	3.004939	TRUE	HP_401992	TGCTGTTG/	CTCTAAATT	XM_288689	Mus musculus LOC332089 (LOC332089), mRNA.			2.5	SM2087	c		9	1.522164293
mPool7_V2MM_181518	1.079946	3.004939	TRUE	HP_401992	TGCTGTTG/	CTCTAAATT	XM_288689	Mus musculus LOC332089 (LOC332089), mR.			2.5	SM2087	c		9	1.522164293
092308m3_V2MM_208446	1.079644	61.29828	TRUE	HP_426236	TGCTGTTG/	GCACGATT/	XM_153091	Mus musculus LOC238058 (LOC238058), mR.			2.14	SM2527	g		7	0.212551731
092308m1_V2MM_137884	1.079517	1.959154	TRUE	HP_358604	TGCTGTTG/	CTCTGGTTA	XM_153684	Mus musculus LOC212115 (LOC212115), mR.			2.4	SM2035	g		2	1.707931489
092308m3_V2MM_89934	1.079505	75.83547	TRUE	HP_311361	TGCTGTTG/	CCTTATAAC	NM_177696	hypothetical protein 4921513O04			2.13	SM2451	d		7	0.120127608
mPool4_V2MM_51895	1.079182	60.13437	TRUE	HP_274283	TGCTGTTG/	CTGCTGCT	NM_020521	vomeronasal 1 receptor, B5	V1rb5		2.15	SM2568	b		7	0.220877236
mPool2_V2MM_85234	1.079054	1.959154	TRUE	HP_306719	TGCTGTTG/	GATACATG/	NM_133740	heterogeneous nuclear ribonucleoprotein methyltransferase-	Hrmt1l3		2.6	SM2136	d		5	1.707931489
mPool4_V2MM_211052	1.078541	75.83547	TRUE	HP_428781	TGCTGTTG/	CAGAGTCT(	NM_198624	RIKEN cDNA 4922504M18 gene	4922504M18Rik		2.11	SM2372	a		1	0.120127608
092308m1_V2MM_131977	1.078112	3.004939	TRUE	HP_352737	TGCTGTTG/	CAAAGGAT	XM_147934	ATP-binding cassette, sub-family D (ALD), member 2	Abcd2		2.5	SM2099	c		12	1.522164293
mPool2_V2MM_96911	1.078062	3.004939	TRUE	HP_318154	TGCTGTTG/	GTCTCAAT/	XM_111099	Mus musculus LOC195045 (LOC195045), mRNA.			2.6	SM2113	e		10	1.522164293
mPool2_V2MM_96911	1.078062	3.004939	TRUE	HP_318154	TGCTGTTG/	GTCTCAAT/	XM_111099	Mus musculus LOC195045 (LOC195045), mR.			2.6	SM2113	e		10	1.522164293
mPool6_V2MM_67650	1.077974	75.83547	TRUE	HP_289631	TGCTGTTG/	CTAATAATT	AK080731	RIKEN cDNA A930024F17 gene	A930024F17Rik		2.8	SM2224	f		6	0.120127608
mPool7_V2MM_200136	1.077826	2.205692	TRUE	HP_418173	TGCTGTTG/	CTTGGAAT(	XM_287596	Mus musculus hypothetical gene supported by AK083439 (LOC330337), mRNA.			2.9	SM2291	c		6	1.656455229
092308m1_V2MM_118377	1.077701	2.205692	TRUE	HP_339314	TGCTGTTG/	CCTTATATG	XM_484607	similar to zinc finger protein 40			2.5	SM2100	c		4	1.656455229
mPool4_V2MM_42233	1.077665	75.83547	TRUE	HP_264843	TGCTGTTG/	CATGATGA	NM_138590	zinc finger, CCHC domain containing 7	Zcchc7		2.15	SM2576	c		7	0.120127608
mPool7_V2MM_193635	1.077653	2.205692	TRUE	HP_284612	TGCTGTTG/	CAGGACAA	NM_001004357	contactin associated protein-like 2	Cntnap2		2.8	SM2247	f		5	1.656455229
mPool2_V2MM_116764	1.077433	3.004939	TRUE	HP_337715	TGCTGTTG/	GTGCCCTG(	XM_139080	Mus musculus similar to 40S ribosomal protein S17 (LOC219110), mRNA.			2.7	SM2153	f		2	1.522164293
mPool2_V2MM_84902	1.076825	3.004939	TRUE	HP_306402	TGCTGTTG/	CCAGCTTG/	NM_029792	beta-1,3-glucuronyltransferase 1 (glucuronosyltransferase P)	B3gat1		2.6	SM2133	a		5	1.522164293
mPool2_V2MM_211035	1.076824	3.004939	TRUE	HP_428766	TGCTGTTG/	CCAGGAGT	AK012792	RIKEN cDNA 1700037C18 gene	1700037C18Rik		2.11	SM2369	d		8	1.522164293
092308m3_V2MM_146523	1.076642	61.29828	TRUE	HP_367234	TGCTGTTG/	CAGCAGGA	XM_162120	Mus musculus LOC231512 (LOC231512), mR.			2.14	SM2535	h		2	0.212551731
mPool2_V2MM_147347	1.075924	3.004939	TRUE	HP_368058	TGCTGTTG/	GCTATTATT	XM_162888	Mus musculus LOC208753 (LOC208753), mRNA.			2.1	SM2334	f		6	1.522164293
mPool6_V2MM_165199	1.075698	75.83547	TRUE	HP_385736	TGCTGTTG/	GCAATGAG	XM_285193	Mus musculus similar to HDCMA18P protein; DKFZP564K112 protein [Homo sapiens] (L(			2.7	SM2199	c		10	0.120127608
092308m3_V2MM_136883	1.075253	75.83547	TRUE	HP_357603	TGCTGTTG/	GCAACAAA	XM_152202	Mus musculus LOC237477 (LOC237477), mRNA.			2.1	SM2325	g		10	0.120127608
092308m3_V2MM_136883	1.075253	75.83547	TRUE	HP_357603	TGCTGTTG/	GCAACAAA	XM_152202	Mus musculus LOC237477 (LOC237477), mR.			2.1	SM2325	g		10	0.120127608
mPool4_V2MM_203384	1.075085	61.29828	TRUE	HP_421337	TGCTGTTG/	CCTCTGAG/	BC042576	expressed sequence AI480556	AI480556		2.16	SM2607	b		2	0.212551731
mPool4_V2MM_156717	1.074767	75.83547	TRUE	HP_377345	TGCTGTTG/	GTGACCTT(	XM_204182	RIKEN cDNA 1520402A15 gene	1520402A15Rik	NA				NA		0.120127608
mPool2_V2MM_89734	1.074742	3.004939	TRUE	HP_311162	TGCTGTTG/	CTCTGATTT	AK083443	zinc finger and BTB domain containing 10	Zbtb10		2.6	SM2145	g		1	1.522164293
092308m1_V2MM_157751	1.074614	2.205692	TRUE	HP_378364	TGCTGTTG/	CACAAGGA	XM_205561	Mus musculus similar to actin alpha 1 skeletal muscle protein (LOC277868), mR.			2.6	SM2117	b		12	1.656455229
092308m3_V2MM_88011	1.074361	75.83547	TRUE	HP_309458	TGCTGTTG/	CAGAGATT/	AK030734	RIKEN cD 5730427M17 gene	5730427M17Rik		2.9	SM2251	h		3	0.120127608
092308m3_V2MM_88011	1.074361	75.83547	TRUE	HP_309458	TGCTGTTG/	CAGAGATT/	AK030734	RIKEN cDNA 5730427M17 gene	5730427M17Rik		2.9	SM2251	h		3	0.120127608
092308m1_V2MM_166317	1.072477	2.205692	TRUE	HP_386847	TGCTGTTG/	GAAAGAAC	XM_285469	Mus musculus LOC333526 (LOC333526), mR.			2.6	SM2122	a		6	1.656455229
092308m3_V2MM_226144	1.072446	62.64426	TRUE	HP_478175	TGCTGTTG/	CACTGTGA(	NM_144954	peptidylprolyl isomerase (cyclophilin)-like 2	Ppil2		2.13	SM2460	e		1	0.203118686
mPool7_V2MM_100976	1.072249	2.205692	TRUE	HP_322152	TGCTGTTG/	CCACATGT	AK021047	RIKEN cDNA C030005H24 gene	C030005H24Rik		2.7	SM2162	f		5	1.656455229
092308m1_V2MM_166964	1.071827	2.205692	TRUE	HP_387486	TGCTGTTG/	CACACCTG/	XM_285643	Mus musculus similar to pol protein [Sus scrofa] (LOC331244), mR.			2.6	SM2120	e		4	1.656455229
mPool4_V2MM_211189	1.071332	75.83547	TRUE	HP_428907	TGCTGTTG/	GATGGATA	XM_140094	Mus musculus similar to protein (peptidyl-prolyl cis/trans isomerase) NIMA-interacting,			2.14	SM2543	h		12	0.120127608
092308m1_V2MM_185026	1.070939	2.205692	TRUE	HP_405498	TGCTGTTG/	CTCTCCTGC	XM_289299	Mus musculus LOC332946 (LOC332946), mR.			2.5	SM2090	c		2	1.656455229
mPool4_V2MM_127095	1.070844	73.80895	TRUE	HP_347927	TGCTGTTG/	CACAGAAT/	XM_144978	Mus musculus similar to 60S RIBOSOMAL PROTEIN L17 (L23) (AMINO ACID STARVATION			2.11	SM2363	c		10	0.131891001
092308m1_V2MM_175193	1.070763	3.004939	TRUE	HP_395678	TGCTGTTG/	CATACAGT(	XM_287389	Rap1, GTPase-activating protein 1	Rap1ga1		2.5	SM2085	e		8	1.522164293
mPool6_V2MM_47980	1.070741	69.72441	TRUE	HP_270445	TGCTGTTG/	GACAGTTT(	NM_008858	protein kinase C, mu	Prkcm		2.7	SM2187	f		7	0.156615137
092308m3_V2MM_90669	1.070686	75.83547	TRUE	HP_312085	TGCTGTTG/	CTCCTGAA(	NM_177837	hypothetical protein 6530439I21			2.13	SM2469	g		9	0.120127608
092308m1_V2MM_140396	1.0698	3.004939	TRUE	HP_361116	TGCTGTTG/	GATAATGG	XM_155935	Mus musculus LOC239788 (LOC239788), mR.			2.4	SM2032	b		3	1.522164293
092308m3_V2MM_98720	1.069336	75.83547	TRUE	HP_319946	TGCTGTTG/	GAGACAGT	XM_112920	Mus musculus LOC210461 (LOC210461), mR.			2.13	SM2455	e		7	0.120127608
092308m1_V2MM_122382	1.068725	3.004939	TRUE	HP_343250	TGCTGTTG/	CTAAGTCT	XM_142162	Mus musculus similar to protein tyrosine phosphatase 4a1 [Rattus norvegicus] (LOC245(			2.6	SM2111	c		10	1.522164293
mPool4_V2MM_60184	1.068674	75.83547	TRUE	HP_282349	TGCTGTTG/	GGATTAAA	NM_177120	RIKEN cDNA D130060J02 gene	D130060J02Rik		2.16	SM2634	c		1	0.120127608
mPool4_V2MM_133449	1.068415	75.83547	TRUE	HP_354188	TGCTGTTG/	CCTGGAA(	XM_149251	RIKEN cDNA 9430008C03 gene	9430008C03Rik		2.14	SM2550	f		2	0.120127608
092308m1_V2MM_129450	1.068051	2.205692	TRUE	HP_350257	TGCTGTTG/	CAAATTAG/	XM_146305	Mus musculus similar to pol protein [Sus scrofa] (LOC215546), mRNA.			2.6	SM2102	f		9	1.656455229
092308m1_V2MM_129450	1.068051	2.205692	TRUE	HP_350257	TGCTGTTG/	CAAATTAG/	XM_146305	Mus musculus similar to pol protein [Sus scrofa] (LOC215546), mR.			2.6	SM2102	f		9	1.656455229
092308m1_V2MM_89673	1.067954	2.205692	TRUE	HP_311101	TGCTGTTG/	CCTACACT/	NM_177645	RIKEN cD C430010P07 gene	C430010P07Rik		2.6	SM2108	e		2	1.656455229
mPool5_V2MM_15935	1.067465	75.83547	TRUE	HP_239245	TGCTGTTG/	CTGTAAGG	NM_153086	RIKEN cDNA 9430022F06 gene	9430022F06Rik	NA				NA		0.120127608
mPool2_V2MM_7485	1.067043	3.004939	TRUE	HP_230991	TGCTGTTG/	GCAAAGTT/	NM_010864	myosin Va	Myo5a		2.7	SM2164	b		12	1.522164293

mPool4_V2MM_140479	1.066998	75.83547	TRUE	HP_361198	TGCTGTTG/GCAAGAAA	XM_155983	Mus musculus LOC239813 (LOC239813), mRNA.			2.16	SM2638	f		6	0.120127608
mPool5_V2MM_20282	1.066837	75.83547	TRUE	HP_243483	TGCTGTTG/GTGTAGCA.	NM_019512	transcription elongation regulator 1 (CA150)	Tcerg1	NA				NA		0.120127608
092308m1_V2MM_137402	1.066813	2.205692	TRUE	HP_358122	TGCTGTTG/CAGTGAAA	XM_153111	Mus musculus LOC238071 (LOC238071), mR.			2.5	SM2056	a		2	1.656455229
mPool7_V2MM_98417	1.066493	3.004939	TRUE	HP_319644	TGCTGTTG/GTGACATT(	NM_198860	expressed sequence AI646023	AI646023		2.9	SM2264	d		2	1.522164293
mPool7_V2MM_98417	1.066493	3.004939	TRUE	HP_319644	TGCTGTTG/GTGACATT(	NM_198860	expressed sequence AI646023	AI646023		2.9	SM2264	d		2	1.522164293
mPool2_V2MM_203294	1.066437	2.205692	TRUE	HP_421252	TGCTGTTG/CACTTCGA(	XM_138241	Mus musculus similar to hypothetical protein A030003A19 (LOC217854), mRNA.			2.1	SM2318	a		12	1.656455229
092308m1_V2MM_168602	1.06633	2.205692	TRUE	HP_389105	TGCTGTTG/GGGAACTC	XM_286025	Mus musculus similar to Glyceraldehyde 3-phosphate dehydrogese (GAPDH) (LOC33053			2.6	SM2114	g		12	1.656455229
mPool2_V2MM_85330	1.066277	2.205692	TRUE	HP_306811	TGCTGTTG/CTTATTGA/	NM_133992	ubiquitin specific protease 52	Usp52		2.6	SM2147	g		4	1.656455229
mPool7_V2MM_92544	1.066099	3.004939	TRUE	HP_313891	TGCTGTTG/CAGAGTAC.	NM_178730	RIKEN cDNA 4732406D01 gene	4732406D01Rik		2.9	SM2259	c		10	1.522164293
092308m3_V2MM_113493	1.066081	75.83547	TRUE	HP_334480	TGCTGTTG/GCTCCATC/	XM_136990	gene model 225, (NCBI)	Gm225		2.13	SM2499	h		2	0.120127608
mPool4_V2MM_201436	1.065901	75.83547	TRUE	HP_419465	TGCTGTTG/GCAAGAAA	XM_288243	Mus musculus LOC332514 (LOC332514), mRNA.			2.16	SM2650	e		6	0.120127608
092308m1_V2MM_177744	1.065799	2.205692	TRUE	HP_398224	TGCTGTTG/GCTTCTGG(	XM_287939	Mus musculus hypothetical gene supported by AK040448 (LOC330996), mR.			2.5	SM2067	g		5	1.656455229
092308m1_V2MM_177744	1.065799	2.205692	TRUE	HP_398224	TGCTGTTG/GCTTCTGG(	XM_287939	Mus musculus hypothetical gene supported by AK040448 (LOC330996), mRNA.			2.5	SM2067	g		5	1.656455229
092308m1_V2MM_146947	1.065289	3.004939	TRUE	HP_367658	TGCTGTTG/CCTCCATTA	XM_162472	Mus musculus LOC243393 (LOC243393), mR.			2.4	SM2034	c		7	1.522164293
mPool7_V2MM_195615	1.06522	3.004939	TRUE	HP_413748	TGCTGTTG/CATAGGTG(	XM_112000	Mus musculus similar to PROBABLE POL POLYPROTEIN (LOC194862), mRNA.			2.8	SM2249	a		11	1.522164293
092308m3_V2MM_116930	1.065095	75.83547	TRUE	HP_10912	TGCTGTTG/CAGTTTGA(	XM_139159	gene model 295, (NCBI)	Gm295		2.1	SM2319	h		10	0.120127608
092308m3_V2MM_116930	1.065095	75.83547	TRUE	HP_10912	TGCTGTTG/CAGTTTGA(	XM_139159	gene model 295, (NCBI)	Gm295		2.1	SM2319	h		10	0.120127608
092308m3_V2MM_234378	1.065083	75.83547	TRUE	HP_486257	TGCTGTTG/CAGTGGTT(	XM_126867	sorting nexin 13	Snx13		2.14	SM2501	c		4	0.120127608
092308m3_V2MM_86838	1.064907	75.83547	TRUE	HP_308308	TGCTGTTG/CTGCCAAG(	NM_173784	RIKEN cD 9630054F20 gene	9630054F20Rik		2.13	SM2482	h		8	0.120127608
mPool2_V2MM_2745	1.064804	2.205692	TRUE	HP_226357	TGCTGTTG/CTGGAACT(	NM_026401	mitochondrial ribosomal protein 63	Mrp63		2.11	SM2376	a		11	1.656455229
mPool4_V2MM_242217	1.064744	75.83547	TRUE	HP_493966	TGCTGTTG/GGTCCAGT.	XM_146000	Mus musculus similar to voltage-dependent calcium channel gamma-3 subunit [Rattus r			2.14	SM2550	d		5	0.120127608
092308m3_V2MM_197702	1.064463	75.83547	TRUE	HP_415768	TGCTGTTG/GCCAGATA'	NM_181750	R3H domain (binds single-stranded nucleic acids)	R3hdm		2.13	SM2471	f		3	0.120127608
mPool2_V2MM_360	1.064181	3.004939	TRUE	HP_224030	TGCTGTTG/CACCACCT/	NM_008262	one cut domain, family member 1	Onecut1		2.11	SM2377	d		6	1.522164293
mPool4_V2MM_228959	1.064096	75.83547	TRUE	HP_480947	TGCTGTTG/CTAAGGGT.	XM_290162	Mus musculus LOC333780 (LOC333780), mRNA.		NA				NA		0.120127608
092308m1_V2MM_120081	1.063959	1.959154	TRUE	HP_340989	TGCTGTTG/CTGACAGA'	XM_140755	gene model 1285, (NCBI)	Gm1285		2.5	SM2098	g		9	1.707931489
mPool2_V2MM_111339	1.063452	3.004939	TRUE	HP_332356	TGCTGTTG/CTACCATC(	BC048657	RIKEN cDNA 1700007N14 gene	1700007N14Rik		2.7	SM2162	f		12	1.522164293
092308m3_V2MM_36601	1.06314	75.83547	TRUE	HP_259377	TGCTGTTG/CTGTTGCT(	NM_008623	myelin protein zero	Mpz		2.12	SM2435	g		7	0.120127608
092308m3_V2MM_91709	1.063074	75.83547	TRUE	HP_313078	TGCTGTTG/CACACACT(	AK044024	echinoderm microtubule associated protein like 5	Eml5		2.13	SM2466	g		4	0.120127608
092308m3_V2MM_209367	1.063001	75.83547	TRUE	HP_427140	TGCTGTTG/GCTGAAAT.	XM_487119	similar to LRRGT00126			2.14	SM2511	e		5	0.120127608
mPool7_V2MM_90534	1.062392	2.205692	TRUE	HP_311951	TGCTGTTG/CATTTAAA(	NM_177812	hypothetical protein 4932411K12			2.9	SM2260	a		7	1.656455229
mPool7_V2MM_90534	1.062392	2.205692	TRUE	HP_311951	TGCTGTTG/CATTTAAA(	NM_177812	hypothetical protein 4932411K12			2.9	SM2260	a		7	1.656455229
092308m3_V2MM_42445	1.062023	75.83547	TRUE	HP_265051	TGCTGTTG/CCATGACC/	NM_010269	ganglioside-induced differentiation-associated-protein 2	Gdap2		2.12	SM2444	a		6	0.120127608
mPool7_V2MM_61649	1.061566	3.004939	TRUE	HP_283778	TGCTGTTG/CCTTCACTG	AK007918	immunoglobulin heavy chain 1a (serum IgG2a)	Igh-1a		2.8	SM2229	g		2	1.522164293
mPool4_V2MM_131402	1.061378	75.83547	TRUE	HP_352165	TGCTGTTG/GCAAGAAT	XM_147640	hypothetical LOC209550			2.11	SM2365	b		3	0.120127608
mPool7_V2MM_208684	1.061349	2.205692	TRUE	HP_426466	TGCTGTTG/GAGACAAG	XM_156894	Mus musculus LOC213222 (LOC213222), mRNA.			2.1	SM2325	b		2	1.656455229
mPool7_V2MM_208684	1.061349	2.205692	TRUE	HP_426466	TGCTGTTG/GAGACAAG	XM_156894	Mus musculus LOC213222 (LOC213222), mR.			2.1	SM2325	b		2	1.656455229
mPool7_V2MM_199862	1.061291	2.205692	TRUE	HP_417900	TGCTGTTG/CAAGTGAA.	XM_289016	Mus musculus LOC332676 (LOC332676), mRNA.			2.9	SM2285	b		5	1.656455229
092308m1_V2MM_68043	1.061107	2.205692	TRUE	HP_290014	TGCTGTTG/CTGCATGA(	NM_022328	myeloid/lymphoid or mixed lineage-leukemia translocation to	Mllt1		2.4	SM2024	c		8	1.656455229
mPool6_V2MM_15144	1.061088	75.83547	TRUE	HP_238473	TGCTGTTG/CTATTCAGT	NM_011631	tumor rejection antigen gp96	Tra1		2.7	SM2182	h		9	0.120127608
mPool4_V2MM_214399	1.06069	75.83547	TRUE	HP_431992	TGCTGTTG/GCACAAGT(	XM_143688	Mus musculus similar to ribosomal protein S6 (LOC230046), mRNA.			2.16	SM2648	b		2	0.120127608
mPool2_V2MM_115925	1.060636	2.205692	TRUE	HP_336882	TGCTGTTG/CTGATTCA/	BC027395	RIKEN cDNA A530094I17 gene	A530094I17Rik		2.7	SM2163	c		4	1.656455229
mPool5_V2MM_266	1.060593	75.83547	TRUE	HP_223938	TGCTGTTG/CACACCCT(	NM_026418	regulator of G-protein signalling 10	Rgs10	NA				NA		0.120127608
mPool4_V2MM_132613	1.060413	75.83547	TRUE	HP_353363	TGCTGTTG/CAGACCCT/	XM_128781	lysocardiolipin acyltransferase	Lycat		2.14	SM2541	c		8	0.120127608
092308m1_V2MM_161203	1.06032	3.004939	TRUE	HP_381774	TGCTGTTG/CACACTAG(	XM_283807	Mus musculus hypothetical gene supported by AK032464 (LOC329551), mR.			2.6	SM2122	c		5	1.522164293
mPool6_V2MM_167800	1.059933	75.83547	TRUE	HP_388312	TGCTGTTG/GGAACTTT(	XM_285839	Mus musculus similar to heterochromatin protein 1 alpha [Xenopus laevis] (LOC329963)			2.7	SM2189	d		5	0.120127608
mPool4_V2MM_103876	1.059256	75.83547	TRUE	HP_325014	TGCTGTTG/CTGTGCTA(	AK088543	CWF19-like 1, cell cycle control (S. pombe)	Cwf19l1		2.16	SM2611	e		11	0.120127608
092308m3_V2MM_103164	1.058881	61.29828	TRUE	HP_324306	TGCTGTTG/CAGGCATT/	XM_128857	RIKEN cD 4631422C13 gene	4631422C13Rik		2.14	SM2502	d		1	0.212551731
mPool2_V2MM_125463	1.058296	2.205692	TRUE	HP_346307	TGCTGTTG/GTCAGACT'	XM_144020	Mus musculus similar to urea transporter [Rattus norvegicus] (LOC230714), mRNA.			2.5	SM2098	a		4	1.656455229
mPool2_V2MM_125463	1.058296	2.205692	TRUE	HP_346307	TGCTGTTG/GTCAGACT'	XM_144020	Mus musculus similar to urea transporter [Rattus norvegicus] (LOC230714), mR.			2.5	SM2098	a		4	1.656455229
mPool6_V2MM_69804	1.058202	66.95036	TRUE	HP_291728	TGCTGTTG/GTACTTGT/	NM_013761	serine racemase	Srr		2.8	SM2223	f		6	0.1742471
mPool7_V2MM_173410	1.058149	3.004939	TRUE	HP_393896	TGCTGTTG/CGTCCTACT	AK089549	RIKEN cDNA 4930401B06 gene	4930401B06Rik		2.9	SM2281	g		2	1.522164293
mPool7_V2MM_147742	1.058049	3.004939	TRUE	HP_368453	TGCTGTTG/CAACCCAG(	XM_163232	Mus musculus LOC243940 (LOC243940), mRNA.			2.1	SM2328	b		5	1.522164293
092308m1_V2MM_74764	1.056543	3.004939	TRUE	HP_296561	TGCTGTTG/CAGGCTAA.	NM_176934	RIKEN cD D930046H04 gene	D930046H04Rik		2.4	SM2029	f		1	1.522164293
mPool5_V2MM_15968	1.056227	75.83547	TRUE	HP_239277	TGCTGTTG/CTGTCCTTA	NM_054055	solute carrier family 13 (sodium-dependent dicarboxylate trar	Slc13a3	NA				NA		0.120127608
092308m1_V2MM_172502	1.056035	2.205692	TRUE	HP_392988	TGCTGTTG/CCTCACTTA	AK035848	expressed sequence AI449023	AI449023		2.5	SM2063	b		5	1.656455229
mPool2_V2MM_20348	1.055883	3.004939	TRUE	HP_243547	TGCTGTTG/GTGTTATT(	NM_007625	chromobox homolog 4 (Drosophila Pc class)	Cbx4		2.7	SM2164	e		12	1.522164293
092308m3_V2MM_47484	1.055828	62.64426	TRUE	HP_269964	TGCTGTTG/CTGCTCAG(	XM_484349	similar to RIKEN cD 1700001E04			2.12	SM2436	a		12	0.203118686
092308m3_V2MM_57318	1.055725	75.83547	TRUE	HP_279576	TGCTGTTG/CTTGAAAT	NM_172592	splicing factor, arginine/serine-rich 12	Sfrs12		2.12	SM2402	g		12	0.120127608
mPool7_V2MM_174282	1.055218	3.004939	TRUE	HP_394767	TGCTGTTG/CACCAGGG	XM_287196	Mus musculus hypothetical gene supported by AK034924 (LOC329550), mR.			2.5	SM2084	c		1	1.522164293
mPool7_V2MM_174282	1.055218	3.004939	TRUE	HP_394767	TGCTGTTG/CACCAGGG	XM_287196	Mus musculus hypothetical gene supported by AK034924 (LOC329550), mRNA.			2.5	SM2084	c		1	1.522164293
092308m3_V2MM_34276	1.055123	66.95036	TRUE	HP_125796	TGCTGTTG/CCATGATC(	NM_139061	vacuolar protein sorting 54 (yeast)	Vps54		2.12	SM2419	h		12	0.1742471
092308m3_V2MM_207845	1.054475	75.83547	TRUE	HP_425645	TGCTGTTG/GACTGATT/	XM_153454	gene model 599, (NCBI)	Gm599		2.14	SM2509	e		12	0.120127608

mPool4_V2MM_88782	1.05437	75.83547	TRUE	HP_310218	TGCTGTTG/	CACCTTATT	NM_175494	zinc finger protein 367	Zfp367		2.16	SM2616	g		11	0.120127608
mPool4_V2MM_31057	1.054283	61.29828	TRUE	HP_253967	TGCTGTTG/	CTCTCTGAC	NM_028354	RIKEN cDNA 2810481F14 gene	2810481F14Rik		2.15	SM2595	d		6	0.212551731
mPool4_V2MM_51575	1.053992	75.83547	TRUE	HP_273970	TGCTGTTG/	CTCTCGATC	XM_283179	RIKEN cDNA 4930420O11 gene	4930420O11Rik	NA				NA		0.120127608
mPool2_V2MM_6215	1.052072	3.004939	TRUE	HP_229754	TGCTGTTG/	CTCTAGATC	NM_007790	chondroitin sulfate proteoglycan 6	Cspg6		2.11	SM2381	d		1	1.522164293
092308m1_V2MM_136245	1.051001	3.004939	TRUE	HP_356965	TGCTGTTG/	CCAAATTTC	XM_151569	Mus musculus LOC241206 (LOC241206), mRNA.			2.5	SM2053	e		8	1.522164293
092308m1_V2MM_136245	1.051001	3.004939	TRUE	HP_356965	TGCTGTTG/	CCAAATTTC	XM_151569	Mus musculus LOC241206 (LOC241206), mR.			2.5	SM2053	e		8	1.522164293
mPool2_V2MM_117873	1.050928	3.004939	TRUE	HP_338813	TGCTGTTG/	GTGAAATA(	XM_139566	Mus musculus LOC239654 (LOC239654), mRNA.			2.11	SM2368	a		9	1.522164293
mPool2_V2MM_1862	1.050504	3.004939	TRUE	HP_225495	TGCTGTTG/	CGGAGTTT(	NM_054073	testis specific gene A13	Tsga13		2.11	SM2376	d		10	1.522164293
mPool2_V2MM_91444	1.050326	3.004939	TRUE	HP_252513	TGCTGTTG/	CACCCATTT	NM_178266	membrane-bound transcription factor protease, site 2	Mbtps2		2.6	SM2134	b		10	1.522164293
mPool4_V2MM_122378	1.050001	75.83547	TRUE	HP_343246	TGCTGTTG/	CCTTGAGA/	XM_142158	Mus musculus similar to Transcription factor BTF3 (RNA polymerase B transcription fact			2.14	SM2535	a		10	0.120127608
mPool6_V2MM_44055	1.049997	75.83547	TRUE	HP_266621	TGCTGTTG/	CTGTGCTG(	NM_138650	diacylglycerol kinase, gamma	Dgkg		2.7	SM2186	b		10	0.120127608
092308m3_V2MM_91042	1.049878	75.83547	TRUE	HP_312458	TGCTGTTG/	CTCCGCAT/	NM_177909	solute carrier family 9 (sodium/hydrogen exchanger), isoform	Slc9a9		2.13	SM2485	c		3	0.120127608
mPool7_V2MM_175917	1.049243	3.004939	TRUE	HP_396399	TGCTGTTG/	CCATTCAG(	AK029922	RIKEN cDNA 4930528J11 gene	4930528J11Rik		2.5	SM2083	d		4	1.522164293
mPool7_V2MM_175917	1.049243	3.004939	TRUE	HP_396399	TGCTGTTG/	CCATTCAG(	AK029922	RIKEN cD 4930528J11 gene	4930528J11Rik		2.5	SM2083	d		4	1.522164293
mPool4_V2MM_7997	1.049229	61.29828	TRUE	HP_231489	TGCTGTTG/	GCTATAGC(	NM_175449	RIKEN cDNA A630077B13 gene	A630077B13Rik		2.16	SM2608	a		10	0.212551731
092308m1_V2MM_139580	1.048557	3.004939	TRUE	HP_360300	TGCTGTTG/	CCAACATA/	XM_155213	Mus musculus LOC210767 (LOC210767), mR.			2.4	SM2044	d		2	1.522164293
092308m1_V2MM_139580	1.048557	3.004939	TRUE	HP_360300	TGCTGTTG/	CCAACATA/	XM_155213	Mus musculus LOC210767 (LOC210767), mRNA.			2.4	SM2044	d		2	1.522164293
092308m3_V2MM_232128	1.048307	75.83547	TRUE	HP_484080	TGCTGTTG/	GAAATTTA/	BC054732	gametogenetin binding protein 1			2.13	SM2490	h		12	0.120127608
mPool4_V2MM_118081	1.047956	60.13437	TRUE	HP_339018	TGCTGTTG/	GCCAATAC/	XM_139678	Mus musculus similar to G protein pathway suppressor 2 [Homo sapiens] (LOC239854),			2.16	SM2641	f		2	0.220877236
092308m1_V2MM_143461	1.047696	2.205692	TRUE	HP_364173	TGCTGTTG/	GCAGAAAC	XM_158745	Mus musculus LOC228665 (LOC228665), mR.			2.4	SM2035	f		12	1.656455229
mPool4_V2MM_61399	1.047397	75.83547	TRUE	HP_283534	TGCTGTTG/	GTGTGAAG	NM_145978	PDZ and LIM domain 2	Pdlim2		2.15	SM2591	f		11	0.120127608
092308m1_V2MM_118252	1.047317	3.004939	TRUE	HP_339189	TGCTGTTG/	GTGGAAAT	XM_139741	Fgfr1 oncogene partner	Fgfr1op		2.6	SM2111	a		7	1.522164293
092308m1_V2MM_118252	1.047317	3.004939	TRUE	HP_339189	TGCTGTTG/	GTGGAAAT	XM_139741	Fgfr1 oncogene partner	Fgfr1op		2.6	SM2111	a		7	1.522164293
mPool6_V2MM_64823	1.047046	75.83547	TRUE	HP_286872	TGCTGTTG/	CTTTGGTT(	XM_484640	similar to Ab2-450			2.7	SM2176	c		6	0.120127608
mPool7_V2MM_103141	1.046707	3.004939	TRUE	HP_324284	TGCTGTTG/	GCAAATAT	AK006077	RIKEN cD 1700018A14 gene	1700018A14Rik		2.9	SM2297	f		9	1.522164293
mPool7_V2MM_103141	1.046707	3.004939	TRUE	HP_324284	TGCTGTTG/	GCAAATAT	AK006077	RIKEN cDNA 1700018A14 gene	1700018A14Rik		2.9	SM2297	f		9	1.522164293
mPool6_V2MM_39468	1.045732	35.99881	TRUE	HP_262153	TGCTGTTG/	GGAATCAT(	NM_008778	p21 (CDKN1A)-activated kinase 3	Pak3		2.7	SM2187	h		9	0.443711899
092308m3_V2MM_55209	1.045139	75.83547	TRUE	HP_277511	TGCTGTTG/	CCCTGTGT	NM_177121	RIKEN cD B930095G15 gene	B930095G15Rik		2.12	SM2416	a		11	0.120127608
mPool7_V2MM_197443	1.044533	3.004939	TRUE	HP_140039	TGCTGTTG/	CCATTATAT	NM_011669	ubiquitin specific protease 12	Usp12		2.9	SM2258	c		9	1.522164293
mPool6_V2MM_167646	1.044461	61.29828	TRUE	HP_388159	TGCTGTTG/	GACATCAA(	XM_285809	Mus musculus similar to ORF1 (LOC332897), mRNA.			2.8	SM2201	f		4	0.212551731
092308m3_V2MM_32954	1.044446	75.83547	TRUE	HP_255822	TGCTGTTG/	CAAGGAAG	NM_145519	FERM, RhoGEF and pleckstrin domain protein 2	Farp2		2.12	SM2433	f		6	0.120127608
mPool4_V2MM_136971	1.044195	75.83547	TRUE	HP_357691	TGCTGTTG/	GCTTGGCT(	XM_152300	Mus musculus LOC237573 (LOC237573), mRNA.			2.14	SM2530	b		2	0.120127608
092308m3_V2MM_142279	1.043338	75.83547	TRUE	HP_362992	TGCTGTTG/	GTAAAGTG	XM_157529	Mus musculus LOC240393 (LOC240393), mR.			2.1	SM2339	a		2	0.120127608
092308m3_V2MM_142279	1.043338	75.83547	TRUE	HP_362992	TGCTGTTG/	GTAAAGTG	XM_157529	Mus musculus LOC240393 (LOC240393), mRNA.			2.1	SM2339	a		2	0.120127608
mPool5_V2MM_16573	1.043251	75.83547	TRUE	HP_239868	TGCTGTTG/	GACACAAA	NM_053265	RIKEN cDNA 4930433N12 gene	4930433N12Rik	NA				NA		0.120127608
mPool2_V2MM_108119	1.043203	3.004939	TRUE	HP_329192	TGCTGTTG/	CACGTGGA	AK005862	RIKEN cDNA 1700011F03 gene	1700011F03Rik		2.7	SM2164	e		1	1.522164293
mPool7_V2MM_74533	1.04288	3.004939	TRUE	HP_296335	TGCTGTTG/	CAGACTGT/	NM_026041	RIKEN cDNA 2810430M08 gene	2810430M08Rik		2.8	SM2244	f		8	1.522164293
mPool6_V2MM_73490	1.042751	75.83547	TRUE	HP_295322	TGCTGTTG/	GGCCAAAT	NM_172809	RIKEN cDNA E130115J16 gene	E130115J16Rik		2.8	SM2217	e		4	0.120127608
092308m3_V2MM_140153	1.042084	61.29828	TRUE	HP_360873	TGCTGTTG/	GAGTATGG	XM_155758	Mus musculus LOC239449 (LOC239449), mR.			2.14	SM2520	c		8	0.212551731
mPool5_V2MM_14799	1.042082	75.83547	TRUE	HP_106265	TGCTGTTG/	CGCATTGG/	XM_140330	Mus musculus LOC240277 (LOC240277), mRNA.		NA				NA		0.120127608
mPool2_V2MM_191993	1.042071	3.004939	TRUE	HP_326025	TGCTGTTG/	CAGCAGAA	NM_207298	cerebral endothelial cell adhesion molecule 1	Ceecam1		2.1	SM2319	a		4	1.522164293
mPool5_V2MM_165457	1.041182	75.83547	TRUE	HP_385994	TGCTGTTG/	CTGCCGAT(	XM_285253	similar to ribosomal protein S18		NA				NA		0.120127608
092308m1_V2MM_186362	1.04079	3.004939	TRUE	HP_406834	TGCTGTTG/	CTGGCCTT	XM_289526	Mus musculus LOC333250 (LOC333250), mR.			2.5	SM2094	h		6	1.522164293
mPool7_V2MM_70807	1.040609	3.004939	TRUE	HP_292709	TGCTGTTG/	CAGAGTGG	NM_025466	gastrokine 1	Gkn1		2.8	SM2230	b		8	1.522164293
092308m1_V2MM_135142	1.040175	3.004939	TRUE	HP_355864	TGCTGTTG/	CTGAGTAT(	XM_150350	UDP-glucuronosyltransferase 1 family, member 1	Ugt1a1		2.4	SM2049	f		2	1.522164293
092308m3_V2MM_25922	1.040067	66.95036	TRUE	HP_248979	TGCTGTTG/	CCCAGAGC	NM_008507	linker of T-cell receptor pathways	Lnk		2.12	SM2423	b		10	0.1742471
mPool6_V2MM_65976	1.039859	75.83547	TRUE	HP_288005	TGCTGTTG/	GTCTGTCC	NM_033583	protocadherin gamma subfamily C, 5	Pcdhgc5		2.8	SM2224	c		5	0.120127608
mPool2_V2MM_113971	1.039781	3.004939	TRUE	HP_334958	TGCTGTTG/	CAGAGTCA(	XM_137221	RIKEN cDNA 4930503E15 gene	4930503E15Rik		2.6	SM2148	c		4	1.522164293
mPool7_V2MM_83943	1.03967	3.004939	TRUE	HP_305465	TGCTGTTG/	CACAAGAA	NM_026279	RIKEN cDNA 2310026E23 gene	2310026E23Rik		2.9	SM2267	e		11	1.522164293
mPool7_V2MM_64334	1.03946	3.004939	TRUE	HP_286392	TGCTGTTG/	CTGCTCCC/	XM_139182	Mus musculus similar to Cell division protein kinase 4 (Cyclin-dependent kinase 4) (PSK-.			2.8	SM2234	e		7	1.522164293
mPool4_V2MM_149309	1.039383	75.83547	TRUE	HP_370019	TGCTGTTG/	CAAACCAG(	XM_164753	Mus musculus LOC244771 (LOC244771), mRNA.			2.16	SM2637	b		9	0.120127608
mPool4_V2MM_181709	1.039156	75.83547	TRUE	HP_402183	TGCTGTTG/	CAATTACT(	XM_288722	Mus musculus LOC332139 (LOC332139), mRNA.			2.16	SM2649	d		3	0.120127608
092308m3_V2MM_234790	1.038584	61.29828	TRUE	HP_486662	TGCTGTTG/	CAGTGAGG	XM_135684	RIKEN cD 1700022L20 gene	1700022L20Rik		2.13	SM2496	d		4	0.212551731
mPool2_V2MM_99576	1.038574	3.004939	TRUE	HP_320775	TGCTGTTG/	CCTTTGTAC	XM_125901	DNA segment, Chr 10, ERATO Doi 516, expressed	D10Ertd516e		2.6	SM2133	c		2	1.522164293
mPool2_V2MM_85489	1.038327	3.004939	TRUE	HP_306970	TGCTGTTG/	CGTTTATTA	NM_146394	olfactory receptor 1278	Olfr1278		2.6	SM2140	f		10	1.522164293
mPool7_V2MM_180102	1.038005	3.004939	TRUE	HP_400576	TGCTGTTG/	GAGATAGC	XM_288450	Mus musculus LOC331788 (LOC331788), mRNA.			2.9	SM2275	c		6	1.522164293
mPool7_V2MM_62812	1.03753	3.004939	TRUE	HP_284909	TGCTGTTG/	CATGGCGG	NM_013845	receptor tyrosine kinase-like orphan receptor 1	Ror1		2.8	SM2228	b		12	1.522164293
mPool5_V2MM_10711	1.037528	75.83547	TRUE	NA	NA	NA		NA	NA	NA		NA	NA	NA		0.120127608
mPool4_V2MM_35547	1.037486	69.72441	TRUE	HP_258340	TGCTGTTG/	CTACACTC(	NM_027797	RIKEN cDNA 5530601I19 gene	5530601I19Rik		2.15	SM2578	a		9	0.156615137
mPool2_V2MM_69797	1.037427	3.004939	TRUE	HP_142673	TGCTGTTG/	GTACATAT(	NM_146239	PCTAIRE-motif protein kinase 2	Pctk2		2.11	SM2392	f		4	1.522164293
mPool6_V2MM_163146	1.037413	75.83547	TRUE	HP_383702	TGCTGTTG/	GTTCTTGA(	AK054302	expressed sequence C87414	C87414		2.8	SM2210	e		5	0.120127608
092308m1_V2MM_74079	1.037347	2.205692	TRUE	HP_295894	TGCTGTTG/	CAACGGCA	NM_011121	polo-like kise 1 (Drosophila)	Plk1		2.4	SM2026	c		9	1.656455229

mPool5_V2MM_162011	1.037001	75.83547	TRUE	HP_382574	TGCTGTTG/	GTTTGCAG(	XM_284146	Mus musculus similar to ubiquitin-conjugating enzyme MHR6BN [Mus musculus] (LOC3:		NA				NA		0.120127608
mPool2_V2MM_165679	1.036851	3.004939	TRUE	HP_386213	TGCTGTTG/	CAAGAGAC	XM_285302	Mus musculus similar to 60S RIBOSOMAL PROTEIN L29 (P23) (LOC332391), mRNA.			2.6	SM2126	f		3	1.522164293
092308m3_V2MM_42041	1.036744	75.83547	TRUE	HP_264658	TGCTGTTG/	CAGTCAGT(	X12807	ubiquitin specific protease 53	Usp53		2.12	SM2441	h		7	0.120127608
mPool2_V2MM_208035	1.036333	3.004939	TRUE	HP_425832	TGCTGTTG/	GCAAAGAT	XM_154889	Mus musculus LOC239181 (LOC239181), mRNA.			2.1	SM2347	b		6	1.522164293
mPool7_V2MM_174581	1.03601	3.004939	TRUE	HP_395066	TGCTGTTG/	GTGGGTAA	XM_287256	Mus musculus hypothetical gene supported by AK035260 (LOC329691), mR.			2.5	SM2081	h		2	1.522164293
mPool7_V2MM_174581	1.03601	3.004939	TRUE	HP_395066	TGCTGTTG/	GTGGGTAA	XM_287256	Mus musculus hypothetical gene supported by AK035260 (LOC329691), mRNA.			2.5	SM2081	h		2	1.522164293
092308m3_V2MM_115612	1.035959	75.83547	TRUE	HP_336573	TGCTGTTG/	GGATCTTG	XM_138573	RIKEN cD 3110004L20 gene	3110004L20Rik		2.13	SM2493	c		7	0.120127608
mPool2_V2MM_101418	1.035595	3.004939	TRUE	HP_322589	TGCTGTTG/	CAGTCTTG/	AK029536	tetratricopeptide repeat domain 18	Ttc18		2.7	SM2155	c		10	1.522164293
mPool2_V2MM_109358	1.035054	3.004939	TRUE	HP_330419	TGCTGTTG/	CCGACAAT/	NM_207239	general transcription factor III C 1	Gtf3c1		2.7	SM2159	g		5	1.522164293
mPool4_V2MM_154320	1.034916	75.83547	TRUE	HP_374961	TGCTGTTG/	GGCCGTCC/	XM_196729	Mus musculus LOC270718 (LOC270718), mRNA.		NA				NA		0.120127608
mPool4_V2MM_73116	1.034783	75.83547	TRUE	HP_294958	TGCTGTTG/	GCAACATG(	NM_029377	RIKEN cDNA 1810054G18 gene	1810054G18Rik		2.15	SM2592	b		6	0.120127608
092308m1_V2MM_147297	1.034673	2.205692	TRUE	HP_368008	TGCTGTTG/	GCAGTTAA(	NM_162853	Mus musculus LOC243690 (LOC243690), mR.			2.4	SM2044	f		10	1.656455229
mPool7_V2MM_90318	1.034474	2.205692	TRUE	HP_311740	TGCTGTTG/	GTGACTAT/	NM_177774	serine-arginine repressor protein			2.9	SM2269	a		11	1.656455229
mPool4_V2MM_239676	1.034311	75.83547	TRUE	HP_491475	TGCTGTTG/	CATAGTCA(	XM_141952	similar to P38IP protein			2.16	SM2641	b		12	0.120127608
mPool2_V2MM_211494	1.032269	3.004939	TRUE	HP_429190	TGCTGTTG/	GACCTAGT(	U46151	t-complex-associated testis expressed 2	Tcte2		2.11	SM2359	h		8	1.522164293
092308m3_V2MM_51918	1.032177	75.83547	TRUE	HP_274305	TGCTGTTG/	CTGGAAGT	AK122577	rosbin, round spermatid basic protein 1	Rsbn1		2.12	SM2443	c		6	0.120127608
mPool5_V2MM_2668	1.031032	59.99801	TRUE	HP_226281	TGCTGTTG/	CTGCCATG/	NM_023217	pyroglutamyl-peptidase I	Pgpep1	NA				NA		0.221863149
mPool7_V2MM_178962	1.030997	3.004939	TRUE	HP_399436	TGCTGTTG/	CAGAAAGT	XM_288249	Mus musculus LOC332523 (LOC332523), mRNA.			2.9	SM2289	f		2	1.522164293
092308m3_V2MM_44089	1.030989	75.83547	TRUE	HP_266655	TGCTGTTG/	CTGTGTTG1	NM_027927	RIKEN cD 1110020M19 gene	1110020M19Rik		2.12	SM2413	a		4	0.120127608
092308m3_V2MM_97149	1.03066	75.83547	TRUE	HP_318391	TGCTGTTG/	GTATCATG(	XM_111258	Mus musculus similar to high sulfur protein B2E [Rattus norvegicus] (LOC193248), mR.			2.13	SM2459	b		12	0.120127608
092308m3_V2MM_93056	1.030498	75.83547	TRUE	HP_314390	TGCTGTTG/	GTCAGTAT	NM_178896	expressed sequence AI836376	AI836376		2.9	SM2254	e		8	0.120127608
092308m3_V2MM_93056	1.030498	75.83547	TRUE	HP_314390	TGCTGTTG/	GTCAGTAT	NM_178896	expressed sequence AI836376	AI836376		2.9	SM2254	e		8	0.120127608
092308m3_V2MM_203341	1.030199	75.83547	TRUE	HP_421296	TGCTGTTG/	CAGATTGT(	XM_485514	similar to U4/U6 small nuclear ribonucleoprotein Prp4 (U4/U6 snRNP 60 kDa protein) (V			2.9	SM2298	d		6	0.120127608
092308m3_V2MM_203341	1.030199	75.83547	TRUE	HP_421296	TGCTGTTG/	CAGATTGT(	XM_485514	similar to U4/U6 small nuclear ribonucleoprotein Prp4 (U4/U6 snRNP 60 kDa protein) (V			2.9	SM2298	d		6	0.120127608
092308m3_V2MM_83844	1.029695	75.83547	TRUE	HP_305368	TGCTGTTG/	GATTAAAT	NM_025700	phosphoglucomutase 1	Pgm1		2.9	SM2270	c		9	0.120127608
092308m3_V2MM_83844	1.029695	75.83547	TRUE	HP_305368	TGCTGTTG/	GATTAAAT	NM_025700	phosphoglucomutase 1	Pgm1		2.9	SM2270	c		9	0.120127608
092308m1_V2MM_155626	1.029604	3.004939	TRUE	HP_376265	TGCTGTTG/	GGATTCTT/	XM_197656	Mus musculus LOC271914 (LOC271914), mR.			2.6	SM2120	b		5	1.522164293
092308m3_V2MM_97554	1.029561	75.83547	TRUE	HP_318795	TGCTGTTG/	CAAACCAA(	XM_290002	gene model 865, (NCBI)	Gm865		2.9	SM2269	b		1	0.120127608
092308m3_V2MM_97554	1.029561	75.83547	TRUE	HP_318795	TGCTGTTG/	CAAACCAA(	XM_290002	gene model 865, (NCBI)	Gm865		2.9	SM2269	b		1	0.120127608
mPool7_V2MM_76042	1.029073	2.205692	TRUE	HP_297810	TGCTGTTG/	CCTGCATG(	NM_173434	RIKEN cDNA 9930111J21 gene	9930111J21Rik		2.8	SM2241	g		4	1.656455229
092308m1_V2MM_181518	1.028745	2.205692	TRUE	HP_401992	TGCTGTTG/	CTCTAAATT	XM_288689	Mus musculus LOC332089 (LOC332089), mRNA.			2.5	SM2087	c		9	1.656455229
092308m1_V2MM_181518	1.028745	2.205692	TRUE	HP_401992	TGCTGTTG/	CTCTAAATT	XM_288689	Mus musculus LOC332089 (LOC332089), mR.			2.5	SM2087	c		9	1.656455229
mPool5_V2MM_201709	1.028687	75.83547	TRUE	HP_419737	TGCTGTTG/	CCTTCATG/	XM_289635	Mus musculus LOC333397 (LOC333397), mRNA.		NA				NA		0.120127608
mPool2_V2MM_131164	1.02775	3.004939	TRUE	HP_351929	TGCTGTTG/	GGAGATAA	XM_483957	RIKEN cDNA 1810038L18 gene	1810038L18Rik		2.11	SM2367	h		6	1.522164293
mPool2_V2MM_208122	1.027538	3.004939	TRUE	HP_425917	TGCTGTTG/	GATCCGAA.	XM_193891	similar to CTCL tumor antigen se57-1			2.1	SM2346	b		6	1.522164293
mPool2_V2MM_22815	1.027533	3.004939	TRUE	HP_245958	TGCTGTTG/	CTCCTAAG(	NM_144814	REST corepressor 3	Rcor3		2.11	SM2389	a		10	1.522164293
mPool6_V2MM_194376	1.027353	75.83547	TRUE	HP_412543	TGCTGTTG/	CACACTCT/	NM_011215	protein tyrosine phosphatase, receptor type, N polypeptide 2	Ptprn2		2.7	SM2186	c		11	0.120127608
mPool6_V2MM_160997	1.027295	69.72441	TRUE	HP_381574	TGCTGTTG/	GGAGTTCA.	AK077027	RIKEN cDNA 1700123D08 gene	1700123D08Rik		2.7	SM2191	a		7	0.156615137
mPool6_V2MM_154139	1.026699	75.83547	TRUE	HP_374780	TGCTGTTG/	CTGTGATG(	XM_196600	Mus musculus LOC271617 (LOC271617), mRNA.			2.8	SM2206	b		4	0.120127608
092308m3_V2MM_52990	1.02624	75.83547	TRUE	HP_275361	TGCTGTTG/	GGCAGAAG	NM_010266	guanine deamise	Gda		2.12	SM2403	c		5	0.120127608
mPool2_V2MM_124387	1.026054	2.205692	TRUE	HP_345239	TGCTGTTG/	CATAGTAG(	XM_489896	similar to TDPOZ2			2.1	SM2349	b		4	1.656455229
mPool4_V2MM_230666	1.025793	75.83547	TRUE	HP_482651	TGCTGTTG/	CGACATTC1	XM_287672	Mus musculus hypothetical gene supported by AK039174 (LOC330486), mRNA.		NA				NA		0.120127608
mPool2_V2MM_2631	1.025366	3.004939	TRUE	HP_226244	TGCTGTTG/	CTGATTCAT	NM_134025	peroxisomal biogenesis factor 12	Pex12		2.11	SM2376	h		5	1.522164293
mPool6_V2MM_49051	1.024933	75.83547	TRUE	HP_271499	TGCTGTTG/	GCTGGTTC1	NM_021420	serine/threonine kinase 4	Stk4		2.7	SM2184	f		12	0.120127608
092308m1_V2MM_177262	1.024861	3.004939	TRUE	NA	NA	NA	NA	NA	NA	NA		NA	NA	NA		1.522164293
mPool4_V2MM_177464	1.024538	75.83547	TRUE	HP_397944	TGCTGTTG/	CAGATGGA	XM_489259	hypothetical protein 4930565O14		NA				NA		0.120127608
092308m3_V2MM_109489	1.02428	75.83547	TRUE	HP_330543	TGCTGTTG/	CATCTATCT	BC051545	RIKEN cD 1190003J15 gene	1190003J15Rik		2.14	SM2505	h		7	0.120127608
092308m3_V2MM_170614	1.024236	75.83547	TRUE	HP_391106	TGCTGTTG/	CTGTGGTT(	NM_177374	RIKEN cD 6720458F09 gene	6720458F09Rik		2.12	SM2407	e		1	0.120127608
092308m1_V2MM_181959	1.024213	3.004939	TRUE	HP_402433	TGCTGTTG/	GCACTAAG(	XM_288763	Mus musculus LOC332210 (LOC332210), mRNA.			2.5	SM2085	d		5	1.522164293
092308m1_V2MM_181959	1.024213	3.004939	TRUE	HP_402433	TGCTGTTG/	GCACTAAG(	XM_288763	Mus musculus LOC332210 (LOC332210), mR.			2.5	SM2085	d		5	1.522164293
mPool7_V2MM_96983	1.023916	3.004939	TRUE	HP_318226	TGCTGTTG/	CATCTAATT	XM_111147	Mus musculus similar to pol polyprotein [Trichosurus vulpecula] (LOC211398), mRNA.			2.9	SM2254	b		7	1.522164293
mPool2_V2MM_121182	1.023698	2.205692	TRUE	HP_342065	TGCTGTTG/	GCCACTAA(	XM_141519	RIKEN cDNA 0610030I09 gene	0610030I09Rik		2.5	SM2098	c		5	1.656455229
mPool2_V2MM_121182	1.023698	2.205692	TRUE	HP_342065	TGCTGTTG/	GCCACTAA(	XM_141519	RIKEN cD 0610030I09 gene	0610030I09Rik		2.5	SM2098	c		5	1.656455229
mPool7_V2MM_98179	1.023653	3.004939	TRUE	HP_319412	TGCTGTTG/	CAGCTGGT.	XM_112240	similar to 60S acidic ribosomal protein P1			2.9	SM2267	b		1	1.522164293
mPool4_V2MM_192324	1.023398	75.83547	TRUE	HP_411141	TGCTGTTG/	CTGAGAAG	NM_201367	gene model 1012, (NCBI)	Gm1012		2.16	SM2606	b		2	0.120127608
mPool7_V2MM_183771	1.022688	3.004939	TRUE	HP_404245	TGCTGTTG/	CCTATTAGT	XM_289077	Mus musculus LOC333530 (LOC333530), mR.			2.5	SM2084	g		11	1.522164293
mPool7_V2MM_183771	1.022688	3.004939	TRUE	HP_404245	TGCTGTTG/	CCTATTAGT	XM_289077	Mus musculus LOC333530 (LOC333530), mRNA.			2.5	SM2084	g		11	1.522164293
092308m3_V2MM_89502	1.022388	75.83547	TRUE	HP_310930	TGCTGTTG/	CAAACACT(	AK049924	hypothetical protein C630011I23			2.13	SM2462	e		12	0.120127608
mPool7_V2MM_197025	1.022161	3.004939	TRUE	HP_415111	TGCTGTTG/	GTCTGGTT	NM_178643	RIKEN cDNA C230052I12 gene	C230052I12Rik		2.9	SM2255	h		2	1.522164293
mPool2_V2MM_14560	1.022023	3.004939	TRUE	HP_237905	TGCTGTTG/	CCTGTAAA(	NM_008314	5-hydroxytryptamine (serotonin) receptor 5A	Htr5a		2.11	SM2376	a		10	1.522164293
mPool7_V2MM_203535	1.021606	3.004939	TRUE	HP_205610	TGCTGTTG/	CAGTATAA	XM_139351	similar to cadherin 12, type 2 preproprotein; Br-cadherin; cadherin-12; N-cadherin 2; br			2.1	SM2302	d		7	1.522164293
092308m1_V2MM_83736	1.021131	3.004939	TRUE	HP_305262	TGCTGTTG/	GGGACTAA	NM_024286	popeye domain containing 3	Popdc3		2.6	SM2107	c		5	1.522164293

mPool7_V2MM_101488	1.020777	2.205692	TRUE	HP_322659	TGCTGTTG/	GCTTTACCA	AK016963	RIKEN cDNA 4933428D01 gene	4933428D01Rik		2.9	SM2300	d		9	1.656455229
092308m3_V2MM_141336	1.020763	75.83547	TRUE	HP_362049	TGCTGTTG/	GTGTCAAA(	XM_156590	Mus musculus LOC239860 (LOC239860), mR.			2.14	SM2515	g		7	0.120127608
092308m3_V2MM_106905	1.020321	75.83547	TRUE	HP_327997	TGCTGTTG/	CTGGCTGT(	XM_131670	Mus musculus similar to rhomboid-related protein [Homo sapiens] (LOC230727), mRNA			2.1	SM2314	a		2	0.120127608
092308m3_V2MM_106905	1.020321	75.83547	TRUE	HP_327997	TGCTGTTG/	CTGGCTGT(	XM_131670	Mus musculus similar to rhomboid-related protein [Homo sapiens] (LOC230727), mR.			2.1	SM2314	a		2	0.120127608
092308m3_V2MM_107803	1.020263	75.83547	TRUE	NA	NA	NA	NA	NA	NA	NA		NA	NA	NA		0.120127608
mPool4_V2MM_122555	1.019963	75.83547	TRUE	HP_343420	TGCTGTTG/	GCTATTCTC	XM_142262	patched domain containing 1	Ptchd1		2.14	SM2540	e		1	0.120127608
092308m3_V2MM_84105	1.019841	75.83547	TRUE	HP_305623	TGCTGTTG/	CTGTTCTTA	BC072625	RIKEN cD 1500010J02 gene	1500010J02Rik		2.13	SM2482	c		1	0.120127608
092308m3_V2MM_54351	1.019756	75.83547	TRUE	HP_276681	TGCTGTTG/	CAGTCTGT(	NM_010293	glucokise activity, related sequence 1	Gk-rs1		2.12	SM2413	g		10	0.120127608
092308m1_V2MM_178244	1.019755	3.004939	TRUE	HP_398718	TGCTGTTG/	CAAGCATC	AK032250	hypothetical protein 6430511F03			2.5	SM2077	c		10	1.522164293
mPool4_V2MM_215074	1.019408	62.64426	TRUE	HP_432642	TGCTGTTG/	CCTTCGCC/	XM_146272	Mus musculus similar to eukaryotic translation initiation factor 3, subunit 3 gamma, 40k			2.16	SM2647	a		2	0.203118686
mPool7_V2MM_205539	1.018911	2.205692	TRUE	HP_423398	TGCTGTTG/	GAGCTTAA(	XM_127914	Mus musculus RIKEN cDNA 4921505C17 gene (4921505C17Rik), mRNA.			2.9	SM2297	h		1	1.656455229
mPool4_V2MM_23788	1.018786	75.83547	TRUE	HP_246908	TGCTGTTG/	GAAAGAAA	NM_145509	RIKEN cDNA 5430435G22 gene	5430435G22Rik	NA				NA		0.120127608
092308m1_V2MM_171419	1.018642	3.004939	TRUE	HP_391908	TGCTGTTG/	CTTACATCT	AK050207	expressed sequence AI448102	AI448102		2.5	SM2089	b		2	1.522164293
092308m1_V2MM_171419	1.018642	3.004939	TRUE	HP_391908	TGCTGTTG/	CTTACATCT	AK050207	expressed sequence AI448102	AI448102		2.5	SM2089	b		2	1.522164293
mPool7_V2MM_175932	1.018612	3.004939	TRUE	HP_396414	TGCTGTTG/	CAGGAATA	XM_287564	Mus musculus hypothetical gene supported by AK089795 (LOC330271), mRNA.			2.5	SM2072	b		10	1.522164293
mPool7_V2MM_175932	1.018612	3.004939	TRUE	HP_396414	TGCTGTTG/	CAGGAATA	XM_287564	Mus musculus hypothetical gene supported by AK089795 (LOC330271), mR.			2.5	SM2072	b		10	1.522164293
092308m1_V2MM_118975	1.018479	3.004939	TRUE	HP_339904	TGCTGTTG/	CAGACATG	XM_140147	Mus musculus similar to ribosomal protein L3 [Bos taurus] (LOC225107), mR.			2.6	SM2104	a		8	1.522164293
mPool4_V2MM_240424	1.018023	75.83547	TRUE	HP_492205	TGCTGTTG/	GATATTCTT	XM_146535	ATP-binding cassette, sub-family E (OABP), member 1	Abce1		2.14	SM2547	g		9	0.120127608
092308m3_V2MM_29496	1.017978	75.83547	TRUE	HP_252457	TGCTGTTG/	CACAGCCA(	NM_009154	sema domain, seven thrombospondin repeats (type 1 and typ	Sema5a		2.12	SM2419	c		4	0.120127608
mPool7_V2MM_175377	1.017508	3.004939	TRUE	NA	NA	NA	NA	NA	NA	NA		NA	NA	NA		1.522164293
092308m1_V2MM_117965	1.017506	3.004939	TRUE	HP_338904	TGCTGTTG/	CTTAGATG	XM_139620	Mus musculus similar to protein phosphatase 2 (formerly 2A), regulatory subunit A (PR (			2.6	SM2104	e		1	1.522164293
092308m1_V2MM_117965	1.017506	3.004939	TRUE	HP_338904	TGCTGTTG/	CTTAGATG	XM_139620	Mus musculus similar to protein phosphatase 2 (formerly 2A), regulatory subunit A (PR (			2.6	SM2104	e		1	1.522164293
mPool2_V2MM_65699	1.017324	3.004939	TRUE	HP_287733	TGCTGTTG/	GGGAAGAA	XM_489719	similar to Calcium-dependent phospholipase A2 precursor (Phosphatidylcholine 2-acylh			2.7	SM2166	f		12	1.522164293
mPool2_V2MM_129888	1.017254	3.004939	TRUE	HP_350689	TGCTGTTG/	GAGCTGAC	BC041776	RIKEN cDNA D630040G17 gene	D630040G17Rik		2.11	SM2353	g		6	1.522164293
092308m1_V2MM_132331	1.017208	3.004939	TRUE	HP_353085	TGCTGTTG/	CCAGTTCT(	XM_358823	leucine rich repeat containing 15	Lrrc15		2.5	SM2099	f		9	1.522164293
092308m1_V2MM_132331	1.017208	3.004939	TRUE	HP_353085	TGCTGTTG/	CCAGTTCT(	XM_358823	leucine rich repeat containing 15	Lrrc15		2.5	SM2099	f		9	1.522164293
092308m1_V2MM_177372	1.017051	2.205692	TRUE	HP_397852	TGCTGTTG/	GGCTTAAA(	XM_287859	EST AA546796	AA546796		2.5	SM2086	h		12	1.656455229
092308m3_V2MM_87647	1.016675	75.83547	TRUE	HP_309097	TGCTGTTG/	CCACTATA/	NM_175212	RIKEN cD 4930438D12 gene	4930438D12Rik		2.9	SM2263	f		5	0.120127608
092308m3_V2MM_87647	1.016675	75.83547	TRUE	HP_309097	TGCTGTTG/	CCACTATA/	NM_175212	RIKEN cDNA 4930438D12 gene	4930438D12Rik		2.9	SM2263	f		5	0.120127608
092308m3_V2MM_103151	1.016447	60.13437	TRUE	HP_198972	TGCTGTTG/	GGAAGTTT(	AK129176	RIKEN cD 4933432P15 gene	4933432P15Rik		2.13	SM2490	h		10	0.220877236
mPool7_V2MM_81933	1.01615	2.205692	TRUE	HP_303521	TGCTGTTG/	GTGTCAAT/	NM_011228	RAB33A, member of RAS oncogene family	Rab33a		2.8	SM2248	d		2	1.656455229
mPool4_V2MM_198821	1.016099	75.83547	TRUE	HP_416875	TGCTGTTG/	CTGTCTCTT	XM_158119	Mus musculus LOC241223 (LOC241223), mRNA.			2.16	SM2642	b		6	0.120127608
092308m3_V2MM_195113	1.016007	75.83547	TRUE	HP_413262	TGCTGTTG/	CCCTTTAG(	NM_177770	cD sequence BC043934	BC043934		2.9	SM2257	g		5	0.120127608
092308m3_V2MM_195113	1.016007	75.83547	TRUE	HP_413262	TGCTGTTG/	CCCTTTAG(	NM_177770	cDNA sequence BC043934	BC043934		2.9	SM2257	g		5	0.120127608
mPool4_V2MM_72595	1.015902	75.83547	TRUE	HP_294449	TGCTGTTG/	CTTACTGG(	NM_146768	olfactory receptor 1099	Olfr1099		2.15	SM2596	d		5	0.120127608
mPool7_V2MM_182098	1.015748	2.205692	TRUE	HP_402572	TGCTGTTG/	CAATATAG/	XM_288786	Mus musculus LOC332247 (LOC332247), mRNA.			2.5	SM2075	c		5	1.656455229
092308m1_V2MM_117945	1.01553	3.004939	TRUE	HP_338884	TGCTGTTG/	GCAACTTC/	XM_139608	Mus musculus similar to glyceraldehyde 3-phosphate dehydrogese [Cavia porcellus] (LO			2.6	SM2101	g		5	1.522164293
092308m1_V2MM_166779	1.015057	2.205692	TRUE	HP_387306	TGCTGTTG/	CGAAGTAA	XM_285585	similar to hypothetical protein MGC18736			2.6	SM2117	b		8	1.656455229
mPool2_V2MM_87712	1.014996	3.004939	TRUE	HP_309162	TGCTGTTG/	CAGAAGAA	NM_175229	serine/arginine repetitive matrix 2	Srrm2		2.6	SM2146	d		9	1.522164293
mPool4_V2MM_34143	1.014927	75.83547	TRUE	HP_256973	TGCTGTTG/	CCAGATTA	NM_133702	RIKEN cDNA 1500002M01 gene	1500002M01Rik		2.15	SM2599	f		6	0.120127608
092308m3_V2MM_38454	1.014729	75.83547	TRUE	HP_261179	TGCTGTTG/	GCATCTATT	NM_030725	syptotagmin 13	Syt13		2.12	SM2402	b		1	0.120127608
mPool5_V2MM_16666	1.014572	75.83547	TRUE	HP_239960	TGCTGTTG/	GACATGAA	NM_008356	interleukin 13 receptor, alpha 2	Il13ra2	NA				NA		0.120127608
092308m3_V2MM_44042	1.014523	75.83547	TRUE	HP_266608	TGCTGTTG/	CTGTGAGC/	NM_008992	ATP-binding cassette, sub-family D (ALD), member 4	Abcd4		2.12	SM2405	d		3	0.120127608
092308m3_V2MM_105139	1.014326	75.83547	TRUE	HP_326263	TGCTGTTG/	CAGTCGAA	AK016085	RIKEN cD 4930550G17 gene	4930550G17Rik		2.14	SM2504	h		2	0.120127608
mPool6_V2MM_160128	1.014306	66.95036	TRUE	HP_380713	TGCTGTTG/	GTTGCTATT	XM_283382	Mus musculus hypothetical gene supported by AK045597 (LOC328725), mRNA.			2.8	SM2203	e		7	0.1742471
mPool2_V2MM_146752	1.014109	3.004939	TRUE	HP_367463	TGCTGTTG/	GCACAATT(	XM_162261	Mus musculus LOC208184 (LOC208184), mRNA.			2.4	SM2046	f		6	1.522164293
mPool2_V2MM_146752	1.014109	3.004939	TRUE	HP_367463	TGCTGTTG/	GCACAATT(	XM_162261	Mus musculus LOC208184 (LOC208184), mR.			2.4	SM2046	f		6	1.522164293
092308m3_V2MM_141285	1.013997	75.83547	TRUE	HP_361998	TGCTGTTG/	CCTGCACA/	XM_156562	Mus musculus LOC224376 (LOC224376), mR.			2.4	SM2031	b		7	0.120127608
092308m1_V2MM_139305	1.013963	3.004939	TRUE	HP_360025	TGCTGTTG/	CGTATTTG	XM_154906	Mus musculus LOC210795 (LOC210795), mR.			2.4	SM2034	b		10	1.522164293
mPool7_V2MM_94362	1.013487	3.004939	TRUE	HP_315654	TGCTGTTG/	CACTTTAG(	NM_183138	cDNA sequence BC037432	BC037432		2.9	SM2267	b		3	1.522164293
mPool2_V2MM_36920	1.013437	3.004939	TRUE	HP_259692	TGCTGTTG/	GAACGTTA	XM_487594	similar to hypothetical protein 4930509O22			2.11	SM2392	g		4	1.522164293
mPool6_V2MM_156731	1.013234	75.83547	TRUE	HP_42605	TGCTGTTG/	GCCAGCAG	AK007436	a disintegrin-like and metalloprotease (reprolysin type) with t	Adamts9		2.7	SM2191	h		3	0.120127608
092308m3_V2MM_83918	1.011765	75.83547	TRUE	HP_305440	TGCTGTTG/	CACAGTGA	NM_026225	component of oligomeric golgi complex 6	Cog6		2.13	SM2469	c		1	0.120127608
092308m3_V2MM_96517	1.011348	75.83547	TRUE	HP_317768	TGCTGTTG/	CAGCCAAA(	XM_110787	UPF3 regulator of nonsense transcripts homolog B (yeast)	Upf3b		2.13	SM2459	h		5	0.120127608
092308m1_V2MM_88072	1.011282	2.205692	TRUE	HP_309518	TGCTGTTG/	GACAGTTT/	AK030699	a disintegrin-like and metalloprotease (reprolysin type) with t	Adamts9		2.6	SM2112	f		5	1.656455229
092308m3_V2MM_207279	1.010713	75.83547	TRUE	HP_425090	TGCTGTTG/	GGAAACAG	XM_157114	Mus musculus LOC212321 (LOC212321), mRNA.			2.1	SM2345	d		5	0.120127608
092308m3_V2MM_207279	1.010713	75.83547	TRUE	HP_425090	TGCTGTTG/	GGAAACAG	XM_157114	Mus musculus LOC212321 (LOC212321), mR.			2.1	SM2345	d		5	0.120127608
mPool2_V2MM_12407	1.010561	3.004939	TRUE	HP_235802	TGCTGTTG/	CAAAGCAG	BC055079	cytidine monophospho-N-acetylneuraminic acid hydroxylase	Cmah		2.11	SM2384	b		2	1.522164293
092308m3_V2MM_142710	1.010315	75.83547	TRUE	HP_363423	TGCTGTTG/	CTCAGCTT(	XM_157854	Mus musculus LOC240626 (LOC240626), mR.			2.14	SM2514	e		6	0.120127608
mPool2_V2MM_95486	1.010231	2.205692	TRUE	HP_316762	TGCTGTTG/	GATATAAA	NM_198014	RIKEN cDNA 9630044O09 gene	9630044O09Rik		2.6	SM2143	d		7	1.656455229
mPool2_V2MM_214278	1.010164	2.205692	TRUE	HP_431879	TGCTGTTG/	CCCTTCAAT	XM_149291	hypothetical gene supported by AK012899			2.11	SM2364	b		7	1.656455229
mPool4_V2MM_241460	1.01016	69.72441	TRUE	HP_493218	TGCTGTTG/	GACAGACC	NM_145475	ceramide kinase	Cerk		2.14	SM2538	a		11	0.156615137

092308m3_V2MM_47098	1.009889	75.83547	TRUE	HP_269588	TGCTGTTG/	CTATGCAA1	NM_138671	cD sequence BC004012	BC004012		2.12	SM2406	a		4	0.120127608
mPool7_V2MM_174426	1.009885	3.004939	TRUE	HP_394911	TGCTGTTG/	CCGTGATT(	XM_287229	Mus musculus hypothetical gene supported by AK077932 (LOC329624), mRNA.			2.5	SM2077	d		12	1.522164293
mPool7_V2MM_174426	1.009885	3.004939	TRUE	HP_394911	TGCTGTTG/	CCGTGATT(	XM_287229	Mus musculus hypothetical gene supported by AK077932 (LOC329624), mR.			2.5	SM2077	d		12	1.522164293
092308m3_V2MM_101337	1.009748	75.83547	TRUE	HP_322509	TGCTGTTG/	CACCCACT/	AY262012	RIKEN cD 1810058I14 gene	1810058I14Rik		2.13	SM2499	e		11	0.120127608
092308m3_V2MM_232796	1.009462	59.99801	TRUE	HP_484729	TGCTGTTG/	CCGTTCTG1	XM_136073	RIKEN cD 4930534P07 gene	4930534P07Rik		2.13	SM2497	e		6	0.221863149
mPool4_V2MM_160731	1.009436	75.83547	TRUE	HP_381310	TGCTGTTG/	GTATGGTT(	XM_283619	Mus musculus hypothetical gene supported by AK087209 (LOC329109), mRNA.		NA				NA		0.120127608
092308m3_V2MM_112952	1.009361	75.83547	TRUE	HP_333944	TGCTGTTG/	CAGATTTG(	NM_207228	testis specific 10	Tsga10		2.1	SM2313	e		6	0.120127608
092308m3_V2MM_112952	1.009361	75.83547	TRUE	HP_333944	TGCTGTTG/	CAGATTTG(	NM_207228	testis specific 10	Tsga10		2.1	SM2313	e		6	0.120127608
mPool4_V2MM_53953	1.009128	60.13437	TRUE	HP_276294	TGCTGTTG/	CACTTTCAT	NM_146593	olfactory receptor 1111	Olfr1111		2.15	SM2600	h		6	0.220877236
mPool4_V2MM_52866	1.008972	70.87591	TRUE	HP_275238	TGCTGTTG/	GCTGCCCT1	NM_177268	ankyrin repeat domain 16	Ankrd16		2.16	SM2607	h		10	0.149501346
092308m3_V2MM_97736	1.008525	75.83547	TRUE	NA	NA	NA	NA	NA	NA	NA		NA	NA	NA		0.120127608
092308m3_V2MM_113117	1.008227	75.83547	TRUE	HP_334107	TGCTGTTG/	CACCCAAT(	XM_136832	Mus musculus similar to translation elongation factor eEF-1 alpha chain - rat (LOC22741			2.1	SM2321	f		2	0.120127608
092308m3_V2MM_113117	1.008227	75.83547	TRUE	HP_334107	TGCTGTTG/	CACCCAAT(	XM_136832	Mus musculus similar to translation elongation factor eEF-1 alpha chain - rat (LOC22741			2.1	SM2321	f		2	0.120127608
092308m1_V2MM_78563	1.00817	2.205692	TRUE	HP_91860	TGCTGTTG/	GAGAAGGA	NM_010461	homeo box B8	Hoxb8		2.4	SM2024	c		6	1.656455229
092308m3_V2MM_51341	1.008164	60.13437	TRUE	HP_273743	TGCTGTTG/	CTCACTGG(	NM_146591	olfactory receptor 1101	Olfr1101		2.12	SM2405	h		10	0.220877236
mPool4_V2MM_103762	1.007856	75.83547	TRUE	HP_324900	TGCTGTTG/	GTCTTAAC(	AK008974	RIKEN cDNA 2210418G03 gene	2210418G03Rik		2.16	SM2611	d		11	0.120127608
mPool7_V2MM_174506	1.007285	2.205692	TRUE	HP_394991	TGCTGTTG/	CGTGCATT(	XM_287244	Mus musculus hypothetical gene supported by AK051302 (LOC329648), mR.			2.5	SM2071	g		2	1.656455229
mPool7_V2MM_174506	1.007285	2.205692	TRUE	HP_394991	TGCTGTTG/	CGTGCATT(	XM_287244	Mus musculus hypothetical gene supported by AK051302 (LOC329648), mRNA.			2.5	SM2071	g		2	1.656455229
mPool2_V2MM_118793	1.007218	3.004939	TRUE	HP_339723	TGCTGTTG/	CCGTAACT(	XM_140066	Mus musculus similar to caspr5 protein isoform 1 (LOC215422), mRNA.			2.11	SM2373	h		7	1.522164293
mPool2_V2MM_177570	1.00699	3.004939	TRUE	HP_398050	TGCTGTTG/	CGCATTGT(	AF064874	hyperpolarization-activated, cyclic nucleotide-gated K+ 4	Hcn4		2.11	SM2383	h		8	1.522164293
mPool2_V2MM_26867	1.006194	3.004939	TRUE	HP_249901	TGCTGTTG/	CTGACATT(	NM_008577	solute carrier family 3 (activators of dibasic and neutral aminc	Slc3a2		2.11	SM2392	h		2	1.522164293
mPool2_V2MM_95815	1.005847	2.205692	TRUE	HP_317083	TGCTGTTG/	CACTGAGG	XM_147508	similar to hypothetical protein MGC39650			2.6	SM2134	f		7	1.656455229
mPool2_V2MM_125961	1.005739	3.004939	TRUE	HP_346804	TGCTGTTG/	CTAACTGT/	XM_144310	RIKEN cDNA 6820424L24 gene	6820424L24Rik		2.11	SM2356	c		3	1.522164293
mPool7_V2MM_135599	1.005654	3.004939	TRUE	HP_356321	TGCTGTTG/	CCTGTTGA(	XM_150820	Mus musculus LOC240684 (LOC240684), mRNA.			2.1	SM2328	h		6	1.522164293
092308m3_V2MM_226962	1.005203	75.83547	TRUE	HP_478970	TGCTGTTG/	GATTTCTTT	NM_011879	IK cytokine	Ik		2.13	SM2468	d		9	0.120127608
mPool4_V2MM_117124	1.004907	75.83547	TRUE	HP_338071	TGCTGTTG/	CAAGGACT(	XM_139231	Mus musculus similar to ferritin light chain 1 [Rattus norvegicus] (LOC223167), mRNA.			2.16	SM2631	c		12	0.120127608
mPool2_V2MM_100976	1.00486	2.205692	TRUE	HP_322152	TGCTGTTG/	CCACATGT1	AK021047	RIKEN cDNA C030005H24 gene	C030005H24Rik		2.7	SM2162	f		5	1.656455229
mPool4_V2MM_126152	1.004792	75.83547	TRUE	NA	NA	NA	NA	NA	NA	NA		NA	NA	NA		0.120127608
092308m3_V2MM_46080	1.004251	70.87591	TRUE	HP_268594	TGCTGTTG/	CATACGAT(	NM_009619	a disintegrin and metalloprotease domain 3 (cyritestin)	Adam3		2.12	SM2411	h		8	0.149501346
mPool4_V2MM_193599	1.003953	75.83547	TRUE	HP_411978	TGCTGTTG/	CCACCCTG1	XM_196564	gene model 1857, (NCBI)	Gm1857	NA				NA		0.120127608
mPool7_V2MM_97565	1.003879	2.205692	TRUE	HP_318806	TGCTGTTG/	CTCCTGTTC	XM_111696	Mus musculus similar to bM332P19.4 (novel 7 transmembrane receptor (rhodopsin fam			2.9	SM2254	a		12	1.656455229
mPool7_V2MM_83471	1.003796	1.959154	TRUE	HP_305011	TGCTGTTG/	CACACATT/	NM_016976	glutamate receptor, metabotropic 1	Grm1		2.9	SM2267	a		2	1.707931489
092308m3_V2MM_236801	1.003457	70.87591	TRUE	HP_488656	TGCTGTTG/	CTGAACCA(	XM_156561	Mus musculus LOC224374 (LOC224374), mR.			2.14	SM2513	a		8	0.149501346
092308m3_V2MM_40451	1.003434	75.83547	TRUE	HP_263114	TGCTGTTG/	GTCTCCCT1	BC023187	cell division cycle 27 homolog (S. cerevisiae)	Cdc27		2.12	SM2423	a		8	0.120127608
mPool4_V2MM_237471	1.003214	75.83547	TRUE	HP_489317	TGCTGTTG/	CGGCCATT1	XM_165211	Mus musculus LOC244976 (LOC244976), mRNA.			2.14	SM2530	d		11	0.120127608
mPool7_V2MM_61822	1.00277	3.004939	TRUE	HP_283944	TGCTGTTG/	CAATATTT(	NM_029466	ADP-ribosylation factor-like 8	Arl8		2.8	SM2236	h		6	1.522164293
mPool2_V2MM_125790	1.002692	3.004939	TRUE	HP_346633	TGCTGTTG/	CATTCAAC/	XM_144231	Mus musculus similar to cadherin EGF LAG seven-pass G-type receptor 2; EGF-like-doma			2.11	SM2351	f		7	1.522164293
mPool6_V2MM_162043	1.002089	75.83547	TRUE	HP_382604	TGCTGTTG/	CAGCCCAG.	XM_284161	F-box and leucine-rich repeat protein 18	Fbxl18		2.7	SM2196	d		1	0.120127608
mPool4_V2MM_60911	1.001915	75.83547	TRUE	HP_283058	TGCTGTTG/	GTCATTGA(	NM_016783	progesterone receptor membrane component 1	Pgrmc1		2.15	SM2572	h		3	0.120127608
mPool4_V2MM_155733	1.001903	75.83547	TRUE	HP_376372	TGCTGTTG/	CTAGCAAC(	XM_197725	Mus musculus LOC271995 (LOC271995), mRNA.		NA				NA		0.120127608
mPool4_V2MM_66644	1.001814	75.83547	TRUE	HP_288655	TGCTGTTG/	CATAGGAG	NM_177386	Scm-like with four mbt domains 2	Sfmbt2		2.16	SM2625	g		10	0.120127608
092308m3_V2MM_149151	1.001777	75.83547	TRUE	HP_369862	TGCTGTTG/	CTCTTCATG	XM_164545	Mus musculus LOC244536 (LOC244536), mRNA.			2.1	SM2341	g		1	0.120127608
092308m3_V2MM_149151	1.001777	75.83547	TRUE	HP_369862	TGCTGTTG/	CTCTTCATG	XM_164545	Mus musculus LOC244536 (LOC244536), mR.			2.1	SM2341	g		1	0.120127608
mPool2_V2MM_137293	1.001524	3.004939	TRUE	HP_358013	TGCTGTTG/	CCATTATA(	XM_152993	Mus musculus similar to hypothetical protein FLJ33318 [Homo sapiens] (LOC217121), m			2.1	SM2338	a		7	1.522164293
mPool7_V2MM_202634	1.001395	3.004939	TRUE	HP_420632	TGCTGTTG/	GAACAGTG	XM_130353	RIKEN cDNA 4833423E24 gene	4833423E24Rik		2.1	SM2301	a		4	1.522164293
mPool2_V2MM_146803	1.001393	3.004939	TRUE	HP_367514	TGCTGTTG/	GACGAGTA	XM_162338	Mus musculus LOC243316 (LOC243316), mRNA.			2.1	SM2336	h		11	1.522164293

Supplementary Table 2: Positive regulators of CSR recovered by the shRNA screen

Gene.Name	log2.ratio	q.value...	Hit?	Codex_hair	Codex_97m	Codex_19m	Codex_Accession	Codex_gene.name	Codex_gene.symbol	OB_Release	OB_Plate	OB_Well_Row	OB_Well_Col
mPool2_V2MM_97177	-3.01397	3.004939	TRUE	HP_318419	TGCTGTTG/	CACTGGAAT	XM_111300	Mus musculus LOC193319 (LOC193319), mRNA.		2.6	SM2143	h	2
mPool2_V2MM_134134	-2.76328	3.004939	TRUE	HP_354862	TGCTGTTG/	GATGTCAT/	XM_149660	RIKEN cDNA 2510016D11 gene	2510016D11Rik	2.11	SM2355	f	5
092308m3_V2MM_85278	-2.60582	94.45693	TRUE	HP_306762	TGCTGTTG/	CTACTCTAA	AK036588	RIKEN cD 4930504E06 gene	4930504E06Rik	2.13	SM2463	d	8
mPool2_V2MM_103707	-2.59919	3.004939	TRUE	HP_324845	TGCTGTTG/	CCAGCCGT(	D17583	proprotein convertase subtilisin/kexin type 5	Pcsk5	2.7	SM2162	a	7
mPool2_V2MM_97000	-2.49072	3.004939	TRUE	HP_318243	TGCTGTTG/	GCTACACAT	XM_111149	Mus musculus LOC193100 (LOC193100), mRNA.		2.6	SM2144	c	3
mPool4_V2MM_7180	-2.46141	61.94057	TRUE	HP_230695	TGCTGTTG/	GAGATTAT	XM_284546	similar to 14-3-3 protein tau (14-3-3 protein theta)		2.15	SM2568	h	5
mPool7_V2MM_97179	-2.43531	3.004939	TRUE	HP_318421	TGCTGTTG/	GCTGCACT(	XM_111300	Mus musculus LOC193319 (LOC193319), mRNA.		2.9	SM2261	a	4
mPool6_V2MM_165503	-2.40109	75.98381	TRUE	HP_386040	TGCTGTTG/	CTCCTAGT(	XM_285264	Mus musculus similar to p47 protein [Rattus norvegicus] (LOC328893), mRNA.		2.8	SM2211	d	12
092308m3_V2MM_108572	-2.39461	84.31789	TRUE	HP_329639	TGCTGTTG/	GCAAGATT	NM_001004182	similar to hypothetical protein MGC33926		2.14	SM2509	a	8
mPool2_V2MM_118099	-2.39241	3.004939	TRUE	HP_339036	TGCTGTTG/	CTGCCATA(	XM_139687	Mus musculus similar to cell division cycle 5-like; CDC5 (cell division cycle 5, S. pombe,		2.11	SM2354	f	1
092308m1_V2MM_167492	-2.36025	3.004939	TRUE	HP_388005	TGCTGTTG/	GGCTATCC(	XM_285777	Mus musculus similar to Involved in chitin synthase III activity, also required for homo		2.6	SM2118	f	1
092308m1_V2MM_75974	-2.28985	3.004939	TRUE	HP_297743	TGCTGTTG/	CCTCCAGG/	NM_146919	olfactory receptor 1188	Olfr1188	2.3	SM2013	f	11
mPool2_V2MM_120528	-2.26478	3.004939	TRUE	HP_341422	TGCTGTTG/	GCTTCCAA/	XM_140984	Mus musculus similar to pyrophosphatase (inorganic); inorganic pyrophosphatase [Hc		2.11	SM2358	b	5
mPool2_V2MM_101727	-2.22288	3.004939	TRUE	HP_322892	TGCTGTTG/	GTGACCCA(	NM_207636	fibronectin type III domain containing 3	Fndc3	2.7	SM2154	b	8
mPool7_V2MM_140140	-2.21765	2.577834	TRUE	HP_360860	TGCTGTTG/	CGCCAAGA	XM_155748	Mus musculus LOC239680 (LOC239680), mRNA.		2.1	SM2331	c	2
092308m3_V2MM_49812	-2.21119	94.45693	TRUE	HP_7094	TGCTGTTG/	CAAGAAAT.	NM_010074	dipeptidylpeptidase 4	Dpp4	2.12	SM2405	f	6
mPool7_V2MM_103335	-2.18174	3.004939	TRUE	HP_324477	TGCTGTTG/	CGGGTCCC	XM_128950	AFG3(ATPase family gene 3)-like 2 (yeast)	Afg3l2	2.7	SM2163	e	12
092308m1_V2MM_181263	-2.13466	3.004939	TRUE	HP_401737	TGCTGTTG/	GAAGCACA	XM_288646	Mus musculus LOC332051 (LOC332051), mR.		2.5	SM2064	g	12
092308m1_V2MM_120524	-2.13057	2.577834	TRUE	HP_341418	TGCTGTTG/	GACGCTTC(	XM_140984	Mus musculus similar to pyrophosphatase (inorganic); inorganic pyrophosphatase [Hc		2.5	SM2098	c	2
mPool7_V2MM_186646	-2.12306	2.577834	TRUE	HP_407116	TGCTGTTG/	GCCCTGAG.	XM_289573	Mus musculus LOC333325 (LOC333325), mRNA.		2.5	SM2096	a	1
mPool7_V2MM_186646	-2.12306	2.577834	TRUE	HP_407116	TGCTGTTG/	GCCCTGAG.	XM_289573	Mus musculus LOC333325 (LOC333325), mR.		2.5	SM2096	a	1
092308m1_V2MM_142193	-2.11035	3.004939	TRUE	HP_362906	TGCTGTTG/	GAGACAAG	XM_157391	Mus musculus LOC240317 (LOC240317), mR.		2.5	SM2055	d	1
092308m1_V2MM_142193	-2.11035	3.004939	TRUE	HP_362906	TGCTGTTG/	GAGACAAG	XM_157391	Mus musculus LOC240317 (LOC240317), mRNA.		2.5	SM2055	d	1
mPool6_V2MM_161518	-2.10709	94.45693	TRUE	HP_382082	TGCTGTTG/	CCCAAGAA(	AK076999	hypothetical protein 4931422A14		2.7	SM2193	h	12
092308m1_V2MM_172001	-2.1042	2.577834	TRUE	HP_392487	TGCTGTTG/	GGTGTGCA	XM_286736	Mus musculus hypothetical gene supported by AK084346 (LOC328677), mR.		2.5	SM2066	d	3
092308m3_V2MM_194937	-2.08482	94.45693	TRUE	HP_413087	TGCTGTTG/	CATGACAC(	XM_113160	similar to serine/threonine protein kise 6; aurora-A		2.13	SM2464	c	12
092308m1_V2MM_186646	-2.08014	3.004939	TRUE	HP_407116	TGCTGTTG/	GCCCTGAG.	XM_289573	Mus musculus LOC333325 (LOC333325), mRNA.		2.5	SM2096	a	1
092308m1_V2MM_186646	-2.08014	3.004939	TRUE	HP_407116	TGCTGTTG/	GCCCTGAG.	XM_289573	Mus musculus LOC333325 (LOC333325), mR.		2.5	SM2096	a	1
092308m3_V2MM_22267	-2.06953	94.45693	TRUE	HP_245425	TGCTGTTG/	CGCTCAGT	NM_008522	lactotransferrin	Ltf	2.12	SM2428	g	8
mPool2_V2MM_211021	-2.06336	2.577834	TRUE	HP_428753	TGCTGTTG/	CGCTTCCA/	XM_140984	Mus musculus similar to pyrophosphatase (inorganic); inorganic pyrophosphatase [Hc		2.11	SM2374	f	4
092308m1_V2MM_65132	-2.05203	3.004939	TRUE	HP_287177	TGCTGTTG/	GAGTTCAA/	NM_026268	dual specificity phosphatase 6	Dusp6	2.3	SM2016	c	10
mPool2_V2MM_140138	-2.04771	2.577834	TRUE	HP_360858	TGCTGTTG/	GCCAAGAG	XM_155748	Mus musculus LOC239680 (LOC239680), mRNA.		2.1	SM2341	a	6
mPool6_V2MM_168564	-2.03668	61.94057	TRUE	HP_389067	TGCTGTTG/	CAAACAAG	XM_286019	Mus musculus similar to hypothetical protein FLJ37794 [Homo sapiens] (LOC330529),		2.7	SM2194	e	11
mPool2_V2MM_11840	-2.01509	3.004939	TRUE	HP_235249	TGCTGTTG/	GGCAAGCA	XM_283677	Cdc42 binding protein kinase alpha	Cdc42bpa	2.11	SM2376	d	7
092308m1_V2MM_187359	-2.01502	3.004939	TRUE	HP_407828	TGCTGTTG/	GACAGCAG	XM_289742	Mus musculus LOC333650 (LOC333650), mR.		2.5	SM2091	f	1
mPool2_V2MM_110610	-2.01085	3.004939	TRUE	HP_331643	TGCTGTTG/	CGTGCGGG	XM_134865	pygopus 1	Pygo1	2.7	SM2161	h	8
mPool2_V2MM_94729	-1.99219	3.004939	TRUE	HP_316013	TGCTGTTG/	GACCAGGA	NM_183223	hypothetical protein D730018G16		2.6	SM2133	b	11
092308m1_V2MM_155300	-1.9908	3.004939	TRUE	HP_375939	TGCTGTTG/	GAACTTGC(	XM_197339	Mus musculus LOC271478 (LOC271478), mR.		2.6	SM2124	f	8
092308m3_V2MM_103329	-1.98731	94.45693	TRUE	HP_324471	TGCTGTTG/	CACTCCATC	XM_128949	RIKEN cD 2310002L13 gene	2310002L13Rik	2.14	SM2502	b	12
mPool6_V2MM_71959	-1.96709	94.45693	TRUE	HP_293827	TGCTGTTG/	CTCCTCATC	NM_177390	myosin ID	Myo1d	2.8	SM2220	c	11
092308m1_V2MM_128861	-1.95678	3.004939	TRUE	HP_349674	TGCTGTTG/	CAGTCACA(	XM_145950	Mus musculus LOC244149 (LOC244149), mR.		NA			NA
mPool2_V2MM_132536	-1.94503	2.577834	TRUE	HP_353287	TGCTGTTG/	GTGAGCAG	AF032130	diabetic embryopathy 1	Dep1	2.11	SM2354	c	12
mPool2_V2MM_210646	-1.94248	3.004939	TRUE	HP_428389	TGCTGTTG/	CACAAGCC.	XM_145353	Mus musculus similar to hypothetical protein C230069C04 [Mus musculus] (LOC2438:		2.1	SM2350	h	7
mPool7_V2MM_70933	-1.93896	3.004939	TRUE	HP_292833	TGCTGTTG/	CAGGAAAT.	NM_007495	astrotactin 1	Astn1	2.8	SM2235	e	12
mPool7_V2MM_141291	-1.93618	3.004939	TRUE	HP_362004	TGCTGTTG/	CCAAAGGA	XM_156564	Mus musculus LOC239914 (LOC239914), mRNA.		2.5	SM2051	h	6
mPool7_V2MM_141291	-1.93618	3.004939	TRUE	HP_362004	TGCTGTTG/	CCAAAGGA	XM_156564	Mus musculus LOC239914 (LOC239914), mR.		2.5	SM2051	h	6
092308m1_V2MM_136313	-1.93453	2.577834	TRUE	HP_357033	TGCTGTTG/	GACACAGA	XM_151633	Mus musculus LOC237290 (LOC237290), mRNA.		2.4	SM2047	b	9
092308m1_V2MM_136313	-1.93453	2.577834	TRUE	HP_357033	TGCTGTTG/	GACACAGA	XM_151633	Mus musculus LOC237290 (LOC237290), mR.		2.4	SM2047	b	9
mPool2_V2MM_18822	-1.92008	3.004939	TRUE	HP_242064	TGCTGTTG/	GCTTGAGG	NM_010918	natural killer tumor recognition sequence	Nktr	2.11	SM2379	a	1
092308m1_V2MM_124412	-1.91646	3.004939	TRUE	HP_345264	TGCTGTTG/	GCAAGAGC	XM_143432	Mus musculus similar to envelope protein [Ovis aries] (LOC229636), mR.		2.5	SM2099	h	3

mPool7_V2MM_91890	-1.90488	3.004939	TRUE	HP_313255	TGCTGTTG	GTCCCATA	NM_178611	leukocyte-associated Ig-like receptor 1	Lair1	2.9	SM2258	e	2
mPool7_V2MM_74594	-1.89434	3.004939	TRUE	HP_296395	TGCTGTTG	CAGATCCT	NM_026028	RIKEN cDNA 2700091N06 gene	2700091N06Rik	2.8	SM2230	c	4
mPool2_V2MM_138194	-1.88746	3.004939	TRUE	HP_358914	TGCTGTTG	GCAAGGAG	XM_154022	Mus musculus LOC238593 (LOC238593), mRNA.		2.1	SM2335	e	12
mPool4_V2MM_122906	-1.87838	94.45693	TRUE	HP_343763	TGCTGTTG	GATGTTAA	XM_142494	Mus musculus similar to serine (or cysteine) proteinase inhibitor, clade B, member 6;		2.16	SM2634	f	4
mPool7_V2MM_203354	-1.878	3.004939	TRUE	HP_421309	TGCTGTTG	CCAGTGCA	XM_129707	RIKEN cDNA 9230112K08 gene	9230112K08Rik	2.1	SM2302	c	3
mPool6_V2MM_76167	-1.86641	61.94057	TRUE	HP_297933	TGCTGTTG	CCTTGTCCT	NM_010653	killer cell lectin-like receptor subfamily C, member 2	Klrc2	2.8	SM2220	c	6
mPool4_V2MM_123129	-1.86044	69.72441	TRUE	HP_343985	TGCTGTTG	GTCATCTC	XM_284594	Mus musculus similar to RIKEN cDNA D130040H23; hypothetical protein D130040H23		2.14	SM2547	h	4
092308m1_V2MM_127303	-1.84752	3.004939	TRUE	HP_348134	TGCTGTTG	CTGTGACT	AK032959	RIKEN cD A430107O13 gene	A430107O13Rik	2.5	SM2100	g	9
092308m1_V2MM_127303	-1.84752	3.004939	TRUE	HP_348134	TGCTGTTG	CTGTGACT	AK032959	RIKEN cDNA A430107O13 gene	A430107O13Rik	2.5	SM2100	g	9
mPool7_V2MM_102694	-1.83953	2.577834	TRUE	HP_323841	TGCTGTTG	GTCCCAGA	XM_128550	RIKEN cDNA 1700065O13 gene	1700065O13Rik	2.1	SM2317	f	8
mPool6_V2MM_76415	-1.83549	94.45693	TRUE	HP_298174	TGCTGTTG	CGGAAACT	NM_007496	AT motif binding factor 1	Atbf1	2.8	SM2214	g	9
092308m1_V2MM_148226	-1.83106	3.004939	TRUE	HP_368937	TGCTGTTG	GATAGCAA	XM_163616	Mus musculus LOC210679 (LOC210679), mRNA.		2.4	SM2037	h	5
092308m1_V2MM_148226	-1.83106	3.004939	TRUE	HP_368937	TGCTGTTG	GATAGCAA	XM_163616	Mus musculus LOC210679 (LOC210679), mR.		2.4	SM2037	h	5
mPool2_V2MM_104143	-1.82611	3.004939	TRUE	HP_325279	TGCTGTTG	GACACAGA	AK090278	myelin protein zero-like 1	Mpzl1	2.7	SM2155	d	3
mPool7_V2MM_114876	-1.80953	3.004939	TRUE	HP_335849	TGCTGTTG	GAGAGGCT	XM_137986	inner mitochondrial membrane peptidase 2-like (S. cerev	Immp2l	2.1	SM2313	c	6
mPool7_V2MM_95836	-1.8095	3.004939	TRUE	HP_317104	TGCTGTTG	CAAGGAAA	BC059847	Rap guanine nucleotide exchange factor (GEF) 6	Rapgef6	2.9	SM2251	e	2
mPool2_V2MM_103974	-1.80287	3.004939	TRUE	HP_325111	TGCTGTTG	CTACTCAG	AK011565	RIKEN cDNA 2610027F03 gene	2610027F03Rik	2.6	SM2148	d	2
092308m1_V2MM_68829	-1.80261	2.577834	TRUE	HP_290775	TGCTGTTG	GAGCCAAG	NM_028784	coagulation factor XIII, A1 subunit	F13a1	2.3	SM2010	g	6
mPool2_V2MM_136313	-1.80045	3.004939	TRUE	HP_357033	TGCTGTTG	GACACAGA	XM_151633	Mus musculus LOC237290 (LOC237290), mR.		2.4	SM2047	b	9
mPool2_V2MM_136313	-1.80045	3.004939	TRUE	HP_357033	TGCTGTTG	GACACAGA	XM_151633	Mus musculus LOC237290 (LOC237290), mRNA.		2.4	SM2047	b	9
mPool2_V2MM_104189	-1.80013	3.004939	TRUE	HP_325325	TGCTGTTG	GCAAGAAG	XM_129579	activating transcription factor 6	Atf6	2.9	SM2299	e	2
mPool4_V2MM_207646	-1.79911	61.94057	TRUE	HP_425449	TGCTGTTG	CCTCCTCCT	XM_155091	Mus musculus LOC239237 (LOC239237), mRNA.		2.16	SM2637	e	7
mPool4_V2MM_28071	-1.79667	94.45693	TRUE	HP_251066	TGCTGTTG	GCAATAGT	NM_145548	cytochrome P450, family 2, subfamily j, polypeptide 13	Cyp2j13	2.15	SM2580	c	6
mPool2_V2MM_18443	-1.79286	3.004939	TRUE	HP_241691	TGCTGTTG	GCGATACC	NM_008173	nuclear receptor subfamily 3, group C, member 1	Nr3c1	2.7	SM2165	e	8
mPool7_V2MM_135220	-1.79265	3.004939	TRUE	HP_355942	TGCTGTTG	GAAGGCAG	AK005539	RIKEN cDNA 1600025M17 gene	1600025M17Rik	2.4	SM2046	c	6
mPool7_V2MM_135220	-1.79265	3.004939	TRUE	HP_355942	TGCTGTTG	GAAGGCAG	AK005539	RIKEN cD 1600025M17 gene	1600025M17Rik	2.4	SM2046	c	6
mPool2_V2MM_103335	-1.79255	3.004939	TRUE	HP_324477	TGCTGTTG	CGGGTCCC	XM_128950	AFG3(ATPase family gene 3)-like 2 (yeast)	Afg3l2	2.7	SM2163	e	12
mPool4_V2MM_183220	-1.78564	61.94057	TRUE	HP_403694	TGCTGTTG	GACATTTA	XM_288981	Mus musculus LOC332636 (LOC332636), mRNA.		NA			NA
mPool7_V2MM_184317	-1.77685	3.004939	TRUE	HP_404789	TGCTGTTG	GCTAGGAG	XM_289177	Mus musculus LOC332792 (LOC332792), mRNA.		2.9	SM2283	e	7
092308m3_V2MM_107135	-1.77631	94.45693	TRUE	HP_328225	TGCTGTTG	CAGATCGA	BC060239	RIKEN cD 2610109H07 gene	2610109H07Rik	2.1	SM2316	e	12
092308m3_V2MM_107135	-1.77631	94.45693	TRUE	HP_328225	TGCTGTTG	CAGATCGA	BC060239	RIKEN cDNA 2610109H07 gene	2610109H07Rik	2.1	SM2316	e	12
mPool2_V2MM_105604	-1.76756	3.004939	TRUE	HP_326715	TGCTGTTG	GCCTTAAG	XM_130642	RIKEN cDNA 5430405G05 gene	5430405G05Rik	2.7	SM2159	d	4
092308m3_V2MM_60570	-1.76738	91.10404	TRUE	HP_282724	TGCTGTTG	GGTCAGCT	NM_177112	RIKEN cD A730037L19 gene	A730037L19Rik	2.12	SM2445	g	10
mPool2_V2MM_104028	-1.76222	3.004939	TRUE	HP_325165	TGCTGTTG	CTGCACCA	AK007727	protein phosphatase 1, regulatory (inhibitor) subunit 12	Ppp1r12b	2.6	SM2150	d	8
mPool4_V2MM_133732	-1.76035	94.45693	TRUE	HP_354465	TGCTGTTG	CTCCATAG	XM_149430	RIKEN cDNA 1110019J04 gene	1110019J04Rik	2.14	SM2543	h	11
mPool2_V2MM_6674	-1.75621	3.004939	TRUE	HP_230202	TGCTGTTG	CTTCTGAG	NM_023480	fumarylacetoacetate hydrolase domain containing 1	Fahd1	2.11	SM2374	e	11
mPool2_V2MM_209710	-1.7515	3.004939	TRUE	HP_427475	TGCTGTTG	CCAGCGCA	XM_164952	Mus musculus LOC244840 (LOC244840), mRNA.		2.1	SM2338	g	3
092308m3_V2MM_26723	-1.74485	94.45693	TRUE	HP_249759	TGCTGTTG	CTCCTACA	NM_146290	olfactory receptor 125	Olfr125	2.12	SM2423	b	1
092308m1_V2MM_169934	-1.74329	3.004939	TRUE	HP_390429	TGCTGTTG	GAATTGGC	XM_488597	hypothetical protein A730013G04		2.6	SM2116	d	1
mPool4_V2MM_39314	-1.74239	61.94057	TRUE	HP_262004	TGCTGTTG	GCTTCATA	NM_019700	pseudouridine synthase 1	Pus1	2.15	SM2571	a	2
092308m1_V2MM_179056	-1.7409	2.577834	TRUE	HP_399530	TGCTGTTG	GAGACACA	XM_288267	Mus musculus LOC332540 (LOC332540), mR.		2.5	SM2062	c	8
mPool2_V2MM_83999	-1.73108	3.004939	TRUE	HP_305519	TGCTGTTG	GTGCAGGT	NM_026555	reticulocalbin 3, EF-hand calcium binding domain	Rcn3	2.6	SM2146	g	5
mPool2_V2MM_131830	-1.72847	2.577834	TRUE	HP_352590	TGCTGTTG	CTTCCCTTA	XM_147853	RIKEN cDNA 6330531I01 gene	6330531I01Rik	2.11	SM2352	b	6
mPool4_V2MM_134335	-1.72829	81.56557	TRUE	HP_355061	TGCTGTTG	GCAATGTT	XM_149793	RIKEN cDNA 1700013D24 gene	1700013D24Rik	2.16	SM2636	b	1
092308m3_V2MM_94017	-1.72819	61.94057	TRUE	HP_315314	TGCTGTTG	GTCCATCT	NM_183014	zinc finger protein 184 (Kruppel-like)	Zfp184	2.13	SM2467	g	11
mPool2_V2MM_214630	-1.7239	3.004939	TRUE	HP_432211	TGCTGTTG	GGAGCACA	XM_142373	Mus musculus similar to transitin - chicken (LOC235747), mRNA.		2.11	SM2370	c	5
mPool2_V2MM_82543	-1.7208	3.004939	TRUE	HP_304115	TGCTGTTG	CTACGCAC	NM_009712	arylsulfatase B	Arsb	2.6	SM2135	a	10
mPool7_V2MM_77611	-1.71416	3.004939	TRUE	HP_299326	TGCTGTTG	CTGTGTGA	NM_013862	RAB GTPase activating protein 1-like	Rabgap1l	2.8	SM2244	d	12
092308m1_V2MM_128847	-1.71414	2.577834	TRUE	HP_349660	TGCTGTTG	GGCTTCAT	XM_145945	gene model 493, (NCBI)	Gm493	2.6	SM2114	a	2
092308m1_V2MM_128453	-1.70527	3.004939	TRUE	HP_349267	TGCTGTTG	GCATCAGA	XM_145689	similar to glyceraldehyde-3-phosphate dehydrogese		2.6	SM2101	b	2
092308m1_V2MM_128453	-1.70527	3.004939	TRUE	HP_349267	TGCTGTTG	GCATCAGA	XM_145689	similar to glyceraldehyde-3-phosphate dehydrogenase		2.6	SM2101	b	2
mPool2_V2MM_162734	-1.70277	3.004939	TRUE	HP_383292	TGCTGTTG	CGGAGAAG	NM_001002770	RIKEN cDNA C330011M18 gene	C330011M18Rik	2.6	SM2132	a	6

mPool7_V2MM_205110	-1.70119	3.004939	TRUE	HP_422986	TGCTGTTG/	CAGCTTCC(	XM_129248	RIKEN cDNA E430027O22 gene	E430027O22Rik	2.1	SM2306	f	12
mPool7_V2MM_148841	-1.69981	2.577834	TRUE	HP_369552	TGCTGTTG/	CAAGTCAT/	XM_164191	Mus musculus LOC214871 (LOC214871), mRNA.		2.1	SM2331	e	9
mPool2_V2MM_123260	-1.69908	3.004939	TRUE	HP_344116	TGCTGTTG/	GAGCACAC	XM_142848	Mus musculus similar to glyceraldehyde-3-phosphate dehydrogenase [Mus musculus]		2.11	SM2362	h	5
mPool2_V2MM_104196	-1.6928	3.004939	TRUE	HP_325332	TGCTGTTG/	CTCTGCTC/	XM_129596	SLAM family member 8	Slamf8	2.7	SM2161	c	3
mPool2_V2MM_157493	-1.68681	3.004939	TRUE	HP_378112	TGCTGTTG/	CCAAGGGA	XM_205299	Mus musculus similar to Putative nuclear protein family member, nematode specific [		2.6	SM2129	h	10
mPool6_V2MM_74478	-1.68356	94.45693	TRUE	HP_296280	TGCTGTTG/	CAGAAATT(	NM_033564	Mpv17 transgene, kidney disease mutant-like	Mpv17l	2.7	SM2173	c	2
mPool6_V2MM_223220	-1.68255	61.94057	TRUE	HP_475395	TGCTGTTG/	CAGAAGTC	XM_205300	RIKEN cDNA 6330583I20 gene	6330583I20Rik	2.8	SM2204	g	11
mPool2_V2MM_137651	-1.67784	3.004939	TRUE	HP_358371	TGCTGTTG/	GACCCAGG	XM_153438	Mus musculus LOC238181 (LOC238181), mRNA.		2.1	SM2341	g	6
mPool7_V2MM_208359	-1.67542	3.004939	TRUE	HP_426151	TGCTGTTG/	CAGCAGAA	XM_193873	gene model 630, (NCBI)	Gm630	2.1	SM2325	a	4
092308m3_V2MM_112509	-1.67193	61.94057	TRUE	HP_333513	TGCTGTTG/	CATTACATC	XM_136532	Mus musculus similar to ribosomal protein S11 [Rattus norvegicus] (LOC241025), mR.		2.13	SM2495	h	2
mPool6_V2MM_79246	-1.67003	61.94057	TRUE	HP_300913	TGCTGTTG/	GATGTATC/	NM_030199	RIKEN cDNA 2610029D06 gene	2610029D06Rik	2.8	SM2222	d	1
092308m1_V2MM_90225	-1.66895	3.004939	TRUE	HP_311648	TGCTGTTG/	CGAAAGCC	NM_177759	hypothetical protein C130098D09		2.6	SM2107	e	6
092308m1_V2MM_90225	-1.66895	3.004939	TRUE	HP_311648	TGCTGTTG/	CGAAAGCC	NM_177759	hypothetical protein C130098D09		2.6	SM2107	e	6
092308m1_V2MM_127671	-1.66774	3.004939	TRUE	HP_348493	TGCTGTTG/	CTCAGCAT(	BC056221	zinc finger protein 583	Zfp583	2.5	SM2097	c	5
mPool2_V2MM_123359	-1.66748	3.004939	TRUE	HP_344215	TGCTGTTG/	GACCACAA(	XM_142917	Mus musculus similar to ubiquitin-protein ligase (EC 6.3.2.19) - mouse (LOC207950), n		2.5	SM2098	f	6
mPool2_V2MM_123359	-1.66748	3.004939	TRUE	HP_344215	TGCTGTTG/	GACCACAA(	XM_142917	Mus musculus similar to ubiquitin-protein ligase (EC 6.3.2.19) - mouse (LOC207950), n		2.5	SM2098	f	6
092308m3_V2MM_116736	-1.6642	66.95036	TRUE	HP_337687	TGCTGTTG/	CTATCCAT/	XM_487340	similar to MGC64482 protein		2.13	SM2492	b	7
mPool4_V2MM_20522	-1.65974	94.45693	TRUE	HP_243716	TGCTGTTG/	GTTTGATC/	XM_140427	similar to Profilin I		2.16	SM2636	b	11
mPool6_V2MM_79410	-1.65445	94.45693	TRUE	HP_301071	TGCTGTTG/	GCAATATT(	NM_010599	potassium voltage-gated channel, shaker-related subfam	Kcnab3	2.8	SM2216	f	3
mPool2_V2MM_6980	-1.64164	3.004939	TRUE	HP_230502	TGCTGTTG/	GACCGCAA	NM_019499	MAD2 (mitotic arrest deficient, homolog)-like 1 (yeast)	Mad2l1	2.11	SM2375	a	9
092308m1_V2MM_183272	-1.63987	3.004939	TRUE	HP_403746	TGCTGTTG/	CAAAGGGA	XM_288990	Mus musculus LOC332654 (LOC332654), mRNA.		2.5	SM2092	d	8
092308m1_V2MM_183272	-1.63987	3.004939	TRUE	HP_403746	TGCTGTTG/	CAAAGGGA	XM_288990	Mus musculus LOC332654 (LOC332654), mR.		2.5	SM2092	d	8
mPool2_V2MM_128453	-1.63312	3.004939	TRUE	HP_349267	TGCTGTTG/	GCATCAGA	XM_145689	similar to glyceraldehyde-3-phosphate dehydrogenase		2.6	SM2101	b	2
mPool2_V2MM_128453	-1.63312	3.004939	TRUE	HP_349267	TGCTGTTG/	GCATCAGA	XM_145689	similar to glyceraldehyde-3-phosphate dehydrogese		2.6	SM2101	b	2
mPool2_V2MM_119061	-1.63155	3.004939	TRUE	HP_339984	TGCTGTTG/	CCGTCCATC	XM_140198	RIKEN cDNA 9430020K01 gene	9430020K01Rik	2.1	SM2339	g	10
092308m1_V2MM_173738	-1.62256	3.004939	TRUE	HP_394224	TGCTGTTG/	CAACCCAA(	XM_287084	Mus musculus LOC329351 (LOC329351), mR.		2.5	SM2089	g	9
mPool4_V2MM_162858	-1.6199	61.94057	TRUE	HP_383416	TGCTGTTG/	GACTCAGA	XM_284524	Mus musculus hypothetical gene supported by AK034278 (LOC330994), mRNA.		NA			NA
mPool7_V2MM_198838	-1.61612	3.004939	TRUE	HP_416892	TGCTGTTG/	CACACCTT/	XM_488967	hypothetical gene supported by AK076684		2.9	SM2293	h	1
092308m3_V2MM_90076	-1.61511	84.31789	TRUE	HP_311501	TGCTGTTG/	GTCAGAAG	NM_177728	hypothetical protein 9430004M15		2.13	SM2457	d	8
mPool6_V2MM_222498	-1.60069	94.45693	TRUE	HP_474758	TGCTGTTG/	GAGAGACC	AK129480	RIKEN cDNA 5330421F07 gene	5330421F07Rik	2.7	SM2189	g	5
092308m1_V2MM_179796	-1.59801	3.004939	TRUE	HP_400270	TGCTGTTG/	GGCTTGGA	XM_288398	Mus musculus LOC331719 (LOC331719), mR.		2.5	SM2080	e	2
mPool2_V2MM_13615	-1.59662	3.004939	TRUE	HP_236980	TGCTGTTG/	CCAAGGAG	NM_031192	renin 1 structural	Ren1	2.11	SM2380	b	2
mPool2_V2MM_120401	-1.59644	3.004939	TRUE	HP_341299	TGCTGTTG/	GAAAGACA	XM_140902	Mus musculus similar to hypothetical protein DKFZp761J139 [Homo sapiens] (LOC227		2.11	SM2355	d	4
092308m3_V2MM_22100	-1.59545	75.98381	TRUE	HP_245262	TGCTGTTG/	CGAAGAAG	NM_031405	arsete resistance protein 2		2.11	SM2400	c	5
mPool7_V2MM_180334	-1.59438	3.004939	TRUE	HP_400808	TGCTGTTG/	CCTCACAA1	XM_288490	Mus musculus LOC331843 (LOC331843), mRNA.		2.9	SM2274	e	12
092308m3_V2MM_32592	-1.58446	81.56557	TRUE	HP_255470	TGCTGTTG/	GTATCTATC	AY266418	expressed sequence AI553587	AI553587	2.11	SM2398	f	9
mPool7_V2MM_104189	-1.58242	3.004939	TRUE	HP_325325	TGCTGTTG/	GCAAGAAG	XM_129579	activating transcription factor 6	Atf6	2.9	SM2299	e	2
092308m1_V2MM_145253	-1.58196	3.004939	TRUE	HP_365964	TGCTGTTG/	CACTGAAA(	XM_160706	Mus musculus LOC214721 (LOC214721), mR.		2.4	SM2047	h	6
092308m1_V2MM_61821	-1.58193	3.004939	TRUE	HP_283943	TGCTGTTG/	CAATAGGA	NM_009804	catalase	Cat	2.3	SM2012	e	12
mPool6_V2MM_63084	-1.58116	94.45693	TRUE	HP_285174	TGCTGTTG/	CCTCAGCT/	NM_030189	RIKEN cDNA 4930555I21 gene	4930555I21Rik	2.7	SM2180	g	2
mPool4_V2MM_22743	-1.57862	79.16495	TRUE	HP_245889	TGCTGTTG/	CTCATGGA(	NM_008698	4-nitrophenylphosphatase domain and non-neuronal SN.	Nipsnap1	2.15	SM2568	a	1
092308m3_V2MM_51765	-1.57826	84.31789	TRUE	HP_274158	TGCTGTTG/	CTGAGCGT(	NM_016739	GPI-anchored membrane protein 1	Gpiap1	2.12	SM2407	f	1
mPool4_V2MM_202299	-1.57639	70.87591	TRUE	HP_420310	TGCTGTTG/	GCACTTGG(	XM_127683	RIKEN cDNA 4930474F22 gene	4930474F22Rik	2.16	SM2617	h	7
092308m1_V2MM_148837	-1.57423	3.004939	TRUE	HP_369548	TGCTGTTG/	GAGCAAGT	XM_164191	Mus musculus LOC214871 (LOC214871), mR.		2.1	SM2326	e	7
092308m1_V2MM_148837	-1.57423	3.004939	TRUE	HP_369548	TGCTGTTG/	GAGCAAGT	XM_164191	Mus musculus LOC214871 (LOC214871), mRNA.		2.1	SM2326	e	7
mPool2_V2MM_134882	-1.57268	3.004939	TRUE	HP_355606	TGCTGTTG/	GTCAGCAG	XM_150145	guanylate cyclase 1, soluble, alpha 2	Gucy1a2	2.11	SM2360	b	7
092308m3_V2MM_103023	-1.56319	94.45693	TRUE	HP_324168	TGCTGTTG/	GCAAATGA	AK010224	protein phosphatase 1B, magnesium dependent, beta isc	Ppm1b	2.1	SM2321	c	12
092308m3_V2MM_103023	-1.56319	94.45693	TRUE	HP_324168	TGCTGTTG/	GCAAATGA	AK010224	protein phosphatase 1B, magnesium dependent, beta isc	Ppm1b	2.1	SM2321	c	12
mPool4_V2MM_120517	-1.56156	94.45693	TRUE	HP_341411	TGCTGTTG/	CATATTATT	XM_140972	similar to glyceraldehyde-3-phosphate dehydrogenase		2.16	SM2632	b	6
mPool2_V2MM_89190	-1.55945	2.577834	TRUE	HP_310620	TGCTGTTG/	GACTGTCC(	NM_177346	G protein-coupled receptor 149	Gpr149	2.6	SM2108	e	5
mPool2_V2MM_89190	-1.55945	2.577834	TRUE	HP_310620	TGCTGTTG/	GACTGTCC(	NM_177346	G protein-coupled receptor 149	Gpr149	2.6	SM2108	e	5
mPool6_V2MM_73215	-1.55536	94.45693	TRUE	HP_295054	TGCTGTTG/	GCCATGAG	NM_013643	protein tyrosine phosphatase, non-receptor type 5	Ptpn5	2.7	SM2174	f	12

092308m1_V2MM_78488	-1.55322	3.004939	TRUE	HP_300171	TGCTGTTG	GACTTCAT	NM_146712	Mus musculus olfactory receptor MOR258-4P (MOR258-4P), mR.			2.3	SM2011	c		12
mPool7_V2MM_204887	-1.55242	3.004939	TRUE	HP_422772	TGCTGTTG	CATCCTTTA	XM_136303	Mus musculus similar to ribosomal protein S24 [Rattus norvegicus] (LOC226643), mRN			2.1	SM2314	h		9
mPool6_V2MM_93304	-1.55031	94.45693	TRUE	HP_296408	TGCTGTTG	CAGATGCT	NM_181061	SoxLZ/Sox6 leucine zipper binding protein in testis	Solt		2.7	SM2180	c		6
mPool7_V2MM_137419	-1.54713	2.577834	TRUE	HP_358139	TGCTGTTG	GCATCACA	XM_153137	Mus musculus LOC238086 (LOC238086), mRNA.			2.1	SM2327	a		12
092308m3_V2MM_85598	-1.54514	83.62456	TRUE	HP_307079	TGCTGTTG	CTCCTTAAT	NM_146420	olfactory receptor 738	Olfr738		2.13	SM2473	g		5
092308m3_V2MM_42118	-1.54333	70.87591	TRUE	HP_264734	TGCTGTTG	CAGTTAAA	NM_177008	RIKEN cD 4932435O22 gene	4932435O22Rik		2.11	SM2395	h		7
mPool2_V2MM_131607	-1.53988	3.004939	TRUE	HP_352369	TGCTGTTG	GCCACACT	NM_001002771	RIKEN cDNA A830005F24 gene	A830005F24Rik		2.11	SM2367	f		7
mPool2_V2MM_86435	-1.53751	2.577834	TRUE	HP_307911	TGCTGTTG	CGAGAGCA	NM_172371	solute carrier family 16 (monocarboxylic acid transporter	Slc16a13		2.6	SM2138	d		2
mPool4_V2MM_241660	-1.53351	73.80895	TRUE	HP_493416	TGCTGTTG	GCTTCCTTA	XM_146007	Mus musculus similar to pol protein [Homo sapiens] (LOC233858), mRNA.			2.14	SM2532	h		1
092308m3_V2MM_227242	-1.52956	94.45693	TRUE	HP_479245	TGCTGTTG	CTTCTCCCA	XM_111220	Mus musculus similar to Chain A, Horf6 A Novel Human Peroxidase Enzyme (LOC1931			2.13	SM2480	d		11
092308m3_V2MM_20884	-1.52836	84.31789	TRUE	HP_244069	TGCTGTTG	CACCAGAG	NM_175687	RIKEN cD A230050P20 gene	A230050P20Rik		2.11	SM2398	e		9
mPool2_V2MM_100937	-1.52681	3.004939	TRUE	HP_322113	TGCTGTTG	GACTGCTG	AK005570	RIKEN cDNA 1600032L17 gene	1600032L17Rik		2.6	SM2149	f		11
mPool4_V2MM_261981	-1.52658	91.10404	TRUE	HP_522118	TGCTGTTG	GATATCTC	NM_027218	RIKEN cDNA 1810046I24 gene	1810046I24Rik		2.15	SM2578	e		6
092308m3_V2MM_114121	-1.52459	94.45693	TRUE	HP_335107	TGCTGTTG	CCTCAACT	XM_137280	similar to E2a-Pbx1-associated protein; amyloid-beta precursor protein intracellular d			2.13	SM2498	c		10
092308m1_V2MM_148683	-1.52063	3.004939	TRUE	HP_369394	TGCTGTTG	CAGGTCAA	XM_164030	Mus musculus LOC244396 (LOC244396), mR.			2.4	SM2033	a		5
mPool2_V2MM_114687	-1.52061	3.004939	TRUE	HP_335661	TGCTGTTG	GAAGGACA	XM_137873	Mus musculus similar to hypothetical protein MGC15396 [Homo sapiens] (LOC215062			2.7	SM2155	b		11
092308m1_V2MM_123359	-1.5176	3.004939	TRUE	HP_344215	TGCTGTTG	GACCACAA	XM_142917	Mus musculus similar to ubiquitin-protein ligase (EC 6.3.2.19) - mouse (LOC207950), n			2.5	SM2098	f		6
092308m1_V2MM_123359	-1.5176	3.004939	TRUE	HP_344215	TGCTGTTG	GACCACAA	XM_142917	Mus musculus similar to ubiquitin-protein ligase (EC 6.3.2.19) - mouse (LOC207950), n			2.5	SM2098	f		6
mPool2_V2MM_110128	-1.51571	3.004939	TRUE	HP_331169	TGCTGTTG	GCCACTTT	XM_134412	expressed sequence AA589507	AA589507		2.6	SM2150	a		11
mPool2_V2MM_212456	-1.50644	3.004939	TRUE	HP_430122	TGCTGTTG	CAGCGGCT	XM_145988	Mus musculus similar to SKAP55 homologue [Mus musculus] (LOC233817), mRNA.			2.11	SM2355	a		6
mPool4_V2MM_19230	-1.50379	61.94057	TRUE	HP_242459	TGCTGTTG	GGCATTGT	NM_053265	RIKEN cDNA 4930433N12 gene	4930433N12Rik		2.15	SM2586	b		10
mPool4_V2MM_118091	-1.50336	79.16495	TRUE	HP_339028	TGCTGTTG	GGTATCTT	XM_139683	Mus musculus similar to helix-destabilizing protein - rat (LOC224379), mRNA.			2.11	SM2352	f		10
092308m3_V2MM_43637	-1.50296	84.31789	TRUE	HP_266210	TGCTGTTG	CTGACAGG	NM_172514	expressed sequence AI661017	AI661017		2.12	SM2402	a		10
mPool6_V2MM_11401	-1.50209	61.94057	TRUE	HP_234813	TGCTGTTG	GAGATGAG	NM_007714	CDC like kinase 4	Clk4		2.7	SM2184	e		3
092308m1_V2MM_77037	-1.50169	3.004939	TRUE	HP_298770	TGCTGTTG	CTCTGTGC	NM_010563	Mus musculus internexin neurol intermediate filament protein, alpha (I), mR.			2.4	SM2025	e		3
mPool4_V2MM_181159	-1.50165	91.71214	TRUE	HP_401633	TGCTGTTG	CACTACAA	XM_288628	Mus musculus LOC328450 (LOC328450), mRNA.		NA				NA	
092308m3_V2MM_225247	-1.50087	84.31789	TRUE	HP_477299	TGCTGTTG	CAAATATT	XM_487526	similar to peptidylprolyl isomerase A			2.13	SM2456	c		6
mPool4_V2MM_154815	-1.49881	92.61223	TRUE	HP_375456	TGCTGTTG	CACACTCT	XM_197030	Mus musculus LOC271091 (LOC271091), mRNA.		NA				NA	
092308m3_V2MM_235032	-1.49851	61.94057	TRUE	HP_486901	TGCTGTTG	TTCAACTC	XM_195334	Mus musculus similar to hypothetical protein C230069C04 [Mus musculus] (LOC2702			2.14	SM2527	b		9
mPool4_V2MM_170940	-1.49811	84.31789	TRUE	HP_391430	TGCTGTTG	GTGCCTCT	XM_286523	gene model 806, (NCBI)	Gm806	NA				NA	
092308m3_V2MM_99591	-1.49585	83.62456	TRUE	HP_320789	TGCTGTTG	CTAGTCTC	NM_010009	cytochrome P450, family 27, subfamily b, polypeptide 1	Cyp27b1		2.13	SM2467	g		5
mPool6_V2MM_190416	-1.49279	94.45693	TRUE	HP_301189	TGCTGTTG	GCATAATT	NM_198415	creatine kinase, mitochondrial 2	Ckmt2		2.7	SM2186	d		2
mPool2_V2MM_38692	-1.48997	3.004939	TRUE	HP_261409	TGCTGTTG	GCCCGGAG	NM_011789	adenomatosis polyposis coli 2	Apc2		2.11	SM2389	f		6
092308m1_V2MM_123348	-1.48953	3.004939	TRUE	HP_344204	TGCTGTTG	GCCTTGAG	XM_488280	similar to Ig heavy chain precursor V region (108A) - mouse			2.6	SM2103	e		12
092308m3_V2MM_236722	-1.4882	70.87591	TRUE	HP_488577	TGCTGTTG	CTGGGAAT	XM_157901	Mus musculus LOC225982 (LOC225982), mR.			2.14	SM2509	e		10
092308m3_V2MM_83145	-1.48512	61.94057	TRUE	HP_304696	TGCTGTTG	CTTCGAGA	BC057080	ATP-binding cassette, sub-family C (CFTR/MRP), member	Abcc8		2.13	SM2460	e		8
092308m1_V2MM_147672	-1.48486	3.004939	TRUE	HP_368383	TGCTGTTG	CAAAGGGA	XM_163172	Mus musculus LOC243916 (LOC243916), mR.			2.4	SM2034	a		6
092308m1_V2MM_147672	-1.48486	3.004939	TRUE	HP_368383	TGCTGTTG	CAAAGGGA	XM_163172	Mus musculus LOC243916 (LOC243916), mRNA.			2.4	SM2034	a		6
mPool7_V2MM_179060	-1.48276	2.577834	TRUE	HP_399534	TGCTGTTG	GCACAACT	XM_288268	Mus musculus LOC332541 (LOC332541), mRNA.			2.9	SM2278	d		2
092308m1_V2MM_131572	-1.48124	3.004939	TRUE	HP_352334	TGCTGTTG	CTTTGAGT	XM_147719	EDAR (ectodysplasin-A receptor)-associated death doma	Edaradd		2.5	SM2099	b		5
092308m3_V2MM_53367	-1.48018	83.62456	TRUE	HP_275730	TGCTGTTG	GTGGCGCT	NM_016718	ninjurin 2	Ninj2		2.12	SM2444	e		3
092308m1_V2MM_185950	-1.47997	3.004939	TRUE	HP_406422	TGCTGTTG	CACCCAGA	XM_289456	Mus musculus LOC333141 (LOC333141), mRNA.			2.5	SM2091	a		11
092308m1_V2MM_185950	-1.47997	3.004939	TRUE	HP_406422	TGCTGTTG	CACCCAGA	XM_289456	Mus musculus LOC333141 (LOC333141), mR.			2.5	SM2091	a		11
mPool4_V2MM_51380	-1.47394	84.31789	TRUE	HP_273780	TGCTGTTG	CTCAGTAA	NM_147107	olfactory receptor 974	Olfr974		2.15	SM2590	f		1
mPool7_V2MM_202787	-1.47141	3.004939	TRUE	HP_420775	TGCTGTTG	CTGCTGTA	XM_126758	keratin complex 1, acidic, gene 5	Krt1-5		2.9	SM2300	c		5
092308m1_V2MM_139512	-1.47102	3.004939	TRUE	HP_360232	TGCTGTTG	GATACTCA	XM_155133	Mus musculus LOC239257 (LOC239257), mRNA.			2.5	SM2058	b		11
092308m1_V2MM_139512	-1.47102	3.004939	TRUE	HP_360232	TGCTGTTG	GATACTCA	XM_155133	Mus musculus LOC239257 (LOC239257), mR.			2.5	SM2058	b		11
mPool4_V2MM_240550	-1.46993	61.94057	TRUE	HP_492331	TGCTGTTG	CATACCTC	XM_148947	Mus musculus LOC212511 (LOC212511), mRNA.			2.14	SM2532	b		8
092308m3_V2MM_234151	-1.46913	87.58579	TRUE	HP_486037	TGCTGTTG	GTTATATC	XM_128873	RIKEN cD 4833446K15 gene	4833446K15Rik		2.13	SM2486	d		11
092308m1_V2MM_66996	-1.46774	3.004939	TRUE	HP_288997	TGCTGTTG	CCCAGGTC	NM_009814	calsequestrin 2	Casq2		2.4	SM2028	c		2
092308m1_V2MM_133568	-1.4664	3.004939	TRUE	HP_354306	TGCTGTTG	CAGTTCAG	XM_149327	RIKEN cD 4930565D16 gene	4930565D16Rik		2.6	SM2101	a		8
mPool4_V2MM_37223	-1.46383	91.71214	TRUE	HP_259987	TGCTGTTG	GACATGGA	NM_144787	jumonji domain containing 2C	Jmjd2c		2.15	SM2593	d		11

092308m3_V2MM_208722	-1.46253	61.94057	TRUE	NA	NA	NA	NA	NA	NA	NA		NA	NA	NA	
092308m3_V2MM_101581	-1.46136	61.94057	TRUE	HP_322749	TGCTGTTG/	GATGTAAC'	XM_127726	Mus musculus RIKEN cD 5830451P18 gene (5830451P18Rik), mR.			2.13	SM2497	f		8
mPool4_V2MM_175055	-1.46101	66.95036	TRUE	HP_395540	TGCTGTTG/	CCTCCATG(	XM_287364	Mus musculus hypothetical gene supported by AK034470 (LOC329914), mRNA.		NA				NA	
092308m3_V2MM_94727	-1.4605	79.16495	TRUE	HP_316011	TGCTGTTG/	CACCTAGC/	NM_183223	hypothetical protein D730018G16			2.13	SM2457	b		8
092308m3_V2MM_107726	-1.45865	81.56557	TRUE	HP_328808	TGCTGTTG/	GCATGCTT(	XM_132286	RIKEN cD 2310005C01 gene	2310005C01Rik		2.13	SM2487	d		11
092308m3_V2MM_56000	-1.45703	61.94057	TRUE	HP_278290	TGCTGTTG/	CGGCTATT1	NM_031867	taste receptor, type 1, member 1	Tas1r1		2.12	SM2438	a		1
mPool7_V2MM_107310	-1.45554	3.004939	TRUE	NA	NA	NA	NA	NA	NA	NA		NA	NA	NA	
mPool2_V2MM_125122	-1.45538	2.577834	TRUE	HP_345966	TGCTGTTG/	CCAATCGA/	NM_143807	Mus musculus similar to alpha-2u-globulin IV precursor - mouse (LOC209155), mRNA.			2.11	SM2370	f		1
092308m3_V2MM_85733	-1.4537	87.58579	TRUE	HP_307214	TGCTGTTG/	CAGACACC'	NM_146453	olfactory receptor 693	Olfr693		2.13	SM2466	c		1
mPool4_V2MM_179954	-1.45351	61.94057	TRUE	HP_400428	TGCTGTTG/	CTTCCCTAT	XM_288425	Unknown (protein for MGC:58818)		NA				NA	
mPool4_V2MM_193883	-1.45134	61.94057	TRUE	HP_7949	TGCTGTTG/	CAGTTCTC(	XM_195649	Spi-B transcription factor (Spi-1/PU.1 related)	Spib	NA				NA	
092308m3_V2MM_39102	-1.44456	70.87591	TRUE	HP_261800	TGCTGTTG/	GCTCAACT/	NM_026972	CD209b antigen	Cd209b		2.12	SM2424	b		9
092308m1_V2MM_139336	-1.44353	3.004939	TRUE	HP_360056	TGCTGTTG/	GACACACT(	XM_154947	Mus musculus LOC211279 (LOC211279), mR.			2.4	SM2034	e		2
092308m1_V2MM_139336	-1.44353	3.004939	TRUE	HP_360056	TGCTGTTG/	GACACACT(	XM_154947	Mus musculus LOC211279 (LOC211279), mRNA.			2.4	SM2034	e		2
mPool7_V2MM_173742	-1.4395	3.004939	TRUE	HP_394228	TGCTGTTG/	CCCAACCC/	XM_287084	Mus musculus LOC329351 (LOC329351), mR.			2.5	SM2063	b		7
mPool7_V2MM_173742	-1.4395	3.004939	TRUE	HP_394228	TGCTGTTG/	CCCAACCC/	XM_287084	Mus musculus LOC329351 (LOC329351), mRNA.			2.5	SM2063	b		7
mPool2_V2MM_131980	-1.43675	3.004939	TRUE	HP_352740	TGCTGTTG/	CTACCCAG(	XM_147934	ATP-binding cassette, sub-family D (ALD), member 2	Abcd2		2.11	SM2369	d		10
mPool7_V2MM_145243	-1.4367	3.004939	TRUE	HP_365954	TGCTGTTG/	CAGGCTTC(	XM_160705	Mus musculus LOC214719 (LOC214719), mRNA.			2.5	SM2052	c		11
mPool7_V2MM_145243	-1.4367	3.004939	TRUE	HP_365954	TGCTGTTG/	CAGGCTTC(	XM_160705	Mus musculus LOC214719 (LOC214719), mR.			2.5	SM2052	c		11
mPool7_V2MM_83310	-1.43126	3.004939	TRUE	HP_304856	TGCTGTTG/	GACGCCAT(	NM_012014	G protein-regulated inducer of neurite outgrowth 1	Gprin1		2.9	SM2258	b		7
092308m1_V2MM_153108	-1.43075	3.004939	TRUE	HP_373769	TGCTGTTG/	GTGTCAAT(	XM_195744	Mus musculus similar to olfactory receptor MOR168-1 [Mus musculus] (LOC270145), ı			2.6	SM2124	b		9
mPool2_V2MM_29618	-1.42881	3.004939	TRUE	HP_252576	TGCTGTTG/	CACGTTCC/	NM_008634	microtubule-associated protein 1 B	Mtap1b		2.11	SM2389	b		9
mPool4_V2MM_94893	-1.42359	61.94057	TRUE	HP_316176	TGCTGTTG/	CAGTCTCTT	NM_183281	RIKEN cDNA 2310005G13 gene	2310005G13Rik		2.16	SM2613	c		12
mPool2_V2MM_134470	-1.42356	3.004939	TRUE	HP_355196	TGCTGTTG/	CCACCTGG(	XM_149892	Mus musculus LOC232948 (LOC232948), mR.			2.6	SM2102	d		5
mPool2_V2MM_134470	-1.42356	3.004939	TRUE	HP_355196	TGCTGTTG/	CCACCTGG(	XM_149892	Mus musculus LOC232948 (LOC232948), mRNA.			2.6	SM2102	d		5
092308m3_V2MM_21318	-1.42149	61.94057	TRUE	HP_244493	TGCTGTTG/	CAGCTATC/	NM_028231	potassium large conductance calcium-activated channel,	Kcnmb2		2.11	SM2399	a		6
mPool2_V2MM_105479	-1.42045	3.004939	TRUE	HP_326594	TGCTGTTG/	CAGTGCAG	XM_130532	ribosome binding protein 1	Rrbp1		2.7	SM2163	f		4
mPool2_V2MM_128296	-1.41817	3.004939	TRUE	HP_349110	TGCTGTTG/	GCAAGGAA	XM_145626	Mus musculus similar to envelope protein [Ovis aries] (LOC233260), mRNA.			2.11	SM2358	c		12
092308m1_V2MM_83373	-1.41801	3.004939	TRUE	HP_304917	TGCTGTTG/	CACAACAG'	NM_013587	low density lipoprotein receptor-related protein associat	Lrpap1		2.6	SM2112	e		6
092308m1_V2MM_83373	-1.41801	3.004939	TRUE	HP_304917	TGCTGTTG/	CACAACAG'	NM_013587	low density lipoprotein receptor-related protein associat	Lrpap1		2.6	SM2112	e		6
mPool2_V2MM_133342	-1.41632	3.004939	TRUE	HP_354082	TGCTGTTG/	CAAGTGGC	NM_207206	expressed sequence AI505034	AI505034		2.11	SM2367	h		5
mPool2_V2MM_130089	-1.41596	3.004939	TRUE	HP_350888	TGCTGTTG/	CTGAAGGG	XM_146606	DNA segment, Chr 8, ERATO Doi 738, expressed	D8Ertd738e		2.11	SM2352	g		11
092308m3_V2MM_44041	-1.41573	94.45693	TRUE	HP_266607	TGCTGTTG/	CTGTGACT1	NM_009645	activation-induced cytidine deamise	Aicda		2.12	SM2412	a		10
092308m1_V2MM_122265	-1.40751	2.577834	TRUE	HP_343136	TGCTGTTG/	GGAAGTAG	XM_142100	similar to RIKEN cD 9230105E10 gene			2.6	SM2105	f		3
mPool7_V2MM_94292	-1.40665	3.004939	TRUE	HP_315584	TGCTGTTG/	GTCCAGAG	NM_183123	RIKEN cDNA 9530002K18 gene	9530002K18Rik		2.6	SM2139	f		1
092308m3_V2MM_23589	-1.4048	94.45693	TRUE	HP_246717	TGCTGTTG/	CTTATGAG/	NM_172475	FERM domain containing 4A	Frmd4a		2.12	SM2424	g		11
mPool4_V2MM_207015	-1.40442	73.80895	TRUE	HP_424830	TGCTGTTG/	GTCCTTCAT	XM_195072	Mus musculus LOC271513 (LOC271513), mRNA.		NA				NA	
mPool6_V2MM_169664	-1.40426	91.71214	TRUE	HP_390159	TGCTGTTG/	CTGAAGAT/	AK039689	hypothetical protein A330087I24			2.7	SM2197	a		1
mPool4_V2MM_167242	-1.40374	94.45693	TRUE	HP_387756	TGCTGTTG/	GAACTATT(	XM_285725	Mus musculus similar to Extracellular calcium-sensing receptor precursor (CaSR) (Para		NA				NA	
092308m1_V2MM_179859	-1.40243	3.004939	TRUE	HP_400333	TGCTGTTG/	GACACTGG	XM_288409	Mus musculus LOC331732 (LOC331732), mR.			2.5	SM2085	g		3
092308m1_V2MM_179859	-1.40243	3.004939	TRUE	HP_400333	TGCTGTTG/	GACACTGG	XM_288409	Mus musculus LOC331732 (LOC331732), mRNA.			2.5	SM2085	g		3
092308m1_V2MM_154726	-1.40196	3.004939	TRUE	HP_375367	TGCTGTTG/	GTGAGAAC	XM_196981	cD sequence BC065123	BC065123		2.6	SM2114	g		10
mPool7_V2MM_81662	-1.39931	3.004939	TRUE	HP_303261	TGCTGTTG/	GTGACACT(	NM_025663	RIKEN cDNA 2610029K21 gene	2610029K21Rik		2.8	SM2244	g		10
mPool2_V2MM_155044	-1.3989	3.004939	TRUE	HP_375685	TGCTGTTG/	CATCTGGG(	XM_197160	Mus musculus LOC271249 (LOC271249), mRNA.			2.6	SM2126	e		12
092308m3_V2MM_27067	-1.39768	84.31789	TRUE	HP_250096	TGCTGTTG/	CTGGTATT(	NM_133862	fibrinogen, gamma polypeptide	Fgg		2.11	SM2393	e		7
mPool4_V2MM_129244	-1.3963	69.72441	TRUE	HP_350055	TGCTGTTG/	CTTACGTC(	XM_146208	RIKEN cDNA A130072A22 gene	A130072A22Rik		2.14	SM2550	b		11
mPool7_V2MM_111439	-1.39518	2.577834	TRUE	HP_332453	TGCTGTTG/	CGAGATCC'	XM_147068	Mus musculus similar to glyceraldehyde-3-phosphate dehydrogenase [Mus musculus]			2.1	SM2304	a		4
092308m3_V2MM_59827	-1.39358	61.94057	TRUE	HP_282000	TGCTGTTG/	GCTGGTTT/	NM_173155	amyotrophic lateral sclerosis 2 (juvenile) chromosome re	Als2cr2		2.12	SM2446	b		4
092308m1_V2MM_154672	-1.39325	3.004939	TRUE	HP_375313	TGCTGTTG/	GTTGACAA'	XM_196964	Mus musculus LOC270974 (LOC270974), mR.			2.6	SM2117	c		1
mPool4_V2MM_124567	-1.39318	75.98381	TRUE	HP_345418	TGCTGTTG/	CTTTGCTCG	XM_143509	Mus musculus LOC242201 (LOC242201), mRNA.			2.14	SM2537	b		1
mPool2_V2MM_98122	-1.39291	3.004939	TRUE	HP_319355	TGCTGTTG/	GACATCAA(	XM_112160	Mus musculus similar to RNP particle component [Mus musculus] (LOC195745), mRN/			2.6	SM2108	d		7
mPool2_V2MM_98122	-1.39291	3.004939	TRUE	HP_319355	TGCTGTTG/	GACATCAA(	XM_112160	Mus musculus similar to RNP particle component [Mus musculus] (LOC195745), mR.			2.6	SM2108	d		7

092308m1_V2MM_156913	-1.39274	3.004939	TRUE	HP_377538	TGCTGTTG/	CATCGCAC/	XM_204568	Mus musculus similar to hypothetical protein FLJ35757 [Homo sapiens] (LOC278367),		2.6	SM2125	f	8
mPool4_V2MM_32654	-1.39156	83.62456	TRUE	HP_255532	TGCTGTTG/	GTCTAAAT(	NM_144802	RIKEN cDNA 2810036L13 gene	2810036L13Rik	2.15	SM2599	g	3
092308m3_V2MM_88803	-1.38574	61.94057	TRUE	HP_310239	TGCTGTTG/	CGTAGAAC	NM_175498	paraneoplastic antigen MA2	Pnma2	2.13	SM2461	a	5
092308m3_V2MM_225072	-1.38506	92.61223	TRUE	HP_477127	TGCTGTTG/	CTATAAGT(	NM_011712	WW domain binding protein 5	Wbp5	2.13	SM2485	c	8
mPool2_V2MM_154724	-1.38502	3.004939	TRUE	HP_375365	TGCTGTTG/	GAGAGACA	XM_196981	cDNA sequence BC065123	BC065123	2.6	SM2127	d	5
mPool2_V2MM_118498	-1.38059	3.004939	TRUE	HP_339432	TGCTGTTG/	CTGCCAGA(	XM_139878	similar to ZFP421		2.11	SM2374	h	1
mPool6_V2MM_158432	-1.37853	61.94057	TRUE	HP_379042	TGCTGTTG/	CTTGCCAA(	XM_206630	Mus musculus LOC279600 (LOC279600), mRNA.		2.7	SM2190	e	8
092308m3_V2MM_225897	-1.37735	70.87591	TRUE	HP_477936	TGCTGTTG/	CCGTTGTT(	NM_175327	RIKEN cD B630019K06 gene	B630019K06Rik	2.13	SM2483	e	7
mPool7_V2MM_170934	-1.3767	3.004939	TRUE	HP_391424	TGCTGTTG/	CCAAGCAG	AK079388	RIKEN cDNA D130012G24 gene	D130012G24Rik	2.9	SM2284	c	2
092308m3_V2MM_101489	-1.37251	94.45693	TRUE	HP_322660	TGCTGTTG/	GGAAGCAA	XM_127665	RIKEN cDNA 9230112D13 gene	9230112D13Rik	2.1	SM2305	a	9
092308m3_V2MM_101489	-1.37251	94.45693	TRUE	HP_322660	TGCTGTTG/	GGAAGCAA	XM_127665	RIKEN cD 9230112D13 gene	9230112D13Rik	2.1	SM2305	a	9
mPool2_V2MM_215149	-1.3689	2.577834	TRUE	HP_432710	TGCTGTTG/	GCCACATA/	XM_142310	Mus musculus similar to helix-destabilizing protein - rat (LOC237236), mRNA.		2.11	SM2369	d	7
mPool2_V2MM_204126	-1.36833	3.004939	TRUE	HP_422038	TGCTGTTG/	CAGCCGCC,	BC020182	RIKEN cDNA 2700082O15 gene	2700082O15Rik	2.1	SM2316	b	11
092308m3_V2MM_42370	-1.36728	66.95036	TRUE	HP_264977	TGCTGTTG/	CCAATAGT(	NM_016804	metaxin 2	Mtx2	2.12	SM2404	a	3
mPool7_V2MM_101678	-1.36617	3.004939	TRUE	HP_322844	TGCTGTTG/	CAGAGACT.	XM_127737	RIKEN cDNA 1300010F03 gene	1300010F03Rik	2.9	SM2299	a	11
mPool7_V2MM_101678	-1.36617	3.004939	TRUE	HP_322844	TGCTGTTG/	CAGAGACT.	XM_127737	RIKEN cD 1300010F03 gene	1300010F03Rik	2.9	SM2299	a	11
mPool4_V2MM_82725	-1.36544	83.62456	TRUE	HP_304289	TGCTGTTG/	GGATTCGA.	NM_010236	folylpolyglutamyl synthetase	Fpgs	2.15	SM2571	c	8
mPool6_V2MM_159484	-1.36379	61.94057	TRUE	HP_380077	TGCTGTTG/	GTTCTAGC(	XM_283130	Mus musculus hypothetical gene supported by AK044328 (LOC328229), mRNA.		2.7	SM2195	c	8
092308m1_V2MM_99611	-1.36241	3.004939	TRUE	HP_320809	TGCTGTTG/	GGCACATT(	XM_125928	glutamise 2 (liver, mitochondrial)	Gls2	2.6	SM2107	a	1
mPool4_V2MM_187384	-1.36142	61.94057	TRUE	HP_407853	TGCTGTTG/	CAGACAAA	XM_289748	Mus musculus LOC333655 (LOC333655), mRNA.		NA			NA
mPool7_V2MM_173741	-1.36138	3.004939	TRUE	HP_394227	TGCTGTTG/	CCAACCCA/	XM_287084	Mus musculus LOC329351 (LOC329351), mR.		2.5	SM2063	g	11
mPool7_V2MM_173741	-1.36138	3.004939	TRUE	HP_394227	TGCTGTTG/	CCAACCCA/	XM_287084	Mus musculus LOC329351 (LOC329351), mRNA.		2.5	SM2063	g	11
092308m3_V2MM_98954	-1.36056	94.45693	TRUE	HP_320171	TGCTGTTG/	CAGTGCTT(	XM_125055	similar to RIKEN cD 2610524H06 gene		2.13	SM2454	a	7
092308m3_V2MM_42986	-1.35869	92.61223	TRUE	HP_265586	TGCTGTTG/	CGGTCGAA	NM_172597	RIKEN cD 5730420B22 gene	5730420B22Rik	2.12	SM2437	c	1
092308m1_V2MM_185518	-1.3564	3.004939	TRUE	HP_405990	TGCTGTTG/	GACATCCT(	XM_289384	Mus musculus LOC333052 (LOC333052), mR.		2.5	SM2064	g	8
092308m3_V2MM_146213	-1.35602	84.31789	TRUE	HP_366924	TGCTGTTG/	CCAAGAAC	XM_161832	Mus musculus LOC243054 (LOC243054), mR.		2.14	SM2518	c	9
mPool6_V2MM_157770	-1.35372	61.94057	TRUE	HP_378383	TGCTGTTG/	GAGGTAGC	XM_205592	gene model 725, (NCBI)	Gm725	2.7	SM2200	h	9
mPool4_V2MM_180763	-1.35284	61.94057	TRUE	HP_401237	TGCTGTTG/	CCAAGTCC(	XM_288561	Mus musculus LOC331947 (LOC331947), mRNA.		NA			NA
092308m1_V2MM_142390	-1.35274	3.004939	TRUE	HP_363103	TGCTGTTG/	GCCAGAAG	XM_157619	Mus musculus LOC212132 (LOC212132), mRNA.		2.5	SM2053	c	11
092308m1_V2MM_142390	-1.35274	3.004939	TRUE	HP_363103	TGCTGTTG/	GCCAGAAG	XM_157619	Mus musculus LOC212132 (LOC212132), mR.		2.5	SM2053	c	11
mPool4_V2MM_238628	-1.35262	94.45693	TRUE	HP_490456	TGCTGTTG/	CAAGAATC	XM_144237	Mus musculus similar to RIKEN cDNA 4933431D05 [Mus musculus] (LOC211320), mRN		2.14	SM2533	a	4
mPool4_V2MM_163605	-1.34703	81.56557	TRUE	HP_384160	TGCTGTTG/	CAATCACA(	XM_284820	Mus musculus similar to preferentially expressed antigen in melanoma; melanoma an		NA			NA
092308m3_V2MM_44066	-1.34618	79.16495	TRUE	HP_266632	TGCTGTTG/	CTGTGGCC(	U83509	angiopoietin 1	Angpt1	2.12	SM2445	f	9
mPool4_V2MM_120385	-1.34548	73.80895	TRUE	HP_341283	TGCTGTTG/	CATCATCA(	XM_140891	Mus musculus similar to KIAA1422 protein [Homo sapiens] (LOC241281), mRNA.		2.14	SM2531	g	4
mPool7_V2MM_105908	-1.34458	3.004939	TRUE	HP_327015	TGCTGTTG/	GATGCACT	XM_130859	progesterone receptor membrane component 2	Pgrmc2	2.1	SM2306	e	12
mPool2_V2MM_13680	-1.34365	3.004939	TRUE	HP_237045	TGCTGTTG/	CCACCGAA,	NM_026416	S100 calcium binding protein A16	S100a16	2.11	SM2374	d	9
mPool2_V2MM_89835	-1.34335	3.004939	TRUE	HP_311263	TGCTGTTG/	CTCCTTACC	BC048817	RIKEN cDNA A730098D12 gene	A730098D12Rik	2.6	SM2138	c	2
mPool7_V2MM_208064	-1.34097	3.004939	TRUE	HP_425860	TGCTGTTG/	CCTGCTCT(	XM_484075	hypothetical protein B230105J10		2.1	SM2325	h	12
mPool4_V2MM_112349	-1.33971	94.45693	TRUE	HP_333354	TGCTGTTG/	CTGCCCATT	XM_136437	Mus musculus similar to pol protein (LOC226335), mRNA.		2.16	SM2622	d	1
092308m1_V2MM_66675	-1.33916	3.004939	TRUE	HP_288686	TGCTGTTG/	CATCTGGA(	NM_176843	RIKEN cD 1110055N21 gene	1110055N21Rik	2.4	SM2025	g	1
mPool4_V2MM_123074	-1.33868	91.71214	TRUE	HP_343930	TGCTGTTG/	GTGTTCTA(	XM_485478	similar to RIKEN cDNA 1700029I01		2.14	SM2540	f	7
092308m1_V2MM_173741	-1.33731	3.004939	TRUE	HP_394227	TGCTGTTG/	CCAACCCA/	XM_287084	Mus musculus LOC329351 (LOC329351), mR.		2.5	SM2063	g	11
092308m1_V2MM_173741	-1.33731	3.004939	TRUE	HP_394227	TGCTGTTG/	CCAACCCA/	XM_287084	Mus musculus LOC329351 (LOC329351), mRNA.		2.5	SM2063	g	11
092308m3_V2MM_38253	-1.33723	91.71214	TRUE	HP_260982	TGCTGTTG/	GATTTCATA	NM_172310	RIKEN cD A530046H20 gene	A530046H20Rik	2.11	SM2400	a	9
mPool4_V2MM_47319	-1.33562	70.87591	TRUE	HP_269803	TGCTGTTG/	CTCTTATTC	AK083728	RIKEN cDNA 2410004I17 gene	2410004I17Rik	2.16	SM2609	a	5
092308m1_V2MM_66766	-1.33537	3.004939	TRUE	HP_288774	TGCTGTTG/	CCAAGAGC	BC039216	CLIP associating protein 1	Clasp1	2.4	SM2023	g	7
mPool2_V2MM_117196	-1.33516	3.004939	TRUE	HP_338143	TGCTGTTG/	CAAGCACT(	XM_139255	Mus musculus similar to Eukaryotic translation initiation factor 4B (eIF-4B) (LOC22321		2.7	SM2156	b	12
092308m3_V2MM_85604	-1.32808	61.94057	TRUE	HP_307085	TGCTGTTG/	CAATATTCT	NM_146423	olfactory receptor 887	Olfr887	2.13	SM2457	c	5
092308m1_V2MM_167114	-1.32572	3.004939	TRUE	HP_387628	TGCTGTTG/	CTAATGTT(	XM_285698	similar to meltrin alpha		2.6	SM2116	b	9
mPool6_V2MM_25178	-1.32393	79.16495	TRUE	HP_248251	TGCTGTTG/	CAATTGAA(	NM_133729	RIKEN cDNA 2610018G03 gene	2610018G03Rik	2.7	SM2182	a	4
092308m1_V2MM_175119	-1.32324	3.004939	TRUE	HP_395604	TGCTGTTG/	CAGTTCAC/	AK045802	CUB and Sushi multiple domains 2	Csmd2	2.5	SM2082	a	7
092308m1_V2MM_175119	-1.32324	3.004939	TRUE	HP_395604	TGCTGTTG/	CAGTTCAC/	AK045802	CUB and Sushi multiple domains 2	Csmd2	2.5	SM2082	a	7

mPool6_V2MM_153077	-1.32323	91.71214	TRUE	HP_373738	TGCTGTTG/	GCCTCTATC	XM_195727	Mus musculus similar to ribosomal protein S24 [Rattus norvegicus] (LOC270093), mRN			2.7	SM2191	a		1
092308m3_V2MM_205209	-1.31904	83.62456	TRUE	HP_423082	TGCTGTTG/	GGCAACGT	XM_137847	type I hair keratin KA36			2.13	SM2486	a		9
mPool4_V2MM_160039	-1.3186	91.10404	TRUE	HP_380624	TGCTGTTG/	CACTCAATC	AK028773	gene model 1600, (NCBI)	Gm1600	NA				NA	
mPool4_V2MM_5128	-1.31635	84.31789	TRUE	HP_228687	TGCTGTTG/	CATGTATCT	NM_019482	pannexin 1	Panx1		2.15	SM2569	b		4
mPool4_V2MM_1623	-1.31557	83.62456	TRUE	HP_225262	TGCTGTTG/	CGAATTATC	NM_025896	prolactin like protein I			2.15	SM2579	a		9
mPool4_V2MM_241330	-1.31555	66.95036	TRUE	HP_493091	TGCTGTTG/	GGTCAAAT/	NM_199020	cDNA sequence BC057593	BC057593		2.14	SM2543	h		5
mPool6_V2MM_81833	-1.3138	94.45693	TRUE	HP_303424	TGCTGTTG/	GTGGATAT	NM_172926	sorting nexin 14	Snx14		2.8	SM2218	f		12
092308m1_V2MM_134027	-1.31181	2.577834	TRUE	HP_354757	TGCTGTTG/	CAAGTAAG.	XM_486176	LOC434363			2.6	SM2104	d		11
092308m3_V2MM_91675	-1.31032	70.87591	TRUE	HP_313044	TGCTGTTG/	CTGGGTAT(	NM_178402	RIKEN cD 5330426L24 gene	5330426L24Rik		2.9	SM2262	g		5
092308m3_V2MM_91675	-1.31032	70.87591	TRUE	HP_313044	TGCTGTTG/	CTGGGTAT(	NM_178402	RIKEN cDNA 5330426L24 gene	5330426L24Rik		2.9	SM2262	g		5
mPool7_V2MM_184309	-1.30813	2.577834	TRUE	HP_404781	TGCTGTTG/	GCTATCAG/	XM_289176	Mus musculus LOC332799 (LOC332799), mRNA.			2.5	SM2077	a		5
mPool7_V2MM_184309	-1.30813	2.577834	TRUE	HP_404781	TGCTGTTG/	GCTATCAG/	XM_289176	Mus musculus LOC332799 (LOC332799), mR.			2.5	SM2077	a		5
092308m3_V2MM_194627	-1.30799	83.62456	TRUE	HP_412784	TGCTGTTG/	CTCAGTGT(	NM_175331	RIKEN cD C630002B14 gene	C630002B14Rik		2.12	SM2449	c		9
mPool4_V2MM_262180	-1.30529	83.62456	TRUE	HP_523965	TGCTGTTG/	CATACCAT/	NM_175690	RIKEN cDNA A730027B03 gene	A730027B03Rik		2.16	SM2617	d		5
mPool4_V2MM_178783	-1.30516	61.94057	TRUE	HP_399257	TGCTGTTG/	GGCACTAC	XM_288219	Mus musculus LOC332488 (LOC332488), mRNA.		NA				NA	
mPool6_V2MM_162762	-1.30423	61.94057	TRUE	HP_383320	TGCTGTTG/	CAACATGA/	XM_486210	similar to PIP-1 protein precursor			2.8	SM2210	a		6
mPool4_V2MM_174556	-1.30319	92.96229	TRUE	HP_395041	TGCTGTTG/	CATATTCCT	XM_287252	Mus musculus hypothetical gene supported by AK084244 (LOC329673), mRNA.		NA				NA	
mPool4_V2MM_126785	-1.30246	83.62456	TRUE	HP_347625	TGCTGTTG/	GTCACTCC/	XM_144802	similar to immunoglobulin light chain variable region			2.14	SM2545	f		1
mPool4_V2MM_197560	-1.30223	92.61223	TRUE	HP_415628	TGCTGTTG/	CTCATTTAC	NM_207155	olfactory receptor 117	Olfr117		2.15	SM2575	a		12
092308m3_V2MM_83906	-1.3021	61.94057	TRUE	HP_305428	TGCTGTTG/	CACTATTTC	NM_026185	RIKEN cD 1300007F04 gene	1300007F04Rik		2.13	SM2464	c		2
092308m3_V2MM_54623	-1.3016	81.56557	TRUE	HP_276946	TGCTGTTG/	CCAACATTC	NM_146869	olfactory receptor 147	Olfr147		2.12	SM2446	b		8
092308m1_V2MM_157571	-1.29801	3.004939	TRUE	HP_378189	TGCTGTTG/	GCCTTCGA(	AY244804	synovial sarcoma, X member A, breakpoint 1	Ssxa1		2.6	SM2118	f		8
mPool4_V2MM_58227	-1.29753	84.31789	TRUE	HP_280459	TGCTGTTG/	GAGATACA	NM_138591	G elongation factor	Gfm		2.16	SM2603	a		12
mPool7_V2MM_185950	-1.29746	2.577834	TRUE	HP_406422	TGCTGTTG/	CACCCAGA/	XM_289456	Mus musculus LOC333141 (LOC333141), mR.			2.5	SM2091	a		11
mPool7_V2MM_185950	-1.29746	2.577834	TRUE	HP_406422	TGCTGTTG/	CACCCAGA/	XM_289456	Mus musculus LOC333141 (LOC333141), mRNA.			2.5	SM2091	a		11
mPool6_V2MM_161166	-1.29506	61.94057	TRUE	HP_381738	TGCTGTTG/	GACCTCCA(	AK047497	transmembrane 9 superfamily protein member 4	Tm9sf4		2.7	SM2197	h		8
mPool2_V2MM_124526	-1.2948	3.004939	TRUE	HP_345378	TGCTGTTG/	CAGTCAGT(	XM_143496	Mus musculus similar to Ribonucleoside-diphosphate reductase M2 chain (Ribonuclec			2.11	SM2373	b		3
092308m1_V2MM_157567	-1.29479	3.004939	TRUE	HP_378185	TGCTGTTG/	CCTTCGAG(	AY244804	synovial sarcoma, X member A, breakpoint 1	Ssxa1		2.6	SM2121	f		12
mPool6_V2MM_81574	-1.29099	61.94057	TRUE	HP_303175	TGCTGTTG/	GTCTAGTA/	XM_355775	similar to Ig V-K167 precursor			2.8	SM2225	g		1
mPool2_V2MM_6071	-1.29085	3.004939	TRUE	HP_229612	TGCTGTTG/	CTATGAGG.	NM_023120	guanine nucleotide binding protein (G protein), beta poly	Gnb1l		2.11	SM2379	g		1
mPool4_V2MM_24963	-1.28929	61.94057	TRUE	HP_248047	TGCTGTTG/	GTGCATAA(	NM_146022	hypothetical protein MGC7717			2.15	SM2586	c		9
mPool4_V2MM_166206	-1.28687	61.94057	TRUE	HP_386736	TGCTGTTG/	GAAACATC.	XM_488341	similar to glyceraldehyde-3-phosphate dehydrogenase		NA				NA	
mPool2_V2MM_149737	-1.28442	3.004939	TRUE	HP_370447	TGCTGTTG/	CAGCACAT(	XM_165151	similar to 60S ribosomal protein L5			2.4	SM2039	c		7
mPool2_V2MM_149737	-1.28442	3.004939	TRUE	HP_370447	TGCTGTTG/	CAGCACAT(	XM_165151	similar to 60S ribosomal protein L5			2.4	SM2039	c		7
mPool4_V2MM_48544	-1.28018	61.94057	TRUE	HP_271003	TGCTGTTG/	GCACAGAA	XM_485408	RIKEN cDNA A330015D16 gene	A330015D16Rik		2.16	SM2610	g		4
092308m3_V2MM_207901	-1.27975	92.61223	TRUE	HP_425698	TGCTGTTG/	CGGGACTC	BC075693	RIKEN cD 1190002H09 gene	1190002H09Rik		2.14	SM2524	c		1
mPool4_V2MM_167810	-1.27811	73.80895	TRUE	HP_388322	TGCTGTTG/	CAAAGCAA	XM_285844	Mus musculus similar to MAP2 RNA trans-acting protein MARTA1 [Rattus norvegicus]		NA				NA	
mPool4_V2MM_129056	-1.27586	70.87591	TRUE	HP_349869	TGCTGTTG/	CTGTCCTCT	XM_146110	Mus musculus LOC211996 (LOC211996), mRNA.			2.16	SM2635	f		1
mPool4_V2MM_109499	-1.27258	61.94057	TRUE	HP_330553	TGCTGTTG/	CGCTAAAC(	XM_133916	leucine rich repeat containing 27	Lrrc27		2.16	SM2620	a		5
mPool4_V2MM_2614	-1.27149	83.62456	TRUE	HP_226227	TGCTGTTG/	CTGATCCT/	AY462059	kininogen 2	Kng2		2.15	SM2564	c		9
092308m3_V2MM_226824	-1.26976	91.71214	TRUE	HP_478833	TGCTGTTG/	CTGAAGAG	NM_008296	heat shock factor 1	Hsf1		2.13	SM2469	f		6
092308m1_V2MM_175115	-1.26835	3.004939	TRUE	HP_395600	TGCTGTTG/	CTTGGGCC/	XM_287375	hypothetical protein 9630027E11			2.5	SM2092	a		10
092308m1_V2MM_184309	-1.26779	2.577834	TRUE	HP_404781	TGCTGTTG/	GCTATCAG/	XM_289176	Mus musculus LOC332799 (LOC332799), mRNA.			2.5	SM2077	a		5
092308m1_V2MM_184309	-1.26779	2.577834	TRUE	HP_404781	TGCTGTTG/	GCTATCAG/	XM_289176	Mus musculus LOC332799 (LOC332799), mR.			2.5	SM2077	a		5
092308m3_V2MM_142716	-1.26747	70.87591	TRUE	HP_363429	TGCTGTTG/	GTGTAGAC	XM_157855	Mus musculus LOC240627 (LOC240627), mR.			2.14	SM2528	e		12
mPool4_V2MM_35512	-1.26449	70.87591	TRUE	HP_258305	TGCTGTTG/	CGTTGTTC/	NM_146096	RIKEN cDNA 5730446C15 gene	5730446C15Rik		2.15	SM2593	c		4
092308m1_V2MM_178676	-1.26448	3.004939	TRUE	HP_399150	TGCTGTTG/	GAGCACAA	XM_288201	Mus musculus LOC332471 (LOC332471), mR.			2.5	SM2084	c		6
mPool7_V2MM_68690	-1.26221	3.004939	TRUE	HP_290640	TGCTGTTG/	GACTGTAA	NM_053144	protocadherin beta 19	Pcdhb19		2.8	SM2241	a		8
092308m3_V2MM_235893	-1.25921	61.94057	TRUE	HP_487756	TGCTGTTG/	CGGCAAGC	XM_159076	Mus musculus LOC241849 (LOC241849), mR.			2.14	SM2522	c		6
mPool5_V2MM_161755	-1.25886	61.94057	TRUE	HP_382319	TGCTGTTG/	GGTACAGG	XM_284033	gene model 1369, (NCBI)	Gm1369	NA				NA	
mPool4_V2MM_4938	-1.25854	79.16495	TRUE	HP_228500	TGCTGTTG/	CAGCTCAC	NM_025907	RIKEN cDNA 1600013P15 gene	1600013P15Rik		2.16	SM2602	f		2
mPool2_V2MM_88981	-1.25801	3.004939	TRUE	HP_310415	TGCTGTTG/	GGCGAAAG	AK048755	zinc finger protein 291	Zfp291		2.6	SM2109	f		6

mPool2_V2MM_88981	-1.25801	3.004939	TRUE	HP_310415	TGCTGTTG/	GGCGAAAG	AK048755	zinc finger protein 291	Zfp291		2.6	SM2109	f		6
092308m1_V2MM_134469	-1.25749	3.004939	TRUE	HP_355195	TGCTGTTG/	CACCTGGG	XM_149892	Mus musculus LOC232948 (LOC232948), mR.			2.6	SM2111	c		4
092308m1_V2MM_134469	-1.25749	3.004939	TRUE	HP_355195	TGCTGTTG/	CACCTGGG	XM_149892	Mus musculus LOC232948 (LOC232948), mRNA.			2.6	SM2111	c		4
mPool4_V2MM_158562	-1.25548	73.80895	TRUE	HP_379172	TGCTGTTG/	CCCATGGA	XM_206765	Mus musculus LOC279190 (LOC279190), mRNA.		NA				NA	
092308m3_V2MM_25777	-1.25536	94.45693	TRUE	HP_248836	TGCTGTTG/	CCACTTCTC	NM_145406	solute carrier family 10 (sodium/bile acid cotransporter f	Slc10a3		2.12	SM2430	a		11
mPool4_V2MM_34194	-1.25299	91.10404	TRUE	HP_257021	TGCTGTTG/	CCAGTATA	XM_138190	Mus musculus similar to phosphoglycerate kinase 1 [Mus musculus] (LOC217777), mR			2.16	SM2606	g		6
092308m1_V2MM_159114	-1.25213	3.004939	TRUE	NA	NA	NA	NA	NA	NA	NA		NA	NA	NA	
092308m3_V2MM_238159	-1.25136	61.94057	TRUE	HP_489997	TGCTGTTG/	CAGACAGC	XM_488917	RIKEN cD 4930532M18 gene	4930532M18Rik		2.14	SM2528	b		2
mPool6_V2MM_222819	-1.24963	61.94057	TRUE	HP_475047	TGCTGTTG/	GAGATCAC	XM_195501	CDK5 regulatory subunit associated protein 2	Cdk5rap2		2.8	SM2208	d		6
mPool4_V2MM_47189	-1.24858	94.45693	TRUE	HP_269676	TGCTGTTG/	CTCATTCAC	NM_032398	plasmalemma vesicle associated protein	Plvap		2.15	SM2580	f		10
092308m3_V2MM_225052	-1.24683	94.45693	TRUE	HP_477107	TGCTGTTG/	CACTCAAT	NM_146616	olfactory receptor 305	Olfr305		2.13	SM2470	b		11
mPool4_V2MM_141668	-1.24671	94.45693	TRUE	HP_362381	TGCTGTTG/	GTTTGGAT	XM_156858	Mus musculus similar to pol protein [Phascolarctos cinereus] (LOC215430), mRNA.			2.1	SM2343	g		1
mPool6_V2MM_223433	-1.24335	91.10404	TRUE	HP_475590	TGCTGTTG/	CGCTTTCTC	AK122512	RIKEN cDNA D630023B12 gene	D630023B12Rik		2.8	SM2211	d		6
092308m3_V2MM_136886	-1.23916	94.45693	TRUE	HP_357606	TGCTGTTG/	CTCCTCTCC	XM_152202	Mus musculus LOC237477 (LOC237477), mR.			2.14	SM2524	b		11
mPool6_V2MM_31499	-1.2387	92.96229	TRUE	HP_254400	TGCTGTTG/	CTGTCTCCA	NM_145508	dual-specificity tyrosine-(Y)-phosphorylation regulated ki	Dyrk3		2.7	SM2184	a		11
mPool4_V2MM_127985	-1.23817	84.31789	TRUE	HP_348803	TGCTGTTG/	CTGATGTC	XM_145478	Mus musculus similar to endogenous retroviral family W, env(C7), member 1 (syncytir			2.11	SM2356	d		12
mPool2_V2MM_148226	-1.23617	3.004939	TRUE	HP_368937	TGCTGTTG/	GATAGCAA	XM_163616	Mus musculus LOC210679 (LOC210679), mRNA.			2.4	SM2037	h		5
mPool2_V2MM_148226	-1.23617	3.004939	TRUE	HP_368937	TGCTGTTG/	GATAGCAA	XM_163616	Mus musculus LOC210679 (LOC210679), mR.			2.4	SM2037	h		5
mPool6_V2MM_70875	-1.23603	84.31789	TRUE	HP_292776	TGCTGTTG/	CAGCCTCT	NM_011177	protease, serine, 18	Prss18		2.8	SM2220	a		2
mPool4_V2MM_262024	-1.23592	61.94057	TRUE	HP_522386	TGCTGTTG/	CTCTCTTCG	NM_053134	protocadherin beta 9	Pcdhb9		2.15	SM2586	g		7
092308m1_V2MM_172467	-1.23288	3.004939	TRUE	HP_392953	TGCTGTTG/	GCCTCTGT	XM_286829	similar to hypothetical protein			2.5	SM2079	h		5
mPool4_V2MM_65023	-1.23271	61.94057	TRUE	HP_287070	TGCTGTTG/	GAGATGAG	NM_010666	keratin complex-1, acidic, gene C29	Krt1-c29		2.15	SM2563	a		10
mPool2_V2MM_122154	-1.23154	3.004939	TRUE	HP_343026	TGCTGTTG/	GATGCCAT	XM_142059	Mus musculus similar to Ubiquitin-conjugating enzyme E2 C (Ubiquitin-protein ligase (			2.11	SM2365	h		3
mPool2_V2MM_20872	-1.23072	3.004939	TRUE	HP_244057	TGCTGTTG/	CACATTCCA	NM_008714	Notch gene homolog 1 (Drosophila)	Notch1		2.7	SM2165	g		2
mPool4_V2MM_240500	-1.22852	73.80895	TRUE	HP_492281	TGCTGTTG/	CATTGGCT	XM_145915	RIKEN cDNA 9230119C12 gene	9230119C12Rik		2.14	SM2542	e		3
mPool7_V2MM_98857	-1.22618	3.004939	TRUE	HP_320083	TGCTGTTG/	GAGAACAT	XM_113175	Mus musculus LOC195639 (LOC195639), mRNA.			2.9	SM2262	g		11
mPool6_V2MM_62727	-1.22579	91.71214	TRUE	HP_284825	TGCTGTTG/	CATATTACA	NM_146981	olfactory receptor 1260	Olfr1260		2.8	SM2222	e		7
mPool6_V2MM_161871	-1.22533	69.72441	TRUE	HP_382435	TGCTGTTG/	GAACTAAT	AK005913	expressed sequence AW538212	AW538212		2.7	SM2200	e		8
mPool6_V2MM_79192	-1.22349	61.94057	TRUE	HP_300860	TGCTGTTG/	GATCTTCTA	NM_007402	a disintegrin and metalloprotease domain 7	Adam7		2.8	SM2225	g		7
mPool6_V2MM_71365	-1.22318	61.94057	TRUE	HP_293256	TGCTGTTG/	CGATAGAG	AK086463	RIKEN cDNA D930030K17 gene	D930030K17Rik		2.8	SM2222	f		6
mPool4_V2MM_196233	-1.22266	83.62456	TRUE	NA	NA	NA	NA	NA	NA	NA		NA	NA	NA	
092308m3_V2MM_87937	-1.22179	81.56557	TRUE	HP_309384	TGCTGTTG/	GTCTGACC	NM_175276	formin-family protein FHOS2			2.13	SM2468	a		6
092308m3_V2MM_49774	-1.22113	69.72441	TRUE	HP_272212	TGCTGTTG/	CAAATATC	NM_022032	PERP, TP53 apoptosis effector	Perp		2.12	SM2407	h		2
mPool4_V2MM_261891	-1.21976	73.80895	TRUE	HP_523461	TGCTGTTG/	CTAACATG/	NM_172648	interferon activated gene 205	Ifi205		2.15	SM2600	f		12
mPool6_V2MM_68880	-1.21934	79.16495	TRUE	HP_290826	TGCTGTTG/	GAGGATAA	NM_172880	hypothetical protein A030012E10			2.8	SM2216	h		6
mPool6_V2MM_160030	-1.2187	69.72441	TRUE	HP_380620	TGCTGTTG/	CTCCGACT	NM_177093	RIKEN cDNA C330018J07 gene	C330018J07Rik		2.8	SM2204	c		9
mPool4_V2MM_199187	-1.21864	83.62456	TRUE	HP_417239	TGCTGTTG/	CCTTAAGG	XM_289196	Mus musculus LOC332829 (LOC332829), mRNA.		NA				NA	
mPool4_V2MM_221128	-1.21826	66.95036	TRUE	HP_454066	TGCTGTTG/	CAGTGGTC	NM_146793	olfactory receptor 1271	Olfr1271		2.16	SM2603	e		4
092308m1_V2MM_141850	-1.2165	3.004939	TRUE	HP_362563	TGCTGTTG/	GGTCTAGG	XM_157056	Mus musculus LOC209953 (LOC209953), mRNA.			2.4	SM2048	d		11
092308m1_V2MM_141850	-1.2165	3.004939	TRUE	HP_362563	TGCTGTTG/	GGTCTAGG	XM_157056	Mus musculus LOC209953 (LOC209953), mR.			2.4	SM2048	d		11
092308m1_V2MM_159933	-1.21588	3.004939	TRUE	HP_380526	TGCTGTTG/	CACTGAGG	NM_001002782	RIKEN cD F830031D20 gene	F830031D20Rik		2.6	SM2125	d		10
mPool4_V2MM_166290	-1.21491	91.71214	TRUE	HP_386820	TGCTGTTG/	CTGCTATT	XM_285465	Mus musculus similar to 40S ribosomal protein SA (P40) (34/67 kDa laminin receptor)			2.16	SM2649	b		1
mPool5_V2MM_11368	-1.21475	73.80895	TRUE	HP_234780	TGCTGTTG/	GAGAAGCA	NM_145157	defensin beta 19	Defb19	NA				NA	
mPool4_V2MM_164633	-1.21413	70.87591	TRUE	HP_385176	TGCTGTTG/	GATCTAAC	XM_285055	Mus musculus similar to ribosomal protein L7a, cytosolic [validated] - rat (LOC328294		NA				NA	
mPool7_V2MM_107175	-1.21399	3.004939	TRUE	HP_262574	TGCTGTTG/	GGCTCTCA	XM_355569	similar to Speer1-ps1 protein			2.1	SM2317	h		5
mPool4_V2MM_210878	-1.21085	75.98381	TRUE	HP_428613	TGCTGTTG/	CCGGCTCA	XM_357715	gene model 1424, (NCBI)	Gm1424		2.14	SM2540	e		12
mPool2_V2MM_83373	-1.20955	3.004939	TRUE	HP_304917	TGCTGTTG/	CACAACAG	NM_013587	low density lipoprotein receptor-related protein associat	Lrpap1		2.6	SM2112	e		6
mPool2_V2MM_83373	-1.20955	3.004939	TRUE	HP_304917	TGCTGTTG/	CACAACAG	NM_013587	low density lipoprotein receptor-related protein associat	Lrpap1		2.6	SM2112	e		6
092308m3_V2MM_32588	-1.2091	61.94057	TRUE	HP_255466	TGCTGTTG/	GTAGTTGA	NM_027403	pregncy-specific glycoprotein 21	Psg21		2.12	SM2419	f		5
092308m1_V2MM_179082	-1.2067	3.004939	TRUE	HP_399556	TGCTGTTG/	CGTAGGCT	XM_288271	Mus musculus LOC332544 (LOC332544), mR.			2.5	SM2079	c		6
mPool4_V2MM_5226	-1.20568	61.94057	TRUE	HP_228783	TGCTGTTG/	CCACTCAC	NM_175224	methionyl aminopeptidase 1	Metap1		2.16	SM2618	d		11
mPool4_V2MM_28887	-1.20394	84.31789	TRUE	HP_251862	TGCTGTTG/	GTCATGGA	NM_144863	WD repeat domain 36	Wdr36		2.15	SM2587	h		8

mPool6_V2MM_168164	-1.20275	70.87591	TRUE	HP_388671	TGCTGTTG/	CATCTAGT/	XM_285921	Mus musculus similar to hypothetical protein FLJ11159 [Homo sapiens] (LOC330284),			2.7	SM2198	a		11
mPool4_V2MM_152299	-1.20233	87.58579	TRUE	HP_372962	TGCTGTTG/	CAGCTAGC/	XM_195255	gene model 650, (NCBI)	Gm650	NA				NA	
092308m3_V2MM_91443	-1.20181	81.56557	TRUE	HP_247863	TGCTGTTG/	GGTATAAA	NM_178266	membrane-bound transcription factor protease, site 2	Mbtps2		2.13	SM2483	f		3
092308m3_V2MM_49690	-1.20067	61.94057	TRUE	HP_272128	TGCTGTTG/	GAGATATT	NM_020597	beta-microseminoprotein	Msmb		2.12	SM2407	f		8
092308m1_V2MM_134470	-1.2004	2.577834	TRUE	HP_355196	TGCTGTTG/	CCACCTGG(	XM_149892	Mus musculus LOC232948 (LOC232948), mRNA.			2.6	SM2102	d		5
092308m1_V2MM_134470	-1.2004	2.577834	TRUE	HP_355196	TGCTGTTG/	CCACCTGG(	XM_149892	Mus musculus LOC232948 (LOC232948), mR.			2.6	SM2102	d		5
092308m3_V2MM_100520	-1.20036	83.62456	TRUE	HP_321706	TGCTGTTG/	CTGGGATC.	AK014558	RIKEN cD 4632408A20 gene	4632408A20Rik		2.13	SM2499	a		2
mPool4_V2MM_32586	-1.19886	69.72441	TRUE	HP_255464	TGCTGTTG/	GTACTCCT/	NM_133688	RIKEN cDNA 4930469P12 gene	4930469P12Rik		2.15	SM2591	h		3
mPool2_V2MM_2359	-1.19852	3.004939	TRUE	HP_225980	TGCTGTTG/	CTCGGTCA(	NM_019488	solute carrier family 2, (facilitated glucose transporter), r	Slc2a8		2.11	SM2379	h		12
092308m3_V2MM_91061	-1.19706	79.16495	TRUE	HP_312477	TGCTGTTG/	GAAAGGA/	XM_485708	similar to CD sequence BC061212			2.13	SM2470	a		1
092308m1_V2MM_144752	-1.19677	2.577834	TRUE	HP_365463	TGCTGTTG/	GGTATGAG	XM_160252	Mus musculus LOC213626 (LOC213626), mR.			2.4	SM2031	b		1
092308m3_V2MM_226545	-1.19291	73.80895	TRUE	HP_478565	TGCTGTTG/	CAAGCTCG	NM_177663	expressed sequence C80587	C80587		2.13	SM2461	b		1
mPool6_V2MM_223607	-1.19238	73.80895	TRUE	HP_475749	TGCTGTTG/	GCTGCAGT.	XM_285224	Mus musculus similar to KRAB-zinc finger protein KZF-1 [Rattus norvegicus] (LOC3287!			2.8	SM2210	h		10
mPool4_V2MM_28545	-1.19183	61.94057	TRUE	HP_251533	TGCTGTTG/	GCTCAACA	NM_144787	jumonji domain containing 2C	Jmjd2c		2.15	SM2600	h		7
mPool2_V2MM_130611	-1.19111	3.004939	TRUE	HP_351393	TGCTGTTG/	GACATCCC/	XM_146952	Mus musculus similar to groucho-related gene 1 protein (LOC235489), mRNA.			2.11	SM2362	b		7
mPool4_V2MM_162153	-1.19069	94.45693	TRUE	HP_382714	TGCTGTTG/	GCCATGGA	XM_284224	gene model 765, (NCBI)	Gm765	NA				NA	
mPool4_V2MM_134010	-1.1903	61.94057	TRUE	HP_354740	TGCTGTTG/	CCTCTTCTA	AK017143	RIKEN cDNA 5031425E22 gene	5031425E22Rik		2.14	SM2531	c		2
mPool4_V2MM_31930	-1.19029	61.94057	TRUE	HP_254819	TGCTGTTG/	GAGGAGCT	NM_172487	RIKEN cDNA B230206N24 gene	B230206N24Rik		2.15	SM2590	h		9
092308m1_V2MM_136268	-1.19008	3.004939	TRUE	HP_356988	TGCTGTTG/	CAAAGAGA	XM_151591	Mus musculus LOC213852 (LOC213852), mRNA.			2.4	SM2030	g		11
092308m1_V2MM_136268	-1.19008	3.004939	TRUE	HP_356988	TGCTGTTG/	CAAAGAGA	XM_151591	Mus musculus LOC213852 (LOC213852), mR.			2.4	SM2030	g		11
mPool4_V2MM_28035	-1.1896	61.94057	TRUE	HP_251031	TGCTGTTG/	GCAAAGAT.	NM_007954	esterase 1	Es1		2.15	SM2569	a		8
092308m3_V2MM_137426	-1.18904	61.94057	TRUE	HP_358146	TGCTGTTG/	GTCCATAT(	XM_153139	Mus musculus LOC238089 (LOC238089), mR.			2.14	SM2520	h		8
092308m3_V2MM_136806	-1.18873	83.62456	TRUE	HP_357526	TGCTGTTG/	GCTACTCA	XM_152158	Mus musculus similar to small nuclear ribonucleoprotein-associated protein N - rat (L(			2.14	SM2528	d		7
092308m3_V2MM_88671	-1.18823	92.61223	TRUE	HP_310108	TGCTGTTG/	CTCGTACA(	NM_175463	RIKEN cD A930012O16 gene	A930012O16Rik		2.13	SM2470	e		10
mPool4_V2MM_71213	-1.18799	94.45693	TRUE	HP_293107	TGCTGTTG/	CATGCAAA(	NM_009766	bombesin-like receptor 3	Brs3		2.15	SM2569	a		10
mPool4_V2MM_202241	-1.18786	73.80895	TRUE	HP_420255	TGCTGTTG/	CAGCTAGT(	XM_136293	gene model 1302, (NCBI)	Gm1302		2.16	SM2620	f		5
092308m1_V2MM_95833	-1.18777	3.004939	TRUE	HP_317101	TGCTGTTG/	CAAATGGC.	BC059847	Rap guanine nucleotide exchange factor (GEF) 6	Rapgef6		2.6	SM2112	f		10
mPool4_V2MM_170243	-1.18719	73.80895	TRUE	HP_390738	TGCTGTTG/	CTCAGCTT(	XM_286418	Mus musculus hypothetical gene supported by AK044531 (LOC328072), mRNA.		NA				NA	
092308m1_V2MM_71080	-1.18607	3.004939	TRUE	HP_292979	TGCTGTTG/	CAGTGTCG(	NM_173440	nuclear receptor interacting protein 1	Nrip1		2.4	SM2026	h		2
mPool4_V2MM_240135	-1.18574	91.10404	TRUE	HP_491923	TGCTGTTG/	CTACCGAA	XM_142637	similar to hypothetical protein FLJ21628			2.14	SM2533	f		10
mPool4_V2MM_15649	-1.18533	91.71214	TRUE	HP_238970	TGCTGTTG/	CTGATCTAT	NM_031258	chordin-like 1	Chrdl1		2.15	SM2590	e		8
mPool4_V2MM_184423	-1.18441	81.56557	TRUE	HP_404895	TGCTGTTG/	GCCTTAAG(	XM_289196	Mus musculus LOC332829 (LOC332829), mRNA.		NA				NA	
092308m1_V2MM_176042	-1.18391	3.004939	TRUE	HP_396524	TGCTGTTG/	CCCTGAGG	AK032533	hypothetical protein 6430584L05			2.5	SM2074	b		10
092308m3_V2MM_84819	-1.18334	84.31789	TRUE	HP_306319	TGCTGTTG/	CAGGTAGT.	NM_029512	RIKEN cD 5830472M02 gene	5830472M02Rik		2.13	SM2476	d		9
mPool2_V2MM_88600	-1.18329	3.004939	TRUE	HP_310037	TGCTGTTG/	CTCAGATG(	NM_175443	ethanolamine kinase 2	Etnk2		2.6	SM2137	d		3
mPool4_V2MM_101531	-1.18307	81.56557	TRUE	HP_90480	TGCTGTTG/	CTTCTAGA(	AK034323	sterile alpha motif domain containing 4	Samd4		2.16	SM2624	e		5
mPool2_V2MM_85682	-1.18182	3.004939	TRUE	HP_307163	TGCTGTTG/	CAGCTGTC/	NM_146442	olfactory receptor 934	Olfr934		2.6	SM2143	c		12
mPool4_V2MM_33730	-1.18132	92.96229	TRUE	HP_256573	TGCTGTTG/	CAGTCCTA	NM_029270	Rho GTPase activating protein 24	Arhgap24		2.15	SM2598	f		6
mPool2_V2MM_93786	-1.18098	3.004939	TRUE	HP_315088	TGCTGTTG/	CCTCAACC(	NM_181817	G protein-coupled receptor 100	Gpr100		2.6	SM2133	f		6
092308m3_V2MM_37046	-1.17923	91.10404	TRUE	HP_259817	TGCTGTTG/	GAATGATC(	NM_181732	RIKEN cD 2610208M17 gene	2610208M17Rik		2.12	SM2427	c		9
092308m3_V2MM_94887	-1.17907	83.62456	TRUE	HP_316170	TGCTGTTG/	CCTCCTTCC	AK011447	RIKEN cD 2610018I05 gene	2610018I05Rik		2.9	SM2268	e		3
092308m3_V2MM_94887	-1.17907	83.62456	TRUE	HP_316170	TGCTGTTG/	CCTCCTTCC	AK011447	RIKEN cDNA 2610018I05 gene	2610018I05Rik		2.9	SM2268	e		3
mPool5_V2MM_164042	-1.17874	94.45693	TRUE	HP_384592	TGCTGTTG/	CATCCTCCC	XM_284909	Mus musculus similar to env protein [Homo sapiens] (LOC327892), mRNA.		NA				NA	
mPool6_V2MM_64870	-1.17774	66.95036	TRUE	HP_286918	TGCTGTTG/	GAAGTATT(	NM_011308	nuclear receptor co-repressor 1	Ncor1		2.8	SM2222	a		8
092308m3_V2MM_202196	-1.177	84.31789	TRUE	HP_420215	TGCTGTTG/	CTGGGAAG	AK011120	RIKEN cD 2510049J12 gene	2510049J12Rik		2.13	SM2491	h		9
092308m3_V2MM_46348	-1.17698	94.45693	TRUE	HP_268855	TGCTGTTG/	CCATTGCTT	NM_009431	SH2 domain binding protein 1 (tetratricopeptide repeat (	Sh2bp1		2.12	SM2438	f		12
092308m3_V2MM_204092	-1.17682	61.94057	TRUE	HP_422007	TGCTGTTG/	CCATCCATA	XM_131867	cD sequence BC025458	BC025458		2.13	SM2489	g		11
mPool6_V2MM_156258	-1.17626	94.45693	TRUE	HP_376897	TGCTGTTG/	CGCATCTC(	XM_198245	Mus musculus LOC272635 (LOC272635), mRNA.			2.8	SM2209	f		10
mPool6_V2MM_167461	-1.17543	91.71214	TRUE	HP_387974	TGCTGTTG/	GTACCAAA	XM_285772	Mus musculus similar to sterol carrier protein 2, liver [Mus musculus] (LOC329779), m			2.8	SM2211	e		8
mPool4_V2MM_227942	-1.17502	70.87591	TRUE	HP_479932	TGCTGTTG/	CTATTCCTA	XM_287089	Mus musculus hypothetical gene supported by BC039973 (LOC329350), mRNA.			2.16	SM2649	b		11
092308m3_V2MM_110848	-1.17278	94.45693	TRUE	HP_331874	TGCTGTTG/	GAGATGCA	XM_135077	expressed sequence AV228068	AV228068		2.9	SM2300	e		2
092308m3_V2MM_110848	-1.17278	94.45693	TRUE	HP_331874	TGCTGTTG/	GAGATGCA	XM_135077	expressed sequence AV228068	AV228068		2.9	SM2300	e		2

mPool4_V2MM_163041	-1.17229	84.31789	TRUE	HP_383597	TGCTGTTG/	CAAACTCT(	XM_284617	calcium channel, voltage-dependent, gamma subunit 7	Cacng7	NA				NA	
092308m3_V2MM_195907	-1.17176	94.45693	TRUE	HP_414026	TGCTGTTG/	GCCATATG	NM_146436	olfactory receptor 998	Olfr998		2.9	SM2268	g		5
092308m3_V2MM_195907	-1.17176	94.45693	TRUE	HP_414026	TGCTGTTG/	GCCATATG	NM_146436	olfactory receptor 998	Olfr998		2.9	SM2268	g		5
092308m3_V2MM_141759	-1.17157	91.71214	TRUE	HP_362472	TGCTGTTG/	GTCAGCTA(	XM_156950	Mus musculus LOC225066 (LOC225066), mR.			2.14	SM2514	a		9
mPool4_V2MM_15548	-1.17108	84.31789	TRUE	HP_238869	TGCTGTTG/	CTGAATAT(	NM_053228	vomeronasal 1 receptor, B7	V1rb7		2.15	SM2598	e		1
mPool4_V2MM_194280	-1.17107	94.45693	TRUE	HP_412454	TGCTGTTG/	GGAATTGT	NM_177887	cDNA sequence BC022651	BC022651		2.16	SM2620	d		5
mPool2_V2MM_109324	-1.17066	3.004939	TRUE	HP_330385	TGCTGTTG/	GGAACTGT	XM_355941	RIKEN cDNA 1110033K02 gene	1110033K02Rik		2.1	SM2308	g		10
092308m3_V2MM_203551	-1.17024	83.62456	TRUE	HP_421497	TGCTGTTG/	CAGTGTAT/	AK010407	zinc finger protein 511	Zfp511		2.13	SM2496	h		1
092308m3_V2MM_50809	-1.17022	92.61223	TRUE	HP_273218	TGCTGTTG/	CGACCCAT(	NM_001005524	olfactory receptor 194	Olfr194		2.12	SM2438	h		9
mPool4_V2MM_160529	-1.16995	70.87591	TRUE	HP_381108	TGCTGTTG/	GCACTTAT/	AK076800	hypothetical gene supported by AK076800; BC048609		NA				NA	
mPool2_V2MM_141850	-1.16763	3.004939	TRUE	HP_362563	TGCTGTTG/	GGTCTAGG	XM_157056	Mus musculus LOC209953 (LOC209953), mRNA.			2.4	SM2048	d		11
mPool2_V2MM_141850	-1.16763	3.004939	TRUE	HP_362563	TGCTGTTG/	GGTCTAGG	XM_157056	Mus musculus LOC209953 (LOC209953), mR.			2.4	SM2048	d		11
mPool6_V2MM_223173	-1.16676	75.98381	TRUE	HP_475354	TGCTGTTG/	CTAACATC	XM_284686	RIKEN cDNA 1700023I07 gene	1700023I07Rik		2.7	SM2197	d		2
mPool6_V2MM_63704	-1.16646	92.61223	TRUE	HP_285778	TGCTGTTG/	CTATTTCCG	NM_172974	COP9 (constitutive photomorphogenic) homolog, subuni	Cops7b		2.8	SM2217	d		10
mPool6_V2MM_78301	-1.16612	94.45693	TRUE	HP_299993	TGCTGTTG/	GACATTTC/	NM_025692	ubiquitin-activating enzyme E1-domain containing 1	Ube1dc1		2.8	SM2220	h		2
092308m3_V2MM_50010	-1.16555	61.94057	TRUE	HP_272441	TGCTGTTG/	CACCATGA(	NM_029299	RIKEN cD 1700001L23 gene	1700001L23Rik		2.12	SM2411	c		6
mPool4_V2MM_183952	-1.16547	79.16495	TRUE	HP_404426	TGCTGTTG/	GAGTATCC/	XM_289110	Mus musculus LOC333602 (LOC333602), mRNA.		NA				NA	
092308m3_V2MM_31772	-1.16539	92.61223	TRUE	HP_254665	TGCTGTTG/	GAAGTAAA	NM_027432	RIKEN cD 2610312E17 gene	2610312E17Rik		2.11	SM2400	g		9
mPool6_V2MM_67828	-1.16535	61.94057	TRUE	HP_289804	TGCTGTTG/	CTCCGAGT/	NM_011010	olfactory marker protein	Omp		2.8	SM2225	c		6
092308m3_V2MM_43199	-1.16487	91.71214	TRUE	HP_265792	TGCTGTTG/	CTATTGTTA	NM_177329	RIKEN cD A530053G22 gene	A530053G22Rik		2.12	SM2442	g		9
mPool4_V2MM_186155	-1.16405	91.10404	TRUE	HP_406627	TGCTGTTG/	CTCATCAA/	XM_289491	Mus musculus LOC333197 (LOC333197), mRNA.		NA				NA	
092308m3_V2MM_56273	-1.16338	94.45693	TRUE	HP_278557	TGCTGTTG/	CTCACCTG	NM_172529	expressed sequence AU067744	AU067744		2.12	SM2441	a		6
mPool6_V2MM_62092	-1.1631	84.31789	TRUE	HP_284207	TGCTGTTG/	CACCTTGA	NM_025511	RIKEN cDNA 2310005N03 gene	2310005N03Rik		2.8	SM2220	a		1
092308m3_V2MM_30944	-1.16256	83.62456	TRUE	HP_253855	TGCTGTTG/	CTCCATTA(	BC038377	RIKEN cD 4933426L22 gene	4933426L22Rik		2.12	SM2422	d		6
mPool4_V2MM_206630	-1.16233	61.94057	TRUE	HP_424450	TGCTGTTG/	CATTTCGC(	XM_155119	Mus musculus LOC239254 (LOC239254), mRNA.			2.16	SM2645	h		5
mPool4_V2MM_47886	-1.15966	83.62456	TRUE	HP_270354	TGCTGTTG/	GAAGTAAT.	NM_138587	DNA segment, Chr 6, Wayne State University 176, expres	D6Wsu176e		2.15	SM2588	b		11
mPool4_V2MM_65957	-1.15959	61.94057	TRUE	HP_287986	TGCTGTTG/	GTCTCAGT(	NM_026255	solute carrier family 25 (mitochondrial carrier, phosphat	Slc25a26		2.15	SM2594	g		8
mPool4_V2MM_210432	-1.15913	92.61223	TRUE	HP_428184	TGCTGTTG/	CACCCTGA	XM_487875	similar to Heat shock cognate 71 kDa protein			2.14	SM2538	a		7
mPool6_V2MM_163262	-1.15683	61.94057	TRUE	HP_383817	TGCTGTTG/	GAGACCAA	AK016974	RIKEN cDNA 4933428M09 gene	4933428M09Rik		2.7	SM2190	h		2
092308m3_V2MM_42626	-1.15605	75.98381	TRUE	HP_265230	TGCTGTTG/	CCTTCTATA	NM_172647	F11 receptor	F11r		2.12	SM2407	f		6
mPool6_V2MM_161264	-1.15437	92.61223	TRUE	HP_381835	TGCTGTTG/	CAGTTATA(	XM_283830	TRAF2 and NCK interacting kinase	Tnik		2.7	SM2193	b		1
mPool4_V2MM_32090	-1.15413	92.61223	TRUE	HP_254978	TGCTGTTG/	GCCACTGC(	NM_172478	ornithine aminotransferase-like 1	Oatl1	NA				NA	
092308m1_V2MM_137990	-1.15407	3.004939	TRUE	HP_358710	TGCTGTTG/	CTGTGTGG.	XM_153775	Mus musculus LOC238452 (LOC238452), mR.			2.4	SM2049	e		5
092308m1_V2MM_137990	-1.15407	3.004939	TRUE	HP_358710	TGCTGTTG/	CTGTGTGG.	XM_153775	Mus musculus LOC238452 (LOC238452), mRNA.			2.4	SM2049	e		5
mPool4_V2MM_10538	-1.15341	61.94057	TRUE	HP_233968	TGCTGTTG/	CTCTGGAT/	NM_027204	mitochondrial ribosomal protein L12	Mrpl12		2.15	SM2592	c		10
mPool2_V2MM_124343	-1.15137	3.004939	TRUE	HP_345195	TGCTGTTG/	CATCTGGA.	XM_143390	Mus musculus similar to ribosomal protein L13; 60S ribosomal protein L13; breast bas			2.11	SM2372	g		2
mPool4_V2MM_61465	-1.15076	84.31789	TRUE	HP_283599	TGCTGTTG/	GTGTTTAA	NM_172611	cDNA sequence BC021523	BC021523		2.15	SM2587	h		10
mPool4_V2MM_202335	-1.15048	91.71214	TRUE	HP_420345	TGCTGTTG/	GGCTTCTA(	XM_138942	potassium channel, subfamily K, member 16	Kcnk16		2.16	SM2614	b		6
mPool6_V2MM_67581	-1.14969	70.87591	TRUE	HP_289562	TGCTGTTG/	CGGCCTTT(	NM_053094	CD163 antigen	Cd163		2.8	SM2223	b		3
mPool4_V2MM_167754	-1.14947	70.87591	TRUE	HP_388267	TGCTGTTG/	CCTATATG	XM_285832	Mus musculus similar to cyclase-associated protein homologue [Rattus norvegicus] (L(		NA				NA	
mPool6_V2MM_65889	-1.14862	94.45693	TRUE	HP_287918	TGCTGTTG/	GTCACATG.	NM_013918	ubiquitin specific protease 25	Usp25		2.7	SM2176	b		7
mPool7_V2MM_194256	-1.14707	3.004939	TRUE	HP_412430	TGCTGTTG/	CGTTACAG	NM_178674	F-box and leucine-rich repeat protein 21	Fbxl21		2.9	SM2253	g		10
mPool4_V2MM_109076	-1.14579	61.94057	TRUE	HP_330142	TGCTGTTG/	CAGATAGT(	NM_007461	amyloid beta (A4) precursor protein-binding, family A, m	Apba2		2.16	SM2617	a		11
mPool4_V2MM_216973	-1.14533	84.31789	TRUE	HP_450079	TGCTGTTG/	CTATCTACC	NM_010162	exostoses (multiple) 1	Ext1		2.15	SM2575	a		1
092308m1_V2MM_149737	-1.14337	3.004939	TRUE	HP_370447	TGCTGTTG/	CAGCACAT(	XM_165151	similar to 60S ribosomal protein L5			2.4	SM2039	c		7
092308m1_V2MM_149737	-1.14337	3.004939	TRUE	HP_370447	TGCTGTTG/	CAGCACAT(	XM_165151	similar to 60S ribosomal protein L5			2.4	SM2039	c		7
092308m1_V2MM_145550	-1.14249	2.577834	TRUE	HP_366261	TGCTGTTG/	CAATGTAG.	XM_161053	Mus musculus LOC230170 (LOC230170), mRNA.			2.4	SM2047	a		8
092308m1_V2MM_145550	-1.14249	2.577834	TRUE	HP_366261	TGCTGTTG/	CAATGTAG.	XM_161053	Mus musculus LOC230170 (LOC230170), mR.			2.4	SM2047	a		8
mPool7_V2MM_112924	-1.14114	3.004939	TRUE	HP_333916	TGCTGTTG/	GTGACAGA	XM_136719	Mus musculus LOC213721 (LOC213721), mRNA.			2.1	SM2321	a		8
092308m3_V2MM_82257	-1.14099	94.45693	TRUE	HP_303837	TGCTGTTG/	CGGCCAGG	NM_007736	procollagen, type IV, alpha 5	Col4a5		2.7	SM2168	c		12
092308m3_V2MM_82257	-1.14099	94.45693	TRUE	HP_303837	TGCTGTTG/	CGGCCAGG	NM_007736	procollagen, type IV, alpha 5	Col4a5		2.7	SM2168	c		12
092308m3_V2MM_147982	-1.14091	84.31789	TRUE	HP_368693	TGCTGTTG/	CCCAAAGT/	XM_163384	Mus musculus LOC244043 (LOC244043), mRNA.			2.1	SM2336	b		9

092308m3_V2MM_147982	-1.14091	84.31789	TRUE	HP_368693	TGCTGTTG/	CCCAAAGT/	XM_163384	Mus musculus LOC244043 (LOC244043), mR.			2.1	SM2336	b		9
092308m3_V2MM_48412	-1.14055	83.62456	TRUE	HP_270873	TGCTGTTG/	GATCACAT(	NM_029013	RIKEN cD 4933428M03 gene	4933428M03Rik		2.12	SM2445	f		4
mPool2_V2MM_212554	-1.13935	3.004939	TRUE	HP_430220	TGCTGTTG/	GGTCCCAC1	XM_146377	similar to 40S ribosomal protein S7 (S8)			2.1	SM2349	e		6
mPool6_V2MM_169113	-1.13831	83.62456	TRUE	HP_389613	TGCTGTTG/	CTCAGTTA(	XM_286141	similar to High mobility group protein 2 (HMG-2)			2.7	SM2192	d		4
mPool2_V2MM_120454	-1.13675	3.004939	TRUE	HP_341350	TGCTGTTG/	GTAAACAA1	XM_357189	similar to basic transcription factor 3			2.11	SM2353	h		1
092308m3_V2MM_45381	-1.13625	94.45693	TRUE	HP_267915	TGCTGTTG/	GTCGCATA1	NM_020614	TATA box binding protein (Tbp)-associated factor, R poly	Taf1b		2.12	SM2410	f		6
mPool4_V2MM_2704	-1.13459	91.71214	TRUE	HP_226317	TGCTGTTG/	CTGCTATT/	NM_153063	zinc finger protein 472	Zfp472		2.15	SM2598	g		12
mPool6_V2MM_168290	-1.13311	83.62456	TRUE	HP_388796	TGCTGTTG/	GAGAGATT	XM_285952	Mus musculus similar to interferon gamma inducible protein 30; lysosomal thiol reduc			2.7	SM2199	e		8
mPool4_V2MM_242264	-1.13198	61.94057	TRUE	HP_494011	TGCTGTTG/	CCATTCTCT	XM_140965	Mus musculus similar to constitutive photomorphogenic protein 1 [Mus musculus] (L(			2.14	SM2549	f		7
mPool4_V2MM_134038	-1.13116	92.96229	TRUE	HP_354768	TGCTGTTG/	GAACTAAC1	NM_001002765	RIKEN cDNA E130018O15 gene	E130018O15Rik		2.14	SM2543	c		2
mPool6_V2MM_223466	-1.13099	91.71214	TRUE	HP_475619	TGCTGTTG/	CTGGAGAC	XM_284914	Mus musculus similar to single-strand DNA binding protein [Rattus norvegicus] (LOC3:			2.7	SM2197	f		7
mPool6_V2MM_62966	-1.13072	69.72441	TRUE	HP_285058	TGCTGTTG/	CCAGTTCT(	NM_008080	UDP-N-acetyl-alpha-D-galactosamine:(N-acetylneuramin	Galgt1		2.8	SM2214	g		7
092308m3_V2MM_205067	-1.12995	94.45693	TRUE	HP_422944	TGCTGTTG/	CTCTCCTCC	XM_137300	Mus musculus similar to cytochrome c oxidase subunit VIIc [Mus musculus] (LOC2374			2.1	SM2306	h		9
092308m3_V2MM_205067	-1.12995	94.45693	TRUE	HP_422944	TGCTGTTG/	CTCTCCTCC	XM_137300	Mus musculus similar to cytochrome c oxidase subunit VIIc [Mus musculus] (LOC2374			2.1	SM2306	h		9
092308m3_V2MM_225216	-1.12851	61.94057	TRUE	HP_477270	TGCTGTTG/	GAAGATAT.	XM_109910	similar to hepatitis A virus cellular receptor 1; T-cell immunoglobulin and mucin doma			2.13	SM2467	b		6
mPool6_V2MM_157354	-1.12674	73.80895	TRUE	HP_377975	TGCTGTTG/	CTTCTACTA	XM_358716	similar to hypothetical protein FLJ36601			2.7	SM2192	h		3
mPool4_V2MM_90738	-1.12612	61.94057	TRUE	HP_312154	TGCTGTTG/	CGGACCCT1	NM_177849	hypothetical protein A630008I04			2.16	SM2610	e		11
mPool2_V2MM_142390	-1.12499	3.004939	TRUE	HP_363103	TGCTGTTG/	GCCAGAAG	XM_157619	Mus musculus LOC212132 (LOC212132), mRNA.			2.5	SM2053	c		11
mPool2_V2MM_142390	-1.12499	3.004939	TRUE	HP_363103	TGCTGTTG/	GCCAGAAG	XM_157619	Mus musculus LOC212132 (LOC212132), mR.			2.5	SM2053	c		11
092308m1_V2MM_98998	-1.12496	3.004939	TRUE	HP_320214	TGCTGTTG/	CTAACTGA(	AK122468	pleckstrin homology domain containing, family G (with R	Plekhg1		2.6	SM2112	a		11
092308m3_V2MM_88104	-1.12454	94.45693	TRUE	HP_309550	TGCTGTTG/	CCCTCTACA	NM_175321	RIKEN cD 4931414L13 gene	4931414L13Rik		2.13	SM2474	g		1
mPool4_V2MM_211824	-1.1241	61.94057	TRUE	HP_429509	TGCTGTTG/	GACTCCAT(	AK038838	RIKEN cDNA A230067G21 gene	A230067G21Rik		2.16	SM2640	d		2
mPool4_V2MM_8790	-1.12372	94.45693	TRUE	HP_232270	TGCTGTTG/	CAAAGAAA	XM_142190	Mus musculus similar to argonaute 5 protein [Mus musculus] (LOC245644), mRNA.			2.15	SM2586	c		4
mPool6_V2MM_64724	-1.12339	66.95036	TRUE	HP_286774	TGCTGTTG/	CTTGACCT(	NM_173402	regulator of G-protein signaling 12	Rgs12		2.8	SM2223	c		1
mPool5_V2MM_180307	-1.12306	92.61223	TRUE	HP_400781	TGCTGTTG/	CACTCGAT1	XM_288485	Mus musculus LOC331838 (LOC331838), mRNA.		NA				NA	
mPool4_V2MM_125223	-1.12185	79.16495	TRUE	HP_346067	TGCTGTTG/	CTAATTGT(	XM_143858	similar to Elongation factor 1-alpha 1 (EF-1-alpha-1) (Elongation factor 1 A-1) (eEF1A-1			2.14	SM2549	g		1
092308m1_V2MM_169056	-1.12162	3.004939	TRUE	HP_389556	TGCTGTTG/	CCTTCCTG(	XM_286128	RIKEN cD 8430439C15 gene	8430439C15Rik		2.6	SM2119	a		1
mPool4_V2MM_193877	-1.12159	61.94057	TRUE	HP_412138	TGCTGTTG/	CTCATGTG(	XM_355325	gene model 998, (NCBI)	Gm998	NA				NA	
mPool4_V2MM_238613	-1.12121	84.31789	TRUE	HP_490441	TGCTGTTG/	CATATACA/	NM_001003915	solute carrier family 5 (sodium/glucose cotransporter), n	Slc5a12		2.14	SM2538	f		5
092308m3_V2MM_47832	-1.12101	61.94057	TRUE	HP_270301	TGCTGTTG/	GAAATTGA(	NM_016688	programmed cell death protein 7	Pdcd7		2.12	SM2446	f		6
mPool2_V2MM_93782	-1.11898	3.004939	TRUE	HP_315084	TGCTGTTG/	GACAGCCT.	NM_181816	RIKEN cDNA 4933401K09 gene	4933401K09Rik		2.6	SM2140	c		1
mPool2_V2MM_210543	-1.11884	3.004939	TRUE	HP_428291	TGCTGTTG/	CCCAGATT(	AK017962	RIKEN cDNA 5830432E09 gene	5830432E09Rik		2.11	SM2358	d		2
mPool4_V2MM_121327	-1.11739	91.10404	TRUE	HP_342209	TGCTGTTG/	GAAGAAAG	XM_141603	Mus musculus similar to glyceraldehyde-3-phosphate dehydrogenase [Meriones ungu			2.16	SM2648	c		12
mPool4_V2MM_125806	-1.11644	91.10404	TRUE	HP_346649	TGCTGTTG/	CTAAGACT/	XM_144238	Mus musculus similar to protease [Mus musculus] (LOC211321), mRNA.			2.14	SM2543	f		3
092308m3_V2MM_84004	-1.11643	94.45693	TRUE	HP_305524	TGCTGTTG/	CACGCCTG(	AK031571	cell division cycle associated 8	Cdca8		2.13	SM2471	h		12
092308m3_V2MM_54737	-1.11625	91.71214	TRUE	HP_277054	TGCTGTTG/	CCACTTATT	XM_485778	similar to immunoglobulin light chain variable region			2.12	SM2413	b		3
mPool4_V2MM_122450	-1.11608	84.31789	TRUE	HP_343316	TGCTGTTG/	GGTATAGA	XM_142198	Mus musculus similar to actin (LOC209541), mRNA.			2.11	SM2373	g		6
mPool6_V2MM_166975	-1.11602	91.10404	TRUE	HP_387497	TGCTGTTG/	GTGATACA(	XM_285645	Mus musculus similar to hypothetical protein FLJ10891 [Homo sapiens] (LOC331229),			2.7	SM2190	f		12
092308m3_V2MM_106827	-1.11556	61.94057	TRUE	HP_327920	TGCTGTTG/	GAGGAAAC	XM_131568	hypothetical protein LOC230595			2.14	SM2503	g		7
mPool4_V2MM_206616	-1.11493	75.98381	TRUE	HP_424437	TGCTGTTG/	GAGCTGCT.	XM_163663	Mus musculus LOC209415 (LOC209415), mRNA.			2.16	SM2647	a		5
mPool4_V2MM_123202	-1.11482	91.10404	TRUE	HP_344058	TGCTGTTG/	CTGTTGGA(	XM_488264	similar to hypothetical protein FLJ38281			2.15	SM2551	b		5
mPool6_V2MM_160295	-1.11426	91.10404	TRUE	HP_380877	TGCTGTTG/	GATCTAAA(	XM_484638	hypothetical gene supported by AK089217			2.7	SM2188	b		12
mPool4_V2MM_55261	-1.1132	66.95036	TRUE	HP_277563	TGCTGTTG/	CCCTTTCCT	NM_146305	olfactory receptor 420	Olfr420		2.15	SM2600	h		12
mPool4_V2MM_26347	-1.11252	94.45693	TRUE	HP_249396	TGCTGTTG/	CCTTTGCTC	NM_146206	two pore segment channel 2	Tpcn2	NA				NA	
mPool4_V2MM_167738	-1.11179	91.10404	TRUE	HP_388251	TGCTGTTG/	CTTTCCAGT	XM_285828	similar to RIKEN cDNA 4732496O08		NA				NA	
mPool4_V2MM_57154	-1.11173	94.45693	TRUE	HP_279417	TGCTGTTG/	CTGTGTTTC	BC056964	RIKEN cDNA 4930553M18 gene	4930553M18Rik		2.15	SM2598	a		1
mPool4_V2MM_133428	-1.1113	61.94057	TRUE	HP_354168	TGCTGTTG/	GAATATTT(	BC072638	RIKEN cDNA E130013N09 gene	E130013N09Rik		2.16	SM2637	f		5
mPool4_V2MM_142311	-1.11109	61.94057	TRUE	HP_363024	TGCTGTTG/	GACATGTT(	XM_157563	Mus musculus LOC211517 (LOC211517), mRNA.			2.16	SM2641	g		5
mPool6_V2MM_163740	-1.11104	70.87591	TRUE	HP_384295	TGCTGTTG/	CCAGGACC(	XM_284845	RIKEN cDNA 4930513F16 gene	4930513F16Rik		2.7	SM2195	c		2
mPool6_V2MM_161433	-1.11076	92.61223	TRUE	HP_382002	TGCTGTTG/	GAGAGATA	AK036551	hypothetical protein 9830132P13			2.7	SM2188	c		8
mPool4_V2MM_130738	-1.11039	84.31789	TRUE	HP_351516	TGCTGTTG/	CGCTCAGC/	XM_147006	Mus musculus similar to extracellular matrix protein papilin [Drosophila melanogaster			2.14	SM2549	h		9
mPool4_V2MM_161281	-1.10928	92.61223	TRUE	HP_381852	TGCTGTTG/	CTGCAAAT(	XM_283835	Mus musculus hypothetical gene supported by AK043341 (LOC329611), mRNA.		NA				NA	

mPool4_V2MM_11751	-1.10859	94.45693	TRUE	HP_235160	TGCTGTTG/	GCTTTATA/	NM_145367	thioredoxin domain containing 5	Txndc5		2.15	SM2581	a		9
mPool4_V2MM_242002	-1.10677	84.31789	TRUE	HP_493754	TGCTGTTG/	GGAATCAT/	AK006834	RIKEN cDNA 1700058M13 gene	1700058M13Rik		2.14	SM2539	c		8
mPool4_V2MM_60660	-1.10668	91.71214	TRUE	HP_282811	TGCTGTTG/	GGTTCATC1	NM_146341	olfactory receptor 1259	Olfr1259		2.15	SM2588	g		1
mPool4_V2MM_331	-1.10664	92.61223	TRUE	HP_224003	TGCTGTTG/	CACATCAG/	NM_026498	mitochondrial ribosomal protein S11	Mrps11		2.15	SM2592	f		3
mPool4_V2MM_57357	-1.10662	87.58579	TRUE	HP_279613	TGCTGTTG/	CTTGTTCCT	NM_146567	olfactory receptor 843	Olfr843		2.15	SM2587	b		11
mPool4_V2MM_19223	-1.10651	84.31789	TRUE	HP_242452	TGCTGTTG/	GGCATTAT(	NM_012012	exonuclease 1	Exo1		2.15	SM2569	f		3
mPool4_V2MM_165318	-1.10604	61.94057	TRUE	HP_385855	TGCTGTTG/	GAAAGAAT	XM_285225	zinc finger protein 206	Zfp206		2.16	SM2649	d		6
mPool2_V2MM_152825	-1.10451	3.004939	TRUE	HP_373487	TGCTGTTG/	GGGACCAG	XM_195572	Mus musculus similar to large subunit ribosomal protein	L36a [Rattus norvegicus] (LO(		2.6	SM2126	b		11
092308m3_V2MM_60764	-1.10438	91.71214	TRUE	HP_282914	TGCTGTTG/	GTAGAGAT	BC023914	expressed sequence AI315068	AI315068		2.12	SM2402	d		10
092308m3_V2MM_106505	-1.10434	94.45693	TRUE	HP_327605	TGCTGTTG/	CTACTGAA(	AK011611	RIKEN cD 2610029I01 gene	2610029I01Rik		2.13	SM2487	b		3
mPool4_V2MM_124287	-1.10327	61.94057	TRUE	HP_345139	TGCTGTTG/	GCATTGTC1	NM_207278	tigger transposable element derived 4	Tigd4		2.14	SM2539	a		3
mPool4_V2MM_222434	-1.10301	70.87591	TRUE	HP_474698	TGCTGTTG/	CCTGCTCC/	XM_196881	Mus musculus LOC270881 (LOC270881), mRNA.		NA				NA	
mPool4_V2MM_128833	-1.10227	94.45693	TRUE	HP_349646	TGCTGTTG/	CTTATGATT	XM_145937	Mus musculus similar to Glyceraldehyde 3-phosphate dehydrogenase (GAPDH) (LOC2			2.16	SM2633	f		7
mPool4_V2MM_230502	-1.10114	61.94057	TRUE	HP_482487	TGCTGTTG/	GGTGTACC/	AK052790	hypothetical protein D730003B17			2.16	SM2650	c		2
mPool4_V2MM_262501	-1.10086	91.10404	TRUE	HP_523031	TGCTGTTG/	CCTACATCT	NM_183166	hypothetical protein 4930549O06			2.16	SM2620	b		8
mPool6_V2MM_163301	-1.10041	62.64426	TRUE	HP_383856	TGCTGTTG/	CAAATTAA1	AK038641	RIKEN cDNA A230052C13 gene	A230052C13Rik		2.8	SM2208	f		9
092308m3_V2MM_90177	-1.09978	79.16495	TRUE	HP_311601	TGCTGTTG/	GTTATCCTT	NM_177748	killer immunoglobulin-like receptor-like 2			2.9	SM2259	d		7
092308m3_V2MM_90177	-1.09978	79.16495	TRUE	HP_311601	TGCTGTTG/	GTTATCCTT	NM_177748	killer immunoglobulin-like receptor-like 2			2.9	SM2259	d		7
mPool4_V2MM_197044	-1.09976	92.61223	TRUE	HP_415130	TGCTGTTG/	GGAACATG	NM_026851	mitochondrial ribosomal protein L52	Mrpl52		2.15	SM2589	b		10
mPool2_V2MM_161333	-1.09943	3.004939	TRUE	HP_381903	TGCTGTTG/	CAAAGAAG	AK053020	hypothetical protein D930017F01			2.6	SM2128	g		7
092308m3_V2MM_39079	-1.09907	61.94057	TRUE	HP_261777	TGCTGTTG/	GCTATCAA(	NM_146025	cD sequence BC034054	BC034054		2.11	SM2398	f		3
mPool2_V2MM_147547	-1.09889	3.004939	TRUE	HP_368258	TGCTGTTG/	GAATGTTA(	XM_163078	Mus musculus LOC243797 (LOC243797), mRNA.			2.1	SM2333	e		5
mPool4_V2MM_48431	-1.09881	75.98381	TRUE	HP_270891	TGCTGTTG/	GATCTATA1	NM_031878	SWI/SNF related, matrix associated, actin dependent reg	Smarcd2		2.15	SM2588	e		9
mPool7_V2MM_209029	-1.09832	2.577834	TRUE	HP_426806	TGCTGTTG/	GCTCTCCA1	XM_194892	Mus musculus similar to vomeronasal 1 receptor, I3 [Mus musculus] (LOC268633), mF			2.1	SM2329	f		10
mPool7_V2MM_197460	-1.09798	3.004939	TRUE	HP_415530	TGCTGTTG/	GCCTTTCTA	NM_175523	RIKEN cDNA A930026L03 gene	A930026L03Rik		2.9	SM2266	a		9
mPool7_V2MM_80810	-1.0964	3.004939	TRUE	HP_302434	TGCTGTTG/	GGCAAGAA	NM_175308	MOB1, Mps One Binder kinase activator-like 2C (yeast)	Mobkl2c		2.8	SM2233	a		3
mPool4_V2MM_9776	-1.09626	83.62456	TRUE	HP_233226	TGCTGTTG/	CATTTATA/	NM_027279	RIKEN cDNA 2810422O20 gene	2810422O20Rik		2.15	SM2583	f		11
mPool7_V2MM_204674	-1.09548	3.004939	TRUE	HP_422566	TGCTGTTG/	GAGACTCA(	XM_135584	hypothetical LOC236262			2.1	SM2317	e		7
mPool4_V2MM_132161	-1.09499	83.62456	TRUE	HP_352915	TGCTGTTG/	CCTCTACTA	AK048159	mitogen activated protein kinase 1	Mapk1		2.14	SM2537	d		11
mPool7_V2MM_174445	-1.09406	3.004939	TRUE	HP_394930	TGCTGTTG/	GGCTAAGA	XM_488995	hypothetical gene supported by AK040598			2.9	SM2282	d		3
mPool4_V2MM_230920	-1.09252	91.10404	TRUE	HP_482905	TGCTGTTG/	CCCACTTCC	XM_287382	gene model 831, (NCBI)	Gm831	NA				NA	
092308m3_V2MM_99559	-1.09111	79.16495	TRUE	HP_320758	TGCTGTTG/	CACCTCATT	NM_178773	transmembrane protein 16D (eight membrane-spanning	Tmem16d		2.9	SM2262	h		10
092308m3_V2MM_99559	-1.09111	79.16495	TRUE	HP_320758	TGCTGTTG/	CACCTCATT	NM_178773	transmembrane protein 16D (eight membrane-spanning	Tmem16d		2.9	SM2262	h		10
092308m3_V2MM_47138	-1.0902	75.98381	TRUE	HP_269626	TGCTGTTG/	CTCAGAAG(	NM_177148	RIKEN cD B230317F23 gene	B230317F23Rik		2.12	SM2447	a		1
092308m3_V2MM_88260	-1.08921	94.45693	TRUE	HP_309702	TGCTGTTG/	CTGGGTCT(	NM_175355	RIKEN cD E330013P04 gene	E330013P04Rik		2.13	SM2460	f		4
092308m3_V2MM_88260	-1.08921	94.45693	TRUE	HP_309702	TGCTGTTG/	CTGGGTCT(	NM_175355	RIKEN cDNA E330013P04 gene	E330013P04Rik		2.13	SM2460	f		4
092308m3_V2MM_224981	-1.08908	62.64426	TRUE	HP_477037	TGCTGTTG/	CCCTTCCCA	NM_178606	D segment, Chr 10, University of California at Los Angele:	D10Ucla1		2.13	SM2477	h		6
mPool7_V2MM_137990	-1.08886	2.577834	TRUE	HP_358710	TGCTGTTG/	CTGTGTGG,	XM_153775	Mus musculus LOC238452 (LOC238452), mRNA.			2.4	SM2049	e		5
mPool7_V2MM_137990	-1.08886	2.577834	TRUE	HP_358710	TGCTGTTG/	CTGTGTGG,	XM_153775	Mus musculus LOC238452 (LOC238452), mR.			2.4	SM2049	e		5
092308m3_V2MM_191422	-1.08654	91.71214	TRUE	HP_263884	TGCTGTTG/	CAAGGAGT	NM_183366	a disintegrin and metalloprotease domain 28	Adam28		2.12	SM2411	a		11
mPool2_V2MM_168105	-1.08627	2.577834	TRUE	HP_388613	TGCTGTTG/	CAAGGTCA1	XM_285909	Mus musculus similar to Eukaryotic translation initiation	factor 3 subunit 6 (eIF-3 p48)		2.6	SM2129	c		3
mPool7_V2MM_171565	-1.08609	3.004939	TRUE	HP_392054	TGCTGTTG/	GCATCTAG(	AK041537	hypothetical protein A630020A06			2.5	SM2077	e		4
mPool7_V2MM_171565	-1.08609	3.004939	TRUE	HP_392054	TGCTGTTG/	GCATCTAG(	AK041537	hypothetical protein A630020A06			2.5	SM2077	e		4
mPool6_V2MM_20758	-1.08542	94.45693	TRUE	HP_243943	TGCTGTTG/	CAATGTGG1	NM_019731	expressed in non-metastatic cells 4, protein	Nme4		2.7	SM2187	c		10
mPool4_V2MM_241615	-1.08507	92.61223	TRUE	HP_493371	TGCTGTTG/	GAGTACCA(	BC052040	cDNA sequence BC052040	BC052040		2.14	SM2542	e		1
092308m3_V2MM_82769	-1.0848	94.45693	TRUE	HP_304333	TGCTGTTG/	CTGTTCTCC	NM_010314	guanine nucleotide binding protein (G protein), gamma t		10090	2.13	SM2485	b		5
mPool2_V2MM_82828	-1.08445	3.004939	TRUE	HP_304389	TGCTGTTG/	CAGCCCAA(	NM_010571	insulin receptor substrate 3	Irs3		2.6	SM2139	d		5
mPool2_V2MM_149240	-1.08441	3.004939	TRUE	HP_369950	TGCTGTTG/	GTCATCAC/	XM_164674	Mus musculus LOC209941 (LOC209941), mRNA.			2.1	SM2338	g		2
mPool4_V2MM_21116	-1.08416	92.61223	TRUE	HP_244295	TGCTGTTG/	CAGACAAT(	NM_146213	cDNA sequence BC026374	BC026374		2.15	SM2583	d		12
mPool2_V2MM_119182	-1.08262	3.004939	TRUE	HP_340104	TGCTGTTG/	CTATGTGT(	XM_140293	Mus musculus similar to 40S RIBOSOMAL PROTEIN S21	(LOC225141), mRNA.		2.11	SM2357	a		8
mPool4_V2MM_173106	-1.08197	62.64426	TRUE	HP_393592	TGCTGTTG/	GCATTCAG/	XM_286959	hypothetical LOC329092		NA				NA	
092308m3_V2MM_43319	-1.08171	91.10404	TRUE	HP_265905	TGCTGTTG/	CTCATCATT	NM_146315	olfactory receptor 62	Olfr62		2.12	SM2417	e		1

092308m3_V2MM_27431	-1.08137	94.45693	TRUE	HP_250451	TGCTGTTG/	GACAAGAA	NM_009273	sigl recognition particle 14	Srp14		2.11	SM2398	h		4
mPool4_V2MM_182128	-1.08102	73.80895	TRUE	HP_402602	TGCTGTTG/	GAGCTTCC(	XM_288791	Mus musculus LOC332252 (LOC332252), mRNA.		NA				NA	
mPool6_V2MM_63825	-1.0808	94.45693	TRUE	HP_285895	TGCTGTTG/	CTCATCTAC	NM_146865	olfactory receptor 992	Olfr992		2.8	SM2216	f		2
mPool7_V2MM_173489	-1.07809	3.004939	TRUE	HP_393975	TGCTGTTG/	GAGCACAT	AK040646	RIKEN cDNA E130008O17 gene	E130008O17Rik		2.5	SM2072	e		1
mPool7_V2MM_173489	-1.07809	3.004939	TRUE	HP_393975	TGCTGTTG/	GAGCACAT	AK040646	RIKEN cD E130008O17 gene	E130008O17Rik		2.5	SM2072	e		1
mPool4_V2MM_202311	-1.0774	61.94057	TRUE	NA	NA	NA	NA	NA	NA	NA		NA	NA	NA	
mPool4_V2MM_392	-1.07692	91.71214	TRUE	HP_224062	TGCTGTTG/	CACCGAAT(	NM_030565	cDNA sequence BC004044	BC004044		2.15	SM2582	b		10
092308m3_V2MM_59485	-1.07677	91.71214	TRUE	HP_281663	TGCTGTTG/	GCCTTTAG(	NM_028513	actin related protein M2			2.12	SM2402	c		8
mPool6_V2MM_71135	-1.0765	69.72441	TRUE	HP_293029	TGCTGTTG/	CATATACC(	NM_011343	SEC61, gamma subunit	Sec61g		2.8	SM2217	e		2
mPool4_V2MM_241316	-1.07624	61.94057	TRUE	HP_493077	TGCTGTTG/	CCTGAAGA.	XM_142033	Mus musculus similar to baculoviral IAP repeat-containing 6 [Homo sapiens] (LOC236(			2.14	SM2544	c		4
mPool4_V2MM_13345	-1.07513	73.80895	TRUE	HP_236715	TGCTGTTG/	CAGTTACT(	AK172981	cDNA sequence BC004022	BC004022		2.15	SM2588	h		6
mPool4_V2MM_241882	-1.07461	87.58579	TRUE	HP_493636	TGCTGTTG/	GAGTGTTA(	XM_143154	LOC434684			2.14	SM2533	e		7
mPool4_V2MM_183062	-1.07252	92.61223	TRUE	HP_403536	TGCTGTTG/	CAGTTATA(	XM_288953	Mus musculus LOC332601 (LOC332601), mRNA.		NA				NA	
mPool4_V2MM_230934	-1.07114	83.62456	TRUE	HP_482919	TGCTGTTG/	CAGAGAGA	AK035916	hypothetical protein 9630017O17		NA				NA	
092308m3_V2MM_207884	-1.06961	91.71214	TRUE	HP_425682	TGCTGTTG/	GTTAATAT(	XM_487654	similar to ribosomal protein L6			2.14	SM2509	b		11
mPool4_V2MM_195522	-1.06825	83.62456	TRUE	HP_413660	TGCTGTTG/	GCCATTGC1	NM_027290	minichromosome maintenance deficient 10 (S. cerevisiae	Mcm10		2.15	SM2579	e		2
mPool4_V2MM_80207	-1.06784	94.45693	TRUE	HP_301845	TGCTGTTG/	GCGTTTAT/	NM_025442	asparagine-linked glycosylation 5 homolog (yeast, dolich	Alg5		2.15	SM2584	g		4
mPool4_V2MM_148697	-1.06743	69.72441	TRUE	HP_369408	TGCTGTTG/	GTTTCTCTT	XM_164034	Mus musculus LOC244398 (LOC244398), mRNA.			2.16	SM2636	b		3
mPool4_V2MM_154219	-1.06719	61.94057	TRUE	HP_374860	TGCTGTTG/	GAGATACT(	XM_196680	LOC433292			2.16	SM2648	f		12
mPool4_V2MM_125995	-1.06658	91.71214	TRUE	HP_346838	TGCTGTTG/	CTATCTCTA	XM_487863	similar to apurinic/apyrimidinic endonuclease 2			2.16	SM2628	h		7
mPool4_V2MM_26609	-1.06632	84.31789	TRUE	HP_249648	TGCTGTTG/	CTATTAACC	AF371320	brain and acute leukemia, cytoplasmic	Baalc		2.15	SM2598	h		8
mPool4_V2MM_120762	-1.06494	61.94057	TRUE	HP_341654	TGCTGTTG/	CCTGTTCCA	XM_141181	Mus musculus similar to olfactory receptor MOR225-2 [Mus musculus] (LOC228294),			2.16	SM2640	c		7
mPool7_V2MM_192349	-1.06386	2.577834	TRUE	HP_325049	TGCTGTTG/	GATGCCAG	NM_199447	expressed sequence AA408556	AA408556		2.1	SM2315	g		6
mPool4_V2MM_25595	-1.06384	73.80895	TRUE	HP_248657	TGCTGTTG/	CAGTTGGC	XM_149258	zinc finger protein 217	Zfp217		2.16	SM2643	b		7
092308m3_V2MM_147348	-1.06319	61.94057	TRUE	HP_368059	TGCTGTTG/	GACTCACC(	XM_162896	Mus musculus LOC208761 (LOC208761), mR.			2.14	SM2526	c		1
092308m3_V2MM_84648	-1.06261	91.71214	TRUE	HP_306154	TGCTGTTG/	CTGACTAT/	NM_028811	elongation protein 3 homolog (S. cerevisiae)	Elp3		2.13	SM2475	g		9
mPool6_V2MM_63375	-1.06196	94.45693	TRUE	HP_285456	TGCTGTTG/	CGCTTCCAT	AF074881	histone deacetylase 3	Hdac3		2.8	SM2215	b		10
mPool4_V2MM_221317	-1.06163	69.72441	TRUE	HP_454252	TGCTGTTG/	CTCATCATC	NM_146941	olfactory receptor 353	Olfr353		2.15	SM2578	a		1
mPool4_V2MM_59942	-1.05986	66.95036	TRUE	HP_282114	TGCTGTTG/	GCTTTCCTG	NM_029211	ring finger protein 121	Rnf121		2.15	SM2599	g		9
mPool2_V2MM_7196	-1.05964	3.004939	TRUE	HP_230710	TGCTGTTG/	GAGCCAAA	NM_029850	B-cell CLL/lymphoma 7A	Bcl7a		2.11	SM2386	f		3
mPool4_V2MM_197644	-1.05946	94.45693	TRUE	HP_415710	TGCTGTTG/	GACTTACTT	XM_124482	ATP synthase, H+ transporting, mitochondrial F0 comple:	Atp5l		2.16	SM2611	b		9
092308m3_V2MM_49632	-1.05942	94.45693	TRUE	HP_272070	TGCTGTTG/	GTTACTTCA	NM_016928	toll-like receptor 5	Tlr5		2.12	SM2407	h		4
mPool4_V2MM_220109	-1.05815	92.61223	TRUE	HP_453084	TGCTGTTG/	GGAAATAT(	NM_146736	olfactory receptor 491	Olfr491		2.15	SM2596	d		2
mPool4_V2MM_168161	-1.05749	79.16495	TRUE	HP_388668	TGCTGTTG/	CTAGTAGT(	XM_285921	Mus musculus similar to hypothetical protein FLJ11159 [Homo sapiens] (LOC330284),		NA				NA	
mPool4_V2MM_181834	-1.0568	94.45693	TRUE	HP_402308	TGCTGTTG/	CCTGTGGT1	XM_288742	Mus musculus LOC332186 (LOC332186), mRNA.		NA				NA	
mPool4_V2MM_102126	-1.05669	92.61223	TRUE	HP_323284	TGCTGTTG/	CACACCAT1	XM_128114	RIKEN cDNA 4930415O20 gene	4930415O20Rik		2.16	SM2609	a		2
092308m3_V2MM_29468	-1.05622	61.94057	TRUE	HP_252430	TGCTGTTG/	CACACCCA/	NM_009356	testicular serine protease 2	Tesp2		2.12	SM2433	a		4
mPool4_V2MM_43108	-1.05483	92.96229	TRUE	HP_265704	TGCTGTTG/	CTACAAAG	NM_029288	RIKEN cDNA 1700001E04 gene	1700001E04Rik		2.16	SM2604	h		11
092308m1_V2MM_154088	-1.05384	3.004939	TRUE	HP_374729	TGCTGTTG/	CGAGTACA(	XM_196480	myosin VI	Myo6		2.6	SM2123	c		2
mPool6_V2MM_154811	-1.05328	94.45693	TRUE	HP_375452	TGCTGTTG/	GACTCCGA.	XM_197020	Mus musculus LOC271072 (LOC271072), mRNA.			2.8	SM2204	c		12
mPool4_V2MM_31675	-1.05276	69.72441	TRUE	HP_254570	TGCTGTTG/	CTTGCCAA(	NM_009364	tissue factor pathway inhibitor 2	Tfpi2		2.15	SM2572	g		1
mPool4_V2MM_13901	-1.05234	91.10404	TRUE	HP_237261	TGCTGTTG/	CCCAAGTT(	NM_027063	RIKEN cDNA 1700013G24 gene	1700013G24Rik		2.15	SM2594	b		6
mPool4_V2MM_136695	-1.05203	79.16495	TRUE	HP_357415	TGCTGTTG/	GGATCATT(	XM_152040	Mus musculus LOC237418 (LOC237418), mRNA.			2.16	SM2648	c		10
mPool4_V2MM_122525	-1.05062	94.45693	TRUE	HP_343390	TGCTGTTG/	CTCCAAAT/	XM_488391	similar to forkhead box R2			2.11	SM2358	f		2
mPool6_V2MM_192732	-1.0502	81.56557	TRUE	HP_275341	TGCTGTTG/	GGATTAAG	BC052474	mitogen-activated protein kinase kinase kinase kinase 6			2.7	SM2184	g		9
mPool4_V2MM_111247	-1.04982	94.45693	TRUE	HP_332264	TGCTGTTG/	GGCTGAAA	AK015052	RIKEN cDNA 4930402K13 gene	4930402K13Rik		2.16	SM2615	c		1
092308m3_V2MM_197152	-1.0492	92.96229	TRUE	HP_415234	TGCTGTTG/	CTCAATAC/	NM_028986	zinc finger protein 336	Zfp336		2.13	SM2484	d		9
mPool2_V2MM_119312	-1.04901	3.004939	TRUE	HP_340234	TGCTGTTG/	CATTTACA/	XM_140341	similar to RIKEN cDNA 5430401O09 gene			2.11	SM2352	e		12
mPool4_V2MM_128042	-1.04832	87.58579	TRUE	HP_348860	TGCTGTTG/	GGGACATC	XM_145505	Mus musculus similar to hypothetical protein MGC45586 [Homo sapiens] (LOC233061			2.14	SM2538	e		4
092308m3_V2MM_50233	-1.04822	83.62456	TRUE	HP_272661	TGCTGTTG/	CAGACGGT	NM_029023	serine caroboxypeptidase 1	Scpep1		2.12	SM2408	d		5
mPool4_V2MM_262409	-1.04715	92.61223	TRUE	HP_524616	TGCTGTTG/	CTGGGTAC	NM_198417	RIKEN cDNA C030039L03 gene	C030039L03Rik		2.16	SM2605	a		11
mPool6_V2MM_67834	-1.04654	92.61223	TRUE	HP_289810	TGCTGTTG/	CTCCTATAC	NM_029416	zinc finger protein 393	Zfp393		2.8	SM2225	h		6

mPool6_V2MM_68015	-1.04641	84.31789	TRUE	HP_289986	TGCTGTTG/	CTGATCAA(	NM_007406	adenylate cyclase 7	Adcy7		2.8	SM2220	g		11
mPool7_V2MM_206354	-1.04543	2.577834	TRUE	HP_424181	TGCTGTTG/	CCTGGATG/	XM_136503	Mus musculus similar to Glyceraldehyde 3-phosphate dehydrogenase (GAPDH) (38 kD			2.1	SM2303	h		12
092308m1_V2MM_79639	-1.04447	3.004939	TRUE	HP_301294	TGCTGTTG/	GCCAATCA(	AK085850	RIKEN cD D830016O14 gene	D830016O14Rik		2.3	SM2001	b		11
mPool4_V2MM_112142	-1.04307	91.10404	TRUE	HP_333148	TGCTGTTG/	CTCTTGAA(	XM_136345	Mus musculus similar to Exocyst complex component Sec8 (LOC240873), mRNA.			2.16	SM2624	b		7
mPool4_V2MM_160582	-1.04191	61.94057	TRUE	HP_381161	TGCTGTTG/	CACCTTCTC	XM_283570	Mus musculus hypothetical gene supported by AK083061 (LOC329079), mRNA.			2.16	SM2650	f		12
mPool6_V2MM_44133	-1.04115	92.61223	TRUE	HP_266699	TGCTGTTG/	CTTACATCG	NM_177343	calcium/calmodulin-dependent protein kinase ID	Camk1d		2.7	SM2185	c		4
mPool4_V2MM_13935	-1.04105	83.62456	TRUE	HP_237295	TGCTGTTG/	CCCACTCTC	BC034893	RIKEN cDNA 3110037K17 gene	3110037K17Rik		2.15	SM2586	a		2
mPool4_V2MM_41094	-1.0408	92.61223	TRUE	HP_263739	TGCTGTTG/	CAAAGATC(	NM_139220	defensin beta 7	Defb7		2.15	SM2596	b		3
092308m3_V2MM_195103	-1.03972	75.98381	TRUE	HP_413252	TGCTGTTG/	GAAAGGAT	NM_153153	supervillin	Svil		2.12	SM2450	e		6
mPool4_V2MM_238407	-1.03944	94.45693	TRUE	HP_490241	TGCTGTTG/	CCTCCTTAC	XM_146661	Mus musculus similar to protease [Mus musculus] (LOC244731), mRNA.			2.14	SM2537	e		12
092308m3_V2MM_195127	-1.03931	61.94057	TRUE	HP_413276	TGCTGTTG/	CCACAATC(	NM_177628	hypothetical protein A030013D21			2.13	SM2451	b		6
mPool4_V2MM_38269	-1.0392	62.64426	TRUE	HP_260998	TGCTGTTG/	GCAAATAT(	NM_027617	spermatogenesis associated 1	Spata1		2.16	SM2603	f		1
mPool4_V2MM_169478	-1.03746	92.61223	TRUE	HP_389973	TGCTGTTG/	CTCTTCGAT	XM_286268	Mus musculus hypothetical gene supported by AK038490 (LOC327786), mRNA.		NA				NA	
mPool4_V2MM_126910	-1.03708	94.45693	TRUE	HP_347746	TGCTGTTG/	GACCTCCT/	XM_144878	similar to Glyceraldehyde 3-phosphate dehydrogenase, liver (GAPDH)			2.1	SM2329	c		1
mPool4_V2MM_131566	-1.03681	94.45693	TRUE	HP_352328	TGCTGTTG/	CAGACCTT(	XM_147716	gene model 528, (NCBI)	Gm528		2.16	SM2632	d		10
mPool4_V2MM_170226	-1.03646	84.31789	TRUE	HP_390721	TGCTGTTG/	GTCATCTG(	XM_286414	hypothetical LOC328082		NA				NA	
mPool5_V2MM_6667	-1.03628	92.61223	TRUE	NA	NA	NA	NA	NA	NA	NA	NA		NA	NA	
092308m3_V2MM_60584	-1.03603	79.16495	TRUE	HP_282737	TGCTGTTG/	GGTCCTCT(	XM_484893	similar to Diacylglycerol kise, delta (Diglyceride kise) (DGK-delta) (DAG kise delta) (13(			2.12	SM2446	f		12
mPool4_V2MM_195190	-1.03532	84.31789	TRUE	HP_413336	TGCTGTTG/	CGACCAAG	NM_146400	olfactory receptor 1288	Olfr1288		2.15	SM2582	f		6
mPool6_V2MM_9789	-1.03397	92.61223	TRUE	HP_233239	TGCTGTTG/	CATTTGCAT	NM_007700	conserved helix-loop-helix ubiquitous kinase	Chuk		2.7	SM2185	f		11
mPool4_V2MM_192555	-1.03354	83.62456	TRUE	HP_249251	TGCTGTTG/	CCTATATA/	NM_144959	SWI/SNF related, matrix associated, actin dependent reg	Smarca3		2.16	SM2602	h		11
092308m1_V2MM_70649	-1.03042	3.004939	TRUE	HP_292554	TGCTGTTG/	CACTCCAG(	AF152344	prostaglandin F2 receptor negative regulator	Ptgfrn		2.3	SM2010	e		7
mPool4_V2MM_61507	-1.03027	70.87591	TRUE	HP_283640	TGCTGTTG/	GTTATCCTG	NM_023852	RAB3C, member RAS oncogene family	Rab3c		2.15	SM2571	h		3
092308m3_V2MM_231358	-1.02993	94.45693	TRUE	HP_483331	TGCTGTTG/	CGCAAAGG	XM_128440	RIKEN cD 4930432O21 gene	4930432O21Rik		2.13	SM2487	e		3
mPool4_V2MM_124135	-1.02986	94.45693	TRUE	HP_344987	TGCTGTTG/	CCAAATAT(	XM_143316	Mus musculus similar to peptidoglycan recognition protein-I-beta precursor (LOC242(			2.1	SM2347	b		11
092308m3_V2MM_194412	-1.02963	94.45693	TRUE	HP_412576	TGCTGTTG/	CGGGTAAT	NM_177758	zinc finger protein 31	Zfp31		2.13	SM2451	g		12
mPool6_V2MM_36475	-1.02961	92.96229	TRUE	HP_259255	TGCTGTTG/	CTGTATTCC	NM_008778	p21 (CDKN1A)-activated kinase 3	Pak3		2.7	SM2186	d		9
mPool4_V2MM_153946	-1.02938	83.62456	TRUE	HP_374589	TGCTGTTG/	CCTCCTGG1	XM_196410	gene model 682, (NCBI)	Gm682	NA				NA	
mPool6_V2MM_161946	-1.02931	91.10404	TRUE	HP_382510	TGCTGTTG/	GAATTTGA/	XM_284122	Mus musculus hypothetical gene supported by AK036030 (LOC330162), mRNA.			2.7	SM2192	c		6
092308m1_V2MM_66341	-1.02841	3.004939	TRUE	HP_288358	TGCTGTTG/	CACGCCAA/	NM_026340	processing of precursor 1, ribonuclease P/MRP family, (S	Pop1		2.4	SM2024	g		1
mPool6_V2MM_53756	-1.02767	83.62456	TRUE	HP_276104	TGCTGTTG/	CACATCCG1	NM_177270	cyclin-dependent kinase-like 2 (CDC2-related kinase)	Cdkl2		2.7	SM2184	h		1
mPool4_V2MM_109969	-1.02765	92.61223	TRUE	HP_331012	TGCTGTTG/	GGCTCCTC(	XM_134253	RIKEN cDNA 9430098E02 gene	9430098E02Rik		2.16	SM2625	h		7
mPool4_V2MM_5400	-1.0271	61.94057	TRUE	HP_228951	TGCTGTTG/	CCCATCAA(	NM_153514	Rho-related BTB domain containing 2	Rhobtb2		2.15	SM2591	d		2
092308m3_V2MM_225134	-1.02703	83.62456	TRUE	HP_477188	TGCTGTTG/	GATTGGGA	XM_109346	similar to ribosomal protein			2.13	SM2458	c		8
092308m3_V2MM_31917	-1.02448	61.94057	TRUE	HP_254806	TGCTGTTG/	GAGCTAAG	NM_019787	SEC23B (S. cerevisiae)	Sec23b		2.12	SM2426	a		12
mPool4_V2MM_211180	-1.02407	92.96229	TRUE	HP_332247	TGCTGTTG/	CTCAAGAA(	XM_142922	hypothetical LOC333463			2.14	SM2531	b		2
mPool4_V2MM_125567	-1.02375	81.56557	TRUE	HP_346410	TGCTGTTG/	GGACAGCA	XM_144096	Mus musculus similar to CG17097-PB [Drosophila melanogaster] (LOC230826), mRNA			2.16	SM2641	c		3
mPool4_V2MM_177396	-1.02346	73.80895	TRUE	HP_397876	TGCTGTTG/	GCCATAGG	AK054405	hypothetical protein E330022O07		NA				NA	
092308m3_V2MM_29857	-1.02344	91.71214	TRUE	HP_178098	TGCTGTTG/	CAGCAATT1	NM_144874	COX15 homolog, cytochrome c oxidase assembly protein	Cox15		2.11	SM2399	h		8
mPool6_V2MM_40359	-1.02207	61.94057	TRUE	HP_263025	TGCTGTTG/	GTCCCACA1	XM_132928	guanylate cyclase 2c	Gucy2c		2.7	SM2182	a		5
092308m3_V2MM_206944	-1.02203	70.87591	TRUE	HP_424761	TGCTGTTG/	CATGTGGC/	XM_153803	Mus musculus LOC238472 (LOC238472), mR.			2.14	SM2526	d		6
092308m3_V2MM_232487	-1.02047	94.45693	TRUE	HP_484431	TGCTGTTG/	GCAATTCT/	XM_137107	similar to hypothetical protein MGC37588			2.14	SM2508	g		2
mPool4_V2MM_128653	-1.019	94.45693	TRUE	HP_349466	TGCTGTTG/	GTCTTTACA	XM_145830	similar to tripartite motif protein TRIM30 isoform alpha			2.14	SM2545	d		8
mPool4_V2MM_118802	-1.0182	91.71214	TRUE	HP_339732	TGCTGTTG/	GCCTGACT/	XM_140072	Mus musculus similar to transcription factor IIIA [Mus musculus] (LOC240124), mRNA			2.16	SM2643	b		6
mPool6_V2MM_153186	-1.01776	94.45693	TRUE	HP_373846	TGCTGTTG/	GACTGAGT	AK029407	RIKEN cDNA 4833431D13 gene	4833431D13Rik		2.7	SM2196	a		12
mPool4_V2MM_15891	-1.01651	83.62456	TRUE	HP_239202	TGCTGTTG/	CTGGGTCA(	NM_018857	mesothelin	Msln		2.15	SM2573	h		9
092308m3_V2MM_53619	-1.01644	61.94057	TRUE	HP_275973	TGCTGTTG/	CAAGGGCA	NM_172562	transcriptiol adaptor 2 (ADA2 homolog, yeast)-like	Tada2l		2.12	SM2437	a		5
092308m3_V2MM_116730	-1.01572	92.61223	TRUE	HP_337681	TGCTGTTG/	CATGCTAT(	XM_487339	similar to granzyme H splice variant 2			2.14	SM2501	h		5
mPool6_V2MM_66315	-1.01454	84.31789	TRUE	HP_288332	TGCTGTTG/	CACCCTTCC	NM_008001	FYVE, RhoGEF and PH domain containing 1	Fgd1		2.7	SM2176	h		8
092308m3_V2MM_111537	-1.01444	94.45693	TRUE	HP_332550	TGCTGTTG/	CAGTTCCA(	XM_135913	cD sequence BC023829	BC023829		2.14	SM2503	b		1
mPool4_V2MM_97336	-1.01443	84.31789	TRUE	HP_318578	TGCTGTTG/	CCTTACATC	XM_111450	Mus musculus similar to vomeronasal receptor V1RH19 [Mus musculus] (LOC193461),			2.16	SM2617	h		11
092308m1_V2MM_151088	-1.0129	3.004939	TRUE	HP_371761	TGCTGTTG/	CCCACAGC1	NM_027498	cD sequence BC033915	BC033915		2.4	SM2030	e		3

mPool6_V2MM_69142	-1.0106	66.95036	TRUE	HP_291081	TGCTGTTG	GCAATTAT	NM_172829	beta galactoside alpha 2,6 sialyltransferase 2	St6gal2	2.8	SM2221	a	12
mPool6_V2MM_67102	-1.01006	75.98381	TRUE	HP_122456	TGCTGTTG	CCCTGGAG	NM_008067	gamma-aminobutyric acid (GABA-A) receptor, subunit al	Gabra3	2.8	SM2220	a	3
mPool4_V2MM_107883	-1.00821	84.31789	TRUE	HP_328963	TGCTGTTG	CCACTCAG	XM_132393	RIKEN cDNA 1700016K13 gene	1700016K13Rik	2.16	SM2624	g	12
mPool4_V2MM_39325	-1.00645	69.72441	TRUE	HP_262014	TGCTGTTG	GCTTCTAC	NM_134233	vomeronasal 1 receptor, G11	V1rg11	2.15	SM2579	c	7
092308m3_V2MM_140236	-1.006	61.94057	TRUE	HP_360956	TGCTGTTG	GTGTTGCT	XM_155809	Mus musculus LOC239484 (LOC239484), mR.		2.14	SM2520	a	2
092308m3_V2MM_36248	-1.00558	73.80895	TRUE	HP_259029	TGCTGTTG	CTGCCTAA	NM_024189	YY1 associated factor 2	Yaf2	2.11	SM2396	g	8
mPool4_V2MM_122598	-1.00516	94.45693	TRUE	HP_343462	TGCTGTTG	GTCCTTAG	XM_142291	Mus musculus similar to differentiation inhibitor protein	Id2A - human (LOC237200),	2.14	SM2548	g	2
mPool6_V2MM_222672	-1.00483	94.45693	TRUE	HP_474917	TGCTGTTG	GCCTGTAC	XM_285831	similar to zinc finger, MYND domain containing 12		2.8	SM2209	c	10
092308m3_V2MM_97963	-1.00454	73.80895	TRUE	HP_319199	TGCTGTTG	CCAATTTC	XM_111999	Mus musculus similar to intracisterl A particles [Mus musculus] (LOC194861), mR.		2.13	SM2484	c	8
mPool4_V2MM_13117	-1.00387	94.45693	TRUE	HP_236493	TGCTGTTG	CAGCTCTC	NM_153151	acetyl-Coenzyme A acetyltransferase 3	Acat3	2.15	SM2600	g	7
mPool6_V2MM_163964	-1.00381	79.16495	TRUE	HP_384517	TGCTGTTG	CAGGTAGC	XM_284893	Mus musculus similar to eukaryotic translation initiation	factor 3, subunit 6 48kDa [M	2.7	SM2191	g	11
mPool4_V2MM_14535	-1.00371	94.45693	TRUE	HP_237880	TGCTGTTG	CCTGGAGA	NM_008443	kinesin family member 3A	Kif3a	2.15	SM2574	g	4
092308m3_V2MM_106066	-1.00351	92.96229	TRUE	HP_327172	TGCTGTTG	GTCATCTC	XM_355410	gene model 1015, (NCBI)	Gm1015	2.14	SM2507	a	10
092308m3_V2MM_227516	-1.00222	92.61223	TRUE	HP_479514	TGCTGTTG	CTGGGAAA	NM_181988	RAS-like, estrogen-regulated, growth-inhibitor	Rerg	2.13	SM2482	d	12
mPool4_V2MM_165848	-1.00215	91.10404	TRUE	HP_386382	TGCTGTTG	CACAGGAG	XM_285339	plakophilin 4	Pkp4	2.16	SM2649	a	3
mPool4_V2MM_212070	-1.00156	94.45693	TRUE	HP_429747	TGCTGTTG	GAGACAGA	XM_148860	armadillo repeat containing 4	Armc4	2.16	SM2647	c	4
mPool6_V2MM_160135	-1.00145	94.45693	TRUE	HP_380720	TGCTGTTG	GCAACTTA	XM_283389	Mus musculus hypothetical gene supported by AK029312 (LOC328736), mRNA.		2.7	SM2200	d	7
mPool4_V2MM_199657	-1.00126	61.94057	TRUE	HP_417697	TGCTGTTG	GGCCCAAC	XM_288545	Mus musculus LOC331931 (LOC331931), mRNA.		NA			NA
mPool2_V2MM_98049	-1.00081	2.577834	TRUE	HP_319283	TGCTGTTG	CAGAATGA	XM_112084	Mus musculus similar to RIKEN cDNA 2610028L19 (LOC195712), mRNA.		2.6	SM2141	h	7
